# A *Plasmodium* Whole-Genome Synteny Map: Indels and Synteny Breakpoints as Foci for Species-Specific Genes

**DOI:** 10.1371/journal.ppat.0010044

**Published:** 2005-12-23

**Authors:** Taco W. A Kooij, Jane M Carlton, Shelby L Bidwell, Neil Hall, Jai Ramesar, Chris J Janse, Andrew P Waters

**Affiliations:** 1 Department of Parasitology, Malaria Group, Leiden University Medical Centre, Leiden, The Netherlands; 2 The Institute for Genomic Research, Rockville, Maryland, United States of America; 3 Pathogen Sequencing Unit, The Wellcome Trust Sanger Institute, The Wellcome Trust Genome Campus, Hinxton, Cambridge, United Kingdom; Stanford University, United States of America

## Abstract

Whole-genome comparisons are highly informative regarding genome evolution and can reveal the conservation of genome organization and gene content, gene regulatory elements, and presence of species-specific genes. Initial comparative genome analyses of the human malaria parasite *Plasmodium falciparum* and rodent malaria parasites (RMPs) revealed a core set of 4,500 *Plasmodium* orthologs located in the highly syntenic central regions of the chromosomes that sharply defined the boundaries of the variable subtelomeric regions. We used composite RMP contigs, based on partial DNA sequences of three RMPs, to generate a whole-genome synteny map of *P. falciparum* and the RMPs. The core regions of the 14 chromosomes of *P. falciparum* and the RMPs are organized in 36 synteny blocks, representing groups of genes that have been stably inherited since these malaria species diverged, but whose relative organization has altered as a result of a predicted minimum of 15 recombination events. *P. falciparum*-specific genes and gene families are found in the variable subtelomeric regions (575 genes), at synteny breakpoints (42 genes), and as intrasyntenic indels (126 genes). Of the 168 non-subtelomeric *P. falciparum* genes, including two newly discovered gene families, 68% are predicted to be exported to the surface of the blood stage parasite or infected erythrocyte. Chromosomal rearrangements are implicated in the generation and dispersal of *P. falciparum*-specific gene families, including one encoding receptor-associated protein kinases. The data show that both synteny breakpoints and intrasyntenic indels can be foci for species-specific genes with a predicted role in host-parasite interactions and suggest that, besides rearrangements in the subtelomeric regions, chromosomal rearrangements may also be involved in the generation of species-specific gene families. A majority of these genes are expressed in blood stages, suggesting that the vertebrate host exerts a greater selective pressure than the mosquito vector, resulting in the acquisition of diversity.

## Synopsis

Malaria, caused by the parasite *Plasmodium falciparum,* is one of the most devastating infectious diseases. Rodent malaria parasites (RMPs), such as *P. berghei, P. chabaudi,* and *P. yoelii,* are used as models for *P. falciparum*. For the use of these models in studies of human disease, insight into both the similarities and differences in the genomics and biology of these parasites is important. The availability of significant but partial genome data of the RMPs enabled the construction of a virtual composite RMP genome and its comparison with the *P. falciparum* genome, generating a so-called synteny map. Analysis of this map provided the desired comparative insights. A high level of conservation exists between roughly 85% of the genes at the level of content and order, but 168 *P. falciparum*-specific genes that disrupted the conserved genome segments were identified. The majority of these genes were predicted to play a role in host–parasite interactions. This study indicates that determination of the synteny breakpoints may help to rapidly identify the species-specific gene content of future *Plasmodium* genomes, providing the malaria research community with a powerful investigative tool. The findings may also be of interest to those studying chromosomal evolution.

## Introduction

Comparative genomics enables inferences to be drawn concerning the coding potential of related genomes and the evolutionary forces that have influenced genome organization [1]. The resolving power of whole-genome comparisons to a large extent depends upon the proximity of the phylogenetic relationship between the species. Comparative eukaryotic genome studies of several species from a wide range of lineages and different times of divergence have revealed that the level of both the conservation of organization and the recombination rates are relatively variable. Human and mouse, which diverged ~75 million years (My) ago, have a predicted gene content that is 80% orthologous [2] arranged in 281 synteny blocks (SBs) larger than 1 Mb [3]. Three-way alignment of the human genome with that of mouse and rat confirmed the conservation of ~280 SBs between human and each of the rodent genomes, while the more closely related rat and mouse genomes are ~90% orthologous with a reduced number of 105 shared SBs of larger average size [4]. Subsequent publication of the chicken genome, which diverged from the mammalian genomes ~310 My ago, provided the first nonmammalian amniote genome sequence and allowed a four-way whole-genome comparison [5] revealing 586 smaller, conserved SBs. Here, roughly 50% of the human genes have a chicken ortholog reducing to 35% that have orthologs in both chicken and pufferfish (estimated time of divergence ~450 My). These data show that, in terms of the extent of organization and gene homology, the level of genomic conservation can generally be considered to be relatively proportional to the time of divergence, within these species. However, a more recent comparison of genome sequences from eight mammals demonstrated that the rates of chromosomal rearrangements can vary both between species and in time (about 0.2–2 breaks/My) [6].

In contrast with the relatively slow evolution of mammalian and chicken chromosome structure, gene order and linkage in Diptera species has altered at a much higher rate. Although 50% of the genes are orthologs, little conservation of synteny could be observed in comparisons of the genomes of the fruit fly with two different malaria mosquitoes, which diverged ~250 My ago [7,8]. Even in the more closely related Diptera [8,9], extensive reshuffling and inversion have altered the gene order and organization, although genes were found to be located on the same chromosome arms. Similarly, the genomes of the nematodes *Caenorhabditis elegans* and *C. briggsae,* which diverged ~100 My ago, share 60% gene orthology but are arranged as 4,837 microsyntenic clusters [10].

The continuing efforts to sequence a variety of unicellular parasites has resulted in the publication of a comparison of the genome sequences of three human protozoan pathogens, *Trypanosoma brucei, T. cruzi,* and *Leishmania major* [11], and two apicomplexan parasites infecting cattle, *Theileria annulata* and *T. parva* [12]. The two *Theileria* species are very closely related, with 81% *(T. annulata)* and 86% *(T. parva)* orthologous genes and no interchromosomal rearrangements [12], comparable to the well-conserved genomes of four yeast species that diverged only 5–20 My ago and show relatively few (1–5) translocations [13]. The trypanosomatid species *T. brucei* and *L. major* share 68% and 75% gene orthology, respectively, organized in 110 SBs, despite having diverged as long as 200–500 My ago (chromosomal recombination rate of ~0.2–0.5 breaks/My) [11]. In conclusion, these comparative genome studies indicate that effective recombination rates and levels of gene orthology can vary greatly between species but are relatively low in protozoa.

In both pathogenic bacteria and certain unicellular eukaryotes (e.g., the trypanosomatids listed above), including members of the genus *Plasmodium* that are the etiological agents of malaria, the organization and gene content of the subtelomeric regions of chromosomes are highly variable and typically contain large gene families encoding proteins that may be involved in host-pathogen interactions and antigenic variation [14]. The subtelomeric regions of *P. falciparum,* for example, harbor a repertoire of unique gene families, including 59 *var* [15–17], 149 *rif,* and 28 *stevor* [18,19]. The *var* family encodes the erythrocyte membrane protein 1 (PfEMP1), which is a variant antigen expressed at the erythrocyte surface. PfEMP1 is involved in the binding of parasite-infected erythrocytes to receptors of host endothelial cells, erythrocytes, lymphocytes, and blood platelets [14], is subject to antigenic variation, and is thought to play a role in virulence. Other *Plasmodium* species lack the *P. falciparum*-specific *var, rif,* and *stevor* families, but the subtelomeric regions of their chromosomes also harbor (species-specific) gene families. For example, the human parasite *P. vivax; P. knowlesi,* which infects primates; and three rodent malaria parasites (RMPs; *P. berghei, P. chabaudi,* and *P. yoelii*) share the *pir* superfamily [20,21]. Proteins encoded by the *pir* superfamily are also found on the surface of infected erythrocytes and may be implicated in antigenic variation [21]. It is generally believed that the subtelomeric location of gene families confers an enhanced capacity for gene diversification and amplification through mechanisms of ectopic recombination that may be between different chromosomes [22]. Such recombination may be facilitated through the clustering of telomeres at the nuclear periphery [23].

Genome sequence data for *Plasmodium* species are extensive and include a complete genome sequence for the major human pathogen *P. falciparum* [24] and 5× coverage of the genome of a RMP, *P. yoelii* [25]. The *P. yoelii* contigs, when aligned with the 14 *P. falciparum* chromosomes, demonstrated extensive similarity over the relatively short length of these contigs. However, similarity was evident only in the core regions of the chromosomes mainly containing conserved genes (4,500) that are present in all characterized *Plasmodium* species [20] and which are bounded by the variable subtelomeric regions that contain the different gene families. In addition to the genome sequence of *P. yoelii,* partial genome sequence and analysis have been published for two other RMPs, *P. berghei* and *P. chabaudi,* whose core genome sequence and organization are so similar [26–28] that it has proved possible to merge the sequenced DNA contigs of the three RMPs to form composite RMP (cRMP) contigs that cover 90% of the core RMP genomes [20,25]. In this study, the cRMP contigs and 138 sequence tagged site (STS) markers (Table S1) have been used to produce a whole-genome synteny map for the three RMPs that, when compared with the *P. falciparum* genome, identified 36 SBs describing the core genome. This synteny map shows that species-specific genes—including rapidly evolving *P. falciparum* gene families—are found not only in the subtelomeric regions but also at synteny breakpoints (SBPs) and as intrasyntenic indels. Our data suggest that chromosomal rearrangements in the core regions might be involved in the generation and subsequent dispersal of one such *P. falciparum*-specific gene family. These results show that not only recombination in the more frequently recombining subtelomeric regions but also chromosome-internal rearrangements may influence diversity and complexity of the *Plasmodium* genome, increasing the ability of the parasite to successfully interact with its vertebrate host.

## Results

### A Whole-Genome Synteny Map of Four *Plasmodium* Species

**Figure 1:**
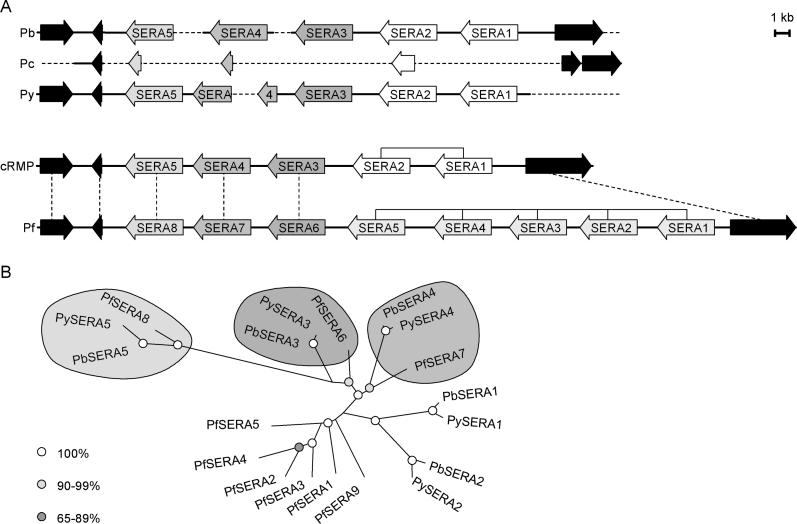
Deduced Organization of the cRMP *sera* Locus (A) The combination of three *P. berghei,* six *P. chabaudi,* and two *P. yoelii* contigs (thick black lines) in a region of Pfchr2 containing eight *sera* copies demonstrates the strength of the “composite genome approach.” Syntenic genes (black, linked by dashed vertical lines; left, PFB0315w and PFB0320c; right, PFB0365w) flank the *sera* clusters and reveal the presence of five *sera* genes in the RMPs. (B) Phylogenetic analysis revealed a close relation between *pfsera8, pfsera7,* and *pfsera6* and their syntenic orthologs in the RMPs (shaded gray, linked by dashed vertical lines in [A]). Other *sera* copies (*pfsera1–5, pbsera1–2,* and *pysera1–2*) clustered in species-specific groups (linked by solid horizontal lines in [A]). Circles represent branch points with bootstrap values of 100% (white), 90%–99% (light gray), and 65%–89% (dark gray).

A total of 7,392 contigs of the three RMPs, aligned with the *P. falciparum* genome, were used to generate 910 cRMP contigs (see Materials and Methods, Figure 1, and Tables 1 and S2). The tiling paths of all cRMP contigs are shown for both the individual *P. falciparum* and RMP chromosomes (Tables S3–S30). The cRMP contigs that were syntenic with the *P. falciparum* genome totaled 17.2 Mb (75%) of the 22.9 Mb *P. falciparum* genome, equivalent to 90% of the predicted total region of synteny. After linkage of the aligned cRMP contigs 229 gaps remained. No synteny could be observed in the subtelomeric regions of chromosomes between RMPs and *P. falciparum* [25], largely due to divergence of subtelomeric repeat sequences and gene families, but also to the poor assembly of these regions in the RMP genome projects [20].

**Table 1:**
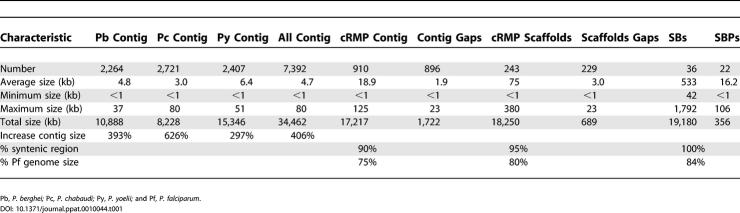
Summary of the Characteristics of the cRMP Contigs, Scaffolds, SBs, and SBPs

**Figure 2:**
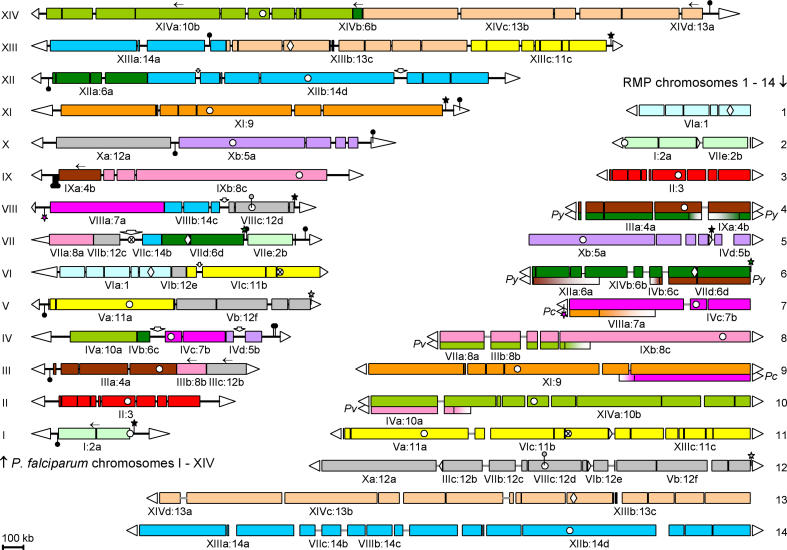
A Whole-Genome Synteny Map of *P. falciparum* and Three RMPs Synteny map of the core regions of all chromosomes of *P. falciparum* (left) and the RMPs (right), showing the 36 SBs, 22 SBPs, 14 CAT regions, *P. falciparum*-specific indels, and translocations in the RMP chromosomes. The 36 SBs, colored according to their chromosomal location in the cRMP genome, are named with a Roman and an Arabic number referring to the corresponding chromosome location in *P. falciparum* and the cRMP genome, respectively. Letters give the order in which the SBs are connected. Small arrows indicate the inverted orientation of a SB in *P. falciparum* relative to the cRMP genome. Indels containing *P. falciparum*-specific intrasyntenic genes are indicated through interruption of the colored SBs. *P. falciparum* telomeres are shown as white arrow heads (▹). SBs forming the cRMP chromosomes are linked by gray lines. In the cRMP genomes, the 23 coinciding subtelomeric linked ends are shown as white arrowheads (▹) and the five *P. falciparum* subtelomeric ends that are chromosomal internal in the cRMP chromosomes are indicated by small white arrowheads (▹). The 11 syntenic *P. falciparum* CAT regions [29] are shown as white circles (○), two inconsistent CAT regions as white circles with a cross (⊗), and three newly recognized CAT regions as white diamonds (⋄). Chromosome-internal *var* clusters are shown as white arrows (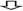); stars and circles on sticks indicate *rrna* gene units (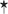) and *tstk* genes (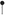); black stars and circles represent nonsyntenic genes; while syntenic genes (three *rrna* gene units and one *tstk* gene) are colored according to their chromosomal location in the RMPs. Bars under the cRMP chromosomes represent the differences in the organization of the SBs of *P. yoelii, P. chabaudi,* and *P. vinckei* as a result of translocations. Colors indicate the cRMP chromosome with which recombination has taken place, while color gradients represent the ill-defined regions of the translocation breakpoints.

When the alignment of the cRMP contigs with the *P. falciparum* genome was examined, 19 were identified with MUMmer hits to two different *P. falciparum* chromosomes, indicating that these contigs covered a SBP between the cRMP and the *P. falciparum* genomes. In addition, three SBPs were determined by chromosome mapping of STS markers and confirmed by PCR analysis, linking the cRMP contigs on either side of the SBP (unpublished data). In total, we found 22 SBPs in the core regions of the *P. falciparum* genome when compared to the core cRMP genome. Since the cRMP and *P. falciparum* genomes comprise 14 chromosomes, these 22 SBPs define a total of 36 SBs. Chromosome mapping of 138 *P. berghei* and *P. yoelii* STS markers (see Table S1) confirmed the 22 SBPs and the chromosomal location of the 36 SBs in the RMPs. The majority (23 of 28) of *P. falciparum* subtelomeric regions coincided with putative locations of cRMP subtelomeric regions, while the remaining five *P. falciparum* subtelomeric linked SBs were linked to SBPs in the cRMP genome. Conversely, five SBs that are linked to SBPs in *P. falciparum* were linked to subtelomeric regions in the cRMP genome. Figure 2 shows the reciprocal synteny maps of the *P. falciparum* and cRMP genomes.

Centrally located AT-rich (CAT) regions of 2–3 kb (average >97% AT) found on all *P. falciparum* chromosomes (with the exception of *P. falciparum* Chromosome 13 [Pfchr13]) have been predicted to be centromeres [29], and functional proof for their centromere function is accruing (S. Iwanaga, CJJ, and APW, unpublished data). While no CAT regions had been sequenced in the RMP genomes, genes immediately up- and downstream of 11 of the *P. falciparum* CAT regions were syntenic and located at 11 different cRMP chromosomes (Figure 2, Table S31). The predicted centromere of Pfchr7 is located in a SBP and therefore cannot be syntenic, and RMP sequences aligning with the predicted centromere of Pfchr6 did not show an elevated AT content in the cRMP chromosome. Assuming complete synteny of the CAT regions, we suggested new positions for the CAT regions of Pfchr6, 7, and 13 in the regions syntenic with cRMP Chromosome 1 (cRMPchr1), 6, and 13, respectively. Unpublished releases of the latest *P. falciparum* sequences confirmed these predictions (M. Berriman, personal communication). These results indicate that each of the 14 cRMP chromosomes contained one of the syntenic regions surrounding the *P. falciparum* CAT regions. Cloning and sequencing of two 1.5-kb regions of cRMPchr5 and 13 that aligned with the CAT regions of Pfchr10 and Pfchr13, respectively, revealed these were also extremely AT-rich (>97%) and consistent with the size and gene paucity of the *P. falciparum* CAT regions.

Comparison of the organization and location of common orthologous gene families of RMPs and *P. falciparum* allowed species-specific features of these families to be defined. For example, *P. falciparum* possesses a cluster of eight genes encoding putative serine proteases known as *sera* [30,31]. The *P. berghei* and *P. yoelii* databases both contain five *sera,* whose organization in the individual RMP genomes was unresolved, yet could be reconstructed using the cRMP contigs, demonstrating one utility of the cRMP contig construction (see Figure 1A). Combining the synteny analysis with standard phylogenetic analysis (see Figure 1B) indicated that all RMP *sera* cluster at a single locus on cRMPchr3, which aligns with the *P. falciparum sera* cluster on Pfchr2. Within these clusters, direct orthologs for three *sera* (RMP *sera3*–*5* and *P. falciparum sera6*–*8*) were immediately adjacent and thus syntenic. The remaining RMP *sera1*–*2* and the *pfsera1*–*5* are also immediately adjacent to one another and each positioned similarly within the *sera* cluster in both genomes but form different phylogenetic clades and can be considered species-specific.

### Inferring the Pathway of Synteny Rearrangements Between the cRMP and *P. falciparum* Genomes

The organization of the three RMP genomes is highly conserved, and only one or two chromosomal rearrangements were noted when the genomes of the individual RMP species were compared with the cRMP genome (Figure 2). The organization of the *P. berghei* genome is identical to that of the cRMP genome, suggesting it is also most similar to the genome structure of the most recent common ancestor of the RMPs.

**Figure 3:**
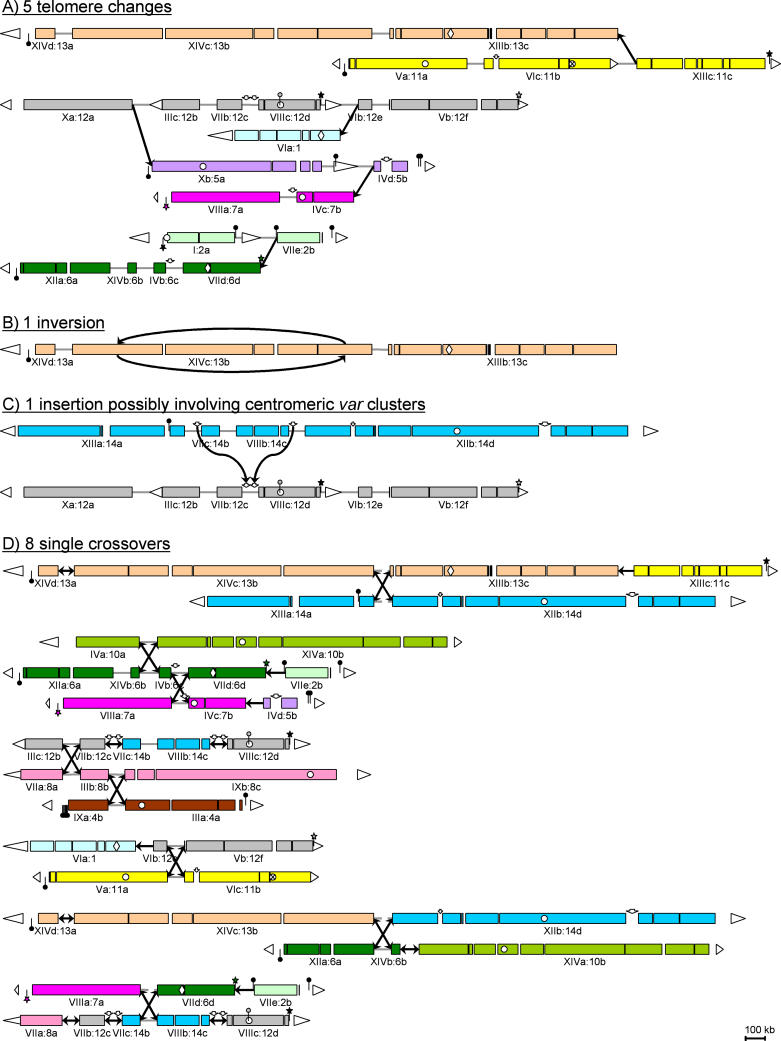
Schematic Representation of the 15 Recombination Events Schematic representation of the 15 recombination events that would permit the 36 SBs to be rearranged to generate the *P. falciparum* genome from the cRMP genome. See Figure 2 for the numbering of the SBs and the symbols used in this figure. Gray lines between SBs represent links as present in the cRMP genome; gray dashed lines indicate intermediate links, and black arrows show links corresponding to the *P. falciparum* genome. Five subtelomeric regions of the cRMP genome must become chromosome-internal in the *P. falciparum* genome (A), thereby generating five subtelomeric regions in *P. falciparum* that are linked to SBPs in the cRMP genome. SB “XIVc:13b” is inverted (B), and SBs “VIIc:14b” and “VIIIb:14c” are inserted between SBs “VIIb:12c” and “VIIIc:12d,” a process likely to involve chromosome-internal clusters of *var* and *rif* genes possibly mediated by *vicar* genes (C). Eight single crossover events generate the remaining links between the remaining SBs (D).

The *P. falciparum* genome organization could be generated from the cRMP genome in a minimum of 15 recombination events when the following assumptions were made: (i) that the resulting genome always consists of 14 chromosomes; (ii) that all chromosomes always contain only one of the SBs containing a CAT region; and (iii) that a recombination event generating a subtelomeric from a chromosome-internal region (or vice versa, collectively termed telomere conversions) has happened only once. These 15 recombination events included eight single crossover events, five telomere conversions, one inversion of an entire SB, and one insertion involving an intersyntenic *var* cluster (Figure 3). This most parsimonious pattern of gross chromosomal rearrangements was supported by analysis using the GRIMM (genome rearrangements in man and mouse) algorithm [3] that identified one inversion and 15 translocations, counting the *var* cluster insertion as two single translocation events (unpublished data). The relatively low number of 15 rearrangements events suggests that gross chromosomal rearrangements resulting in the loss of or change in synteny is infrequent in *Plasmodium*. However, the same recombination events could be associated with the formation and dispersal of (members of) species-specific gene families (see below).

### *P. falciparum*-Specific Genes Are Found Both at SBPs and in Intrasyntenic Indels

**Figure 4:**
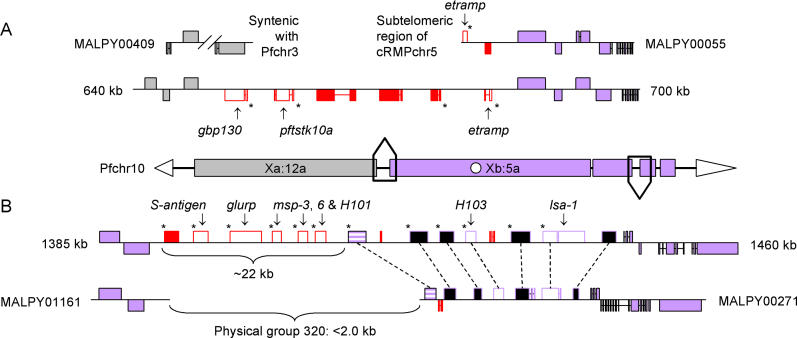
Inter- and Intrasyntenic Indels Contain Clusters of *P. falciparum*-Specific Genes (A) A detailed illustration of the SBP between the SBs “Xa:12a” and “Xb:5a” (Pfchr10) flanked by *P. yoelii* contigs MALPY00409 (gray) and MALPY00055 (purple). The last gene on MALPY00409 is located on Pfchr3 (“IIIc:12b”) and defines the SBP; MALPY00055 is the last syntenic contig flanking a subtelomeric region that contains a cRMP-specific gene encoding a hypothetical protein (red) and a nonsyntenic *etramp* (white with red outline). The *P. falciparum* intersyntenic region contains three annotated genes (white with red outline): *gbp130, pftstk10a,* and *etramp;* and three genes encoding hypothetical proteins (red). Interestingly, four of six genes encode putative secreted proteins with N-terminal transmembrane domains destined for the parasite surface or infected host cell membrane (asterisks; see Table S33). (B) A detailed illustration of a ~22-kb indel within SB “Xb:5a” that contains *P. falciparum*-specific genes directly upstream of a region containing genes that are highly diverged in the RMPs. Only four of 12 genes annotated on MALPY00271 have a clear ortholog (purple) and the last gene (PY01020), which encodes a hypothetical protein, shows low similarity at the N-terminal end with PF10_0348 (horizontal purple lines). Comparison of the *P. yoelii* and *P. falciparum* annotations revealed the presence in both species of six genes with the same orientation and comparable size, including four genes that encode hypothetical proteins (black with purple outline) and two annotated genes (white with purple outline): the putative *P. yoelii lsa1,* and the putative *msp* paralog *P. yoelii H103*. MALPY01161 and MALPY00271 are physically linked as determined with Grouper software and are therefore between 500 and 2,000 bp apart, leaving no space for the remaining genes in the ~22 kb *P. falciparum* indel that include *S-antigen, glurp, msp3, msp6,* and *H101* (all white with red outline) and one gene encoding a hypothetical protein (red). In the entire regions, 12 of 15 genes encode putative secreted proteins destined for the parasite surface or infected host cell membrane (asterisks; see Table S34).

The average size of species-specific DNA regions located between SBs (intersyntenic regions) is significantly smaller in the cRMP genome (~2.5 kb, range 0.4–15 kb) than in the *P. falciparum* genome (~16 kb, range 0.7–106 kb). Only four of the 19 intersyntenic regions in the cRMP genome for which sequence data are available contain a species-specific open reading frame, but only the nonsyntenic *c-rrna* gene unit on cRMPchr5 is known to be expressed (Tables 2 and S32). In contrast, eight of the 22 intersyntenic regions in *P. falciparum* contain clusters of one to 13 genes without RMP orthologs (Tables 2 and S33). These 42 intersyntenic genes include 14 *var* and six *rif* genes, as well as five other genes, which all encode proteins containing the *Plasmodium* export element/vacuolar transport signal motif (PEXEL/VTS) [32,33]—e.g., glycophorin-binding protein 130 precursor: GBP130 [34] and two receptor-associated protein kinases: PfTSTK7a, and PfTSTK10a (see also below). The PEXEL/VTS motif is one element that is associated with transport of the proteins to the surface of the infected erythrocyte. A further 12 genes encode proteins with a transmembrane domain at the N-terminal end (e.g., MAL7P1.58 of the *pfmc-2tm* family, which encodes proteins localized to the Maurer's clefts [35]), seven of which also have a signal peptide (e.g., PF10_0164 of the *etramp* family [36] and five *var internal cluster associated repeat [vicar]* genes; see also below). Figure 4A provides a detailed example of the SBP on Pfchr10 and alignment of the flanking syntenic regions with *P. yoelii* contigs. In conclusion, it seems that the majority of the intersyntenic, *P. falciparum*-specific, SBP-associated genes encode predicted exported proteins destined for the membrane surface of the cell-free parasite or the infected erythrocyte.

**Table 2:**
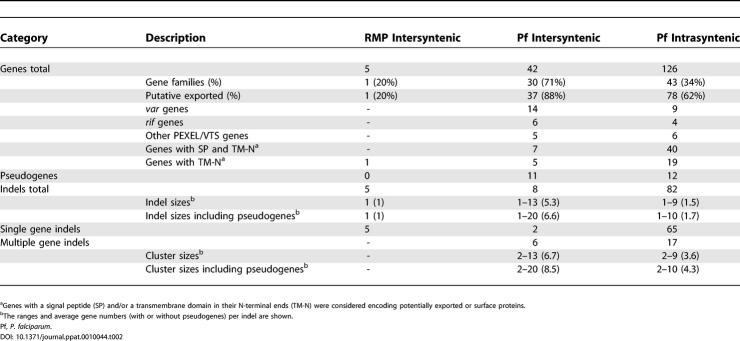
Summary of Inter- and Intrasyntenic Gene Content of *P. falciparum* and Comparison to Intersyntenic Gene Content of the RMPs

In addition to the species-specific genes located at SBPs, *P. falciparum*-specific genes were also found clustered in small intrasyntenic regions that interrupt the SBs (i.e. indels, Tables 2 and S34). These 82 indels, including four *var* clusters, range in size from one to nine genes but are generally less gene-rich than the intersyntenic regions (1.5 genes/indel compared to 5.3 genes/SBP). Whereas only two of eight SBPs contain a single *P. falciparum*-specific gene, 65 of 82 of the intrasyntenic indels contain only one gene. The 126 intrasyntenic, *P. falciparum*-specific genes include nine *var* and four *rif* genes as well as an additional six genes with the PEXEL/VTS motif [32,33] including *pftstk13* (MAL13P1.109, see also Discussion). Another 59 of these genes encode proteins with an N-terminal transmembrane domain, 40 of which also contain a signal peptide, giving a total of 78 genes encoding potential secreted or surface proteins. For example, a multigenic indel on Pfchr10 (Figure 4B) contains a cluster of six *P. falciparum*-specific genes that are all expressed in merozoites [37–39] and encode three known merozoite surface protein paralogs (MSP3, MSP6, and H101), glutamate-rich protein (GLURP), S-antigen, and a hypothetical protein containing a signal peptide sequence. The presence of a fourth *msp* paralog H103 in the neighboring syntenic region suggests that the gene content of this indel might have arisen in part through local gene duplication [40].

### Evolution of Gene Families Associated with Recombination Events at SBPs

**Figure 5:**
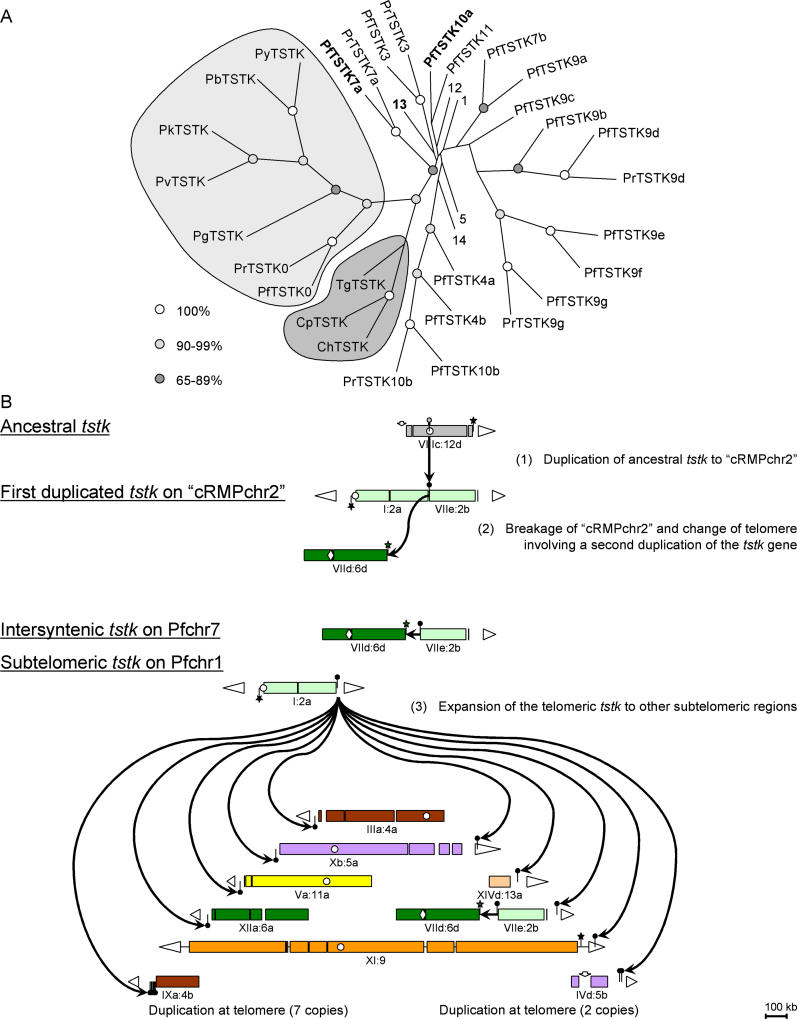
Origin and Putative Mechanism of Expansion of the *tstk* Family in *P. falciparum* (A) Analysis of *P. falciparum*-specific genes at the SBPs revealed a gene family encoding receptor-associated protein kinases (TSTK). Maximum likelihood distances were calculated for the C-terminal 400 amino acids of all TSTKs, including those found for other *Plasmodium* species, *Toxoplasma gondii, Cryptosporidium parvum,* and *C. hominis*. The tree was rooted using the clade with the three non-*Plasmodium* sequences as the outgroup (shaded dark gray). The syntenic progenitor genes clearly form one clade (shaded light gray), while the clustering of the other 20 mainly subtelomeric *pftstk* is more ambiguous (the three non-subtelomeric copies are shown in bold and include *pftstk7a,* which appears most closely related to the clade of progenitor genes). Circles represent branch points with bootstrap values of 100% (white), 90%–99% (light gray) and 65%–89% (dark gray). (B) See Figure 2 for the numbering of the SBs and the symbols used in this figure. Based on the 15 recombination events described in Figure 3 and the phylogenetic analysis of the *tstk* family, we suggest the origin and putative evolution of the *pftstk* family as shown here. Phylogenetic analysis suggests that the intersyntenic *pftstk7a* is most closely related to the progenitor founder gene, *pftstk0*. Interestingly, this gene is the first nonsyntenic gene upstream of SB “VIIe:2b.” This SB is linked in the cRMP genome to SB “I:2a” that in *P. falciparum* is also flanked by a member of the *tstk* family, the subtelomeric *pftstk1*. Based on these observations we suggest that the founder gene *pftstk0* was duplicated after the split of *P. falciparum* from the other *Plasmodium* species but before SBs “VIIe:2b” and “I:2a” were separated (1). This gene was then directly involved in the breakage of this link, creating Pfchr1 (“I:2a”) and destroying the telomere of “VIId:6d” by addition of “VIIe:2b” (2). During this recombination process, the gene was duplicated and is now present not only as two chromosome-internal copies on “VIIIc:12d” *(pftstk0)* and between “VIId:6d” and “VIIe:2b” *(pftstk7a)* but also as a first telomeric copy on the newly formed telomere of Pfchr1 *(pftstk1)*. From here the gene could expand to the other subtelomeric regions (3). Local gene duplications resulted in the generation of seven copies on Pfchr9 and two copies on Pfchr4. After a copy of *pftstk* ended up at the left-hand cRMP subtelomeric end of SB “Xb:5a,” the telomere conversion linked SB “Xa:12a” to SB “Xb:5a,” which turned this telomeric copy into an intersyntenic gene *(pftstk10a)*. The last non-subtelomeric copy, *pftstk13,* most likely resulted from a different process of mobility of *P. falciparum*-specific elements creating the intrasyntenic genes.

In order to analyze whether recombination events in the core regions that resulted in the loss of synteny are associated with the dispersal and formation of species-specific gene families, all intersyntenic genes of *P. falciparum* and the RMPs were analyzed for the presence and location of orthologous genes in their respective genomes. In addition to members of the *var, rif,* and *rrna* families, one intrasyntenic *(pftstk13)* and two intersyntenic *(pftstk7a* and *pftstk10a) P. falciparum* genes were identified that belong to a gene family encoding 21 transforming growth factor β receptor-like serine/threonine protein kinases (PfTSTK) [41–43]. In addition to these three genes, 17 members are located in the subtelomeric regions of 10 different chromosomes (Table S35), and one member is located adjacent to the Pfchr8 CAT region (M. Berriman, personal communication). In the RMP genome there is a single member of this family on cRMPchr12 syntenic to the copy near the Pfchr8 CAT region. Phylogenetic analysis groups these syntenic kinases in the same clade as the unique members of all other characterized *Plasmodium* species, with exception of the proteins encoded by the multiple *tstk* genes found in *Plasmodium reichenowi,* a very close relative of *P. falciparum* infecting chimpanzees [44]. These findings suggest that the syntenic *pftstk* on Pfchr8 could be the progenitor gene of this *P. falciparum*-specific gene family (Figure 5A).

Two different recombination pathways that would generate the *pftstk* family are consistent with the data. (i) A copy of the syntenic, orthologous progenitor *pftstk* on Pfchr8 relocated to a subtelomeric region, where it underwent extensive gene duplication and redistribution. The centrally located *pftstk* genes could then have originated from telomere changes. (ii) Combining the information on the location and phylogeny of the *pftstk* family with the predicted 15 synteny rearrangements suggests that both chromosome-internal rearrangements resulting in the loss of synteny and subtelomeric recombination are associated with the evolution and distribution of this family (Figure 5B). *P. falciparum*-specific duplication/translocation of the ancestral *tstk* to an ancestral “cRMPchr2” followed by chromosome breakage and recombination may have led to the translocation of a *tstk* copy to a subtelomeric position (Pfchr1). Additional subtelomeric copies may be translocated to the nine additional subtelomeric locations by ectopic recombination events between different chromosomes similar to the events suggested to play a crucial role in the generation of *var* gene diversity [23]. The intersyntenic copy on Pfchr10 might be the result of a subsequent recombination event leading to the internalization of this gene. The intrasyntenic *pftstk13* may have originated independently of the mechanism that generated this gene family in a similar (if obscure) mechanism to other intrasyntenic genes with apparent subtelomeric origin, including the *var* and *rif* genes. All the predicted duplication and translocation events required to distribute the *pftstk* family could be linked to the proposed rearrangement pathway that converts the RMP genome organization to that of *P. falciparum*. Since there are alternative pathways for the order of the suggested SBP recombination events (also indicated by the GRIMM algorithm analysis; Table S36), further elucidation of the pathway of recombination from the genome organization of the most recent common ancestor of *Plasmodium* awaits the availability of the genome of a third species [45].

### Identification of a New Putative Gene Family Associated with Chromosome-Internal *var* Clusters

Since repetitive sequences might be associated with recombination events between SBs, the intergenic regions flanking SBPs were examined using the MEME algorithm. This analysis resulted in the identification of a highly conserved *P. falciparum*-specific gene family consisting of seven putative genes and eight pseudogenes termed *var internal cluster associated repeat (vicar)* genes. These genes were found to be associated with five of seven chromosome-internal *var* clusters. Of these seven genes, five have a signal peptide and five genes have one or two transmembrane domains; only one of these genes is identified in the current annotation (MAL7P1.39) and is supported by transcriptome data [38]. The sequences correspond to the previously described GC-rich elements that were suggested to serve as regulatory elements for *var*-related genetic processes [29]. No other repetitive sequences were identified that, in the light of current knowledge, could be associated with chromosomal recombination events.

## Discussion

The generation of composite contigs from three closely related *Plasmodium* species infecting rodents greatly facilitated the construction of a synteny map between the RMPs and *P. falciparum* and significantly reduced the need for experimental data from PCR and STS mapping studies. Current contig assembly algorithms rely upon a minimum of 95% sequence identity between sequence reads [46], a criterion not met by the RMP sequences. The high degree of synteny and similarity of gene content of the core *Plasmodium* genome enabled the compilation of cRMP contigs using sequences of the three RMPs with a lower sequence identity by aligning them to the assembled *P. falciparum* sequence. With only 229 gaps remaining and the location of 138 STS markers identified, the synteny map is a comprehensive tool for identifying the location of most genes. Individually, cRMP contigs are not sufficient to build an entire composite genome, since coverage and linkage of the cRMP scaffolds are incomplete. An unknown proportion of small rearrangements such as single gene insertions, inversions, or deletions will have been missed. Thus the need for continued sequencing to completion of at least one RMP genome remains. Approximately 4,500 (85%) of the 5,300 predicted *P. falciparum* genes have an ortholog in at least one of the RMPs, and these likely represent the core set of *Plasmodium* genes [20]. A similar level of orthology is seen in the genome organization, since the 36 SBs cover 84% of both genomes.

The synteny maps of *P. falciparum* and cRMP demonstrated that only a minimum of 15 recombination events are needed to generate the *P. falciparum* genome from the 36 SBs of the RMPs, compared with 245 events needed to convert the human genome organization to that of the mouse [3]. This relatively low number of *Plasmodium* genome rearrangements suggests either that divergence of *P. falciparum* and the RMPs might be relatively recent or that chromosomal rearrangements in *Plasmodium* are infrequent, either as a result of unknown (intrinsic) features of the DNA or due to some higher order organization of the genome [26]. Because the evolutionary relationships and the time of divergence between *P. falciparum* and other *Plasmodium* species is unclear [44,47–52], it is not yet possible to draw conclusions on the rate of chromosomal rearrangements in *Plasmodium*. A rough estimate consistent with published data would be that *P. falciparum* diverged and developed separately between 50 and 200 My ago. Thus the effective chromosomal recombination rate would be between 0.08 and 0.3 breaks/My. In comparison, the recombination rate in yeast species appears to be ~0.2 breaks/My [13]. Both are at the lower end of the range of rates observed for different mammalian species [6]. The genomes of different trypanosomatid species were also suggested to have a low recombination rate [11].

In many species, centromeres have been associated with chromosomal rearrangements and have proven to be positionally dynamic, with transposable elements often found to function in centromere relocation [1]. *Plasmodium* centromeres have not been functionally characterized but based on previous predictions, preliminary functional evidence (S. Iwanaga, CJJ, and APW), and the distribution of the CAT regions as demonstrated by the *Plasmodium* synteny map, it is tempting to suggest that the predicted centromeres of *Plasmodium* are positionally static. One of the assumptions upon which the initial intuitive derivation of the minimum 15 recombination events was based was that each chromosome at any time always contains one CAT region and one only, in keeping with their still-hypothetical function as centromeres. The GRIMM analysis did not include such an assumption, yet it predicted the same number of rearrangements, while maintaining a single SB containing a CAT region in each newly formed chromosome, emphasizing their predicted lack of involvement in the recombination events identified in this study. Furthermore, these recombination events are also unlikely to involve transposable elements, since these were not found in a cross-species comparison of the sequences in the vicinity of SBPs, consistent with previous studies [24].

In contrast to the low number of chromosomal rearrangements in the *Plasmodium* genomes, a relatively large proportion (15%) of the *P. falciparum* genes have no readily identifiable ortholog in any of the RMPs. These genes (including the well known *var, rif,* and *stevor* families) are mainly located in the subtelomeric regions, which appear to have a higher rate of gene evolution in many organisms, including *Plasmodium* [1,22]. However, this study shows that a significant proportion of *P. falciparum*-specific genes and members of gene families are not restricted to the subtelomeric region of the chromosomes but can be found as intrasyntenic indels and at SBPs. The majority (115 genes [68%]) of these 168 genes encode predicted or known surface or secreted proteins that are predominantly expressed in asexual blood stage parasites (both infected erythrocytes and merozoites) and thus are involved in parasite interactions with the human host and possibly associated with immune selection/evasion. Interestingly, several of the larger clusters of genes, such as the indel containing *msp3* and *msp6,* appear to be coordinately expressed and may even be transcribed in an operon-like manner [53], despite earlier analyses that did not find evidence for the existence of such clusters [37]. Perhaps surprisingly, indels containing RMP-specific genes were not readily found, and although this may be in part due to the incomplete RMP genome sequence data that are currently available, the depth of coverage of the cRMP genome suggests that RMP indels are not as frequent as in *P. falciparum*. However, indels are not absent from the RMP genomes, and evidence is accumulating for RMP indels that contain members of the *pir* superfamily normally found in the subtelomeric regions reminiscent of the organization of the *var* family in the *P. falciparum* genome (see Tables S3–S30) [20,21].

To test whether SBPs are significantly more associated with chromosome-internal *P. falciparum*-specific genes than what might be expected based on a random distribution of the SBPs, we used computer simulations to generate randomly distributed SBPs in the genome and compared these with the inter- and intrasyntenic gene content. Using a conservative and a more relaxed approach (see Materials and Methods), we showed that based on a random breakage model, between 1.9 and 3.0 of the 22 SBPs on average could be expected to be associated with *P. falciparum*-specific gene clusters. This is significantly different (*p* < 0.001) from the observed association of eight (36%) of the 22 SBPs with *P. falciparum*-specific genes. This result indicates a nonrandom distribution of *P. falciparum*-specific genes associating with a higher frequency to SBPs and, therefore, with chromosomal rearrangements that have led to loss of synteny. Interestingly, from comparisons of the human and mouse genomes, evidence has emerged for a similar nonrandom distribution of repeat sequences in the genome and their association with SBPs [54,55].

The presence of members of species-specific gene families at the SBPs suggests that recombination events resulting in loss of synteny helped shape species-specific gene content. SBPs and the intrasyntenic indels might therefore distinguish islands where variations in gene content occur (and then evolve) between the different *Plasmodium* species. The location and phylogeny of the *pftstk* family and the chromosomal rearrangements between SBs were consistent with different possible recombination pathways and mechanisms. Interestingly, the processes of gene duplication and translocation described for the *tstk* family could also be associated with the generation of two other gene families in *P. falciparum* encoding acyl-CoA binding proteins (ACP; four *P. falciparum* genes and one cRMP gene) and acyl-CoA synthetases (ACS; 11 *P. falciparum* genes and three cRMP genes). Both families have one syntenic copy in *P. falciparum* and the RMPs that are located in the *P. falciparum* genome next to an indel. The syntenic *acp* is located next to an indel on Pfchr8, and the syntenic *acs* next to an indel on Pfchr2 (PFB0685c). This latter gene appears to have undergone local gene duplication, followed by relocalization and expansion to seven subtelomeric copies in *P. falciparum* (unpublished data). In conclusion, our data show that both SBPs and intrasyntenic indels can be foci for species-specific genes with a predicted role in host-parasite interactions and indicate that not only rearrangements in the subtelomeric regions but also chromosomal rearrangements are involved in the generation of species-specific gene families. The majority are expressed in blood stages (complete list in Table S34), suggesting that the vertebrate host exerts a greater selective pressure than the mosquito vector, resulting in the acquisition of diversity.

It is already evident that a single recombinational mechanism underlying the origin of the inter- and intrasyntenic gene content or the generation of gene families in *P. falciparum* cannot be postulated. The 42 SBP-associated genes of *P. falciparum* can be classified into three groups: (i) two single genes that are associated with single crossover events; (ii) three clusters of genes (total 12 genes) that might have their origin in subtelomeric regions that became chromosome-internal after a telomere change (these include the SBPs containing *pftstk* genes); and (iii) three *var* clusters, two associated with the insertion of SBs “VIIc:14b” and “VIIb:14c” and one associated with a single crossover event (total 28 genes; see Table S33). Thus it is clear that different recombination mechanisms were involved in shaping the *P. falciparum* genome. Evidence from both the 15 SBP-associated recombination events and previous *var* gene classifications [56] cannot be reconciled with an origin of central *var* clusters associated with telomere recombination changes and subsequent internalization of subtelomeric *var* genes. Both SBP and intrasyntenic *var* clusters are associated with the *vicar* genes identified in this study and previously described as the GC-rich elements [29]. The position of *vicar* elements is consistent with an as yet unproven role in recombination.

The pairwise whole-genome comparison presented here, while indicating that 15 chromosomal rearrangements can create the *P. falciparum* genome organization from that of the RMP, does not resolve the organization of the most recent common ancestor, which requires more complete *Plasmodium* genomes. Genome-wide comparison of the location and distribution of SBPs between different *Plasmodium* species should provide a reliable dataset enabling construction of a definitive phylogeny of the genus and resolving issues of precise clade topology [45]. In addition, whole-genome comparisons and the identification of SBPs might prove to be an effective means of identifying species-specific genes and members of gene families that are involved in host-parasite interactions and immune evasion, including antigenic variation.

## Materials and Methods

### Creation of a cRMP genome.

7,215 contigs of three RMP genomes, *P. yoelii yoelii* (17XNL line) [25], *P. berghei* (ANKA strain), and *P. chabaudi chabaudi* (AS strain) [20] were previously aligned with the *P. falciparum* genome using MUMmer to identify annotation-independent protein similarities [57]. We manually aligned an additional 177 contigs using linkage data from the *P. yoelii* genome publication and by performing BLASTN analyses with ~500-bp sized sequences from the ends of the RMP contigs, thus closing gaps in the synteny map and “walking” toward the telomeric ends. Linking of these 7,392 contigs through identification of overlapping contigs resulted in the generation of 910 cRMP contigs (see Figure 1A for an example of the procedure to generate cRMP contigs). The high level of nucleotide identity between the genomes of the three RMPs (*P. yoelii* versus *P. berghei,* 91.3%; *P. yoelii* versus *P. chabaudi,* 88.1%; and *P. berghei* versus *P. chabaudi,* 87.1%) facilitated this process. The cRMP contigs that showed MUMmer hits to two different *P. falciparum* chromosomes revealed SBPs. Linkage between adjacent *P. y. yoelii* contigs had previously been established using Grouper [58], through the alignment of overlapping *P. yoelii* expressed sequence tags and by PCR amplification [25]. Combining these data with the 910 cRMP contigs resulted in the generation of 243 scaffolds of linked cRMP contigs. STS markers were used to determine chromosomal locations of the linked cRMP contigs. These markers included 79 previously described and 59 new markers strategically chosen based on the position of the SBPs (see Table S1). All markers were hybridized to chromosomes of *P. yoelii, P. berghei, P. chabaudi,* and *P. vinckei* that had been separated by pulsed field gel electrophoresis [27].

### Analysis of the synteny map of the cRMP and *P. falciparum* genomes.

Intergenic sequences flanking the SBs at all 22 *P. falciparum* SBPs as well as the five subtelomere linked ends that are chromosome-internal in the RMPs (92 kb in total) were analyzed for repetitive motifs using MEME [59]. The intergenic sequences of the 20 RMP SBPs for which sequence was available were also analyzed. Nonsyntenic genes were compared with the genome data of the different *Plasmodium* species by TBLASTN analysis, and the expression profiles and putative functions of these genes were investigated using data available from PlasmoDB [30,31,38,39]. The predicted protein sequences of the *tstk* family members were analyzed for functional domains by SMART [60].

GRIMM [3] was used to confirm the suggested minimum 15 recombination events. To test the significance of the association between SBPs and *P. falciparum*-specific gene content, we used computer simulations to reassign the 22 chromosome-internal SBPs to random positions in the core genome of *P. falciparum,* thus excluding the subtelomeric regions. We used two different approaches: The first approach utilized the sizes of the entire SBP regions, including the species-specific gene content, while the second approach utilized fixed SBP sizes (5 kb, slightly larger than the largest noncoding intergenic, intersyntenic regions). For both approaches, we counted the number of associations of the virtual SBPs of 1,000 random distributions with the locations of all inter- and intrasyntenic genes.

Phylogenetic analyses of members of the TSTK and SERA families were performed using manually corrected ClustalW alignments [61]. Protein parsimonies, pairwise distances and maximum likelihood distances were calculated using different regions of alignment with algorithms and matrices from the phylogeny inference package (PHYLIP) [62] and gave comparable results. For the final tree construction, 100 bootstrap trees were generated (each with 10× jumbling) of a manually corrected alignment of roughly 400 amino acids of the C-terminal ends of all TSTKs containing the serine/threonine protein kinase domain using SEQBOOT [63]. Maximum likelihood distances [64] were calculated using default parameter settings and 10× jumbling. The 100 bootstrap trees thus constructed were combined using CONSENSE [65]. The tree was rooted using the clade of non-*Plasmodium* TSTKs as the outgroup with RETREE, and the final tree was drawn using DRAWTREE, both also available from PHYLIP [62].

## Supporting Information

Table S1 STS Marker List (111 KB XLS)

Table S2 Details of the cRMPchrs (64 KB TXT)

Table S3 Pfchr1 (138 KB XLS)

Table S4 Pfchr2 (214 KB XLS)

Table S5 Pfchr3 (257 KB XLS)

Table S6 Pfchr4 (238 KB XLS)

Table S7 Pfchr5 (369 KB XLS)

Table S8 Pfchr6 (382 KB XLS)

Table S9 Pfchr7 (303 KB XLS)

Table S10 Pfchr8 (346 KB XLS)

Table S11 Pfchr9 (357 KB XLS)

Table S12 Pfchr10 (381 KB XLS)

Table S13 Pfchr11 (540 KB XLS)

Table S14 Pfchr12 (585 KB XLS)

Table S15 Pfchr13 (714 KB XLS)

Table S16 Pfchr14 (809 KB XLS)

Table S17 cRMPchr1 (215 KB DOC)

Table S18 cRMPchr2 (216 KB DOC)

Table S19 cRMPchr3 (235 KB DOC)

Table S20 cRMPchr4 (270 KB DOC)

Table S21 cRMPchr5 (296 KB DOC)

Table S22 cRMPchr6 (332 KB DOC)

Table S23 cRMPchr7 (285 KB DOC)

Table S24 cRMPchr8 (429 KB DOC)

Table S25 cRMPchr9 (598 KB DOC)

Table S26 cRMPchr10 (513 KB DOC)

Table S27 cRMPchr11 (592 KB DOC)

Table S28 cRMPchr12 (627 KB DOC)

Table S29 cRMPchr13 (842 KB DOC)

Table S30 cRMPchr14 (845 KB DOC)

Table S31 Centromere Predictions (68 KB DOC)

Table S32 RMP Intersyntenic Genes (38 KB DOC)

Table S33 Pf Intersyntenic Genes (175 KB DOC)

Table S34 Pf Intrasyntenic Genes (477 KB DOC)

Table S35 Pf Subtelomeric Genes (687 KB TXT)

Table S36 GRIMM Analysis (171 KB DOC)

### Accession Numbers

The GenBank (http://www.ncbi.nlm.nih.gov) accession numbers for the sequences of two putative *P. yoelii* centromeres (Chromosomes 5 and 13) are DQ054838 and DQ054839, respectively.

All datasets will become available through the official Web site of the *Plasmodium* genome project, PlasmoDB (http://plasmodb.org) [30,31]. The PlasmoDB accession numbers for the *P. falciparum* cluster of eight genes encoding putative serine proteases known as *sera* are PFB0325c–PFB0360c. The PlasmoDB accession numbers for other genes and gene products discussed in this paper are, for *P. falciparum*: *etramp* (PF10_0164), *gbp130* (PF10_0159), *glurp* (PF10_0344), *H101* (PF10_0347), *H103* (PF10_0352), hypothetical protein (PF10_0342), *lsa1* (PF10_0356), *msp3* (PF10_0345), *msp6* (PF10_0346), *pftstk1* (PFA0130c), *pftstk7a* (MAL7P1.144), *pftstk10a* (PF10_0160), *pftstk13* (MAL13P1.109), and *S-antigen* (PF10_0343); for *P. berghei*: *H103* (PB105993.00.0), *lsa1* (PB101910.00.0+PB105996.00.0), and five *sera* (PB000649.01.0, PB000352.01.0, PB000107.03.0, PB107093.00.0, PB000108.03.0); for *P. yoelii*: *etramp* (PY00205), *H103* (PY01016), *lsa1* (PY01014), and five *sera* (PY02063, PY02062+PY00294, PY00293, PY00292, PY00291).

*P. berghei* and *P. yoelii* gene models referred to in the text are available from GeneDB (http://www.genedb.org), and GeneIndices (http://www.tigr.org/tdb/tgi/protist.shtml).

## Abbreviations

CAT: centrally located AT-rich
cRMP: composite rodent malaria parasite
cRMPchr: composite rodent malaria parasite chromosome
My: million years
PEXEL/VTS: *Plasmodium* export element/vacuolar transport signal
Pfchr: *P. falciparum* chromosome
RMP: rodent malaria parasite
SB: synteny block
SBP: synteny breakpoint
STS: sequence tagged site
TSTK: transforming growth factor β receptor-like serine/threonine protein kinases
