# Supplementary material for: A Plasmodium Whole-Genome Synteny Map: Indels and Synteny Breakpoints as Foci for Species-Specific Genes
Source: PLoS Pathog. 2005 Dec 23;1(4):e44. doi: 10.1371/journal.ppat.0010044 (PMC1317653; doi:10.1371/journal.ppat.0010044)
Supplement: Supplementary file 1 [file ppat.0010044.st017.doc]

Table S17 - RMPchr1


# Table S17 - RMPchr1

|  |  |  |  |  |  |  |  |  |  |  |  |  |  |  |  |  |  |  |  |
| --- | --- | --- | --- | --- | --- | --- | --- | --- | --- | --- | --- | --- | --- | --- | --- | --- | --- | --- | --- |
| Table S17: Tiling path of RMP contigs combined to form cRMP chromosome 1. | | | | | | |  |  |  |  |  |  |  |  |  |  |  |  |  |
|  |  |  |  |  |  |  |  |  |  |  |  |  |  |  |  |  |  |  |  |
| Tiling paths of P. berghei (Pb), P. c. chabaudi (Pc) and P. y. yoelii (Py) contigs combined into composite rodent malaria parasite (cRMP) | | | | | | | | | |  |  |  |  |  |  |  |  |  |  |
| contigs aligned against parts of the 14 chromosomes of P. falciparum (Pf). The cRMP contigs are further linked by physical linkage | | | | | | | | | |  |  |  |  |  |  |  |  |  |  |
| and PCR (see Methods) to form cRMP scaffolds. Different synteny blocks with homology to different P. falciparum chromosomes | | | | | | | | | |  |  |  |  |  |  |  |  |  |  |
| together form the cRMP chromosomes. The tables also show gaps in the alignment (black lines), cRMP telomeres (white bars) and | | | | | | | | | |  |  |  |  |  |  |  |  |  |  |
| predicted centromeres (plum/black bars). Contigs, physical groups or PCR groups interrupted by inter- or intrasyntenic regions are | | | | | | | | | |  |  |  |  |  |  |  |  |  |  |
| printed in bold. A summary of the number of RMP contigs, cRMP contigs and cRMP scaffolds as well as their average, minimum, | | | | | | | | | |  |  |  |  |  |  |  |  |  |  |
| maximum and total sizes is provided. Column headings are as follows: | | | | |  |  |  |  |  |  |  |  |  |  |  |  |  |  |  |
|  |  |  |  |  |  |  |  |  |  |  |  |  |  |  |  |  |  |  |  |
| Pf chr.: P. falciparum chromosome to which the RMP contigs are aligned | | | | | |  |  |  |  |  |  |  |  |  |  |  |  |  |  |
| (#: PROmer analysis� from this study; JC# and TK#: Carlton et al.; man#: manual BLAST analysis; | | | | | | |  |  |  |  |  |  |  |  |  |  |  |  |  |
| i: inversed orientation of the synteny block; colours refer to those used in Fig. 2 of the paper); | | | | | | |  |  |  |  |  |  |  |  |  |  |  |  |  |
| Pb Pc Py contig: names of the aligned RMP contigs; | | |  |  |  |  |  |  |  |  |  |  |  |  |  |  |  |  |  |
| Size: size (bp) of the aligned RMP contigs; | |  |  |  |  |  |  |  |  |  |  |  |  |  |  |  |  |  |  |
| +/-: orientation of the aligned RMP contigs; | |  |  |  |  |  |  |  |  |  |  |  |  |  |  |  |  |  |  |
| Start contig & End contig: estimated start & end coordinates of alignment of the RMP contigs based on the coordinates | | | | | | | | |  |  |  |  |  |  |  |  |  |  |  |
| of PROmer hits corrected for the contig�s size and orientation and presence of inter- and intrasyntenic regions | | | | | | | |  |  |  |  |  |  |  |  |  |  |  |  |
| (the start position of the first RMP contig in the alignment was set at zero); | | | | | |  |  |  |  |  |  |  |  |  |  |  |  |  |  |
| cRMP contig: names of cRMP contigs | |  |  |  |  |  |  |  |  |  |  |  |  |  |  |  |  |  |  |
| (the first 2 digits refer to the cRMP chromosome number and the following 3 digits designate the order of the contigs); | | | | | | | | |  |  |  |  |  |  |  |  |  |  |  |
| cRMP contig size: size of cRMP contigs based on the corrected coordinates of alignment; | | | | | | |  |  |  |  |  |  |  |  |  |  |  |  |  |
| Gap size to next cRMP contig: size of gaps between neighbouring cRMP contigs; | | | | | | |  |  |  |  |  |  |  |  |  |  |  |  |  |
| Start cRMP contig & End cRMP contig: estimated start & end coordinates of alignment of the cRMP contigs; | | | | | | | |  |  |  |  |  |  |  |  |  |  |  |  |
| Phys. group (Py): physical linkage of neighbouring P. y. yoelii contigs; | | | | | |  |  |  |  |  |  |  |  |  |  |  |  |  |  |
| PCR group (Py): neighbouring P. y. yoelii contigs were linked previously or for this study by PCR; | | | | | | |  |  |  |  |  |  |  |  |  |  |  |  |  |
| PCR size (Py): size of the PCR product; | |  |  |  |  |  |  |  |  |  |  |  |  |  |  |  |  |  |  |
| cRMP scaffold: names of cRMP scaffolds | |  |  |  |  |  |  |  |  |  |  |  |  |  |  |  |  |  |  |
| (the first 2 digits refer to the cRMP chromosome number and the following 2 digits designate the order of the scaffolds); | | | | | | | | |  |  |  |  |  |  |  |  |  |  |  |
| cRMP scaffold size: size of cRMP scaffolds; | | |  |  |  |  |  |  |  |  |  |  |  |  |  |  |  |  |  |
| Gap size to next cRMP scaffold: size of gaps between neighbouring cRMP scaffolds; | | | | | | |  |  |  |  |  |  |  |  |  |  |  |  |  |
| Start cRMP scaffold & End cRMP scaffold: estimated start & end coordinates of alignment of the cRMP scaffolds; | | | | | | | | |  |  |  |  |  |  |  |  |  |  |  |
| Overlap: Linkage between RMP contigs established through manual BLAST analysis. | | | | | | |  |  |  |  |  |  |  |  |  |  |  |  |  |
|  |  |  |  |  |  |  |  |  |  |  |  |  |  |  |  |  |  |  |  |
| Pf chr. | Pb Pc Py contig | Size | +/- | Start contig | End contig | cRMP contig | cRMP contig size | Gap size to next cRMP contig | Start cRMP contig | End cRMP contig | Phys. group (Py) | PCR group (Py) | PCR size (Py) | cRMP scaffold | cRMP scaffold size | Gap size to next cRMP scaffold | Start cRMP scaffold | End cRMP scaffold | Overlap (manual) |
| Telomere | |  |  |  |  |  |  |  |  |  |  |  |  |  |  |  |  |  |  |
| man6 | PbContig4911 | 7363 | - | 0 | 7363 |  |  |  |  |  |  |  |  |  |  |  |  |  | \| |
| man6 | Pb.c000601476.Contig1 | 4793 | - | 2568 | 7361 |  |  |  |  |  |  |  |  |  |  |  |  |  | \| |
| man6 | PcContig4008 | 1271 | - | 2803 | 4074 |  |  |  |  |  |  |  |  |  |  |  |  |  | \| |
| man6 | MALPY02056 | 13598 | - | 3876 | 17474 |  |  |  |  |  |  |  |  |  |  |  |  |  | \| |
| man6 | PbContig120 | 8862 | - | 15249 | 24111 |  |  |  |  |  |  |  |  |  |  |  |  |  | \| |
| man6 | MALPY02042 | 6869 | - | 16060 | 22929 |  |  |  |  |  | 47B |  |  |  |  |  |  |  | \| |
| man6 | Pb.c001301723.Contig1 | 5716 | - | 17942 | 23658 |  |  |  |  |  |  |  |  |  |  |  |  |  | \| |
| man6 | PcContig6063 | 3471 | + | 20402 | 23873 |  |  |  |  |  |  |  |  |  |  |  |  |  | \| |
| man6 | MALPY01618 | 2997 | - | 23304 | 26301 |  |  |  |  |  | 47B |  |  |  |  |  |  |  | \| |
| man6 | PcContig6551 | 5142 | - | 23795 | 28937 |  |  |  |  |  |  |  |  |  |  |  |  |  | MALPY00623 |
| 6 | Pc.c000402774.Contig1 | 5061 | - | 27307 | 32260 |  |  |  |  |  |  |  |  |  |  |  |  |  |  |
| 6 | Pb.c001601985.Contig2 | 5618 | - | 29839 | 35620 |  |  |  |  |  |  |  |  |  |  |  |  |  |  |
| 6 | MALPY03325 | 1012 | - | 29846 | 30900 |  |  |  |  |  | 47B |  |  |  |  |  |  |  |  |
| 6 | MALPY00623 | 4584 | - | 30938 | 35525 |  |  |  |  |  | 47B |  |  |  |  |  |  |  |  |
| 6 | Pc.c000003075.Contig1 | 1040 | + | 33352 | 34395 | cRMPc01001 | 35620 | 1259 | 0 | 35620 |  |  |  |  |  |  |  |  |  |
| man6 | MALPY02396 | 3613 | + | 36879 | 40492 |  |  |  |  |  | 47B |  |  |  |  |  |  |  | MALPY00507/PbContig261 |
| 6 | Pc.c000703437.Contig1 | 2576 | + | 38816 | 41389 |  |  |  |  |  |  |  |  |  |  |  |  |  |  |
| 6 | PbContig261 | 21156 | + | 38886 | 60054 |  |  |  |  |  |  |  |  |  |  |  |  |  |  |
| 6 | MALPY00507 | 12307 | + | 39729 | 51764 |  |  |  |  |  | 47B |  |  |  |  |  |  |  |  |
| 6 | Pc.c000503072.Contig1 | 1438 | + | 41791 | 43253 |  |  |  |  |  |  |  |  |  |  |  |  |  |  |
| 6 | PcContig448 | 10168 | + | 43164 | 53428 |  |  |  |  |  |  |  |  |  |  |  |  |  |  |
| 6 | MALPY02527 | 2705 | - | 52094 | 54746 |  |  |  |  |  | 47B |  |  |  |  |  |  |  |  |
| 6 | MALPY02983 | 609 | + | 53529 | 54138 |  |  |  |  |  |  | 353 | 250 |  |  |  |  |  |  |
| 6 | PcContig703 | 2378 | + | 53638 | 56001 |  |  |  |  |  |  |  |  |  |  |  |  |  |  |
| 6 | MALPY03473 | 1390 | - | 54009 | 55527 |  |  |  |  |  |  |  |  |  |  |  |  |  |  |
| 6 | MALPY00058 | 20510 | - | 54067 | 75281 |  |  |  |  |  | 47B | 353 |  |  |  |  |  |  |  |
| 6 | Pc.c000102659.Contig1 | 2767 | - | 56280 | 58972 |  |  |  |  |  |  |  |  |  |  |  |  |  |  |
| 6 | Pc.c000603030.Contig1 | 2556 | + | 63334 | 65887 |  |  |  |  |  |  |  |  |  |  |  |  |  |  |
| 6 | Pc.c000703885.Contig1 | 745 | + | 66401 | 67146 |  |  |  |  |  |  |  |  |  |  |  |  |  |  |
| 6 | Pb.c000301175.Contig2 | 5117 | + | 69480 | 74537 |  |  |  |  |  |  |  |  |  |  |  |  |  |  |
| 6 | Pc.c000602892.Contig1 | 5507 | - | 70700 | 76244 |  |  |  |  |  |  |  |  |  |  |  |  |  |  |
| 6 | MALPY00711 | 9644 | + | 74893 | 84279 |  |  |  |  |  | 1148 |  |  |  |  |  |  |  |  |
| 6 | PbContig285 | 9638 | - | 75151 | 84535 |  |  |  |  |  |  |  |  |  |  |  |  |  |  |
| 6 | Pc.c000002697.Contig1 | 4258 | - | 75787 | 79784 |  |  |  |  |  |  |  |  |  |  |  |  |  |  |
| 6 | Pc.c001404881.Contig1 | 1774 | - | 79714 | 81491 | cRMPc01002 | 47656 | 92 | 36879 | 84535 |  |  |  |  |  |  |  |  |  |
| 6 | MALPY00015 | 9521 | + | 84627 | 94284 |  |  |  |  |  | 1148 | 307 | 2200 |  |  |  |  |  |  |
| 6 | Pb.c001904307.Contig1 | 7544 | - | 85332 | 93100 |  |  |  |  |  |  |  |  |  |  |  |  |  |  |
| 6 | Pc.c001304142.Contig1 | 1429 | - | 85436 | 86868 |  |  |  |  |  |  |  |  |  |  |  |  |  |  |
| 6 | Pc.c001203245.Contig1 | 5563 | + | 87795 | 93703 | cRMPc01003 | 9657 | 757 | 84627 | 94284 |  |  |  |  |  |  |  |  |  |
| 6 | Pb.c002006855.Contig1 | 1907 | - | 95041 | 96948 | cRMPc01004 | 1907 | 53 | 95041 | 96948 |  |  |  |  |  |  |  |  |  |
| 6 | PcContig150 | 5031 | - | 97001 | 102012 |  |  |  |  |  |  |  |  |  |  |  |  |  |  |
| 6 | Pb.c000801360.Contig1 | 5300 | - | 97564 | 102754 |  |  |  |  |  |  |  |  |  |  |  |  |  |  |
| 6 | MALPY01346 | 3942 | + | 97909 | 101690 |  |  |  |  |  | 915 | 307 |  |  |  |  |  |  |  |
| 6 | MALPY00927 | 9641 | + | 101596 | 111153 |  |  |  |  |  | 915 |  |  |  |  |  |  |  |  |
| 6 | PcContig454 | 2141 | + | 102069 | 104311 |  |  |  |  |  |  |  |  |  |  |  |  |  |  |
| 6 | PbContig163 | 6253 | - | 102642 | 108889 |  |  |  |  |  |  |  |  |  |  |  |  |  |  |
| 6 | PcContig641 | 3361 | - | 104379 | 107847 | cRMPc01005 | 14152 | 2366 | 97001 | 111153 |  |  |  | cRMPs0101 | 111153 | 2366 | 0 | 111153 |  |
| TK6 | MALPY01735 | 5046 | - | 113519 | 119936 | cRMPc01006 | 6417 | 7 | 113519 | 119936 | 165 |  |  |  |  |  |  |  |  |
| 6 | MALPY03032 | 1752 | + | 119943 | 121689 | cRMPc01007 | 1746 | 626 | 119943 | 121689 | 165 |  |  | cRMPs0102 | 8170 | 626 | 113519 | 121689 |  |
| 6 | Pc.c001103697.Contig1 | 7117 | - | 122315 | 129554 |  |  |  |  |  |  |  |  |  |  |  |  |  |  |
| 6 | MALPY01955 | 3563 | + | 122543 | 126103 |  |  |  |  |  |  | 341 | 2200 |  |  |  |  |  |  |
| 6 | MALPY01771 | 6152 | + | 127893 | 134228 | cRMPc01008 | 11913 | 4011 | 122315 | 134228 |  | 341 |  | cRMPs0103 | 11913 | 4011 | 122315 | 134228 |  |
| man6 | MALPY01494 - Ins | 10122 | + | 138239 | 148907 |  |  |  |  |  |  |  |  |  |  |  |  |  |  |
| man6 | Pb.c001602074.Contig2 - Ins | 2211 | - | 144736 | 147507 |  |  |  |  |  |  |  |  |  |  |  |  |  |  |
| man6 | PcContig774 - Ins | 3308 | - | 145068 | 149078 |  |  |  |  |  |  |  |  |  |  |  |  |  |  |
| 6 | Pb.c001402318.Contig1 | 1348 | - | 147239 | 148581 | cRMPc01009 | 10839 | 2625 | 138239 | 149078 |  |  |  | cRMPs0104 | 10839 | 2625 | 138239 | 149078 |  |
| 6 | MALPY01098 | 3777 | - | 151703 | 155483 |  |  |  |  |  | 111 |  |  |  |  |  |  |  |  |
| 6 | Pc.c001504956.Contig1 | 3835 | - | 153158 | 156948 |  |  |  |  |  |  |  |  |  |  |  |  |  |  |
| 6 | Pb.c001101960.Contig1 | 3773 | - | 154287 | 157980 |  |  |  |  |  |  |  |  |  |  |  |  |  |  |
| 6 | MALPY03002 | 1921 | + | 155325 | 157207 | cRMPc01010 | 6277 | 35 | 151703 | 157980 | 111 | 29 | 2200 |  |  |  |  |  |  |
| 6 | Pb.c000601613.Contig1 | 4053 | - | 158015 | 162122 |  |  |  |  |  |  |  |  |  |  |  |  |  |  |
| 6 | MALPY01521 | 4304 | - | 158383 | 162717 |  |  |  |  |  | 88 | 29 |  |  |  |  |  |  |  |
| 6 | PcContig287 | 4924 | - | 160246 | 165337 |  |  |  |  |  |  |  |  |  |  |  |  |  |  |
| 6 | PbContig181 | 5816 | + | 162397 | 168424 |  |  |  |  |  |  |  |  |  |  |  |  |  |  |
| 6 | MALPY01806 | 7219 | + | 162458 | 169426 |  |  |  |  |  | 88 |  |  |  |  |  |  |  |  |
| 6 | Pc.c000503033.Contig1 | 4752 | - | 165489 | 170103 | cRMPc01011 | 12088 | 322 | 158015 | 170103 |  |  |  |  |  |  |  |  |  |
| 6 | MALPY00466 | 6603 | + | 170425 | 177028 | cRMPc01012 | 6603 | 980 | 170425 | 177028 | 88 | 285 | 1800 |  |  |  |  |  |  |
| 6 | MALPY00170 | 12419 | - | 178008 | 191517 |  |  |  |  |  |  | 285 | 8000 |  |  |  |  |  |  |
| 6 | Pc.c001504920.Contig1 | 3292 | + | 184251 | 187508 |  |  |  |  |  |  |  |  |  |  |  |  |  |  |
| 6 | Pb.c000501609.Contig1 | 1156 | - | 185731 | 186881 | cRMPc01013 | 13509 | 1307 | 178008 | 191517 |  |  |  |  |  |  |  |  |  |
| 6 | Pc.c000803182.Contig1 | 9401 | - | 192824 | 203839 |  |  |  |  |  |  |  |  |  |  |  |  |  |  |
| TK6 | MALPY00896 | 2236 | + | 198725 | 201058 |  |  |  |  |  | 14 |  |  |  |  |  |  |  |  |
| 6 | Pb.c000301504.Contig1 | 2244 | - | 199689 | 202011 |  |  |  |  |  |  |  |  |  |  |  |  |  |  |
| 6 | MALPY00895 | 4439 | - | 200505 | 204609 |  |  |  |  |  | 14 | 285 |  |  |  |  |  |  |  |
| 6 | Pb.c000701585.Contig1 | 3212 | - | 202140 | 205265 |  |  |  |  |  |  |  |  |  |  |  |  |  |  |
| 6 | PcContig186 | 12795 | + | 204578 | 219649 |  |  |  |  |  |  |  |  |  |  |  |  |  |  |
| 6 | MALPY00277 | 20590 | - | 204892 | 228460 |  |  |  |  |  | 14 |  |  |  |  |  |  |  |  |
| 6 | PbContig6 | 3657 | - | 206129 | 209797 |  |  |  |  |  |  |  |  |  |  |  |  |  |  |
| 6 | Pb.c001001740.Contig1 | 3365 | - | 209848 | 213210 |  |  |  |  |  |  |  |  |  |  |  |  |  |  |
| 6 | Pb.c001803368.Contig1 | 1145 | - | 213838 | 214974 |  |  |  |  |  |  |  |  |  |  |  |  |  |  |
| 6 | Pb.c001202026.Contig1 | 4783 | - | 216490 | 221309 |  |  |  |  |  |  |  |  |  |  |  |  |  |  |
| 6 | Pc.c001103764.Contig1 | 2994 | + | 221450 | 224444 |  |  |  |  |  |  |  |  |  |  |  |  |  |  |
| 6 | Pb.c001101842.Contig1 | 2252 | - | 223839 | 226350 |  |  |  |  |  |  |  |  |  |  |  |  |  |  |
| 6 | PcContig271 | 4989 | + | 224540 | 229924 |  |  |  |  |  |  |  |  |  |  |  |  |  |  |
| 6 | Pb.c001001703.Contig1 | 1066 | - | 226562 | 227628 |  |  |  |  |  |  |  |  |  |  |  |  |  |  |
| 6 | Pb.c000301479.Contig1 | 2194 | - | 227745 | 229884 |  |  |  |  |  |  |  |  |  |  |  |  |  |  |
| man6 | MALPY00060 - Ins | 2899 | - | 228377 | 231334 |  |  |  |  |  | 14 | 340 | 1400 |  |  |  |  |  |  |
| 6 | PbContig149 | 7624 | + | 229343 | 237501 |  |  |  |  |  |  |  |  |  |  |  |  |  |  |
| 6 | Pc.c000403165.Contig1 | 1021 | + | 229735 | 230787 |  |  |  |  |  |  |  |  |  |  |  |  |  |  |
| 6 | Pc.c001405089.Contig1 | 1345 | - | 229753 | 231129 |  |  |  |  |  |  |  |  |  |  |  |  |  |  |
| 6 | MALPY01914 | 5476 | - | 232512 | 238060 |  |  |  |  |  | 340 | 340 |  |  |  |  |  |  |  |
| 6 | PcContig618 | 3544 | - | 234527 | 238086 | cRMPc01014 | 45262 | 204 | 192824 | 238086 |  |  |  |  |  |  |  |  |  |
| 6 | MALPY00180 | 5327 | + | 238290 | 243842 |  |  |  |  |  | 340 |  |  |  |  |  |  |  |  |
| 6 | Pb.c001803816.Contig1 | 637 | - | 241438 | 242072 | cRMPc01015 | 5552 | 52 | 238290 | 243842 |  |  |  |  |  |  |  |  |  |
| 6 | Pb.c001803376.Contig1 | 1877 | - | 243894 | 245747 |  |  |  |  |  |  |  |  |  |  |  |  |  |  |
| 6 | MALPY00695 | 5401 | + | 244148 | 249409 |  |  |  |  |  | 340 | 19 | 1000 |  |  |  |  |  |  |
| 6 | Pc.c000202852.Contig1 | 2894 | - | 244404 | 247175 |  |  |  |  |  |  |  |  |  |  |  |  |  |  |
| 6 | Pb.c002006888.Contig1 | 2493 | - | 245618 | 248250 |  |  |  |  |  |  |  |  |  |  |  |  |  |  |
| 6 | PcContig622 | 1406 | - | 247526 | 248982 |  |  |  |  |  |  |  |  |  |  |  |  |  |  |
| 6 | Pb.c002007474.Contig1 | 889 | - | 248428 | 249409 | cRMPc01016 | 5515 | 418 | 243894 | 249409 |  |  |  |  |  |  |  |  |  |
| 6 | Pc.c000403359.Contig1 | 7938 | - | 249827 | 258263 |  |  |  |  |  |  |  |  |  |  |  |  |  |  |
| 6 | MALPY00368 | 9033 | - | 250229 | 259524 |  |  |  |  |  |  | 19 | 2100 |  |  |  |  |  |  |
| 6 | Pb.c000701572.Contig1 | 1843 | - | 252244 | 254384 |  |  |  |  |  |  |  |  |  |  |  |  |  |  |
| 6 | Pb.c000101415.Contig1 | 3678 | - | 254520 | 258318 | cRMPc01017 | 9697 | 1608 | 249827 | 259524 |  |  |  |  |  |  |  |  |  |
| 6 | MALPY01504 | 12840 | - | 261132 | 275343 |  |  |  |  |  |  | 19 | 450 |  |  |  |  |  |  |
| 6 | Pc.c000403354.Contig1 | 1072 | + | 262339 | 263573 |  |  |  |  |  |  |  |  |  |  |  |  |  |  |
| 6 | Pb.c000401741.Contig1 | 2568 | + | 263233 | 265888 |  |  |  |  |  |  |  |  |  |  |  |  |  |  |
| 6 | Pc.c000503177.Contig1 | 3929 | + | 263855 | 267784 |  |  |  |  |  |  |  |  |  |  |  |  |  |  |
| 6 | Pb.c001402118.Contig1 | 3003 | - | 269453 | 272459 |  |  |  |  |  |  |  |  |  |  |  |  |  |  |
| 6 | Pc.c001708881.Contig1 | 578 | + | 270664 | 271251 |  |  |  |  |  |  |  |  |  |  |  |  |  |  |
| 6 | Pb.c001101869.Contig1 | 3200 | - | 272441 | 275564 |  |  |  |  |  |  |  |  |  |  |  |  |  |  |
| 6 | Pc.c000002112.Contig1 | 2702 | + | 273571 | 276306 |  |  |  |  |  |  |  |  |  |  |  |  |  |  |
| 6 | MALPY00724 | 5649 | + | 275104 | 280753 |  |  |  |  |  | 58 | 19 |  |  |  |  |  |  |  |
| 6 | Pb.c001101870.Contig1 | 4186 | + | 275628 | 279865 |  |  |  |  |  |  |  |  |  |  |  |  |  |  |
| 6 | PcContig436 | 4302 | - | 278660 | 282962 |  |  |  |  |  |  |  |  |  |  |  |  |  |  |
| 6 | PbContig367 | 3652 | + | 280347 | 283999 |  |  |  |  |  |  |  |  |  |  |  |  |  |  |
| 6 | MALPY00864 | 11126 | - | 281092 | 293302 |  |  |  |  |  | 58 |  |  |  |  |  |  |  |  |
| 6 | Pc.c000302959.Contig1 | 5228 | - | 285061 | 290470 |  |  |  |  |  |  |  |  |  |  |  |  |  |  |
| 6 | PbContig357 | 8715 | + | 285627 | 294951 |  |  |  |  |  |  |  |  |  |  |  |  |  |  |
| 6 | Pc.c000002707.Contig1 | 3432 | - | 291172 | 294788 |  |  |  |  |  |  |  |  |  |  |  |  |  |  |
| 6 | MALPY00619 | 5531 | - | 294196 | 299727 |  |  |  |  |  | 58 |  |  |  |  |  |  |  |  |
| 6 | Pb.c001001749.Contig1 | 4250 | + | 296921 | 301269 |  |  |  |  |  |  |  |  |  |  |  |  |  |  |
| 6 | Pc.c000503591.Contig1 | 590 | + | 298267 | 298857 |  |  |  |  |  |  |  |  |  |  |  |  |  |  |
| 6 | Pc.c000303005.Contig1 | 5099 | + | 299225 | 304725 |  |  |  |  |  |  |  |  |  |  |  |  |  |  |
| 6 | MALPY00033 | 9480 | + | 299927 | 309398 |  |  |  |  |  | 58 | 317 | 2300 |  |  |  |  |  |  |
| 6 | Pb.c001001750.Contig1 | 2629 | + | 301588 | 304226 | cRMPc01018 | 48266 | 354 | 261132 | 309398 |  |  |  |  |  |  |  |  |  |
| 6 | Pc.c001103672.Contig1 | 4761 | + | 309752 | 314976 |  |  |  |  |  |  |  |  |  |  |  |  |  |  |
| 6 | Pb.c000301363.Contig1 | 2215 | - | 311277 | 313498 |  |  |  |  |  |  |  |  |  |  |  |  |  |  |
| 6 | MALPY02773 | 2862 | - | 312255 | 315393 |  |  |  |  |  |  | 317 | 900 |  |  |  |  |  |  |
| 6 | Pb.c000301653.Contig1 | 1170 | - | 313430 | 314600 | cRMPc01019 | 5641 | 34 | 309752 | 315393 |  |  |  |  |  |  |  |  |  |
| 6 | Pb.c000901424.Contig1 | 5742 | - | 315427 | 321374 |  |  |  |  |  |  |  |  |  |  |  |  |  |  |
| 6 | Pc.c001104281.Contig1 | 1275 | + | 315443 | 316820 |  |  |  |  |  |  |  |  |  |  |  |  |  |  |
| 6 | MALPY01068 | 5415 | + | 315623 | 321073 |  |  |  |  |  |  | 317 | 2400 |  |  |  |  |  |  |
| 6 | Pc.c000203207.Contig1 | 1267 | - | 318090 | 319429 |  |  |  |  |  |  |  |  |  |  |  |  |  |  |
| 6 | Pc.c001505395.Contig1 | 897 | - | 319804 | 320695 | cRMPc01020 | 5947 | 3747 | 315427 | 321374 |  |  |  |  |  |  |  |  |  |
| 6 | MALPY01529 | 2050 | + | 325121 | 327231 |  |  |  |  |  | 64 | 317 |  |  |  |  |  |  |  |
| 6 | MALPY01218 | 3987 | + | 326363 | 330254 |  |  |  |  |  | 64 |  |  |  |  |  |  |  |  |
| 6 | PcContig12 | 2761 | - | 326759 | 329383 |  |  |  |  |  |  |  |  |  |  |  |  |  |  |
| 6 | Pb.c000501452.Contig1 | 4101 | - | 328338 | 332439 |  |  |  |  |  |  |  |  |  |  |  |  |  |  |
| 6 | MALPY00349 | 7002 | - | 328605 | 335607 |  |  |  |  |  | 64 |  |  |  |  |  |  |  |  |
| 6 | PcContig455 | 1424 | - | 329582 | 331006 |  |  |  |  |  |  |  |  |  |  |  |  |  |  |
| 6 | PcContig180 | 12163 | - | 333380 | 345537 |  |  |  |  |  |  |  |  |  |  |  |  |  |  |
| 6 | MALPY02426 | 5773 | - | 336718 | 342455 |  |  |  |  |  | 64 |  |  |  |  |  |  |  |  |
| 6 | Pb.c001301887.Contig1 | 5596 | + | 342502 | 347984 |  |  |  |  |  |  |  |  |  |  |  |  |  |  |
| 6 | MALPY01367 | 6039 | - | 342700 | 348637 |  |  |  |  |  | 64 - Ins |  |  |  |  |  |  |  |  |
| 6 | Pb.c001502614.Contig1 | 4024 | + | 347507 | 351534 |  |  |  |  |  |  |  |  |  |  |  |  |  |  |
| 6 | MALPY00536 | 8349 | + | 349137 | 357637 |  |  |  |  |  | 64 - Ins |  |  |  |  |  |  |  |  |
| 6 | Pb.c001202112.Contig1 | 3986 | + | 353671 | 357823 |  |  |  |  |  |  |  |  |  |  |  |  |  |  |
| 6 | Pc.c001405392.Contig1 | 2645 | - | 354552 | 357226 |  |  |  |  |  |  |  |  |  |  |  |  |  |  |
| 6 | MALPY03430 | 378 | - | 355864 | 356242 |  |  |  |  |  |  | 233 | 2500 |  |  |  |  |  |  |
| 6 | PcContig777 | 8208 | - | 357639 | 366923 |  |  |  |  |  |  |  |  |  |  |  |  |  |  |
| 6 | Pb.c001502382.Contig1 | 2963 | + | 358276 | 361212 |  |  |  |  |  |  |  |  |  |  |  |  |  |  |
| 6 | MALPY01335 | 16820 | + | 359485 | 377996 |  |  |  |  |  | 526 | 233 |  |  |  |  |  |  |  |
| 6 | Pb.c000801749.Contig1 | 6706 | + | 363378 | 371007 |  |  |  |  |  |  |  |  |  |  |  |  |  |  |
| 6 | Pc.c001203916.Contig1 | 2378 | - | 367450 | 370306 |  |  |  |  |  |  |  |  |  |  |  |  |  |  |
| 6 | MALPY03257 | 819 | + | 367527 | 368351 |  |  |  |  |  |  |  |  |  |  |  |  |  |  |
| 6 | MALPY03572 | 835 | - | 367604 | 368444 |  |  |  |  |  |  | 189\_alt | 2100 |  |  |  |  |  |  |
| 6 | Pc.c000302867.Contig1 | 2901 | + | 370779 | 373980 |  |  |  |  |  |  |  |  |  |  |  |  |  |  |
| 6 | Pb.c001702883.Contig1 | 1941 | + | 371367 | 373320 |  |  |  |  |  |  |  |  |  |  |  |  |  |  |
| 6 | Pb.c000001804.Contig1 | 3528 | - | 375718 | 379231 |  |  |  |  |  |  |  |  |  |  |  |  |  |  |
| 6 | MALPY00138 | 3466 | + | 378996 | 382560 |  |  |  |  |  | 526 - Ins | 189\_alt |  |  |  |  |  |  |  |
| 6 | Pb.c000701469.Contig1 | 3641 | + | 379536 | 383177 |  |  |  |  |  |  |  |  |  |  |  |  |  |  |
| 6 | MALPY00884 | 22454 | + | 383060 | 407415 |  |  |  |  |  | 526 - Ins |  |  |  |  |  |  |  |  |
| 6 | Pc.c001002686.Contig1 | 3431 | - | 383552 | 386968 |  |  |  |  |  |  |  |  |  |  |  |  |  |  |
| 6 | PbContig310 | 2175 | - | 383906 | 386040 |  |  |  |  |  |  |  |  |  |  |  |  |  |  |
| 6 | MALPY03355 | 488 | - | 384231 | 384716 |  |  |  |  |  |  | 220 | 800 |  |  |  |  |  |  |
| JC6 | MALPY03279 | 349 | + | 385236 | 385579 |  |  |  |  |  |  | 220 |  |  |  |  |  |  |  |
| 6 | Pb.c002006840.Contig1 | 1793 | - | 386051 | 387844 |  |  |  |  |  |  |  |  |  |  |  |  |  |  |
| 6 | Pc.c001812744.Contig1 | 1040 | - | 387663 | 388709 |  |  |  |  |  |  |  |  |  |  |  |  |  |  |
| 6 | Pb.c001202000.Contig1 | 2471 | - | 388539 | 391187 |  |  |  |  |  |  |  |  |  |  |  |  |  |  |
| 6 | Pc.c001708954.Contig1 | 643 | - | 388796 | 389427 |  |  |  |  |  |  |  |  |  |  |  |  |  |  |
| 6 | Pc.c001103802.Contig1 | 1079 | - | 389745 | 390854 |  |  |  |  |  |  |  |  |  |  |  |  |  |  |
| 6 | Pc.c001304648.Contig1 | 817 | - | 394408 | 395225 |  |  |  |  |  |  |  |  |  |  |  |  |  |  |
| 6 | Pb.c001301840.Contig1 | 7457 | + | 394839 | 402599 |  |  |  |  |  |  |  |  |  |  |  |  |  |  |
| 6 | PcContig326 | 3029 | - | 396899 | 400024 |  |  |  |  |  |  |  |  |  |  |  |  |  |  |
| 6 | Pc.c001606609.Contig1 | 1029 | + | 400396 | 401425 |  |  |  |  |  |  |  |  |  |  |  |  |  |  |
| 6 | Pc.c001606651.Contig1 | 910 | + | 401697 | 402607 |  |  |  |  |  |  |  |  |  |  |  |  |  |  |
| 6 | Pc.c001813247.Contig1 | 638 | + | 403283 | 403855 |  |  |  |  |  |  |  |  |  |  |  |  |  |  |
| 6 | Pc.c001813272.Contig1 | 605 | + | 404443 | 405048 | cRMPc01021 | 82294 | 1826 | 325121 | 407415 |  |  |  | cRMPs0105 | 255712 | 1826 | 151703 | 407415 |  |
| Centromere \* - gap 1826 bp | |  |  | 405000 |  |  |  |  |  |  |  |  |  |  |  |  |  |  |  |
| 6 | MALPY00821 | 6716 | + | 409241 | 415798 |  |  |  |  |  |  |  |  |  |  |  |  |  |  |
| 6 | MALPY03515 | 821 | - | 412150 | 412971 | cRMPc01022 | 6557 | 1138 | 409241 | 415798 |  |  |  | cRMPs0106 | 6557 | 1138 | 409241 | 415798 |  |
| 6 | MALPY00773 | 4526 | + | 416936 | 421462 | cRMPc01023 | 4526 | 12450 | 416936 | 421462 |  |  |  | cRMPs0107 | 4526 | 12450 | 416936 | 421462 |  |
| 6 | MALPY02218 | 6581 | - | 433912 | 440879 |  |  |  |  |  |  | 60 | 900 |  |  |  |  |  |  |
| 6 | Pc.c000803695.Contig1 | 5629 | - | 434303 | 439962 |  |  |  |  |  |  |  |  |  |  |  |  |  |  |
| 6 | Pb.c001101856.Contig1 | 3192 | + | 434765 | 437984 |  |  |  |  |  |  |  |  |  |  |  |  |  |  |
| 6 | MALPY00962 | 14129 | - | 436115 | 450304 |  |  |  |  |  |  | 60 |  |  |  |  |  |  |  |
| 6 | PcContig549 | 5120 | + | 443928 | 449138 |  |  |  |  |  |  |  |  |  |  |  |  |  |  |
| 6 | Pb.c000301123.Contig1 | 3329 | + | 446593 | 449991 |  |  |  |  |  |  |  |  |  |  |  |  |  |  |
| 6 | MALPY03451 | 823 | + | 447093 | 447919 | cRMPc01024 | 16392 | 1270 | 433912 | 450304 |  | 250 | 4000 |  |  |  |  |  |  |
| 6 | MALPY00880 | 14784 | - | 451574 | 465832 |  |  |  |  |  | 26A | 250 |  |  |  |  |  |  |  |
| 6 | Pb.c001101899.Contig1 | 1670 | - | 452482 | 454380 |  |  |  |  |  |  |  |  |  |  |  |  |  |  |
| 6 | Pc.c000402794.Contig1 | 3192 | + | 453691 | 456883 |  |  |  |  |  |  |  |  |  |  |  |  |  |  |
| 6 | Pb.c000501464.Contig1 | 8545 | - | 455212 | 463785 |  |  |  |  |  |  |  |  |  |  |  |  |  |  |
| 6 | Pc.c000702684.Contig1 | 2186 | - | 460141 | 462333 |  |  |  |  |  |  |  |  |  |  |  |  |  |  |
| 6 | Pc.c000702733.Contig2 | 1900 | + | 460426 | 462332 |  |  |  |  |  |  |  |  |  |  |  |  |  |  |
| 6 | Pc.c000803598.Contig1 | 624 | + | 461034 | 461661 |  |  |  |  |  |  |  |  |  |  |  |  |  |  |
| 6 | Pb.c001904938.Contig1 | 933 | - | 463188 | 464234 | cRMPc01025 | 14258 | 580 | 451574 | 465832 |  |  |  |  |  |  |  |  |  |
| TK6 | MALPY01516 | 4926 | - | 466412 | 471249 |  |  |  |  |  | 26A |  |  |  |  |  |  |  |  |
| 6 | Pc.c000803253.Contig1 | 3399 | + | 470247 | 473736 |  |  |  |  |  |  |  |  |  |  |  |  |  |  |
| 6 | Pb.c001301803.Contig1 | 2168 | - | 471202 | 473545 |  |  |  |  |  |  |  |  |  |  |  |  |  |  |
| 6 | MALPY00451 | 10012 | + | 471233 | 481527 |  |  |  |  |  | 26A |  |  |  |  |  |  |  |  |
| 6 | Pb.c001402466.Contig1 | 2345 | - | 475207 | 477543 |  |  |  |  |  |  |  |  |  |  |  |  |  |  |
| 6 | Pc.c000202240.Contig2 | 3778 | + | 475381 | 480177 |  |  |  |  |  |  |  |  |  |  |  |  |  |  |
| 6 | Pc.c000803614.Contig1 | 615 | + | 478843 | 479458 |  |  |  |  |  |  |  |  |  |  |  |  |  |  |
| 6 | PcContig293 | 3514 | + | 479998 | 483512 | cRMPc01026 | 17100 | 390 | 466412 | 483512 |  |  |  |  |  |  |  |  |  |
| 6 | MALPY01606 | 6951 | + | 483902 | 491042 |  |  |  |  |  | 26A | 135 | 3000 |  |  |  |  |  |  |
| 6 | Pc.c001304274.Contig1 | 3680 | + | 486779 | 490612 | cRMPc01027 | 7140 | 7396 | 483902 | 491042 |  |  |  |  |  |  |  |  |  |
| 6 | MALPY00288 | 24146 | - | 498438 | 524978 |  |  |  |  |  | 1145 | 135 |  |  |  |  |  |  |  |
| 6 | Pb.c000301415.Contig1 | 2195 | - | 503711 | 506725 |  |  |  |  |  |  |  |  |  |  |  |  |  |  |
| 6 | Pc.c000202149.Contig1 | 3304 | + | 504060 | 508304 |  |  |  |  |  |  |  |  |  |  |  |  |  |  |
| 6 | Pb.c000301416.Contig1 | 7774 | - | 506961 | 514775 |  |  |  |  |  |  |  |  |  |  |  |  |  |  |
| 6 | PcContig399 | 1581 | + | 508362 | 509949 |  |  |  |  |  |  |  |  |  |  |  |  |  |  |
| 6 | MALPY00177 | 3884 | - | 518968 | 529857 |  |  |  |  |  | 1145 |  |  |  |  |  |  |  |  |
| 6 | Pb.c000301477.Contig1 | 5136 | + | 520456 | 525889 |  |  |  |  |  |  |  |  |  |  |  |  |  |  |
| 6 | Pc.c000703792.Contig1 | 1215 | + | 523512 | 524751 | cRMPc01028 | 31419 | 500 | 498438 | 529857 |  |  |  |  |  |  |  |  |  |
| JC6 | MALPY02601 | 3329 | xxx | 530357 | 533686 | cRMPc01029 | 3329 |  | 530357 | 533686 | 1145 |  |  | cRMPs0108 | 99774 |  | 433912 | 533686 |  |
| Telomere | |  |  |  |  |  |  |  |  |  |  |  |  |  |  |  |  |  |  |
|  |  | Pb Pc Py contig |  |  |  |  | cRMP contig | Gap to next cRMP contig |  |  |  |  |  |  | cRMP scaffold | Gap to next cRMP scaffold |  |  |  |
|  | Number | 215 |  |  |  | Number | 29 | 28 |  |  |  |  |  | Number | 8 | 7 |  |  |  |
|  | Average size (bp) | 4628 |  |  |  | Average size (bp) | 16803 | 1657 |  |  |  |  |  | Average size (bp) | 63581 | 3577 |  |  |  |
|  | Minimum size (bp) | 349 |  |  |  | Minimum size (bp) | 1746 | 7 |  |  |  |  |  | Minimum size (bp) | 4526 | 626 |  |  |  |
|  | Maximum size (bp) | 24146 |  |  |  | Maximum size (bp) | 82294 | 12450 |  |  |  |  |  | Maximum size (bp) | 255712 | 12450 |  |  |  |
|  | Total size (bp) | 995046 |  |  |  | Total size (bp) | 487279 | 46407 |  |  |  |  |  | Total size (bp) | 508644 | 25042 |  |  |  |
|  |  |  |  |  |  |  |  |  |  |  |  |  |  |  |  |  |  |  |  |
