# Supplementary material for: A Plasmodium Whole-Genome Synteny Map: Indels and Synteny Breakpoints as Foci for Species-Specific Genes
Source: PLoS Pathog. 2005 Dec 23;1(4):e44. doi: 10.1371/journal.ppat.0010044 (PMC1317653; doi:10.1371/journal.ppat.0010044)
Supplement: Supplementary file 2 [file ppat.0010044.st018.doc]

Table S18 - RMPchr2


# Table S18 - RMPchr2

|  |  |  |  |  |  |  |  |  |  |  |  |  |  |  |  |  |  |  |  |
| --- | --- | --- | --- | --- | --- | --- | --- | --- | --- | --- | --- | --- | --- | --- | --- | --- | --- | --- | --- |
| Table S18: Tiling path of RMP contigs combined to form cRMP chromosome 2. | | | | | | |  |  |  |  |  |  |  |  |  |  |  |  |  |
|  |  |  |  |  |  |  |  |  |  |  |  |  |  |  |  |  |  |  |  |
| Tiling paths of P. berghei (Pb), P. c. chabaudi (Pc) and P. y. yoelii (Py) contigs combined into composite rodent malaria parasite (cRMP) | | | | | | | | | |  |  |  |  |  |  |  |  |  |  |
| contigs aligned against parts of the 14 chromosomes of P. falciparum (Pf). The cRMP contigs are further linked by physical linkage | | | | | | | | | |  |  |  |  |  |  |  |  |  |  |
| and PCR (see Methods) to form cRMP scaffolds. Different synteny blocks with homology to different P. falciparum chromosomes | | | | | | | | | |  |  |  |  |  |  |  |  |  |  |
| together form the cRMP chromosomes. The tables also show gaps in the alignment (black lines), cRMP telomeres (white bars) and | | | | | | | | | |  |  |  |  |  |  |  |  |  |  |
| predicted centromeres (plum/black bars). Contigs, physical groups or PCR groups interrupted by inter- or intrasyntenic regions are | | | | | | | | | |  |  |  |  |  |  |  |  |  |  |
| printed in bold. A summary of the number of RMP contigs, cRMP contigs and cRMP scaffolds as well as their average, minimum, | | | | | | | | | |  |  |  |  |  |  |  |  |  |  |
| maximum and total sizes is provided. Column headings are as follows: | | | | |  |  |  |  |  |  |  |  |  |  |  |  |  |  |  |
|  |  |  |  |  |  |  |  |  |  |  |  |  |  |  |  |  |  |  |  |
| Pf chr.: P. falciparum chromosome to which the RMP contigs are aligned | | | | | |  |  |  |  |  |  |  |  |  |  |  |  |  |  |
| (#: PROmer analysis� from this study; JC# and TK#: Carlton et al.; man#: manual BLAST analysis; | | | | | | |  |  |  |  |  |  |  |  |  |  |  |  |  |
| i: inversed orientation of the synteny block; colours refer to those used in Fig. 2 of the paper); | | | | | | |  |  |  |  |  |  |  |  |  |  |  |  |  |
| Pb Pc Py contig: names of the aligned RMP contigs; | | |  |  |  |  |  |  |  |  |  |  |  |  |  |  |  |  |  |
| Size: size (bp) of the aligned RMP contigs; | |  |  |  |  |  |  |  |  |  |  |  |  |  |  |  |  |  |  |
| +/-: orientation of the aligned RMP contigs; | |  |  |  |  |  |  |  |  |  |  |  |  |  |  |  |  |  |  |
| Start contig & End contig: estimated start & end coordinates of alignment of the RMP contigs based on the coordinates | | | | | | | | |  |  |  |  |  |  |  |  |  |  |  |
| of PROmer hits corrected for the contig�s size and orientation and presence of inter- and intrasyntenic regions | | | | | | | |  |  |  |  |  |  |  |  |  |  |  |  |
| (the start position of the first RMP contig in the alignment was set at zero); | | | | | |  |  |  |  |  |  |  |  |  |  |  |  |  |  |
| cRMP contig: names of cRMP contigs | |  |  |  |  |  |  |  |  |  |  |  |  |  |  |  |  |  |  |
| (the first 2 digits refer to the cRMP chromosome number and the following 3 digits designate the order of the contigs); | | | | | | | | |  |  |  |  |  |  |  |  |  |  |  |
| cRMP contig size: size of cRMP contigs based on the corrected coordinates of alignment; | | | | | | |  |  |  |  |  |  |  |  |  |  |  |  |  |
| Gap size to next cRMP contig: size of gaps between neighbouring cRMP contigs; | | | | | | |  |  |  |  |  |  |  |  |  |  |  |  |  |
| Start cRMP contig & End cRMP contig: estimated start & end coordinates of alignment of the cRMP contigs; | | | | | | | |  |  |  |  |  |  |  |  |  |  |  |  |
| Phys. group (Py): physical linkage of neighbouring P. y. yoelii contigs; | | | | | |  |  |  |  |  |  |  |  |  |  |  |  |  |  |
| PCR group (Py): neighbouring P. y. yoelii contigs were linked previously or for this study by PCR; | | | | | | |  |  |  |  |  |  |  |  |  |  |  |  |  |
| PCR size (Py): size of the PCR product; | |  |  |  |  |  |  |  |  |  |  |  |  |  |  |  |  |  |  |
| cRMP scaffold: names of cRMP scaffolds | |  |  |  |  |  |  |  |  |  |  |  |  |  |  |  |  |  |  |
| (the first 2 digits refer to the cRMP chromosome number and the following 2 digits designate the order of the scaffolds); | | | | | | | | |  |  |  |  |  |  |  |  |  |  |  |
| cRMP scaffold size: size of cRMP scaffolds; | | |  |  |  |  |  |  |  |  |  |  |  |  |  |  |  |  |  |
| Gap size to next cRMP scaffold: size of gaps between neighbouring cRMP scaffolds; | | | | | | |  |  |  |  |  |  |  |  |  |  |  |  |  |
| Start cRMP scaffold & End cRMP scaffold: estimated start & end coordinates of alignment of the cRMP scaffolds; | | | | | | | | |  |  |  |  |  |  |  |  |  |  |  |
| Overlap: Linkage between RMP contigs established through manual BLAST analysis. | | | | | | |  |  |  |  |  |  |  |  |  |  |  |  |  |
|  |  |  |  |  |  |  |  |  |  |  |  |  |  |  |  |  |  |  |  |
| Pf chr. | Pb Pc Py contig | Size | +/- | Start contig | End contig | cRMP contig | cRMP contig size | Gap size to next cRMP contig | Start cRMP contig | End cRMP contig | Phys. group (Py) | PCR group (Py) | PCR size (Py) | cRMP scaffold | cRMP scaffold size | Gap size to next cRMP scaffold | Start cRMP scaffold | End cRMP scaffold | Overlap (manual) |
| Telomere | |  |  |  |  |  |  |  |  |  |  |  |  |  |  |  |  |  |  |
| Centromere | |  |  |  |  |  |  |  |  |  |  |  |  |  |  |  |  |  |  |
| i1 | MALPY00384 | 4825 | + | 0 | 4946 |  |  |  |  |  | 1239A | 256 | 450 |  |  |  |  |  |  |
| i1 | Pb.c000501475.Contig1 | 3775 | - | 2567 | 6834 |  |  |  |  |  |  |  |  |  |  |  |  |  |  |
| i1 | Pc.c001605839.Contig1 | 999 | + | 3510 | 4527 |  |  |  |  |  |  |  |  |  |  |  |  |  |  |
| i1 | Pc.c001606333.Contig1 | 1347 | + | 4972 | 6319 |  |  |  |  |  |  |  |  |  |  |  |  |  |  |
| i1 | MALPY02016 | 3722 | - | 5342 | 9064 | cRMPc02001 | 9064 | 1759 | 0 | 9064 | 1239A | 256 | 500 |  |  |  |  |  |  |
| i1 | MALPY02965 | 2044 | + | 10823 | 12870 | cRMPc02002 | 2047 | 458 | 10823 | 12870 | 10 | 256 | 3500 |  |  |  |  |  |  |
| i1 | Pb.c001803349.Contig1 | 9349 | + | 13328 | 22711 |  |  |  |  |  |  |  |  |  |  |  |  |  |  |
| i1 | MALPY01800 | 7280 | + | 13395 | 20568 |  |  |  |  |  | 10 | 22 | 600 |  |  |  |  |  |  |
| i1 | Pc.c000702653.Contig1 | 3306 | - | 14211 | 17658 |  |  |  |  |  |  |  |  |  |  |  |  |  |  |
| i1 | MALPY03449 | 487 | + | 17401 | 17888 |  |  |  |  |  |  | 256 |  |  |  |  |  |  |  |
| i1 | Pc.c000703844.Contig1 | 3655 | - | 17889 | 22341 |  |  |  |  |  |  |  |  |  |  |  |  |  |  |
| i1 | MALPY01329 | 6532 | - | 20359 | 26980 |  |  |  |  |  | 10 | 22 | 3000 |  |  |  |  |  |  |
| i1 | Pb.c001803514.Contig1 | 3015 | + | 22930 | 26100 | cRMPc02003 | 13652 | 1606 | 13328 | 26980 |  |  |  |  |  |  |  |  |  |
| i1 | MALPY00652 | 5844 | - | 28586 | 34526 |  |  |  |  |  | 10 | 27 | 600 |  |  |  |  |  |  |
| i1 | PbContig356 | 8269 | - | 28794 | 38356 |  |  |  |  |  |  |  |  |  |  |  |  |  |  |
| i1 | Pc.c000402888.Contig1 | 3786 | - | 30429 | 34329 |  |  |  |  |  |  |  |  |  |  |  |  |  |  |
| i1 | MALPY03422 | 583 | + | 31063 | 31655 |  |  |  |  |  |  | 22 |  |  |  |  |  |  |  |
| i1 | MALPY02664 | 2037 | - | 34975 | 37422 |  |  |  |  |  | 10 | 27 |  |  |  |  |  |  |  |
| i1 | PcContig144 | 1952 | - | 35107 | 37508 |  |  |  |  |  |  |  |  |  |  |  |  |  |  |
| i1 | MALPY02210 | 4396 | - | 35781 | 40251 |  |  |  |  |  | 10 | 65 | 6500 |  |  |  |  |  |  |
| i1 | PbContig191 | 9601 | - | 38837 | 50841 |  |  |  |  |  |  |  |  |  |  |  |  |  |  |
| i1 | MALPY00660 | 10299 | - | 40009 | 52318 |  |  |  |  |  | 149 |  |  |  |  |  |  |  |  |
| i1 | PcContig498 | 10371 | + | 40310 | 52926 |  |  |  |  |  |  |  |  |  |  |  |  |  |  |
| i1 | MALPY03056 | 536 | + | 49613 | 50149 | cRMPc02004 | 24340 | 2478 | 28586 | 52926 |  | 65 |  |  |  |  |  |  |  |
| i1 | MALPY00118 | 18636 | - | 55404 | 74438 |  |  |  |  |  | 149 |  |  |  |  |  |  |  |  |
| i1 | PbContig177 | 11746 | - | 59101 | 71697 |  |  |  |  |  |  |  |  |  |  |  |  |  |  |
| i1 | Pc.c000903874.Contig1 | 1214 | + | 60441 | 61740 |  |  |  |  |  |  |  |  |  |  |  |  |  |  |
| i1 | PcContig276 | 10052 | + | 62367 | 72502 |  |  |  |  |  |  |  |  |  |  |  |  |  |  |
| i1 | Pb.c001301503.Contig1 | 5480 | - | 72144 | 77526 |  |  |  |  |  |  |  |  |  |  |  |  |  |  |
| i1 | Pc.c001103706.Contig1 | 4477 | + | 73644 | 78081 |  |  |  |  |  |  |  |  |  |  |  |  |  |  |
| i1 | MALPY03410 | 1020 | + | 74481 | 75513 |  |  |  |  |  |  | 230 | 1800 |  |  |  |  |  |  |
| i1 | MALPY00378 | 4550 | + | 76475 | 81071 |  |  |  |  |  | 61 | 230 |  |  |  |  |  |  |  |
| i1 | PcContig754 | 1702 | + | 78400 | 80151 |  |  |  |  |  |  |  |  |  |  |  |  |  |  |
| i1 | Pb.c001202028.Contig1 | 1628 | - | 78443 | 80122 | cRMPc02005 | 25667 | 311 | 55404 | 81071 |  |  |  |  |  |  |  |  |  |
| i1 | MALPY00870 | 3360 | + | 81382 | 84745 | cRMPc02006 | 3363 | 571 | 81382 | 84745 | 61 | 63 | 1200 |  |  |  |  |  |  |
| i1 | Pb.c000701602.Contig1 | 3639 | - | 85316 | 88931 |  |  |  |  |  |  |  |  |  |  |  |  |  |  |
| i1 | Pc.c001103685.Contig1 | 3792 | - | 85487 | 89582 |  |  |  |  |  |  |  |  |  |  |  |  |  |  |
| i1 | MALPY02998 | 1997 | + | 85722 | 87695 |  |  |  |  |  | 61 | 29 | 1100 |  |  |  |  |  |  |
| i1 | MALPY03011 | 1602 | + | 86381 | 87983 |  |  |  |  |  | 61 | 63 |  |  |  |  |  |  |  |
| i1 | MALPY02346 | 3248 | + | 88640 | 91740 |  |  |  |  |  | 75 | 29 | 500 |  |  |  |  |  |  |
| i1 | Pb.c000001279.Contig1 | 7992 | - | 89351 | 98690 |  |  |  |  |  |  |  |  |  |  |  |  |  |  |
| i1 | Pc.c001104242.Contig1 | 933 | + | 89652 | 90556 |  |  |  |  |  |  |  |  |  |  |  |  |  |  |
| i1 | Pc.c001706619.Contig1 | 1977 | + | 90985 | 92974 |  |  |  |  |  |  |  |  |  |  |  |  |  |  |
| i1 | MALPY03455 | 886 | - | 91990 | 92882 |  |  |  |  |  |  | 29 |  |  |  |  |  |  |  |
| i1 | MALPY01593 | 4320 | + | 92046 | 97504 |  |  |  |  |  | 75 |  |  |  |  |  |  |  |  |
| iTK1 | MALPY01511 | 4844 | + | 98271 | 103055 | cRMPc02007 | 17739 | 1606 | 85316 | 103055 | 75 |  |  |  |  |  |  |  |  |
| i1 | MALPY00171 | 15668 | - | 104661 | 120416 |  |  |  |  |  | 75 | 54 | 2300 |  |  |  |  |  |  |
| i1 | Pc.c000702695.Contig2 | 10109 | - | 113579 | 125466 |  |  |  |  |  |  |  |  |  |  |  |  |  |  |
| i1 | Pb.c001301843.Contig1 | 1784 | - | 116409 | 118343 |  |  |  |  |  |  |  |  |  |  |  |  |  |  |
| i1 | MALPY03254 | 668 | + | 116813 | 117552 |  |  |  |  |  |  | 221 | 750 |  |  |  |  |  |  |
| i1 | MALPY03488 | 737 | - | 117638 | 118318 |  |  |  |  |  |  | 221 |  |  |  |  |  |  |  |
| i1 | Pb.c001301842.Contig1 | 1546 | - | 118630 | 120176 |  |  |  |  |  |  |  |  |  |  |  |  |  |  |
| i1 | MALPY02068 | 10689 | + | 124149 | 136110 |  |  |  |  |  | 554 | 54 | 1100 |  |  |  |  |  |  |
| i1 | Pb.c000901449.Contig2 | 4079 | + | 125767 | 130674 |  |  |  |  |  |  |  |  |  |  |  |  |  |  |
| i1 | Pc.c001003239.Contig1 | 2638 | - | 125897 | 128575 |  |  |  |  |  |  |  |  |  |  |  |  |  |  |
| i1 | Pc.c001606491.Contig1 | 1081 | - | 129091 | 130427 |  |  |  |  |  |  |  |  |  |  |  |  |  |  |
| i1 | Pc.c000202720.Contig1 | 3485 | - | 130783 | 134672 |  |  |  |  |  |  |  |  |  |  |  |  |  |  |
| i1 | PbContig311 | 6560 | + | 133514 | 139234 |  |  |  |  |  |  |  |  |  |  |  |  |  |  |
| i1 | MALPY02342 | 3881 | + | 135815 | 138919 |  |  |  |  |  | 554 | 24 | 9000 |  |  |  |  |  |  |
| i1 | PcContig71 | 5354 | - | 136221 | 140783 |  |  |  |  |  |  |  |  |  |  |  |  |  |  |
| i1 | MALPY03063 | 772 | + | 136816 | 137588 | cRMPc02008 | 36122 | 4369 | 104661 | 140783 |  | 54 |  |  |  |  |  |  |  |
| i1 | MALPY01184 | 7418 | + | 145152 | 152866 |  |  |  |  |  | 384A | 24 | 350 |  |  |  |  |  |  |
| i1 | PbContig305 | 3201 | - | 146422 | 149671 |  |  |  |  |  |  |  |  |  |  |  |  |  |  |
| i1 | Pc.c001002744.Contig1 | 3832 | + | 147471 | 151714 |  |  |  |  |  |  |  |  |  |  |  |  |  |  |
| i1 | Pb.c000301473.Contig1 | 2683 | - | 149912 | 152889 |  |  |  |  |  |  |  |  |  |  |  |  |  |  |
| i1 | Pc.c000202874.Contig1 | 1933 | + | 152070 | 152070 |  |  |  |  |  |  |  |  |  |  |  |  |  |  |
| iman1 | MALPY02252 - Ins | 4181 | - | 152687 | 157015 |  |  |  |  |  | 384A | 24 | 600 |  |  |  |  |  |  |
| i1 | Pc.c000103062.Contig1 | 1015 | - | 154684 | 155699 | cRMPc02009 | 11863 | 79 | 145152 | 157015 |  |  |  |  |  |  |  |  |  |
| i1 | MALPY02524 | 6344 | + | 157094 | 163291 |  |  |  |  |  | 384A | 24 | 400 |  |  |  |  |  |  |
| i1 | Pc.c001405315.Contig1 | 2467 | - | 157915 | 160396 |  |  |  |  |  |  |  |  |  |  |  |  |  |  |
| i1 | Pc.c001203859.Contig1 | 3032 | - | 161478 | 164519 |  |  |  |  |  |  |  |  |  |  |  |  |  |  |
| i1 | PbContig354 | 7851 | + | 162134 | 162134 |  |  |  |  |  |  |  |  |  |  |  |  |  |  |
| i1 | Pb.c000101374.Contig1 | 4940 | + | 162830 | 167867 |  |  |  |  |  |  |  |  |  |  |  |  |  |  |
| i1 | MALPY00273 | 3538 | + | 163725 | 167457 |  |  |  |  |  | 407 | 24 | 3000 |  |  |  |  |  |  |
| i1 | Pc.c001812912.Contig1 | 1088 | - | 164231 | 165319 |  |  |  |  |  |  |  |  |  |  |  |  |  |  |
| i1 | Pc.c001405055.Contig1 | 1460 | - | 165735 | 167241 |  |  |  |  |  |  |  |  |  |  |  |  |  |  |
| i1 | MALPY01399 | 8498 | - | 167606 | 176770 |  |  |  |  |  | 407 | 158 | 6500 |  |  |  |  |  |  |
| i1 | PcContig161 | 1745 | - | 168194 | 169966 |  |  |  |  |  |  |  |  |  |  |  |  |  |  |
| i1 | Pb.c002007925.Contig1 | 616 | - | 168578 | 169194 |  |  |  |  |  |  |  |  |  |  |  |  |  |  |
| i1 | Pb.c000501402.Contig1 | 2658 | - | 170057 | 172778 |  |  |  |  |  |  |  |  |  |  |  |  |  |  |
| i1 | Pc.c001606018.Contig1 | 8566 | - | 170788 | 179402 |  |  |  |  |  |  |  |  |  |  |  |  |  |  |
| i1 | MALPY03432 | 378 | - | 171798 | 172176 |  |  |  |  |  |  | 24 |  |  |  |  |  |  |  |
| i1 | MALPY00620 | 6148 | + | 176774 | 182305 |  |  |  |  |  | 407 |  |  |  |  |  |  |  |  |
| i1 | Pb.c002005812.Contig1 | 3686 | - | 178449 | 182135 |  |  |  |  |  |  |  |  |  |  |  |  |  |  |
| i1 | Pc.c000503139.Contig1 | 1406 | - | 179831 | 181237 |  |  |  |  |  |  |  |  |  |  |  |  |  |  |
| i1 | PcContig728 | 4031 | - | 181738 | 185772 |  |  |  |  |  |  |  |  |  |  |  |  |  |  |
| i1 | Pb.c000501403.Contig1 | 5224 | + | 183770 | 189399 |  |  |  |  |  |  |  |  |  |  |  |  |  |  |
| i1 | MALPY01887 | 10457 | - | 184162 | 196407 |  |  |  |  |  | 1321 | 158 | 500 |  |  |  |  |  |  |
| i1 | Pc.c000903444.Contig1 | 757 | - | 191979 | 192736 |  |  |  |  |  |  |  |  |  |  |  |  |  |  |
| i1 | Pb.c001402406.Contig1 | 1066 | + | 191982 | 193048 |  |  |  |  |  |  |  |  |  |  |  |  |  |  |
| i1 | Pb.c000401451.Contig1 | 3028 | - | 194116 | 197825 |  |  |  |  |  |  |  |  |  |  |  |  |  |  |
| i1 | MALPY02407 | 3950 | - | 197270 | 201441 |  |  |  |  |  | 1321 | 158 | 3000 |  |  |  |  |  |  |
| i1 | Pb.c000401169.Contig2 | 3443 | - | 198144 | 201587 |  |  |  |  |  |  |  |  |  |  |  |  |  |  |
| i1 | Pc.c001605969.Contig1 | 2422 | + | 199282 | 201707 | cRMPc02010 | 44613 | 142 | 157094 | 201707 |  |  |  |  |  |  |  |  |  |
| i1 | MALPY01308 | 9441 | - | 201849 | 211368 |  |  |  |  |  | 260 | 172 | 400 |  |  |  |  |  |  |
| i1 | Pb.c000901776.Contig1 | 10134 | + | 202452 | 212664 |  |  |  |  |  |  |  |  |  |  |  |  |  |  |
| i1 | MALPY03379 | 785 | - | 204349 | 205134 | cRMPc02011 | 10815 | 1321 | 201849 | 212664 |  | 158 |  |  |  |  |  |  |  |
| i1 | MALPY01472 | 12868 | + | 213985 | 226732 |  |  |  |  |  | 260 | 172 | 1000 |  |  |  |  |  |  |
| i1 | PbContig347 | 8978 | - | 217809 | 226766 |  |  |  |  |  |  |  |  |  |  |  |  |  |  |
| i1 | PcContig656 | 2063 | + | 222567 | 224609 |  |  |  |  |  |  |  |  |  |  |  |  |  |  |
| i1 | Pc.c001003277.Contig1 | 3420 | - | 225870 | 229293 |  |  |  |  |  |  |  |  |  |  |  |  |  |  |
| i1 | Pb.c001502380.Contig1 | 9448 | - | 227658 | 238565 |  |  |  |  |  |  |  |  |  |  |  |  |  |  |
| i1 | MALPY03378 | 1008 | - | 228173 | 229184 |  |  |  |  |  | 260 | 172 | 300 |  |  |  |  |  |  |
| i1 | MALPY03145 | 1399 | - | 229111 | 230507 |  |  |  |  |  | 260 | 172 | 350 |  |  |  |  |  |  |
| i1 | MALPY02961 | 2258 | - | 230617 | 232955 |  |  |  |  |  |  | 172 | 250 |  |  |  |  |  |  |
| i1 | Pc.c000602348.Contig1 | 3858 | + | 232175 | 237551 |  |  |  |  |  |  |  |  |  |  |  |  |  |  |
| i1 | MALPY02673 | 4530 | - | 233904 | 238677 |  |  |  |  |  | 119 | 172 |  |  |  |  |  |  |  |
| i1 | Pc.c000603425.Contig1 | 872 | - | 234881 | 235762 |  |  |  |  |  |  |  |  |  |  |  |  |  |  |
| i1 | Pc.c001103674.Contig1 | 3649 | + | 235274 | 239139 | cRMPc02012 | 25154 | 1049 | 213985 | 239139 |  |  |  |  |  |  |  |  |  |
| i1 | MALPY01642 | 5692 | - | 240188 | 246109 |  |  |  |  |  | 119 |  |  |  |  |  |  |  |  |
| i1 | Pc.c001812934.Contig1 | 522 | + | 240786 | 241308 |  |  |  |  |  |  |  |  |  |  |  |  |  |  |
| i1 | Pc.c001812935.Contig1 | 812 | + | 241566 | 242390 |  |  |  |  |  |  |  |  |  |  |  |  |  |  |
| i1 | Pb.c000401453.Contig1 | 5570 | + | 241931 | 247369 |  |  |  |  |  |  |  |  |  |  |  |  |  |  |
| i1 | PcContig537 | 4779 | + | 242454 | 247089 |  |  |  |  |  |  |  |  |  |  |  |  |  |  |
| i1 | MALPY00327 | 12251 | - | 247118 | 260254 |  |  |  |  |  | 119 |  |  |  |  |  |  |  |  |
| i1 | Pc.c001003283.Contig1 | 2449 | + | 248210 | 250849 |  |  |  |  |  |  |  |  |  |  |  |  |  |  |
| i1 | Pb.c000301484.Contig1 | 3211 | + | 248398 | 251730 |  |  |  |  |  |  |  |  |  |  |  |  |  |  |
| i1 | Pc.c000303386.Contig1 | 1510 | - | 253069 | 254534 |  |  |  |  |  |  |  |  |  |  |  |  |  |  |
| i1 | Pb.c001904355.Contig1 | 4840 | - | 254369 | 259416 |  |  |  |  |  |  |  |  |  |  |  |  |  |  |
| i1 | Pc.c000502940.Contig1 | 1589 | - | 255582 | 257328 |  |  |  |  |  |  |  |  |  |  |  |  |  |  |
| i1 | Pc.c000502938.Contig1 | 1035 | - | 257424 | 258459 |  |  |  |  |  |  |  |  |  |  |  |  |  |  |
| iTK1 | MALPY02449 | 2967 | + | 260142 | 263052 |  |  |  |  |  | 119 |  |  |  |  |  |  |  |  |
| iman1 | MALPY02122 | 2575 | + | 261449 | 264035 | cRMPc02013 | 23847 | 5531 | 240188 | 264035 | 119 |  |  |  |  |  |  |  |  |
| iTK1 | MALPY02121 | 5545 | - | 269566 | 275900 | cRMPc02014 | 6334 | 335 | 269566 | 275900 | 119 |  |  | cRMPs0201 | 275900 | 335 | 0 | 275900 |  |
| iman1 | MALPY02534 | 2752 | + | 276235 | 278692 |  |  |  |  |  | 3 |  |  |  |  |  |  |  |  |
| i1 | MALPY01244 | 10812 | + | 278291 | 288531 |  |  |  |  |  | 3 |  |  |  |  |  |  |  |  |
| i1 | Pb.c001502620.Contig1 | 5610 | + | 279766 | 285418 |  |  |  |  |  |  |  |  |  |  |  |  |  |  |
| i1 | Pc.c001303494.Contig2 | 8766 | + | 281076 | 290211 |  |  |  |  |  |  |  |  |  |  |  |  |  |  |
| i1 | MALPY03239 | 583 | - | 282886 | 283469 |  |  |  |  |  |  |  |  |  |  |  |  |  |  |
| i1 | PbContig184 | 2817 | + | 287034 | 289851 |  |  |  |  |  |  |  |  |  |  |  |  |  |  |
| iTK1 | MALPY01497 | 9925 | + | 288900 | 300062 |  |  |  |  |  | 3 |  |  |  |  |  |  |  |  |
| i1 | PbContig262 | 8753 | + | 297820 | 307188 |  |  |  |  |  |  |  |  |  |  |  |  |  |  |
| i1 | MALPY01129 | 9774 | - | 300819 | 311420 |  |  |  |  |  | 3 | 84 | 600 |  |  |  |  |  |  |
| i1 | Pc.c000202196.Contig4 | 5006 | - | 300946 | 306298 |  |  |  |  |  |  |  |  |  |  |  |  |  |  |
| i1 | Pc.c001103860.Contig1 | 2473 | - | 308149 | 310631 |  |  |  |  |  |  |  |  |  |  |  |  |  |  |
| i1 | Pb.c001702884.Contig1 | 2330 | - | 308348 | 310687 |  |  |  |  |  |  |  |  |  |  |  |  |  |  |
| i1 | MALPY02220 | 4722 | - | 311055 | 315792 |  |  |  |  |  | 3 | 84 | 3000 |  |  |  |  |  |  |
| i1 | PcContig111 | 7375 | - | 311157 | 320165 |  |  |  |  |  |  |  |  |  |  |  |  |  |  |
| i1 | Pb.c000201553.Contig1 | 2530 | + | 312479 | 315078 |  |  |  |  |  |  |  |  |  |  |  |  |  |  |
| iTK1 | MALPY00774 | 2072 | - | 316819 | 318603 |  |  |  |  |  | 3 |  |  |  |  |  |  |  |  |
| i1 | MALPY00402 | 7116 | - | 319566 | 326415 |  |  |  |  |  |  | 84 |  |  |  |  |  |  |  |
| i1 | Pb.c000901747.Contig1 | 2676 | + | 319966 | 322642 |  |  |  |  |  |  |  |  |  |  |  |  |  |  |
| i1 | PcContig105 | 1284 | - | 320532 | 321816 |  |  |  |  |  |  |  |  |  |  |  |  |  |  |
| i1 | Pc.c001708849.Contig1 | 692 | - | 322467 | 323165 |  |  |  |  |  |  |  |  |  |  |  |  |  |  |
| i1 | Pb.c000801717.Contig1 | 4074 | + | 322575 | 326654 |  |  |  |  |  |  |  |  |  |  |  |  |  |  |
| i1 | Pc.c000903281.Contig1 | 2903 | - | 323517 | 326420 |  |  |  |  |  |  |  |  |  |  |  |  |  |  |
| i1 | MALPY03484 | 715 | + | 324380 | 325095 |  |  |  |  |  |  |  |  |  |  |  |  |  |  |
| man1 | MALPY00382 - BP | 29838 | + | 325522 | 360971 |  |  |  |  |  |  |  |  |  |  |  |  |  |  |
| 7 | PcContig33 | 4916 | - | 327039 | 332766 |  |  |  |  |  |  |  |  |  |  |  |  |  |  |
| 7 | PbContig174 | 6132 | + | 327504 | 334398 |  |  |  |  |  |  |  |  |  |  |  |  |  |  |
| 7 | Pc.c000703319.Contig1 | 1104 | - | 343040 | 344141 |  |  |  |  |  |  |  |  |  |  |  |  |  |  |
| 7 | PbContig78 | 7350 | - | 349342 | 357471 |  |  |  |  |  |  |  |  |  |  |  |  |  |  |
| 7 | PcContig752 | 7235 | - | 349411 | 357586 |  |  |  |  |  |  |  |  |  |  |  |  |  |  |
| 7 | MALPY03576 | 1410 | + | 350894 | 352336 |  |  |  |  |  |  |  |  |  |  |  |  |  |  |
| 7 | Pc.c001812590.Contig1 | 1026 | + | 357713 | 358757 |  |  |  |  |  |  |  |  |  |  |  |  |  |  |
| 7 | Pb.c001904502.Contig1 | 1563 | + | 357889 | 360055 |  |  |  |  |  |  |  |  |  |  |  |  |  |  |
| 7 | Pc.c001707884.Contig1 | 1421 | + | 360243 | 361520 | cRMPc02015 | 85285 | 3380 | 276235 | 361520 |  |  |  | cRMPs0202 | 85285 | 3380 | 276235 | 361520 |  |
| 7 | PcContig606 | 6193 | - | 364900 | 371662 |  |  |  |  |  |  |  |  |  |  |  |  |  |  |
| 7 | MALPY00694 | 3060 | - | 365137 | 368440 |  |  |  |  |  |  |  |  |  |  |  |  |  |  |
| 7 | Pb.c001803469.Contig1 | 1967 | - | 365355 | 367322 |  |  |  |  |  |  |  |  |  |  |  |  |  |  |
| 7 | MALPY00575 | 24610 | - | 366926 | 392826 |  |  |  |  |  |  |  |  |  |  |  |  |  |  |
| 7 | Pb.c000601622.Contig1 | 2148 | + | 370112 | 372254 |  |  |  |  |  |  |  |  |  |  |  |  |  |  |
| 7 | Pb.c001001746.Contig1 | 12409 | - | 375341 | 388494 |  |  |  |  |  |  |  |  |  |  |  |  |  |  |
| 7 | PcContig196 | 8037 | - | 376002 | 384546 |  |  |  |  |  |  |  |  |  |  |  |  |  |  |
| 7 | Pc.c001304096.Contig1 | 757 | - | 381875 | 382647 |  |  |  |  |  |  |  |  |  |  |  |  |  |  |
| 7 | Pc.c001304357.Contig1 | 2183 | - | 387162 | 389500 | cRMPc02016 | 27926 | 150 | 364900 | 392826 |  |  |  | cRMPs0203 | 27926 | 150 | 364900 | 392826 |  |
| 7 | MALPY00338 | 5159 | + | 392976 | 398138 |  |  |  |  |  |  | 348 | 1600 |  |  |  |  |  |  |
| 7 | Pc.c001505757.Contig1 | 677 | + | 395716 | 396480 | cRMPc02017 | 5162 | 1357 | 392976 | 398138 |  |  |  |  |  |  |  |  |  |
| 7 | MALPY00385 | 7268 | - | 399495 | 407285 |  |  |  |  |  | 743 | 348 |  |  |  |  |  |  |  |
| 7 | Pb.c001803870.Contig1 | 642 | - | 401598 | 402243 |  |  |  |  |  |  |  |  |  |  |  |  |  |  |
| 7 | Pb.c000001701.Contig1 | 2504 | - | 403742 | 406312 |  |  |  |  |  |  |  |  |  |  |  |  |  |  |
| 7 | Pc.c000102465.Contig1 | 2883 | - | 404096 | 407024 | cRMPc02018 | 7790 | 867 | 399495 | 407285 |  |  |  |  |  |  |  |  |  |
| 7 | MALPY00027 | 9622 | + | 408152 | 417568 |  |  |  |  |  | 743 |  |  |  |  |  |  |  |  |
| 7 | Pc.c001812438.Contig1 | 757 | - | 411760 | 412517 |  |  |  |  |  |  |  |  |  |  |  |  |  |  |
| 7 | Pc.c000002042.Contig1 | 5557 | - | 413608 | 418874 |  |  |  |  |  |  |  |  |  |  |  |  |  |  |
| 7 | Pb.c000101614.Contig1 | 955 | - | 413991 | 414946 |  |  |  |  |  |  |  |  |  |  |  |  |  |  |
| 7 | Pb.c000701545.Contig1 | 2837 | - | 414951 | 417627 |  |  |  |  |  |  |  |  |  |  |  |  |  |  |
| 7 | MALPY01240 | 3996 | + | 418409 | 422276 |  |  |  |  |  | 302 |  |  |  |  |  |  |  |  |
| 7 | Pb.c002112309.Contig1 | 1006 | - | 420964 | 421913 | cRMPc02019 | 14124 | 702 | 408152 | 422276 |  |  |  |  |  |  |  |  |  |
| 7 | MALPY00406 | 4832 | + | 422978 | 427945 |  |  |  |  |  | 302 |  |  |  |  |  |  |  |  |
| 7 | Pc.c001606530.Contig1 | 1013 | - | 424833 | 425882 |  |  |  |  |  |  |  |  |  |  |  |  |  |  |
| 7 | Pc.c000702685.Contig1 | 3143 | - | 426255 | 429398 |  |  |  |  |  |  |  |  |  |  |  |  |  |  |
| 7 | MALPY02984 | 1121 | - | 428155 | 429276 |  |  |  |  |  | 302 |  |  |  |  |  |  |  |  |
| 7 | PbContig300 | 3499 | - | 428387 | 431886 |  |  |  |  |  |  |  |  |  |  |  |  |  |  |
| 7 | MALPY00344 | 11744 | + | 429467 | 440966 |  |  |  |  |  | 302 |  |  |  |  |  |  |  |  |
| 7 | Pb.c000701523.Contig1 | 3940 | + | 436213 | 439805 | cRMPc02020 | 17988 | 4300 | 422978 | 440966 |  |  |  | cRMPs0204 | 47990 | 4300 | 392976 | 440966 |  |
| 7 | Pb.c000501206.Contig3 | 9447 | + | 445266 | 454651 |  |  |  |  |  |  |  |  |  |  |  |  |  |  |
| 7 | MALPY00838 | 8112 | - | 445401 | 453331 |  |  |  |  |  | 298 |  |  |  |  |  |  |  |  |
| 7 | PcContig517 | 2320 | + | 447555 | 449848 |  |  |  |  |  |  |  |  |  |  |  |  |  |  |
| 7 | Pc.c000803416.Contig1 | 3876 | + | 451052 | 454931 |  |  |  |  |  |  |  |  |  |  |  |  |  |  |
| 7 | MALPY00395 | 51301 | - | 453955 | 505650 |  |  |  |  |  | 298 |  |  |  |  |  |  |  |  |
| 7 | Pb.c000301462.Contig1 | 3182 | + | 454963 | 458148 |  |  |  |  |  |  |  |  |  |  |  |  |  |  |
| 7 | Pc.c000003006.Contig1 | 2430 | + | 455704 | 458134 |  |  |  |  |  |  |  |  |  |  |  |  |  |  |
| 7 | Pb.c000601255.Contig2 | 11235 | - | 465935 | 477628 |  |  |  |  |  |  |  |  |  |  |  |  |  |  |
| 7 | PcContig556 | 7445 | + | 466793 | 474299 |  |  |  |  |  |  |  |  |  |  |  |  |  |  |
| 7 | Pc.c000503503.Contig1 | 903 | + | 474480 | 475347 |  |  |  |  |  |  |  |  |  |  |  |  |  |  |
| 7 | Pb.c001904789.Contig1 | 1104 | - | 477379 | 478483 |  |  |  |  |  |  |  |  |  |  |  |  |  |  |
| 7 | Pb.c000701482.Contig1 | 584 | + | 477698 | 478282 |  |  |  |  |  |  |  |  |  |  |  |  |  |  |
| 7 | PbContig14 | 37075 | - | 477886 | 515013 |  |  |  |  |  |  |  |  |  |  |  |  |  |  |
| 7 | Pc.c000202745.Contig1 | 2523 | + | 481566 | 483834 |  |  |  |  |  |  |  |  |  |  |  |  |  |  |
| 7 | Pc.c001813091.Contig1 | 662 | - | 489815 | 490495 |  |  |  |  |  |  |  |  |  |  |  |  |  |  |
| 7 | PcContig325 | 3206 | - | 490924 | 494112 |  |  |  |  |  |  |  |  |  |  |  |  |  |  |
| 7 | Pc.c000303027.Contig1 | 4842 | - | 494305 | 499060 |  |  |  |  |  |  |  |  |  |  |  |  |  |  |
| 7 | Pc.c001404804.Contig1 | 1432 | - | 499099 | 500531 |  |  |  |  |  |  |  |  |  |  |  |  |  |  |
| 7 | Pc.c000603016.Contig1 | 1542 | - | 501471 | 503001 |  |  |  |  |  |  |  |  |  |  |  |  |  |  |
| 7 | MALPY03176 | 183 | + | 503158 | 503341 |  |  |  |  |  |  |  |  |  |  |  |  |  |  |
| 7 | MALPY03403 | 318 | - | 503197 | 503515 |  |  |  |  |  |  |  |  |  |  |  |  |  |  |
| 7 | Pc.c000002700.Contig1 | 2292 | - | 503862 | 506363 |  |  |  |  |  |  |  |  |  |  |  |  |  |  |
| 7 | MALPY00850 | 10395 | - | 505254 | 515958 |  |  |  |  |  | 298 |  |  |  |  |  |  |  |  |
| 7 | PcContig743 | 5634 | - | 506184 | 511819 |  |  |  |  |  |  |  |  |  |  |  |  |  |  |
| 7 | MALPY03282 | 251 | - | 508526 | 508786 |  |  |  |  |  |  | 12 | 2000 |  |  |  |  |  |  |
| 7 | MALPY03246 | 762 | - | 510274 | 511033 |  |  |  |  |  |  | 12 | 1900 |  |  |  |  |  |  |
| 7 | PcContig251 | 2031 | + | 511205 | 513426 |  |  |  |  |  |  |  |  |  |  |  |  |  |  |
| 7 | MALPY02991 | 660 | + | 512594 | 513248 | cRMPc02021 | 70692 | 11058 | 445266 | 515958 |  | 12 |  | cRMPs0205 | 70692 | 11058 | 445266 | 515958 |  |
| 7 | MALPY00426 | 6008 | - | 527016 | 533057 |  |  |  |  |  |  |  |  |  |  |  |  |  |  |
| 7 | Pb.c000701476.Contig1 | 4412 | - | 528755 | 533200 |  |  |  |  |  |  |  |  |  |  |  |  |  |  |
| 7 | PcContig102 | 6806 | + | 528971 | 536613 |  |  |  |  |  |  |  |  |  |  |  |  |  |  |
| 7 | Pb.c001903639.Contig2 | 1862 | + | 534196 | 536052 |  |  |  |  |  |  |  |  |  |  |  |  |  |  |
| man7 | MALPY00837 - Ins | 2743 | + | 534243 | 537342 |  |  |  |  |  |  |  |  |  |  |  |  |  |  |
| 7 | PbContig337 | 9940 | + | 536773 | 546667 |  |  |  |  |  |  |  |  |  |  |  |  |  |  |
| 7 | Pc.c000202724.Contig1 | 2633 | + | 537313 | 539928 |  |  |  |  |  |  |  |  |  |  |  |  |  |  |
| man7 | MALPY01395 | 9109 | - | 537681 | 546790 |  |  |  |  |  |  |  |  |  |  |  |  |  | PbContig337 |
| man7 | PbContig6426 | 1495 | - | 546612 | 548107 |  |  |  |  |  |  |  |  |  |  |  |  |  | \| |
| man7 | MALPY02541 | 12895 | + | 547596 | 560491 | cRMPc02022 | 33475 |  | 527016 | 560491 |  |  |  | cRMPs0206 | 33475 |  | 527016 | 560491 | \| |
| Telomere | |  |  |  |  |  |  |  |  |  |  |  |  |  |  |  |  |  |  |
|  |  | Pb Pc Py contig |  |  |  |  | cRMP contig | Gap to next cRMP contig |  |  |  |  |  |  | cRMP scaffold | Gap to next cRMP scaffold |  |  |  |
|  | Number | 222 |  |  |  | Number | 22 | 21 |  |  |  |  |  | Number | 6 | 5 |  |  |  |
|  | Average size (bp) | 4645 |  |  |  | Average size (bp) | 23503 | 2068 |  |  |  |  |  | Average size (bp) | 90211 | 3845 |  |  |  |
|  | Minimum size (bp) | 183 |  |  |  | Minimum size (bp) | 2047 | 79 |  |  |  |  |  | Minimum size (bp) | 27926 | 150 |  |  |  |
|  | Maximum size (bp) | 51301 |  |  |  | Maximum size (bp) | 85285 | 11058 |  |  |  |  |  | Maximum size (bp) | 275900 | 11058 |  |  |  |
|  | Total size (bp) | 1031181 |  |  |  | Total size (bp) | 517062 | 43429 |  |  |  |  |  | Total size (bp) | 541268 | 19223 |  |  |  |
|  |  |  |  |  |  |  |  |  |  |  |  |  |  |  |  |  |  |  |  |
