# Supplementary material for: A Plasmodium Whole-Genome Synteny Map: Indels and Synteny Breakpoints as Foci for Species-Specific Genes
Source: PLoS Pathog. 2005 Dec 23;1(4):e44. doi: 10.1371/journal.ppat.0010044 (PMC1317653; doi:10.1371/journal.ppat.0010044)
Supplement: Supplementary file 3 [file ppat.0010044.st019.doc]

Table S19 - RMPchr3


# Table S19 - RMPchr3

|  |  |  |  |  |  |  |  |  |  |  |  |  |  |  |  |  |  |  |  |
| --- | --- | --- | --- | --- | --- | --- | --- | --- | --- | --- | --- | --- | --- | --- | --- | --- | --- | --- | --- |
| Table S19: Tiling path of RMP contigs combined to form cRMP chromosome 3. | | | | | | |  |  |  |  |  |  |  |  |  |  |  |  |  |
|  |  |  |  |  |  |  |  |  |  |  |  |  |  |  |  |  |  |  |  |
| Tiling paths of P. berghei (Pb), P. c. chabaudi (Pc) and P. y. yoelii (Py) contigs combined into composite rodent malaria parasite (cRMP) | | | | | | | | | |  |  |  |  |  |  |  |  |  |  |
| contigs aligned against parts of the 14 chromosomes of P. falciparum (Pf). The cRMP contigs are further linked by physical linkage | | | | | | | | | |  |  |  |  |  |  |  |  |  |  |
| and PCR (see Methods) to form cRMP scaffolds. Different synteny blocks with homology to different P. falciparum chromosomes | | | | | | | | | |  |  |  |  |  |  |  |  |  |  |
| together form the cRMP chromosomes. The tables also show gaps in the alignment (black lines), cRMP telomeres (white bars) and | | | | | | | | | |  |  |  |  |  |  |  |  |  |  |
| predicted centromeres (plum/black bars). Contigs, physical groups or PCR groups interrupted by inter- or intrasyntenic regions are | | | | | | | | | |  |  |  |  |  |  |  |  |  |  |
| printed in bold. A summary of the number of RMP contigs, cRMP contigs and cRMP scaffolds as well as their average, minimum, | | | | | | | | | |  |  |  |  |  |  |  |  |  |  |
| maximum and total sizes is provided. Column headings are as follows: | | | | |  |  |  |  |  |  |  |  |  |  |  |  |  |  |  |
|  |  |  |  |  |  |  |  |  |  |  |  |  |  |  |  |  |  |  |  |
| Pf chr.: P. falciparum chromosome to which the RMP contigs are aligned | | | | | |  |  |  |  |  |  |  |  |  |  |  |  |  |  |
| (#: PROmer analysis� from this study; JC# and TK#: Carlton et al.; man#: manual BLAST analysis; | | | | | | |  |  |  |  |  |  |  |  |  |  |  |  |  |
| i: inversed orientation of the synteny block; colours refer to those used in Fig. 2 of the paper); | | | | | | |  |  |  |  |  |  |  |  |  |  |  |  |  |
| Pb Pc Py contig: names of the aligned RMP contigs; | | |  |  |  |  |  |  |  |  |  |  |  |  |  |  |  |  |  |
| Size: size (bp) of the aligned RMP contigs; | |  |  |  |  |  |  |  |  |  |  |  |  |  |  |  |  |  |  |
| +/-: orientation of the aligned RMP contigs; | |  |  |  |  |  |  |  |  |  |  |  |  |  |  |  |  |  |  |
| Start contig & End contig: estimated start & end coordinates of alignment of the RMP contigs based on the coordinates | | | | | | | | |  |  |  |  |  |  |  |  |  |  |  |
| of PROmer hits corrected for the contig�s size and orientation and presence of inter- and intrasyntenic regions | | | | | | | |  |  |  |  |  |  |  |  |  |  |  |  |
| (the start position of the first RMP contig in the alignment was set at zero); | | | | | |  |  |  |  |  |  |  |  |  |  |  |  |  |  |
| cRMP contig: names of cRMP contigs | |  |  |  |  |  |  |  |  |  |  |  |  |  |  |  |  |  |  |
| (the first 2 digits refer to the cRMP chromosome number and the following 3 digits designate the order of the contigs); | | | | | | | | |  |  |  |  |  |  |  |  |  |  |  |
| cRMP contig size: size of cRMP contigs based on the corrected coordinates of alignment; | | | | | | |  |  |  |  |  |  |  |  |  |  |  |  |  |
| Gap size to next cRMP contig: size of gaps between neighbouring cRMP contigs; | | | | | | |  |  |  |  |  |  |  |  |  |  |  |  |  |
| Start cRMP contig & End cRMP contig: estimated start & end coordinates of alignment of the cRMP contigs; | | | | | | | |  |  |  |  |  |  |  |  |  |  |  |  |
| Phys. group (Py): physical linkage of neighbouring P. y. yoelii contigs; | | | | | |  |  |  |  |  |  |  |  |  |  |  |  |  |  |
| PCR group (Py): neighbouring P. y. yoelii contigs were linked previously or for this study by PCR; | | | | | | |  |  |  |  |  |  |  |  |  |  |  |  |  |
| PCR size (Py): size of the PCR product; | |  |  |  |  |  |  |  |  |  |  |  |  |  |  |  |  |  |  |
| cRMP scaffold: names of cRMP scaffolds | |  |  |  |  |  |  |  |  |  |  |  |  |  |  |  |  |  |  |
| (the first 2 digits refer to the cRMP chromosome number and the following 2 digits designate the order of the scaffolds); | | | | | | | | |  |  |  |  |  |  |  |  |  |  |  |
| cRMP scaffold size: size of cRMP scaffolds; | | |  |  |  |  |  |  |  |  |  |  |  |  |  |  |  |  |  |
| Gap size to next cRMP scaffold: size of gaps between neighbouring cRMP scaffolds; | | | | | | |  |  |  |  |  |  |  |  |  |  |  |  |  |
| Start cRMP scaffold & End cRMP scaffold: estimated start & end coordinates of alignment of the cRMP scaffolds; | | | | | | | | |  |  |  |  |  |  |  |  |  |  |  |
| Overlap: Linkage between RMP contigs established through manual BLAST analysis. | | | | | | |  |  |  |  |  |  |  |  |  |  |  |  |  |
|  |  |  |  |  |  |  |  |  |  |  |  |  |  |  |  |  |  |  |  |
| Pf chr. | Pb Pc Py contig | Size | +/- | Start contig | End contig | cRMP contig | cRMP contig size | Gap size to next cRMP contig | Start cRMP contig | End cRMP contig | Phys. group (Py) | PCR group (Py) | PCR size (Py) | cRMP scaffold | cRMP scaffold size | Gap size to next cRMP scaffold | Start cRMP scaffold | End cRMP scaffold | Overlap (manual) |
| Telomere | |  |  |  |  |  |  |  |  |  |  |  |  |  |  |  |  |  |  |
| man2 | PbContig4608 | 3821 | + | 0 | 3821 |  |  |  |  |  |  |  |  |  |  |  |  |  |  |
| man2 | PcContig6247 | 4149 | + | 92 | 4241 |  |  |  |  |  |  |  |  |  |  |  |  |  |  |
| man2 | MALPY00125 - Ins | 10347 | + | 359 | 10871 |  |  |  |  |  |  | 331 | 6500 |  |  |  |  |  |  |
| 2 | Pb.c000101034.Contig1 | 7132 | + | 435 | 7540 |  |  |  |  |  |  |  |  |  |  |  |  |  |  |
| 2 | Pc.c001405448.Contig1 | 604 | + | 6838 | 7439 |  |  |  |  |  |  |  |  |  |  |  |  |  |  |
| man2 | MALPY01266 | 4363 | - | 10676 | 15072 | cRMPc03001 | 15072 | 8904 | 0 | 15072 |  |  |  |  |  |  |  |  |  |
| 2 | PcContig179 | 6614 | - | 23976 | 31731 |  |  |  |  |  |  |  |  |  |  |  |  |  |  |
| 2 | Pb.c001301862.Contig1 | 1136 | + | 25218 | 26351 |  |  |  |  |  |  |  |  |  |  |  |  |  |  |
| 2 | MALPY02793 | 2656 | - | 25448 | 28104 |  |  |  |  |  | 70 | 331 |  |  |  |  |  |  |  |
| 2 | PbContig13 | 8290 | + | 27621 | 36047 |  |  |  |  |  |  |  |  |  |  |  |  |  |  |
| 2 | MALPY02428 | 2973 | - | 28154 | 31557 |  |  |  |  |  | 70 |  |  |  |  |  |  |  |  |
| 2 | MALPY00407 | 6636 | + | 31213 | 38933 |  |  |  |  |  | 70 |  |  |  |  |  |  |  |  |
| 2 | Pc.c001606286.Contig1 | 932 | - | 31688 | 32620 |  |  |  |  |  |  |  |  |  |  |  |  |  |  |
| 2 | Pb.c001502009.Contig1 | 7687 | + | 37220 | 46156 |  |  |  |  |  |  |  |  |  |  |  |  |  |  |
| 2 | MALPY02357 | 4224 | + | 39236 | 43793 |  |  |  |  |  | 70 |  |  |  |  |  |  |  |  |
| 2 | Pc.c001204082.Contig1 | 2191 | - | 39952 | 42547 |  |  |  |  |  |  |  |  |  |  |  |  |  |  |
| 2 | MALPY00454 | 5234 | + | 44145 | 49686 |  |  |  |  |  | 70 |  |  |  |  |  |  |  |  |
| 2 | PcContig760 | 4256 | + | 46534 | 51216 |  |  |  |  |  |  |  |  |  |  |  |  |  |  |
| 2 | Pb.c001001754.Contig1 | 6028 | + | 48357 | 55417 |  |  |  |  |  |  |  |  |  |  |  |  |  |  |
| 2 | MALPY00933 | 4904 | - | 49887 | 55695 |  |  |  |  |  | 70 | 146 | 4000 |  |  |  |  |  |  |
| 2 | Pc.c000302915.Contig1 | 623 | + | 51630 | 52265 |  |  |  |  |  |  |  |  |  |  |  |  |  |  |
| 2 | Pc.c000602324.Contig3 | 3540 | + | 51670 | 55565 | cRMPc03002 | 31719 | 4200 | 23976 | 55695 |  |  |  |  |  |  |  |  |  |
| man2 | MALPY00241 - Ins | 25879 | + | 59895 | 86200 |  |  |  |  |  | 118 | 146 | 1100 |  |  |  |  |  |  |
| 2 | Pb.c000901885.Contig1 | 2647 | + | 65175 | 67420 |  |  |  |  |  |  |  |  |  |  |  |  |  |  |
| 2 | Pb.c001803344.Contig1 | 5820 | + | 68904 | 75995 |  |  |  |  |  |  |  |  |  |  |  |  |  |  |
| 2 | PcContig141 | 1054 | - | 69666 | 71332 |  |  |  |  |  |  |  |  |  |  |  |  |  |  |
| 2 | Pc.c000502927.Contig1 | 2487 | - | 72160 | 74647 |  |  |  |  |  |  |  |  |  |  |  |  |  |  |
| 2 | Pc.c000503078.Contig1 | 4128 | - | 74739 | 78867 |  |  |  |  |  |  |  |  |  |  |  |  |  |  |
| man2 | Pb.c001301738.Contig1 - Ins | 8114 | + | 77104 | 85827 |  |  |  |  |  |  |  |  |  |  |  |  |  |  |
| 2 | Pc.c000803433.Contig1 | 2894 | - | 79542 | 82421 | cRMPc03003 | 26305 | 1001 | 59895 | 86200 |  |  |  |  |  |  |  |  |  |
| 2 | MALPY00693 | 7956 | - | 87201 | 95416 |  |  |  |  |  | 858A | 146 | 300 |  |  |  |  |  |  |
| 2 | Pb.c001904256.Contig1 | 4041 | + | 89756 | 94139 |  |  |  |  |  |  |  |  |  |  |  |  |  |  |
| 2 | Pc.c000702731.Contig2 | 4838 | - | 90338 | 95488 |  |  |  |  |  |  |  |  |  |  |  |  |  |  |
| 2 | Pc.c000702712.Contig2 | 681 | - | 91864 | 92633 |  |  |  |  |  |  |  |  |  |  |  |  |  |  |
| 2 | Pb.c001904386.Contig1 | 1980 | - | 93232 | 95161 |  |  |  |  |  |  |  |  |  |  |  |  |  |  |
| 2 | MALPY03234 | 847 | - | 93819 | 94666 |  |  |  |  |  |  | 146 | 250 |  |  |  |  |  |  |
| 2 | Pc.c001813308.Contig1 | 766 | - | 94474 | 95189 |  |  |  |  |  |  |  |  |  |  |  |  |  |  |
| 2 | MALPY00298 | 14313 | - | 94735 | 109341 |  |  |  |  |  |  | 146 | 10000 |  |  |  |  |  |  |
| 2 | Pc.c000202902.Contig1 | 2975 | + | 95337 | 98312 |  |  |  |  |  |  |  |  |  |  |  |  |  |  |
| 2 | Pb.c001402419.Contig1 | 662 | - | 95702 | 96364 |  |  |  |  |  |  |  |  |  |  |  |  |  |  |
| 2 | PbContig402 | 14909 | - | 96705 | 113646 |  |  |  |  |  |  |  |  |  |  |  |  |  |  |
| 2 | Pc.c000103094.Contig1 | 1178 | + | 98870 | 100040 |  |  |  |  |  |  |  |  |  |  |  |  |  |  |
| 2 | Pc.c000903354.Contig1 | 7449 | - | 102604 | 111091 |  |  |  |  |  |  |  |  |  |  |  |  |  |  |
| 2 | MALPY03513 | 866 | - | 103224 | 104038 |  |  |  |  |  |  | 146 | 300 |  |  |  |  |  |  |
| 2 | MALPY03161 | 381 | + | 104125 | 104506 |  |  |  |  |  |  | 146 | 4000 |  |  |  |  |  |  |
| 2 | MALPY03354 | 770 | - | 109691 | 110458 |  |  |  |  |  |  | 146 | 500 |  |  |  |  |  |  |
| 2 | MALPY01582 | 9655 | + | 111288 | 121036 |  |  |  |  |  | 118 | 146 |  |  |  |  |  |  |  |
| 2 | Pb.c001702403.Contig2 | 3013 | + | 114055 | 117176 |  |  |  |  |  |  |  |  |  |  |  |  |  |  |
| 2 | MALPY03380 | 836 | + | 115235 | 116068 |  |  |  |  |  |  | 343 | 10000 |  |  |  |  |  |  |
| 2 | Pc.c000402773.Contig1 | 1068 | - | 115292 | 116432 |  |  |  |  |  |  |  |  |  |  |  |  |  |  |
| 2 | Pc.c000503441.Contig1 | 756 | - | 116533 | 117289 | cRMPc03004 | 33835 | 3182 | 87201 | 121036 |  |  |  |  |  |  |  |  |  |
| 2 | PcContig672 | 9160 | - | 124218 | 133372 |  |  |  |  |  |  |  |  |  |  |  |  |  |  |
| TK2 | MALPY00018 | 6790 | + | 124376 | 132192 | cRMPc03005 | 9154 | 674 | 124218 | 133372 | 118 |  |  |  |  |  |  |  |  |
| 2 | MALPY00017 | 3510 | + | 134046 | 137556 |  |  |  |  |  | 118 | 343 |  |  |  |  |  |  |  |
| 2 | Pb.c000801443.Contig2 | 7504 | + | 134614 | 142155 |  |  |  |  |  |  |  |  |  |  |  |  |  |  |
| 2 | PcContig336 | 3700 | + | 136319 | 140138 |  |  |  |  |  |  |  |  |  |  |  |  |  |  |
| 2 | MALPY01971 | 2594 | - | 137477 | 140077 |  |  |  |  |  | 118 |  |  |  |  |  |  |  |  |
| man2 | MALPY02806 - Exp | 2159 | - | 137852 | 140969 | cRMPc03006 | 8109 | 5574 | 134046 | 142155 | 118 | 257 | 6000 |  |  |  |  |  |  |
| 2 | MALPY00568 | 11014 | - | 147729 | 162426 |  |  |  |  |  | 346 | 257 |  |  |  |  |  |  |  |
| 2 | Pb.c001001748.Contig1 | 7084 | - | 148121 | 155195 |  |  |  |  |  |  |  |  |  |  |  |  |  |  |
| 2 | PcContig139 | 1754 | + | 149626 | 151380 |  |  |  |  |  |  |  |  |  |  |  |  |  |  |
| 2 | Pc.c001304684.Contig1 | 733 | + | 152690 | 153420 |  |  |  |  |  |  |  |  |  |  |  |  |  |  |
| 2 | Pb.c000401497.Contig1 | 4193 | + | 155858 | 163795 |  |  |  |  |  |  |  |  |  |  |  |  |  |  |
| 2 | Pc.c000703940.Contig1 | 692 | + | 156646 | 157349 |  |  |  |  |  |  |  |  |  |  |  |  |  |  |
| man2 | MALPY00082 - Exp | 18877 | - | 158194 | 175373 |  |  |  |  |  | 346 | 297 | 1100 |  |  |  |  |  |  |
| 2 | Pb.c001702316.Contig2 | 1969 | + | 160282 | 162251 |  |  |  |  |  |  |  |  |  |  |  |  |  |  |
| man2 | Pb.c001001366.Contig2 - Exp | 21025 | - | 160705 | 181732 |  |  |  |  |  |  |  |  |  |  |  |  |  |  |
| 2 | Pc.c001104131.Contig1 | 1382 | + | 165485 | 166909 |  |  |  |  |  |  |  |  |  |  |  |  |  |  |
| 2 | MALPY02114 | 6279 | - | 176681 | 183526 |  |  |  |  |  | 483 | 297 |  |  |  |  |  |  |  |
| 2 | Pc.c000003121.Contig1 | 1145 | + | 178716 | 179956 |  |  |  |  |  |  |  |  |  |  |  |  |  |  |
| 2 | Pc.c000002710.Contig1 | 2351 | + | 179967 | 182516 |  |  |  |  |  |  |  |  |  |  |  |  |  |  |
| 2 | PbContig206 | 4587 | + | 183121 | 187902 |  |  |  |  |  |  |  |  |  |  |  |  |  |  |
| 2 | MALPY01010 | 9907 | - | 183447 | 193426 |  |  |  |  |  | 483 | 349 | 600 |  |  |  |  |  |  |
| 2 | PcContig479 | 4339 | - | 183705 | 188126 |  |  |  |  |  |  |  |  |  |  |  |  |  |  |
| 2 | Pb.c000101347.Contig1 | 3964 | - | 190678 | 194735 | cRMPc03007 | 47006 | 1626 | 147729 | 194735 |  |  |  |  |  |  |  |  |  |
| 2 | MALPY00569 | 2128 | - | 196361 | 198489 |  |  |  |  |  | 253A | 349 |  |  |  |  |  |  |  |
| 2 | Pb.c000101346.Contig1 | 2862 | - | 196747 | 199612 | cRMPc03008 | 3251 | 985 | 196361 | 199612 |  |  |  |  |  |  |  |  |  |
| 2 | MALPY01476 | 5451 | - | 200597 | 206246 |  |  |  |  |  | 253A |  |  |  |  |  |  |  |  |
| 2 | Pb.c001602396.Contig1 | 3106 | - | 201075 | 204181 |  |  |  |  |  |  |  |  |  |  |  |  |  |  |
| 2 | Pc.c001203216.Contig1 | 1794 | - | 202626 | 204420 |  |  |  |  |  |  |  |  |  |  |  |  |  |  |
| 2 | Pb.c001602366.Contig1 | 6377 | - | 204783 | 211160 |  |  |  |  |  |  |  |  |  |  |  |  |  |  |
| 2 | Pc.c001606198.Contig1 | 2589 | - | 204890 | 207479 |  |  |  |  |  |  |  |  |  |  |  |  |  |  |
| TK2 | MALPY01475 | 4695 | - | 206247 | 211217 | cRMPc03009 | 10620 | 230 | 200597 | 211217 |  |  |  |  |  |  |  |  |  |
| 2 | MALPY01147 | 12369 | - | 211447 | 225518 |  |  |  |  |  | 253A | 286 | 2000 |  |  |  |  |  |  |
| 2 | Pb.c002006856.Contig1 | 1942 | + | 212868 | 214816 |  |  |  |  |  |  |  |  |  |  |  |  |  |  |
| 2 | Pc.c000402821.Contig1 | 4211 | - | 213223 | 217650 |  |  |  |  |  |  |  |  |  |  |  |  |  |  |
| 2 | Pb.c000901811.Contig1 | 3002 | + | 214936 | 218067 |  |  |  |  |  |  |  |  |  |  |  |  |  |  |
| 2 | PcContig595 | 5672 | - | 219451 | 225156 |  |  |  |  |  |  |  |  |  |  |  |  |  |  |
| 2 | Pb.c001702739.Contig1 | 6114 | + | 219701 | 225809 | cRMPc03010 | 14362 | 2115 | 211447 | 225809 |  |  |  |  |  |  |  |  |  |
| 2 | MALPY00839 | 5634 | + | 227924 | 233504 |  |  |  |  |  | 1205 | 286 |  |  |  |  |  |  |  |
| 2 | Pb.c001602387.Contig1 | 5035 | - | 228297 | 233326 |  |  |  |  |  |  |  |  |  |  |  |  |  |  |
| 2 | Pc.c001104173.Contig1 | 1685 | + | 230557 | 232239 |  |  |  |  |  |  |  |  |  |  |  |  |  |  |
| 2 | MALPY00091 | 19789 | + | 233289 | 256407 |  |  |  |  |  | 1205 |  |  |  |  |  |  |  |  |
| 2 | Pc.c001103843.Contig1 | 2404 | - | 235315 | 237715 |  |  |  |  |  |  |  |  |  |  |  |  |  |  |
| 2 | Pb.c000101291.Contig1 | 2415 | - | 236649 | 239435 |  |  |  |  |  |  |  |  |  |  |  |  |  |  |
| 2 | MALPY03157 | 841 | + | 236802 | 237643 |  |  |  |  |  |  |  |  |  |  |  |  |  |  |
| 2 | Pc.c000002684.Contig1 | 3144 | - | 238049 | 241054 |  |  |  |  |  |  |  |  |  |  |  |  |  |  |
| 2 | Pb.c000401435.Contig1 | 3400 | - | 240689 | 244089 |  |  |  |  |  |  |  |  |  |  |  |  |  |  |
| 2 | PcContig2 | 6988 | - | 241143 | 248429 |  |  |  |  |  |  |  |  |  |  |  |  |  |  |
| 2 | Pb.c001001688.Contig1 | 8113 | - | 244581 | 256188 |  |  |  |  |  |  |  |  |  |  |  |  |  |  |
| 2 | Pc.c000202805.Contig1 | 1892 | - | 252561 | 254450 | cRMPc03011 | 28483 | 1742 | 227924 | 256407 |  |  |  | cRMPs0301 | 256407 | 1742 | 0 | 256407 |  |
| 2 | Pb.c001201658.Contig1 | 3928 | - | 258149 | 262166 |  |  |  |  |  |  |  |  |  |  |  |  |  |  |
| 2 | MALPY02853 | 2173 | - | 258620 | 260793 |  |  |  |  |  |  | 277 | 600 |  |  |  |  |  |  |
| 2 | PcContig750 | 12111 | + | 260851 | 274672 |  |  |  |  |  |  |  |  |  |  |  |  |  |  |
| 2 | MALPY02192 | 9325 | - | 261075 | 271662 |  |  |  |  |  | 209 | 277 |  |  |  |  |  |  |  |
| 2 | PbContig17 | 6322 | - | 262753 | 269845 |  |  |  |  |  |  |  |  |  |  |  |  |  |  |
| 2 | MALPY03272 | 814 | - | 265471 | 266285 |  |  |  |  |  |  |  |  |  |  |  |  |  |  |
| 2 | MALPY00124 | 3521 | + | 271300 | 274852 |  |  |  |  |  | 209 |  |  |  |  |  |  |  |  |
| 2 | PbContig275 | 5453 | + | 271399 | 276994 |  |  |  |  |  |  |  |  |  |  |  |  |  |  |
| 2 | PcContig78 | 6546 | + | 274888 | 282611 |  |  |  |  |  |  |  |  |  |  |  |  |  |  |
| 2 | MALPY00123 | 8249 | - | 276253 | 284570 |  |  |  |  |  | 209 |  |  |  |  |  |  |  |  |
| 2 | Pb.c001202107.Contig1 | 5167 | + | 278553 | 283821 | cRMPc03012 | 26421 | 352 | 258149 | 284570 |  |  |  |  |  |  |  |  |  |
| 2 | PcContig284 | 891 | + | 284922 | 285813 |  |  |  |  |  |  |  |  |  |  |  |  |  |  |
| 2 | MALPY03043 | 1045 | - | 285028 | 286073 | cRMPc03013 | 1151 | 3500 | 284922 | 286073 | 209 | 104 - NS | 3500 |  |  |  |  |  |  |
| 2 | MALPY03206 | 1086 | + | 289573 | 290662 |  |  |  |  |  |  | 104 - NS | 4000 |  |  |  |  |  |  |
| 2 | Pb.c001904474.Contig1 | 1264 | - | 290319 | 291583 | cRMPc03014 | 2010 | 3079 | 289573 | 291583 |  |  |  |  |  |  |  |  |  |
| Centromere - PCR 4000 bp | |  |  | 292000 |  |  |  |  |  |  |  |  |  |  |  |  |  |  |  |
| 2 | MALPY01600 | 4215 | - | 294662 | 298892 |  |  |  |  |  |  | 104 | 350 |  |  |  |  |  |  |
| 2 | Pc.c001606569.Contig1 | 611 | - | 298036 | 298662 | cRMPc03015 | 4230 | 69 | 294662 | 298892 |  |  |  |  |  |  |  |  |  |
| 2 | MALPY01301 | 5128 | - | 298961 | 303964 |  |  |  |  |  | 517A | 104 |  |  |  |  |  |  |  |
| 2 | Pb.c001301448.Contig2 | 5815 | + | 300408 | 306418 |  |  |  |  |  |  |  |  |  |  |  |  |  |  |
| 2 | MALPY03174 | 660 | - | 300755 | 301436 |  |  |  |  |  |  |  |  |  |  |  |  |  |  |
| 2 | MALPY03175 | 484 | - | 300929 | 301434 |  |  |  |  |  |  | 118 | 4300 |  |  |  |  |  |  |
| 2 | Pc.c001812501.Contig1 | 774 | + | 302067 | 302819 |  |  |  |  |  |  |  |  |  |  |  |  |  |  |
| 2 | PcContig771 | 3096 | + | 303762 | 307212 |  |  |  |  |  |  |  |  |  |  |  |  |  |  |
| 2 | MALPY03556 | 398 | + | 304844 | 305242 |  |  |  |  |  |  | 118 | 500 |  |  |  |  |  |  |
| 2 | MALPY03058 | 454 | + | 305377 | 305831 |  |  |  |  |  |  | 118 |  |  |  |  |  |  |  |
| 2 | MALPY03523 | 784 | - | 305894 | 306678 |  |  |  |  |  |  |  |  |  |  |  |  |  |  |
| 2 | MALPY01415 | 7640 | + | 306801 | 314441 |  |  |  |  |  | 1154 |  |  |  |  |  |  |  |  |
| 2 | Pb.c001702749.Contig1 | 3335 | + | 307003 | 310338 |  |  |  |  |  |  |  |  |  |  |  |  |  |  |
| 2 | Pc.c000703846.Contig1 | 994 | + | 307962 | 308956 |  |  |  |  |  |  |  |  |  |  |  |  |  |  |
| 2 | Pc.c000703304.Contig1 | 1974 | + | 309029 | 311003 | cRMPc03016 | 15480 | 743 | 298961 | 314441 |  |  |  |  |  |  |  |  |  |
| 2 | Pb.c000501429.Contig1 | 5018 | + | 315184 | 320181 |  |  |  |  |  |  |  |  |  |  |  |  |  |  |
| 2 | Pc.c000702725.Contig1 | 3765 | - | 315973 | 319759 |  |  |  |  |  |  |  |  |  |  |  |  |  |  |
| 2 | MALPY00688 | 5651 | + | 315980 | 322184 |  |  |  |  |  | 1154 |  |  |  |  |  |  |  |  |
| 2 | Pb.c001001448.Contig1 | 5156 | - | 317323 | 323126 |  |  |  |  |  |  |  |  |  |  |  |  |  |  |
| 2 | MALPY01819 | 4914 | - | 319587 | 324591 |  |  |  |  |  | 7 |  |  |  |  |  |  |  |  |
| 2 | Pc.c000703271.Contig1 | 1384 | - | 320497 | 321962 |  |  |  |  |  |  |  |  |  |  |  |  |  |  |
| JC2 | MALPY03165 | 141 | - | 320778 | 320919 |  |  |  |  |  |  | 235 | 150 |  |  |  |  |  |  |
| 2 | MALPY03433 | 129 | + | 320915 | 321044 |  |  |  |  |  |  | 235 |  |  |  |  |  |  |  |
| 2 | Pc.c000803240.Contig1 | 2657 | - | 322006 | 324663 |  |  |  |  |  |  |  |  |  |  |  |  |  |  |
| 2 | MALPY02865 | 2674 | - | 322856 | 327613 |  |  |  |  |  | 7 - Ins |  |  |  |  |  |  |  |  |
| 2 | Pb.c000701216.Contig3 | 3659 | - | 323607 | 327299 | cRMPc03017 | 12429 | 500 | 315184 | 327613 |  |  |  |  |  |  |  |  |  |
| 2 | MALPY01034 | 9662 | - | 328113 | 338464 |  |  |  |  |  | 7 - Ins |  |  |  |  |  |  |  |  |
| 2 | PbContig145 | 6163 | + | 332276 | 339513 |  |  |  |  |  |  |  |  |  |  |  |  |  |  |
| 2 | MALPY03171 | 367 | - | 332939 | 333297 |  |  |  |  |  |  |  |  |  |  |  |  |  |  |
| 2 | Pc.c000603089.Contig1 | 4107 | + | 333559 | 338272 |  |  |  |  |  |  |  |  |  |  |  |  |  |  |
| 2 | MALPY03512 | 410 | - | 335805 | 336209 |  |  |  |  |  |  |  |  |  |  |  |  |  |  |
| 2 | Pc.c000603087.Contig1 | 1459 | + | 338334 | 339802 |  |  |  |  |  |  |  |  |  |  |  |  |  |  |
| 2 | MALPY01870 | 2138 | - | 338375 | 340522 | cRMPc03018 | 12409 | 4112 | 328113 | 340522 | 7 |  |  | cRMPs0302 | 82373 | 4112 | 258149 | 340522 |  |
| 2 | MALPY00964 | 5045 | + | 344634 | 349676 | cRMPc03019 | 5042 | 919 | 344634 | 349676 |  |  |  | cRMPs0303 | 5042 | 919 | 344634 | 349676 |  |
| 2 | Pb.c000201245.Contig1 | 7824 | + | 350595 | 358534 |  |  |  |  |  |  |  |  |  |  |  |  |  |  |
| 2 | PcContig666 | 4189 | - | 352601 | 356598 |  |  |  |  |  |  |  |  |  |  |  |  |  |  |
| 2 | MALPY02485 | 5048 | + | 354620 | 359917 |  |  |  |  |  |  | 230 | 500 |  |  |  |  |  |  |
| 2 | Pc.c000503161.Contig1 | 856 | - | 357385 | 358238 |  |  |  |  |  |  |  |  |  |  |  |  |  |  |
| 2 | Pc.c001003669.Contig1 | 10940 | - | 358232 | 370407 |  |  |  |  |  |  |  |  |  |  |  |  |  |  |
| 2 | MALPY00845 | 24199 | - | 360101 | 389331 |  |  |  |  |  | 445 | 230 |  |  |  |  |  |  |  |
| 2 | PbContig66 | 6629 | + | 362829 | 369878 |  |  |  |  |  |  |  |  |  |  |  |  |  |  |
| 2 | PbContig290 | 13418 | + | 374541 | 388477 |  |  |  |  |  |  |  |  |  |  |  |  |  |  |
| 2 | PcContig358 | 8200 | - | 375144 | 383878 | cRMPc03020 | 38736 | 247 | 350595 | 389331 |  |  |  |  |  |  |  |  |  |
| 2 | MALPY01176 | 26083 | - | 389578 | 417267 |  |  |  |  |  | 445 | 15 | 500 |  |  |  |  |  |  |
| 2 | PbContig379 | 5743 | - | 399387 | 405218 |  |  |  |  |  |  |  |  |  |  |  |  |  |  |
| 2 | Pb.c001702327.Contig1 | 2229 | - | 405231 | 407460 |  |  |  |  |  |  |  |  |  |  |  |  |  |  |
| 2 | Pb.c001802779.Contig1.0 | 1454 | - | 405990 | 407444 |  |  |  |  |  |  |  |  |  |  |  |  |  |  |
| 2 | PcContig108 | 2071 | + | 406064 | 408144 |  |  |  |  |  |  |  |  |  |  |  |  |  |  |
| man2 | Pb.c000601557.Contig1 - Ins | 16554 | + | 407879 | 425815 |  |  |  |  |  |  |  |  |  |  |  |  |  |  |
| 2 | PcContig53 | 3373 | - | 408609 | 412215 |  |  |  |  |  |  |  |  |  |  |  |  |  |  |
| 2 | Pc.c000803440.Contig1 | 2667 | - | 412417 | 415115 |  |  |  |  |  |  |  |  |  |  |  |  |  |  |
| 2 | PcContig466 | 3362 | - | 415761 | 419201 |  |  |  |  |  |  |  |  |  |  |  |  |  |  |
| 2 | MALPY02993 | 1554 | - | 417333 | 418887 |  |  |  |  |  |  | 15 | 1300 |  |  |  |  |  |  |
| 2 | MALPY00888 | 3218 | + | 420371 | 423574 |  |  |  |  |  | 65 | 15 |  |  |  |  |  |  |  |
| 2 | MALPY00942 | 6641 | + | 422976 | 429650 |  |  |  |  |  | 65 |  |  |  |  |  |  |  |  |
| 2 | Pb.c001202091.Contig1 | 5253 | + | 427949 | 433064 |  |  |  |  |  |  |  |  |  |  |  |  |  |  |
| 2 | MALPY02162 | 8218 | + | 429523 | 437615 |  |  |  |  |  | 65 |  |  |  |  |  |  |  |  |
| 2 | Pc.c000903413.Contig1 | 2549 | - | 431924 | 434467 | cRMPc03021 | 48037 | 651 | 389578 | 437615 |  |  |  |  |  |  |  |  |  |
| 2 | MALPY01643 | 4548 | - | 438266 | 443042 |  |  |  |  |  | 65 |  |  |  |  |  |  |  |  |
| 2 | Pb.c000401424.Contig1 | 2024 | + | 441225 | 443210 |  |  |  |  |  |  |  |  |  |  |  |  |  |  |
| man2 | PbContig5407 | 7068 | + | 441723 | 451672 |  |  |  |  |  |  |  |  |  |  |  |  |  | MALPY01865/MALPY01986 |
| 2 | Pc.c000503596.Contig1 | 1500 | - | 442235 | 443735 |  |  |  |  |  |  |  |  |  |  |  |  |  |  |
| TK2 | MALPY01865 | 3313 | - | 443704 | 446449 |  |  |  |  |  | 65 |  |  |  |  |  |  |  |  |
| 2 | Pb.c000401427.Contig1 | 9177 | + | 447306 | 459015 |  |  |  |  |  |  |  |  |  |  |  |  |  |  |
| man2 | MALPY01986 | 4045 | + | 449276 | 453759 |  |  |  |  |  | 1218 |  |  |  |  |  |  |  |  |
| 2 | Pc.c000202667.Contig1 | 4094 | + | 452754 | 456913 |  |  |  |  |  |  |  |  |  |  |  |  |  |  |
| man2 | MALPY00053 - Exp | 9582 | - | 455688 | 466195 |  |  |  |  |  | 1218 |  |  |  |  |  |  |  |  |
| 2 | Pc.c000503131.Contig1 | 1345 | + | 455990 | 457335 |  |  |  |  |  |  |  |  |  |  |  |  |  |  |
| 2 | Pc.c000502457.Contig1 | 1572 | + | 456842 | 458414 |  |  |  |  |  |  |  |  |  |  |  |  |  |  |
| man2 | Pc.c000602850.Contig1 - Exp | 4384 | + | 456930 | 462150 |  |  |  |  |  |  |  |  |  |  |  |  |  |  |
| 2 | Pc.c001003490.Contig1 | 2008 | + | 461768 | 463776 |  |  |  |  |  |  |  |  |  |  |  |  |  |  |
| 2 | Pc.c001605897.Contig1 | 1096 | - | 461851 | 462944 |  |  |  |  |  |  |  |  |  |  |  |  |  |  |
| 2 | Pb.c002112246.Contig1 | 966 | + | 462077 | 463037 |  |  |  |  |  |  |  |  |  |  |  |  |  |  |
| 2 | Pb.c000401178.Contig3 | 10127 | - | 462562 | 474613 |  |  |  |  |  |  |  |  |  |  |  |  |  |  |
| 2 | Pc.c000803413.Contig1 | 1852 | + | 462580 | 464462 |  |  |  |  |  |  |  |  |  |  |  |  |  |  |
| 2 | MALPY00296 | 13317 | + | 467769 | 481001 |  |  |  |  |  |  |  |  |  |  |  |  |  |  |
| 2 | Pc.c000602841.Contig1 | 6928 | - | 468478 | 475403 |  |  |  |  |  |  |  |  |  |  |  |  |  |  |
| 2 | Pb.c000301459.Contig1 | 1582 | - | 474761 | 476439 |  |  |  |  |  |  |  |  |  |  |  |  |  |  |
| 2 | PbContig85 | 6781 | + | 476612 | 483429 |  |  |  |  |  |  |  |  |  |  |  |  |  |  |
| 2 | Pc.c000302375.Contig3 | 13550 | - | 477792 | 493411 |  |  |  |  |  |  |  |  |  |  |  |  |  |  |
| 2 | MALPY00297 | 14918 | - | 483766 | 499179 |  |  |  |  |  |  | 81 | 10000 |  |  |  |  |  |  |
| 2 | Pb.c001602465.Contig1 | 2032 | + | 488772 | 491156 | cRMPc03022 | 60913 | 930 | 438266 | 499179 |  |  |  |  |  |  |  |  |  |
| 2 | MALPY00329 | 15781 | - | 500109 | 516758 |  |  |  |  |  |  | 81 | 800 |  |  |  |  |  |  |
| 2 | Pb.c000901781.Contig1 | 5127 | - | 501002 | 506195 |  |  |  |  |  |  |  |  |  |  |  |  |  |  |
| 2 | Pc.c001606028.Contig1 | 946 | + | 504308 | 505254 |  |  |  |  |  |  |  |  |  |  |  |  |  |  |
| 2 | PbContig269 | 7530 | - | 510461 | 518024 |  |  |  |  |  |  |  |  |  |  |  |  |  |  |
| 2 | Pc.c000503570.Contig1 | 1577 | - | 514178 | 515764 | cRMPc03023 | 17915 | 2099 | 500109 | 518024 |  |  |  |  |  |  |  |  |  |
| 2 | MALPY00624 | 3858 | + | 520123 | 523966 |  |  |  |  |  |  | 81 | 700 |  |  |  |  |  |  |
| 2 | Pb.c000601509.Contig1 | 6588 | + | 520619 | 527958 |  |  |  |  |  |  |  |  |  |  |  |  |  |  |
| 2 | Pc.c001812909.Contig1 | 505 | + | 523699 | 524276 |  |  |  |  |  |  |  |  |  |  |  |  |  |  |
| 2 | MALPY01428 | 7789 | - | 524353 | 532808 |  |  |  |  |  |  | 81 | 1200 |  |  |  |  |  |  |
| 2 | Pc.c001103901.Contig1 | 2072 | + | 525708 | 527780 |  |  |  |  |  |  |  |  |  |  |  |  |  |  |
| 2 | Pc.c001103905.Contig1 | 7534 | + | 528329 | 535851 |  |  |  |  |  |  |  |  |  |  |  |  |  |  |
| 2 | Pb.c001001361.Contig1 | 6500 | + | 530296 | 536785 |  |  |  |  |  |  |  |  |  |  |  |  |  |  |
| 2 | MALPY01972 | 4410 | - | 533604 | 537987 |  |  |  |  |  |  | 81 |  |  |  |  |  |  |  |
| 2 | MALPY03294 | 828 | + | 535688 | 536483 |  |  |  |  |  |  |  |  |  |  |  |  |  |  |
| 2 | Pc.c000703317.Contig1 | 3312 | - | 536175 | 539487 |  |  |  |  |  |  |  |  |  |  |  |  |  |  |
| man2 | PcContig5563 | 3393 | - | 536276 | 539669 |  |  |  |  |  |  |  |  |  |  |  |  |  | MALPY01972/PbContig4511 |
| man2 | PbContig4511 | 5997 | + | 539376 | 546275 |  |  |  |  |  |  |  |  |  |  |  |  |  | PcContig5563/MALPY00459 |
| 2 | Pb.c001602444.Contig1 | 5915 | - | 540229 | 546141 |  |  |  |  |  |  |  |  |  |  |  |  |  |  |
| 2 | MALPY00459 | 2926 | + | 541807 | 544686 |  |  |  |  |  |  | 31 | 450 |  |  |  |  |  |  |
| 2 | MALPY03004 | 619 | + | 544666 | 545285 |  |  |  |  |  |  | 31 | 2000 |  |  |  |  |  |  |
| 2 | PcContig261 | 1976 | - | 544693 | 546666 |  |  |  |  |  |  |  |  |  |  |  |  |  |  |
| 2 | MALPY02221 | 6933 | + | 546449 | 554796 |  |  |  |  |  | 451 | 31 |  |  |  |  |  |  |  |
| 2 | Pb.c000801702.Contig1 | 3444 | - | 546767 | 550448 |  |  |  |  |  |  |  |  |  |  |  |  |  |  |
| 2 | Pc.c000903453.Contig1 | 6096 | + | 547205 | 554700 |  |  |  |  |  |  |  |  |  |  |  |  |  |  |
| 2 | MALPY03357 | 408 | + | 547474 | 547882 |  |  |  |  |  |  | 110 | 800 |  |  |  |  |  |  |
| 2 | Pb.c001903688.Contig1 | 3458 | - | 552364 | 556185 |  |  |  |  |  |  |  |  |  |  |  |  |  |  |
| 2 | MALPY03326 | 1425 | + | 555334 | 556732 | cRMPc03024 | 36609 | 353 | 520123 | 556732 | 451 | 110 | 1200 |  |  |  |  |  |  |
| 2 | Pb.c000901474.Contig1 | 5620 | - | 557085 | 562641 |  |  |  |  |  |  |  |  |  |  |  |  |  |  |
| 2 | MALPY01449 | 5438 | + | 557352 | 562612 |  |  |  |  |  | 748 | 110 |  |  |  |  |  |  |  |
| 2 | Pb.c000601797.Contig1 | 629 | - | 561178 | 561795 | cRMPc03025 | 5556 | 157 | 557085 | 562641 |  |  |  |  |  |  |  |  |  |
| 2 | MALPY01122 | 9671 | - | 562798 | 572724 |  |  |  |  |  | 748 | 279 | 1500 |  |  |  |  |  |  |
| 2 | Pc.c001203992.Contig1 | 5118 | - | 564770 | 570795 |  |  |  |  |  |  |  |  |  |  |  |  |  |  |
| 2 | Pb.c001101816.Contig1 | 4830 | - | 565156 | 570817 | cRMPc03026 | 9926 | 789 | 562798 | 572724 |  |  |  |  |  |  |  |  |  |
| 2 | MALPY00328 | 6779 | + | 573513 | 581030 |  |  |  |  |  | 900 | 279 |  |  |  |  |  |  |  |
| 2 | Pb.c001201956.Contig1 | 6052 | + | 574770 | 581342 |  |  |  |  |  |  |  |  |  |  |  |  |  |  |
| 2 | PcContig593 | 2972 | - | 577269 | 580932 | cRMPc03027 | 7829 | 1824 | 573513 | 581342 |  |  |  |  |  |  |  |  |  |
| 2 | MALPY01463 | 15808 | - | 583166 | 601347 |  |  |  |  |  | 900 |  |  |  |  |  |  |  |  |
| 2 | Pc.c000702677.Contig1 | 5401 | - | 583738 | 589139 |  |  |  |  |  |  |  |  |  |  |  |  |  |  |
| 2 | Pb.c001904278.Contig1 | 7565 | + | 586590 | 594428 |  |  |  |  |  |  |  |  |  |  |  |  |  |  |
| 2 | Pc.c000602918.Contig1 | 1005 | - | 591081 | 592095 |  |  |  |  |  |  |  |  |  |  |  |  |  |  |
| 2 | MALPY03215 | 469 | - | 591305 | 591773 |  |  |  |  |  |  |  |  |  |  |  |  |  |  |
| 2 | Pb.c000101278.Contig1 | 4617 | + | 596453 | 601029 |  |  |  |  |  |  |  |  |  |  |  |  |  |  |
| 2 | PcContig489 | 6160 | + | 597658 | 604779 |  |  |  |  |  |  |  |  |  |  |  |  |  |  |
| 2 | Pb.c001802860.Contig1 | 3670 | + | 601895 | 605758 |  |  |  |  |  |  |  |  |  |  |  |  |  |  |
| 2 | MALPY01374 | 11715 | + | 602352 | 614450 |  |  |  |  |  | 900 |  |  |  |  |  |  |  |  |
| 2 | Pb.c001904381.Contig1 | 1538 | + | 606999 | 608570 |  |  |  |  |  |  |  |  |  |  |  |  |  |  |
| 2 | Pc.c000502974.Contig1 | 1110 | + | 607899 | 609021 | cRMPc03028 | 31284 |  | 583166 | 614450 |  |  |  | cRMPs0304 | 263855 |  | 350595 | 614450 |  |
| Telomere | |  |  |  |  |  |  |  |  |  |  |  |  |  |  |  |  |  |  |
|  |  | Pb Pc Py contig |  |  |  |  | cRMP contig | Gap to next cRMP contig |  |  |  |  |  |  | cRMP scaffold | Gap to next cRMP scaffold |  |  |  |
|  | Number | 232 |  |  |  | Number | 28 | 27 |  |  |  |  |  | Number | 4 | 3 |  |  |  |
|  | Average size (bp) | 4528 |  |  |  | Average size (bp) | 20139 | 1872 |  |  |  |  |  | Average size (bp) | 151919 | 2258 |  |  |  |
|  | Minimum size (bp) | 129 |  |  |  | Minimum size (bp) | 1151 | 69 |  |  |  |  |  | Minimum size (bp) | 5042 | 919 |  |  |  |
|  | Maximum size (bp) | 26083 |  |  |  | Maximum size (bp) | 60913 | 8904 |  |  |  |  |  | Maximum size (bp) | 263855 | 4112 |  |  |  |
|  | Total size (bp) | 1050383 |  |  |  | Total size (bp) | 563893 | 50557 |  |  |  |  |  | Total size (bp) | 607677 | 6773 |  |  |  |
|  |  |  |  |  |  |  |  |  |  |  |  |  |  |  |  |  |  |  |  |
