# Supplementary material for: A Plasmodium Whole-Genome Synteny Map: Indels and Synteny Breakpoints as Foci for Species-Specific Genes
Source: PLoS Pathog. 2005 Dec 23;1(4):e44. doi: 10.1371/journal.ppat.0010044 (PMC1317653; doi:10.1371/journal.ppat.0010044)
Supplement: Supplementary file 4 [file ppat.0010044.st020.doc]

Table S20 - RMPchr4


# Table S20 - RMPchr4

|  |  |  |  |  |  |  |  |  |  |  |  |  |  |  |  |  |  |  |  |
| --- | --- | --- | --- | --- | --- | --- | --- | --- | --- | --- | --- | --- | --- | --- | --- | --- | --- | --- | --- |
| Table S20: Tiling path of RMP contigs combined to form cRMP chromosome 4. | | | | | | |  |  |  |  |  |  |  |  |  |  |  |  |  |
|  |  |  |  |  |  |  |  |  |  |  |  |  |  |  |  |  |  |  |  |
| Tiling paths of P. berghei (Pb), P. c. chabaudi (Pc) and P. y. yoelii (Py) contigs combined into composite rodent malaria parasite (cRMP) | | | | | | | | | |  |  |  |  |  |  |  |  |  |  |
| contigs aligned against parts of the 14 chromosomes of P. falciparum (Pf). The cRMP contigs are further linked by physical linkage | | | | | | | | | |  |  |  |  |  |  |  |  |  |  |
| and PCR (see Methods) to form cRMP scaffolds. Different synteny blocks with homology to different P. falciparum chromosomes | | | | | | | | | |  |  |  |  |  |  |  |  |  |  |
| together form the cRMP chromosomes. The tables also show gaps in the alignment (black lines), cRMP telomeres (white bars) and | | | | | | | | | |  |  |  |  |  |  |  |  |  |  |
| predicted centromeres (plum/black bars). Contigs, physical groups or PCR groups interrupted by inter- or intrasyntenic regions are | | | | | | | | | |  |  |  |  |  |  |  |  |  |  |
| printed in bold. A summary of the number of RMP contigs, cRMP contigs and cRMP scaffolds as well as their average, minimum, | | | | | | | | | |  |  |  |  |  |  |  |  |  |  |
| maximum and total sizes is provided. Column headings are as follows: | | | | |  |  |  |  |  |  |  |  |  |  |  |  |  |  |  |
|  |  |  |  |  |  |  |  |  |  |  |  |  |  |  |  |  |  |  |  |
| Pf chr.: P. falciparum chromosome to which the RMP contigs are aligned | | | | | |  |  |  |  |  |  |  |  |  |  |  |  |  |  |
| (#: PROmer analysis� from this study; JC# and TK#: Carlton et al.; man#: manual BLAST analysis; | | | | | | |  |  |  |  |  |  |  |  |  |  |  |  |  |
| i: inversed orientation of the synteny block; colours refer to those used in Fig. 2 of the paper); | | | | | | |  |  |  |  |  |  |  |  |  |  |  |  |  |
| Pb Pc Py contig: names of the aligned RMP contigs; | | |  |  |  |  |  |  |  |  |  |  |  |  |  |  |  |  |  |
| Size: size (bp) of the aligned RMP contigs; | |  |  |  |  |  |  |  |  |  |  |  |  |  |  |  |  |  |  |
| +/-: orientation of the aligned RMP contigs; | |  |  |  |  |  |  |  |  |  |  |  |  |  |  |  |  |  |  |
| Start contig & End contig: estimated start & end coordinates of alignment of the RMP contigs based on the coordinates | | | | | | | | |  |  |  |  |  |  |  |  |  |  |  |
| of PROmer hits corrected for the contig�s size and orientation and presence of inter- and intrasyntenic regions | | | | | | | |  |  |  |  |  |  |  |  |  |  |  |  |
| (the start position of the first RMP contig in the alignment was set at zero); | | | | | |  |  |  |  |  |  |  |  |  |  |  |  |  |  |
| cRMP contig: names of cRMP contigs | |  |  |  |  |  |  |  |  |  |  |  |  |  |  |  |  |  |  |
| (the first 2 digits refer to the cRMP chromosome number and the following 3 digits designate the order of the contigs); | | | | | | | | |  |  |  |  |  |  |  |  |  |  |  |
| cRMP contig size: size of cRMP contigs based on the corrected coordinates of alignment; | | | | | | |  |  |  |  |  |  |  |  |  |  |  |  |  |
| Gap size to next cRMP contig: size of gaps between neighbouring cRMP contigs; | | | | | | |  |  |  |  |  |  |  |  |  |  |  |  |  |
| Start cRMP contig & End cRMP contig: estimated start & end coordinates of alignment of the cRMP contigs; | | | | | | | |  |  |  |  |  |  |  |  |  |  |  |  |
| Phys. group (Py): physical linkage of neighbouring P. y. yoelii contigs; | | | | | |  |  |  |  |  |  |  |  |  |  |  |  |  |  |
| PCR group (Py): neighbouring P. y. yoelii contigs were linked previously or for this study by PCR; | | | | | | |  |  |  |  |  |  |  |  |  |  |  |  |  |
| PCR size (Py): size of the PCR product; | |  |  |  |  |  |  |  |  |  |  |  |  |  |  |  |  |  |  |
| cRMP scaffold: names of cRMP scaffolds | |  |  |  |  |  |  |  |  |  |  |  |  |  |  |  |  |  |  |
| (the first 2 digits refer to the cRMP chromosome number and the following 2 digits designate the order of the scaffolds); | | | | | | | | |  |  |  |  |  |  |  |  |  |  |  |
| cRMP scaffold size: size of cRMP scaffolds; | | |  |  |  |  |  |  |  |  |  |  |  |  |  |  |  |  |  |
| Gap size to next cRMP scaffold: size of gaps between neighbouring cRMP scaffolds; | | | | | | |  |  |  |  |  |  |  |  |  |  |  |  |  |
| Start cRMP scaffold & End cRMP scaffold: estimated start & end coordinates of alignment of the cRMP scaffolds; | | | | | | | | |  |  |  |  |  |  |  |  |  |  |  |
| Overlap: Linkage between RMP contigs established through manual BLAST analysis. | | | | | | |  |  |  |  |  |  |  |  |  |  |  |  |  |
|  |  |  |  |  |  |  |  |  |  |  |  |  |  |  |  |  |  |  |  |
| Pf chr. | Pb Pc Py contig | Size | +/- | Start contig | End contig | cRMP contig | cRMP contig size | Gap size to next cRMP contig | Start cRMP contig | End cRMP contig | Phys. group (Py) | PCR group (Py) | PCR size (Py) | cRMP scaffold | cRMP scaffold size | Gap size to next cRMP scaffold | Start cRMP scaffold | End cRMP scaffold | Overlap (manual) |
| Telomere | |  |  |  |  |  |  |  |  |  |  |  |  |  |  |  |  |  |  |
| 3 | Pb.c002006981.Contig1 | 829 | - | 0 | 829 |  |  |  |  |  |  |  |  |  |  |  |  |  |  |
| 3 | MALPY02702 | 3435 | + | 142 | 3532 |  |  |  |  |  |  | 108 | 3000 |  |  |  |  |  |  |
| 3 | PcContig691 | 2028 | + | 473 | 2478 |  |  |  |  |  |  |  |  |  |  |  |  |  |  |
| 3 | Pb.c002006982.Contig1 | 3224 | - | 762 | 3932 | cRMPc04001 | 3932 | 138 | 0 | 3932 |  |  |  |  |  |  |  |  |  |
| man3 | MALPY02247 | 2766 | - | 4070 | 6836 |  |  |  |  |  | 682 |  |  |  |  |  |  |  | Pb.c000101320.Contig1 |
| 3 | Pb.c000101320.Contig1 | 5173 | + | 6678 | 11857 |  |  |  |  |  |  |  |  |  |  |  |  |  |  |
| 3 | MALPY02246 | 6364 | - | 6828 | 13155 |  |  |  |  |  | 682 | 108 - Ins | 250 |  |  |  |  |  |  |
| 3 | Pc.c001707790.Contig1 | 601 | + | 7114 | 7727 |  |  |  |  |  |  |  |  |  |  |  |  |  |  |
| 3 | Pc.c000703421.Contig1 | 2521 | - | 7360 | 9881 | cRMPc04002 | 9085 | 350 | 4070 | 13155 |  |  |  |  |  |  |  |  |  |
| 3 | MALPY03151 | 1907 | - | 13505 | 15421 | cRMPc04003 | 1916 | 168 | 13505 | 15421 |  | 108 - Ins | 250 |  |  |  |  |  |  |
| 3 | MALPY01188 | 9484 | + | 15589 | 23951 |  |  |  |  |  |  | 108 | 1500 |  |  |  |  |  |  |
| 3 | Pb.c001802868.Contig1 | 3933 | + | 17387 | 20853 |  |  |  |  |  |  |  |  |  |  |  |  |  |  |
| 3 | Pc.c000603004.Contig1 | 3278 | + | 20280 | 23525 |  |  |  |  |  |  |  |  |  |  |  |  |  |  |
| 3 | Pb.c001502049.Contig2 | 7245 | + | 20662 | 26904 |  |  |  |  |  |  |  |  |  |  |  |  |  |  |
| 3 | MALPY00432 | 3133 | + | 23590 | 26745 |  |  |  |  |  | 21A | 108 | 400 |  |  |  |  |  |  |
| 3 | Pc.c000202906.Contig1 | 8655 | + | 25622 | 35081 |  |  |  |  |  |  |  |  |  |  |  |  |  |  |
| 3 | MALPY01813 | 7548 | + | 26933 | 34890 |  |  |  |  |  | 21A | 108 | 6500 |  |  |  |  |  |  |
| 3 | Pb.c002007892.Contig1 | 1408 | + | 28208 | 29616 |  |  |  |  |  |  |  |  |  |  |  |  |  |  |
| 3 | Pb.c001402117.Contig1 | 4885 | + | 30333 | 35236 | cRMPc04004 | 19647 | 16 | 15589 | 35236 |  |  |  |  |  |  |  |  |  |
| 3 | Pc.c000202178.Contig2 | 2249 | + | 35252 | 37501 | cRMPc04005 | 2249 | 277 | 35252 | 37501 |  |  |  |  |  |  |  |  |  |
| 3 | MALPY01598 | 4937 | - | 37778 | 40906 |  |  |  |  |  | 21A |  |  |  |  |  |  |  |  |
| 3 | Pc.c001707813.Contig1 | 3293 | + | 40315 | 43581 |  |  |  |  |  |  |  |  |  |  |  |  |  |  |
| 3 | MALPY00355 | 8485 | + | 40810 | 49313 |  |  |  |  |  | 696 | 108 |  |  |  |  |  |  |  |
| 3 | Pb.c001101917.Contig1 | 2195 | + | 41828 | 44017 |  |  |  |  |  |  |  |  |  |  |  |  |  |  |
| 3 | Pb.c001101919.Contig1 | 2922 | + | 44360 | 47076 |  |  |  |  |  |  |  |  |  |  |  |  |  |  |
| 3 | MALPY03565 | 173 | - | 44407 | 44583 |  |  |  |  |  |  | 324 | 3000 |  |  |  |  |  |  |
| 3 | Pc.c000402872.Contig1 | 1688 | + | 45066 | 46745 |  |  |  |  |  |  |  |  |  |  |  |  |  |  |
| 3 | PcContig559 | 2552 | + | 46947 | 49502 | cRMPc04006 | 11724 | 194 | 37778 | 49502 |  |  |  |  |  |  |  |  |  |
| 3 | MALPY02504 | 5620 | - | 49696 | 55139 |  |  |  |  |  | 696 | 324 | 2000 |  |  |  |  |  |  |
| 3 | Pc.c000903426.Contig1 | 3180 | - | 50190 | 53298 |  |  |  |  |  |  |  |  |  |  |  |  |  |  |
| 3 | Pb.c001702858.Contig1 | 2601 | - | 51368 | 53996 | cRMPc04007 | 5443 | 2377 | 49696 | 55139 |  |  |  |  |  |  |  |  |  |
| 3 | MALPY00141 | 15312 | + | 57516 | 74924 |  |  |  |  |  | 1146 | 324 | 650 |  |  |  |  |  |  |
| 3 | PbContig307 | 7678 | + | 58561 | 67044 |  |  |  |  |  |  |  |  |  |  |  |  |  |  |
| 3 | Pc.c001204461.Contig1 | 881 | - | 58910 | 59758 |  |  |  |  |  |  |  |  |  |  |  |  |  |  |
| 3 | Pc.c000203338.Contig1 | 5941 | - | 60385 | 66668 |  |  |  |  |  |  |  |  |  |  |  |  |  |  |
| 3 | Pb.c001702905.Contig1 | 5540 | + | 67772 | 74210 |  |  |  |  |  |  |  |  |  |  |  |  |  |  |
| 3 | Pc.c000903691.Contig1 | 3363 | - | 71999 | 75380 | cRMPc04008 | 17864 | 116 | 57516 | 75380 |  |  |  |  |  |  |  |  |  |
| 3 | MALPY01025 | 11810 | + | 75496 | 86821 |  |  |  |  |  | 1146 | 324 |  |  |  |  |  |  |  |
| 3 | PbContig84 | 6609 | - | 75873 | 82036 |  |  |  |  |  |  |  |  |  |  |  |  |  |  |
| 3 | Pc.c000903487.Contig1 | 3069 | - | 76516 | 79594 |  |  |  |  |  |  |  |  |  |  |  |  |  |  |
| 3 | Pc.c000302864.Contig1 | 1344 | - | 79192 | 80536 |  |  |  |  |  |  |  |  |  |  |  |  |  |  |
| 3 | MALPY03183 | 532 | - | 79499 | 80031 |  |  |  |  |  |  |  |  |  |  |  |  |  |  |
| 3 | Pc.c000403275.Contig1 | 811 | - | 80732 | 81543 |  |  |  |  |  |  |  |  |  |  |  |  |  |  |
| man3 | PbContig2 - Ins | 15723 | + | 83209 | 99788 |  |  |  |  |  |  |  |  |  |  |  |  |  |  |
| 3 | MALPY00900 | 3737 | - | 90085 | 93897 |  |  |  |  |  | 106 |  |  |  |  |  |  |  |  |
| 3 | PcContig443 | 2691 | - | 91466 | 94148 |  |  |  |  |  |  |  |  |  |  |  |  |  |  |
| man3 | MALPY02692 | 2363 | + | 94250 | 96613 |  |  |  |  |  | 106 |  |  |  |  |  |  |  | PbContig24 |
| TK3 | MALPY01163 | 2148 | - | 96694 | 98844 | cRMPc04009 | 24292 | 873 | 75496 | 99788 |  |  |  | cRMPs0401 | 99788 | 873 | 0 | 99788 |  |
| 3 | MALPY02340 | 2122 | - | 100661 | 110351 |  |  |  |  |  | 174 |  |  |  |  |  |  |  |  |
| 3 | Pc.c000002546.Contig1 | 3615 | + | 103457 | 107099 |  |  |  |  |  |  |  |  |  |  |  |  |  |  |
| 3 | MALPY02742 | 4286 | + | 103681 | 107994 |  |  |  |  |  | 174 |  |  |  |  |  |  |  |  |
| 3 | PbContig38 | 3238 | + | 104697 | 107961 |  |  |  |  |  |  |  |  |  |  |  |  |  |  |
| 3 | PcContig200 | 3495 | + | 107717 | 111227 |  |  |  |  |  |  |  |  |  |  |  |  |  |  |
| 3 | Pb.c001702919.Contig1 | 2851 | - | 108608 | 111492 |  |  |  |  |  |  |  |  |  |  |  |  |  |  |
| 3 | MALPY02448 | 2883 | + | 110645 | 113534 | cRMPc04010 | 12873 | 779 | 100661 | 113534 | 174 | 131 | 8000 |  |  |  |  |  |  |
| TK3 | MALPY02698 | 2214 | - | 114313 | 116146 |  |  |  |  |  | 174 |  |  |  |  |  |  |  |  |
| man3 | MALPY01414 | 3597 | - | 115785 | 119382 | cRMPc04011 | 5069 | 2134 | 114313 | 119382 | 174 |  |  |  |  |  |  |  | MALPY02698 |
| 3 | MALPY01578 | 6891 | - | 121516 | 128413 |  |  |  |  |  |  | 131 | 400 |  |  |  |  |  |  |
| 3 | Pb.c001001673.Contig1 | 3888 | - | 122043 | 125931 |  |  |  |  |  |  |  |  |  |  |  |  |  |  |
| 3 | Pc.c000903251.Contig1 | 4364 | + | 124552 | 128922 |  |  |  |  |  |  |  |  |  |  |  |  |  |  |
| 3 | Pb.c002112118.Contig1 | 1937 | + | 126214 | 128205 | cRMPc04012 | 7406 | 44 | 121516 | 128922 |  |  |  |  |  |  |  |  |  |
| 3 | MALPY01182 | 4773 | - | 128966 | 133457 |  |  |  |  |  |  | 131 | 2500 |  |  |  |  |  |  |
| 3 | PcContig242 | 1317 | + | 130178 | 131495 |  |  |  |  |  |  |  |  |  |  |  |  |  |  |
| 3 | Pb.c001101859.Contig1 | 3933 | + | 130589 | 134677 |  |  |  |  |  |  |  |  |  |  |  |  |  |  |
| 3 | Pc.c000803216.Contig1 | 2771 | + | 131562 | 134433 |  |  |  |  |  |  |  |  |  |  |  |  |  |  |
| 3 | PcContig15 | 2761 | + | 134522 | 137268 |  |  |  |  |  |  |  |  |  |  |  |  |  |  |
| 3 | MALPY03205 | 1604 | + | 135096 | 136659 |  |  |  |  |  | 166 | 131 |  |  |  |  |  |  |  |
| 3 | MALPY00730 | 10399 | - | 136833 | 148826 |  |  |  |  |  | 166 |  |  |  |  |  |  |  |  |
| 3 | Pc.c001203969.Contig1 | 2310 | + | 138176 | 140423 |  |  |  |  |  |  |  |  |  |  |  |  |  |  |
| 3 | PcContig716 | 2645 | + | 141151 | 144546 |  |  |  |  |  |  |  |  |  |  |  |  |  |  |
| 3 | PcContig669 | 13239 | + | 145568 | 160491 |  |  |  |  |  |  |  |  |  |  |  |  |  |  |
| 3 | PbContig122 | 5225 | + | 145708 | 151300 |  |  |  |  |  |  |  |  |  |  |  |  |  |  |
| 3 | MALPY03184 | 619 | - | 147653 | 148260 |  |  |  |  |  |  | 221 | 1500 |  |  |  |  |  |  |
| 3 | MALPY01936 | 7639 | + | 149697 | 158590 |  |  |  |  |  | 166 | 221 | 6500 |  |  |  |  |  |  |
| 3 | PbContig132 | 11583 | - | 153858 | 166775 |  |  |  |  |  |  |  |  |  |  |  |  |  |  |
| TK3 | MALPY02725 | 2978 | - | 158645 | 161332 |  |  |  |  |  | 166 |  |  |  |  |  |  |  |  |
| 3 | MALPY01678 | 7591 | - | 163668 | 172049 |  |  |  |  |  | 71 | 221 | 225 |  |  |  |  |  |  |
| 3 | Pc.c000002496.Contig1 | 15177 | - | 164346 | 180278 |  |  |  |  |  |  |  |  |  |  |  |  |  |  |
| 3 | PbContig232 | 6031 | + | 168531 | 174167 |  |  |  |  |  |  |  |  |  |  |  |  |  |  |
| 3 | MALPY00314 | 3165 | + | 171870 | 174616 |  |  |  |  |  | 71 | 221 | 800 |  |  |  |  |  |  |
| 3 | MALPY02390 | 6368 | - | 174789 | 181157 |  |  |  |  |  | 203 | 221 |  |  |  |  |  |  |  |
| 3 | Pb.c000401506.Contig1 | 5503 | + | 175447 | 180952 |  |  |  |  |  |  |  |  |  |  |  |  |  |  |
| 3 | PcContig20 | 11187 | - | 180685 | 192750 |  |  |  |  |  |  |  |  |  |  |  |  |  |  |
| 3 | MALPY01873 | 5535 | + | 181013 | 186896 |  |  |  |  |  | 203 |  |  |  |  |  |  |  |  |
| 3 | PbContig83 | 5480 | + | 181458 | 187504 |  |  |  |  |  |  |  |  |  |  |  |  |  |  |
| 3 | MALPY03501 | 395 | + | 182349 | 182744 |  |  |  |  |  |  | 159 | 6500 |  |  |  |  |  |  |
| 3 | MALPY02276 | 4503 | - | 188276 | 193041 |  |  |  |  |  |  | 159 | 3000 |  |  |  |  |  |  |
| 3 | Pb.c000601611.Contig1 | 4274 | + | 188709 | 193219 | cRMPc04013 | 64253 | 61 | 128966 | 193219 |  |  |  |  |  |  |  |  |  |
| TK3 | MALPY02323 | 2639 | - | 193280 | 195584 | cRMPc04014 | 2304 | 823 | 193280 | 195584 | 228 |  |  |  |  |  |  |  |  |
| 3 | MALPY01758 | 10035 | + | 196407 | 207537 |  |  |  |  |  | 228 | 159 | 700 |  |  |  |  |  |  |
| 3 | Pc.c000502960.Contig1 | 5299 | - | 198091 | 203335 |  |  |  |  |  |  |  |  |  |  |  |  |  |  |
| 3 | Pb.c001402175.Contig1 | 3992 | + | 198656 | 202653 |  |  |  |  |  |  |  |  |  |  |  |  |  |  |
| 3 | Pb.c001001802.Contig1 | 7150 | + | 203902 | 211381 |  |  |  |  |  |  |  |  |  |  |  |  |  |  |
| 3 | PcContig650 | 2132 | - | 207694 | 209793 |  |  |  |  |  |  |  |  |  |  |  |  |  |  |
| 3 | MALPY00529 | 28652 | - | 208024 | 237299 |  |  |  |  |  |  | 159 |  |  |  |  |  |  |  |
| 3 | Pc.c000703866.Contig1 | 805 | - | 209949 | 210754 |  |  |  |  |  |  |  |  |  |  |  |  |  |  |
| 3 | Pb.c000701430.Contig1 | 6745 | + | 215828 | 222535 |  |  |  |  |  |  |  |  |  |  |  |  |  |  |
| 3 | Pc.c001103051.Contig1 | 4917 | - | 216047 | 220979 |  |  |  |  |  |  |  |  |  |  |  |  |  |  |
| 3 | Pb.c001803552.Contig1 | 3050 | + | 223624 | 226677 |  |  |  |  |  |  |  |  |  |  |  |  |  |  |
| 3 | MALPY03186 | 283 | - | 225002 | 225292 |  |  |  |  |  |  |  |  |  |  |  |  |  |  |
| 3 | Pb.c001502023.Contig2 | 18649 | + | 227317 | 247606 |  |  |  |  |  |  |  |  |  |  |  |  |  |  |
| 3 | PcContig597 | 9087 | + | 231124 | 240820 |  |  |  |  |  |  |  |  |  |  |  |  |  |  |
| 3 | MALPY00642 | 16088 | - | 238619 | 255180 |  |  |  |  |  | 289 |  |  |  |  |  |  |  |  |
| 3 | Pc.c000202768.Contig1 | 3266 | + | 241981 | 245346 |  |  |  |  |  |  |  |  |  |  |  |  |  |  |
| 3 | Pc.c000502385.Contig3 | 6139 | + | 245879 | 251869 |  |  |  |  |  |  |  |  |  |  |  |  |  |  |
| 3 | Pb.c000601239.Contig1 | 6865 | + | 247516 | 254370 |  |  |  |  |  |  |  |  |  |  |  |  |  |  |
| 3 | PcContig27 | 1421 | + | 252115 | 253578 |  |  |  |  |  |  |  |  |  |  |  |  |  |  |
| 3 | Pb.c001101505.Contig1 | 3713 | + | 254660 | 258377 |  |  |  |  |  |  |  |  |  |  |  |  |  |  |
| 3 | MALPY00996 | 6219 | + | 255367 | 261729 |  |  |  |  |  | 289 |  |  |  |  |  |  |  |  |
| 3 | Pc.c001606566.Contig1 | 721 | - | 255931 | 256652 |  |  |  |  |  |  |  |  |  |  |  |  |  |  |
| 3 | Pc.c000803285.Contig1 | 1411 | - | 256837 | 258248 |  |  |  |  |  |  |  |  |  |  |  |  |  |  |
| 3 | PbContig353 | 12653 | + | 259054 | 272703 |  |  |  |  |  |  |  |  |  |  |  |  |  |  |
| 3 | Pc.c000603005.Contig1 | 3543 | - | 260496 | 264027 |  |  |  |  |  |  |  |  |  |  |  |  |  |  |
| 3 | MALPY01185 | 11076 | - | 260715 | 272350 |  |  |  |  |  | 289 |  |  |  |  |  |  |  |  |
| 3 | MALPY03549 | 819 | - | 261084 | 261900 |  |  |  |  |  |  |  |  |  |  |  |  |  |  |
| 3 | Pc.c000102617.Contig1 | 4451 | + | 272313 | 276816 |  |  |  |  |  |  |  |  |  |  |  |  |  |  |
| 3 | MALPY00816 | 8272 | - | 272607 | 281500 |  |  |  |  |  | 289 |  |  |  |  |  |  |  |  |
| 3 | Pb.c001903694.Contig1 | 2559 | + | 273214 | 275719 |  |  |  |  |  |  |  |  |  |  |  |  |  |  |
| 3 | Pb.c000101083.Contig2 | 4993 | - | 274657 | 280327 |  |  |  |  |  |  |  |  |  |  |  |  |  |  |
| 3 | Pc.c000102490.Contig1 | 2510 | + | 277623 | 280306 | cRMPc04015 | 85093 | 122 | 196407 | 281500 |  |  |  |  |  |  |  |  |  |
| 3 | Pb.c001702753.Contig1 | 3778 | + | 281622 | 285276 |  |  |  |  |  |  |  |  |  |  |  |  |  |  |
| 3 | MALPY02655 | 3203 | - | 281894 | 284877 |  |  |  |  |  | 289 | 252 | 9000 |  |  |  |  |  |  |
| 3 | Pc.c000502397.Contig1 | 4016 | + | 283263 | 287278 |  |  |  |  |  |  |  |  |  |  |  |  |  |  |
| TK3 | MALPY01615 | 4886 | - | 284963 | 289897 | cRMPc04016 | 8275 | 2155 | 281622 | 289897 | 289 |  |  |  |  |  |  |  |  |
| 3 | MALPY02163 | 3896 | - | 292052 | 296049 |  |  |  |  |  |  | 252 |  |  |  |  |  |  |  |
| 3 | Pc.c001405242.Contig1 | 725 | + | 293076 | 293801 |  |  |  |  |  |  |  |  |  |  |  |  |  |  |
| 3 | Pb.c001502566.Contig1 | 2760 | + | 293218 | 296116 |  |  |  |  |  |  |  |  |  |  |  |  |  |  |
| 3 | Pc.c001405147.Contig1 | 749 | + | 294489 | 295329 | cRMPc04017 | 4064 | 3756 | 292052 | 296116 |  |  |  | cRMPs0402 | 195455 | 3756 | 100661 | 296116 |  |
| 3 | MALPY02047 | 6027 | + | 299872 | 305920 | cRMPc04018 | 6048 | 6950 | 299872 | 305920 |  |  |  | cRMPs0403 | 6048 | 6950 | 299872 | 305920 |  |
| 3 | Pb.c001201936.Contig1 | 7299 | + | 312870 | 320166 |  |  |  |  |  |  |  |  |  |  |  |  |  |  |
| 3 | MALPY02704 | 5862 | - | 313582 | 319670 |  |  |  |  |  |  |  |  |  |  |  |  |  |  |
| man3 | PcContig63 - Ins | 7817 | + | 314866 | 324234 |  |  |  |  |  |  |  |  |  |  |  |  |  |  |
| 3 | MALPY01409 | 3714 | + | 322838 | 326769 |  |  |  |  |  |  | 42 | 1500 |  |  |  |  |  |  |
| 3 | Pc.c001606000.Contig1 | 1597 | + | 324035 | 326342 |  |  |  |  |  |  |  |  |  |  |  |  |  |  |
| 3 | Pb.c001803533.Contig1 | 1633 | - | 325844 | 327480 | cRMPc04019 | 14610 | 574 | 312870 | 327480 |  |  |  |  |  |  |  |  |  |
| 3 | MALPY01682 | 7925 | + | 328054 | 336063 |  |  |  |  |  | 46A | 42 | 3000 |  |  |  |  |  |  |
| 3 | PbContig89 | 15500 | + | 330183 | 348145 |  |  |  |  |  |  |  |  |  |  |  |  |  |  |
| TK3 | MALPY02786 | 3448 | + | 339393 | 340614 |  |  |  |  |  | 46A |  |  |  |  |  |  |  |  |
| 3 | MALPY02124 | 5269 | - | 341030 | 346254 |  |  |  |  |  | 953 | 42 | 3000 |  |  |  |  |  |  |
| 3 | Pc.c001707862.Contig1 | 1746 | - | 341469 | 343215 |  |  |  |  |  |  |  |  |  |  |  |  |  |  |
| 3 | Pc.c001003771.Contig1 | 1135 | + | 344531 | 345669 |  |  |  |  |  |  |  |  |  |  |  |  |  |  |
| man3 | MALPY01917 | 2829 | - | 346123 | 349452 |  |  |  |  |  | 953 |  |  |  |  |  |  |  | Pc.c001003771.Contig1/Pb.c001402479.Contig1 |
| man3 | Pb.c001402479.Contig1 | 890 | - | 348717 | 350107 |  |  |  |  |  |  |  |  |  |  |  |  |  | MALPY01917/MALPY00685 |
| 3 | MALPY00685 | 11762 | + | 349672 | 360113 |  |  |  |  |  |  | 42 | 2000 |  |  |  |  |  |  |
| 3 | Pc.c000302907.Contig1 | 2641 | + | 350231 | 352872 |  |  |  |  |  |  |  |  |  |  |  |  |  |  |
| 3 | Pb.c001301852.Contig1 | 2775 | - | 350579 | 353354 |  |  |  |  |  |  |  |  |  |  |  |  |  |  |
| 3 | Pc.c000102568.Contig1 | 5882 | + | 356502 | 362393 |  |  |  |  |  |  |  |  |  |  |  |  |  |  |
| 3 | MALPY00463 | 21463 | - | 361516 | 382825 |  |  |  |  |  | 731 | 42 |  |  |  |  |  |  |  |
| 3 | Pb.c000201558.Contig1 | 4176 | - | 364078 | 368245 |  |  |  |  |  |  |  |  |  |  |  |  |  |  |
| 3 | Pc.c000602331.Contig3 | 3938 | + | 366778 | 370729 |  |  |  |  |  |  |  |  |  |  |  |  |  |  |
| 3 | PbContig276 | 3937 | - | 368787 | 372594 |  |  |  |  |  |  |  |  |  |  |  |  |  |  |
| 3 | Pc.c000602877.Contig1 | 3111 | + | 370400 | 373688 |  |  |  |  |  |  |  |  |  |  |  |  |  |  |
| 3 | MALPY03003 | 542 | + | 371751 | 372299 |  |  |  |  |  |  |  |  |  |  |  |  |  |  |
| 3 | Pb.c000201303.Contig2 | 3775 | - | 372816 | 376513 |  |  |  |  |  |  |  |  |  |  |  |  |  |  |
| 3 | Pc.c000703315.Contig1 | 2576 | + | 373936 | 376504 |  |  |  |  |  |  |  |  |  |  |  |  |  |  |
| 3 | PcContig36 | 7918 | + | 377278 | 386803 |  |  |  |  |  |  |  |  |  |  |  |  |  |  |
| 3 | Pb.c001803326.Contig1 | 1713 | - | 377326 | 379036 |  |  |  |  |  |  |  |  |  |  |  |  |  |  |
| 3 | Pb.c000601172.Contig1 | 9093 | - | 382129 | 391504 |  |  |  |  |  |  |  |  |  |  |  |  |  |  |
| 3 | MALPY00464 | 6589 | + | 384287 | 390963 |  |  |  |  |  | 731 | 130 | 900 |  |  |  |  |  |  |
| 3 | Pc.c001505046.Contig1 | 1494 | + | 390849 | 392352 |  |  |  |  |  |  |  |  |  |  |  |  |  |  |
| 3 | MALPY03204 | 598 | + | 390980 | 391590 |  |  |  |  |  |  | 130 | 800 |  |  |  |  |  |  |
| 3 | Pb.c000301156.Contig1 | 14496 | - | 391271 | 407506 |  |  |  |  |  |  |  |  |  |  |  |  |  |  |
| 3 | MALPY01380 | 12017 | + | 391468 | 405075 |  |  |  |  |  | 133 | 130 |  |  |  |  |  |  |  |
| 3 | PcContig54 | 8015 | + | 391869 | 401600 |  |  |  |  |  |  |  |  |  |  |  |  |  |  |
| 3 | PcContig383 | 2102 | - | 401532 | 403634 |  |  |  |  |  |  |  |  |  |  |  |  |  |  |
| 3 | Pc.c001103704.Contig1 | 2616 | - | 404552 | 407190 |  |  |  |  |  |  |  |  |  |  |  |  |  |  |
| 3 | MALPY01077 | 11862 | + | 404975 | 417625 |  |  |  |  |  | 133 |  |  |  |  |  |  |  |  |
| 3 | PcContig14 | 2790 | + | 407494 | 410242 |  |  |  |  |  |  |  |  |  |  |  |  |  |  |
| 3 | PbContig210 | 10014 | - | 410642 | 421199 |  |  |  |  |  |  |  |  |  |  |  |  |  |  |
| 3 | Pc.c001505437.Contig1 | 1182 | - | 412922 | 414101 |  |  |  |  |  |  |  |  |  |  |  |  |  |  |
| 3 | Pc.c000102581.Contig1 | 6022 | - | 414448 | 420865 |  |  |  |  |  |  |  |  |  |  |  |  |  |  |
| 3 | MALPY00881 | 7365 | - | 417946 | 425237 | cRMPc04020 | 97183 | 1715 | 328054 | 425237 | 133 |  |  | cRMPs0404 | 112367 | 1715 | 312870 | 425237 |  |
| TK3 | MALPY01842 | 3069 | - | 426952 | 430042 |  |  |  |  |  | 291 |  |  |  |  |  |  |  |  |
| 3 | Pb.c000901796.Contig1 | 5195 | + | 427574 | 432775 |  |  |  |  |  |  |  |  |  |  |  |  |  |  |
| 3 | MALPY00798 | 4348 | + | 429673 | 434197 |  |  |  |  |  | 291 |  |  |  |  |  |  |  |  |
| 3 | Pc.c001707716.Contig1 | 1028 | - | 429768 | 430813 | cRMPc04021 | 7245 | 4821 | 426952 | 434197 |  |  |  | cRMPs0405 | 7245 | 4821 | 426952 | 434197 |  |
| 3 | MALPY00628 | 14102 | + | 439018 | 453376 |  |  |  |  |  |  |  |  |  |  |  |  |  |  |
| 3 | Pb.c002007177.Contig1 | 2981 | - | 440436 | 443416 |  | 14358 | 13390 | 439018 | 453376 |  |  |  | cRMPs0406 | 14358 | 13390 | 439018 | 453376 |  |
| Centromere - gap 13390 bp | |  |  | 460000 |  |  |  |  |  |  |  |  |  |  |  |  |  |  |  |
| 3 | MALPY02636 | 3741 | + | 466766 | 470780 | cRMPc04022 | 4014 | 1656 | 466766 | 470780 |  |  |  | cRMPs0407 | 4014 | 1656 | 466766 | 470780 |  |
| 3 | MALPY01507 | 17475 | - | 472436 | 488701 |  |  |  |  |  |  | 179 | 2500 |  |  |  |  |  |  |
| 3 | PbContig179 | 2551 | - | 473886 | 476437 |  |  |  |  |  |  |  |  |  |  |  |  |  |  |
| 3 | Pb.c000701574.Contig1 | 8357 | - | 478412 | 487326 |  |  |  |  |  |  |  |  |  |  |  |  |  |  |
| 3 | Pc.c000202834.Contig1 | 2494 | + | 479243 | 481109 |  |  |  |  |  |  |  |  |  |  |  |  |  |  |
| 3 | Pc.c000602981.Contig1 | 2448 | + | 481953 | 484620 |  |  |  |  |  |  |  |  |  |  |  |  |  |  |
| 3 | Pc.c000003077.Contig1 | 2281 | - | 485028 | 487309 | cRMPc04023 | 16265 | 1136 | 472436 | 488701 |  |  |  |  |  |  |  |  |  |
| 3 | MALPY01913 | 8178 | - | 489837 | 498018 |  |  |  |  |  |  | 179 | 2000 |  |  |  |  |  |  |
| 3 | Pb.c000701242.Contig2 | 16188 | + | 494668 | 509327 |  |  |  |  |  |  |  |  |  |  |  |  |  |  |
| 3 | PcContig583 | 4836 | - | 496881 | 501714 |  |  |  |  |  |  |  |  |  |  |  |  |  |  |
| 3 | MALPY01960 | 7890 | - | 497095 | 504542 |  |  |  |  |  |  | 179 | 1200 |  |  |  |  |  |  |
| 3 | MALPY00997 | 9351 | - | 506890 | 516241 |  |  |  |  |  | 359 | 179 | 4000 |  |  |  |  |  |  |
| 3 | PcContig628 | 4607 | - | 507765 | 512740 |  |  |  |  |  |  |  |  |  |  |  |  |  |  |
| 3 | Pb.c001201650.Contig2 | 5417 | + | 510603 | 516020 | cRMPc04024 | 26404 | 1178 | 489837 | 516241 |  |  |  |  |  |  |  |  |  |
| TK3 | MALPY01866 | 4123 | + | 517419 | 521270 |  |  |  |  |  | 359 |  |  |  |  |  |  |  |  |
| 3 | Pb.c000201625.Contig1 | 3789 | - | 520832 | 525080 |  |  |  |  |  |  |  |  |  |  |  |  |  |  |
| 3 | MALPY03232 | 1384 | + | 521976 | 523532 |  |  |  |  |  | 825 | 179 |  |  |  |  |  |  |  |
| 3 | Pc.c001203218.Contig1 | 3230 | + | 522250 | 526072 |  |  |  |  |  |  |  |  |  |  |  |  |  |  |
| 3 | MALPY01044 | 5169 | + | 522554 | 528179 |  |  |  |  |  | 825 |  |  |  |  |  |  |  |  |
| 3 | Pb.c000801836.Contig1 | 7865 | + | 525820 | 534076 |  |  |  |  |  |  |  |  |  |  |  |  |  |  |
| 3 | MALPY02160 | 6227 | + | 528871 | 535488 |  |  |  |  |  |  | 132 | 1800 |  |  |  |  |  |  |
| 3 | PcContig758 | 2392 | + | 529864 | 532522 | cRMPc04025 | 18069 | 636 | 517419 | 535488 |  |  |  |  |  |  |  |  |  |
| 3 | Pc.c001811919.Contig1 | 2900 | - | 536124 | 539012 |  |  |  |  |  |  |  |  |  |  |  |  |  |  |
| 3 | PbContig340 | 5686 | + | 536287 | 541907 |  |  |  |  |  |  |  |  |  |  |  |  |  |  |
| 3 | MALPY03062 | 956 | + | 536989 | 537945 |  |  |  |  |  |  | 132 |  |  |  |  |  |  |  |
| 3 | MALPY01546 - BP | 2512 | - | 538912 | 541567 | cRMPc04026 | 5783 | 2763 | 536124 | 541907 |  | new | 4500 |  |  |  |  |  |  |
| i9 | Pc.c001811890.Contig1 | 2884 | + | 544670 | 547531 |  |  |  |  |  |  |  |  |  |  |  |  |  |  |
| i9 | MALPY00994 - BP | 4233 | - | 546067 | 551152 |  |  |  |  |  |  | new |  |  |  |  |  |  |  |
| i9 | Pb.c001702849.Contig1 | 4062 | + | 549088 | 553168 |  |  |  |  |  |  |  |  |  |  |  |  |  |  |
| i9 | MALPY01573 | 7469 | - | 549493 | 559018 |  |  |  |  |  |  | 263 | 2500 |  |  |  |  |  |  |
| i9 | MALPY03153 | 532 | + | 550011 | 550549 |  |  |  |  |  |  | 322 | 2800 |  |  |  |  |  |  |
| i9 | Pc.c000903471.Contig1 | 1258 | + | 555096 | 556475 |  |  |  |  |  |  |  |  |  |  |  |  |  |  |
| i9 | Pb.c001803325.Contig1 | 3056 | + | 555440 | 558546 |  |  |  |  |  |  |  |  |  |  |  |  |  |  |
| i9 | MALPY02563 | 3913 | - | 557111 | 561042 | cRMPc04027 | 16372 | 780 | 544670 | 561042 |  | 322 |  |  |  |  |  |  |  |
| i9 | MALPY02811 | 2595 | + | 561822 | 564417 |  |  |  |  |  | 924 | 263 |  |  |  |  |  |  |  |
| i9 | PcContig460 | 8102 | - | 562415 | 570475 |  |  |  |  |  |  |  |  |  |  |  |  |  |  |
| i9 | Pb.c001202190.Contig1 | 2783 | - | 563265 | 566051 |  |  |  |  |  |  |  |  |  |  |  |  |  |  |
| i9 | MALPY01551 | 10045 | + | 565128 | 575176 |  |  |  |  |  | 924 | 323 | 2200 |  |  |  |  |  |  |
| i9 | MALPY01980 | 3202 | - | 571879 | 575144 |  |  |  |  |  | 502 | 323 |  |  |  |  |  |  |  |
| i9 | Pc.c001405204.Contig1 | 793 | - | 572186 | 572979 |  |  |  |  |  |  |  |  |  |  |  |  |  |  |
| i9 | Pc.c001204554.Contig1 | 1269 | - | 573072 | 574338 | cRMPc04028 | 13354 | 48 | 561822 | 575176 |  |  |  |  |  |  |  |  |  |
| iJC9 | MALPY01119 | 5179 | - | 575224 | 580415 |  |  |  |  |  | 502 |  |  |  |  |  |  |  |  |
| i9 | PcContig513 | 6801 | + | 578605 | 585457 |  |  |  |  |  |  |  |  |  |  |  |  |  |  |
| i9 | MALPY01404 | 7336 | - | 581495 | 588900 |  |  |  |  |  | 502 |  |  |  |  |  |  |  |  |
| i9 | Pb.c000401569.Contig1 | 4410 | - | 581676 | 586119 |  |  |  |  |  |  |  |  |  |  |  |  |  |  |
| i9 | Pc.c001708515.Contig1 | 833 | - | 585748 | 586542 |  |  |  |  |  |  |  |  |  |  |  |  |  |  |
| i9 | Pb.c001101955.Contig1 | 1836 | - | 588171 | 590010 |  |  |  |  |  |  |  |  |  |  |  |  |  |  |
| i9 | Pc.c001707707.Contig1 | 2099 | - | 589533 | 591638 |  |  |  |  |  |  |  |  |  |  |  |  |  |  |
| i9 | MALPY03000 | 1928 | - | 590253 | 592187 | cRMPc04029 | 16963 | 1326 | 575224 | 592187 |  | 26 | 1600 |  |  |  |  |  |  |
| i9 | MALPY02131 | 4870 | + | 593513 | 598359 |  |  |  |  |  | 173 | 26 |  |  |  |  |  |  |  |
| i9 | Pc.c001811928.Contig1 | 4809 | - | 594616 | 599401 |  |  |  |  |  |  |  |  |  |  |  |  |  |  |
| i9 | PbContig280 | 5144 | - | 596978 | 601832 |  |  |  |  |  |  |  |  |  |  |  |  |  |  |
| i9 | MALPY00486 | 4150 | + | 597088 | 600916 |  |  |  |  |  | 173 | 292 | 500 |  |  |  |  |  |  |
| i9 | Pc.c000202672.Contig1 | 1599 | - | 600447 | 602151 |  |  |  |  |  |  |  |  |  |  |  |  |  |  |
| i9 | MALPY03506 | 713 | - | 600970 | 601752 |  |  |  |  |  |  | 292 |  |  |  |  |  |  |  |
| i9 | MALPY03027 | 1704 | - | 601733 | 603622 |  |  |  |  |  |  |  |  |  |  |  |  |  |  |
| i9 | Pb.c001001713.Contig1 | 11398 | - | 603456 | 615049 |  |  |  |  |  |  |  |  |  |  |  |  |  |  |
| i9 | PcContig213 | 3813 | - | 604021 | 608141 |  |  |  |  |  |  |  |  |  |  |  |  |  |  |
| i9 | MALPY03559 | 840 | + | 604331 | 605171 |  |  |  |  |  |  |  |  |  |  |  |  |  |  |
| i9 | MALPY01396 | 18934 | + | 605186 | 625011 |  |  |  |  |  |  |  |  |  |  |  |  |  |  |
| i9 | Pc.c000502910.Contig1 | 710 | + | 608026 | 608736 |  |  |  |  |  |  |  |  |  |  |  |  |  |  |
| i9 | Pc.c000603489.Contig1 | 979 | + | 608834 | 609774 |  |  |  |  |  |  |  |  |  |  |  |  |  |  |
| i9 | Pc.c000202705.Contig1 | 3613 | - | 612010 | 615716 |  |  |  |  |  |  |  |  |  |  |  |  |  |  |
| i9 | MALPY03139 | 210 | + | 614594 | 614804 |  |  |  |  |  |  |  |  |  |  |  |  |  |  |
| i9 | Pb.c001001712.Contig1 | 3493 | - | 615312 | 618808 |  |  |  |  |  |  |  |  |  |  |  |  |  |  |
| i9 | Pc.c001606501.Contig1 | 632 | - | 616279 | 616869 |  |  |  |  |  |  |  |  |  |  |  |  |  |  |
| i9 | PcContig653 | 2170 | - | 616928 | 619098 |  |  |  |  |  |  |  |  |  |  |  |  |  |  |
| i9 | Pb.c000901735.Contig1 | 9680 | + | 619243 | 629248 |  |  |  |  |  |  |  |  |  |  |  |  |  |  |
| i9 | Pc.c001812185.Contig1 | 1007 | - | 619693 | 620700 |  |  |  |  |  |  |  |  |  |  |  |  |  |  |
| i9 | Pc.c000803277.Contig1 | 2428 | - | 621377 | 624111 |  |  |  |  |  |  |  |  |  |  |  |  |  |  |
| i9 | MALPY00016 | 20208 | + | 625199 | 647293 |  |  |  |  |  |  | 63 | 4000 |  |  |  |  |  |  |
| i9 | MALPY03408 | 821 | + | 628425 | 629258 |  |  |  |  |  |  |  |  |  |  |  |  |  |  |
| i9 | PbContig147 | 34246 | + | 630490 | 667880 |  |  |  |  |  |  |  |  |  |  |  |  |  |  |
| i9 | Pc.c000202208.Contig2 | 2093 | - | 636643 | 638721 |  |  |  |  |  |  |  |  |  |  |  |  |  |  |
| i9 | PcContig30 | 6428 | - | 641594 | 647916 |  |  |  |  |  |  |  |  |  |  |  |  |  |  |
| i9 | MALPY03480 | 337 | + | 645967 | 646300 |  |  |  |  |  |  |  |  |  |  |  |  |  |  |
| i9 | MALPY01419 | 4913 | + | 650299 | 655233 |  |  |  |  |  |  | 63 | 2000 |  |  |  |  |  |  |
| i9 | Pc.c001605847.Contig1 | 1078 | + | 651664 | 652751 |  |  |  |  |  |  |  |  |  |  |  |  |  |  |
| i9 | Pc.c001605827.Contig1 | 1758 | + | 653757 | 655629 |  |  |  |  |  |  |  |  |  |  |  |  |  |  |
| i9 | MALPY02181 | 2648 | + | 657297 | 659945 |  |  |  |  |  |  | 63 | 450 |  |  |  |  |  |  |
| i9 | Pc.c000403370.Contig1 | 6411 | + | 658331 | 664701 |  |  |  |  |  |  |  |  |  |  |  |  |  |  |
| i9 | MALPY01493 | 5880 | - | 659916 | 665796 |  |  |  |  |  | 439 | 63 |  |  |  |  |  |  |  |
| i9 | MALPY00304 | 38444 | + | 665498 | 707570 |  |  |  |  |  | 439 | 276 | 850 |  |  |  |  |  |  |
| i9 | Pc.c000003029.Contig1 | 1184 | + | 667273 | 668457 |  |  |  |  |  |  |  |  |  |  |  |  |  |  |
| i9 | Pb.c000901412.Contig1 | 7319 | - | 668165 | 675121 |  |  |  |  |  |  |  |  |  |  |  |  |  |  |
| i9 | Pc.c001505658.Contig1 | 853 | + | 673841 | 674694 |  |  |  |  |  |  |  |  |  |  |  |  |  |  |
| i9 | Pc.c001606354.Contig1 | 779 | + | 674792 | 675550 |  |  |  |  |  |  |  |  |  |  |  |  |  |  |
| i9 | Pb.c002007063.Contig1 | 2370 | - | 675467 | 677822 |  |  |  |  |  |  |  |  |  |  |  |  |  |  |
| i9 | Pb.c001001812.Contig1 | 3898 | + | 680123 | 684367 |  |  |  |  |  |  |  |  |  |  |  |  |  |  |
| i9 | PcContig683 | 3647 | + | 680768 | 684676 |  |  |  |  |  |  |  |  |  |  |  |  |  |  |
| i9 | Pb.c001101971.Contig1 | 6313 | + | 684972 | 691378 |  |  |  |  |  |  |  |  |  |  |  |  |  |  |
| i9 | Pc.c001303501.Contig1 | 3708 | + | 685775 | 689573 |  |  |  |  |  |  |  |  |  |  |  |  |  |  |
| i9 | Pb.c001702368.Contig1 | 3924 | + | 691911 | 695686 |  |  |  |  |  |  |  |  |  |  |  |  |  |  |
| i9 | Pc.c000502944.Contig1 | 1099 | + | 692770 | 693722 |  |  |  |  |  |  |  |  |  |  |  |  |  |  |
| i9 | Pb.c000701446.Contig1 | 8868 | + | 698215 | 707543 |  |  |  |  |  |  |  |  |  |  |  |  |  |  |
| i9 | Pc.c000303447.Contig1 | 3669 | + | 698372 | 702007 |  |  |  |  |  |  |  |  |  |  |  |  |  |  |
| i9 | PcContig676 | 1031 | + | 702438 | 703469 |  |  |  |  |  |  |  |  |  |  |  |  |  |  |
| i9 | Pc.c000802696.Contig2 | 2507 | + | 703796 | 706576 | cRMPc04030 | 114057 | 862 | 593513 | 707570 |  |  |  |  |  |  |  |  |  |
| i9 | MALPY00294 | 17252 | + | 708432 | 726258 |  |  |  |  |  |  | 276 | 2000 |  |  |  |  |  |  |
| i9 | Pb.c002007820.Contig1 | 1319 | - | 709272 | 710627 |  |  |  |  |  |  |  |  |  |  |  |  |  |  |
| i9 | Pb.c001502609.Contig1 | 2418 | - | 711089 | 713639 |  |  |  |  |  |  |  |  |  |  |  |  |  |  |
| i9 | PbContig33 | 6957 | + | 713660 | 720979 |  |  |  |  |  |  |  |  |  |  |  |  |  |  |
| i9 | Pc.c001003250.Contig1 | 4393 | - | 717698 | 722263 |  |  |  |  |  |  |  |  |  |  |  |  |  |  |
| i9 | Pb.c001402042.Contig1 | 2520 | - | 720522 | 723045 |  |  |  |  |  |  |  |  |  |  |  |  |  |  |
| i9 | Pb.c001101540.Contig2 | 5198 | - | 721000 | 726171 | cRMPc04031 | 17826 | 3648 | 708432 | 726258 |  |  |  |  |  |  |  |  |  |
| i9 | MALPY00495 | 12235 | - | 729906 | 742785 |  |  |  |  |  |  | 276 |  |  |  |  |  |  |  |
| i9 | Pc.c000602392.Contig2 | 3089 | - | 733384 | 736486 |  |  |  |  |  |  |  |  |  |  |  |  |  |  |
| i9 | MALPY02206 | 2515 | + | 737599 | 740102 |  |  |  |  |  |  |  |  |  |  |  |  |  |  |
| i9 | Pb.c001904538.Contig1 | 1869 | + | 737835 | 739704 |  |  |  |  |  |  |  |  |  |  |  |  |  |  |
| i9 | Pb.c001905057.Contig1 | 716 | + | 739635 | 740336 |  |  |  |  |  |  |  |  |  |  |  |  |  |  |
| iman9 | MALPY02400 | 3353 | + | 742251 | 745604 |  |  |  |  |  |  |  |  |  |  |  |  |  | \| |
| iman9 | PbContig3018 | 2609 | + | 742379 | 744988 |  |  |  |  |  |  |  |  |  |  |  |  |  | MALPY00495 |
| iman9 | PcContig6973 | 6448 | + | 742557 | 749005 |  |  |  |  |  |  |  |  |  |  |  |  |  | \| |
| iman9 | PbContig4273 | 3102 | + | 748599 | 751701 | cRMPc04032 | 21795 |  | 729906 | 751701 |  |  |  | cRMPs0408 | 279265 |  | 472436 | 751701 | \| |
| Telomere | |  |  |  |  |  |  |  |  |  |  |  |  |  |  |  |  |  |  |
|  |  | Pb Pc Py contig |  |  |  |  | cRMP contig | Gap to next cRMP contig |  |  |  |  |  |  | cRMP scaffold | Gap to next cRMP scaffold |  |  |  |
|  | Number | 289 |  |  |  | Number | 33 | 32 |  |  |  |  |  | Number | 8 | 7 |  |  |  |
|  | Average size (bp) | 4983 |  |  |  | Average size (bp) | 21086 | 1746 |  |  |  |  |  | Average size (bp) | 89818 | 4737 |  |  |  |
|  | Minimum size (bp) | 173 |  |  |  | Minimum size (bp) | 1916 | 16 |  |  |  |  |  | Minimum size (bp) | 4014 | 873 |  |  |  |
|  | Maximum size (bp) | 38444 |  |  |  | Maximum size (bp) | 114057 | 13390 |  |  |  |  |  | Maximum size (bp) | 279265 | 13390 |  |  |  |
|  | Total size (bp) | 1440231 |  |  |  | Total size (bp) | 695835 | 55866 |  |  |  |  |  | Total size (bp) | 718540 | 33161 |  |  |  |
|  |  |  |  |  |  |  |  |  |  |  |  |  |  |  |  |  |  |  |  |
