# Supplementary material for: A Plasmodium Whole-Genome Synteny Map: Indels and Synteny Breakpoints as Foci for Species-Specific Genes
Source: PLoS Pathog. 2005 Dec 23;1(4):e44. doi: 10.1371/journal.ppat.0010044 (PMC1317653; doi:10.1371/journal.ppat.0010044)
Supplement: Supplementary file 5 [file ppat.0010044.st021.doc]

Table S21 - RMPchr5


# Table S21 - RMPchr5

|  |  |  |  |  |  |  |  |  |  |  |  |  |  |  |  |  |  |  |  |
| --- | --- | --- | --- | --- | --- | --- | --- | --- | --- | --- | --- | --- | --- | --- | --- | --- | --- | --- | --- |
| Table S21: Tiling path of RMP contigs combined to form cRMP chromosome 5. | | | | | | |  |  |  |  |  |  |  |  |  |  |  |  |  |
|  |  |  |  |  |  |  |  |  |  |  |  |  |  |  |  |  |  |  |  |
| Tiling paths of P. berghei (Pb), P. c. chabaudi (Pc) and P. y. yoelii (Py) contigs combined into composite rodent malaria parasite (cRMP) | | | | | | | | | |  |  |  |  |  |  |  |  |  |  |
| contigs aligned against parts of the 14 chromosomes of P. falciparum (Pf). The cRMP contigs are further linked by physical linkage | | | | | | | | | |  |  |  |  |  |  |  |  |  |  |
| and PCR (see Methods) to form cRMP scaffolds. Different synteny blocks with homology to different P. falciparum chromosomes | | | | | | | | | |  |  |  |  |  |  |  |  |  |  |
| together form the cRMP chromosomes. The tables also show gaps in the alignment (black lines), cRMP telomeres (white bars) and | | | | | | | | | |  |  |  |  |  |  |  |  |  |  |
| predicted centromeres (plum/black bars). Contigs, physical groups or PCR groups interrupted by inter- or intrasyntenic regions are | | | | | | | | | |  |  |  |  |  |  |  |  |  |  |
| printed in bold. A summary of the number of RMP contigs, cRMP contigs and cRMP scaffolds as well as their average, minimum, | | | | | | | | | |  |  |  |  |  |  |  |  |  |  |
| maximum and total sizes is provided. Column headings are as follows: | | | | |  |  |  |  |  |  |  |  |  |  |  |  |  |  |  |
|  |  |  |  |  |  |  |  |  |  |  |  |  |  |  |  |  |  |  |  |
| Pf chr.: P. falciparum chromosome to which the RMP contigs are aligned | | | | | |  |  |  |  |  |  |  |  |  |  |  |  |  |  |
| (#: PROmer analysis� from this study; JC# and TK#: Carlton et al.; man#: manual BLAST analysis; | | | | | | |  |  |  |  |  |  |  |  |  |  |  |  |  |
| i: inversed orientation of the synteny block; colours refer to those used in Fig. 2 of the paper); | | | | | | |  |  |  |  |  |  |  |  |  |  |  |  |  |
| Pb Pc Py contig: names of the aligned RMP contigs; | | |  |  |  |  |  |  |  |  |  |  |  |  |  |  |  |  |  |
| Size: size (bp) of the aligned RMP contigs; | |  |  |  |  |  |  |  |  |  |  |  |  |  |  |  |  |  |  |
| +/-: orientation of the aligned RMP contigs; | |  |  |  |  |  |  |  |  |  |  |  |  |  |  |  |  |  |  |
| Start contig & End contig: estimated start & end coordinates of alignment of the RMP contigs based on the coordinates | | | | | | | | |  |  |  |  |  |  |  |  |  |  |  |
| of PROmer hits corrected for the contig�s size and orientation and presence of inter- and intrasyntenic regions | | | | | | | |  |  |  |  |  |  |  |  |  |  |  |  |
| (the start position of the first RMP contig in the alignment was set at zero); | | | | | |  |  |  |  |  |  |  |  |  |  |  |  |  |  |
| cRMP contig: names of cRMP contigs | |  |  |  |  |  |  |  |  |  |  |  |  |  |  |  |  |  |  |
| (the first 2 digits refer to the cRMP chromosome number and the following 3 digits designate the order of the contigs); | | | | | | | | |  |  |  |  |  |  |  |  |  |  |  |
| cRMP contig size: size of cRMP contigs based on the corrected coordinates of alignment; | | | | | | |  |  |  |  |  |  |  |  |  |  |  |  |  |
| Gap size to next cRMP contig: size of gaps between neighbouring cRMP contigs; | | | | | | |  |  |  |  |  |  |  |  |  |  |  |  |  |
| Start cRMP contig & End cRMP contig: estimated start & end coordinates of alignment of the cRMP contigs; | | | | | | | |  |  |  |  |  |  |  |  |  |  |  |  |
| Phys. group (Py): physical linkage of neighbouring P. y. yoelii contigs; | | | | | |  |  |  |  |  |  |  |  |  |  |  |  |  |  |
| PCR group (Py): neighbouring P. y. yoelii contigs were linked previously or for this study by PCR; | | | | | | |  |  |  |  |  |  |  |  |  |  |  |  |  |
| PCR size (Py): size of the PCR product; | |  |  |  |  |  |  |  |  |  |  |  |  |  |  |  |  |  |  |
| cRMP scaffold: names of cRMP scaffolds | |  |  |  |  |  |  |  |  |  |  |  |  |  |  |  |  |  |  |
| (the first 2 digits refer to the cRMP chromosome number and the following 2 digits designate the order of the scaffolds); | | | | | | | | |  |  |  |  |  |  |  |  |  |  |  |
| cRMP scaffold size: size of cRMP scaffolds; | | |  |  |  |  |  |  |  |  |  |  |  |  |  |  |  |  |  |
| Gap size to next cRMP scaffold: size of gaps between neighbouring cRMP scaffolds; | | | | | | |  |  |  |  |  |  |  |  |  |  |  |  |  |
| Start cRMP scaffold & End cRMP scaffold: estimated start & end coordinates of alignment of the cRMP scaffolds; | | | | | | | | |  |  |  |  |  |  |  |  |  |  |  |
| Overlap: Linkage between RMP contigs established through manual BLAST analysis. | | | | | | |  |  |  |  |  |  |  |  |  |  |  |  |  |
|  |  |  |  |  |  |  |  |  |  |  |  |  |  |  |  |  |  |  |  |
| Pf chr. | Pb Pc Py contig | Size | +/- | Start contig | End contig | cRMP contig | cRMP contig size | Gap size to next cRMP contig | Start cRMP contig | End cRMP contig | Phys. group (Py) | PCR group (Py) | PCR size (Py) | cRMP scaffold | cRMP scaffold size | Gap size to next cRMP scaffold | Start cRMP scaffold | End cRMP scaffold | Overlap (manual) |
| Telomere | |  |  |  |  |  |  |  |  |  |  |  |  |  |  |  |  |  |  |
| 10 | MALPY00055 | 20460 | - | 0 | 20572 |  |  |  |  |  |  |  |  |  |  |  |  |  |  |
| 10 | PbContig146 | 6479 | + | 6555 | 13013 |  |  |  |  |  |  |  |  |  |  |  |  |  |  |
| 10 | Pb.c000101109.Contig1 | 616 | + | 9296 | 9912 |  |  |  |  |  |  |  |  |  |  |  |  |  |  |
| 10 | Pb.c002007587.Contig1 | 733 | + | 14293 | 15023 |  |  |  |  |  |  |  |  |  |  |  |  |  |  |
| 10 | PbContig96 | 7144 | - | 15340 | 22484 | cRMPc05001 | 22484 | 74 | 0 | 22484 |  |  |  | cRMPs0501 | 22484 | 74 | 0 | 22484 |  |
| 10 | MALPY00729 | 17715 | + | 22558 | 40886 |  |  |  |  |  | 307 |  |  |  |  |  |  |  |  |
| 10 | Pb.c000001811.Contig1 | 11282 | - | 24558 | 35780 |  |  |  |  |  |  |  |  |  |  |  |  |  |  |
| 10 | Pc.c000703888.Contig1 | 786 | - | 27458 | 28223 |  |  |  |  |  |  |  |  |  |  |  |  |  |  |
| 10 | Pc.c001304620.Contig1 | 3258 | - | 34632 | 37890 |  |  |  |  |  |  |  |  |  |  |  |  |  |  |
| 10 | PbContig77 | 9285 | - | 36441 | 46089 |  |  |  |  |  |  |  |  |  |  |  |  |  |  |
| 10 | MALPY03323 | 1555 | + | 37739 | 39306 |  |  |  |  |  |  |  |  |  |  |  |  |  |  |
| 10 | Pc.c001606757.Contig1 | 628 | - | 40361 | 40944 |  |  |  |  |  |  |  |  |  |  |  |  |  |  |
| 10 | MALPY00708 | 7366 | - | 40818 | 48414 |  |  |  |  |  | 307 | 188 | 3000 |  |  |  |  |  |  |
| 10 | Pc.c001003379.Contig1 | 3634 | - | 41137 | 44771 |  |  |  |  |  |  |  |  |  |  |  |  |  |  |
| 10 | MALPY03155 | 926 | - | 44913 | 45845 |  |  |  |  |  |  |  |  |  |  |  |  |  |  |
| 10 | Pc.c001003231.Contig1 | 1643 | + | 46103 | 47746 |  |  |  |  |  |  |  |  |  |  |  |  |  |  |
| 10 | Pc.c001003951.Contig1 | 2116 | + | 46519 | 48635 |  |  |  |  |  |  |  |  |  |  |  |  |  |  |
| man10 | MALPY01105 | 2808 | - | 48296 | 51104 |  |  |  |  |  | 754 |  |  |  |  |  |  |  | MALPY02456 |
| 10 | PbContig34 | 3719 | - | 48377 | 52342 |  |  |  |  |  |  |  |  |  |  |  |  |  |  |
| 10 | MALPY02456 | 3044 | + | 50608 | 53913 | cRMPc05002 | 31355 | 1698 | 22558 | 53913 | 754 | 188 | 3500 |  |  |  |  |  |  |
| 10 | MALPY01570 | 8746 | + | 55611 | 64899 |  |  |  |  |  |  | 188 | 2500 |  |  |  |  |  |  |
| 10 | PcContig284 | 5092 | + | 55615 | 60673 |  |  |  |  |  |  |  |  |  |  |  |  |  |  |
| 10 | Pb.c001803845.Contig1 | 2007 | + | 56214 | 58287 |  |  |  |  |  |  |  |  |  |  |  |  |  |  |
| 10 | Pb.c001803882.Contig1 | 1083 | + | 59309 | 60395 |  |  |  |  |  |  |  |  |  |  |  |  |  |  |
| 10 | Pc.c000603028.Contig1 | 2470 | - | 61261 | 64067 | cRMPc05003 | 9288 | 3473 | 55611 | 64899 |  |  |  |  |  |  |  |  |  |
| 10 | MALPY00686 | 7235 | + | 68372 | 75725 |  |  |  |  |  |  | 188 | 2500 |  |  |  |  |  |  |
| 10 | Pb.c001702896.Contig1 | 3069 | + | 72477 | 75658 |  |  |  |  |  |  |  |  |  |  |  |  |  |  |
| 10 | Pc.c000302846.Contig1 | 5949 | - | 74503 | 80713 |  |  |  |  |  |  |  |  |  |  |  |  |  |  |
| 10 | MALPY01214 | 23255 | + | 78608 | 105052 |  |  |  |  |  |  | 188 | 4000 |  |  |  |  |  |  |
| 10 | Pb.c002112116.Contig1 | 3442 | - | 79680 | 83124 |  |  |  |  |  |  |  |  |  |  |  |  |  |  |
| 10 | PcContig558 | 1784 | - | 80095 | 81885 |  |  |  |  |  |  |  |  |  |  |  |  |  |  |
| 10 | PcContig722 | 10310 | + | 84052 | 94412 |  |  |  |  |  |  |  |  |  |  |  |  |  |  |
| 10 | Pb.c000001800.Contig1 | 4261 | - | 84666 | 88900 |  |  |  |  |  |  |  |  |  |  |  |  |  |  |
| 10 | Pb.c001803551.Contig1 | 3269 | - | 98783 | 102109 |  |  |  |  |  |  |  |  |  |  |  |  |  |  |
| 10 | Pc.c001708576.Contig1 | 676 | + | 101329 | 101990 | cRMPc05004 | 36680 | 1013 | 68372 | 105052 |  |  |  |  |  |  |  |  |  |
| 10 | Pc.c000002073.Contig1 | 3682 | + | 106065 | 109862 |  |  |  |  |  |  |  |  |  |  |  |  |  |  |
| 10 | Pb.c000901422.Contig1 | 3287 | + | 106659 | 109980 |  |  |  |  |  |  |  |  |  |  |  |  |  |  |
| 10 | MALPY01645 | 10628 | + | 107066 | 117950 |  |  |  |  |  | 529 | 188 |  |  |  |  |  |  |  |
| 10 | Pc.c000102539.Contig1 | 2021 | + | 110206 | 112426 |  |  |  |  |  |  |  |  |  |  |  |  |  |  |
| 10 | Pb.c002006818.Contig1 | 2442 | - | 110910 | 113579 |  |  |  |  |  |  |  |  |  |  |  |  |  |  |
| 10 | Pc.c000702722.Contig2 | 3767 | + | 112646 | 116466 | cRMPc05005 | 11885 | 923 | 106065 | 117950 |  |  |  |  |  |  |  |  |  |
| 10 | MALPY00164 | 31262 | - | 118873 | 149984 |  |  |  |  |  | 529 |  |  |  |  |  |  |  |  |
| 10 | Pb.c000301159.Contig1 | 7497 | + | 123431 | 130925 |  |  |  |  |  |  |  |  |  |  |  |  |  |  |
| 10 | PcContig138 | 6352 | - | 124881 | 130687 |  |  |  |  |  |  |  |  |  |  |  |  |  |  |
| 10 | PbContig252 | 6953 | + | 134246 | 141250 |  |  |  |  |  |  |  |  |  |  |  |  |  |  |
| 10 | Pc.c001303504.Contig1 | 7685 | - | 134620 | 142348 |  |  |  |  |  |  |  |  |  |  |  |  |  |  |
| 10 | MALPY03366 | 1233 | - | 141466 | 142708 |  |  |  |  |  |  |  |  |  |  |  |  |  |  |
| 10 | Pb.c000901775.Contig1 | 5363 | + | 141864 | 148367 |  |  |  |  |  |  |  |  |  |  |  |  |  |  |
| 10 | MALPY03381 | 875 | - | 141955 | 142830 |  |  |  |  |  |  |  |  |  |  |  |  |  |  |
| 10 | PcContig531 | 3616 | + | 142354 | 146410 |  |  |  |  |  |  |  |  |  |  |  |  |  |  |
| 10 | Pc.c000402803.Contig1 | 3928 | - | 145807 | 149720 | cRMPc05006 | 31111 | 1453 | 118873 | 149984 |  |  |  |  |  |  |  |  |  |
| 10 | MALPY01406 | 15024 | - | 151437 | 167719 |  |  |  |  |  | 529 | 39 | 1700 |  |  |  |  |  |  |
| 10 | Pb.c001402167.Contig1 | 7041 | + | 156613 | 164540 |  |  |  |  |  |  |  |  |  |  |  |  |  |  |
| 10 | Pc.c001812577.Contig1 | 863 | - | 156899 | 157948 |  |  |  |  |  |  |  |  |  |  |  |  |  |  |
| 10 | MALPY03188 | 323 | - | 157515 | 157838 |  |  |  |  |  |  |  |  |  |  |  |  |  |  |
| 10 | MALPY03074 | 512 | + | 157536 | 158045 |  |  |  |  |  |  |  |  |  |  |  |  |  |  |
| 10 | Pc.c000703903.Contig1 | 1227 | + | 158798 | 160118 |  |  |  |  |  |  |  |  |  |  |  |  |  |  |
| 10 | PcContig418 | 3217 | + | 160758 | 163999 |  |  |  |  |  |  |  |  |  |  |  |  |  |  |
| 10 | PcContig632 | 1753 | + | 164247 | 166576 |  |  |  |  |  |  |  |  |  |  |  |  |  |  |
| 10 | Pb.c000001777.Contig1 | 2297 | + | 165179 | 167510 |  |  |  |  |  |  |  |  |  |  |  |  |  |  |
| 10 | MALPY03582 | 670 | - | 166649 | 167293 | cRMPc05007 | 16282 | 957 | 151437 | 167719 |  |  |  |  |  |  |  |  |  |
| 10 | Pc.c000903469.Contig1 | 3671 | - | 168676 | 172463 |  |  |  |  |  |  |  |  |  |  |  |  |  |  |
| 10 | MALPY00898 | 4868 | + | 168955 | 173922 |  |  |  |  |  | 123 | 39 |  |  |  |  |  |  |  |
| 10 | Pb.c001903655.Contig1 | 4380 | - | 169018 | 173520 |  |  |  |  |  |  |  |  |  |  |  |  |  |  |
| TK10 | MALPY02293 | 3830 | + | 173775 | 177870 |  |  |  |  |  | 123 |  |  |  |  |  |  |  |  |
| 10 | Pb.c001301857.Contig1 | 2194 | - | 177408 | 179569 |  |  |  |  |  |  |  |  |  |  |  |  |  |  |
| 10 | MALPY01548 | 5831 | + | 177449 | 183219 |  |  |  |  |  | 123 | 288 | 300 |  |  |  |  |  |  |
| 10 | Pc.c000803780.Contig1 | 2125 | - | 179039 | 181104 |  |  |  |  |  |  |  |  |  |  |  |  |  |  |
| 10 | Pb.c002006946.Contig1 | 1897 | + | 181555 | 183452 |  |  |  |  |  |  |  |  |  |  |  |  |  |  |
| 10 | MALPY00617 | 9402 | - | 182837 | 193318 |  |  |  |  |  | 29 | 288 |  |  |  |  |  |  |  |
| 10 | Pb.c000801366.Contig1 | 4870 | + | 183486 | 188536 |  |  |  |  |  |  |  |  |  |  |  |  |  |  |
| 10 | Pc.c000203262.Contig1 | 1165 | - | 185595 | 186760 |  |  |  |  |  |  |  |  |  |  |  |  |  |  |
| 10 | PcContig546 | 5187 | + | 186813 | 193297 |  |  |  |  |  |  |  |  |  |  |  |  |  |  |
| 10 | MALPY03242 | 840 | - | 189295 | 190171 |  |  |  |  |  |  |  |  |  |  |  |  |  |  |
| 10 | MALPY03415 | 732 | - | 189304 | 190078 |  |  |  |  |  |  |  |  |  |  |  |  |  |  |
| 10 | Pb.c001101546.Contig2 | 3992 | + | 189980 | 195542 |  |  |  |  |  |  |  |  |  |  |  |  |  |  |
| 10 | Pc.c001003435.Contig1 | 7997 | + | 192833 | 204381 |  |  |  |  |  |  |  |  |  |  |  |  |  |  |
| 10 | MALPY02766 | 2165 | - | 193570 | 195735 |  |  |  |  |  | 29 |  |  |  |  |  |  |  |  |
| 10 | MALPY01486 | 16922 | + | 196333 | 216144 |  |  |  |  |  | 29 | 178 | 1400 |  |  |  |  |  |  |
| 10 | Pc.c000102622.Contig1 | 2142 | + | 204955 | 207079 |  |  |  |  |  |  |  |  |  |  |  |  |  |  |
| 10 | Pb.c001402083.Contig1 | 2134 | + | 213895 | 216074 |  |  |  |  |  |  |  |  |  |  |  |  |  |  |
| 10 | Pc.c000703413.Contig1 | 1149 | - | 215224 | 216373 | cRMPc05008 | 47697 | 1193 | 168676 | 216373 |  |  |  |  |  |  |  |  |  |
| 10 | MALPY01229 | 6866 | - | 217566 | 224678 |  |  |  |  |  | 472 | 178 |  |  |  |  |  |  |  |
| 10 | Pb.c000701486.Contig1 | 3144 | + | 219790 | 222820 |  |  |  |  |  |  |  |  |  |  |  |  |  |  |
| 10 | PcContig247 | 4875 | + | 220888 | 226034 |  |  |  |  |  |  |  |  |  |  |  |  |  |  |
| 10 | MALPY00550 | 10481 | - | 225210 | 235682 |  |  |  |  |  | 472 |  |  |  |  |  |  |  |  |
| 10 | Pb.c000301422.Contig1 | 3907 | + | 227228 | 231091 |  |  |  |  |  |  |  |  |  |  |  |  |  |  |
| 10 | Pc.c001707718.Contig1 | 1140 | - | 227816 | 228962 |  |  |  |  |  |  |  |  |  |  |  |  |  |  |
| 10 | Pc.c001708141.Contig1 | 875 | + | 228977 | 229837 | cRMPc05009 | 18116 | 3608 | 217566 | 235682 |  |  |  |  |  |  |  |  |  |
| 10 | MALPY01490 | 5737 | + | 239290 | 245060 |  |  |  |  |  | 472 |  |  |  |  |  |  |  |  |
| 10 | Pb.c001702907.Contig1 | 4343 | - | 240410 | 244778 |  |  |  |  |  |  |  |  |  |  |  |  |  |  |
| 10 | Pc.c001707741.Contig1 | 1358 | + | 242808 | 244166 |  |  |  |  |  |  |  |  |  |  |  |  |  |  |
| 10 | MALPY01584 | 3879 | - | 244933 | 248837 |  |  |  |  |  | 472 | 275 | 2500 |  |  |  |  |  |  |
| 10 | Pb.c001904508.Contig1 | 3126 | + | 245271 | 248400 |  |  |  |  |  |  |  |  |  |  |  |  |  |  |
| 10 | PcContig700 | 1294 | + | 245853 | 247147 |  |  |  |  |  |  |  |  |  |  |  |  |  |  |
| 10 | Pb.c001904629.Contig1 | 893 | + | 248660 | 249552 |  |  |  |  |  |  |  |  |  |  |  |  |  |  |
| 10 | MALPY03405 | 695 | - | 248921 | 249616 | cRMPc05010 | 10326 | 2890 | 239290 | 249616 |  |  |  |  |  |  |  |  |  |
| 10 | MALPY02840 | 3259 | + | 252506 | 255911 |  |  |  |  |  |  | 275 | 3500 |  |  |  |  |  |  |
| man10 | Syntenic Centromere |  |  | 252506 | 283717 |  |  |  |  |  |  |  |  |  |  |  |  |  |  |
| 10 | MALPY00449 | 22413 | + | 259411 | 283717 |  |  |  |  |  | 80\_alt | 275 |  |  |  |  |  |  |  |
| 10 | Pb.c000301375.Contig1 | 2781 | - | 260256 | 263340 |  |  |  |  |  |  |  |  |  |  |  |  |  |  |
| 10 | Pc.c000602953.Contig1 | 2859 | + | 260337 | 263360 |  |  |  |  |  |  |  |  |  |  |  |  |  |  |
| 10 | Pb.c000301376.Contig1 | 4111 | - | 263608 | 267710 |  |  |  |  |  |  |  |  |  |  |  |  |  |  |
| 10 | Pb.c000601596.Contig1 | 1775 | - | 269522 | 271276 |  |  |  |  |  |  |  |  |  |  |  |  |  |  |
| 10 | Pb.c000901890.Contig1 | 3250 | - | 271412 | 274815 |  |  |  |  |  |  |  |  |  |  |  |  |  |  |
| 10 | Pc.c001304664.Contig1 | 1583 | - | 272090 | 273641 |  |  |  |  |  |  |  |  |  |  |  |  |  |  |
| 10 | PbContig243 | 20193 | - | 276804 | 298336 |  |  |  |  |  |  |  |  |  |  |  |  |  |  |
| 10 | Pc.c000602378.Contig3 | 3441 | + | 276895 | 280628 |  |  |  |  |  |  |  |  |  |  |  |  |  |  |
| 10 | MALPY01013 | 12651 | - | 283899 | 297186 |  |  |  |  |  | 80\_alt |  |  |  |  |  |  |  |  |
| 10 | Pc.c001505032.Contig1 | 2262 | + | 286164 | 288522 |  |  |  |  |  |  |  |  |  |  |  |  |  |  |
| 10 | Pc.c001104265.Contig1 | 844 | + | 289729 | 290621 |  |  |  |  |  |  |  |  |  |  |  |  |  |  |
| 10 | Pc.c000202244.Contig1 | 3306 | + | 291104 | 294830 |  |  |  |  |  |  |  |  |  |  |  |  |  |  |
| 10 | MALPY00404 | 12051 | + | 297310 | 309255 |  |  |  |  |  | 80\_alt |  |  |  |  |  |  |  |  |
| 10 | Pb.c000201243.Contig2 | 9041 | - | 298700 | 307647 |  |  |  |  |  |  |  |  |  |  |  |  |  |  |
| 10 | Pc.c001103090.Contig1 | 3569 | + | 305884 | 309756 |  |  |  |  |  |  |  |  |  |  |  |  |  |  |
| 10 | Pb.c001001792.Contig1 | 2720 | - | 307855 | 310903 |  |  |  |  |  |  |  |  |  |  |  |  |  |  |
| 10 | MALPY02198 | 2210 | - | 308514 | 310959 |  |  |  |  |  | 80\_alt |  |  |  |  |  |  |  |  |
| 10 | Pc.c000802658.Contig2 | 3215 | + | 309983 | 313204 |  |  |  |  |  |  |  |  |  |  |  |  |  |  |
| 10 | MALPY00631 | 15711 | + | 310666 | 327603 |  |  |  |  |  | 80\_alt | 294 | 5000 |  |  |  |  |  |  |
| 10 | PbContig20 | 13634 | - | 312047 | 325936 |  |  |  |  |  |  |  |  |  |  |  |  |  |  |
| 10 | Pc.c000703389.Contig1 | 4342 | + | 313319 | 317667 |  |  |  |  |  |  |  |  |  |  |  |  |  |  |
| 10 | Pc.c001003496.Contig1 | 1473 | - | 319792 | 321223 |  |  |  |  |  |  |  |  |  |  |  |  |  |  |
| 10 | Pc.c000102513.Contig1 | 2206 | + | 319995 | 322377 |  |  |  |  |  |  |  |  |  |  |  |  |  |  |
| 10 | Pc.c000101994.Contig1 | 5062 | + | 322186 | 327485 |  |  |  |  |  |  |  |  |  |  |  |  |  |  |
| 10 | MALPY03545 | 780 | + | 323944 | 324727 | cRMPc05011 | 75097 | 6050 | 252506 | 327603 |  |  |  |  |  |  |  |  |  |
| 10 | MALPY00391 | 9054 | - | 333653 | 342755 |  |  |  |  |  |  | 294 | 1300 |  |  |  |  |  |  |
| 10 | Pb.c002007567.Contig1 | 1285 | + | 336231 | 337516 |  |  |  |  |  |  |  |  |  |  |  |  |  |  |
| 10 | Pb.c000701579.Contig1 | 6709 | - | 337391 | 344113 |  |  |  |  |  |  |  |  |  |  |  |  |  |  |
| 10 | Pc.c000703394.Contig1 | 2843 | - | 341738 | 344682 |  |  |  |  |  |  |  |  |  |  |  |  |  |  |
| 10 | MALPY03539 | 816 | - | 342038 | 342848 |  |  |  |  |  |  |  |  |  |  |  |  |  |  |
| 10 | MALPY01563 | 8671 | + | 343107 | 352550 |  |  |  |  |  | 465 | 294 |  |  |  |  |  |  |  |
| 10 | Pc.c000903993.Contig1 | 1339 | + | 349465 | 350804 |  |  |  |  |  |  |  |  |  |  |  |  |  |  |
| 10 | Pc.c001103810.Contig1 | 1648 | + | 350990 | 352683 |  |  |  |  |  |  |  |  |  |  |  |  |  |  |
| 10 | Pb.c001702872.Contig1 | 1070 | + | 351265 | 352362 | cRMPc05012 | 19030 | 137 | 333653 | 352683 |  |  |  |  |  |  |  |  |  |
| 10 | PbContig225 | 15500 | + | 352820 | 368338 |  |  |  |  |  |  |  |  |  |  |  |  |  |  |
| 10 | MALPY01903 | 12834 | - | 355051 | 370834 |  |  |  |  |  | 465 | 336 | 1600 |  |  |  |  |  |  |
| 10 | Pc.c001003300.Contig1 | 2497 | + | 356116 | 358781 |  |  |  |  |  |  |  |  |  |  |  |  |  |  |
| 10 | Pb.c001201979.Contig1 | 1797 | + | 357976 | 359716 |  |  |  |  |  |  |  |  |  |  |  |  |  |  |
| 10 | PcContig299 | 7907 | + | 359312 | 367219 |  |  |  |  |  |  |  |  |  |  |  |  |  |  |
| 10 | Pc.c000402711.Contig1 | 4269 | + | 368794 | 373063 |  |  |  |  |  |  |  |  |  |  |  |  |  |  |
| 10 | Pb.c000901875.Contig1 | 2468 | - | 369112 | 371580 |  |  |  |  |  |  |  |  |  |  |  |  |  |  |
| 10 | MALPY00030 | 7036 | - | 372550 | 379788 |  |  |  |  |  | 117B | 336 |  |  |  |  |  |  |  |
| 10 | Pb.c001702879.Contig1 | 6448 | + | 373770 | 380464 |  |  |  |  |  |  |  |  |  |  |  |  |  |  |
| 10 | Pc.c001304922.Contig1 | 748 | - | 374006 | 374784 |  |  |  |  |  |  |  |  |  |  |  |  |  |  |
| 10 | Pc.c001505133.Contig1 | 1271 | - | 375268 | 376539 |  |  |  |  |  |  |  |  |  |  |  |  |  |  |
| 10 | MALPY03228 | 769 | - | 375565 | 376334 |  |  |  |  |  |  |  |  |  |  |  |  |  |  |
| TK10 | MALPY02506 | 7036 | - | 378195 | 381690 | cRMPc05013 | 28870 | 2866 | 352820 | 381690 | 117B |  |  | cRMPs0502 | 359132 | 2866 | 22558 | 381690 |  |
| man10 | MALPY02044 | 3138 | - | 384556 | 388148 |  |  |  |  |  |  |  |  |  |  |  |  |  |  |
| man10 | MALPY01733 | 2639 | - | 387624 | 390318 | cRMPc05014 | 5762 | 13157 | 384556 | 390318 |  |  |  |  |  |  |  |  |  |
| 10 | PcContig322 | 5345 | - | 403475 | 408829 |  |  |  |  |  |  |  |  |  |  |  |  |  |  |
| 10 | MALPY01891 | 4916 | + | 403487 | 408397 |  |  |  |  |  |  |  |  |  |  |  |  |  |  |
| 10 | Pb.c000301440.Contig1 | 1682 | + | 406078 | 407760 | cRMPc05015 | 5354 | 115 | 403475 | 408829 |  |  |  | cRMPs0503 | 24273 | 115 | 384556 | 408829 |  |
| 10 | MALPY01132 | 12865 | - | 408944 | 422508 |  |  |  |  |  | 450 |  |  |  |  |  |  |  |  |
| 10 | Pb.c001402460.Contig1 | 1298 | - | 409034 | 410380 |  |  |  |  |  |  |  |  |  |  |  |  |  |  |
| 10 | Pb.c001904483.Contig1 | 1306 | - | 410748 | 412054 |  |  |  |  |  |  |  |  |  |  |  |  |  |  |
| 10 | Pc.c001811706.Contig1 | 1813 | - | 411298 | 413081 | cRMPc05016 | 13564 | 710 | 408944 | 422508 |  |  |  |  |  |  |  |  |  |
| 10 | MALPY02322 | 3607 | + | 423218 | 426825 |  |  |  |  |  | 450 | 283 | 500 |  |  |  |  |  |  |
| 10 | Pc.c000803299.Contig1 | 1679 | - | 424039 | 425892 |  |  |  |  |  |  |  |  |  |  |  |  |  |  |
| 10 | PbContig112 | 12204 | - | 425311 | 437771 |  |  |  |  |  |  |  |  |  |  |  |  |  |  |
| 10 | MALPY03013 | 486 | + | 427561 | 428047 |  |  |  |  |  |  |  |  |  |  |  |  |  |  |
| 10 | MALPY02775 | 2336 | + | 429328 | 431664 |  |  |  |  |  |  | 283 - NS | 10000 |  |  |  |  |  |  |
| 10 | Pc.c000002566.Contig1 | 3801 | + | 430174 | 434108 | cRMPc05017 | 14553 | 3893 | 423218 | 437771 |  |  |  |  |  |  |  |  |  |
| 10 | MALPY02019 | 9716 | + | 441664 | 451783 |  |  |  |  |  |  | 283 - NS | 6000 |  |  |  |  |  |  |
| 10 | Pc.c001404029.Contig2 | 3932 | + | 442470 | 446581 |  |  |  |  |  |  |  |  |  |  |  |  |  |  |
| 10 | Pb.c000201533.Contig1 | 4484 | + | 442513 | 446997 |  |  |  |  |  |  |  |  |  |  |  |  |  |  |
| 10 | PcContig21 | 6016 | - | 447385 | 453404 | cRMPc05018 | 11740 | 2078 | 441664 | 453404 |  |  |  |  |  |  |  |  |  |
| 10 | Pb.c000401209.Contig1 | 10733 | - | 455482 | 468768 |  |  |  |  |  |  |  |  |  |  |  |  |  |  |
| 10 | PcContig729 | 6022 | - | 457281 | 463858 |  |  |  |  |  |  |  |  |  |  |  |  |  |  |
| 10 | MALPY01137 | 5146 | + | 457578 | 463227 |  |  |  |  |  |  | 283 | 500 |  |  |  |  |  |  |
| 10 | MALPY01934 | 11081 | + | 464825 | 475760 |  |  |  |  |  | 6 | 283 |  |  |  |  |  |  |  |
| 10 | Pb.c001201677.Contig1 | 4468 | - | 468314 | 472791 |  |  |  |  |  |  |  |  |  |  |  |  |  |  |
| 10 | Pc.c000502985.Contig1 | 4790 | + | 468572 | 473540 |  |  |  |  |  |  |  |  |  |  |  |  |  |  |
| 10 | Pb.c001302095.Contig1 | 921 | + | 472899 | 473820 |  |  |  |  |  |  |  |  |  |  |  |  |  |  |
| 10 | Pb.c001301919.Contig1 | 2429 | + | 473884 | 476313 |  |  |  |  |  |  |  |  |  |  |  |  |  |  |
| 10 | MALPY01973 | 3120 | - | 475680 | 478800 |  |  |  |  |  | 6 | 313 | 200 |  |  |  |  |  |  |
| 10 | PcContig431 | 4804 | - | 478211 | 483497 |  |  |  |  |  |  |  |  |  |  |  |  |  |  |
| 10 | MALPY00835 | 8676 | - | 479233 | 487338 |  |  |  |  |  | 506 | 313 |  |  |  |  |  |  |  |
| 10 | Pb.c000101437.Contig1 | 3750 | + | 480651 | 484401 |  |  |  |  |  |  |  |  |  |  |  |  |  |  |
| 10 | Pc.c000803387.Contig1 | 4436 | - | 482856 | 487298 |  |  |  |  |  |  |  |  |  |  |  |  |  |  |
| 10 | Pb.c001602041.Contig1 | 3882 | - | 484577 | 488465 |  |  |  |  |  |  |  |  |  |  |  |  |  |  |
| 10 | MALPY01765 | 9700 | + | 487259 | 497319 |  |  |  |  |  | 506 | 7 | 1600 |  |  |  |  |  |  |
| 10 | Pc.c001002685.Contig2 | 8477 | - | 487721 | 496186 |  |  |  |  |  |  |  |  |  |  |  |  |  |  |
| 10 | Pb.c000101116.Contig2 | 9157 | - | 488762 | 497907 | cRMPc05019 | 42425 | 1908 | 455482 | 497907 |  |  |  |  |  |  |  |  |  |
| 10 | MALPY00377 | 4522 | + | 499815 | 504349 |  |  |  |  |  | 130 |  |  |  |  |  |  |  |  |
| 10 | Pb.c000901831.Contig1 | 9715 | - | 500311 | 510066 |  |  |  |  |  |  |  |  |  |  |  |  |  |  |
| 10 | Pc.c000202688.Contig1 | 4476 | - | 502695 | 506499 |  |  |  |  |  |  |  |  |  |  |  |  |  |  |
| 10 | MALPY02542 | 4367 | + | 503698 | 507228 | cRMPc05020 | 10251 | 1778 | 499815 | 510066 | 130 | 7 | 2000 |  |  |  |  |  |  |
| 10 | MALPY00808 | 4311 | + | 511844 | 516233 |  |  |  |  |  |  | 7 | 300 |  |  |  |  |  |  |
| 10 | Pb.c001903626.Contig1 | 3591 | - | 515807 | 519688 |  |  |  |  |  |  |  |  |  |  |  |  |  |  |
| 10 | MALPY00571 | 16444 | + | 516013 | 534012 |  |  |  |  |  |  | 7 |  |  |  |  |  |  |  |
| 10 | Pc.c001103875.Contig1 | 1953 | - | 516985 | 519335 |  |  |  |  |  |  |  |  |  |  |  |  |  |  |
| 10 | Pb.c001702822.Contig1 | 4819 | - | 523415 | 528231 |  |  |  |  |  |  |  |  |  |  |  |  |  |  |
| 10 | PcContig148 | 3012 | - | 525663 | 528687 |  |  |  |  |  |  |  |  |  |  |  |  |  |  |
| 10 | Pb.c001101828.Contig1 | 7688 | - | 530438 | 539463 |  |  |  |  |  |  |  |  |  |  |  |  |  |  |
| 10 | Pc.c000603022.Contig1 | 1688 | + | 532075 | 533763 |  |  |  |  |  |  |  |  |  |  |  |  |  |  |
| 10 | MALPY01234 | 13520 | + | 536631 | 550454 |  |  |  |  |  | 40 |  |  |  |  |  |  |  |  |
| 10 | Pc.c000303033.Contig1 | 1909 | + | 538109 | 540006 |  |  |  |  |  |  |  |  |  |  |  |  |  |  |
| 10 | PcContig482 | 5939 | + | 540443 | 546156 |  |  |  |  |  |  |  |  |  |  |  |  |  |  |
| 10 | PbContig236 | 11214 | - | 543009 | 555120 |  |  |  |  |  |  |  |  |  |  |  |  |  |  |
| 10 | Pc.c000303064.Contig1 | 2942 | + | 546605 | 549572 |  |  |  |  |  |  |  |  |  |  |  |  |  |  |
| 10 | MALPY01405 | 9043 | + | 550152 | 559303 |  |  |  |  |  | 40 | 37 | 4500 |  |  |  |  |  |  |
| 10 | PcContig310 | 5834 | - | 550676 | 556573 | cRMPc05021 | 47459 | 282 | 511844 | 559303 |  |  |  |  |  |  |  |  |  |
| 10 | PcContig153 | 2708 | - | 559585 | 562293 | cRMPc05022 | 2708 | 1630 | 559585 | 562293 |  |  |  |  |  |  |  |  |  |
| 10 | MALPY00147 | 11598 | - | 563923 | 575830 |  |  |  |  |  | 690 | 37 |  |  |  |  |  |  |  |
| 10 | Pb.c002113282.Contig1 | 779 | - | 563923 | 564702 |  |  |  |  |  |  |  |  |  |  |  |  |  |  |
| 10 | PcContig553 | 5065 | + | 564499 | 570007 |  |  |  |  |  |  |  |  |  |  |  |  |  |  |
| 10 | Pb.c000101335.Contig1 | 3519 | - | 564907 | 568467 |  |  |  |  |  |  |  |  |  |  |  |  |  |  |
| 10 | Pb.c000501515.Contig1 | 3484 | - | 568880 | 572361 |  |  |  |  |  |  |  |  |  |  |  |  |  |  |
| 10 | MALPY01977 | 11562 | + | 575099 | 588919 |  |  |  |  |  | 690 - Ins |  |  |  |  |  |  |  |  |
| 10 | Pb.c000501169.Contig2 | 5162 | - | 576120 | 581283 | cRMPc05023 | 24996 | 130 | 563923 | 588919 |  |  |  |  |  |  |  |  |  |
| 10 | Pc.c001003372.Contig1 | 3248 | - | 589049 | 592348 |  |  |  |  |  |  |  |  |  |  |  |  |  |  |
| 10 | MALPY01976 | 4923 | - | 589419 | 594882 |  |  |  |  |  | 690 - Ins | 301 | 1300 |  |  |  |  |  |  |
| 10 | Pb.c001201671.Contig3 | 6143 | + | 591147 | 598334 |  |  |  |  |  |  |  |  |  |  |  |  |  |  |
| 10 | Pb.c001202357.Contig1 | 646 | + | 592779 | 593425 |  |  |  |  |  |  |  |  |  |  |  |  |  |  |
| 10 | PcContig636 | 1888 | + | 592823 | 594750 |  |  |  |  |  |  |  |  |  |  |  |  |  |  |
| 10 | Pc.c000502979.Contig1 | 2269 | + | 595867 | 598136 |  |  |  |  |  |  |  |  |  |  |  |  |  |  |
| 10 | MALPY02129 | 3960 | - | 596144 | 600092 |  |  |  |  |  | 39 | 301 |  |  |  |  |  |  |  |
| 10 | Pc.c000803270.Contig1 | 3185 | + | 598259 | 601441 |  |  |  |  |  |  |  |  |  |  |  |  |  |  |
| 10 | Pb.c001202231.Contig1 | 3885 | - | 600017 | 603902 |  |  |  |  |  |  |  |  |  |  |  |  |  |  |
| 10 | MALPY01402 | 7637 | - | 600104 | 607708 |  |  |  |  |  | 39 |  |  |  |  |  |  |  |  |
| 10 | Pb.c000201617.Contig1 | 2530 | - | 603180 | 605737 | cRMPc05024 | 18659 | 747 | 589049 | 607708 |  |  |  | cRMPs0504 | 198764 | 747 | 408944 | 607708 |  |
| 10 | MALPY00227 | 2642 | + | 608455 | 611064 |  |  |  |  |  |  | 243 | 6000 |  |  |  |  |  |  |
| 10 | PbContig28 | 2889 | - | 608741 | 611558 |  |  |  |  |  |  |  |  |  |  |  |  |  |  |
| 10 | Pc.c001811875.Contig1 | 581 | + | 609433 | 610014 |  |  |  |  |  |  |  |  |  |  |  |  |  |  |
| 10 | Pc.c001304868.Contig1 | 3216 | - | 609600 | 612753 | cRMPc05025 | 4298 | 4441 | 608455 | 612753 |  |  |  |  |  |  |  |  |  |
| 10 | Pc.c000202226.Contig1 | 3527 | + | 617194 | 620933 |  |  |  |  |  |  |  |  |  |  |  |  |  |  |
| 10 | MALPY00890 | 6415 | - | 617715 | 624488 |  |  |  |  |  | 1221 | 243 |  |  |  |  |  |  |  |
| 10 | Pb.c001502500.Contig1 | 7444 | + | 617742 | 625094 |  |  |  |  |  |  |  |  |  |  |  |  |  |  |
| 10 | MALPY03557 | 692 | - | 619727 | 620419 |  |  |  |  |  |  |  |  |  |  |  |  |  |  |
| 10 | Pc.c001813343.Contig1 | 608 | + | 622348 | 622975 |  |  |  |  |  |  |  |  |  |  |  |  |  |  |
| 10 | MALPY00715 | 9981 | - | 623732 | 633985 |  |  |  |  |  | 1221 | 3 | 400 |  |  |  |  |  |  |
| 10 | Pc.c001003412.Contig1 | 2823 | + | 624960 | 627718 |  |  |  |  |  |  |  |  |  |  |  |  |  |  |
| 10 | Pb.c001602505.Contig1 | 6517 | + | 625130 | 632222 | cRMPc05026 | 16791 | 847 | 617194 | 633985 |  |  |  |  |  |  |  |  |  |
| 10 | MALPY01465 | 10264 | + | 634832 | 644944 |  |  |  |  |  |  | 3 | 4500 |  |  |  |  |  |  |
| 10 | PcContig658 | 7956 | + | 635008 | 643351 |  |  |  |  |  |  |  |  |  |  |  |  |  |  |
| 10 | Pb.c001402051.Contig1 | 3726 | + | 635243 | 639046 |  |  |  |  |  |  |  |  |  |  |  |  |  |  |
| 10 | Pb.c001502088.Contig1 | 5143 | + | 640023 | 645131 | cRMPc05027 | 10299 | 4950 | 634832 | 645131 |  |  |  |  |  |  |  |  |  |
| 10 | PcContig495 | 5075 | + | 650081 | 655156 |  |  |  |  |  |  |  |  |  |  |  |  |  |  |
| 10 | PbContig363 | 14578 | - | 650098 | 664635 |  |  |  |  |  |  |  |  |  |  |  |  |  |  |
| 10 | MALPY00891 | 13900 | - | 653273 | 667107 |  |  |  |  |  | 320 | 3 |  |  |  |  |  |  |  |
| 10 | Pc.c000703207.Contig1 | 3701 | + | 654622 | 658296 |  |  |  |  |  |  |  |  |  |  |  |  |  |  |
| 10 | PcContig176 | 4216 | + | 660776 | 665244 |  |  |  |  |  |  |  |  |  |  |  |  |  |  |
| 10 | PbContig304 | 18791 | - | 664633 | 683462 |  |  |  |  |  |  |  |  |  |  |  |  |  |  |
| 10 | Pc.c001003252.Contig1 | 3244 | + | 665236 | 668486 |  |  |  |  |  |  |  |  |  |  |  |  |  |  |
| 10 | MALPY00131 | 13405 | - | 666289 | 680411 |  |  |  |  |  | 320 |  |  |  |  |  |  |  |  |
| 10 | Pc.c001204377.Contig1 | 1627 | + | 670287 | 671914 |  |  |  |  |  |  |  |  |  |  |  |  |  |  |
| 10 | Pc.c000002565.Contig1 | 2425 | - | 673859 | 676347 |  |  |  |  |  |  |  |  |  |  |  |  |  |  |
| 10 | MALPY01746 | 18506 | + | 680034 | 698195 |  |  |  |  |  | 320 |  |  |  |  |  |  |  |  |
| 10 | PcContig45 | 2727 | - | 681203 | 683821 |  |  |  |  |  |  |  |  |  |  |  |  |  |  |
| 10 | MALPY03292 | 852 | - | 682513 | 683377 |  |  |  |  |  |  |  |  |  |  |  |  |  |  |
| 10 | MALPY03219 | 331 | + | 682861 | 683192 |  |  |  |  |  |  |  |  |  |  |  |  |  |  |
| 10 | MALPY03496 | 910 | + | 682937 | 683811 |  |  |  |  |  |  |  |  |  |  |  |  |  |  |
| 10 | Pb.c001903712.Contig1 | 3202 | + | 683794 | 686978 |  |  |  |  |  |  |  |  |  |  |  |  |  |  |
| 10 | MALPY03218 | 402 | - | 683858 | 684260 |  |  |  |  |  |  |  |  |  |  |  |  |  |  |
| 10 | Pc.c000703267.Contig1 | 2993 | - | 683895 | 686876 |  |  |  |  |  |  |  |  |  |  |  |  |  |  |
| 10 | MALPY03329 | 703 | - | 683966 | 684672 |  |  |  |  |  |  |  |  |  |  |  |  |  |  |
| 10 | PbContig278 | 11847 | + | 686184 | 698557 |  |  |  |  |  |  |  |  |  |  |  |  |  |  |
| 10 | Pc.c000903821.Contig1 | 2530 | + | 692447 | 694977 |  |  |  |  |  |  |  |  |  |  |  |  |  |  |
| 10 | MALPY03577 | 242 | + | 694094 | 694327 |  |  |  |  |  |  |  |  |  |  |  |  |  |  |
| 10 | Pc.c000903361.Contig1 | 1689 | + | 694919 | 696612 |  |  |  |  |  |  |  |  |  |  |  |  |  |  |
| 10 | MALPY01161 | 9322 | - | 697892 | 706862 |  |  |  |  |  | 320 - Ins |  |  |  |  |  |  |  |  |
| 10 | PbContig118 | 7623 | + | 698012 | 705294 |  |  |  |  |  |  |  |  |  |  |  |  |  |  |
| 10 | Pc.c001404025.Contig2 | 3450 | - | 702128 | 705620 | cRMPc05028 | 56781 | 500 | 650081 | 706862 |  |  |  |  |  |  |  |  |  |
| 10 | MALPY00271 | 33877 | - | 707362 | 741696 |  |  |  |  |  | 320 - Ins | 268 | 1100 |  |  |  |  |  |  |
| man10 | PbContig54 - Ins | 25362 | - | 722052 | 747909 |  |  |  |  |  |  |  |  |  |  |  |  |  |  |
| 10 | PcContig29 | 11734 | + | 724159 | 736189 |  |  |  |  |  |  |  |  |  |  |  |  |  |  |
| 10 | Pc.c000602934.Contig1 | 1433 | + | 736118 | 737569 |  |  |  |  |  |  |  |  |  |  |  |  |  |  |
| 10 | MALPY03235 | 1620 | - | 737232 | 738855 |  |  |  |  |  |  |  |  |  |  |  |  |  |  |
| 10 | Pc.c000602930.Contig1 | 2102 | - | 740059 | 742161 |  |  |  |  |  |  |  |  |  |  |  |  |  |  |
| 10 | MALPY01157 | 17025 | + | 742670 | 759285 |  |  |  |  |  | 63 | 268 |  |  |  |  |  |  |  |
| 10 | Pc.c001504983.Contig1 | 2683 | - | 743118 | 745834 |  |  |  |  |  |  |  |  |  |  |  |  |  |  |
| 10 | MALPY03562 | 1421 | + | 744512 | 745967 |  |  |  |  |  |  |  |  |  |  |  |  |  |  |
| 10 | Pc.c001505441.Contig1 | 1303 | - | 745788 | 747091 |  |  |  |  |  |  |  |  |  |  |  |  |  |  |
| 10 | Pb.c001101932.Contig1 | 5626 | - | 748651 | 754625 |  |  |  |  |  |  |  |  |  |  |  |  |  |  |
| 10 | Pc.c001505101.Contig1 | 4374 | - | 749238 | 753901 | cRMPc05029 | 51923 | 272 | 707362 | 759285 |  |  |  |  |  |  |  |  |  |
| 10 | Pb.c001803397.Contig1 | 4136 | + | 759557 | 763693 |  |  |  |  |  |  |  |  |  |  |  |  |  |  |
| 10 | MALPY01226 | 14447 | + | 760140 | 774667 |  |  |  |  |  | 63 |  |  |  |  |  |  |  |  |
| 10 | PcContig508 | 8023 | + | 761083 | 769063 |  |  |  |  |  |  |  |  |  |  |  |  |  |  |
| 10 | Pb.c002113020.Contig1 | 1048 | + | 763886 | 764925 |  |  |  |  |  |  |  |  |  |  |  |  |  |  |
| 10 | Pb.c000201575.Contig1 | 5254 | + | 765117 | 770385 |  |  |  |  |  |  |  |  |  |  |  |  |  |  |
| 10 | MALPY03563 | 849 | + | 767512 | 768346 |  |  |  |  |  |  |  |  |  |  |  |  |  |  |
| 10 | Pb.c000201739.Contig1 | 1148 | + | 770627 | 771775 |  |  |  |  |  |  |  |  |  |  |  |  |  |  |
| 10 | Pb.c001904444.Contig1 | 3933 | - | 772404 | 776269 |  |  |  |  |  |  |  |  |  |  |  |  |  |  |
| TK10 | MALPY01023 | 2935 | + | 774705 | 777632 | cRMPc05030 | 18075 | 670 | 759557 | 777632 | 63 |  |  |  |  |  |  |  |  |
| TK10 | MALPY01513 | 2724 | + | 778302 | 781026 |  |  |  |  |  | 63 |  |  |  |  |  |  |  |  |
| 10 | Pc.c000703400.Contig1 | 3129 | - | 780624 | 783908 |  |  |  |  |  |  |  |  |  |  |  |  |  |  |
| 10 | MALPY02375 | 6460 | - | 781147 | 787676 |  |  |  |  |  | 63 | 296 | 500 |  |  |  |  |  |  |
| 10 | Pb.c001402140.Contig1 | 2399 | + | 782218 | 784767 |  |  |  |  |  |  |  |  |  |  |  |  |  |  |
| 10 | Pb.c001402487.Contig1 | 724 | + | 784814 | 785538 |  |  |  |  |  |  |  |  |  |  |  |  |  |  |
| 10 | Pb.c001502456.Contig1 | 2774 | + | 786031 | 788805 |  |  |  |  |  |  |  |  |  |  |  |  |  |  |
| 10 | MALPY00527 - BP | 13479 | - | 788315 | 801794 | cRMPc05031 | 23492 | 1800 | 778302 | 801794 | 945 | 296 - BP | 1800 |  |  |  |  |  |  |
| 4 | MALPY01752 - BP | 11015 | - | 803594 | 815829 |  |  |  |  |  | 945 | 296 - BP |  |  |  |  |  |  |  |
| 4 | Pb.c001802846.Contig1 | 3880 | + | 805731 | 810985 |  |  |  |  |  |  |  |  |  |  |  |  |  |  |
| 4 | Pc.c001707832.Contig1 | 5108 | - | 811837 | 817107 |  |  |  |  |  |  |  |  |  |  |  |  |  |  |
| 4 | Pb.c002112144.Contig1 | 1382 | + | 812462 | 813880 | cRMPc05032 | 13513 | 706 | 803594 | 817107 |  |  |  |  |  |  |  |  |  |
| 4 | MALPY03045 | 524 | - | 817813 | 818337 |  |  |  |  |  |  |  |  |  |  |  |  |  |  |
| 4 | MALPY02112 | 10899 | - | 818304 | 824210 |  |  |  |  |  |  | 296 | 2500 |  |  |  |  |  |  |
| 4 | Pb.c001803941.Contig1 | 1027 | + | 818383 | 819410 |  |  |  |  |  |  |  |  |  |  |  |  |  |  |
| 4 | Pb.c001301454.Contig1 | 5921 | - | 818487 | 824507 |  |  |  |  |  |  |  |  |  |  |  |  |  |  |
| 4 | Pc.c000502461.Contig1 | 5402 | - | 818663 | 824065 | cRMPc05033 | 6694 | 1873 | 817813 | 824507 |  |  |  |  |  |  |  |  |  |
| 4 | MALPY02133 | 16097 | + | 826380 | 843649 |  |  |  |  |  | 1207 - Ins | 296 | 1300 |  |  |  |  |  |  |
| 4 | Pb.c001201931.Contig1 | 11311 | - | 827803 | 840946 |  |  |  |  |  |  |  |  |  |  |  |  |  |  |
| 4 | Pc.c000903482.Contig1 | 1316 | - | 833813 | 835270 |  |  |  |  |  |  |  |  |  |  |  |  |  |  |
| 4 | Pc.c000502465.Contig1 | 4069 | - | 837178 | 841232 |  |  |  |  |  |  |  |  |  |  |  |  |  |  |
| 4 | Pc.c001505655.Contig1 | 801 | - | 840934 | 841735 | cRMPc05034 | 17269 | 500 | 826380 | 843649 |  |  |  |  |  |  |  |  |  |
| TK4 | MALPY00677 | 5271 | - | 844149 | 849508 | cRMPc05035 | 5359 | 6936 | 844149 | 849508 | 1207 - Ins |  |  | cRMPs0505 | 241053 | 6936 | 608455 | 849508 |  |
| 4 | Pb.c001904539.Contig1 | 3317 | - | 856444 | 859761 |  |  |  |  |  |  |  |  |  |  |  |  |  |  |
| 4 | MALPY01788 | 13495 | - | 856637 | 872903 |  |  |  |  |  | 180 |  |  |  |  |  |  |  |  |
| 4 | PcContig1 | 8383 | - | 860433 | 871700 |  |  |  |  |  |  |  |  |  |  |  |  |  |  |
| 4 | Pb.c001702873.Contig1 | 5658 | - | 860520 | 868223 |  |  |  |  |  |  |  |  |  |  |  |  |  |  |
| 4 | MALPY03435 | 1477 | - | 864993 | 866458 | cRMPc05036 | 16459 | 2285 | 856444 | 872903 |  |  |  |  |  |  |  |  |  |
| 4 | MALPY01222 | 15637 | - | 875188 | 891514 |  |  |  |  |  | 180 |  |  |  |  |  |  |  |  |
| 4 | PbContig386 | 2231 | + | 879419 | 881648 |  |  |  |  |  |  |  |  |  |  |  |  |  |  |
| 4 | Pb.c001202070.Contig1 | 1036 | + | 881299 | 882335 |  |  |  |  |  |  |  |  |  |  |  |  |  |  |
| 4 | PbContig228 | 3181 | + | 884879 | 888236 |  |  |  |  |  |  |  |  |  |  |  |  |  |  |
| 4 | MALPY03498 | 292 | - | 885426 | 885706 |  |  |  |  |  |  |  |  |  |  |  |  |  |  |
| 4 | Pc.c000002568.Contig1 | 5832 | - | 886350 | 892936 |  |  |  |  |  |  |  |  |  |  |  |  |  |  |
| 4 | Pb.c000001278.Contig2 | 3925 | - | 888025 | 892593 |  |  |  |  |  |  |  |  |  |  |  |  |  |  |
| 4 | MALPY01609 | 5663 | + | 891632 | 898189 |  |  |  |  |  | 180 |  |  |  |  |  |  |  |  |
| 4 | Pc.c001304163.Contig1 | 1089 | - | 893136 | 894237 |  |  |  |  |  |  |  |  |  |  |  |  |  |  |
| 4 | PbContig183 | 6153 | - | 894570 | 901437 |  |  |  |  |  |  |  |  |  |  |  |  |  |  |
| 4 | Pc.c001504191.Contig1 | 2805 | + | 894826 | 898286 |  |  |  |  |  |  |  |  |  |  |  |  |  |  |
| 4 | MALPY02193 | 24139 | + | 898378 | 925664 |  |  |  |  |  | 180 | 290 |  |  |  |  |  |  |  |
| 4 | Pc.c000703956.Contig1 | 1580 | + | 898867 | 900456 |  |  |  |  |  |  |  |  |  |  |  |  |  |  |
| 4 | PcContig324 | 9654 | + | 901190 | 911466 |  |  |  |  |  |  |  |  |  |  |  |  |  |  |
| 4 | Pb.c001101981.Contig1 | 2240 | + | 902963 | 905372 |  |  |  |  |  |  |  |  |  |  |  |  |  |  |
| 4 | Pb.c000801753.Contig1 | 3583 | + | 907684 | 911267 |  |  |  |  |  |  |  |  |  |  |  |  |  |  |
| 4 | Pb.c001001742.Contig1 | 3627 | - | 916427 | 920859 |  |  |  |  |  |  |  |  |  |  |  |  |  |  |
| 4 | Pc.c001003980.Contig1 | 936 | - | 917709 | 919002 |  |  |  |  |  |  |  |  |  |  |  |  |  |  |
| 4 | Pc.c001707900.Contig1 | 1354 | - | 919830 | 921157 |  |  |  |  |  |  |  |  |  |  |  |  |  |  |
| 4 | Pb.c002006945.Contig1 | 1600 | - | 921772 | 923372 | cRMPc05037 | 50476 | 993 | 875188 | 925664 |  |  |  |  |  |  |  |  |  |
| man4 | PcContig6880 | 7797 | + | 926657 | 934454 |  |  |  |  |  |  |  |  |  |  |  |  |  | MALPY01153 |
| JC4 | MALPY01153 | 4128 | - | 926664 | 930792 |  |  |  |  |  |  | 290 | 1000 |  |  |  |  |  | \| |
| man4 | PbContig1513 | 1213 | - | 930121 | 931334 |  |  |  |  |  |  |  |  |  |  |  |  |  | \| |
| JC4 | MALPY01062 | 6156 | - | 931426 | 937582 |  |  |  |  |  |  | 290 | 1195 |  |  |  |  |  | \| |
| man4 | PcContig7072 | 5620 | + | 934400 | 940020 |  |  |  |  |  |  |  |  |  |  |  |  |  | \| |
| man4 | PbContig4956 | 3511 | + | 935134 | 938645 |  |  |  |  |  |  |  |  |  |  |  |  |  | \| |
| JC4 | MALPY02761 | 2665 | - | 938161 | 940826 |  |  |  |  |  |  | 290 | 500 |  |  |  |  |  | \| |
| man4 | PcContig3451 | 1319 | - | 940000 | 941319 |  |  |  |  |  |  |  |  |  |  |  |  |  | \| |
| man4 | PbContig6514 | 1742 | - | 940396 | 942138 | cRMPc05038 | 15481 |  | 926657 | 942138 |  |  |  | cRMPs0506 | 85694 |  | 856444 | 942138 | MALPY02761 |
| Telomere | |  |  |  |  |  |  |  |  |  |  |  |  |  |  |  |  |  |  |
|  |  | Pb Pc Py contig |  |  |  |  | cRMP contig | Gap to next cRMP contig |  |  |  |  |  |  | cRMP scaffold | Gap to next cRMP scaffold |  |  |  |
|  | Number | 337 |  |  |  | Number | 38 | 37 |  |  |  |  |  | Number | 6 | 5 |  |  |  |
|  | Average size (bp) | 5190 |  |  |  | Average size (bp) | 22700 | 2150 |  |  |  |  |  | Average size (bp) | 155233 | 2148 |  |  |  |
|  | Minimum size (bp) | 242 |  |  |  | Minimum size (bp) | 2708 | 74 |  |  |  |  |  | Minimum size (bp) | 22484 | 74 |  |  |  |
|  | Maximum size (bp) | 33877 |  |  |  | Maximum size (bp) | 75097 | 13157 |  |  |  |  |  | Maximum size (bp) | 359132 | 6936 |  |  |  |
|  | Total size (bp) | 1749162 |  |  |  | Total size (bp) | 862602 | 79536 |  |  |  |  |  | Total size (bp) | 931400 | 10738 |  |  |  |
|  |  |  |  |  |  |  |  |  |  |  |  |  |  |  |  |  |  |  |  |
