# Supplementary material for: A Plasmodium Whole-Genome Synteny Map: Indels and Synteny Breakpoints as Foci for Species-Specific Genes
Source: PLoS Pathog. 2005 Dec 23;1(4):e44. doi: 10.1371/journal.ppat.0010044 (PMC1317653; doi:10.1371/journal.ppat.0010044)
Supplement: Supplementary file 6 [file ppat.0010044.st022.doc]

Table S22 - RMPchr6


# Table S22 - RMPchr6

|  |  |  |  |  |  |  |  |  |  |  |  |  |  |  |  |  |  |  |  |
| --- | --- | --- | --- | --- | --- | --- | --- | --- | --- | --- | --- | --- | --- | --- | --- | --- | --- | --- | --- |
| Table S22: Tiling path of RMP contigs combined to form cRMP chromosome 6. | | | | | | |  |  |  |  |  |  |  |  |  |  |  |  |  |
|  |  |  |  |  |  |  |  |  |  |  |  |  |  |  |  |  |  |  |  |
| Tiling paths of P. berghei (Pb), P. c. chabaudi (Pc) and P. y. yoelii (Py) contigs combined into composite rodent malaria parasite (cRMP) | | | | | | | | | |  |  |  |  |  |  |  |  |  |  |
| contigs aligned against parts of the 14 chromosomes of P. falciparum (Pf). The cRMP contigs are further linked by physical linkage | | | | | | | | | |  |  |  |  |  |  |  |  |  |  |
| and PCR (see Methods) to form cRMP scaffolds. Different synteny blocks with homology to different P. falciparum chromosomes | | | | | | | | | |  |  |  |  |  |  |  |  |  |  |
| together form the cRMP chromosomes. The tables also show gaps in the alignment (black lines), cRMP telomeres (white bars) and | | | | | | | | | |  |  |  |  |  |  |  |  |  |  |
| predicted centromeres (plum/black bars). Contigs, physical groups or PCR groups interrupted by inter- or intrasyntenic regions are | | | | | | | | | |  |  |  |  |  |  |  |  |  |  |
| printed in bold. A summary of the number of RMP contigs, cRMP contigs and cRMP scaffolds as well as their average, minimum, | | | | | | | | | |  |  |  |  |  |  |  |  |  |  |
| maximum and total sizes is provided. Column headings are as follows: | | | | |  |  |  |  |  |  |  |  |  |  |  |  |  |  |  |
|  |  |  |  |  |  |  |  |  |  |  |  |  |  |  |  |  |  |  |  |
| Pf chr.: P. falciparum chromosome to which the RMP contigs are aligned | | | | | |  |  |  |  |  |  |  |  |  |  |  |  |  |  |
| (#: PROmer analysis� from this study; JC# and TK#: Carlton et al.; man#: manual BLAST analysis; | | | | | | |  |  |  |  |  |  |  |  |  |  |  |  |  |
| i: inversed orientation of the synteny block; colours refer to those used in Fig. 2 of the paper); | | | | | | |  |  |  |  |  |  |  |  |  |  |  |  |  |
| Pb Pc Py contig: names of the aligned RMP contigs; | | |  |  |  |  |  |  |  |  |  |  |  |  |  |  |  |  |  |
| Size: size (bp) of the aligned RMP contigs; | |  |  |  |  |  |  |  |  |  |  |  |  |  |  |  |  |  |  |
| +/-: orientation of the aligned RMP contigs; | |  |  |  |  |  |  |  |  |  |  |  |  |  |  |  |  |  |  |
| Start contig & End contig: estimated start & end coordinates of alignment of the RMP contigs based on the coordinates | | | | | | | | |  |  |  |  |  |  |  |  |  |  |  |
| of PROmer hits corrected for the contig�s size and orientation and presence of inter- and intrasyntenic regions | | | | | | | |  |  |  |  |  |  |  |  |  |  |  |  |
| (the start position of the first RMP contig in the alignment was set at zero); | | | | | |  |  |  |  |  |  |  |  |  |  |  |  |  |  |
| cRMP contig: names of cRMP contigs | |  |  |  |  |  |  |  |  |  |  |  |  |  |  |  |  |  |  |
| (the first 2 digits refer to the cRMP chromosome number and the following 3 digits designate the order of the contigs); | | | | | | | | |  |  |  |  |  |  |  |  |  |  |  |
| cRMP contig size: size of cRMP contigs based on the corrected coordinates of alignment; | | | | | | |  |  |  |  |  |  |  |  |  |  |  |  |  |
| Gap size to next cRMP contig: size of gaps between neighbouring cRMP contigs; | | | | | | |  |  |  |  |  |  |  |  |  |  |  |  |  |
| Start cRMP contig & End cRMP contig: estimated start & end coordinates of alignment of the cRMP contigs; | | | | | | | |  |  |  |  |  |  |  |  |  |  |  |  |
| Phys. group (Py): physical linkage of neighbouring P. y. yoelii contigs; | | | | | |  |  |  |  |  |  |  |  |  |  |  |  |  |  |
| PCR group (Py): neighbouring P. y. yoelii contigs were linked previously or for this study by PCR; | | | | | | |  |  |  |  |  |  |  |  |  |  |  |  |  |
| PCR size (Py): size of the PCR product; | |  |  |  |  |  |  |  |  |  |  |  |  |  |  |  |  |  |  |
| cRMP scaffold: names of cRMP scaffolds | |  |  |  |  |  |  |  |  |  |  |  |  |  |  |  |  |  |  |
| (the first 2 digits refer to the cRMP chromosome number and the following 2 digits designate the order of the scaffolds); | | | | | | | | |  |  |  |  |  |  |  |  |  |  |  |
| cRMP scaffold size: size of cRMP scaffolds; | | |  |  |  |  |  |  |  |  |  |  |  |  |  |  |  |  |  |
| Gap size to next cRMP scaffold: size of gaps between neighbouring cRMP scaffolds; | | | | | | |  |  |  |  |  |  |  |  |  |  |  |  |  |
| Start cRMP scaffold & End cRMP scaffold: estimated start & end coordinates of alignment of the cRMP scaffolds; | | | | | | | | |  |  |  |  |  |  |  |  |  |  |  |
| Overlap: Linkage between RMP contigs established through manual BLAST analysis. | | | | | | |  |  |  |  |  |  |  |  |  |  |  |  |  |
|  |  |  |  |  |  |  |  |  |  |  |  |  |  |  |  |  |  |  |  |
| Pf chr. | Pb Pc Py contig | Size | +/- | Start contig | End contig | cRMP contig | cRMP contig size | Gap size to next cRMP contig | Start cRMP contig | End cRMP contig | Phys. group (Py) | PCR group (Py) | PCR size (Py) | cRMP scaffold | cRMP scaffold size | Gap size to next cRMP scaffold | Start cRMP scaffold | End cRMP scaffold | Overlap (manual) |
| Telomere | |  |  |  |  |  |  |  |  |  |  |  |  |  |  |  |  |  |  |
| JC12 | MALPY00907 | 7061 | xxx | 0 | 7061 | cRMPc06001 | 7061 | 500 | 0 | 7061 | 31 |  |  |  |  |  |  |  |  |
| man12 | MALPY00560 | 7069 | + | 7561 | 14630 |  |  |  |  |  | 31 |  |  |  |  |  |  |  | PbContig5596 |
| man12 | PbContig5596 | 7759 | - | 9419 | 17178 |  |  |  |  |  |  |  |  |  |  |  |  |  | MALPY00560/MALPY00396 |
| 12 | Pb.c001101533.Contig1 | 6375 | + | 10801 | 17170 |  |  |  |  |  |  |  |  |  |  |  |  |  |  |
| 12 | MALPY00396 | 2443 | - | 14566 | 17033 | cRMPc06002 | 9617 | 461 | 7561 | 17178 | 31 |  |  | cRMPs0601 | 17178 | 461 | 0 | 17178 |  |
| 12 | Pb.c001001996.Contig1 | 1160 | - | 17639 | 18805 |  |  |  |  |  |  |  |  |  |  |  |  |  |  |
| 12 | MALPY03253 | 1013 | + | 17801 | 18817 | cRMPc06003 | 1178 | 149 | 17639 | 18817 |  | 21 | 2500 |  |  |  |  |  |  |
| 12 | PbContig109 | 9339 | - | 18966 | 27880 |  |  |  |  |  |  |  |  |  |  |  |  |  |  |
| 12 | MALPY01256 | 6681 | + | 21259 | 27487 |  |  |  |  |  |  | 21 |  |  |  |  |  |  |  |
| 12 | Pc.c001708639.Contig1 | 909 | + | 22319 | 23228 |  |  |  |  |  |  |  |  |  |  |  |  |  |  |
| 12 | MALPY03005 | 714 | - | 23351 | 24064 |  |  |  |  |  |  | 41 | 2000 |  |  |  |  |  |  |
| man12 | MALPY00130 - Ins | 10787 | + | 25797 | 36197 |  |  |  |  |  |  | 41 |  |  |  |  |  |  |  |
| 12 | Pc.c001304329.Contig1 | 1067 | + | 26144 | 27317 |  |  |  |  |  |  |  |  |  |  |  |  |  |  |
| 12 | Pb.c000901472.Contig1 | 6148 | + | 28958 | 35107 |  |  |  |  |  |  |  |  |  |  |  |  |  |  |
| 12 | Pc.c001003449.Contig1 | 3611 | + | 31053 | 34664 | cRMPc06004 | 17231 | 1577 | 18966 | 36197 |  |  |  | cRMPs0602 | 18558 | 1577 | 17639 | 36197 |  |
| 12 | Pc.c000402953.Contig1 | 2808 | - | 37774 | 40563 |  |  |  |  |  |  |  |  |  |  |  |  |  |  |
| 12 | MALPY03128 | 1852 | + | 38267 | 40129 | cRMPc06005 | 2789 | 1351 | 37774 | 40563 |  |  |  | cRMPs0603 | 2789 | 1351 | 37774 | 40563 |  |
| 12 | MALPY01539 | 4354 | - | 41914 | 46268 | cRMPc06006 | 4354 | 2997 | 41914 | 46268 |  |  |  | cRMPs0604 | 4354 | 2997 | 41914 | 46268 |  |
| 12 | Pc.c000202729.Contig1 | 2727 | + | 49265 | 51992 |  |  |  |  |  |  |  |  |  |  |  |  |  |  |
| 12 | MALPY02986 | 502 | + | 50545 | 51047 | cRMPc06007 | 2727 | 95 | 49265 | 51992 |  |  |  | cRMPs0605 | 2727 | 95 | 49265 | 51992 |  |
| 12 | MALPY00405 | 18042 | - | 52087 | 70863 |  |  |  |  |  |  | 249 | 1300 |  |  |  |  |  |  |
| 12 | PcContig171 | 2444 | + | 52590 | 55338 |  |  |  |  |  |  |  |  |  |  |  |  |  |  |
| 12 | Pc.c001405127.Contig1 | 1045 | - | 55500 | 56574 |  |  |  |  |  |  |  |  |  |  |  |  |  |  |
| 12 | Pc.c000002605.Contig1 | 1769 | - | 56772 | 58525 |  |  |  |  |  |  |  |  |  |  |  |  |  |  |
| 12 | Pb.c001602491.Contig1 | 5540 | - | 62735 | 68307 |  |  |  |  |  |  |  |  |  |  |  |  |  |  |
| 12 | Pc.c000302831.Contig1 | 4198 | - | 65435 | 69792 |  |  |  |  |  |  |  |  |  |  |  |  |  |  |
| 12 | Pb.c001904334.Contig1 | 1519 | - | 68426 | 69939 | cRMPc06008 | 18776 | 466 | 52087 | 70863 |  |  |  |  |  |  |  |  |  |
| 12 | MALPY01639 | 5777 | - | 71329 | 77106 |  |  |  |  |  | 436 | 249 |  |  |  |  |  |  |  |
| 12 | Pc.c001605888.Contig1 | 3120 | + | 75131 | 78251 |  |  |  |  |  |  |  |  |  |  |  |  |  |  |
| 12 | Pb.c000701524.Contig1 | 1904 | + | 75789 | 77693 | cRMPc06009 | 6922 | 537 | 71329 | 78251 |  |  |  |  |  |  |  |  |  |
| 12 | MALPY01673 | 14426 | + | 78788 | 93997 |  |  |  |  |  | 436 |  |  |  |  |  |  |  |  |
| 12 | PbContig182 | 6788 | + | 79638 | 86438 |  |  |  |  |  |  |  |  |  |  |  |  |  |  |
| 12 | Pc.c000202734.Contig1 | 4368 | + | 84402 | 89439 |  |  |  |  |  |  |  |  |  |  |  |  |  |  |
| 12 | Pc.c000003108.Contig1 | 617 | + | 84952 | 85569 |  |  |  |  |  |  |  |  |  |  |  |  |  |  |
| 12 | MALPY03129 | 580 | - | 88978 | 89552 |  |  |  |  |  |  |  |  |  |  |  |  |  |  |
| 12 | Pc.c000202731.Contig1 | 3975 | + | 89535 | 93495 | cRMPc06010 | 15209 | 786 | 78788 | 93997 |  |  |  | cRMPs0606 | 41910 | 786 | 52087 | 93997 |  |
| 12 | Pb.c001001378.Contig1 | 8491 | + | 94783 | 103647 |  |  |  |  |  |  |  |  |  |  |  |  |  |  |
| 12 | MALPY01948 | 6824 | + | 95721 | 102997 |  |  |  |  |  |  |  |  |  |  |  |  |  |  |
| 12 | Pc.c000002601.Contig1 | 6342 | - | 97673 | 104432 | cRMPc06011 | 9649 | 2665 | 94783 | 104432 |  |  |  | cRMPs0607 | 9649 | 2665 | 94783 | 104432 |  |
| 12 | MALPY00859 | 3006 | - | 107097 | 109947 |  |  |  |  |  |  | 151 | 1400 |  |  |  |  |  |  |
| 12 | Pc.c000202204.Contig5 | 5980 | - | 107467 | 113463 |  |  |  |  |  |  |  |  |  |  |  |  |  |  |
| 12 | Pb.c001402160.Contig1 | 2457 | + | 107792 | 110321 |  |  |  |  |  |  |  |  |  |  |  |  |  |  |
| 12 | MALPY00265 | 10931 | + | 112244 | 123419 |  |  |  |  |  |  | 151 | 900 |  |  |  |  |  |  |
| 12 | Pb.c001101898.Contig1 | 4485 | - | 117843 | 122711 |  |  |  |  |  |  |  |  |  |  |  |  |  |  |
| 12 | Pc.c001404877.Contig1 | 1559 | + | 119115 | 120662 |  |  |  |  |  |  |  |  |  |  |  |  |  |  |
| 12 | Pc.c001204056.Contig1 | 5010 | + | 121853 | 126863 |  |  |  |  |  |  |  |  |  |  |  |  |  |  |
| 12 | Pb.c001001726.Contig1 | 7416 | - | 124229 | 130988 |  |  |  |  |  |  |  |  |  |  |  |  |  |  |
| 12 | MALPY01451 | 2218 | + | 124734 | 126952 |  |  |  |  |  | 163 | 151 |  |  |  |  |  |  |  |
| 12 | MALPY01452 | 2396 | - | 126272 | 128674 |  |  |  |  |  | 163 |  |  |  |  |  |  |  |  |
| 12 | PcContig23 | 10239 | - | 127425 | 139588 |  |  |  |  |  |  |  |  |  |  |  |  |  |  |
| 12 | MALPY01707 | 11279 | + | 129954 | 141621 |  |  |  |  |  | 163 |  |  |  |  |  |  |  |  |
| 12 | PbContig82 | 10016 | - | 132793 | 143151 |  |  |  |  |  |  |  |  |  |  |  |  |  |  |
| 12 | Pc.c000102511.Contig1 | 1105 | - | 140263 | 141371 | cRMPc06012 | 36054 | 1248 | 107097 | 143151 |  |  |  | cRMPs0608 | 36054 | 1248 | 107097 | 143151 |  |
| 12 | Pb.c001402220.Contig1 | 4984 | + | 144399 | 149458 |  |  |  |  |  |  |  |  |  |  |  |  |  |  |
| 12 | MALPY02711 | 4105 | + | 145583 | 149796 |  |  |  |  |  |  | 396 | 6000 |  |  |  |  |  |  |
| 12 | Pc.c000903356.Contig1 | 1681 | + | 146880 | 148573 | cRMPc06013 | 5397 | 6316 | 144399 | 149796 |  |  |  |  |  |  |  |  |  |
| 12 | MALPY00477 | 10165 | - | 156112 | 167182 |  |  |  |  |  | 351 | 396 |  |  |  |  |  |  |  |
| 12 | Pc.c001003838.Contig1 | 1420 | + | 156536 | 157953 |  |  |  |  |  |  |  |  |  |  |  |  |  |  |
| 12 | Pb.c000201616.Contig1 | 7353 | + | 157435 | 166217 |  |  |  |  |  |  |  |  |  |  |  |  |  |  |
| 12 | Pc.c001003290.Contig1 | 2708 | + | 158401 | 161019 |  |  |  |  |  |  |  |  |  |  |  |  |  |  |
| 12 | Pc.c000703363.Contig1 | 2444 | + | 163107 | 165714 |  |  |  |  |  |  |  |  |  |  |  |  |  |  |
| 12 | MALPY00480 | 2502 | + | 166867 | 169363 |  |  |  |  |  | 351 |  |  |  |  |  |  |  |  |
| 12 | Pb.c000701563.Contig1 | 1142 | + | 166922 | 168058 |  |  |  |  |  |  |  |  |  |  |  |  |  |  |
| 12 | PcContig227 | 1252 | + | 167608 | 168845 | cRMPc06014 | 13251 | 1172 | 156112 | 169363 |  |  |  | cRMPs0609 | 24964 | 1172 | 144399 | 169363 |  |
| 12 | MALPY02014 | 7083 | + | 170535 | 177548 |  |  |  |  |  | 190 |  |  |  |  |  |  |  |  |
| 12 | Pc.c001304355.Contig1 | 1437 | + | 172112 | 173552 |  |  |  |  |  |  |  |  |  |  |  |  |  |  |
| 12 | PbContig395 | 10172 | - | 173871 | 184217 |  |  |  |  |  |  |  |  |  |  |  |  |  |  |
| 12 | Pc.c000903424.Contig1 | 4328 | + | 173985 | 178316 |  |  |  |  |  |  |  |  |  |  |  |  |  |  |
| 12 | MALPY02741 | 4975 | + | 177831 | 182599 |  |  |  |  |  | 190 |  |  |  |  |  |  |  |  |
| 12 | Pc.c000202179.Contig1.0 | 3416 | + | 178737 | 181937 |  |  |  |  |  |  |  |  |  |  |  |  |  |  |
| man12 | MALPY00245 - Ins | 7856 | + | 183590 | 191806 |  |  |  |  |  | 1084 |  |  |  |  |  |  |  |  |
| 12 | PcContig749 | 6819 | - | 184340 | 191345 |  |  |  |  |  |  |  |  |  |  |  |  |  |  |
| 12 | PbContig255 | 4823 | - | 185341 | 190339 |  |  |  |  |  |  |  |  |  |  |  |  |  |  |
| 12 | Pb.c001301518.Contig2 | 15820 | - | 190954 | 207360 |  |  |  |  |  |  |  |  |  |  |  |  |  |  |
| 12 | Pc.c000803288.Contig1 | 6942 | - | 192312 | 199413 |  |  |  |  |  |  |  |  |  |  |  |  |  |  |
| 12 | MALPY00503 | 18681 | + | 192373 | 210708 |  |  |  |  |  | 1084 |  |  |  |  |  |  |  |  |
| 12 | Pc.c000103092.Contig1 | 1781 | + | 204051 | 206028 |  |  |  |  |  |  |  |  |  |  |  |  |  |  |
| 12 | Pc.c001204494.Contig1 | 846 | - | 207047 | 207891 |  |  |  |  |  |  |  |  |  |  |  |  |  |  |
| 12 | MALPY03338 | 370 | - | 207114 | 207484 |  |  |  |  |  |  |  |  |  |  |  |  |  |  |
| 12 | Pb.c001101873.Contig1 | 2397 | - | 207812 | 210210 | cRMPc06015 | 40173 | 151 | 170535 | 210708 |  |  |  | cRMPs0610 | 40173 | 151 | 170535 | 210708 |  |
| 12 | Pc.c001405052.Contig1 | 2039 | - | 210859 | 213036 |  |  |  |  |  |  |  |  |  |  |  |  |  |  |
| 12 | Pb.c001001737.Contig1 | 3930 | - | 211129 | 215209 |  |  |  |  |  |  |  |  |  |  |  |  |  |  |
| 12 | MALPY02561 | 3725 | - | 211306 | 215184 | cRMPc06016 | 4350 | 2368 | 210859 | 215209 |  | 465 | 6500 |  |  |  |  |  |  |
| man12 | MALPY00755 | 3824 | - | 217577 | 221548 |  |  |  |  |  | 1060 |  |  |  |  |  |  |  |  |
| 12 | MALPY01953 | 9888 | - | 221172 | 231371 |  |  |  |  |  | 1060 | 465 |  |  |  |  |  |  |  |
| 12 | Pb.c002113213.Contig1 | 912 | + | 221881 | 222802 | cRMPc06017 | 13794 | 2 | 217577 | 231371 |  |  |  |  |  |  |  |  |  |
| man12 | MALPY01387 | 2420 | - | 231373 | 233815 |  |  |  |  |  | 1060 |  |  |  |  |  |  |  |  |
| 12 | MALPY02148 | 3761 | - | 233780 | 237683 |  |  |  |  |  | 1060 | 2 - Ins | 2600 |  |  |  |  |  |  |
| 12 | Pb.c000601584.Contig1 | 1186 | + | 234562 | 235748 |  |  |  |  |  |  |  |  |  |  |  |  |  |  |
| 12 | Pc.c000703390.Contig1 | 1932 | + | 235111 | 237210 |  |  |  |  |  |  |  |  |  |  |  |  |  |  |
| 12 | Pb.c001301796.Contig1 | 1655 | + | 236036 | 237711 | cRMPc06018 | 6338 | 2572 | 231373 | 237711 |  |  |  |  |  |  |  |  |  |
| 12 | MALPY01248 | 16133 | + | 240283 | 256418 |  |  |  |  |  | 393 | 2 - Ins |  |  |  |  |  |  |  |
| 12 | PbContig309 | 12712 | + | 241061 | 253854 |  |  |  |  |  |  |  |  |  |  |  |  |  |  |
| 12 | Pc.c000602384.Contig1 | 3044 | - | 242133 | 245140 |  |  |  |  |  |  |  |  |  |  |  |  |  |  |
| 12 | Pc.c000002057.Contig2 | 3807 | + | 246910 | 250714 | cRMPc06019 | 16135 | 476 | 240283 | 256418 |  |  |  |  |  |  |  |  |  |
| 12 | MALPY01019 | 5497 | + | 256894 | 262394 | cRMPc06020 | 5500 | 510 | 256894 | 262394 | 393 |  |  |  |  |  |  |  |  |
| 12 | MALPY00021 | 20103 | + | 262904 | 283115 |  |  |  |  |  | 393 |  |  |  |  |  |  |  |  |
| 12 | PbContig73 | 3097 | + | 265354 | 268448 |  |  |  |  |  |  |  |  |  |  |  |  |  |  |
| 12 | Pb.c001502536.Contig1 | 3465 | + | 268464 | 271935 |  |  |  |  |  |  |  |  |  |  |  |  |  |  |
| 12 | Pb.c000501444.Contig1 | 7938 | - | 273543 | 281481 |  |  |  |  |  |  |  |  |  |  |  |  |  |  |
| 12 | Pc.c000703354.Contig1 | 1972 | + | 273673 | 275642 |  |  |  |  |  |  |  |  |  |  |  |  |  |  |
| 12 | Pc.c001708501.Contig1 | 649 | + | 275815 | 276464 |  |  |  |  |  |  |  |  |  |  |  |  |  |  |
| 12 | Pb.c001703222.Contig1 | 1205 | - | 282015 | 283220 | cRMPc06021 | 20316 | 108 | 262904 | 283220 |  |  |  |  |  |  |  |  |  |
| JC12 | MALPY03177 | 1463 | + | 283328 | 284761 |  |  |  |  |  | 393 |  |  |  |  |  |  |  |  |
| 12 | MALPY02063 | 13338 | - | 284671 | 299277 |  |  |  |  |  | 393 | 50 | 600 |  |  |  |  |  |  |
| 12 | Pb.c001904352.Contig1 | 2877 | - | 286373 | 289211 |  |  |  |  |  |  |  |  |  |  |  |  |  |  |
| 12 | Pc.c001003953.Contig1 | 908 | + | 286566 | 287471 |  |  |  |  |  |  |  |  |  |  |  |  |  |  |
| 12 | Pb.c000601524.Contig1 | 2341 | - | 289481 | 291816 |  |  |  |  |  |  |  |  |  |  |  |  |  |  |
| 12 | Pb.c000401762.Contig1 | 1018 | - | 291800 | 292782 |  |  |  |  |  |  |  |  |  |  |  |  |  |  |
| 12 | Pc.c001003447.Contig1 | 2732 | + | 294380 | 297157 |  |  |  |  |  |  |  |  |  |  |  |  |  |  |
| 12 | Pc.c000803643.Contig1 | 627 | + | 297912 | 298518 |  |  |  |  |  |  |  |  |  |  |  |  |  |  |
| 12 | MALPY02249 | 5684 | - | 299169 | 305387 |  |  |  |  |  |  | 50 |  |  |  |  |  |  |  |
| 12 | Pb.c000401465.Contig1 | 1434 | + | 300681 | 302112 | cRMPc06022 | 22059 | 4448 | 283328 | 305387 |  |  |  | cRMPs0611 | 94528 | 4448 | 210859 | 305387 |  |
| 12 | MALPY01121 | 5213 | + | 309835 | 315198 |  |  |  |  |  |  |  |  |  |  |  |  |  |  |
| 12 | Pc.c001813437.Contig1 | 676 | - | 312030 | 312706 |  |  |  |  |  |  |  |  |  |  |  |  |  |  |
| 12 | Pb.c001001433.Contig1 | 2632 | - | 312450 | 315223 |  |  |  |  |  |  |  |  |  |  |  |  |  |  |
| 12 | Pc.c000703327.Contig1 | 2010 | - | 312677 | 314826 |  |  |  |  |  |  |  |  |  |  |  |  |  |  |
| 12 | MALPY03479 | 777 | - | 312972 | 313875 |  |  |  |  |  |  | 362 | 3000 |  |  |  |  |  |  |
| 12 | MALPY02358 | 3246 | + | 313022 | 316408 |  |  |  |  |  |  |  |  |  |  |  |  |  |  |
| 12 | Pb.c001502447.Contig1 | 3188 | + | 316133 | 319321 |  |  |  |  |  |  |  |  |  |  |  |  |  |  |
| 12 | MALPY02311 | 2334 | - | 316152 | 318487 | cRMPc06023 | 9486 | 1666 | 309835 | 319321 |  | 362 - NS | 2500 |  |  |  |  |  |  |
| 12 | MALPY01059 | 3230 | + | 320987 | 323718 |  |  |  |  |  | 169 | 362 - NS | 3000 |  |  |  |  |  |  |
| 12 | Pb.c001905073.Contig1 | 1484 | + | 321322 | 322806 |  |  |  |  |  |  |  |  |  |  |  |  |  |  |
| man12 | MALPY01768 | 3287 | - | 322551 | 326988 |  |  |  |  |  | 169 |  |  |  |  |  |  |  |  |
| 12 | Pc.c001812441.Contig1 | 1148 | - | 322916 | 324034 | cRMPc06024 | 6001 | 321 | 320987 | 326988 |  |  |  |  |  |  |  |  |  |
| 12 | MALPY00064 | 3611 | + | 327309 | 330926 |  |  |  |  |  |  | 362 |  |  |  |  |  |  |  |
| 12 | Pb.c000001782.Contig1 | 3650 | - | 329263 | 333372 |  |  |  |  |  |  |  |  |  |  |  |  |  |  |
| 12 | Pc.c000603488.Contig1 | 702 | - | 329724 | 330462 |  |  |  |  |  |  |  |  |  |  |  |  |  |  |
| 12 | MALPY02818 | 2419 | - | 330145 | 333125 |  |  |  |  |  |  | 359 | 2500 |  |  |  |  |  |  |
| 12 | Pc.c000703326.Contig1 | 1674 | - | 330815 | 332738 | cRMPc06025 | 6063 | 1515 | 327309 | 333372 |  |  |  |  |  |  |  |  |  |
| 12 | MALPY00497 | 12351 | - | 334887 | 346511 |  |  |  |  |  | 85 | 359 |  |  |  |  |  |  |  |
| 12 | Pb.c001201968.Contig1 | 3707 | + | 335615 | 339413 |  |  |  |  |  |  |  |  |  |  |  |  |  |  |
| 12 | Pc.c000303035.Contig1 | 1274 | + | 335686 | 337021 |  |  |  |  |  |  |  |  |  |  |  |  |  |  |
| 12 | Pc.c001505093.Contig1 | 1114 | + | 337235 | 338319 |  |  |  |  |  |  |  |  |  |  |  |  |  |  |
| 12 | Pb.c000301457.Contig1 | 4189 | + | 341621 | 345624 |  |  |  |  |  |  |  |  |  |  |  |  |  |  |
| 12 | Pc.c001706597.Contig1 | 3076 | - | 341773 | 344622 |  |  |  |  |  |  |  |  |  |  |  |  |  |  |
| 12 | Pc.c000602830.Contig1 | 8490 | + | 343440 | 352092 |  |  |  |  |  |  |  |  |  |  |  |  |  |  |
| 12 | MALPY01006 | 10326 | - | 345702 | 356598 |  |  |  |  |  | 85 |  |  |  |  |  |  |  |  |
| 12 | Pb.c002007043.Contig1 | 4138 | + | 351738 | 356397 |  |  |  |  |  |  |  |  |  |  |  |  |  |  |
| 12 | MALPY02489 | 5238 | + | 356527 | 361765 |  |  |  |  |  | 85 |  |  |  |  |  |  |  |  |
| 12 | MALPY00871 | 7530 | - | 359130 | 366759 |  |  |  |  |  | 85 |  |  |  |  |  |  |  |  |
| 12 | Pb.c000201838.Contig1 | 1263 | - | 359309 | 360572 |  |  |  |  |  |  |  |  |  |  |  |  |  |  |
| 12 | Pb.c001904438.Contig1 | 2147 | - | 359848 | 362031 |  |  |  |  |  |  |  |  |  |  |  |  |  |  |
| 12 | Pc.c000602844.Contig1 | 6898 | - | 360303 | 367417 |  |  |  |  |  |  |  |  |  |  |  |  |  |  |
| 12 | Pc.c000303276.Contig1 | 620 | - | 360996 | 361643 |  |  |  |  |  |  |  |  |  |  |  |  |  |  |
| 12 | Pb.c002112747.Contig1 | 787 | - | 362400 | 363184 |  |  |  |  |  |  |  |  |  |  |  |  |  |  |
| 12 | Pb.c001904433.Contig1 | 3981 | - | 364457 | 368360 |  |  |  |  |  |  |  |  |  |  |  |  |  |  |
| 12 | MALPY00629 | 8270 | - | 365648 | 373840 |  |  |  |  |  | 85 | 400 | 1400 |  |  |  |  |  |  |
| 12 | Pc.c000602365.Contig2 | 3044 | - | 367422 | 370388 | cRMPc06026 | 38953 | 317 | 334887 | 373840 |  |  |  |  |  |  |  |  |  |
| 12 | MALPY00669 | 19002 | + | 374157 | 394387 |  |  |  |  |  |  | 400 |  |  |  |  |  |  |  |
| 12 | Pc.c001404835.Contig1 | 1190 | - | 375490 | 376680 |  |  |  |  |  |  |  |  |  |  |  |  |  |  |
| 12 | Pb.c000801784.Contig1 | 7547 | + | 376027 | 383960 |  |  |  |  |  |  |  |  |  |  |  |  |  |  |
| 12 | Pc.c000803245.Contig1 | 4520 | - | 376604 | 381387 |  |  |  |  |  |  |  |  |  |  |  |  |  |  |
| 12 | MALPY02988 | 651 | - | 378935 | 379613 |  |  |  |  |  |  |  |  |  |  |  |  |  |  |
| 12 | Pc.c000803221.Contig1 | 4903 | - | 382583 | 388048 |  |  |  |  |  |  |  |  |  |  |  |  |  |  |
| 12 | Pb.c001001804.Contig1 | 3439 | + | 384710 | 388143 |  |  |  |  |  |  |  |  |  |  |  |  |  |  |
| 12 | Pb.c000401746.Contig1 | 1170 | - | 392675 | 393902 |  |  |  |  |  |  |  |  |  |  |  |  |  |  |
| 12 | Pc.c001605000.Contig2 | 1872 | - | 393573 | 395583 |  |  |  |  |  |  |  |  |  |  |  |  |  |  |
| 12 | MALPY03468 | 561 | + | 394974 | 395532 | cRMPc06027 | 21426 | 2501 | 374157 | 395583 |  | 353 | 2300 |  |  |  |  |  |  |
| 12 | Pc.c000803849.Contig1 | 1985 | - | 398084 | 400201 |  |  |  |  |  |  |  |  |  |  |  |  |  |  |
| 12 | MALPY00320 | 5152 | - | 398320 | 403563 |  |  |  |  |  |  | 353 | 4000 |  |  |  |  |  |  |
| 12 | Pb.c001001453.Contig1 | 4325 | + | 399628 | 404067 |  |  |  |  |  |  |  |  |  |  |  |  |  |  |
| 12 | Pc.c000803258.Contig1 | 2796 | - | 400531 | 403501 | cRMPc06028 | 5983 | 1020 | 398084 | 404067 |  |  |  |  |  |  |  |  |  |
| 12 | Pb.c001202116.Contig1 | 2668 | + | 405087 | 407758 |  |  |  |  |  |  |  |  |  |  |  |  |  |  |
| 12 | Pc.c001707902.Contig1 | 1607 | + | 406646 | 408250 |  |  |  |  |  |  |  |  |  |  |  |  |  |  |
| 12 | MALPY01120 | 5079 | + | 407515 | 412438 |  |  |  |  |  |  | 353 |  |  |  |  |  |  |  |
| 12 | Pb.c001703153.Contig1 | 754 | + | 408227 | 409089 |  |  |  |  |  |  |  |  |  |  |  |  |  |  |
| 12 | Pc.c001003253.Contig1 | 3879 | + | 408487 | 412253 |  |  |  |  |  |  |  |  |  |  |  |  |  |  |
| 12 | PbContig207 | 3654 | + | 408976 | 412516 |  |  |  |  |  |  |  |  |  |  |  |  |  |  |
| 12 | MALPY03490 | 823 | + | 410128 | 410915 | cRMPc06029 | 7429 | 265 | 405087 | 412516 |  | 115 | 2200 |  |  |  |  |  |  |
| 12 | MALPY01435 | 6517 | - | 412781 | 418860 |  |  |  |  |  |  | 115 |  |  |  |  |  |  |  |
| 12 | Pb.c001601977.Contig1 | 6802 | - | 413834 | 420281 |  |  |  |  |  |  |  |  |  |  |  |  |  |  |
| 12 | Pc.c001304317.Contig1 | 3264 | + | 413877 | 417134 |  |  |  |  |  |  |  |  |  |  |  |  |  |  |
| 12 | Pc.c000603047.Contig1 | 2930 | + | 417418 | 420077 |  |  |  |  |  |  |  |  |  |  |  |  |  |  |
| 12 | MALPY01191 | 8422 | - | 419704 | 428062 |  |  |  |  |  |  | 257 | 3000 |  |  |  |  |  |  |
| 12 | Pb.c001101557.Contig1 | 6688 | - | 422515 | 429153 |  |  |  |  |  |  |  |  |  |  |  |  |  |  |
| 12 | Pc.c001002680.Contig1 | 5393 | - | 423372 | 428761 |  |  |  |  |  |  |  |  |  |  |  |  |  |  |
| 12 | Pc.c001003386.Contig1 | 2578 | - | 428371 | 431060 |  |  |  |  |  |  |  |  |  |  |  |  |  |  |
| 12 | MALPY02445 | 4766 | - | 430260 | 435026 |  |  |  |  |  |  | 257 |  |  |  |  |  |  |  |
| 12 | PbContig31 - BP | 12648 | - | 430905 | 444275 |  |  |  |  |  |  |  |  |  |  |  |  |  |  |
| 12 | Pc.c001204391.Contig1 | 1449 | - | 431328 | 432777 |  |  |  |  |  |  |  |  |  |  |  |  |  |  |
| i14 | MALPY00682 | 8468 | - | 434973 | 444187 |  |  |  |  |  | 230 |  |  |  |  |  |  |  |  |
| i14 | Pc.c001203823.Contig1 | 8464 | + | 437822 | 447220 |  |  |  |  |  |  |  |  |  |  |  |  |  |  |
| i14 | MALPY03437 | 795 | - | 439947 | 440742 |  |  |  |  |  |  |  |  |  |  |  |  |  |  |
| i14 | Pb.c001502891.Contig1 | 680 | + | 443789 | 444469 |  |  |  |  |  |  |  |  |  |  |  |  |  |  |
| i14 | MALPY01634 | 5137 | + | 444218 | 449367 |  |  |  |  |  | 230 |  |  |  |  |  |  |  |  |
| i14 | Pb.c001302103.Contig1 | 3508 | + | 444989 | 448491 |  |  |  |  |  |  |  |  |  |  |  |  |  |  |
| i14 | Pc.c000303503.Contig1 | 3829 | + | 447422 | 451542 |  |  |  |  |  |  |  |  |  |  |  |  |  |  |
| i14 | MALPY02776 | 3681 | + | 449641 | 453319 |  |  |  |  |  | 230 | 486 | 23000 |  |  |  |  |  |  |
| i14 | Pb.c001904464.Contig1 | 3060 | - | 450657 | 453717 | cRMPc06030 | 40936 | 10214 | 412781 | 453717 |  |  |  |  |  |  |  |  |  |
| i14 | MALPY01004 | 6382 | - | 463931 | 470324 |  |  |  |  |  | 18 |  |  |  |  |  |  |  |  |
| i14 | Pb.c001301780.Contig1 | 4651 | - | 464342 | 468984 |  |  |  |  |  |  |  |  |  |  |  |  |  |  |
| i14 | Pc.c001204356.Contig1 | 546 | + | 465148 | 465694 |  |  |  |  |  |  |  |  |  |  |  |  |  |  |
| i14 | MALPY03553 | 657 | - | 466513 | 467170 |  |  |  |  |  |  | 486 |  |  |  |  |  |  |  |
| i14 | Pb.c001301775.Contig1 | 1862 | - | 469954 | 471924 |  |  |  |  |  |  |  |  |  |  |  |  |  |  |
| i14 | MALPY03038 | 1610 | - | 470433 | 472133 |  |  |  |  |  | 18 |  |  |  |  |  |  |  |  |
| i14 | Pc.c001405329.Contig1 | 794 | - | 470575 | 471366 |  |  |  |  |  |  |  |  |  |  |  |  |  |  |
| i14 | Pc.c001405393.Contig1 | 1091 | - | 471512 | 472603 |  |  |  |  |  |  |  |  |  |  |  |  |  |  |
| i14 | Pb.c001301777.Contig1 | 6076 | - | 472089 | 478172 |  |  |  |  |  |  |  |  |  |  |  |  |  |  |
| i14 | MALPY02764 | 2180 | + | 472148 | 474328 |  |  |  |  |  | 18 | 421 | 1900 |  |  |  |  |  |  |
| i14 | Pc.c001606024.Contig1 | 1335 | - | 473390 | 474725 |  |  |  |  |  |  |  |  |  |  |  |  |  |  |
| i14 | MALPY00152 | 2674 | - | 474905 | 477591 |  |  |  |  |  |  | 421 | 1500 |  |  |  |  |  |  |
| i14 | Pc.c001203880.Contig1 | 2225 | - | 475384 | 477612 |  |  |  |  |  |  |  |  |  |  |  |  |  |  |
| i14 | MALPY01350 - BP | 3668 | + | 477879 | 481525 |  |  |  |  |  | 947 | 421 | 2000 |  |  |  |  |  |  |
| i14 | PbContig203 - BP | 7661 | + | 478673 | 486907 |  |  |  |  |  |  |  |  |  |  |  |  |  |  |
| 4 | MALPY01232 | 7524 | - | 481499 | 489234 |  |  |  |  |  | 947 | 314 | 400 |  |  |  |  |  |  |
| 4 | Pc.c001103066.Contig2 | 2695 | + | 482114 | 484770 |  |  |  |  |  |  |  |  |  |  |  |  |  |  |
| 4 | Pc.c000903907.Contig1 | 844 | + | 483986 | 484803 |  |  |  |  |  |  |  |  |  |  |  |  |  |  |
| 4 | Pc.c000903353.Contig1 | 2943 | + | 485053 | 488053 | cRMPc06031 | 25303 | 3778 | 463931 | 489234 |  |  |  |  |  |  |  |  |  |
| 4 | Pc.c001303491.Contig1 | 5266 | + | 493012 | 498335 |  |  |  |  |  |  |  |  |  |  |  |  |  |  |
| 4 | MALPY01510 | 13665 | + | 493737 | 510413 |  |  |  |  |  | 418 | 314 |  |  |  |  |  |  |  |
| 4 | Pb.c001602373.Contig1 | 5618 | + | 496174 | 502534 |  |  |  |  |  |  |  |  |  |  |  |  |  |  |
| 4 | PcContig346 | 3798 | - | 501871 | 505681 |  |  |  |  |  |  |  |  |  |  |  |  |  |  |
| 4 | Pb.c002007071.Contig1 | 1897 | + | 507353 | 509289 | cRMPc06032 | 17401 | 1306 | 493012 | 510413 |  |  |  |  |  |  |  |  |  |
| JC4 | MALPY01223 | 2564 | xxx | 510000 |  |  |  |  |  |  | 418 | 215A | 2500 |  |  |  |  |  |  |
| 4 | Pc.c000402279.Contig1 | 3285 | + | 511719 | 514851 |  |  |  |  |  |  |  |  |  |  |  |  |  |  |
| 4 | MALPY02996 | 1223 | - | 512945 | 514159 | cRMPc06033 | 3132 | 1338 | 511719 | 514851 |  |  |  |  |  |  |  |  |  |
| 4 | Pb.c001904362.Contig1 | 1521 | + | 516189 | 517710 |  |  |  |  |  |  |  |  |  |  |  |  |  |  |
| man4 | MALPY02618 | 3771 | - | 516985 | 522360 |  |  |  |  |  |  | 215A | 100 |  |  |  |  |  |  |
| 4 | Pb.c000501469.Contig1 | 6340 | - | 520048 | 526508 |  |  |  |  |  |  |  |  |  |  |  |  |  |  |
| man4 | Pb.c000501468.Contig1 | 2948 | + | 520122 | 523070 |  |  |  |  |  |  |  |  |  |  |  |  |  | MALPY02618 |
| 4 | Pc.c000502983.Contig1 | 9586 | + | 521493 | 531438 |  |  |  |  |  |  |  |  |  |  |  |  |  |  |
| man4 | MALPY02617 | 3952 | + | 522342 | 526294 |  |  |  |  |  |  |  |  |  |  |  |  |  | \| |
| man4 | PcContig4452 | 2229 | + | 525932 | 528161 |  |  |  |  |  |  |  |  |  |  |  |  |  | \| |
| man4 | MALPY02502 | 7970 | + | 526827 | 532189 |  |  |  |  |  |  |  |  |  |  |  |  |  | \| |
| man4 | PbContig5293 | 6502 | - | 529381 | 535883 |  |  |  |  |  |  |  |  |  |  |  |  |  | MALPY02040 |
| 4 | MALPY02040 | 3867 | - | 531831 | 535745 |  |  |  |  |  |  | 215A |  |  |  |  |  |  |  |
| 4 | MALPY03263 | 319 | + | 533816 | 534132 | cRMPc06034 | 19694 | 467 | 516189 | 535883 |  |  |  | cRMPs0612 | 226048 | 467 | 309835 | 535883 |  |
| 4 | MALPY01509 | 3178 | + | 536350 | 539471 |  |  |  |  |  | 131 |  |  |  |  |  |  |  |  |
| 4 | Pb.c000801674.Contig1 - BP | 21773 | - | 536485 | 560713 |  |  |  |  |  |  |  |  |  |  |  |  |  |  |
| 4 | MALPY01685 - BP | 10797 | + | 540656 | 551400 |  |  |  |  |  | 131 |  |  |  |  |  |  |  |  |
| 4 | PcContig274 | 4042 | + | 541258 | 545655 |  |  |  |  |  |  |  |  |  |  |  |  |  |  |
| 4 | Pc.c000502945.Contig1 | 1 - 2538 | + | 545936 | 548748 |  |  |  |  |  |  |  |  |  |  |  |  |  |  |
| 7 | Pc.c001304112.Contig1 | 11783 | - | 549995 | 563948 |  |  |  |  |  |  |  |  |  |  |  |  |  |  |
| 7 | MALPY00272 | 16836 | - | 551698 | 571384 |  |  |  |  |  | 131 |  |  |  |  |  |  |  |  |
| 7 | Pb.c000301362.Contig1 | 6259 | - | 564084 | 570900 |  |  |  |  |  |  |  |  |  |  |  |  |  |  |
| 7 | Pc.c001606760.Contig1 | 1128 | - | 565474 | 566644 |  |  |  |  |  |  |  |  |  |  |  |  |  |  |
| 7 | Pc.c001606601.Contig1 | 1046 | - | 568771 | 569817 |  |  |  |  |  |  |  |  |  |  |  |  |  |  |
| 7 | MALPY02034 | 5751 | + | 570920 | 576722 |  |  |  |  |  | 131 | 342 | 600 |  |  |  |  |  |  |
| 7 | Pb.c000201544.Contig1 | 2982 | + | 574216 | 577234 |  |  |  |  |  |  |  |  |  |  |  |  |  |  |
| 7 | Pc.c001505279.Contig1 | 3270 | + | 577152 | 580422 |  |  |  |  |  |  |  |  |  |  |  |  |  |  |
| 7 | MALPY00162 | 9202 | + | 577252 | 586874 |  |  |  |  |  | 336 | 342 |  |  |  |  |  |  |  |
| 7 | Pb.c001502457.Contig1 | 3888 | + | 578008 | 582087 |  |  |  |  |  |  |  |  |  |  |  |  |  |  |
| 7 | MALPY03568 | 823 | - | 580148 | 580968 |  |  |  |  |  |  | 298 | 6500 |  |  |  |  |  |  |
| 7 | PcContig338 | 3152 | + | 580511 | 583790 |  |  |  |  |  |  |  |  |  |  |  |  |  |  |
| 7 | Pc.c001003899.Contig1 | 637 | + | 584220 | 584860 |  |  |  |  |  |  |  |  |  |  |  |  |  |  |
| 7 | Pb.c000601212.Contig1 | 7833 | + | 586684 | 594927 |  |  |  |  |  |  |  |  |  |  |  |  |  |  |
| 7 | MALPY01315 | 7813 | + | 587289 | 595283 |  |  |  |  |  | 336 | 298 |  |  |  |  |  |  |  |
| 7 | Pc.c000202786.Contig1 | 2738 | - | 590014 | 592800 | cRMPc06035 | 58933 | 639 | 536350 | 595283 |  |  |  |  |  |  |  |  |  |
| 7 | MALPY00223 | 7439 | + | 595922 | 603161 |  |  |  |  |  | 336 | 148 | 500 |  |  |  |  |  |  |
| 7 | Pc.c000402718.Contig1 | 5385 | - | 596098 | 601794 |  |  |  |  |  |  |  |  |  |  |  |  |  |  |
| 7 | Pb.c001702925.Contig1 | 5002 | + | 596400 | 601605 |  |  |  |  |  |  |  |  |  |  |  |  |  |  |
| 7 | Pb.c001702923.Contig1 | 4238 | + | 601826 | 606237 |  |  |  |  |  |  |  |  |  |  |  |  |  |  |
| 7 | MALPY00939 | 20181 | - | 603306 | 624522 |  |  |  |  |  |  | 148 |  |  |  |  |  |  |  |
| 7 | Pc.c000603506.Contig1 | 1108 | + | 603580 | 604667 |  |  |  |  |  |  |  |  |  |  |  |  |  |  |
| 7 | Pc.c001708796.Contig1 | 1531 | + | 605075 | 606621 |  |  |  |  |  |  |  |  |  |  |  |  |  |  |
| 7 | Pb.c001202103.Contig1 | 4237 | + | 607528 | 611723 |  |  |  |  |  |  |  |  |  |  |  |  |  |  |
| 7 | Pb.c001803390.Contig1 | 858 | - | 612226 | 613024 |  |  |  |  |  |  |  |  |  |  |  |  |  |  |
| 7 | MALPY03442 | 702 | - | 612629 | 613301 |  |  |  |  |  |  | 57 | 500 |  |  |  |  |  |  |
| 7 | Pc.c001605830.Contig1 | 1977 | + | 612731 | 614717 |  |  |  |  |  |  |  |  |  |  |  |  |  |  |
| 7 | PbContig119 | 8350 | - | 613086 | 622356 |  |  |  |  |  |  |  |  |  |  |  |  |  |  |
| 7 | MALPY03016 | 564 | - | 613426 | 613954 |  |  |  |  |  |  | 57 |  |  |  |  |  |  |  |
| 7 | Pc.c001505087.Contig1 | 4806 | + | 616089 | 620895 |  |  |  |  |  |  |  |  |  |  |  |  |  |  |
| 7 | Pc.c001708946.Contig1 | 684 | + | 621737 | 622445 |  |  |  |  |  |  |  |  |  |  |  |  |  |  |
| 7 | Pc.c001203933.Contig1 | 2835 | + | 622635 | 625473 |  |  |  |  |  |  |  |  |  |  |  |  |  |  |
| 7 | Pb.c001602067.Contig1 | 6248 | - | 623013 | 629807 |  |  |  |  |  |  |  |  |  |  |  |  |  |  |
| 7 | MALPY03015 | 841 | - | 624820 | 625667 |  |  |  |  |  |  |  |  |  |  |  |  |  |  |
| 7 | Pc.c001203900.Contig1 | 1956 | + | 626022 | 628077 |  |  |  |  |  |  |  |  |  |  |  |  |  |  |
| 7 | MALPY00770 | 19136 | - | 626071 | 646738 |  |  |  |  |  | 456 |  |  |  |  |  |  |  |  |
| 7 | Pc.c001304638.Contig1 | 976 | + | 628071 | 629161 |  |  |  |  |  |  |  |  |  |  |  |  |  |  |
| 7 | Pb.c000501511.Contig1 | 6352 | - | 630224 | 637465 |  |  |  |  |  |  |  |  |  |  |  |  |  |  |
| 7 | MALPY03017 | 670 | + | 637279 | 637924 |  |  |  |  |  |  | 58 | 1500 |  |  |  |  |  |  |
| 7 | MALPY03335 | 348 | + | 639331 | 639679 |  |  |  |  |  |  | 58 | 5000 |  |  |  |  |  |  |
| 7 | Pb.c001502908.Contig1 | 578 | + | 640064 | 640642 |  |  |  |  |  |  |  |  |  |  |  |  |  |  |
| 7 | Pc.c001303444.Contig1 | 3026 | + | 641224 | 644220 |  |  |  |  |  |  |  |  |  |  |  |  |  |  |
| 7 | PbContig283 | 6735 | + | 641729 | 648473 |  |  |  |  |  |  |  |  |  |  |  |  |  |  |
| 7 | Pc.c001404839.Contig1 | 2613 | - | 644251 | 646903 |  |  |  |  |  |  |  |  |  |  |  |  |  |  |
| JC7 | MALPY03120 | 189 | + | 644501 | 644690 |  |  |  |  |  |  | 58 | 2000 |  |  |  |  |  |  |
| 7 | MALPY00846 | 5815 | - | 646785 | 652708 |  |  |  |  |  | 456 | 58 |  |  |  |  |  |  |  |
| 7 | Pc.c001812782.Contig1 | 643 | + | 647756 | 648498 | cRMPc06036 | 56786 | 5133 | 595922 | 652708 |  |  |  | cRMPs0613 | 116358 | 5133 | 536350 | 652708 |  |
| Centromere \* - gap 5133 bp | |  |  | 650000 |  |  |  |  |  |  |  |  |  |  |  |  |  |  |  |
| 7 | MALPY01919 | 4200 | - | 657841 | 662060 |  |  |  |  |  | 5 |  |  |  |  |  |  |  |  |
| 7 | Pb.c000501466.Contig1 | 2447 | - | 658108 | 660540 |  |  |  |  |  |  |  |  |  |  |  |  |  |  |
| 7 | Pb.c000501468.Contig1 | 3625 | - | 660470 | 664098 |  |  |  |  |  |  |  |  |  |  |  |  |  |  |
| 7 | Pc.c000402220.Contig2 | 3193 | - | 660500 | 663696 |  |  |  |  |  |  |  |  |  |  |  |  |  |  |
| 7 | MALPY01668 | 11026 | - | 661325 | 672357 |  |  |  |  |  | 5 | 207 | 1900 |  |  |  |  |  |  |
| 7 | Pb.c000601519.Contig1 | 2920 | - | 664317 | 667210 |  |  |  |  |  |  |  |  |  |  |  |  |  |  |
| 7 | Pc.c000803454.Contig1 | 3518 | + | 667063 | 670686 |  |  |  |  |  |  |  |  |  |  |  |  |  |  |
| 7 | Pb.c000601516.Contig1 | 2341 | - | 667614 | 670047 | cRMPc06037 | 14516 | 1008 | 657841 | 672357 |  |  |  |  |  |  |  |  |  |
| man7 | MALPY01925 - Ins | 13789 | - | 673365 | 686232 |  |  |  |  |  | 239 | 207 |  |  |  |  |  |  |  |
| 7 | Pc.c001204118.Contig1 | 5479 | - | 674944 | 680372 |  |  |  |  |  |  |  |  |  |  |  |  |  |  |
| 7 | Pb.c000401482.Contig1 | 2866 | + | 678955 | 681787 |  |  |  |  |  |  |  |  |  |  |  |  |  |  |
| 7 | MALPY03278 | 333 | - | 680428 | 680761 |  |  |  |  |  |  | 217 | 4000 |  |  |  |  |  |  |
| 7 | PbContig50 | 5670 | + | 682653 | 688230 |  |  |  |  |  |  |  |  |  |  |  |  |  |  |
| 7 | Pc.c000803281.Contig1 | 2627 | + | 683467 | 685810 |  |  |  |  |  |  |  |  |  |  |  |  |  |  |
| 7 | MALPY00676 | 23844 | + | 686299 | 713390 |  |  |  |  |  | 239 | 217 |  |  |  |  |  |  |  |
| 7 | MALPY03189 | 421 | + | 686578 | 686996 |  |  |  |  |  |  | 160 | 3000 |  |  |  |  |  |  |
| 7 | Pb.c001101868.Contig1 | 2223 | + | 688483 | 690799 |  |  |  |  |  |  |  |  |  |  |  |  |  |  |
| 7 | MALPY03079 | 329 | - | 690152 | 690481 |  |  |  |  |  |  | 160 |  |  |  |  |  |  |  |
| 7 | PcContig372 | 2561 | - | 698872 | 701574 |  |  |  |  |  |  |  |  |  |  |  |  |  |  |
| 7 | Pb.c001602570.Contig1 | 1756 | + | 698979 | 700729 |  |  |  |  |  |  |  |  |  |  |  |  |  |  |
| 7 | PbContig359 | 11613 | + | 703381 | 716052 |  |  |  |  |  |  |  |  |  |  |  |  |  |  |
| 7 | Pc.c000302845.Contig1 | 2356 | + | 703412 | 705768 |  |  |  |  |  |  |  |  |  |  |  |  |  |  |
| 7 | Pc.c000904003.Contig1 | 1329 | + | 706598 | 707978 |  |  |  |  |  |  |  |  |  |  |  |  |  |  |
| 7 | PcContig229 | 10417 | + | 708777 | 721125 |  |  |  |  |  |  |  |  |  |  |  |  |  |  |
| 7 | MALPY03203 | 435 | - | 709429 | 709861 |  |  |  |  |  |  | 128 | 2400 |  |  |  |  |  |  |
| 7 | MALPY00093 | 11700 | + | 711817 | 724535 |  |  |  |  |  |  | 128 | 900 |  |  |  |  |  |  |
| 7 | Pb.c001803442.Contig1 | 4339 | + | 719703 | 723794 |  |  |  |  |  |  |  |  |  |  |  |  |  |  |
| 7 | Pc.c001606146.Contig1 | 2714 | - | 721532 | 724249 |  |  |  |  |  |  |  |  |  |  |  |  |  |  |
| 7 | Pb.c001101809.Contig1 | 6372 | + | 724032 | 731156 |  |  |  |  |  |  |  |  |  |  |  |  |  |  |
| 7 | MALPY00379 | 7382 | + | 724549 | 732424 |  |  |  |  |  | 32 | 128 |  |  |  |  |  |  |  |
| 7 | Pc.c001706594.Contig1 | 2425 | - | 726850 | 729263 |  |  |  |  |  |  |  |  |  |  |  |  |  |  |
| 7 | Pc.c001505106.Contig1 | 2003 | - | 729604 | 731586 |  |  |  |  |  |  |  |  |  |  |  |  |  |  |
| 7 | MALPY03481 | 784 | + | 729707 | 730491 |  |  |  |  |  |  | 165 | 2500 |  |  |  |  |  |  |
| 7 | Pb.c001101849.Contig1 | 4006 | + | 731655 | 735611 |  |  |  |  |  |  |  |  |  |  |  |  |  |  |
| 7 | Pc.c001707746.Contig1 | 1918 | - | 732179 | 734093 |  |  |  |  |  |  |  |  |  |  |  |  |  |  |
| 7 | MALPY03223 | 1253 | + | 732329 | 733582 |  |  |  |  |  | 32 | 165 |  |  |  |  |  |  |  |
| 7 | MALPY03315 | 726 | + | 733039 | 733765 |  |  |  |  |  |  | 115 | 500 |  |  |  |  |  |  |
| 7 | MALPY03055 | 875 | + | 733652 | 734512 | cRMPc06038 | 62246 | 561 | 673365 | 735611 |  | 115 |  | cRMPs0614 | 77770 | 561 | 657841 | 735611 |  |
| 7 | Pb.c001101903.Contig1 | 4750 | + | 736172 | 740709 |  |  |  |  |  |  |  |  |  |  |  |  |  |  |
| 7 | MALPY03275 | 1981 | + | 736260 | 738241 |  |  |  |  |  |  |  |  |  |  |  |  |  |  |
| 7 | Pc.c000502909.Contig1 | 6014 | - | 737877 | 743894 |  |  |  |  |  |  |  |  |  |  |  |  |  |  |
| 7 | MALPY00468 | 24324 | + | 738008 | 765457 |  |  |  |  |  |  | 121 | 1000 |  |  |  |  |  |  |
| 7 | PbContig248 | 7671 | + | 742361 | 750028 |  |  |  |  |  |  |  |  |  |  |  |  |  |  |
| 7 | Pc.c000602385.Contig1 | 10106 | + | 744934 | 755058 |  |  |  |  |  |  |  |  |  |  |  |  |  |  |
| 7 | Pb.c001602468.Contig1 | 2062 | - | 761055 | 763126 |  |  |  |  |  |  |  |  |  |  |  |  |  |  |
| 7 | Pc.c001404850.Contig1 | 2502 | - | 761056 | 763567 | cRMPc06039 | 29285 | 825 | 736172 | 765457 |  |  |  |  |  |  |  |  |  |
| 7 | MALPY01457 | 8758 | + | 766282 | 775109 |  |  |  |  |  | 704 | 121 |  |  |  |  |  |  |  |
| 7 | Pc.c000002078.Contig1 | 4684 | - | 767844 | 772724 |  |  |  |  |  |  |  |  |  |  |  |  |  |  |
| 7 | Pb.c001904643.Contig1 | 3430 | + | 774947 | 778300 |  |  |  |  |  |  |  |  |  |  |  |  |  |  |
| 7 | MALPY02023 | 2752 | + | 775700 | 778366 |  |  |  |  |  | 704 |  |  |  |  |  |  |  |  |
| 7 | Pc.c000603248.Contig1 | 2985 | - | 776346 | 779283 |  |  |  |  |  |  |  |  |  |  |  |  |  |  |
| 7 | MALPY00796 | 10107 | - | 779016 | 789755 |  |  |  |  |  | 704 | 149 | 450 |  |  |  |  |  |  |
| 7 | Pb.c001602520.Contig1 | 6421 | - | 779978 | 786298 |  |  |  |  |  |  |  |  |  |  |  |  |  |  |
| 7 | Pc.c001304197.Contig1 | 3067 | + | 781727 | 784791 |  |  |  |  |  |  |  |  |  |  |  |  |  |  |
| 7 | Pb.c000901752.Contig1 | 2587 | - | 786990 | 789577 |  |  |  |  |  |  |  |  |  |  |  |  |  |  |
| 7 | PcContig205 | 2825 | + | 789067 | 791892 |  |  |  |  |  |  |  |  |  |  |  |  |  |  |
| 7 | Pb.c002007045.Contig1 | 4077 | + | 789664 | 794574 |  |  |  |  |  |  |  |  |  |  |  |  |  |  |
| 7 | MALPY03463 | 703 | + | 790024 | 790745 |  |  |  |  |  |  | 149 |  |  |  |  |  |  |  |
| 7 | MALPY02699 | 2409 | - | 791255 | 793649 |  |  |  |  |  | 25 |  |  |  |  |  |  |  |  |
| 7 | MALPY02700 | 2145 | - | 792718 | 794848 | cRMPc06040 | 28566 | 2845 | 766282 | 794848 | 25 |  |  |  |  |  |  |  |  |
| 7 | Pb.c000301367.Contig1 | 4714 | + | 797693 | 802419 |  |  |  |  |  |  |  |  |  |  |  |  |  |  |
| 7 | MALPY00622 | 8530 | + | 797866 | 806259 |  |  |  |  |  | 25 |  |  |  |  |  |  |  |  |
| 7 | Pc.c001304568.Contig1 | 3947 | + | 800334 | 804375 |  |  |  |  |  |  |  |  |  |  |  |  |  |  |
| 7 | Pb.c000301366.Contig1 | 1815 | + | 802598 | 804377 |  |  |  |  |  |  |  |  |  |  |  |  |  |  |
| 7 | PcContig41 | 2589 | + | 804204 | 806790 |  |  |  |  |  |  |  |  |  |  |  |  |  |  |
| 7 | PbContig48 | 8974 | + | 804319 | 813866 |  |  |  |  |  |  |  |  |  |  |  |  |  |  |
| 7 | MALPY00565 | 13080 | - | 807061 | 820296 |  |  |  |  |  | 25 |  |  |  |  |  |  |  |  |
| 7 | Pc.c001505128.Contig1 | 2158 | + | 807613 | 809801 |  |  |  |  |  |  |  |  |  |  |  |  |  |  |
| 7 | Pb.c001602436.Contig1 | 5061 | + | 814528 | 819832 |  |  |  |  |  |  |  |  |  |  |  |  |  |  |
| 7 | Pc.c000402895.Contig1 | 7980 | - | 818714 | 827611 |  |  |  |  |  |  |  |  |  |  |  |  |  |  |
| 7 | MALPY00564 | 8532 | - | 820383 | 829068 |  |  |  |  |  | 25 |  |  |  |  |  |  |  |  |
| 7 | Pb.c000801743.Contig1 | 3812 | + | 821657 | 825622 |  |  |  |  |  |  |  |  |  |  |  |  |  |  |
| 7 | Pb.c000401146.Contig1 | 12319 | + | 828062 | 841744 |  |  |  |  |  |  |  |  |  |  |  |  |  |  |
| 7 | MALPY03230 | 1108 | - | 830362 | 831467 |  |  |  |  |  | 25 |  |  |  |  |  |  |  |  |
| 7 | Pc.c000003123.Contig1 | 11378 | - | 830545 | 844793 |  |  |  |  |  |  |  |  |  |  |  |  |  |  |
| 7 | MALPY01762 | 5967 | - | 831701 | 837677 |  |  |  |  |  | 25 |  |  |  |  |  |  |  |  |
| 7 | MALPY00012 | 13188 | + | 838781 | 853205 |  |  |  |  |  | 25 |  |  |  |  |  |  |  |  |
| 7 | Pb.c001904646.Contig1 | 7666 | + | 842803 | 850469 |  |  |  |  |  |  |  |  |  |  |  |  |  |  |
| 7 | PcContig719 | 5803 | - | 845481 | 851317 |  |  |  |  |  |  |  |  |  |  |  |  |  |  |
| 7 | PcContig215 | 6758 | - | 851172 | 857915 | cRMPc06041 | 60222 | 6101 | 797693 | 857915 |  |  |  | cRMPs0615 | 121743 | 6101 | 736172 | 857915 |  |
| 7 | MALPY00436 | 15184 | + | 864016 | 878603 |  |  |  |  |  | 188 |  |  |  |  |  |  |  |  |
| 7 | Pb.c001904267.Contig1 | 7480 | + | 867183 | 874340 |  |  |  |  |  |  |  |  |  |  |  |  |  |  |
| 7 | Pc.c000102492.Contig1 | 1987 | + | 867436 | 869426 |  |  |  |  |  |  |  |  |  |  |  |  |  |  |
| 7 | PcContig380 | 4067 | + | 869154 | 873086 |  |  |  |  |  |  |  |  |  |  |  |  |  |  |
| 7 | Pc.c001204085.Contig1 | 1248 | + | 872655 | 873888 | cRMPc06042 |  |  |  |  |  |  |  |  |  |  |  |  |  |
| 7 | Pc.c000403108.Contig1 | 623 | + | 872655 | 873263 |  | 14587 | 441 | 864016 | 878603 |  |  |  |  |  |  |  |  |  |
| 7 | MALPY00960 | 4609 | + | 879044 | 883653 |  |  |  |  |  | 188 |  |  |  |  |  |  |  |  |
| 7 | Pb.c001402065.Contig1 | 2219 | + | 879898 | 882117 |  |  |  |  |  |  |  |  |  |  |  |  |  |  |
| 7 | PcContig661 | 4411 | + | 882142 | 887054 |  |  |  |  |  |  |  |  |  |  |  |  |  |  |
| 7 | Pb.c001101879.Contig1 | 3258 | + | 882916 | 886639 |  |  |  |  |  |  |  |  |  |  |  |  |  |  |
| 7 | MALPY00959 | 6062 | + | 883807 | 890637 |  |  |  |  |  | 188 |  |  |  |  |  |  |  |  |
| 7 | PbContig382 | 7081 | + | 887167 | 894993 |  |  |  |  |  |  |  |  |  |  |  |  |  |  |
| 7 | MALPY02863 | 4746 | - | 891256 | 895935 |  |  |  |  |  | 188 | 249 | 1800 |  |  |  |  |  |  |
| 7 | PcContig778 | 10685 | - | 892248 | 903633 |  |  |  |  |  |  |  |  |  |  |  |  |  |  |
| 7 | Pb.c000801824.Contig1 | 6777 | + | 895917 | 902763 |  |  |  |  |  |  |  |  |  |  |  |  |  |  |
| 7 | MALPY00237 | 9152 | - | 896927 | 905355 |  |  |  |  |  |  | 249 |  |  |  |  |  |  |  |
| 7 | PbContig21 | 4795 | - | 903562 | 909767 |  |  |  |  |  |  |  |  |  |  |  |  |  |  |
| 7 | PcContig332 | 2773 | + | 905484 | 908251 |  |  |  |  |  |  |  |  |  |  |  |  |  |  |
| 7 | MALPY03402 | 886 | + | 907670 | 908556 | cRMPc06043 | 30723 | 160 | 879044 | 909767 |  | 198 | 400 |  |  |  |  |  |  |
| 7 | MALPY01080 | 13205 | + | 909927 | 922859 |  |  |  |  |  |  | 198 |  |  |  |  |  |  |  |
| 7 | Pb.c001602099.Contig1 | 2898 | - | 914922 | 917744 |  |  |  |  |  |  |  |  |  |  |  |  |  |  |
| 7 | Pc.c000202751.Contig1 | 6131 | + | 915726 | 921902 |  |  |  |  |  |  |  |  |  |  |  |  |  |  |
| 7 | Pb.c000901431.Contig1 | 7410 | + | 916560 | 924126 |  |  |  |  |  |  |  |  |  |  |  |  |  |  |
| 7 | MALPY02297 | 2294 | - | 923846 | 926443 | cRMPc06044 | 16516 | 500 | 909927 | 926443 |  | new/new2 - NS | 500/2000 |  |  |  |  |  |  |
| man7 | MALPY02200 | 2988 | - | 926943 | 929255 |  |  |  |  |  |  | new2 - NS |  |  |  |  |  |  |  |
| man7 | MALPY01613 | 4828 | - | 928370 | 932364 |  |  |  |  |  | 442 | new - NS |  |  |  |  |  |  |  |
| man7 | MALPY03090 | 1515 | - | 931963 | 933478 | cRMPc06045 | 6535 |  | 926943 | 933478 | 442 |  |  | cRMPs0616 | 69462 |  | 864016 | 933478 |  |
| Telomere | |  |  |  |  |  |  |  |  |  |  |  |  |  |  |  |  |  |  |
|  |  | Pb Pc Py contig |  |  |  |  | cRMP contig | Gap to next cRMP contig |  |  |  |  |  |  | cRMP scaffold | Gap to next cRMP scaffold |  |  |  |
|  | Number | 378 |  |  |  | Number | 45 | 44 |  |  |  |  |  | Number | 16 | 15 |  |  |  |
|  | Average size (bp) | 4592 |  |  |  | Average size (bp) | 19102 | 1679 |  |  |  |  |  | Average size (bp) | 56517 | 1948 |  |  |  |
|  | Minimum size (bp) | 189 |  |  |  | Minimum size (bp) | 1178 | 2 |  |  |  |  |  | Minimum size (bp) | 2727 | 95 |  |  |  |
|  | Maximum size (bp) | 24324 |  |  |  | Maximum size (bp) | 62246 | 10214 |  |  |  |  |  | Maximum size (bp) | 226048 | 6101 |  |  |  |
|  | Total size (bp) | 1735865 |  |  |  | Total size (bp) | 859602 | 73876 |  |  |  |  |  | Total size (bp) | 904265 | 29213 |  |  |  |
|  |  |  |  |  |  |  |  |  |  |  |  |  |  |  |  |  |  |  |  |
