# Supplementary material for: A Plasmodium Whole-Genome Synteny Map: Indels and Synteny Breakpoints as Foci for Species-Specific Genes
Source: PLoS Pathog. 2005 Dec 23;1(4):e44. doi: 10.1371/journal.ppat.0010044 (PMC1317653; doi:10.1371/journal.ppat.0010044)
Supplement: Supplementary file 7 [file ppat.0010044.st023.doc]

Table S23 - RMPchr7


# Table S23 - RMPchr7

|  |  |  |  |  |  |  |  |  |  |  |  |  |  |  |  |  |  |  |  |
| --- | --- | --- | --- | --- | --- | --- | --- | --- | --- | --- | --- | --- | --- | --- | --- | --- | --- | --- | --- |
| Table S23: Tiling path of RMP contigs combined to form cRMP chromosome 7. | | | | | | |  |  |  |  |  |  |  |  |  |  |  |  |  |
|  |  |  |  |  |  |  |  |  |  |  |  |  |  |  |  |  |  |  |  |
| Tiling paths of P. berghei (Pb), P. c. chabaudi (Pc) and P. y. yoelii (Py) contigs combined into composite rodent malaria parasite (cRMP) | | | | | | | | | |  |  |  |  |  |  |  |  |  |  |
| contigs aligned against parts of the 14 chromosomes of P. falciparum (Pf). The cRMP contigs are further linked by physical linkage | | | | | | | | | |  |  |  |  |  |  |  |  |  |  |
| and PCR (see Methods) to form cRMP scaffolds. Different synteny blocks with homology to different P. falciparum chromosomes | | | | | | | | | |  |  |  |  |  |  |  |  |  |  |
| together form the cRMP chromosomes. The tables also show gaps in the alignment (black lines), cRMP telomeres (white bars) and | | | | | | | | | |  |  |  |  |  |  |  |  |  |  |
| predicted centromeres (plum/black bars). Contigs, physical groups or PCR groups interrupted by inter- or intrasyntenic regions are | | | | | | | | | |  |  |  |  |  |  |  |  |  |  |
| printed in bold. A summary of the number of RMP contigs, cRMP contigs and cRMP scaffolds as well as their average, minimum, | | | | | | | | | |  |  |  |  |  |  |  |  |  |  |
| maximum and total sizes is provided. Column headings are as follows: | | | | |  |  |  |  |  |  |  |  |  |  |  |  |  |  |  |
|  |  |  |  |  |  |  |  |  |  |  |  |  |  |  |  |  |  |  |  |
| Pf chr.: P. falciparum chromosome to which the RMP contigs are aligned | | | | | |  |  |  |  |  |  |  |  |  |  |  |  |  |  |
| (#: PROmer analysis� from this study; JC# and TK#: Carlton et al.; man#: manual BLAST analysis; | | | | | | |  |  |  |  |  |  |  |  |  |  |  |  |  |
| i: inversed orientation of the synteny block; colours refer to those used in Fig. 2 of the paper); | | | | | | |  |  |  |  |  |  |  |  |  |  |  |  |  |
| Pb Pc Py contig: names of the aligned RMP contigs; | | |  |  |  |  |  |  |  |  |  |  |  |  |  |  |  |  |  |
| Size: size (bp) of the aligned RMP contigs; | |  |  |  |  |  |  |  |  |  |  |  |  |  |  |  |  |  |  |
| +/-: orientation of the aligned RMP contigs; | |  |  |  |  |  |  |  |  |  |  |  |  |  |  |  |  |  |  |
| Start contig & End contig: estimated start & end coordinates of alignment of the RMP contigs based on the coordinates | | | | | | | | |  |  |  |  |  |  |  |  |  |  |  |
| of PROmer hits corrected for the contig�s size and orientation and presence of inter- and intrasyntenic regions | | | | | | | |  |  |  |  |  |  |  |  |  |  |  |  |
| (the start position of the first RMP contig in the alignment was set at zero); | | | | | |  |  |  |  |  |  |  |  |  |  |  |  |  |  |
| cRMP contig: names of cRMP contigs | |  |  |  |  |  |  |  |  |  |  |  |  |  |  |  |  |  |  |
| (the first 2 digits refer to the cRMP chromosome number and the following 3 digits designate the order of the contigs); | | | | | | | | |  |  |  |  |  |  |  |  |  |  |  |
| cRMP contig size: size of cRMP contigs based on the corrected coordinates of alignment; | | | | | | |  |  |  |  |  |  |  |  |  |  |  |  |  |
| Gap size to next cRMP contig: size of gaps between neighbouring cRMP contigs; | | | | | | |  |  |  |  |  |  |  |  |  |  |  |  |  |
| Start cRMP contig & End cRMP contig: estimated start & end coordinates of alignment of the cRMP contigs; | | | | | | | |  |  |  |  |  |  |  |  |  |  |  |  |
| Phys. group (Py): physical linkage of neighbouring P. y. yoelii contigs; | | | | | |  |  |  |  |  |  |  |  |  |  |  |  |  |  |
| PCR group (Py): neighbouring P. y. yoelii contigs were linked previously or for this study by PCR; | | | | | | |  |  |  |  |  |  |  |  |  |  |  |  |  |
| PCR size (Py): size of the PCR product; | |  |  |  |  |  |  |  |  |  |  |  |  |  |  |  |  |  |  |
| cRMP scaffold: names of cRMP scaffolds | |  |  |  |  |  |  |  |  |  |  |  |  |  |  |  |  |  |  |
| (the first 2 digits refer to the cRMP chromosome number and the following 2 digits designate the order of the scaffolds); | | | | | | | | |  |  |  |  |  |  |  |  |  |  |  |
| cRMP scaffold size: size of cRMP scaffolds; | | |  |  |  |  |  |  |  |  |  |  |  |  |  |  |  |  |  |
| Gap size to next cRMP scaffold: size of gaps between neighbouring cRMP scaffolds; | | | | | | |  |  |  |  |  |  |  |  |  |  |  |  |  |
| Start cRMP scaffold & End cRMP scaffold: estimated start & end coordinates of alignment of the cRMP scaffolds; | | | | | | | | |  |  |  |  |  |  |  |  |  |  |  |
| Overlap: Linkage between RMP contigs established through manual BLAST analysis. | | | | | | |  |  |  |  |  |  |  |  |  |  |  |  |  |
|  |  |  |  |  |  |  |  |  |  |  |  |  |  |  |  |  |  |  |  |
| Pf chr. | Pb Pc Py contig | Size | +/- | Start contig | End contig | cRMP contig | cRMP contig size | Gap size to next cRMP contig | Start cRMP contig | End cRMP contig | Phys. group (Py) | PCR group (Py) | PCR size (Py) | cRMP scaffold | cRMP scaffold size | Gap size to next cRMP scaffold | Start cRMP scaffold | End cRMP scaffold | Overlap (manual) |
| Telomere | |  |  |  |  |  |  |  |  |  |  |  |  |  |  |  |  |  |  |
| JC8 | MALPY03070 | 1887 | xxx |  |  |  |  |  |  |  | 136 |  |  |  |  |  |  |  |  |
| JC8 | MALPY01484 | 3414 | xxx |  |  |  |  |  |  |  | 136 |  |  |  |  |  |  |  |  |
| JC8 | MALPY02830 | 5034 | xxx |  |  |  |  |  |  |  | 136 |  |  |  |  |  |  |  |  |
| JC8 | MALPY02831 | 3055 | xxx |  |  |  |  |  |  |  | 136 |  |  |  |  |  |  |  |  |
| JC8 | MALPY02653 | 2406 | xxx | 0 | 2406 | cRMPc07001 | 2406 | 500 | 0 | 2406 | 401 |  |  |  |  |  |  |  |  |
| 8 | MALPY02241 | 8298 | + | 2906 | 11071 | cRMPc07002 | 8165 | 22876 | 2906 | 11071 | 401 |  |  | RMPs0701 | 11071 | 22876 | 0 | 11071 |  |
| 8 | MALPY02250 | 3829 | + | 33947 | 37782 |  |  |  |  |  |  | 111 | 900 |  |  |  |  |  |  |
| man8 | PbContig5947 | 1316 | + | 34267 | 35583 |  |  |  |  |  |  |  |  |  |  |  |  |  | MALPY02250 |
| 8 | Pb.c001502845.Contig1 | 1181 | + | 35553 | 36719 |  |  |  |  |  |  |  |  |  |  |  |  |  |  |
| 8 | Pc.c001304294.Contig1 | 2509 | + | 36067 | 38215 |  |  |  |  |  |  |  |  |  |  |  |  |  |  |
| 8 | MALPY03585 | 646 | + | 37885 | 38531 |  |  |  |  |  |  | 111 | 800 |  |  |  |  |  |  |
| 8 | Pb.c000701483.Contig1 | 5067 | - | 38499 | 43234 |  |  |  |  |  |  |  |  |  |  |  |  |  |  |
| 8 | MALPY02571 | 5967 | - | 38772 | 44137 |  |  |  |  |  |  | 111 |  |  |  |  |  |  |  |
| 8 | Pc.c000303022.Contig1 | 2077 | - | 38778 | 40864 |  |  |  |  |  |  |  |  |  |  |  |  |  |  |
| 8 | Pc.c000202657.Contig1 | 2968 | - | 40657 | 43625 | cRMPc07003 | 10190 | 574 | 33947 | 44137 |  |  |  | RMPs0702 | 10190 | 574 | 33947 | 44137 |  |
| man8 | MALPY00210 | 2489 | + | 44711 | 47200 |  |  |  |  |  | 563 |  |  |  |  |  |  |  | MALPY02093 |
| 8 | Pb.c001101967.Contig1 | 4115 | + | 46082 | 50203 |  |  |  |  |  |  |  |  |  |  |  |  |  |  |
| 8 | MALPY02093 | 3808 | + | 46654 | 50444 |  |  |  |  |  |  | 54 | 2000 |  |  |  |  |  |  |
| 8 | Pc.c001405395.Contig1 | 1196 | - | 48066 | 49247 | cRMPc07004 | 5733 | 1902 | 44711 | 50444 |  |  |  |  |  |  |  |  |  |
| 8 | MALPY03014 | 1748 | - | 52346 | 54114 | cRMPc07005 | 1768 | 1553 | 52346 | 54114 | 563 | 54 | 1600 |  |  |  |  |  |  |
| 8 | MALPY02551 | 4231 | + | 55667 | 59880 | cRMPc07006 | 4213 | 1080 | 55667 | 59880 |  | 54 | 2300 |  |  |  |  |  |  |
| 8 | MALPY02675 | 4412 | - | 60960 | 65378 |  |  |  |  |  | 1257 | 54 |  |  |  |  |  |  |  |
| 8 | PcContig541 | 7793 | - | 62467 | 71082 |  |  |  |  |  |  |  |  |  |  |  |  |  |  |
| 8 | Pb.c001502044.Contig1 | 5478 | + | 63453 | 69746 |  |  |  |  |  |  |  |  |  |  |  |  |  |  |
| 8 | MALPY02701 | 2385 | + | 65883 | 68393 |  |  |  |  |  | 1257 | 269 | 1500 |  |  |  |  |  |  |
| 8 | MALPY01889 | 8856 | - | 69423 | 78792 |  |  |  |  |  |  | 269 | 2500 |  |  |  |  |  |  |
| 8 | Pb.c002005822.Contig1 | 4033 | + | 72577 | 77172 |  |  |  |  |  |  |  |  |  |  |  |  |  |  |
| 8 | Pc.c001103728.Contig1 | 2198 | + | 72945 | 75334 |  |  |  |  |  |  |  |  |  |  |  |  |  |  |
| 8 | Pc.c000903326.Contig1 | 5173 | + | 75786 | 81370 |  |  |  |  |  |  |  |  |  |  |  |  |  |  |
| 8 | Pb.c001102217.Contig1 | 1729 | - | 78116 | 79848 |  |  |  |  |  |  |  |  |  |  |  |  |  |  |
| 8 | PbContig302 | 4194 | - | 80560 | 84689 |  |  |  |  |  |  |  |  |  |  |  |  |  |  |
| 8 | MALPY00359 | 2755 | + | 82184 | 84871 |  |  |  |  |  |  | 269 | 1400 |  |  |  |  |  |  |
| 8 | Pc.c001707893.Contig1 | 1762 | + | 82774 | 84465 |  |  |  |  |  |  |  |  |  |  |  |  |  |  |
| 8 | Pc.c001103776.Contig1 | 2449 | + | 84791 | 87268 |  |  |  |  |  |  |  |  |  |  |  |  |  |  |
| 8 | MALPY00174 | 10644 | - | 85635 | 95988 |  |  |  |  |  | 968 | 269 | 2600 |  |  |  |  |  |  |
| 8 | Pb.c001602406.Contig1 | 4959 | + | 85692 | 90711 |  |  |  |  |  |  |  |  |  |  |  |  |  |  |
| 8 | Pc.c000203261.Contig1 | 1345 | + | 87481 | 88826 |  |  |  |  |  |  |  |  |  |  |  |  |  |  |
| 8 | Pc.c000002580.Contig1 | 2443 | + | 88743 | 91363 |  |  |  |  |  |  |  |  |  |  |  |  |  |  |
| 8 | Pb.c001904817.Contig1 | 703 | + | 90828 | 91534 | cRMPc07007 | 35028 | 2229 | 60960 | 95988 |  |  |  |  |  |  |  |  |  |
| JC8 | MALPY01086 | 2994 | xxx | 95000 |  |  |  |  |  |  | 968 |  |  |  |  |  |  |  |  |
| 8 | Pc.c001405520.Contig1 | 619 | - | 98217 | 98863 | cRMPc07008 | 646 | 869 | 98217 | 98863 |  |  |  |  |  |  |  |  |  |
| 8 | MALPY01352 | 8736 | - | 99732 | 109561 |  |  |  |  |  | 514 | 269 |  |  |  |  |  |  |  |
| 8 | Pc.c000102627.Contig1 | 6164 | - | 100700 | 107824 |  |  |  |  |  |  |  |  |  |  |  |  |  |  |
| 8 | Pb.c000901882.Contig1 | 6774 | + | 101500 | 109084 |  |  |  |  |  |  |  |  |  |  |  |  |  |  |
| 8 | Pc.c001505107.Contig1 | 954 | - | 107856 | 108810 | cRMPc07009 | 9829 | 1728 | 99732 | 109561 |  |  |  |  |  |  |  |  |  |
| 8 | MALPY02075 | 11059 | - | 111289 | 122879 |  |  |  |  |  | 514 |  |  |  |  |  |  |  |  |
| 8 | PcContig500 | 8172 | + | 117692 | 126799 |  |  |  |  |  |  |  |  |  |  |  |  |  |  |
| 8 | MALPY01046 | 7982 | - | 123031 | 131004 |  |  |  |  |  | 514 | 262 | 2100 |  |  |  |  |  |  |
| 8 | PbContig144 | 3988 | + | 123757 | 127695 |  |  |  |  |  |  |  |  |  |  |  |  |  |  |
| 8 | Pc.c000302906.Contig1 | 618 | + | 124426 | 125035 |  |  |  |  |  |  |  |  |  |  |  |  |  |  |
| 8 | Pb.c000001803.Contig1 | 4157 | + | 130106 | 134262 |  |  |  |  |  |  |  |  |  |  |  |  |  |  |
| 8 | PcContig316 | 12243 | - | 131765 | 145695 |  |  |  |  |  |  |  |  |  |  |  |  |  |  |
| 8 | MALPY03180 | 817 | + | 132874 | 133691 |  |  |  |  |  |  | 262 | 500 |  |  |  |  |  |  |
| 8 | MALPY00145 | 7572 | + | 133727 | 142704 |  |  |  |  |  |  | 262 | 1500 |  |  |  |  |  |  |
| 8 | Pb.c001101876.Contig1 | 4666 | + | 138175 | 143172 |  |  |  |  |  |  |  |  |  |  |  |  |  |  |
| 8 | MALPY01790 | 8009 | - | 144166 | 152559 |  |  |  |  |  |  | 262 | 2400 |  |  |  |  |  |  |
| 8 | Pb.c001502559.Contig1 | 2718 | - | 144284 | 146958 |  |  |  |  |  |  |  |  |  |  |  |  |  |  |
| 8 | Pc.c000503124.Contig1 | 1638 | - | 146178 | 147822 |  |  |  |  |  |  |  |  |  |  |  |  |  |  |
| 8 | Pc.c001708243.Contig1 | 1454 | - | 150880 | 152319 | cRMPc07010 | 41270 | 2627 | 111289 | 152559 |  |  |  |  |  |  |  |  |  |
| 8 | PbContig176 | 2574 | - | 155186 | 157964 |  |  |  |  |  |  |  |  |  |  |  |  |  |  |
| 8 | MALPY02736 | 3805 | - | 155191 | 159848 |  |  |  |  |  |  | 262 | 2000 |  |  |  |  |  |  |
| 8 | Pc.c001003464.Contig1 | 1611 | - | 156561 | 158685 | cRMPc07011 | 4662 | 574 | 155186 | 159848 |  |  |  |  |  |  |  |  |  |
| 8 | Pc.c000403344.Contig1 | 636 | + | 160422 | 161061 | cRMPc07012 | 639 | 395 | 160422 | 161061 |  |  |  |  |  |  |  |  |  |
| 8 | MALPY00259 | 15339 | + | 161456 | 178400 |  |  |  |  |  | 477 | 262 |  |  |  |  |  |  |  |
| 8 | Pc.c000303488.Contig1 | 1507 | + | 161525 | 163032 |  |  |  |  |  |  |  |  |  |  |  |  |  |  |
| 8 | Pb.c001502419.Contig1 | 4581 | - | 161913 | 166863 |  |  |  |  |  |  |  |  |  |  |  |  |  |  |
| 8 | Pb.c000901867.Contig1 | 7897 | + | 168476 | 177384 |  |  |  |  |  |  |  |  |  |  |  |  |  |  |
| 8 | MALPY03516 | 639 | - | 171125 | 171755 |  |  |  |  |  |  | 270 | 1000 |  |  |  |  |  |  |
| 8 | Pc.c000503460.Contig1 | 623 | + | 171839 | 172462 |  |  |  |  |  |  |  |  |  |  |  |  |  |  |
| JC8 | MALPY03542 | 561 | - | 172617 | 173193 |  |  |  |  |  |  | 270 | 5000 |  |  |  |  |  |  |
| 8 | Pc.c001404761.Contig1 | 1491 | - | 175815 | 177276 |  |  |  |  |  |  |  |  |  |  |  |  |  |  |
| 8 | PbContig380 | 4626 | + | 177594 | 183142 |  |  |  |  |  |  |  |  |  |  |  |  |  |  |
| 8 | MALPY00274 | 8442 | + | 178373 | 187987 |  |  |  |  |  | 477 | 270 | 600 |  |  |  |  |  |  |
| 8 | Pb.c001301784.Contig1 | 4874 | + | 183589 | 188499 |  |  |  |  |  |  |  |  |  |  |  |  |  |  |
| 8 | Pc.c001003904.Contig1 | 642 | + | 184099 | 184741 | cRMPc07013 | 27043 | 86 | 161456 | 188499 |  |  |  |  |  |  |  |  |  |
| 8 | MALPY00516 | 9237 | - | 188585 | 197712 |  |  |  |  |  | 304 | 270 |  |  |  |  |  |  |  |
| 8 | Pb.c001202037.Contig1 | 2096 | + | 192992 | 195088 |  |  |  |  |  |  |  |  |  |  |  |  |  |  |
| 8 | Pc.c001304761.Contig1 | 736 | - | 193256 | 193992 |  |  |  |  |  |  |  |  |  |  |  |  |  |  |
| 8 | Pc.c000803389.Contig1 | 942 | - | 194358 | 195294 |  |  |  |  |  |  |  |  |  |  |  |  |  |  |
| 8 | PbContig102 | 10207 | + | 195286 | 205694 |  |  |  |  |  |  |  |  |  |  |  |  |  |  |
| 8 | Pc.c001304156.Contig1 | 1488 | - | 195341 | 196880 |  |  |  |  |  |  |  |  |  |  |  |  |  |  |
| 8 | MALPY02201 | 2880 | - | 198141 | 201249 |  |  |  |  |  | 304 |  |  |  |  |  |  |  |  |
| 8 | Pc.c001003267.Contig1 | 2506 | + | 200569 | 203030 |  |  |  |  |  |  |  |  |  |  |  |  |  |  |
| 8 | MALPY01830 | 8134 | - | 200745 | 208863 | cRMPc07014 | 20278 | 1753 | 188585 | 208863 | 304 | 237 | 1700 |  |  |  |  |  |  |
| 8 | MALPY00885 | 18640 | + | 210616 | 231371 |  |  |  |  |  | 284A | 237 |  |  |  |  |  |  |  |
| 8 | Pc.c000703448.Contig1 | 4976 | + | 213103 | 218079 |  |  |  |  |  |  |  |  |  |  |  |  |  |  |
| 8 | Pb.c000501503.Contig1 | 4626 | - | 214310 | 218936 |  |  |  |  |  |  |  |  |  |  |  |  |  |  |
| 8 | Pc.c001304220.Contig1 | 3575 | + | 220255 | 223788 |  |  |  |  |  |  |  |  |  |  |  |  |  |  |
| 8 | Pb.c000001761.Contig1 | 2277 | - | 222286 | 224563 |  |  |  |  |  |  |  |  |  |  |  |  |  |  |
| 8 | PbContig212 | 12365 | - | 224830 | 238586 |  |  |  |  |  |  |  |  |  |  |  |  |  |  |
| 8 | Pc.c000602896.Contig1 | 4356 | - | 226318 | 231875 |  |  |  |  |  |  |  |  |  |  |  |  |  |  |
| 8 | MALPY03092 | 1961 | - | 231449 | 233410 |  |  |  |  |  | 284A | 8 | 400 |  |  |  |  |  |  |
| 8 | MALPY00572 | 14777 | + | 233196 | 247618 |  |  |  |  |  |  | 8 |  |  |  |  |  |  |  |
| 8 | Pc.c000101986.Contig2 | 3760 | - | 233815 | 237678 |  |  |  |  |  |  |  |  |  |  |  |  |  |  |
| 8 | Pc.c000503605.Contig1 | 987 | + | 237878 | 238913 |  |  |  |  |  |  |  |  |  |  |  |  |  |  |
| 8 | Pc.c000603539.Contig1 | 1342 | + | 239082 | 240415 |  |  |  |  |  |  |  |  |  |  |  |  |  |  |
| 8 | Pc.c000603085.Contig1 | 4705 | + | 239871 | 244576 |  |  |  |  |  |  |  |  |  |  |  |  |  |  |
| 8 | Pb.c001001450.Contig2 | 8582 | + | 239872 | 248047 |  |  |  |  |  |  |  |  |  |  |  |  |  |  |
| 8 | Pc.c000902709.Contig2 | 7266 | - | 244695 | 252681 |  |  |  |  |  |  |  |  |  |  |  |  |  |  |
| 8 | Pb.c001502423.Contig1 | 2493 | + | 248966 | 251429 |  |  |  |  |  |  |  |  |  |  |  |  |  |  |
| 8 | MALPY01377 | 8560 | + | 249700 | 258395 |  |  |  |  |  | 57 | 8 | 2200 |  |  |  |  |  |  |
| 8 | Pb.c000201554.Contig1 | 2588 | + | 252367 | 254850 |  |  |  |  |  |  |  |  |  |  |  |  |  |  |
| 8 | PbContig62 | 8287 | + | 255050 | 263805 |  |  |  |  |  |  |  |  |  |  |  |  |  |  |
| 8 | PcContig79 | 4918 | - | 255324 | 260559 |  |  |  |  |  |  |  |  |  |  |  |  |  |  |
| 8 | MALPY03149 | 297 | - | 256637 | 256958 |  |  |  |  |  |  | 67 | 1100 |  |  |  |  |  |  |
| 8 | MALPY00742 | 21369 | + | 257953 | 278531 |  |  |  |  |  | 57 | 67 |  |  |  |  |  |  |  |
| 8 | Pc.c000803306.Contig1 | 1817 | - | 260957 | 262751 |  |  |  |  |  |  |  |  |  |  |  |  |  |  |
| 8 | Pc.c000402763.Contig1 | 6388 | - | 262879 | 268865 |  |  |  |  |  |  |  |  |  |  |  |  |  |  |
| 8 | Pb.c001401725.Contig1 | 11913 | - | 264384 | 277150 |  |  |  |  |  |  |  |  |  |  |  |  |  |  |
| 8 | Pc.c000403277.Contig1 | 1236 | - | 268996 | 270178 |  |  |  |  |  |  |  |  |  |  |  |  |  |  |
| 8 | Pc.c001003307.Contig1 | 2497 | - | 271625 | 274128 |  |  |  |  |  |  |  |  |  |  |  |  |  |  |
| 8 | Pc.c001003423.Contig1 | 1124 | - | 274466 | 275590 | cRMPc07015 | 67915 | 625 | 210616 | 278531 |  |  |  |  |  |  |  |  |  |
| 8 | MALPY00687 | 16444 | - | 279156 | 296557 |  |  |  |  |  | 57 |  |  |  |  |  |  |  |  |
| 8 | Pb.c000201493.Contig1 | 9593 | - | 279394 | 288993 |  |  |  |  |  |  |  |  |  |  |  |  |  |  |
| 8 | Pc.c000202855.Contig1 | 6177 | - | 280450 | 286630 |  |  |  |  |  |  |  |  |  |  |  |  |  |  |
| 8 | Pb.c000101085.Contig1 | 5416 | - | 291969 | 297451 |  |  |  |  |  |  |  |  |  |  |  |  |  |  |
| 8 | Pc.c001204264.Contig1 | 2069 | - | 292215 | 294285 |  |  |  |  |  |  |  |  |  |  |  |  |  |  |
| 8 | Pc.c001002698.Contig3 | 2895 | - | 294550 | 297445 |  |  |  |  |  |  |  |  |  |  |  |  |  |  |
| 8 | Pc.c001304738.Contig1 | 1226 | - | 294550 | 295776 |  |  |  |  |  |  |  |  |  |  |  |  |  |  |
| 8 | MALPY03143 | 545 | - | 294796 | 295341 |  |  |  |  |  |  | 211 | 1600 |  |  |  |  |  |  |
| 8 | MALPY02612 | 9488 | - | 296971 | 306896 |  |  |  |  |  | 57 | 211 |  |  |  |  |  |  |  |
| 8 | Pb.c001101524.Contig1 | 2572 | - | 297934 | 300585 |  |  |  |  |  |  |  |  |  |  |  |  |  |  |
| 8 | Pc.c000202829.Contig1 | 1540 | - | 298175 | 299837 |  |  |  |  |  |  |  |  |  |  |  |  |  |  |
| 8 | MALPY03324 | 436 | - | 298634 | 299073 |  |  |  |  |  |  | 68 | 9000 |  |  |  |  |  |  |
| 8 | Pb.c001803363.Contig1 | 4150 | + | 302159 | 306842 |  |  |  |  |  |  |  |  |  |  |  |  |  |  |
| 8 | Pc.c000202702.Contig1 | 4762 | + | 303638 | 308823 |  |  |  |  |  |  |  |  |  |  |  |  |  |  |
| 8 | MALPY01277 | 16173 | + | 307096 | 323344 |  |  |  |  |  | 588 | 68 |  |  |  |  |  |  |  |
| 8 | PbContig281 | 6227 | + | 308492 | 314504 |  |  |  |  |  |  |  |  |  |  |  |  |  |  |
| 8 | Pc.c000303300.Contig1 | 7082 | + | 309117 | 316332 |  |  |  |  |  |  |  |  |  |  |  |  |  |  |
| 8 | Pb.c001101804.Contig1 | 3245 | + | 315128 | 318653 |  |  |  |  |  |  |  |  |  |  |  |  |  |  |
| 8 | Pc.c001003498.Contig1 | 2449 | + | 316782 | 319261 |  |  |  |  |  |  |  |  |  |  |  |  |  |  |
| 8 | PbContig277 | 12640 | + | 319434 | 334143 |  |  |  |  |  |  |  |  |  |  |  |  |  |  |
| 8 | Pc.c000402777.Contig1 | 4353 | + | 319666 | 324353 |  |  |  |  |  |  |  |  |  |  |  |  |  |  |
| 8 | MALPY03006 | 1598 | + | 323469 | 325057 |  |  |  |  |  | 588 | 34 | 1900 |  |  |  |  |  |  |
| 8 | MALPY01083 | 7772 | + | 327050 | 335527 |  |  |  |  |  | 395 | 34 |  |  |  |  |  |  |  |
| 8 | PcContig194 | 5711 | + | 329003 | 335455 |  |  |  |  |  |  |  |  |  |  |  |  |  |  |
| 8 | MALPY03133 | 184 | - | 331937 | 332121 |  |  |  |  |  |  | 304 | 3500 |  |  |  |  |  |  |
| 8 | Pb.c001602096.Contig1 | 4944 | + | 333829 | 339068 |  |  |  |  |  |  |  |  |  |  |  |  |  |  |
| 8 | MALPY01938 | 4553 | - | 334730 | 339559 |  |  |  |  |  | 395 | 304 | 800 |  |  |  |  |  |  |
| 8 | Pc.c000202736.Contig1 | 1502 | - | 335330 | 336812 |  |  |  |  |  |  |  |  |  |  |  |  |  |  |
| 8 | Pc.c000803709.Contig1 | 1802 | - | 337183 | 339048 |  |  |  |  |  |  |  |  |  |  |  |  |  |  |
| 8 | MALPY02750 | 4576 | - | 339415 | 344023 |  |  |  |  |  | 168 | 304 | 7000 |  |  |  |  |  |  |
| 8 | PbContig358 | 2109 | + | 341395 | 343498 |  |  |  |  |  |  |  |  |  |  |  |  |  |  |
| 8 | Pc.c001304213.Contig1 | 3843 | + | 341502 | 345339 |  |  |  |  |  |  |  |  |  |  |  |  |  |  |
| man8 | MALPY02823 | 2433 | - | 343900 | 346333 |  |  |  |  |  | 168 |  |  |  |  |  |  |  | MALPY02750 |
| man8 | Pc.Contig7500 | 7221 | + | 346004 | 355842 |  |  |  |  |  |  |  |  |  |  |  |  |  | MALPY00861/MALPY00667 |
| man8 | Pb.c001803407.Contig1 | 2133 | - | 346097 | 348230 |  |  |  |  |  |  |  |  |  |  |  |  |  | \| |
| TK8 | MALPY00861 | 2705 | + | 349927 | 351824 |  |  |  |  |  | 341 |  |  |  |  |  |  |  |  |
| 8 | Pc.c001003274.Contig1 | 2269 | + | 353205 | 355780 |  |  |  |  |  |  |  |  |  |  |  |  |  |  |
| 8 | MALPY00667 | 11703 | - | 353416 | 366041 |  |  |  |  |  | 341 | 304 |  |  |  |  |  |  |  |
| 8 | PbContig349 | 9100 | + | 354090 | 363871 |  |  |  |  |  |  |  |  |  |  |  |  |  |  |
| 8 | Pc.c000303013.Contig1 | 1625 | + | 356062 | 357684 |  |  |  |  |  |  |  |  |  |  |  |  |  |  |
| 8 | Pc.c000303011.Contig1 | 1212 | - | 356618 | 357830 |  |  |  |  |  |  |  |  |  |  |  |  |  |  |
| 8 | MALPY03302 | 843 | - | 356852 | 357695 |  |  |  |  |  |  |  |  |  |  |  |  |  |  |
| 8 | Pc.c000202658.Contig1 | 12213 | + | 357653 | 370856 |  |  |  |  |  |  |  |  |  |  |  |  |  |  |
| TK8 | MALPY01238 | 3858 | + | 365786 | 369566 |  |  |  |  |  | 341 |  |  |  |  |  |  |  |  |
| TK8 | MALPY02055 | 4712 | + | 370103 | 373642 | cRMPc07016 | 94486 | 2090 | 279156 | 373642 | 341 |  |  | RMPs0703 | 328931 | 2090 | 44711 | 373642 |  |
| 8 | MALPY01008 | 5464 | + | 375732 | 381697 |  |  |  |  |  |  | 344 | 1600 |  |  |  |  |  |  |
| 8 | Pb.c000801718.Contig1 | 2924 | + | 376879 | 379998 |  |  |  |  |  |  |  |  |  |  |  |  |  |  |
| 8 | PcContig562 | 3604 | + | 377224 | 380945 |  |  |  |  |  |  |  |  |  |  |  |  |  |  |
| 8 | Pb.c001502910.Contig1 | 1104 | + | 380687 | 381791 |  |  |  |  |  |  |  |  |  |  |  |  |  |  |
| 8 | Pc.c001812182.Contig1 | 550 | + | 381373 | 381923 | cRMPc07017 | 6191 | 426 | 375732 | 381923 |  |  |  |  |  |  |  |  |  |
| 8 | MALPY00311 | 8543 | + | 382349 | 391963 |  |  |  |  |  |  | 344 |  |  |  |  |  |  |  |
| 8 | Pb.c001101962.Contig1 | 5345 | - | 382617 | 388815 |  |  |  |  |  |  |  |  |  |  |  |  |  |  |
| 8 | MALPY03367 | 653 | - | 385322 | 386776 |  |  |  |  |  |  |  |  |  |  |  |  |  |  |
| 8 | PcContig305 | 1748 | + | 385656 | 387404 |  |  |  |  |  |  |  |  |  |  |  |  |  |  |
| 8 | MALPY03351 | 174 | - | 386697 | 386871 |  |  |  |  |  |  |  |  |  |  |  |  |  |  |
| 8 | Pc.c000402730.Contig1 | 1070 | + | 387405 | 388475 |  |  |  |  |  |  |  |  |  |  |  |  |  |  |
| 8 | Pc.c001404820.Contig1 | 3047 | + | 388918 | 392106 |  |  |  |  |  |  |  |  |  |  |  |  |  |  |
| 8 | MALPY03198 | 264 | + | 389905 | 390169 | cRMPc07018 | 9757 | 85 | 382349 | 392106 |  | 319 | 2000 |  |  |  |  |  |  |
| 8 | Pb.c001101906.Contig1 | 6985 | + | 392191 | 399931 |  |  |  |  |  |  |  |  |  |  |  |  |  |  |
| 8 | MALPY00873 | 22272 | + | 392277 | 415638 |  |  |  |  |  |  | 319 |  |  |  |  |  |  |  |
| 8 | Pc.c001708779.Contig1 | 982 | + | 392542 | 393524 |  |  |  |  |  |  |  |  |  |  |  |  |  |  |
| 8 | Pb.c001502601.Contig1 | 3039 | + | 404221 | 407184 |  |  |  |  |  |  |  |  |  |  |  |  |  |  |
| 8 | PcContig490 | 5664 | + | 404531 | 410257 |  |  |  |  |  |  |  |  |  |  |  |  |  |  |
| 8 | Pb.c001904952.Contig1 | 649 | + | 407593 | 408412 | cRMPc07019 | 23447 | 848 | 392191 | 415638 |  |  |  | RMPs0704 | 39906 | 848 | 375732 | 415638 |  |
| 8 | Pc.c001304333.Contig1 | 4122 | + | 416486 | 420587 | cRMPc07020 | 4101 | 844 | 416486 | 420587 |  |  |  | RMPs0705 | 4101 | 844 | 416486 | 420587 |  |
| 8 | MALPY03395 | 914 | + | 421431 | 422333 |  |  |  |  |  |  |  |  |  |  |  |  |  |  |
| 8 | Pc.c001203254.Contig1 | 4184 | - | 421806 | 426469 |  |  |  |  |  |  |  |  |  |  |  |  |  |  |
| 8 | MALPY02061 | 9390 | + | 422605 | 432755 |  |  |  |  |  | 910A |  |  |  |  |  |  |  |  |
| 8 | PbContig162 | 9879 | - | 423296 | 434074 |  |  |  |  |  |  |  |  |  |  |  |  |  |  |
| 8 | Pc.c000002514.Contig1 | 2265 | - | 427652 | 429914 |  |  |  |  |  |  |  |  |  |  |  |  |  |  |
| 8 | MALPY03372 | 333 | - | 430408 | 430741 | cRMPc07021 | 12643 | 573 | 421431 | 434074 |  |  |  | RMPs0706 | 12643 | 573 | 421431 | 434074 |  |
| 8 | Pc.c000803461.Contig1 | 7398 | - | 434647 | 443397 |  |  |  |  |  |  |  |  |  |  |  |  |  |  |
| 8 | MALPY01140 | 2292 | - | 434708 | 437051 |  |  |  |  |  | 714 |  |  |  |  |  |  |  |  |
| 8 | MALPY01141 | 4434 | - | 437656 | 442579 |  |  |  |  |  | 714 | 126 | 6000 |  |  |  |  |  |  |
| 8 | PbContig279 | 13649 | - | 440417 | 456028 |  |  |  |  |  |  |  |  |  |  |  |  |  |  |
| TK8 | MALPY02228 | 3273 | - | 443966 | 446303 |  |  |  |  |  | 74 |  |  |  |  |  |  |  |  |
| 8 | Pc.c000803297.Contig1 | 3469 | + | 446621 | 449976 |  |  |  |  |  |  |  |  |  |  |  |  |  |  |
| 8 | MALPY03201 | 1609 | + | 447278 | 448866 |  |  |  |  |  | 74 | 126 |  |  |  |  |  |  |  |
| 8 | MALPY00025 | 4411 | - | 448862 | 453273 |  |  |  |  |  | 74 |  |  |  |  |  |  |  |  |
| 8 | MALPY01982 | 5593 | - | 453902 | 460165 |  |  |  |  |  | 74 |  |  |  |  |  |  |  |  |
| 8 | Pc.c000503470.Contig1 | 1204 | - | 454578 | 455785 |  |  |  |  |  |  |  |  |  |  |  |  |  |  |
| 8 | Pc.c000302940.Contig1 | 4058 | + | 457987 | 462005 |  |  |  |  |  |  |  |  |  |  |  |  |  |  |
| 8 | Pb.c000301680.Contig1 | 4339 | + | 458118 | 462404 |  |  |  |  |  |  |  |  |  |  |  |  |  |  |
| 8 | MALPY00501 | 2129 | - | 459439 | 461502 | cRMPc07022 | 27757 | 1132 | 434647 | 462404 | 74 | 303 | 2000 |  |  |  |  |  |  |
| 8 | MALPY01186 | 7908 | - | 463536 | 471419 |  |  |  |  |  | 918A | 303 |  |  |  |  |  |  |  |
| 8 | Pb.c000201512.Contig1 | 14230 | + | 463543 | 478181 |  |  |  |  |  |  |  |  |  |  |  |  |  |  |
| 8 | Pc.c001606496.Contig1 | 823 | + | 464988 | 465715 |  |  |  |  |  |  |  |  |  |  |  |  |  |  |
| 8 | Pc.c000602882.Contig1 | 4298 | + | 465867 | 470141 |  |  |  |  |  |  |  |  |  |  |  |  |  |  |
| 8 | MALPY02526 | 3746 | - | 472872 | 476618 |  |  |  |  |  | 353 |  |  |  |  |  |  |  |  |
| 8 | PcContig464 | 7963 | - | 472900 | 481857 |  |  |  |  |  |  |  |  |  |  |  |  |  |  |
| 8 | Pb.c002113200.Contig1 | 602 | - | 473381 | 473983 |  |  |  |  |  |  |  |  |  |  |  |  |  |  |
| 8 | MALPY00627 | 7651 | + | 477054 | 484835 |  |  |  |  |  | 353 | 180 | 900 |  |  |  |  |  |  |
| 8 | Pb.c000201503.Contig1 | 15209 | - | 479368 | 495489 |  |  |  |  |  |  |  |  |  |  |  |  |  |  |
| 8 | Pc.c000203331.Contig1 | 624 | + | 481150 | 481777 |  |  |  |  |  |  |  |  |  |  |  |  |  |  |
| 8 | PcContig291 | 6694 | - | 483236 | 490754 |  |  |  |  |  |  |  |  |  |  |  |  |  |  |
| 8 | MALPY01631 | 5710 | + | 485990 | 491685 |  |  |  |  |  |  | 180 | 4000 |  |  |  |  |  |  |
| 8 | Pc.c000302963.Contig1 | 3593 | - | 490735 | 494328 | cRMPc07023 | 31953 | 3627 | 463536 | 495489 |  |  |  |  |  |  |  |  |  |
| 8 | MALPY01862 | 9752 | - | 499116 | 508871 |  |  |  |  |  |  | 180 | 9000 |  |  |  |  |  |  |
| 8 | PcContig365 | 3649 | - | 501507 | 505111 |  |  |  |  |  |  |  |  |  |  |  |  |  |  |
| 8 | Pb.c001903676.Contig1 | 5816 | - | 502430 | 508228 | cRMPc07024 | 9755 | 2815 | 499116 | 508871 |  |  |  |  |  |  |  |  |  |
| 8 | MALPY01323 | 5665 | - | 511686 | 517357 |  |  |  |  |  |  |  |  |  |  |  |  |  |  |
| 8 | Pc.c001103067.Contig1 | 2688 | + | 512986 | 515908 |  |  |  |  |  |  |  |  |  |  |  |  |  |  |
| 8 | Pb.c001401694.Contig1 | 14245 | + | 516329 | 531164 |  |  |  |  |  |  |  |  |  |  |  |  |  |  |
| 8 | Pc.c001708260.Contig1 | 932 | - | 517164 | 518054 |  |  |  |  |  |  |  |  |  |  |  |  |  |  |
| 8 | MALPY01798 | 6010 | + | 518136 | 524251 |  |  |  |  |  | 37 | 180 |  |  |  |  |  |  |  |
| 8 | Pc.c000603493.Contig1 | 897 | - | 520900 | 521885 |  |  |  |  |  |  |  |  |  |  |  |  |  |  |
| 8 | PcContig499 | 6725 | - | 522123 | 529770 |  |  |  |  |  |  |  |  |  |  |  |  |  |  |
| JC8 | MALPY02896 | 2742 | xxx | 523000 |  |  |  |  |  |  | 467 |  |  |  |  |  |  |  |  |
| 8 | MALPY00156 | 21720 | + | 524880 | 548426 |  |  |  |  |  | 37 |  |  |  |  |  |  |  |  |
| 8 | MALPY03290 | 1746 | - | 525319 | 527068 |  |  |  |  |  | 467 |  |  |  |  |  |  |  |  |
| 8 | MALPY03061 | 1728 | - | 527191 | 528892 |  |  |  |  |  | 184A |  |  |  |  |  |  |  |  |
| 8 | MALPY03296 | 702 | - | 527809 | 528523 |  |  |  |  |  |  |  |  |  |  |  |  |  |  |
| 8 | PcContig519 | 3864 | - | 530133 | 534415 |  |  |  |  |  |  |  |  |  |  |  |  |  |  |
| 8 | Pb.c000201312.Contig1 | 4781 | + | 531633 | 536369 |  |  |  |  |  |  |  |  |  |  |  |  |  |  |
| 8 | Pc.c000602873.Contig1 | 4522 | + | 537632 | 542047 |  |  |  |  |  |  |  |  |  |  |  |  |  |  |
| 8 | Pb.c000601471.Contig1 | 10549 | + | 537831 | 548884 |  |  |  |  |  |  |  |  |  |  |  |  |  |  |
| 8 | Pc.c000002088.Contig2 | 3647 | - | 544299 | 547995 | cRMPc07025 | 37198 | 1454 | 511686 | 548884 |  |  |  | RMPs0707 | 114237 | 1454 | 434647 | 548884 |  |
| 8 | MALPY00150 - BP | 34875 | + | 550338 | 586112 |  |  |  |  |  |  |  |  |  |  |  |  |  |  |
| 8 | Pc.c001504188.Contig2 | 3024 | - | 552733 | 556046 |  |  |  |  |  |  |  |  |  |  |  |  |  |  |
| 8 | Pb.c001301802.Contig1 | 1515 | - | 554164 | 555658 |  |  |  |  |  |  |  |  |  |  |  |  |  |  |
| 8 | Pc.c000202876.Contig1 | 3812 | + | 557253 | 561396 |  |  |  |  |  |  |  |  |  |  |  |  |  |  |
| 8 | PbContig348 | 4059 | - | 557873 | 562207 |  |  |  |  |  |  |  |  |  |  |  |  |  |  |
| 8 | Pc.c000703307.Contig1 | 2584 | - | 561760 | 564910 |  |  |  |  |  |  |  |  |  |  |  |  |  |  |
| 8 | Pb.c001702714.Contig1 | 2513 | - | 562653 | 565266 |  |  |  |  |  |  |  |  |  |  |  |  |  |  |
| 4 | Pb.c000801806.Contig1 | 2066 | - | 565987 | 568705 |  |  |  |  |  |  |  |  |  |  |  |  |  |  |
| 4 | MALPY03500 | 862 | + | 567001 | 567863 |  |  |  |  |  |  |  |  |  |  |  |  |  |  |
| 4 | Pc.c000102664.Contig1 | 4323 | + | 568780 | 573151 |  |  |  |  |  |  |  |  |  |  |  |  |  |  |
| 4 | Pb.c000801810.Contig1 | 9772 | - | 568804 | 579153 |  |  |  |  |  |  |  |  |  |  |  |  |  |  |
| 4 | Pc.c000403265.Contig1 | 1199 | + | 577995 | 579308 |  |  |  |  |  |  |  |  |  |  |  |  |  |  |
| 4 | Pb.c002112264.Contig1 | 1088 | - | 579207 | 580361 |  |  |  |  |  |  |  |  |  |  |  |  |  |  |
| 4 | Pc.c000303356.Contig1 | 1412 | + | 579479 | 581001 | cRMPc07026 | 35774 | 17474 | 550338 | 586112 |  |  |  | RMPs0708 | 35774 | 17474 | 550338 | 586112 |  |
| Centromere - gap 17474 bp | |  |  | 600000 |  |  |  |  |  |  |  |  |  |  |  |  |  |  |  |
| 4 | Pc.c000202170.Contig1 | 9972 | + | 603586 | 614352 |  |  |  |  |  |  |  |  |  |  |  |  |  |  |
| 4 | PbContig27 | 3947 | - | 603824 | 607893 |  |  |  |  |  |  |  |  |  |  |  |  |  |  |
| 4 | MALPY00605 | 24720 | - | 604048 | 631214 |  |  |  |  |  | 224 |  |  |  |  |  |  |  |  |
| 4 | MALPY03477 | 203 | - | 607287 | 607490 |  |  |  |  |  |  |  |  |  |  |  |  |  |  |
| 4 | PbContig222 | 2462 | + | 609649 | 612148 |  |  |  |  |  |  |  |  |  |  |  |  |  |  |
| 4 | Pb.c001502565.Contig1 | 2870 | + | 612378 | 615209 |  |  |  |  |  |  |  |  |  |  |  |  |  |  |
| 4 | Pb.c000201606.Contig1 | 5982 | + | 616419 | 622362 |  |  |  |  |  |  |  |  |  |  |  |  |  |  |
| 4 | Pc.c001812520.Contig1 | 1326 | - | 617223 | 618614 |  |  |  |  |  |  |  |  |  |  |  |  |  |  |
| 4 | Pc.c000803266.Contig1 | 3970 | - | 618945 | 622792 |  |  |  |  |  |  |  |  |  |  |  |  |  |  |
| 4 | Pb.c001402205.Contig1 | 4496 | + | 623978 | 628459 |  |  |  |  |  |  |  |  |  |  |  |  |  |  |
| 4 | PcContig19 | 6226 | - | 630490 | 637052 |  |  |  |  |  |  |  |  |  |  |  |  |  |  |
| 4 | Pb.c001401776.Contig1 | 5166 | - | 632513 | 637982 |  |  |  |  |  |  |  |  |  |  |  |  |  |  |
| 4 | MALPY01211 | 5484 | + | 633472 | 639235 |  |  |  |  |  | 224 |  |  |  |  |  |  |  |  |
| 4 | Pc.c001404004.Contig2 | 3018 | - | 637007 | 640254 |  |  |  |  |  |  |  |  |  |  |  |  |  |  |
| man4 | Pb.c000500823.Contig1 - Ins | 12883 | - | 638353 | 651972 |  |  |  |  |  |  |  |  |  |  |  |  |  |  |
| man4 | MALPY00785 - Ins | 7596 | - | 639071 | 646690 |  |  |  |  |  | 224 | 251 | 5000 |  |  |  |  |  |  |
| man4 | PcContig371 - Ins | 5058 | - | 640358 | 645569 |  |  |  |  |  |  |  |  |  |  |  |  |  |  |
| JC4 | MALPY03491 | 223 | + | 641375 | 641601 |  |  |  |  |  |  |  |  |  |  |  |  |  |  |
| 4 | MALPY00802 | 14482 | - | 649328 | 665067 |  |  |  |  |  | 98 | 251 |  |  |  |  |  |  |  |
| 4 | PbContig289 | 12415 | + | 652801 | 667246 |  |  |  |  |  |  |  |  |  |  |  |  |  |  |
| 4 | PcContig362 | 4245 | + | 653866 | 658910 |  |  |  |  |  |  |  |  |  |  |  |  |  |  |
| 4 | Pc.c000402253.Contig3 | 3541 | + | 659095 | 663397 |  |  |  |  |  |  |  |  |  |  |  |  |  |  |
| 4 | Pc.c000202663.Contig1 | 3366 | + | 663962 | 667396 |  |  |  |  |  |  |  |  |  |  |  |  |  |  |
| 4 | MALPY00803 | 10022 | + | 664426 | 675950 |  |  |  |  |  | 98 |  |  |  |  |  |  |  |  |
| 4 | Pb.c000701557.Contig1 | 1221 | + | 668089 | 669310 |  |  |  |  |  |  |  |  |  |  |  |  |  |  |
| 4 | Pc.c000803800.Contig1 | 2512 | + | 668335 | 671303 |  |  |  |  |  |  |  |  |  |  |  |  |  |  |
| 4 | Pb.c001702941.Contig1 | 2793 | + | 670189 | 672991 |  |  |  |  |  |  |  |  |  |  |  |  |  |  |
| 4 | Pc.c000803204.Contig1 | 2673 | + | 672236 | 674991 |  |  |  |  |  |  |  |  |  |  |  |  |  |  |
| 4 | Pb.c001402439.Contig1 | 640 | - | 674426 | 675066 |  |  |  |  |  |  |  |  |  |  |  |  |  |  |
| 4 | Pb.c001402145.Contig1 | 3163 | - | 675824 | 678990 |  |  |  |  |  |  |  |  |  |  |  |  |  |  |
| 4 | MALPY01325 | 22319 | + | 676575 | 701755 |  |  |  |  |  | 98 | 300 | 12000 |  |  |  |  |  |  |
| 4 | PcContig660 | 2106 | + | 676660 | 678769 |  |  |  |  |  |  |  |  |  |  |  |  |  |  |
| 4 | Pc.c001811699.Contig1 | 1494 | + | 678229 | 679723 |  |  |  |  |  |  |  |  |  |  |  |  |  |  |
| 4 | Pc.c001504971.Contig1 | 1274 | - | 690054 | 691328 |  |  |  |  |  |  |  |  |  |  |  |  |  |  |
| 4 | Pb.c001601992.Contig2 | 8284 | + | 690266 | 698528 |  |  |  |  |  |  |  |  |  |  |  |  |  |  |
| 4 | Pc.c001606523.Contig1 | 1194 | - | 691577 | 692810 |  |  |  |  |  |  |  |  |  |  |  |  |  |  |
| 4 | PcContig662 | 6430 | - | 695504 | 702265 |  |  |  |  |  |  |  |  |  |  |  |  |  |  |
| 4 | Pb.c002007196.Contig1 | 1657 | - | 699183 | 700843 | cRMPc07027 | 98679 | 1060 | 603586 | 702265 |  |  |  |  |  |  |  |  |  |
| TK4 | MALPY02139 | 5049 | + | 703325 | 706942 | cRMPc07028 | 3617 | 12260 | 703325 | 706942 | 98 |  |  |  |  |  |  |  |  |
| 4 | MALPY01391 | 10261 | - | 719202 | 729906 |  |  |  |  |  | 759 | 300 |  |  |  |  |  |  |  |
| 4 | Pb.c001502502.Contig1 | 3309 | + | 719770 | 723085 |  |  |  |  |  |  |  |  |  |  |  |  |  |  |
| 4 | Pc.c000703521.Contig1 | 790 | - | 721893 | 722686 |  |  |  |  |  |  |  |  |  |  |  |  |  |  |
| 4 | Pb.c001502504.Contig1 | 4651 | + | 724134 | 728782 |  |  |  |  |  |  |  |  |  |  |  |  |  |  |
| 4 | Pc.c000703409.Contig1 | 2872 | - | 724970 | 727866 |  |  |  |  |  |  |  |  |  |  |  |  |  |  |
| 4 | Pc.c000703941.Contig1 | 2005 | - | 728455 | 730445 |  |  |  |  |  |  |  |  |  |  |  |  |  |  |
| 4 | MALPY00948 | 6480 | - | 729863 | 736343 |  |  |  |  |  | 759 | 143 | 4000 |  |  |  |  |  |  |
| 4 | Pc.c001708555.Contig1 | 946 | - | 733815 | 734761 |  |  |  |  |  |  |  |  |  |  |  |  |  |  |
| 4 | Pc.c001103116.Contig2 | 3180 | - | 734744 | 737924 |  |  |  |  |  |  |  |  |  |  |  |  |  |  |
| 4 | Pb.c000801843.Contig1 | 5355 | - | 737399 | 742826 |  |  |  |  |  |  |  |  |  |  |  |  |  |  |
| 4 | MALPY02995 | 835 | + | 737749 | 738608 |  |  |  |  |  |  |  |  |  |  |  |  |  |  |
| 4 | MALPY00908 | 4618 | - | 738801 | 743419 |  |  |  |  |  |  |  |  |  |  |  |  |  |  |
| 4 | Pc.c000904008.Contig1 | 833 | - | 738938 | 739771 | cRMPc07029 | 24217 | 1872 | 719202 | 743419 |  |  |  |  |  |  |  |  |  |
| man4 | MALPY02313 | 7236 | + | 745291 | 752691 |  |  |  |  |  |  |  |  |  |  |  |  |  |  |
| 4 | Pc.c000202225.Contig1 | 3262 | - | 751765 | 755027 |  |  |  |  |  |  |  |  |  |  |  |  |  |  |
| 4 | MALPY00943 | 9159 | + | 753229 | 763050 |  |  |  |  |  |  | 143 | 100 |  |  |  |  |  |  |
| 4 | Pc.c000603020.Contig1 | 2851 | - | 758491 | 761624 |  |  |  |  |  |  |  |  |  |  |  |  |  |  |
| 4 | Pb.c001904846.Contig1 | 656 | + | 760551 | 761207 |  |  |  |  |  |  |  |  |  |  |  |  |  |  |
| 4 | Pb.c001101813.Contig1 | 4409 | + | 762411 | 766823 |  |  |  |  |  |  |  |  |  |  |  |  |  |  |
| 4 | PcContig684 | 4146 | - | 762862 | 767008 |  |  |  |  |  |  |  |  |  |  |  |  |  |  |
| 4 | MALPY00931 | 6496 | + | 763132 | 769610 | cRMPc07030 | 24319 | 6939 | 745291 | 769610 | 517B | 143 | 6000 |  |  |  |  |  |  |
| 4 | Pc.c000202815.Contig1 | 4363 | - | 776549 | 781041 |  |  |  |  |  |  |  |  |  |  |  |  |  |  |
| 4 | Pb.c000801773.Contig1 | 2688 | - | 777409 | 780201 |  |  |  |  |  |  |  |  |  |  |  |  |  |  |
| 4 | MALPY00666 | 2857 | - | 777782 | 780717 |  |  |  |  |  | 1215 | 143 | 4500 |  |  |  |  |  |  |
| 4 | MALPY02378 | 3300 | - | 779912 | 785490 |  |  |  |  |  | 1215 |  |  |  |  |  |  |  |  |
| 4 | Pc.c001003856.Contig1 | 695 | - | 783142 | 783831 | cRMPc07031 | 8941 | 60 | 776549 | 785490 |  |  |  |  |  |  |  |  |  |
| 4 | PcContig298 | 8850 | - | 785550 | 794971 |  |  |  |  |  |  |  |  |  |  |  |  |  |  |
| 4 | MALPY01801 | 9265 | + | 786442 | 796024 |  |  |  |  |  | 50A | 143 |  |  |  |  |  |  |  |
| 4 | Pb.c000501430.Contig1 | 6697 | + | 787483 | 794693 | cRMPc07032 | 10474 | 2079 | 785550 | 796024 |  |  |  | RMPs0709 | 192438 | 2079 | 603586 | 796024 |  |
| man4 | MALPY01750 | 2068 | + | 798103 | 800182 | cRMPc07033 | 2079 | 609 | 798103 | 800182 |  |  |  | RMPs0710 | 2079 | 609 | 798103 | 800182 |  |
| man4 | MALPY02757 | 3134 | + | 800791 | 803922 | cRMPc07034 | 3131 | 3503 | 800791 | 803922 |  |  |  | RMPs0711 | 3131 | 3503 | 800791 | 803922 |  |
| man4 | MALPY02403 | 2196 | + | 807425 | 809667 | cRMPc07035 | 2242 | 1764 | 807425 | 809667 |  |  |  | RMPs0712 | 2242 | 1764 | 807425 | 809667 |  |
| TK4 | MALPY01957 | 5438 | + | 811431 | 816643 |  |  |  |  |  | 741A |  |  |  |  |  |  |  |  |
| 4 | Pb.c000801399.Contig4 | 2736 | - | 815999 | 818735 |  |  |  |  |  |  |  |  |  |  |  |  |  |  |
| 4 | MALPY02634 | 2478 | - | 817495 | 819973 | cRMPc07036 | 8542 | 366 | 811431 | 819973 | 741A |  |  | RMPs0713 | 8542 | 366 | 811431 | 819973 |  |
| 4 | Pc.c000903492.Contig1 | 3287 | + | 820339 | 823626 |  |  |  |  |  |  |  |  |  |  |  |  |  |  |
| 4 | Pb.c002112275.Contig1 | 1944 | - | 820660 | 822604 | cRMPc07037 | 3287 | 11542 | 820339 | 823626 |  |  |  | RMPs0714 | 3287 | 11542 | 820339 | 823626 |  |
| man4 | MALPY02381 | 2422 | - | 835168 | 837570 | cRMPc07038 | 2402 | 370 | 835168 | 837570 |  |  |  | RMPs0715 | 2402 | 370 | 835168 | 837570 |  |
| JC4 | MALPY03138 | 795 | + | 837940 | 838765 | cRMPc07039 | 825 | 167 | 837940 | 838765 |  |  |  | RMPs0716 | 825 | 167 | 837940 | 838765 |  |
| 4 | MALPY03010 | 1389 | - | 838932 | 840460 |  |  |  |  |  | 406 |  |  |  |  |  |  |  |  |
| 4 | MALPY00299 | 5296 | - | 840375 | 845653 | cRMPc07040 | 6721 |  | 838932 | 845653 | 406 |  |  | RMPs0717 | 6721 |  | 838932 | 845653 |  |
| Telomere | |  |  |  |  |  |  |  |  |  |  |  |  |  |  |  |  |  |  |
|  |  | Pb Pc Py contig |  |  |  |  | cRMP contig | Gap to next cRMP contig |  |  |  |  |  |  | cRMP scaffold | Gap to next cRMP scaffold |  |  |  |
|  | Number | 311 |  |  |  | Number | 40 | 39 |  |  |  |  |  | Number | 17 | 16 |  |  |  |
|  | Average size (bp) | 4634 |  |  |  | Average size (bp) | 18308 | 2906 |  |  |  |  |  | Average size (bp) | 45795 | 4196 |  |  |  |
|  | Minimum size (bp) | 174 |  |  |  | Minimum size (bp) | 639 | 60 |  |  |  |  |  | Minimum size (bp) | 825 | 167 |  |  |  |
|  | Maximum size (bp) | 24720 |  |  |  | Maximum size (bp) | 98679 | 22876 |  |  |  |  |  | Maximum size (bp) | 328931 | 22876 |  |  |  |
|  | Total size (bp) | 1441136 |  |  |  | Total size (bp) | 732323 | 113330 |  |  |  |  |  | Total size (bp) | 778520 | 67133 |  |  |  |
|  |  |  |  |  |  |  |  |  |  |  |  |  |  |  |  |  |  |  |  |
