# Supplementary material for: A Plasmodium Whole-Genome Synteny Map: Indels and Synteny Breakpoints as Foci for Species-Specific Genes
Source: PLoS Pathog. 2005 Dec 23;1(4):e44. doi: 10.1371/journal.ppat.0010044 (PMC1317653; doi:10.1371/journal.ppat.0010044)
Supplement: Supplementary file 8 [file ppat.0010044.st024.doc]

Table S24 - RMPchr8


# Table S24 - RMPchr8

|  |  |  |  |  |  |  |  |  |  |  |  |  |  |  |  |  |  |  |  |
| --- | --- | --- | --- | --- | --- | --- | --- | --- | --- | --- | --- | --- | --- | --- | --- | --- | --- | --- | --- |
| Table S24: Tiling path of RMP contigs combined to form cRMP chromosome 8. | | | | | | |  |  |  |  |  |  |  |  |  |  |  |  |  |
|  |  |  |  |  |  |  |  |  |  |  |  |  |  |  |  |  |  |  |  |
| Tiling paths of P. berghei (Pb), P. c. chabaudi (Pc) and P. y. yoelii (Py) contigs combined into composite rodent malaria parasite (cRMP) | | | | | | | | | |  |  |  |  |  |  |  |  |  |  |
| contigs aligned against parts of the 14 chromosomes of P. falciparum (Pf). The cRMP contigs are further linked by physical linkage | | | | | | | | | |  |  |  |  |  |  |  |  |  |  |
| and PCR (see Methods) to form cRMP scaffolds. Different synteny blocks with homology to different P. falciparum chromosomes | | | | | | | | | |  |  |  |  |  |  |  |  |  |  |
| together form the cRMP chromosomes. The tables also show gaps in the alignment (black lines), cRMP telomeres (white bars) and | | | | | | | | | |  |  |  |  |  |  |  |  |  |  |
| predicted centromeres (plum/black bars). Contigs, physical groups or PCR groups interrupted by inter- or intrasyntenic regions are | | | | | | | | | |  |  |  |  |  |  |  |  |  |  |
| printed in bold. A summary of the number of RMP contigs, cRMP contigs and cRMP scaffolds as well as their average, minimum, | | | | | | | | | |  |  |  |  |  |  |  |  |  |  |
| maximum and total sizes is provided. Column headings are as follows: | | | | |  |  |  |  |  |  |  |  |  |  |  |  |  |  |  |
|  |  |  |  |  |  |  |  |  |  |  |  |  |  |  |  |  |  |  |  |
| Pf chr.: P. falciparum chromosome to which the RMP contigs are aligned | | | | | |  |  |  |  |  |  |  |  |  |  |  |  |  |  |
| (#: PROmer analysis� from this study; JC# and TK#: Carlton et al.; man#: manual BLAST analysis; | | | | | | |  |  |  |  |  |  |  |  |  |  |  |  |  |
| i: inversed orientation of the synteny block; colours refer to those used in Fig. 2 of the paper); | | | | | | |  |  |  |  |  |  |  |  |  |  |  |  |  |
| Pb Pc Py contig: names of the aligned RMP contigs; | | |  |  |  |  |  |  |  |  |  |  |  |  |  |  |  |  |  |
| Size: size (bp) of the aligned RMP contigs; | |  |  |  |  |  |  |  |  |  |  |  |  |  |  |  |  |  |  |
| +/-: orientation of the aligned RMP contigs; | |  |  |  |  |  |  |  |  |  |  |  |  |  |  |  |  |  |  |
| Start contig & End contig: estimated start & end coordinates of alignment of the RMP contigs based on the coordinates | | | | | | | | |  |  |  |  |  |  |  |  |  |  |  |
| of PROmer hits corrected for the contig�s size and orientation and presence of inter- and intrasyntenic regions | | | | | | | |  |  |  |  |  |  |  |  |  |  |  |  |
| (the start position of the first RMP contig in the alignment was set at zero); | | | | | |  |  |  |  |  |  |  |  |  |  |  |  |  |  |
| cRMP contig: names of cRMP contigs | |  |  |  |  |  |  |  |  |  |  |  |  |  |  |  |  |  |  |
| (the first 2 digits refer to the cRMP chromosome number and the following 3 digits designate the order of the contigs); | | | | | | | | |  |  |  |  |  |  |  |  |  |  |  |
| cRMP contig size: size of cRMP contigs based on the corrected coordinates of alignment; | | | | | | |  |  |  |  |  |  |  |  |  |  |  |  |  |
| Gap size to next cRMP contig: size of gaps between neighbouring cRMP contigs; | | | | | | |  |  |  |  |  |  |  |  |  |  |  |  |  |
| Start cRMP contig & End cRMP contig: estimated start & end coordinates of alignment of the cRMP contigs; | | | | | | | |  |  |  |  |  |  |  |  |  |  |  |  |
| Phys. group (Py): physical linkage of neighbouring P. y. yoelii contigs; | | | | | |  |  |  |  |  |  |  |  |  |  |  |  |  |  |
| PCR group (Py): neighbouring P. y. yoelii contigs were linked previously or for this study by PCR; | | | | | | |  |  |  |  |  |  |  |  |  |  |  |  |  |
| PCR size (Py): size of the PCR product; | |  |  |  |  |  |  |  |  |  |  |  |  |  |  |  |  |  |  |
| cRMP scaffold: names of cRMP scaffolds | |  |  |  |  |  |  |  |  |  |  |  |  |  |  |  |  |  |  |
| (the first 2 digits refer to the cRMP chromosome number and the following 2 digits designate the order of the scaffolds); | | | | | | | | |  |  |  |  |  |  |  |  |  |  |  |
| cRMP scaffold size: size of cRMP scaffolds; | | |  |  |  |  |  |  |  |  |  |  |  |  |  |  |  |  |  |
| Gap size to next cRMP scaffold: size of gaps between neighbouring cRMP scaffolds; | | | | | | |  |  |  |  |  |  |  |  |  |  |  |  |  |
| Start cRMP scaffold & End cRMP scaffold: estimated start & end coordinates of alignment of the cRMP scaffolds; | | | | | | | | |  |  |  |  |  |  |  |  |  |  |  |
| Overlap: Linkage between RMP contigs established through manual BLAST analysis. | | | | | | |  |  |  |  |  |  |  |  |  |  |  |  |  |
|  |  |  |  |  |  |  |  |  |  |  |  |  |  |  |  |  |  |  |  |
| Pf chr. | Pb Pc Py contig | Size | +/- | Start contig | End contig | cRMP contig | cRMP contig size | Gap size to next cRMP contig | Start cRMP contig | End cRMP contig | Phys. group (Py) | PCR group (Py) | PCR size (Py) | cRMP scaffold | cRMP scaffold size | Gap size to next cRMP scaffold | Start cRMP scaffold | End cRMP scaffold | Overlap (manual) |
| Telomere | |  |  |  |  |  |  |  |  |  |  |  |  |  |  |  |  |  |  |
| man7 | AY149028 = PcYAC101 (Fischer) | 80019 | + | 0 | 80019 |  |  |  |  |  |  |  |  |  |  |  |  |  | \| |
| man7 | PbContig5655 | 7130 | + | 64410 | 71540 |  |  |  |  |  |  |  |  |  |  |  |  |  | \| |
| man7 | MALPY01759 | 5381 | + | 64774 | 70155 |  |  |  |  |  |  |  |  |  |  |  |  |  | MALPY02593 |
| man7 | MALPY00003 | 3821 | + | 71075 | 74896 |  |  |  |  |  |  |  |  |  |  |  |  |  |  |
| 7 | PbContig237 | 5446 | + | 72084 | 77620 |  |  |  |  |  |  |  |  |  |  |  |  |  |  |
| man7 | MALPY03007 | 1749 | + | 73159 | 74908 |  |  |  |  |  |  |  |  |  |  |  |  |  |  |
| 7 | MALPY02593 | 3663 | + | 73164 | 76900 |  |  |  |  |  | 1025 |  |  |  |  |  |  |  |  |
| 7 | Pc.c001103085.Contig2 | 2982 | - | 75459 | 78514 |  |  |  |  |  |  |  |  |  |  |  |  |  |  |
| 7 | MALPY01883 | 6048 | + | 77091 | 83091 | cRMPc08001 | 83091 | 399 | 0 | 83091 | 1025 |  |  |  |  |  |  |  |  |
| 7 | Pc.c001708157.Contig1 | 932 | - | 83490 | 84422 |  |  |  |  |  |  |  |  |  |  |  |  |  |  |
| 7 | MALPY00637 | 10499 | + | 83614 | 94481 |  |  |  |  |  | 1025 |  |  |  |  |  |  |  |  |
| 7 | Pb.c001803545.Contig1 | 1512 | + | 85339 | 86849 |  |  |  |  |  |  |  |  |  |  |  |  |  |  |
| 7 | Pb.c001803496.Contig1 | 1250 | + | 86811 | 88073 |  |  |  |  |  |  |  |  |  |  |  |  |  |  |
| 7 | Pb.c001803956.Contig1 | 1310 | + | 89049 | 90425 |  |  |  |  |  |  |  |  |  |  |  |  |  |  |
| 7 | Pc.c001203244.Contig1 | 3236 | + | 90971 | 94219 |  |  |  |  |  |  |  |  |  |  |  |  |  |  |
| 7 | Pb.c002006859.Contig1 | 1583 | + | 91603 | 93198 | cRMPc08002 | 10991 | 7681 | 83490 | 94481 |  |  |  | cRMPs0801 | 94481 | 7681 | 0 | 94481 |  |
| 7 | MALPY00187 | 28320 | - | 102162 | 130644 |  |  |  |  |  |  |  |  |  |  |  |  |  |  |
| 7 | Pb.c000101088.Contig1 | 14697 | + | 102219 | 116907 |  |  |  |  |  |  |  |  |  |  |  |  |  |  |
| JC7 | MALPY03398 | 1549 | + | 104914 | 106463 |  |  |  |  |  |  | 337 | 1400 |  |  |  |  |  |  |
| 7 | Pc.c000803460.Contig1 | 3872 | - | 113183 | 117046 |  |  |  |  |  |  |  |  |  |  |  |  |  |  |
| 7 | PbContig98 | 5948 | + | 117486 | 123482 |  |  |  |  |  |  |  |  |  |  |  |  |  |  |
| 7 | Pc.c001708429.Contig1 | 620 | - | 121100 | 121717 |  |  |  |  |  |  |  |  |  |  |  |  |  |  |
| 7 | Pc.c001304728.Contig1 | 624 | - | 121987 | 122611 | cRMPc08003 | 28482 | 5699 | 102162 | 130644 |  |  |  |  |  |  |  |  |  |
| 7 | MALPY01760 | 4831 | + | 136343 | 141177 | cRMPc08004 | 4834 | 6387 | 136343 | 141177 | 195 | 337 |  |  |  |  |  |  |  |
| JC7 | MALPY01603 | 4597 | xxx | 140000 |  |  |  |  |  |  | 195 |  |  |  |  |  |  |  |  |
| 7 | MALPY00533 | 28594 | - | 147564 | 175978 |  |  |  |  |  | 195 | 196 | 1200 |  |  |  |  |  |  |
| 7 | PcContig167 | 3283 | + | 154737 | 158204 |  |  |  |  |  |  |  |  |  |  |  |  |  |  |
| 7 | Pb.c002007449.Contig1 | 1033 | + | 154973 | 155991 |  |  |  |  |  |  |  |  |  |  |  |  |  |  |
| 7 | Pb.c000701762.Contig1 | 737 | + | 156304 | 157041 |  |  |  |  |  |  |  |  |  |  |  |  |  |  |
| 7 | Pb.c000701501.Contig1 | 2695 | + | 156843 | 159742 |  |  |  |  |  |  |  |  |  |  |  |  |  |  |
| 7 | PcContig321 | 1058 | + | 172617 | 173666 |  |  |  |  |  |  |  |  |  |  |  |  |  |  |
| 7 | Pc.c001812402.Contig1 | 636 | + | 174047 | 174673 |  |  |  |  |  |  |  |  |  |  |  |  |  |  |
| 7 | Pc.c001813004.Contig1 | 617 | + | 174063 | 174673 |  |  |  |  |  |  |  |  |  |  |  |  |  |  |
| 7 | Pc.c001708967.Contig1 | 809 | + | 174379 | 175203 | cRMPc08005 | 28414 | 2972 | 147564 | 175978 |  |  |  |  |  |  |  |  |  |
| 7 | Pc.c000202763.Contig1 | 3966 | + | 178950 | 183081 |  |  |  |  |  |  |  |  |  |  |  |  |  |  |
| 7 | MALPY01656 | 4335 | + | 179969 | 184757 |  |  |  |  |  |  | 196 |  |  |  |  |  |  |  |
| 7 | Pb.c000001793.Contig1 | 3940 | - | 180806 | 185149 |  |  |  |  |  |  |  |  |  |  |  |  |  |  |
| 7 | Pc.c001103914.Contig1 | 1645 | + | 183664 | 185207 | cRMPc08006 | 6257 | 347 | 178950 | 185207 |  |  |  | cRMPs0802 | 83045 | 347 | 102162 | 185207 |  |
| 7 | PcContig539 | 8102 | + | 185554 | 194218 |  |  |  |  |  |  |  |  |  |  |  |  |  |  |
| 7 | MALPY02348 | 3020 | - | 186949 | 190434 |  |  |  |  |  | 539 |  |  |  |  |  |  |  |  |
| 7 | Pb.c000901785.Contig1 | 1230 | + | 187616 | 189374 |  |  |  |  |  |  |  |  |  |  |  |  |  |  |
| 7 | MALPY02039 | 3897 | + | 190626 | 194451 |  |  |  |  |  | 539 | 354 | 400 |  |  |  |  |  |  |
| 7 | Pb.c001803361.Contig1 | 1925 | - | 191597 | 193690 | cRMPc08007 | 8897 | 4744 | 185554 | 194451 |  |  |  |  |  |  |  |  |  |
| 7 | MALPY02000 | 6412 | + | 199195 | 206015 |  |  |  |  |  | 404 | 354 |  |  |  |  |  |  |  |
| 7 | Pc.c000402797.Contig1 | 2561 | - | 201105 | 203666 | cRMPc08008 | 6820 | 451 | 199195 | 206015 |  |  |  |  |  |  |  |  |  |
| 7 | Pb.c001001447.Contig1 | 6391 | + | 206466 | 213177 |  |  |  |  |  |  |  |  |  |  |  |  |  |  |
| 7 | MALPY01243 | 5803 | + | 206928 | 213348 | cRMPc08009 | 6882 | 2872 | 206466 | 213348 | 404 |  |  | cRMPs0803 | 27794 | 2872 | 185554 | 213348 |  |
| 7 | Pc.c000803415.Contig1 | 1961 | - | 216220 | 218181 |  |  |  |  |  |  |  |  |  |  |  |  |  |  |
| 7 | Pb.c000801778.Contig1 | 3718 | + | 216676 | 220394 |  |  |  |  |  |  |  |  |  |  |  |  |  |  |
| 7 | MALPY00278 | 4297 | + | 216956 | 221253 | cRMPc08010 | 5033 | 2109 | 216220 | 221253 |  |  |  | cRMPs0804 | 5033 | 2109 | 216220 | 221253 |  |
| 7 | PcContig445 | 6715 | - | 223362 | 229995 |  |  |  |  |  |  |  |  |  |  |  |  |  |  |
| 7 | Pb.c001904465.Contig1 | 1515 | + | 225885 | 227595 |  |  |  |  |  |  |  |  |  |  |  |  |  |  |
| 7 | MALPY00924 | 16329 | + | 226864 | 244470 |  |  |  |  |  |  | 122 | 700 |  |  |  |  |  |  |
| 7 | Pb.c001702943.Contig1 | 2217 | + | 228061 | 230084 |  |  |  |  |  |  |  |  |  |  |  |  |  |  |
| 7 | Pb.c000701473.Contig1 | 1970 | + | 230290 | 232260 |  |  |  |  |  |  |  |  |  |  |  |  |  |  |
| 7 | Pc.c000603425.Contig1 | 872 | + | 230364 | 231236 |  |  |  |  |  |  |  |  |  |  |  |  |  |  |
| 7 | Pc.c001707774.Contig1 | 1552 | + | 231196 | 232748 |  |  |  |  |  |  |  |  |  |  |  |  |  |  |
| 7 | PbContig273 | 9377 | + | 233947 | 244229 |  |  |  |  |  |  |  |  |  |  |  |  |  |  |
| man7 | PbContig4379 | 5650 | + | 238970 | 244620 |  |  |  |  |  |  |  |  |  |  |  |  |  | MALPY00924 |
| man7 | MALPY00681 | 3598 | - | 244447 | 248045 |  |  |  |  |  |  |  |  |  |  |  |  |  | \| |
| 7 | MALPY00037 | 47020 | - | 245170 | 292683 |  |  |  |  |  | 520 | 122 |  |  |  |  |  |  |  |
| man7 | PbContig3363 | 3321 | - | 245322 | 248643 |  |  |  |  |  |  |  |  |  |  |  |  |  | \| |
| 7 | PcContig31 | 4412 | - | 249514 | 254067 |  |  |  |  |  |  |  |  |  |  |  |  |  |  |
| 7 | PbContig391 | 30031 | + | 259155 | 290043 |  |  |  |  |  |  |  |  |  |  |  |  |  |  |
| 7 | PcContig527 | 2948 | - | 259518 | 262469 |  |  |  |  |  |  |  |  |  |  |  |  |  |  |
| 7 | PcContig226 | 6724 | - | 263884 | 270527 |  |  |  |  |  |  |  |  |  |  |  |  |  |  |
| 7 | MALPY03514 | 547 | + | 273910 | 274460 |  |  |  |  |  |  |  |  |  |  |  |  |  |  |
| 7 | Pc.c001103897.Contig1 | 1877 | - | 279358 | 281235 |  |  |  |  |  |  |  |  |  |  |  |  |  |  |
| 7 | Pc.c000002681.Contig1 | 5330 | - | 281574 | 286426 |  |  |  |  |  |  |  |  |  |  |  |  |  |  |
| 7 | Pc.c001404918.Contig1 | 1588 | - | 287147 | 288735 |  |  |  |  |  |  |  |  |  |  |  |  |  |  |
| 7 | MALPY03306 | 321 | + | 288620 | 288935 |  |  |  |  |  |  |  |  |  |  |  |  |  |  |
| 7 | Pc.c001203940.Contig1 | 2135 | - | 288970 | 291017 |  |  |  |  |  |  |  |  |  |  |  |  |  |  |
| 7 | MALPY03305 | 252 | - | 289355 | 289607 |  |  |  |  |  |  |  |  |  |  |  |  |  |  |
| 7 | Pb.c001301459.Contig2 - BP | 4367 | - | 290002 | 294447 |  |  |  |  |  |  |  |  |  |  |  |  |  |  |
| 7 | MALPY03581 | 683 | - | 290353 | 291036 |  |  |  |  |  |  | 218 | 250 |  |  |  |  |  |  |
| 7 | MALPY00262 - BP | 22657 | - | 291087 | 314736 |  |  |  |  |  | 1141 | 218 | 700 |  |  |  |  |  |  |
| i3 | PcContig159 | 3116 | + | 291464 | 298191 |  |  |  |  |  |  |  |  |  |  |  |  |  |  |
| i3 | Pc.c000903323.Contig1 | 1054 - 3068 | + | 294591 | 294591 |  |  |  |  |  |  |  |  |  |  |  |  |  |  |
| i3 | Pb.c000101043.Contig1 | 3005 | + | 297045 | 300050 |  |  |  |  |  |  |  |  |  |  |  |  |  |  |
| i3 | PcContig427 | 2562 | + | 300326 | 302887 |  |  |  |  |  |  |  |  |  |  |  |  |  |  |
| i3 | Pb.c000401231.Contig2 | 27778 | + | 300767 | 332326 |  |  |  |  |  |  |  |  |  |  |  |  |  |  |
| i3 | Pc.c001813145.Contig1 | 770 | + | 304464 | 305234 |  |  |  |  |  |  |  |  |  |  |  |  |  |  |
| i3 | Pc.c000303492.Contig1 | 1017 | + | 305340 | 306363 |  |  |  |  |  |  |  |  |  |  |  |  |  |  |
| i3 | Pc.c001606039.Contig1 | 4022 | + | 306261 | 310544 |  |  |  |  |  |  |  |  |  |  |  |  |  |  |
| i3 | MALPY03101 | 512 | - | 306917 | 307432 |  |  |  |  |  |  | 239 | 1700 |  |  |  |  |  |  |
| i3 | MALPY03438 | 815 | - | 308793 | 309605 |  |  |  |  |  |  | 239 | 2500 |  |  |  |  |  |  |
| i3 | Pc.c001003943.Contig1 | 1050 | + | 311237 | 312311 |  |  |  |  |  |  |  |  |  |  |  |  |  |  |
| i3 | MALPY03579 | 467 | + | 312813 | 313275 |  |  |  |  |  |  | 239 |  |  |  |  |  |  |  |
| i3 | PcContig679 | 6855 | - | 314663 | 324402 |  |  |  |  |  |  |  |  |  |  |  |  |  |  |
| i3 | MALPY00204 | 12651 | + | 315428 | 330691 |  |  |  |  |  | 1141 | 218 | 4000 |  |  |  |  |  |  |
| i3 | Pc.c000302879.Contig1 | 2474 | + | 325644 | 327774 |  |  |  |  |  |  |  |  |  |  |  |  |  |  |
| i3 | Pc.c001404767.Contig1 | 1509 | + | 329753 | 331238 | cRMPc08011 | 108964 | 159 | 223362 | 332326 |  |  |  |  |  |  |  |  |  |
| i3 | MALPY02429 | 4174 | + | 332485 | 336647 | cRMPc08012 | 4162 | 1045 | 332485 | 336647 |  |  |  |  |  |  |  |  |  |
| i3 | MALPY02024 | 12557 | + | 337692 | 349978 |  |  |  |  |  |  | 218 |  |  |  |  |  |  |  |
| i3 | Pb.c001102190.Contig1 | 1198 | - | 340672 | 341870 |  |  |  |  |  |  |  |  |  |  |  |  |  |  |
| i3 | Pb.c000401236.Contig2 | 14656 | - | 347231 | 362065 |  |  |  |  |  |  |  |  |  |  |  |  |  |  |
| i3 | MALPY00909 | 13929 | + | 350970 | 365827 |  |  |  |  |  | 128 |  |  |  |  |  |  |  |  |
| i3 | Pc.c001606026.Contig1 | 2847 | + | 351126 | 353968 |  |  |  |  |  |  |  |  |  |  |  |  |  |  |
| i3 | Pc.c001104196.Contig1 | 2725 | + | 356192 | 359017 |  |  |  |  |  |  |  |  |  |  |  |  |  |  |
| i3 | MALPY03196 | 691 | - | 358549 | 359345 |  |  |  |  |  |  |  |  |  |  |  |  |  |  |
| i3 | MALPY03195 | 457 | - | 358815 | 359356 |  |  |  |  |  |  | 330 | 1200 |  |  |  |  |  |  |
| i3 | MALPY03573 | 826 | + | 360293 | 361125 |  |  |  |  |  |  | 330 |  |  |  |  |  |  |  |
| i3 | Pc.c000203287.Contig1 | 637 | - | 363938 | 364572 |  |  |  |  |  |  |  |  |  |  |  |  |  |  |
| i3 | Pc.c000202803.Contig1 | 1323 | - | 364534 | 365845 |  |  |  |  |  |  |  |  |  |  |  |  |  |  |
| i3 | Pb.c001904547.Contig1 | 977 | - | 364660 | 365631 |  |  |  |  |  |  |  |  |  |  |  |  |  |  |
| i3 | MALPY00731 | 9880 | - | 365292 | 375065 |  |  |  |  |  | 128 |  |  |  |  |  |  |  |  |
| i3 | Pb.c001202106.Contig1 | 5872 | - | 368001 | 373998 |  |  |  |  |  |  |  |  |  |  |  |  |  |  |
| i3 | Pc.c000003024.Contig1 | 959 | - | 368842 | 369801 |  |  |  |  |  |  |  |  |  |  |  |  |  |  |
| i3 | PcContig164 | 2777 | - | 370788 | 373831 | cRMPc08013 | 37373 | 1921 | 337692 | 375065 |  |  |  | cRMPs0805 | 151703 | 1921 | 223362 | 375065 |  |
| i3 | MALPY02678 | 3351 | - | 376986 | 381093 | cRMPc08014 | 4107 | 837 | 376986 | 381093 |  | 84 | 6500 |  |  |  |  |  |  |
| i3 | Pb.c001904382.Contig1 | 3234 | - | 381930 | 385023 |  |  |  |  |  |  |  |  |  |  |  |  |  |  |
| i3 | MALPY02312 | 2958 | - | 382492 | 385323 |  |  |  |  |  |  | 84 | 2200 |  |  |  |  |  |  |
| i3 | Pc.c001505007.Contig1 | 1381 | + | 383117 | 384332 |  |  |  |  |  |  |  |  |  |  |  |  |  |  |
| i3 | PcContig26 | 4596 | + | 385177 | 390113 |  |  |  |  |  |  |  |  |  |  |  |  |  |  |
| i3 | MALPY03100 | 882 | + | 387444 | 388655 |  |  |  |  |  |  | 84 | 700 |  |  |  |  |  |  |
| i3 | MALPY00759 | 5353 | - | 388742 | 394077 |  |  |  |  |  | 268 | 84 |  |  |  |  |  |  |  |
| i3 | PcContig612 | 6039 | + | 390471 | 396600 |  |  |  |  |  |  |  |  |  |  |  |  |  |  |
| i3 | MALPY02212 | 4027 | - | 394389 | 398614 |  |  |  |  |  | 268 |  |  |  |  |  |  |  |  |
| i3 | PbContig141 | 5235 | - | 395352 | 400796 |  |  |  |  |  |  |  |  |  |  |  |  |  |  |
| iTK3 | MALPY02729 | 3396 | - | 399065 | 403256 | cRMPc08015 | 21326 | 12270 | 381930 | 403256 | 268 |  |  | cRMPs0806 | 26270 | 12270 | 376986 | 403256 |  |
| i3 | MALPY00041 | 11604 | - | 415526 | 427422 |  |  |  |  |  |  | 166 | 800 |  |  |  |  |  |  |
| i3 | MALPY03024 | 535 | - | 417200 | 417759 |  |  |  |  |  |  |  |  |  |  |  |  |  |  |
| i3 | Pb.c001702308.Contig1 | 4457 | + | 418289 | 422749 |  |  |  |  |  |  |  |  |  |  |  |  |  |  |
| i3 | Pc.c001708919.Contig1 | 629 | - | 418914 | 419543 |  |  |  |  |  |  |  |  |  |  |  |  |  |  |
| i3 | Pb.c000701183.Contig1 | 4357 | + | 423248 | 427632 |  |  |  |  |  |  |  |  |  |  |  |  |  |  |
| i3 | Pc.c001003292.Contig1 | 2587 | + | 425839 | 428457 |  |  |  |  |  |  |  |  |  |  |  |  |  |  |
| i3 | MALPY01621 - BP | 6091 | + | 427350 | 433600 |  |  |  |  |  |  | 166 |  |  |  |  |  |  |  |
| i3 | PcContig478 | 1 - 1425 | + | 428501 | 430425 |  |  |  |  |  |  |  |  |  |  |  |  |  |  |
| 9 | PcContig784 | 2465 | + | 430474 | 432936 | cRMPc08016 | 18074 | 2597 | 415526 | 433600 |  |  |  | cRMPs0807 | 18074 | 2597 | 415526 | 433600 |  |
| TK9 | MALPY00534 | 3440 | - | 436197 | 439197 | cRMPc08017 | 3000 | 1340 | 436197 | 439197 | 69 |  |  |  |  |  |  |  |  |
| 9 | MALPY00535 | 9398 | - | 440537 | 450648 |  |  |  |  |  | 69 |  |  |  |  |  |  |  |  |
| 9 | Pb.c002113653.Contig1 | 627 | - | 442141 | 442768 |  |  |  |  |  |  |  |  |  |  |  |  |  |  |
| 9 | PcContig135 | 6368 | + | 444038 | 450583 |  |  |  |  |  |  |  |  |  |  |  |  |  |  |
| 9 | Pb.c001401768.Contig1 | 5912 | + | 444836 | 450941 |  |  |  |  |  |  |  |  |  |  |  |  |  |  |
| 9 | MALPY03284 | 779 | - | 448093 | 448845 | cRMPc08018 | 10404 | 4219 | 440537 | 450941 |  |  |  | cRMPs0808 | 14744 | 4219 | 436197 | 450941 |  |
| 9 | MALPY01236 | 5242 | + | 455160 | 460844 |  |  |  |  |  |  | 287 | 1000 |  |  |  |  |  |  |
| 9 | Pb.c001802859.Contig1 | 4148 | - | 455743 | 460446 |  |  |  |  |  |  |  |  |  |  |  |  |  |  |
| 9 | Pc.c001812787.Contig1 | 636 | - | 455946 | 456582 | cRMPc08019 | 5684 | 953 | 455160 | 460844 |  |  |  |  |  |  |  |  |  |
| 9 | MALPY00470 | 5831 | + | 461797 | 467514 |  |  |  |  |  |  | 287 |  |  |  |  |  |  |  |
| 9 | PbContig39 | 3959 | - | 463697 | 467936 | cRMPc08020 | 6139 | 5268 | 461797 | 467936 |  |  |  | cRMPs0809 | 12776 | 5268 | 455160 | 467936 |  |
| TK9 | MALPY02283 | 2452 | + | 473204 | 475720 | cRMPc08021 | 2516 | 297 | 473204 | 475720 | 679 |  |  |  |  |  |  |  |  |
| man9 | MALPY03224 | 1413 | - | 476017 | 477202 |  |  |  |  |  | 679 |  |  |  |  |  |  |  |  |
| 9 | Pc.c001812939.Contig1 | 755 | - | 476623 | 477387 |  |  |  |  |  |  |  |  |  |  |  |  |  |  |
| man9 | MALPY01708 - Ins | 5872 | + | 477088 | 483550 |  |  |  |  |  | 679 | 113 | 600 |  |  |  |  |  |  |
| 9 | Pb.c001202087.Contig1 | 3099 | + | 477858 | 480957 |  |  |  |  |  |  |  |  |  |  |  |  |  |  |
| 9 | PbContig338 | 6418 | - | 480933 | 487674 |  |  |  |  |  |  |  |  |  |  |  |  |  |  |
| 9 | Pc.c001708712.Contig1 | 1476 | - | 481954 | 483559 |  |  |  |  |  |  |  |  |  |  |  |  |  |  |
| 9 | Pc.c000602858.Contig1 | 7180 | - | 483714 | 491325 |  |  |  |  |  |  |  |  |  |  |  |  |  |  |
| 9 | MALPY03231 | 959 | - | 483722 | 484681 |  |  |  |  |  |  | 113 | 600 |  |  |  |  |  |  |
| 9 | MALPY01881 | 2018 | + | 485108 | 487126 |  |  |  |  |  |  | 113 | 700 |  |  |  |  |  |  |
| 9 | MALPY01450 | 4531 | - | 487344 | 492890 |  |  |  |  |  |  | 113 | 6000 |  |  |  |  |  |  |
| 9 | Pb.c000801818.Contig1 | 2447 | - | 487943 | 490564 |  |  |  |  |  |  |  |  |  |  |  |  |  |  |
| 9 | PcContig68 | 1842 | - | 492440 | 494273 |  |  |  |  |  |  |  |  |  |  |  |  |  |  |
| TK9 | MALPY01983 | 4332 | - | 493919 | 497666 | cRMPc08022 | 21649 | 613 | 476017 | 497666 | 571 |  |  |  |  |  |  |  |  |
| 9 | MALPY00886 | 17996 | - | 498279 | 519255 |  |  |  |  |  | 571 | 113 |  |  |  |  |  |  |  |
| 9 | Pb.c001904892.Contig1 | 883 | + | 498732 | 499591 |  |  |  |  |  |  |  |  |  |  |  |  |  |  |
| 9 | PbContig138 | 2653 | + | 500814 | 503458 |  |  |  |  |  |  |  |  |  |  |  |  |  |  |
| 9 | Pc.c001811688.Contig1 | 2385 | - | 501888 | 504434 |  |  |  |  |  |  |  |  |  |  |  |  |  |  |
| 9 | Pb.c000101395.Contig1 | 3251 | + | 503820 | 508157 |  |  |  |  |  |  |  |  |  |  |  |  |  |  |
| 9 | Pb.c000101398.Contig1 | 3779 | + | 508125 | 511964 |  |  |  |  |  |  |  |  |  |  |  |  |  |  |
| 9 | Pc.c001606041.Contig1 | 1057 | - | 508156 | 509171 |  |  |  |  |  |  |  |  |  |  |  |  |  |  |
| 9 | PcContig120 | 3052 | - | 509297 | 512409 |  |  |  |  |  |  |  |  |  |  |  |  |  |  |
| 9 | Pb.c000101400.Contig1 | 5282 | + | 512695 | 518394 |  |  |  |  |  |  |  |  |  |  |  |  |  |  |
| 9 | Pc.c000402759.Contig1 | 2875 | - | 513405 | 516664 | cRMPc08023 | 20976 | 2061 | 498279 | 519255 |  |  |  |  |  |  |  |  |  |
| man9 | MALPY02482 | 2914 | + | 521316 | 524230 |  |  |  |  |  | 571 |  |  |  |  |  |  |  |  |
| 9 | Pc.c000803798.Contig1 | 1366 | - | 523702 | 525074 |  |  |  |  |  |  |  |  |  |  |  |  |  |  |
| 9 | MALPY01012 | 16718 | + | 524261 | 541698 |  |  |  |  |  | 571 |  |  |  |  |  |  |  |  |
| 9 | Pb.c001101486.Contig4 | 16761 | - | 524318 | 541901 |  |  |  |  |  |  |  |  |  |  |  |  |  |  |
| 9 | Pc.c000002570.Contig1 | 6435 | - | 527460 | 534127 |  |  |  |  |  |  |  |  |  |  |  |  |  |  |
| 9 | Pc.c000302870.Contig1 | 1475 | - | 534132 | 535661 |  |  |  |  |  |  |  |  |  |  |  |  |  |  |
| 9 | MALPY03374 | 805 | + | 534377 | 535176 |  |  |  |  |  |  |  |  |  |  |  |  |  |  |
| 9 | Pc.c000703303.Contig1 | 1447 | - | 535700 | 537085 |  |  |  |  |  |  |  |  |  |  |  |  |  |  |
| 9 | Pc.c000903475.Contig1 | 2977 | - | 538385 | 541373 |  |  |  |  |  |  |  |  |  |  |  |  |  |  |
| 9 | PcContig476 | 3894 | - | 541463 | 545470 |  |  |  |  |  |  |  |  |  |  |  |  |  |  |
| 9 | MALPY03054 | 562 | - | 542047 | 542611 |  |  |  |  |  |  | 112 | 500 |  |  |  |  |  |  |
| 9 | Pb.c000901861.Contig1 | 4988 | - | 542077 | 547176 |  |  |  |  |  |  |  |  |  |  |  |  |  |  |
| 9 | MALPY01755 | 2888 | + | 542988 | 545867 | cRMPc08024 | 25860 | 8762 | 521316 | 547176 |  | 112 |  | cRMPs0810 | 73972 | 8762 | 473204 | 547176 |  |
| 9 | Pc.c001204014.Contig1 | 1399 | + | 555938 | 557337 | cRMPc08025 | 1399 | 755 | 555938 | 557337 |  |  |  | cRMPs0811 | 1399 | 755 | 555938 | 557337 |  |
| 9 | Pb.c000801811.Contig1 | 5866 | + | 558092 | 564140 |  |  |  |  |  |  |  |  |  |  |  |  |  |  |
| 9 | MALPY01772 | 6010 | + | 558838 | 565511 |  |  |  |  |  |  | 247 - Ins | 800 |  |  |  |  |  |  |
| 9 | PcContig782 | 5069 | - | 558870 | 564177 | cRMPc08026 | 7419 | 346 | 558092 | 565511 |  |  |  |  |  |  |  |  |  |
| 9 | Pb.c000301481.Contig1 | 3947 | - | 565857 | 569804 |  |  |  |  |  |  |  |  |  |  |  |  |  |  |
| 9 | MALPY00231 | 11290 | - | 566311 | 577709 |  |  |  |  |  | 511 | 247 - Ins |  |  |  |  |  |  |  |
| 9 | Pb.c000201634.Contig1 | 6846 | - | 569571 | 576971 |  |  |  |  |  |  |  |  |  |  |  |  |  |  |
| 9 | PcContig714 | 4047 | + | 571439 | 575587 |  |  |  |  |  |  |  |  |  |  |  |  |  |  |
| 9 | MALPY03339 | 492 | - | 574551 | 575043 |  |  |  |  |  |  | 329 | 3800 |  |  |  |  |  |  |
| 9 | Pc.c001606563.Contig1 | 1046 | + | 575506 | 576561 | cRMPc08027 | 11852 | 754 | 565857 | 577709 |  |  |  |  |  |  |  |  |  |
| 9 | MALPY00095 | 22812 | + | 578463 | 602055 |  |  |  |  |  | 511 | 329 |  |  |  |  |  |  |  |
| 9 | PcContig412 | 1603 | - | 578695 | 580295 |  |  |  |  |  |  |  |  |  |  |  |  |  |  |
| 9 | PbContig8 | 5480 | - | 578869 | 584346 |  |  |  |  |  |  |  |  |  |  |  |  |  |  |
| 9 | Pb.c000601185.Contig1 | 13652 | - | 585273 | 599125 |  |  |  |  |  |  |  |  |  |  |  |  |  |  |
| 9 | Pc.c000903415.Contig1 | 4148 | + | 594307 | 598411 |  |  |  |  |  |  |  |  |  |  |  |  |  |  |
| 9 | MALPY03476 | 243 | - | 597191 | 597434 |  |  |  |  |  |  |  |  |  |  |  |  |  |  |
| TK9 | MALPY00771 | 5074 | - | 601865 | 606371 | cRMPc08028 | 27908 | 802 | 578463 | 606371 | 511 |  |  | cRMPs0812 | 48279 | 802 | 558092 | 606371 |  |
| 9 | MALPY00985 | 22503 | - | 607173 | 631347 |  |  |  |  |  | 125 |  |  |  |  |  |  |  |  |
| 9 | Pb.c000801664.Contig1 | 8026 | + | 608028 | 616843 |  |  |  |  |  |  |  |  |  |  |  |  |  |  |
| 9 | Pc.c001203848.Contig1 | 3389 | - | 608462 | 612303 |  |  |  |  |  |  |  |  |  |  |  |  |  |  |
| 9 | Pc.c001003396.Contig1 | 2225 | - | 612788 | 614986 |  |  |  |  |  |  |  |  |  |  |  |  |  |  |
| 9 | Pb.c002006852.Contig1 | 1696 | + | 624892 | 626636 |  |  |  |  |  |  |  |  |  |  |  |  |  |  |
| 9 | Pb.c001402075.Contig1 | 5940 | - | 628681 | 636646 |  |  |  |  |  |  |  |  |  |  |  |  |  |  |
| 9 | PcContig393 | 13035 | - | 631978 | 645909 |  |  |  |  |  |  |  |  |  |  |  |  |  |  |
| 9 | MALPY03107 | 1704 | - | 633050 | 634754 |  |  |  |  |  | 125 | 208 | 3000 |  |  |  |  |  |  |
| 9 | MALPY00993 | 7948 | + | 637710 | 646113 |  |  |  |  |  | 154 | 208 |  |  |  |  |  |  |  |
| 9 | PbContig373 | 9742 | - | 638417 | 648583 |  |  |  |  |  |  |  |  |  |  |  |  |  |  |
| 9 | MALPY01586 | 5187 | + | 645402 | 650357 |  |  |  |  |  | 154 | 38 | 1600 |  |  |  |  |  |  |
| 9 | PcContig678 | 1204 | - | 646162 | 647366 |  |  |  |  |  |  |  |  |  |  |  |  |  |  |
| 9 | PcContig256 | 3008 | - | 647517 | 650543 | cRMPc08029 | 43370 | 1500 | 607173 | 650543 |  |  |  |  |  |  |  |  |  |
| 9 | MALPY02062 | 2399 | + | 652043 | 654442 |  |  |  |  |  | 218 | 38 |  |  |  |  |  |  |  |
| 9 | MALPY01500 | 2989 | - | 654056 | 657010 |  |  |  |  |  | 218 |  |  |  |  |  |  |  |  |
| 9 | Pc.c001304283.Contig1 | 2499 | - | 654604 | 657161 |  |  |  |  |  |  |  |  |  |  |  |  |  |  |
| 9 | Pb.c001101887.Contig1 | 1976 | + | 655573 | 657415 | cRMPc08030 | 5372 | 534 | 652043 | 657415 |  |  |  |  |  |  |  |  |  |
| 9 | MALPY01070 | 3716 | + | 657949 | 661789 |  |  |  |  |  | 218 | 43 | 2800 |  |  |  |  |  |  |
| 9 | Pb.c001602048.Contig1 | 6290 | + | 658378 | 664804 |  |  |  |  |  |  |  |  |  |  |  |  |  |  |
| 9 | Pc.c000502396.Contig3 | 6090 | - | 658484 | 664816 | cRMPc08031 | 6867 | 901 | 657949 | 664816 |  |  |  |  |  |  |  |  |  |
| 9 | MALPY00683 | 20371 | + | 665717 | 688756 |  |  |  |  |  |  | 43 | 1300 |  |  |  |  |  |  |
| 9 | Pb.c002007019.Contig1 | 2583 | + | 665816 | 668324 |  |  |  |  |  |  |  |  |  |  |  |  |  |  |
| 9 | Pc.c001404721.Contig1 | 1496 | + | 666635 | 668025 |  |  |  |  |  |  |  |  |  |  |  |  |  |  |
| 9 | Pc.c001204417.Contig1 | 1377 | + | 668343 | 669687 |  |  |  |  |  |  |  |  |  |  |  |  |  |  |
| 9 | Pb.c001803542.Contig1 | 3215 | + | 668822 | 672037 |  |  |  |  |  |  |  |  |  |  |  |  |  |  |
| 9 | Pc.c000002082.Contig1 | 4484 | - | 674852 | 680009 |  |  |  |  |  |  |  |  |  |  |  |  |  |  |
| 9 | Pb.c002006901.Contig1 | 2061 | + | 676974 | 679170 |  |  |  |  |  |  |  |  |  |  |  |  |  |  |
| 9 | PcContig426 | 6929 | - | 682790 | 689799 |  |  |  |  |  |  |  |  |  |  |  |  |  |  |
| 9 | PbContig67 | 3672 | - | 685094 | 688869 | cRMPc08032 | 24082 | 984 | 665717 | 689799 |  |  |  |  |  |  |  |  |  |
| 9 | MALPY01247 | 4048 | - | 690783 | 694933 |  |  |  |  |  |  | 43 | 900 |  |  |  |  |  |  |
| 9 | Pb.c001202325.Contig1 | 1120 | + | 691807 | 692993 |  |  |  |  |  |  |  |  |  |  |  |  |  |  |
| 9 | Pb.c000001641.Contig1 | 5655 | + | 694315 | 700558 |  |  |  |  |  |  |  |  |  |  |  |  |  |  |
| 9 | MALPY00658 | 6787 | - | 695285 | 702555 |  |  |  |  |  |  | 43 | 1800 |  |  |  |  |  |  |
| 9 | Pc.c001605978.Contig1 | 5390 | + | 695498 | 701467 | cRMPc08033 | 11772 | 2149 | 690783 | 702555 |  |  |  |  |  |  |  |  |  |
| 9 | MALPY02153 | 2594 | + | 704704 | 707330 |  |  |  |  |  |  | 43 | 1500 |  |  |  |  |  |  |
| 9 | Pb.c001402191.Contig1 | 1790 | - | 705534 | 707389 | cRMPc08034 | 2685 | 864 | 704704 | 707389 |  |  |  |  |  |  |  |  |  |
| 9 | Pb.c001402199.Contig1 | 1925 | + | 708253 | 710262 |  |  |  |  |  |  |  |  |  |  |  |  |  |  |
| 9 | Pc.c001708176.Contig1 | 831 | - | 708462 | 709293 |  |  |  |  |  |  |  |  |  |  |  |  |  |  |
| 9 | MALPY00733 | 4574 | + | 708489 | 713409 |  |  |  |  |  | 153 | 43 |  |  |  |  |  |  |  |
| 9 | PcContig115 | 3502 | - | 709731 | 713523 |  |  |  |  |  |  |  |  |  |  |  |  |  |  |
| 9 | Pb.c001402123.Contig1 | 4750 | + | 711276 | 715978 |  |  |  |  |  |  |  |  |  |  |  |  |  |  |
| 9 | MALPY00984 | 6483 | - | 714758 | 721130 |  |  |  |  |  | 153 |  |  |  |  |  |  |  |  |
| 9 | Pc.c000803951.Contig1 | 1208 | + | 718305 | 719468 |  |  |  |  |  |  |  |  |  |  |  |  |  |  |
| 9 | Pc.c000703426.Contig1 | 1133 | + | 720878 | 721995 |  |  |  |  |  |  |  |  |  |  |  |  |  |  |
| 9 | MALPY03066 | 1582 | - | 721009 | 722345 |  |  |  |  |  | 153 | 138 | 2500 |  |  |  |  |  |  |
| 9 | Pb.c000401460.Contig1 | 6400 | - | 721131 | 727439 |  |  |  |  |  |  |  |  |  |  |  |  |  |  |
| 9 | Pc.c000703424.Contig1 | 1600 | + | 721889 | 723489 |  |  |  |  |  |  |  |  |  |  |  |  |  |  |
| 9 | MALPY00348 | 12993 | + | 724803 | 738083 |  |  |  |  |  | 54 | 138 |  |  |  |  |  |  |  |
| 9 | Pc.c000303019.Contig1 | 2431 | - | 725466 | 727996 |  |  |  |  |  |  |  |  |  |  |  |  |  |  |
| 9 | Pb.c000701500.Contig1 | 4274 | - | 728187 | 732698 |  |  |  |  |  |  |  |  |  |  |  |  |  |  |
| 9 | PcContig681 | 5008 | - | 728981 | 734227 |  |  |  |  |  |  |  |  |  |  |  |  |  |  |
| 9 | MALPY03271 | 1723 | + | 729089 | 730804 |  |  |  |  |  |  | 40 | 6000 |  |  |  |  |  |  |
| 9 | Pb.c000101315.Contig1 | 1474 | + | 735701 | 737130 |  |  |  |  |  |  |  |  |  |  |  |  |  |  |
| 9 | MALPY02770 | 5359 | - | 738047 | 743695 |  |  |  |  |  | 54 | 40 | 500 |  |  |  |  |  |  |
| 9 | Pb.c000001789.Contig1 | 3757 | + | 738981 | 743091 |  |  |  |  |  |  |  |  |  |  |  |  |  |  |
| 9 | Pc.c000803431.Contig1 | 2802 | - | 739719 | 742883 | cRMPc08035 | 35442 | 16 | 708253 | 743695 |  |  |  |  |  |  |  |  |  |
| 9 | MALPY00256 | 3872 | + | 743711 | 747729 |  |  |  |  |  | 54 | 40 | 4000 |  |  |  |  |  |  |
| 9 | Pc.c001003453.Contig1 | 3258 | - | 744041 | 747409 |  |  |  |  |  |  |  |  |  |  |  |  |  |  |
| 9 | Pb.c001904504.Contig1 | 4059 | - | 744826 | 749017 | cRMPc08036 | 5306 | 3305 | 743711 | 749017 |  |  |  |  |  |  |  |  |  |
| 9 | Pc.c000703458.Contig1 | 3110 | - | 752322 | 755402 |  |  |  |  |  |  |  |  |  |  |  |  |  |  |
| 9 | MALPY00059 | 7716 | + | 752356 | 760546 |  |  |  |  |  |  | 40 | 5000 |  |  |  |  |  |  |
| 9 | Pb.c001301811.Contig1 | 1834 | - | 755257 | 757490 |  |  |  |  |  |  |  |  |  |  |  |  |  |  |
| 9 | Pc.c001505142.Contig1 | 1973 | + | 757190 | 759323 |  |  |  |  |  |  |  |  |  |  |  |  |  |  |
| 9 | Pb.c001702286.Contig1 | 2742 | - | 757877 | 760607 |  |  |  |  |  |  |  |  |  |  |  |  |  |  |
| 9 | Pc.c000403241.Contig1 | 1015 | + | 759622 | 760637 | cRMPc08037 | 8315 | 2907 | 752322 | 760637 |  |  |  |  |  |  |  |  |  |
| 9 | Pc.c000502441.Contig2 | 8103 | - | 763544 | 771647 |  |  |  |  |  |  |  |  |  |  |  |  |  |  |
| 9 | MALPY01407 | 3286 | - | 768314 | 771600 |  |  |  |  |  |  | 40 | 900 |  |  |  |  |  |  |
| 9 | Pb.c002112847.Contig1 | 1014 | + | 770445 | 771444 | cRMPc08038 | 8103 | 865 | 763544 | 771647 |  |  |  |  |  |  |  |  |  |
| 9 | PbContig152 | 4273 | + | 772512 | 776989 |  |  |  |  |  |  |  |  |  |  |  |  |  |  |
| 9 | MALPY02458 | 3384 | - | 772637 | 776246 |  |  |  |  |  |  | 40 |  |  |  |  |  |  |  |
| 9 | Pc.c000202801.Contig1 | 1662 | - | 772997 | 774653 |  |  |  |  |  |  |  |  |  |  |  |  |  |  |
| 9 | Pc.c000303021.Contig1 | 1246 | - | 775065 | 776326 | cRMPc08039 | 4477 | 3734 | 772512 | 776989 |  |  |  | cRMPs0813 | 169816 | 3734 | 607173 | 776989 |  |
| 9 | Pb.c000801690.Contig1 | 4275 | - | 780723 | 784986 |  |  |  |  |  |  |  |  |  |  |  |  |  |  |
| 9 | Pc.c000602947.Contig1 | 2732 | - | 782189 | 784903 |  |  |  |  |  |  |  |  |  |  |  |  |  |  |
| 9 | MALPY00930 | 7718 | - | 782873 | 790498 |  |  |  |  |  | 1308 | 140 | 700 |  |  |  |  |  |  |
| 9 | Pb.c002113121.Contig1 | 1500 | - | 785376 | 786876 |  |  |  |  |  |  |  |  |  |  |  |  |  |  |
| 9 | Pb.c000901835.Contig1 | 986 | - | 789264 | 790251 |  |  |  |  |  |  |  |  |  |  |  |  |  |  |
| 9 | MALPY02578 | 7420 | + | 790248 | 798116 |  |  |  |  |  | 1308 | 140 | 850 |  |  |  |  |  |  |
| 9 | Pb.c000901836.Contig1 | 3067 | - | 790828 | 794408 |  |  |  |  |  |  |  |  |  |  |  |  |  |  |
| 9 | Pc.c001103666.Contig1 | 4898 | - | 793190 | 798267 |  |  |  |  |  |  |  |  |  |  |  |  |  |  |
| 9 | Pb.c001102002.Contig1 | 2375 | - | 794992 | 797373 | cRMPc08040 | 17544 | 309 | 780723 | 798267 |  |  |  |  |  |  |  |  |  |
| 9 | MALPY02605 | 6518 | - | 798576 | 805094 |  |  |  |  |  | 474 | 140 |  |  |  |  |  |  |  |
| 9 | PbContig194 | 9709 | - | 802385 | 812306 |  |  |  |  |  |  |  |  |  |  |  |  |  |  |
| 9 | PcContig417 | 8319 | + | 803319 | 811927 |  |  |  |  |  |  |  |  |  |  |  |  |  |  |
| 9 | MALPY02026 | 9616 | - | 805134 | 814818 |  |  |  |  |  | 474 |  |  |  |  |  |  |  |  |
| 9 | Pc.c000803840.Contig1 | 613 | + | 811947 | 812560 |  |  |  |  |  |  |  |  |  |  |  |  |  |  |
| 9 | MALPY01566 | 8199 | - | 814176 | 822861 |  |  |  |  |  | 474 |  |  |  |  |  |  |  |  |
| 9 | PbContig81 | 5960 | + | 815843 | 822291 |  |  |  |  |  |  |  |  |  |  |  |  |  |  |
| 9 | Pc.c000002512.Contig1 | 1849 | + | 817940 | 819792 |  |  |  |  |  |  |  |  |  |  |  |  |  |  |
| 9 | Pc.c000102501.Contig1 | 1356 | + | 820625 | 821984 | cRMPc08041 | 24285 | 2790 | 798576 | 822861 |  |  |  | cRMPs0814 | 42138 | 2790 | 780723 | 822861 |  |
| 9 | Pc.c000903332.Contig1 | 2280 | + | 825651 | 827751 |  |  |  |  |  |  |  |  |  |  |  |  |  |  |
| 9 | MALPY02481 | 3008 | + | 825844 | 828547 |  |  |  |  |  | 177 |  |  |  |  |  |  |  |  |
| 9 | Pb.c001301794.Contig1 | 7283 | - | 826889 | 834281 |  |  |  |  |  |  |  |  |  |  |  |  |  |  |
| 9 | Pc.c000102567.Contig1 | 2908 | + | 827652 | 830547 |  |  |  |  |  |  |  |  |  |  |  |  |  |  |
| 9 | MALPY00734 | 14240 | - | 828202 | 842947 |  |  |  |  |  | 177 |  |  |  |  |  |  |  |  |
| 9 | Pc.c000202894.Contig1 | 5065 | + | 831970 | 837489 |  |  |  |  |  |  |  |  |  |  |  |  |  |  |
| 9 | Pb.c001502887.Contig1 | 861 | + | 835407 | 836289 |  |  |  |  |  |  |  |  |  |  |  |  |  |  |
| 9 | Pb.c001301451.Contig1 | 4410 | + | 836926 | 841432 |  |  |  |  |  |  |  |  |  |  |  |  |  |  |
| 9 | Pc.c000302848.Contig1 | 3818 | + | 837715 | 841539 |  |  |  |  |  |  |  |  |  |  |  |  |  |  |
| 9 | Pc.c000303363.Contig1 | 1062 | + | 842257 | 843319 |  |  |  |  |  |  |  |  |  |  |  |  |  |  |
| 9 | MALPY02597 | 3861 | + | 843207 | 846939 |  |  |  |  |  | 177 |  |  |  |  |  |  |  |  |
| 9 | Pb.c001402071.Contig1 | 6205 | + | 846870 | 853081 |  |  |  |  |  |  |  |  |  |  |  |  |  |  |
| 9 | MALPY02370 | 5562 | + | 847865 | 853433 |  |  |  |  |  | 177 | 201 | 1000 |  |  |  |  |  |  |
| 9 | Pc.c001003499.Contig1 | 2605 | - | 849050 | 851664 | cRMPc08042 | 27782 | 1303 | 825651 | 853433 |  |  |  |  |  |  |  |  |  |
| 9 | PbContig320 | 5208 | + | 854736 | 859986 |  |  |  |  |  |  |  |  |  |  |  |  |  |  |
| 9 | MALPY00434 | 6394 | - | 854872 | 861405 |  |  |  |  |  |  | 201 | 3000 |  |  |  |  |  |  |
| 9 | Pc.c000602952.Contig1 | 3350 | + | 857503 | 860972 |  |  |  |  |  |  |  |  |  |  |  |  |  |  |
| 9 | Pb.c001502847.Contig1 | 1094 | + | 860082 | 861215 | cRMPc08043 | 6669 | 1722 | 854736 | 861405 |  |  |  |  |  |  |  |  |  |
| 9 | Pc.c000703370.Contig1 | 3492 | + | 863127 | 866650 |  |  |  |  |  |  |  |  |  |  |  |  |  |  |
| 9 | Pb.c001401744.Contig2 | 3312 | + | 863932 | 867221 |  |  |  |  |  |  |  |  |  |  |  |  |  |  |
| 9 | MALPY01687 | 14101 | - | 864439 | 879338 |  |  |  |  |  |  | 201 | 1400 |  |  |  |  |  |  |
| 9 | Pc.c000303459.Contig1 | 974 | - | 865926 | 866912 |  |  |  |  |  |  |  |  |  |  |  |  |  |  |
| 9 | Pc.c000402854.Contig1 | 2450 | - | 867194 | 869653 |  |  |  |  |  |  |  |  |  |  |  |  |  |  |
| 9 | Pb.c001803811.Contig1 | 1097 | + | 867870 | 868967 |  |  |  |  |  |  |  |  |  |  |  |  |  |  |
| 9 | Pb.c001501981.Contig1 | 4044 | - | 871018 | 875330 |  |  |  |  |  |  |  |  |  |  |  |  |  |  |
| 9 | Pc.c000002054.Contig2 | 5694 | - | 872910 | 878610 |  |  |  |  |  |  |  |  |  |  |  |  |  |  |
| 9 | Pb.c001803531.Contig1 | 2821 | + | 877369 | 880058 |  |  |  |  |  |  |  |  |  |  |  |  |  |  |
| 9 | Pc.c001404776.Contig1 | 1869 | - | 878981 | 880820 |  |  |  |  |  |  |  |  |  |  |  |  |  |  |
| 9 | Pb.c002007059.Contig1 | 2548 | + | 880388 | 882927 |  |  |  |  |  |  |  |  |  |  |  |  |  |  |
| 9 | MALPY01705 | 15418 | - | 880828 | 898407 |  |  |  |  |  |  | 201 | 2000 |  |  |  |  |  |  |
| 9 | Pc.c000302830.Contig1 | 3893 | - | 882503 | 886670 |  |  |  |  |  |  |  |  |  |  |  |  |  |  |
| 9 | Pb.c000501474.Contig1 | 2144 | + | 882922 | 885039 |  |  |  |  |  |  |  |  |  |  |  |  |  |  |
| 9 | Pb.c001904498.Contig1 | 4036 | + | 894146 | 898374 |  |  |  |  |  |  |  |  |  |  |  |  |  |  |
| 9 | Pc.c000502967.Contig1 | 595 | + | 896215 | 896810 |  |  |  |  |  |  |  |  |  |  |  |  |  |  |
| 9 | PcContig744 | 6335 | + | 896366 | 903096 |  |  |  |  |  |  |  |  |  |  |  |  |  |  |
| 9 | MALPY01660 | 3305 | + | 899542 | 903126 | cRMPc08044 | 39999 | 99 | 863127 | 903126 | 121 | 201 |  |  |  |  |  |  |  |
| 9 | MALPY01661 | 5617 | - | 903225 | 908971 |  |  |  |  |  | 121 | 204 | 5000 |  |  |  |  |  |  |
| 9 | Pc.c001003999.Contig1 | 4631 | + | 904638 | 909347 |  |  |  |  |  |  |  |  |  |  |  |  |  |  |
| 9 | Pb.c000201291.Contig1 | 7606 | + | 904859 | 912622 |  |  |  |  |  |  |  |  |  |  |  |  |  |  |
| man9 | MALPY02394 | 2431 | + | 910255 | 912686 | cRMPc08045 | 9461 | 3170 | 903225 | 912686 |  |  |  |  |  |  |  |  | Pb.c000201291.Contig1 |
| man9 | PcContig4067 | 1369 | - | 915856 | 917225 |  |  |  |  |  |  |  |  |  |  |  |  |  | MALPY01744 |
| 9 | MALPY01744 | 3715 | + | 916817 | 920532 |  |  |  |  |  |  | 204 | 3500 |  |  |  |  |  |  |
| 9 | Pb.c000801706.Contig1 | 3645 | + | 917541 | 921186 |  |  |  |  |  |  |  |  |  |  |  |  |  |  |
| 9 | PcContig335 | 3364 | + | 918042 | 921406 |  |  |  |  |  |  |  |  |  |  |  |  |  |  |
| TK9 | MALPY00723 | 3991 | - | 921058 | 925642 | cRMPc08046 | 9786 | 531 | 915856 | 925642 | 161 |  |  |  |  |  |  |  |  |
| 9 | MALPY01053 | 15293 | + | 926173 | 942726 |  |  |  |  |  | 161 | 204 | 2600 |  |  |  |  |  |  |
| 9 | PcContig428 | 7712 | + | 926365 | 934761 |  |  |  |  |  |  |  |  |  |  |  |  |  |  |
| 9 | Pb.c000201577.Contig1 | 6885 | + | 929589 | 937335 |  |  |  |  |  |  |  |  |  |  |  |  |  |  |
| 9 | Pc.c001505514.Contig1 | 945 | + | 936037 | 937035 | cRMPc08047 | 16553 | 4108 | 926173 | 942726 |  |  |  |  |  |  |  |  |  |
| 9 | MALPY00684 | 5799 | + | 946834 | 952868 |  |  |  |  |  |  | 90/204 | 4000 |  |  |  |  |  |  |
| 9 | Pb.c001702861.Contig1 | 2418 | + | 949857 | 952298 |  |  |  |  |  |  |  |  |  |  |  |  |  |  |
| 9 | Pc.c001505752.Contig1 | 1281 | - | 950052 | 951365 |  |  |  |  |  |  |  |  |  |  |  |  |  |  |
| 9 | Pc.c000703339.Contig1 | 1973 | - | 951582 | 953637 | cRMPc08048 | 6803 | 583 | 946834 | 953637 |  |  |  |  |  |  |  |  |  |
| 9 | MALPY01448 | 3484 | + | 954220 | 957674 | cRMPc08049 | 3454 | 1457 | 954220 | 957674 |  |  |  |  |  |  |  |  |  |
| 9 | MALPY01436 | 5455 | + | 959131 | 964550 |  |  |  |  |  | 615 | 90 |  |  |  |  |  |  |  |
| 9 | PcContig308 | 4486 | + | 960099 | 964699 |  |  |  |  |  |  |  |  |  |  |  |  |  |  |
| 9 | Pb.c001502449.Contig1 | 2998 | + | 960175 | 963212 | cRMPc08050 | 5568 | 68 | 959131 | 964699 |  |  |  |  |  |  |  |  |  |
| 9 | MALPY03137 | 1982 | - | 964767 | 966746 |  |  |  |  |  | 615 | 94 | 1500 |  |  |  |  |  |  |
| 9 | Pb.c002113218.Contig1 | 614 | - | 966258 | 966869 |  |  |  |  |  |  |  |  |  |  |  |  |  |  |
| 9 | Pc.c001606186.Contig1 | 1766 | + | 966613 | 968379 |  |  |  |  |  |  |  |  |  |  |  |  |  |  |
| 9 | Pb.c001602439.Contig1 | 10411 | - | 967501 | 978328 |  |  |  |  |  |  |  |  |  |  |  |  |  |  |
| 9 | MALPY00050 | 11456 | - | 967746 | 979313 |  |  |  |  |  |  | 94 | 1000 |  |  |  |  |  |  |
| 9 | Pc.c001304244.Contig1 | 5786 | + | 969866 | 975969 |  |  |  |  |  |  |  |  |  |  |  |  |  |  |
| 9 | Pc.c000402705.Contig1 | 4792 | - | 979090 | 983881 |  |  |  |  |  |  |  |  |  |  |  |  |  |  |
| 9 | Pb.c000801794.Contig1 | 4658 | - | 979579 | 984237 |  |  |  |  |  |  |  |  |  |  |  |  |  |  |
| 9 | MALPY02095 | 4183 | + | 980813 | 984996 |  |  |  |  |  |  | 94 |  |  |  |  |  |  |  |
| 9 | Pb.c000801418.Contig1 | 2859 | - | 984763 | 987257 |  |  |  |  |  |  |  |  |  |  |  |  |  |  |
| 9 | Pc.c000503049.Contig1 | 1904 | - | 984778 | 986682 |  |  |  |  |  |  |  |  |  |  |  |  |  |  |
| 9 | MALPY01398 | 8264 | - | 985731 | 993727 |  |  |  |  |  |  | 105 | 150 |  |  |  |  |  |  |
| 9 | PcContig52 | 4952 | + | 986351 | 991318 |  |  |  |  |  |  |  |  |  |  |  |  |  |  |
| 9 | Pb.c001502534.Contig1 | 10847 | - | 987411 | 998996 |  |  |  |  |  |  |  |  |  |  |  |  |  |  |
| 9 | PcContig220 | 3850 | + | 991393 | 995996 |  |  |  |  |  |  |  |  |  |  |  |  |  |  |
| 9 | MALPY01441 | 3676 | - | 993865 | 997389 |  |  |  |  |  | 93 | 105 |  |  |  |  |  |  |  |
| 9 | Pc.c001504220.Contig2 | 1807 | + | 996098 | 997751 |  |  |  |  |  |  |  |  |  |  |  |  |  |  |
| 9 | MALPY03261 | 1749 | - | 997275 | 999046 |  |  |  |  |  | 93 |  |  |  |  |  |  |  |  |
| 9 | PcContig270 | 1196 | + | 997709 | 998992 | cRMPc08051 | 34279 | 15176 | 964767 | 999046 |  |  |  | cRMPs0815 | 173395 | 15176 | 825651 | 999046 |  |
| 9 | Pb.c001702806.Contig1 | 4949 | - | 1014222 | 1019378 |  |  |  |  |  |  |  |  |  |  |  |  |  |  |
| 9 | MALPY00665 | 5454 | - | 1014263 | 1019791 |  |  |  |  |  |  | 181 | 200 |  |  |  |  |  |  |
| 9 | Pc.c000202826.Contig1 | 1689 | + | 1014264 | 1015953 |  |  |  |  |  |  |  |  |  |  |  |  |  |  |
| 9 | Pc.c001404759.Contig1 | 3006 | + | 1016067 | 1019357 | cRMPc08052 | 5569 | 374 | 1014222 | 1019791 |  |  |  |  |  |  |  |  |  |
| 9 | MALPY03238 | 619 | + | 1020165 | 1020769 | cRMPc08053 | 604 | 635 | 1020165 | 1020769 |  | 181 | 900 |  |  |  |  |  |  |
| 9 | MALPY01637 | 7371 | + | 1021404 | 1028611 |  |  |  |  |  | 1118 | 181 |  |  |  |  |  |  |  |
| 9 | Pb.c001502393.Contig1 | 6430 | - | 1022447 | 1028701 |  |  |  |  |  |  |  |  |  |  |  |  |  |  |
| 9 | Pc.c001404840.Contig1 | 3309 | + | 1025956 | 1029265 |  |  |  |  |  |  |  |  |  |  |  |  |  |  |
| 9 | MALPY00659 | 14426 | + | 1028969 | 1042659 |  |  |  |  |  | 1118 |  |  |  |  |  |  |  |  |
| 9 | PbContig74 | 13997 | + | 1032955 | 1047430 |  |  |  |  |  |  |  |  |  |  |  |  |  |  |
| 9 | Pc.c001605923.Contig1 | 1218 | + | 1034873 | 1036091 |  |  |  |  |  |  |  |  |  |  |  |  |  |  |
| 9 | Pc.c000002572.Contig1 | 5535 | + | 1036485 | 1041837 |  |  |  |  |  |  |  |  |  |  |  |  |  |  |
| 9 | MALPY00004 | 2552 | - | 1043517 | 1045935 |  |  |  |  |  |  | 30 | 2500 |  |  |  |  |  |  |
| 9 | Pc.c000903298.Contig1 | 2497 | - | 1043848 | 1046252 | cRMPc08054 | 26026 | 1783 | 1021404 | 1047430 |  |  |  |  |  |  |  |  |  |
| 9 | MALPY02304 | 6449 | - | 1049213 | 1056436 |  |  |  |  |  |  | 30 | 4000 |  |  |  |  |  |  |
| 9 | Pb.c001401750.Contig1 | 8901 | + | 1049402 | 1058966 |  |  |  |  |  |  |  |  |  |  |  |  |  |  |
| 9 | Pc.c000602349.Contig1 | 4258 | - | 1049739 | 1054788 | cRMPc08055 | 9753 | 1603 | 1049213 | 1058966 |  |  |  |  |  |  |  |  |  |
| 9 | MALPY01644 | 12793 | + | 1060569 | 1075548 |  |  |  |  |  |  | 30 |  |  |  |  |  |  |  |
| 9 | Pb.c002007663.Contig1 | 981 | + | 1060806 | 1061940 |  |  |  |  |  |  |  |  |  |  |  |  |  |  |
| 9 | Pb.c000101427.Contig1 | 2137 | + | 1063134 | 1065346 |  |  |  |  |  |  |  |  |  |  |  |  |  |  |
| 9 | Pc.c001003717.Contig1 | 2264 | - | 1073196 | 1075736 | cRMPc08056 | 15167 | 812 | 1060569 | 1075736 |  |  |  | cRMPs0816 | 61514 | 812 | 1014222 | 1075736 |  |
| TK9 | MALPY01703 | 7159 | + | 1076548 | 1084433 | cRMPc08057 | 7885 | 314 | 1076548 | 1084433 | 56 |  |  |  |  |  |  |  |  |
| 9 | MALPY02643 | 5016 | - | 1084747 | 1089805 |  |  |  |  |  | 56 |  |  |  |  |  |  |  |  |
| 9 | Pb.c001803819.Contig1 | 1568 | + | 1086072 | 1087670 | cRMPc08058 | 5058 | 1138 | 1084747 | 1089805 |  |  |  |  |  |  |  |  |  |
| 9 | MALPY00249 | 12058 | - | 1090943 | 1103476 |  |  |  |  |  | 56 |  |  |  |  |  |  |  |  |
| 9 | PcContig177 | 7710 | - | 1094643 | 1103020 |  |  |  |  |  |  |  |  |  |  |  |  |  |  |
| 9 | PbContig197 | 15036 | - | 1096184 | 1111173 |  |  |  |  |  |  |  |  |  |  |  |  |  |  |
| 9 | MALPY01156 | 13190 | - | 1102604 | 1115721 |  |  |  |  |  | 56 |  |  |  |  |  |  |  |  |
| 9 | PcContig221 | 6256 | - | 1103161 | 1109122 | cRMPc08059 | 24778 | 1550 | 1090943 | 1115721 |  |  |  |  |  |  |  |  |  |
| 9 | MALPY00206 | 22851 | - | 1117271 | 1139640 |  |  |  |  |  | 56 |  |  |  |  |  |  |  |  |
| 9 | Pb.c001702859.Contig1 | 3396 | - | 1117782 | 1121178 |  |  |  |  |  |  |  |  |  |  |  |  |  |  |
| 9 | Pb.c000701493.Contig1 | 2177 | - | 1121895 | 1124066 |  |  |  |  |  |  |  |  |  |  |  |  |  |  |
| 9 | Pc.c001505364.Contig1 | 615 | + | 1121976 | 1122591 |  |  |  |  |  |  |  |  |  |  |  |  |  |  |
| 9 | PcContig195 | 5083 | + | 1122632 | 1127715 |  |  |  |  |  |  |  |  |  |  |  |  |  |  |
| 9 | Pb.c000901463.Contig1 | 7683 | - | 1123755 | 1131438 |  |  |  |  |  |  |  |  |  |  |  |  |  |  |
| 9 | MALPY03075 | 510 | - | 1126782 | 1127292 | cRMPc08060 | 22369 | 110 | 1117271 | 1139640 |  |  |  | cRMPs0817 | 63092 | 110 | 1076548 | 1139640 |  |
| 9 | Pb.c000701442.Contig1 | 17892 | + | 1139750 | 1160653 |  |  |  |  |  |  |  |  |  |  |  |  |  |  |
| 9 | MALPY01319 | 12772 | + | 1141260 | 1154485 |  |  |  |  |  | 17 |  |  |  |  |  |  |  |  |
| 9 | Pc.c000703802.Contig1 | 1195 | + | 1143273 | 1144468 |  |  |  |  |  |  |  |  |  |  |  |  |  |  |
| 9 | Pc.c000602336.Contig1 | 2197 | + | 1144849 | 1147046 |  |  |  |  |  |  |  |  |  |  |  |  |  |  |
| 9 | PcContig520 | 905 | + | 1147091 | 1147996 |  |  |  |  |  |  |  |  |  |  |  |  |  |  |
| 9 | Pc.c000002873.Contig1 | 5852 | + | 1148414 | 1154336 |  |  |  |  |  |  |  |  |  |  |  |  |  |  |
| 9 | MALPY00072 | 21482 | + | 1155271 | 1177406 |  |  |  |  |  | 17 |  |  |  |  |  |  |  |  |
| 9 | Pc.c000603437.Contig1 | 1114 | + | 1156962 | 1158076 |  |  |  |  |  |  |  |  |  |  |  |  |  |  |
| 9 | Pb.c001502091.Contig2 | 3544 | + | 1164525 | 1168049 |  |  |  |  |  |  |  |  |  |  |  |  |  |  |
| 9 | PcContig110 | 6645 | + | 1165598 | 1172647 |  |  |  |  |  |  |  |  |  |  |  |  |  |  |
| 9 | PbContig30 | 5668 | + | 1168273 | 1173914 |  |  |  |  |  |  |  |  |  |  |  |  |  |  |
| 9 | Pb.c001001710.Contig1 | 1870 | + | 1174591 | 1176493 |  |  |  |  |  |  |  |  |  |  |  |  |  |  |
| 9 | Pc.c000403297.Contig1 | 602 | - | 1175422 | 1176024 |  |  |  |  |  |  |  |  |  |  |  |  |  |  |
| 9 | Pc.c000302921.Contig1 | 3523 | - | 1176232 | 1179803 |  |  |  |  |  |  |  |  |  |  |  |  |  |  |
| 9 | Pb.c001201676.Contig2 | 6482 | + | 1176699 | 1183005 |  |  |  |  |  |  |  |  |  |  |  |  |  |  |
| 9 | MALPY00817 | 12652 | - | 1177420 | 1188982 |  |  |  |  |  | 17 |  |  |  |  |  |  |  |  |
| 9 | Pb.c000601575.Contig1 | 2243 | + | 1184766 | 1186966 |  |  |  |  |  |  |  |  |  |  |  |  |  |  |
| 9 | Pc.c000002511.Contig1 | 3228 | - | 1184823 | 1188019 | cRMPc08061 | 49232 | 1035 | 1139750 | 1188982 |  |  |  |  |  |  |  |  |  |
| 9 | PcContig317 | 6553 | + | 1190017 | 1197132 |  |  |  |  |  |  |  |  |  |  |  |  |  |  |
| 9 | MALPY01638 | 5812 | + | 1190046 | 1195918 |  |  |  |  |  | 17 | 167 | 3000 |  |  |  |  |  |  |
| 9 | Pb.c001602383.Contig1 | 8050 | - | 1190862 | 1199389 |  |  |  |  |  |  |  |  |  |  |  |  |  |  |
| 9 | Pc.c000903425.Contig1 | 1919 | + | 1197088 | 1199007 |  |  |  |  |  |  |  |  |  |  |  |  |  |  |
| 9 | MALPY03080 | 671 | - | 1198545 | 1199216 | cRMPc08062 | 9372 | 1669 | 1190017 | 1199389 |  | 167 | 1400 |  |  |  |  |  |  |
| 9 | MALPY00126 | 4999 | - | 1201058 | 1206345 |  |  |  |  |  |  | 167 |  |  |  |  |  |  |  |
| 9 | Pb.c000701567.Contig1 | 1601 | + | 1201078 | 1202679 |  |  |  |  |  |  |  |  |  |  |  |  |  |  |
| 9 | Pc.c001304243.Contig1 | 1222 | - | 1201872 | 1203094 |  |  |  |  |  |  |  |  |  |  |  |  |  |  |
| 9 | PbContig403 | 11889 | + | 1202935 | 1217460 |  |  |  |  |  |  |  |  |  |  |  |  |  |  |
| 9 | Pc.c000403327.Contig1 | 1285 | - | 1203550 | 1204838 |  |  |  |  |  |  |  |  |  |  |  |  |  |  |
| 9 | MALPY03018 | 668 | + | 1203686 | 1204354 |  |  |  |  |  |  |  |  |  |  |  |  |  |  |
| 9 | Pc.c000503515.Contig1 | 711 | - | 1205219 | 1205929 |  |  |  |  |  |  |  |  |  |  |  |  |  |  |
| 9 | MALPY03227 | 1159 | - | 1206540 | 1207746 |  |  |  |  |  | 35 |  |  |  |  |  |  |  |  |
| 9 | MALPY01796 | 5687 | - | 1207722 | 1215783 |  |  |  |  |  | 35 |  |  |  |  |  |  |  |  |
| 9 | MALPY03214 | 299 | + | 1207875 | 1208174 |  |  |  |  |  |  | 153 | 5000 |  |  |  |  |  |  |
| 9 | PcContig320 | 3316 | - | 1208783 | 1212114 |  |  |  |  |  |  |  |  |  |  |  |  |  |  |
| 9 | Pc.c000303436.Contig1 | 976 | - | 1214682 | 1215658 |  |  |  |  |  |  |  |  |  |  |  |  |  |  |
| 9 | MALPY03407 | 1726 | + | 1215317 | 1217043 | cRMPc08063 | 16402 | 1070 | 1201058 | 1217460 | 35 | 153 |  | cRMPs0818 | 77710 | 1070 | 1139750 | 1217460 |  |
| 9 | Pc.c000703473.Contig1 | 4768 | - | 1218530 | 1223277 |  |  |  |  |  |  |  |  |  |  |  |  |  |  |
| 9 | Pb.c000701591.Contig1 | 3956 | + | 1220163 | 1224122 |  |  |  |  |  |  |  |  |  |  |  |  |  |  |
| 9 | MALPY01170 | 3751 | + | 1222304 | 1226058 |  |  |  |  |  |  |  |  |  |  |  |  |  |  |
| 9 | Pc.c000602871.Contig1 | 2915 | - | 1223608 | 1226523 | cRMPc08064 | 7993 | 2227 | 1218530 | 1226523 |  |  |  | cRMPs0819 | 7993 | 2227 | 1218530 | 1226523 |  |
| 9 | MALPY01250 | 14180 | - | 1228750 | 1242872 |  |  |  |  |  |  |  |  |  |  |  |  |  |  |
| 9 | Pb.c001301829.Contig1 | 4717 | + | 1235766 | 1240468 |  |  |  |  |  |  |  |  |  |  |  |  |  |  |
| 9 | Pc.c001203960.Contig1 | 2027 | + | 1238775 | 1240787 | cRMPc08065 | 14122 | 2391 | 1228750 | 1242872 |  |  |  | cRMPs0820 | 14122 | 2391 | 1228750 | 1242872 |  |
| 9 | MALPY01879 | 3217 | + | 1245263 | 1248492 | cRMPc08066 | 3229 | 1715 | 1245263 | 1248492 |  | 219 | 1100 |  |  |  |  |  |  |
| 9 | MALPY01675 | 14882 | - | 1250207 | 1265004 |  |  |  |  |  | 327 | 219 |  |  |  |  |  |  |  |
| 9 | Pb.c000801728.Contig1 | 4270 | - | 1253576 | 1257846 |  |  |  |  |  |  |  |  |  |  |  |  |  |  |
| 9 | Pc.c001003234.Contig1 | 7804 | + | 1255207 | 1263134 |  |  |  |  |  |  |  |  |  |  |  |  |  |  |
| 9 | PbContig130 | 6496 | + | 1258550 | 1264685 |  |  |  |  |  |  |  |  |  |  |  |  |  |  |
| 9 | Pc.c001304710.Contig1 | 2872 | + | 1263312 | 1266150 |  |  |  |  |  |  |  |  |  |  |  |  |  |  |
| 9 | Pb.c001602398.Contig1 | 916 | + | 1264568 | 1265484 |  |  |  |  |  |  |  |  |  |  |  |  |  |  |
| 9 | MALPY01292 | 7801 | + | 1264706 | 1273337 |  |  |  |  |  | 327 |  |  |  |  |  |  |  |  |
| 9 | PcContig441 | 7872 | + | 1266557 | 1275072 |  |  |  |  |  |  |  |  |  |  |  |  |  |  |
| 9 | MALPY02307 | 3856 | - | 1273453 | 1277270 |  |  |  |  |  |  |  |  |  |  |  |  |  |  |
| 9 | Pc.c001505406.Contig1 | 1152 | + | 1275262 | 1276396 | cRMPc08067 | 27063 | 7226 | 1250207 | 1277270 |  |  |  | cRMPs0821 | 32007 | 7226 | 1245263 | 1277270 |  |
| 9 | Pc.c000203042.Contig1 | 6230 | + | 1284496 | 1291241 |  |  |  |  |  |  |  |  |  |  |  |  |  |  |
| 9 | MALPY00509 | 6025 | - | 1286524 | 1293114 |  |  |  |  |  |  |  |  |  |  |  |  |  |  |
| 9 | Pb.c001101964.Contig1 | 6628 | + | 1287029 | 1294444 |  |  |  |  |  |  |  |  |  |  |  |  |  |  |
| 9 | MALPY03250 | 783 | - | 1289126 | 1289909 | cRMPc08068 | 9948 | 738 | 1284496 | 1294444 |  | 75 | 5000 |  |  |  |  |  |  |
| 9 | PbContig256 | 4900 | - | 1295182 | 1300066 |  |  |  |  |  |  |  |  |  |  |  |  |  |  |
| 9 | MALPY00903 | 7482 | + | 1295472 | 1303460 | cRMPc08069 | 8278 | 8988 | 1295182 | 1303460 | 330A | 75 | 6000 |  |  |  |  |  |  |
| 9 | MALPY02081 | 6056 | - | 1312448 | 1318651 |  |  |  |  |  |  | 75 | 2200 |  |  |  |  |  |  |
| 9 | Pb.c001502466.Contig1 | 1767 | + | 1314024 | 1315797 | cRMPc08070 | 6203 | 2200 | 1312448 | 1318651 |  |  |  |  |  |  |  |  |  |
| Centromere - PCR 2200 bp | |  |  | 1320000 |  |  |  |  |  |  |  |  |  |  |  |  |  |  |  |
| 9 | MALPY02415 | 6459 | + | 1320851 | 1327310 |  |  |  |  |  | 157A | 75 | 1200 |  |  |  |  |  |  |
| 9 | Pb.c000301388.Contig1 | 3142 | - | 1324963 | 1328088 |  |  |  |  |  |  |  |  |  |  |  |  |  |  |
| 9 | MALPY03452 | 851 | - | 1327204 | 1328052 | cRMPc08071 | 7237 | 251 | 1320851 | 1328088 |  | 75 | 750 |  |  |  |  |  |  |
| 9 | MALPY02813 | 2792 | - | 1328339 | 1331122 |  |  |  |  |  |  | 75 |  |  |  |  |  |  |  |
| 9 | Pc.c000603456.Contig1 | 2411 | + | 1328882 | 1331347 |  |  |  |  |  |  |  |  |  |  |  |  |  |  |
| 9 | Pb.c001904396.Contig1 | 1466 | + | 1329022 | 1330509 |  |  |  |  |  |  |  |  |  |  |  |  |  |  |
| 9 | MALPY03421 | 292 | + | 1329575 | 1329864 | cRMPc08072 | 3008 | 1640 | 1328339 | 1331347 |  |  |  | cRMPs0822 | 46851 | 1640 | 1284496 | 1331347 |  |
| 9 | MALPY01785 | 2483 | - | 1332987 | 1335473 | cRMPc08073 | 2486 | 4492 | 1332987 | 1335473 |  |  |  | cRMPs0823 | 2486 | 4492 | 1332987 | 1335473 |  |
| 9 | MALPY02858 | 3635 | - | 1339965 | 1343600 |  |  |  |  |  |  |  |  |  |  |  |  |  |  |
| 9 | Pc.c001813106.Contig1 | 486 | - | 1342763 | 1343249 | cRMPc08074 | 3635 | 3804 | 1339965 | 1343600 |  |  |  | cRMPs0824 | 3635 | 3804 | 1339965 | 1343600 |  |
| 9 | Pc.c001103748.Contig1 | 3121 | + | 1347404 | 1350691 |  |  |  |  |  |  |  |  |  |  |  |  |  |  |
| 9 | Pb.c000701480.Contig1 | 6580 | + | 1348502 | 1355355 |  |  |  |  |  |  |  |  |  |  |  |  |  |  |
| 9 | MALPY00917 | 20040 | - | 1348592 | 1370144 |  |  |  |  |  | 719 |  |  |  |  |  |  |  |  |
| 9 | Pc.c001405498.Contig1 | 1952 | + | 1351192 | 1353165 |  |  |  |  |  |  |  |  |  |  |  |  |  |  |
| 9 | Pc.c000902739.Contig1 | 3895 | - | 1353681 | 1357540 |  |  |  |  |  |  |  |  |  |  |  |  |  |  |
| 9 | Pb.c001402069.Contig1 | 1927 | + | 1356729 | 1358662 |  |  |  |  |  |  |  |  |  |  |  |  |  |  |
| 9 | Pc.c000102026.Contig2 | 5103 | - | 1358012 | 1364154 |  |  |  |  |  |  |  |  |  |  |  |  |  |  |
| 9 | PbContig378 | 3549 | + | 1358732 | 1363350 |  |  |  |  |  |  |  |  |  |  |  |  |  |  |
| 9 | Pb.c000201306.Contig2 | 4172 | + | 1364231 | 1368553 |  |  |  |  |  |  |  |  |  |  |  |  |  |  |
| 9 | Pc.c000503140.Contig1 | 4203 | + | 1365320 | 1369918 |  |  |  |  |  |  |  |  |  |  |  |  |  |  |
| 9 | MALPY03495 | 670 | + | 1368598 | 1369430 |  |  |  |  |  |  |  |  |  |  |  |  |  |  |
| 9 | MALPY03584 | 635 | + | 1368606 | 1369403 |  |  |  |  |  |  |  |  |  |  |  |  |  |  |
| 9 | Pb.c000701601.Contig1 | 1775 | + | 1368616 | 1370594 |  |  |  |  |  |  |  |  |  |  |  |  |  |  |
| 9 | MALPY01061 | 6649 | + | 1370029 | 1377109 | cRMPc08075 | 29705 | 1062 | 1347404 | 1377109 | 719 | 4 | 8000 |  |  |  |  |  |  |
| TK9 | MALPY02669 | 3962 | - | 1378171 | 1382713 |  |  |  |  |  | 194 |  |  |  |  |  |  |  |  |
| 9 | Pc.c001204266.Contig1 | 637 | - | 1381850 | 1382490 |  |  |  |  |  |  |  |  |  |  |  |  |  |  |
| 9 | Pc.c001404773.Contig1 | 2244 | - | 1381909 | 1384265 |  |  |  |  |  |  |  |  |  |  |  |  |  |  |
| 9 | MALPY01530 | 3125 | - | 1382775 | 1385995 |  |  |  |  |  | 194 | 4 | 2000 |  |  |  |  |  |  |
| 9 | Pb.c001602451.Contig1 | 2180 | + | 1385790 | 1387970 |  |  |  |  |  |  |  |  |  |  |  |  |  |  |
| 9 | Pc.c000202676.Contig1 | 7194 | + | 1385992 | 1392929 |  |  |  |  |  |  |  |  |  |  |  |  |  |  |
| 9 | MALPY00716 | 5193 | - | 1386922 | 1392118 |  |  |  |  |  | 117A | 4 |  |  |  |  |  |  |  |
| 9 | Pb.c001803504.Contig1 | 2828 | + | 1387892 | 1390723 |  |  |  |  |  |  |  |  |  |  |  |  |  |  |
| TK9 | MALPY02183 | 2939 | + | 1392196 | 1395133 |  |  |  |  |  | 117A |  |  |  |  |  |  |  |  |
| man9 | Pb.c000401448.Contig1 | 2451 | + | 1394905 | 1398099 | cRMPc08076 | 19928 | 2269 | 1378171 | 1398099 |  |  |  | cRMPs0825 | 50695 | 2269 | 1347404 | 1398099 |  |
| 9 | Pb.c000301142.Contig2 | 6021 | + | 1400368 | 1406708 |  |  |  |  |  |  |  |  |  |  |  |  |  |  |
| TK9 | MALPY01168 | 3890 | + | 1401224 | 1404861 |  |  |  |  |  | 38B |  |  |  |  |  |  |  |  |
| 9 | MALPY00498 | 10714 | - | 1404882 | 1415840 |  |  |  |  |  | 38B |  |  |  |  |  |  |  |  |
| 9 | Pc.c001812395.Contig1 | 528 | + | 1404953 | 1405481 |  |  |  |  |  |  |  |  |  |  |  |  |  |  |
| 9 | Pc.c000602332.Contig1 | 5878 | + | 1406202 | 1412645 |  |  |  |  |  |  |  |  |  |  |  |  |  |  |
| 9 | Pb.c001502043.Contig2 | 10037 | + | 1407947 | 1417996 |  |  |  |  |  |  |  |  |  |  |  |  |  |  |
| 9 | MALPY03076 | 256 | - | 1410092 | 1410348 |  |  |  |  |  |  |  |  |  |  |  |  |  |  |
| 9 | Pc.c001504186.Contig1 | 6191 | + | 1414101 | 1420485 |  |  |  |  |  |  |  |  |  |  |  |  |  |  |
| 9 | MALPY00197 | 9479 | - | 1415832 | 1425700 |  |  |  |  |  | 38B |  |  |  |  |  |  |  |  |
| 9 | Pb.c001401779.Contig1 | 4288 | + | 1418314 | 1423220 |  |  |  |  |  |  |  |  |  |  |  |  |  |  |
| 9 | PcContig617 | 7067 | + | 1421290 | 1428724 |  |  |  |  |  |  |  |  |  |  |  |  |  |  |
| 9 | PbContig270 | 7506 | + | 1423618 | 1431387 |  |  |  |  |  |  |  |  |  |  |  |  |  |  |
| 9 | Pc.c000803602.Contig1 | 601 | + | 1424935 | 1425536 |  |  |  |  |  |  |  |  |  |  |  |  |  |  |
| 9 | MALPY02032 | 6969 | + | 1425030 | 1432011 |  |  |  |  |  | 38B |  |  |  |  |  |  |  |  |
| 9 | Pc.c000102682.Contig1 | 3704 | + | 1428744 | 1432545 | cRMPc08077 | 32177 | 2073 | 1400368 | 1432545 |  |  |  |  |  |  |  |  |  |
| 9 | MALPY01654 | 9839 | - | 1434618 | 1444676 |  |  |  |  |  | 38B | 195 | 1400 |  |  |  |  |  |  |
| 9 | Pc.c000002720.Contig1.2 | 680 | + | 1436571 | 1437251 |  |  |  |  |  |  |  |  |  |  |  |  |  |  |
| 9 | Pc.c000602339.Contig2 | 5193 | - | 1438746 | 1444168 |  |  |  |  |  |  |  |  |  |  |  |  |  |  |
| 9 | Pb.c001502526.Contig1 | 2354 | + | 1440697 | 1443213 |  |  |  |  |  |  |  |  |  |  |  |  |  |  |
| 9 | MALPY03538 | 437 | - | 1440810 | 1441247 |  |  |  |  |  |  |  |  |  |  |  |  |  |  |
| 9 | Pb.c001502488.Contig1 | 2803 | - | 1444479 | 1447413 |  |  |  |  |  |  |  |  |  |  |  |  |  |  |
| 9 | MALPY00316 | 7513 | + | 1445106 | 1452210 |  |  |  |  |  |  | 195 |  |  |  |  |  |  |  |
| 9 | Pc.c000903468.Contig1 | 5074 | + | 1445861 | 1450878 |  |  |  |  |  |  |  |  |  |  |  |  |  |  |
| 9 | Pb.c000801783.Contig1 | 5352 | + | 1447176 | 1452669 |  |  |  |  |  |  |  |  |  |  |  |  |  |  |
| man9 | PbContig4496 | 5551 | - | 1447198 | 1452749 |  |  |  |  |  |  |  |  |  |  |  |  |  | MALPY00316 |
| man9 | PcContig6939 | 4956 | + | 1450743 | 1455699 |  |  |  |  |  |  |  |  |  |  |  |  |  | \| |
| man9 | MALPY02041 | 9929 | + | 1453191 | 1463120 | cRMPc08078 | 28502 |  | 1434618 | 1463120 |  |  |  | cRMPs0826 | 62752 |  | 1400368 | 1463120 | \| |
| Telomere | |  |  |  |  |  |  |  |  |  |  |  |  |  |  |  |  |  |  |
|  |  | Pb Pc Py contig |  |  |  |  | cRMP contig | Gap to next cRMP contig |  |  |  |  |  |  | cRMP scaffold | Gap to next cRMP scaffold |  |  |  |
|  | Number | 516 |  |  |  | Number | 78 | 77 |  |  |  |  |  | Number | 26 | 25 |  |  |  |
|  | Average size (bp) | 4897 |  |  |  | Average size (bp) | 16439 | 2349 |  |  |  |  |  | Average size (bp) | 52530 | 3894 |  |  |  |
|  | Minimum size (bp) | 243 |  |  |  | Minimum size (bp) | 604 | 16 |  |  |  |  |  | Minimum size (bp) | 1399 | 110 |  |  |  |
|  | Maximum size (bp) | 80019 |  |  |  | Maximum size (bp) | 108964 | 15176 |  |  |  |  |  | Maximum size (bp) | 173395 | 15176 |  |  |  |
|  | Total size (bp) | 2527083 |  |  |  | Total size (bp) | 1282216 | 180904 |  |  |  |  |  | Total size (bp) | 1365776 | 97344 |  |  |  |
|  |  |  |  |  |  |  |  |  |  |  |  |  |  |  |  |  |  |  |  |
