# Supplementary material for: A Plasmodium Whole-Genome Synteny Map: Indels and Synteny Breakpoints as Foci for Species-Specific Genes
Source: PLoS Pathog. 2005 Dec 23;1(4):e44. doi: 10.1371/journal.ppat.0010044 (PMC1317653; doi:10.1371/journal.ppat.0010044)
Supplement: Supplementary file 9 [file ppat.0010044.st025.doc]

Table S25 - RMPchr9


# Table S25 - RMPchr9

|  |  |  |  |  |  |  |  |  |  |  |  |  |  |  |  |  |  |  |  |
| --- | --- | --- | --- | --- | --- | --- | --- | --- | --- | --- | --- | --- | --- | --- | --- | --- | --- | --- | --- |
| Table S25: Tiling path of RMP contigs combined to form cRMP chromosome 9. | | | | | | |  |  |  |  |  |  |  |  |  |  |  |  |  |
|  |  |  |  |  |  |  |  |  |  |  |  |  |  |  |  |  |  |  |  |
| Tiling paths of P. berghei (Pb), P. c. chabaudi (Pc) and P. y. yoelii (Py) contigs combined into composite rodent malaria parasite (cRMP) | | | | | | | | | |  |  |  |  |  |  |  |  |  |  |
| contigs aligned against parts of the 14 chromosomes of P. falciparum (Pf). The cRMP contigs are further linked by physical linkage | | | | | | | | | |  |  |  |  |  |  |  |  |  |  |
| and PCR (see Methods) to form cRMP scaffolds. Different synteny blocks with homology to different P. falciparum chromosomes | | | | | | | | | |  |  |  |  |  |  |  |  |  |  |
| together form the cRMP chromosomes. The tables also show gaps in the alignment (black lines), cRMP telomeres (white bars) and | | | | | | | | | |  |  |  |  |  |  |  |  |  |  |
| predicted centromeres (plum/black bars). Contigs, physical groups or PCR groups interrupted by inter- or intrasyntenic regions are | | | | | | | | | |  |  |  |  |  |  |  |  |  |  |
| printed in bold. A summary of the number of RMP contigs, cRMP contigs and cRMP scaffolds as well as their average, minimum, | | | | | | | | | |  |  |  |  |  |  |  |  |  |  |
| maximum and total sizes is provided. Column headings are as follows: | | | | |  |  |  |  |  |  |  |  |  |  |  |  |  |  |  |
|  |  |  |  |  |  |  |  |  |  |  |  |  |  |  |  |  |  |  |  |
| Pf chr.: P. falciparum chromosome to which the RMP contigs are aligned | | | | | |  |  |  |  |  |  |  |  |  |  |  |  |  |  |
| (#: PROmer analysis� from this study; JC# and TK#: Carlton et al.; man#: manual BLAST analysis; | | | | | | |  |  |  |  |  |  |  |  |  |  |  |  |  |
| i: inversed orientation of the synteny block; colours refer to those used in Fig. 2 of the paper); | | | | | | |  |  |  |  |  |  |  |  |  |  |  |  |  |
| Pb Pc Py contig: names of the aligned RMP contigs; | | |  |  |  |  |  |  |  |  |  |  |  |  |  |  |  |  |  |
| Size: size (bp) of the aligned RMP contigs; | |  |  |  |  |  |  |  |  |  |  |  |  |  |  |  |  |  |  |
| +/-: orientation of the aligned RMP contigs; | |  |  |  |  |  |  |  |  |  |  |  |  |  |  |  |  |  |  |
| Start contig & End contig: estimated start & end coordinates of alignment of the RMP contigs based on the coordinates | | | | | | | | |  |  |  |  |  |  |  |  |  |  |  |
| of PROmer hits corrected for the contig�s size and orientation and presence of inter- and intrasyntenic regions | | | | | | | |  |  |  |  |  |  |  |  |  |  |  |  |
| (the start position of the first RMP contig in the alignment was set at zero); | | | | | |  |  |  |  |  |  |  |  |  |  |  |  |  |  |
| cRMP contig: names of cRMP contigs | |  |  |  |  |  |  |  |  |  |  |  |  |  |  |  |  |  |  |
| (the first 2 digits refer to the cRMP chromosome number and the following 3 digits designate the order of the contigs); | | | | | | | | |  |  |  |  |  |  |  |  |  |  |  |
| cRMP contig size: size of cRMP contigs based on the corrected coordinates of alignment; | | | | | | |  |  |  |  |  |  |  |  |  |  |  |  |  |
| Gap size to next cRMP contig: size of gaps between neighbouring cRMP contigs; | | | | | | |  |  |  |  |  |  |  |  |  |  |  |  |  |
| Start cRMP contig & End cRMP contig: estimated start & end coordinates of alignment of the cRMP contigs; | | | | | | | |  |  |  |  |  |  |  |  |  |  |  |  |
| Phys. group (Py): physical linkage of neighbouring P. y. yoelii contigs; | | | | | |  |  |  |  |  |  |  |  |  |  |  |  |  |  |
| PCR group (Py): neighbouring P. y. yoelii contigs were linked previously or for this study by PCR; | | | | | | |  |  |  |  |  |  |  |  |  |  |  |  |  |
| PCR size (Py): size of the PCR product; | |  |  |  |  |  |  |  |  |  |  |  |  |  |  |  |  |  |  |
| cRMP scaffold: names of cRMP scaffolds | |  |  |  |  |  |  |  |  |  |  |  |  |  |  |  |  |  |  |
| (the first 2 digits refer to the cRMP chromosome number and the following 2 digits designate the order of the scaffolds); | | | | | | | | |  |  |  |  |  |  |  |  |  |  |  |
| cRMP scaffold size: size of cRMP scaffolds; | | |  |  |  |  |  |  |  |  |  |  |  |  |  |  |  |  |  |
| Gap size to next cRMP scaffold: size of gaps between neighbouring cRMP scaffolds; | | | | | | |  |  |  |  |  |  |  |  |  |  |  |  |  |
| Start cRMP scaffold & End cRMP scaffold: estimated start & end coordinates of alignment of the cRMP scaffolds; | | | | | | | | |  |  |  |  |  |  |  |  |  |  |  |
| Overlap: Linkage between RMP contigs established through manual BLAST analysis. | | | | | | |  |  |  |  |  |  |  |  |  |  |  |  |  |
|  |  |  |  |  |  |  |  |  |  |  |  |  |  |  |  |  |  |  |  |
| Pf chr. | Pb Pc Py contig | Size | +/- | Start contig | End contig | cRMP contig | cRMP contig size | Gap size to next cRMP contig | Start cRMP contig | End cRMP contig | Phys. group (Py) | PCR group (Py) | PCR size (Py) | cRMP scaffold | cRMP scaffold size | Gap size to next cRMP scaffold | Start cRMP scaffold | End cRMP scaffold | Overlap (manual) |
| Telomere | |  |  |  |  |  |  |  |  |  |  |  |  |  |  |  |  |  |  |
| man11 | MALPY01076 | 15313 | - | 0 | 15313 |  |  |  |  |  |  |  |  |  |  |  |  |  | \| |
| man11 | PcContig6373 | 4670 | - | 14956 | 19626 |  |  |  |  |  |  |  |  |  |  |  |  |  | MALPY02461 |
| 11 | Pb.c001903622.Contig1 | 3036 | + | 16083 | 19119 |  |  |  |  |  |  |  |  |  |  |  |  |  |  |
| 11 | MALPY02461 | 3455 | + | 16427 | 19882 |  |  |  |  |  |  |  |  |  |  |  |  |  |  |
| 11 | PbContig282 | 3572 | + | 19361 | 23813 |  |  |  |  |  |  |  |  |  |  |  |  |  |  |
| 11 | PcContig400 | 8995 | - | 19677 | 30714 |  |  |  |  |  |  |  |  |  |  |  |  |  |  |
| 11 | MALPY03131 | 1957 | - | 20206 | 23063 |  |  |  |  |  | 330B |  |  |  |  |  |  |  |  |
| 11 | MALPY00242 | 13994 | - | 22445 | 37091 |  |  |  |  |  | 330B |  |  |  |  |  |  |  |  |
| 11 | Pb.c000501114.Contig1 | 8488 | + | 24664 | 33218 |  |  |  |  |  |  |  |  |  |  |  |  |  |  |
| 11 | MALPY03443 | 1425 | - | 25118 | 26470 |  |  |  |  |  |  |  |  |  |  |  |  |  |  |
| 11 | MALPY03375 | 440 | + | 25814 | 26251 |  |  |  |  |  |  |  |  |  |  |  |  |  |  |
| 11 | Pc.c000003022.Contig1 | 1296 | - | 30777 | 32073 |  |  |  |  |  |  |  |  |  |  |  |  |  |  |
| 11 | Pc.c001505125.Contig1 | 2195 | - | 32216 | 34411 |  |  |  |  |  |  |  |  |  |  |  |  |  |  |
| 11 | PbContig105 | 10089 | + | 32969 | 43947 |  |  |  |  |  |  |  |  |  |  |  |  |  |  |
| 11 | Pc.c001707781.Contig1 | 1341 | - | 34420 | 35746 |  |  |  |  |  |  |  |  |  |  |  |  |  |  |
| 11 | MALPY00530 | 6491 | + | 37192 | 44336 |  |  |  |  |  | 330B |  |  |  |  |  |  |  |  |
| 11 | Pc.c000002648.Contig1 | 1163 | - | 37312 | 38481 |  |  |  |  |  |  |  |  |  |  |  |  |  |  |
| 11 | Pb.c000201280.Contig1 | 1525 | + | 44298 | 45796 |  |  |  |  |  |  |  |  |  |  |  |  |  |  |
| 11 | MALPY03117 | 1164 | - | 44379 | 45510 |  |  |  |  |  | 330B | 315 | 600 |  |  |  |  |  |  |
| 11 | Pb.c001702304.Contig1 | 638 | - | 45001 | 45645 |  |  |  |  |  |  |  |  |  |  |  |  |  |  |
| 11 | MALPY00380 | 4099 | - | 45560 | 50403 |  |  |  |  |  | 59 | 315 |  |  |  |  |  |  |  |
| 11 | Pb.c000901816.Contig1 | 2398 | - | 45635 | 47955 |  |  |  |  |  |  |  |  |  |  |  |  |  |  |
| 11 | Pc.c001505684.Contig1 | 906 | + | 45708 | 46602 |  |  |  |  |  |  |  |  |  |  |  |  |  |  |
| 11 | Pb.c001201610.Contig2 | 7846 | - | 48911 | 56954 |  |  |  |  |  |  |  |  |  |  |  |  |  |  |
| 11 | Pc.c001304813.Contig1 | 648 | + | 50154 | 50796 |  |  |  |  |  |  |  |  |  |  |  |  |  |  |
| 11 | MALPY00655 | 4528 | - | 50344 | 54881 |  |  |  |  |  | 59 |  |  |  |  |  |  |  |  |
| 11 | Pc.c000603041.Contig1 | 1795 | + | 51936 | 53734 |  |  |  |  |  |  |  |  |  |  |  |  |  |  |
| 11 | Pc.c001003342.Contig1 | 9099 | - | 54082 | 63262 |  |  |  |  |  |  |  |  |  |  |  |  |  |  |
| 11 | MALPY00654 | 11507 | - | 54243 | 65574 |  |  |  |  |  | 59 |  |  |  |  |  |  |  |  |
| 11 | Pb.c001402126.Contig1 | 1531 | - | 56812 | 58337 |  |  |  |  |  |  |  |  |  |  |  |  |  |  |
| 11 | Pb.c001904408.Contig1 | 2507 | - | 60254 | 62770 |  |  |  |  |  |  |  |  |  |  |  |  |  |  |
| 11 | MALPY03036 | 770 | + | 62110 | 62851 |  |  |  |  |  |  | 86 | 1500 |  |  |  |  |  |  |
| 11 | Pb.c001904944.Contig1 | 979 | - | 62782 | 63761 |  |  |  |  |  |  |  |  |  |  |  |  |  |  |
| 11 | MALPY03536 | 850 | - | 63717 | 64567 |  |  |  |  |  |  | 86 | 1500 |  |  |  |  |  |  |
| 11 | Pc.c000502450.Contig3 | 6566 | - | 65290 | 71884 |  |  |  |  |  |  |  |  |  |  |  |  |  |  |
| 11 | MALPY01093 | 11049 | - | 67162 | 79657 |  |  |  |  |  |  | 86 |  |  |  |  |  |  |  |
| 11 | Pb.c000701511.Contig1 | 2698 | + | 68573 | 71271 |  |  |  |  |  |  |  |  |  |  |  |  |  |  |
| 11 | Pb.c000701512.Contig1 | 4888 | + | 72853 | 77927 |  |  |  |  |  |  |  |  |  |  |  |  |  |  |
| 11 | PcContig257 | 7685 | - | 74349 | 82898 |  |  |  |  |  |  |  |  |  |  |  |  |  |  |
| 11 | Pb.c000701439.Contig1 | 4700 | + | 78509 | 83233 |  |  |  |  |  |  |  |  |  |  |  |  |  |  |
| 11 | MALPY00656 | 7568 | - | 80410 | 88127 |  |  |  |  |  |  |  |  |  |  |  |  |  |  |
| 11 | Pc.c001505609.Contig1 | 819 | - | 82985 | 83807 | cRMPc09001 | 88127 | 3426 | 0 | 88127 |  |  |  | cRMPs0901 | 88127 | 3426 | 0 | 88127 |  |
| 11 | MALPY00788 | 2989 | + | 91553 | 95310 |  |  |  |  |  |  | 124 | 3300 |  |  |  |  |  |  |
| 11 | Pc.c001103678.Contig1 | 4835 | - | 92802 | 97655 | cRMPc09002 | 6102 | 1739 | 91553 | 97655 |  |  |  |  |  |  |  |  |  |
| 11 | MALPY03125 | 712 | + | 99394 | 100100 |  |  |  |  |  |  | 124 |  |  |  |  |  |  |  |
| man11 | PcContig1235 | 1547 | - | 99428 | 101545 |  |  |  |  |  |  |  |  |  |  |  |  |  | MALPY03119/MALPY02709 |
| 11 | MALPY03119 | 577 | + | 99617 | 100194 |  |  |  |  |  |  | 72 | 3100 |  |  |  |  |  |  |
| man11 | MALPY02709 | 3012 | + | 100787 | 103799 |  |  |  |  |  | 30 |  |  |  |  |  |  |  | PcContig1235/PbContig37 |
| 11 | Pc.c000002072.Contig3 | 3488 | - | 103020 | 106508 |  |  |  |  |  |  |  |  |  |  |  |  |  |  |
| 11 | MALPY03023 | 1731 | - | 103383 | 105114 |  |  |  |  |  | 30 | 72 |  |  |  |  |  |  |  |
| 11 | PbContig37 | 4099 | + | 103601 | 107700 | cRMPc09003 | 8306 | 407 | 99394 | 107700 |  |  |  | cRMPs0902 | 16147 | 407 | 91553 | 107700 |  |
| 11 | Pb.c001702707.Contig1 | 7644 | + | 108107 | 115751 |  |  |  |  |  |  |  |  |  |  |  |  |  |  |
| 11 | MALPY01115 | 12557 | - | 111677 | 126950 |  |  |  |  |  | 101 |  |  |  |  |  |  |  |  |
| 11 | Pc.c001304182.Contig1 | 3316 | - | 112087 | 115403 |  |  |  |  |  |  |  |  |  |  |  |  |  |  |
| 11 | Pb.c000801705.Contig1 | 4001 | + | 116782 | 120783 |  |  |  |  |  |  |  |  |  |  |  |  |  |  |
| 11 | Pc.c000803385.Contig1 | 1884 | - | 116788 | 118672 |  |  |  |  |  |  |  |  |  |  |  |  |  |  |
| 11 | Pc.c000302925.Contig1 | 2232 | + | 124138 | 126370 |  |  |  |  |  |  |  |  |  |  |  |  |  |  |
| 11 | PbContig161 | 3457 | + | 124734 | 128192 |  |  |  |  |  |  |  |  |  |  |  |  |  |  |
| 11 | MALPY03401 | 221 | + | 125832 | 126067 |  |  |  |  |  |  | 53 | 300 |  |  |  |  |  |  |
| 11 | MALPY00971 | 17011 | - | 126010 | 143486 |  |  |  |  |  | 101 | 53 | 600 |  |  |  |  |  |  |
| 11 | Pc.c001707994.Contig1 | 2656 | - | 127124 | 129904 |  |  |  |  |  |  |  |  |  |  |  |  |  |  |
| 11 | Pb.c001402154.Contig1 | 2678 | + | 128356 | 131113 |  |  |  |  |  |  |  |  |  |  |  |  |  |  |
| 11 | Pc.c001707823.Contig1 | 1194 | - | 130042 | 131236 |  |  |  |  |  |  |  |  |  |  |  |  |  |  |
| 11 | Pb.c001001701.Contig1 | 3431 | + | 132635 | 136059 |  |  |  |  |  |  |  |  |  |  |  |  |  |  |
| 11 | Pc.c001708184.Contig1 | 566 | + | 133615 | 134181 |  |  |  |  |  |  |  |  |  |  |  |  |  |  |
| 11 | PcContig9 | 5366 | + | 135243 | 140667 |  |  |  |  |  |  |  |  |  |  |  |  |  |  |
| 11 | Pb.c001702837.Contig1 | 4735 | - | 139502 | 144553 |  |  |  |  |  |  |  |  |  |  |  |  |  |  |
| 11 | PcContig529 | 1225 | + | 140840 | 142096 |  |  |  |  |  |  |  |  |  |  |  |  |  |  |
| 11 | Pc.c000603530.Contig1 | 916 | + | 142185 | 143374 |  |  |  |  |  |  |  |  |  |  |  |  |  |  |
| 11 | MALPY03051 | 846 | + | 143344 | 144190 | cRMPc09004 | 36446 | 105 | 108107 | 144553 |  | 53 | 900 |  |  |  |  |  |  |
| 11 | MALPY01885 | 4480 | + | 144658 | 149265 |  |  |  |  |  |  | 53 | 900 |  |  |  |  |  |  |
| 11 | Pb.c001602413.Contig1 | 3103 | + | 146980 | 150083 |  |  |  |  |  |  |  |  |  |  |  |  |  |  |
| 11 | Pc.c001405401.Contig1 | 1046 | - | 148020 | 149414 | cRMPc09005 | 5425 | 670 | 144658 | 150083 |  |  |  |  |  |  |  |  |  |
| 11 | MALPY02211 | 4529 | - | 150753 | 155300 |  |  |  |  |  | 274 | 53 |  |  |  |  |  |  |  |
| 11 | Pc.c000302877.Contig1 | 3328 | - | 154114 | 157855 |  |  |  |  |  |  |  |  |  |  |  |  |  |  |
| 11 | Pb.c001101862.Contig1 | 4198 | + | 154762 | 159196 |  |  |  |  |  |  |  |  |  |  |  |  |  |  |
| 11 | MALPY02178 | 3780 | - | 155419 | 159492 |  |  |  |  |  | 274 |  |  |  |  |  |  |  |  |
| 11 | Pc.c000803715.Contig1 | 624 | + | 157419 | 158043 |  |  |  |  |  |  |  |  |  |  |  |  |  |  |
| 11 | PcContig44 | 6470 | + | 157675 | 164769 |  |  |  |  |  |  |  |  |  |  |  |  |  |  |
| 11 | Pb.c001101863.Contig1 | 5241 | + | 159136 | 164826 |  |  |  |  |  |  |  |  |  |  |  |  |  |  |
| 11 | MALPY00779 | 5487 | + | 159427 | 165008 | cRMPc09006 | 14255 | 198 | 150753 | 165008 | 274 |  |  |  |  |  |  |  |  |
| 11 | MALPY00780 | 2095 | + | 165206 | 167310 | cRMPc09007 | 2104 | 1372 | 165206 | 167310 | 274 |  |  | cRMPs0903 | 59203 | 1372 | 108107 | 167310 |  |
| 11 | MALPY02869 | 2371 | - | 168682 | 171047 |  |  |  |  |  |  | 281 | 800 |  |  |  |  |  |  |
| 11 | Pb.c001402211.Contig1 | 2053 | - | 169255 | 171308 |  |  |  |  |  |  |  |  |  |  |  |  |  |  |
| 11 | MALPY01063 | 12825 | - | 171270 | 183464 |  |  |  |  |  | 482A | 281 |  |  |  |  |  |  |  |
| 11 | Pb.c000401204.Contig1 | 8730 | - | 171278 | 179709 |  |  |  |  |  |  |  |  |  |  |  |  |  |  |
| 11 | PcContig162 | 5348 | - | 178351 | 183693 |  |  |  |  |  |  |  |  |  |  |  |  |  |  |
| 11 | Pb.c001001784.Contig1 | 2144 | - | 179653 | 181815 | cRMPc09008 | 15011 | 429 | 168682 | 183693 |  |  |  |  |  |  |  |  |  |
| 11 | MALPY01535 | 6192 | - | 184122 | 190377 |  |  |  |  |  | 482A | 278 | 2800 |  |  |  |  |  |  |
| 11 | Pc.c000302992.Contig1 | 4180 | - | 185231 | 189375 |  |  |  |  |  |  |  |  |  |  |  |  |  |  |
| 11 | Pb.c001202127.Contig1 | 4692 | + | 185828 | 190547 | cRMPc09009 | 6425 | 3550 | 184122 | 190547 |  |  |  |  |  |  |  |  |  |
| 11 | MALPY01118 | 6189 | + | 194097 | 200535 |  |  |  |  |  |  | 278 |  |  |  |  |  |  |  |
| 11 | PbContig41 | 5618 | - | 195066 | 200666 |  |  |  |  |  |  |  |  |  |  |  |  |  |  |
| 11 | Pc.c001103722.Contig1 | 1629 | - | 195534 | 197166 | cRMPc09010 | 6569 | 1836 | 194097 | 200666 |  |  |  | cRMPs0904 | 31984 | 1836 | 168682 | 200666 |  |
| 11 | MALPY02808 | 4113 | + | 202502 | 206656 | cRMPc09011 | 4154 | 221 | 202502 | 206656 |  | 259 | 1000 |  |  |  |  |  |  |
| 11 | MALPY02505 | 6155 | + | 206877 | 213489 |  |  |  |  |  |  | 259 | 6500 |  |  |  |  |  |  |
| 11 | Pb.c001201667.Contig1 | 4614 | + | 206927 | 212030 | cRMPc09012 | 6612 | 7283 | 206877 | 213489 |  |  |  |  |  |  |  |  |  |
| 11 | MALPY01924 | 7439 | - | 220772 | 228427 |  |  |  |  |  | 501 | 259 |  |  |  |  |  |  |  |
| 11 | Pc.c000703238.Contig1 | 2039 | - | 221396 | 223435 |  |  |  |  |  |  |  |  |  |  |  |  |  |  |
| 11 | Pb.c000401198.Contig1 | 5675 | - | 222176 | 228139 |  |  |  |  |  |  |  |  |  |  |  |  |  |  |
| 11 | Pc.c000703776.Contig1 | 610 | - | 223415 | 224049 |  |  |  |  |  |  |  |  |  |  |  |  |  |  |
| 11 | Pc.c000302336.Contig1 | 5238 | - | 224450 | 229694 |  |  |  |  |  |  |  |  |  |  |  |  |  |  |
| 11 | Pb.c002005838.Contig1 | 3117 | - | 229038 | 232173 |  |  |  |  |  |  |  |  |  |  |  |  |  |  |
| 11 | MALPY00967 | 4242 | - | 229129 | 233377 |  |  |  |  |  | 501 |  |  |  |  |  |  |  |  |
| 11 | PcContig635 | 4049 | + | 231016 | 235065 |  |  |  |  |  |  |  |  |  |  |  |  |  |  |
| 11 | MALPY00246 | 5342 | - | 234850 | 240479 |  |  |  |  |  | 501 | 254 | 1500 |  |  |  |  |  |  |
| 11 | Pb.c000301152.Contig1 | 4341 | + | 235980 | 240715 |  |  |  |  |  |  |  |  |  |  |  |  |  |  |
| 11 | Pc.c001505392.Contig1 | 4211 | + | 236854 | 241462 | cRMPc09013 | 20690 | 145 | 220772 | 241462 |  |  |  |  |  |  |  |  |  |
| 11 | Pb.c000201519.Contig1 | 12008 | - | 241607 | 253491 |  |  |  |  |  |  |  |  |  |  |  |  |  |  |
| 11 | MALPY01585 | 6679 | + | 242432 | 248652 |  |  |  |  |  | 96 | 254 |  |  |  |  |  |  |  |
| 11 | Pc.c001203838.Contig1 | 1606 | + | 244197 | 245985 |  |  |  |  |  |  |  |  |  |  |  |  |  |  |
| 11 | MALPY03300 | 740 | + | 245648 | 246430 |  |  |  |  |  |  |  |  |  |  |  |  |  |  |
| 11 | MALPY03314 | 1075 | - | 245780 | 246876 |  |  |  |  |  |  |  |  |  |  |  |  |  |  |
| 11 | Pc.c001103826.Contig1 | 1432 | + | 246224 | 247664 |  |  |  |  |  |  |  |  |  |  |  |  |  |  |
| 11 | Pc.c001811772.Contig1 | 804 | + | 247603 | 248407 |  |  |  |  |  |  |  |  |  |  |  |  |  |  |
| 11 | MALPY02576 | 5420 | - | 248726 | 254107 | cRMPc09014 | 12500 | 623 | 241607 | 254107 | 96 |  |  |  |  |  |  |  |  |
| 11 | Pb.c002006978.Contig1 | 3215 | - | 254730 | 258053 |  |  |  |  |  |  |  |  |  |  |  |  |  |  |
| 11 | MALPY00765 | 3683 | - | 255152 | 258862 |  |  |  |  |  | 96 | 99 | 1400 |  |  |  |  |  |  |
| 11 | Pc.c000302922.Contig1 | 2815 | - | 256134 | 259046 | cRMPc09015 | 4316 | 357 | 254730 | 259046 |  |  |  |  |  |  |  |  |  |
| 11 | PcContig46 | 3415 | - | 259403 | 262827 |  |  |  |  |  |  |  |  |  |  |  |  |  |  |
| 11 | MALPY02308 | 4365 | + | 260113 | 265431 |  |  |  |  |  | 547 | 99 |  |  |  |  |  |  |  |
| 11 | Pc.c001103736.Contig1.1 | 639 | - | 260378 | 261023 |  |  |  |  |  |  |  |  |  |  |  |  |  |  |
| 11 | PcContig339 | 3341 | + | 263918 | 267274 |  |  |  |  |  |  |  |  |  |  |  |  |  |  |
| 11 | Pb.c001001991.Contig1 | 1046 | + | 264525 | 265571 |  |  |  |  |  |  |  |  |  |  |  |  |  |  |
| JC11 | MALPY00191 | 6526 | + | 264828 | 271369 | cRMPc09016 | 11966 | 2577 | 259403 | 271369 | 547 |  |  | cRMPs0905 | 68867 | 2577 | 202502 | 271369 |  |
| 11 | MALPY02362 | 2550 | + | 273946 | 276496 |  |  |  |  |  |  |  |  |  |  |  |  |  |  |
| 11 | Pb.c000101409.Contig1 | 2136 | + | 274661 | 276797 | cRMPc09017 | 2851 | 591 | 273946 | 276797 |  |  |  | cRMPs0906 | 2851 | 591 | 273946 | 276797 |  |
| 11 | PbContig12 | 2414 | + | 277388 | 279790 |  |  |  |  |  |  |  |  |  |  |  |  |  |  |
| 11 | Pc.c000803347.Contig1 | 1960 | - | 278031 | 279997 |  |  |  |  |  |  |  |  |  |  |  |  |  |  |
| 11 | MALPY01090 | 9974 | - | 278043 | 289293 |  |  |  |  |  |  |  |  |  |  |  |  |  |  |
| 11 | Pb.c000401216.Contig1 | 7834 | - | 281872 | 289842 |  |  |  |  |  |  |  |  |  |  |  |  |  |  |
| 11 | Pc.c000803832.Contig1 | 1355 | - | 282630 | 284170 |  |  |  |  |  |  |  |  |  |  |  |  |  |  |
| 11 | Pc.c000002716.Contig1 | 4855 | - | 284446 | 289578 | cRMPc09018 | 12454 | 1088 | 277388 | 289842 |  |  |  | cRMPs0907 | 12454 | 1088 | 277388 | 289842 |  |
| 11 | MALPY00471 | 24558 | - | 290930 | 316810 |  |  |  |  |  | 332 |  |  |  |  |  |  |  |  |
| 11 | PbContig286 | 8691 | - | 292054 | 301891 |  |  |  |  |  |  |  |  |  |  |  |  |  |  |
| 11 | Pc.c000703417.Contig1 | 3324 | - | 295961 | 299504 |  |  |  |  |  |  |  |  |  |  |  |  |  |  |
| 11 | MALPY03342 | 1028 | - | 299231 | 300367 |  |  |  |  |  |  | 267 | 6500 |  |  |  |  |  |  |
| 11 | PcContig432 | 4406 | - | 299924 | 304513 |  |  |  |  |  |  |  |  |  |  |  |  |  |  |
| 11 | PbContig142 | 8541 | - | 302705 | 311095 |  |  |  |  |  |  |  |  |  |  |  |  |  |  |
| 11 | Pc.c000703819.Contig1 | 1093 | - | 305719 | 306812 |  |  |  |  |  |  |  |  |  |  |  |  |  |  |
| 11 | MALPY03489 | 253 | + | 306742 | 306995 |  |  |  |  |  |  | 267 |  |  |  |  |  |  |  |
| 11 | Pc.c000703818.Contig1 | 756 | - | 306879 | 307638 |  |  |  |  |  |  |  |  |  |  |  |  |  |  |
| 11 | Pc.c001606304.Contig1 | 1195 | - | 309369 | 310558 |  |  |  |  |  |  |  |  |  |  |  |  |  |  |
| 11 | PcContig83 | 6520 | + | 311138 | 317905 |  |  |  |  |  |  |  |  |  |  |  |  |  |  |
| 11 | PbContig100 | 7179 | - | 311446 | 318821 |  |  |  |  |  |  |  |  |  |  |  |  |  |  |
| 11 | Pc.c001708772.Contig1 | 673 | - | 314046 | 314719 |  |  |  |  |  |  |  |  |  |  |  |  |  |  |
| 11 | MALPY00747 | 4523 | - | 317281 | 321795 |  |  |  |  |  | 332 | 69 | 4000 |  |  |  |  |  |  |
| 11 | Pb.c001702823.Contig1 | 1033 | - | 319864 | 320909 | cRMPc09019 | 30865 | 2413 | 290930 | 321795 |  |  |  |  |  |  |  |  |  |
| 11 | Pb.c000501106.Contig1 | 12895 | + | 324208 | 337905 |  |  |  |  |  |  |  |  |  |  |  |  |  |  |
| 11 | MALPY01097 | 7107 | + | 325447 | 332803 |  |  |  |  |  | 197 | 69 |  |  |  |  |  |  |  |
| 11 | PcContig528 | 2017 | - | 325959 | 328075 |  |  |  |  |  |  |  |  |  |  |  |  |  |  |
| 11 | MALPY03096 | 370 | + | 327952 | 328322 |  |  |  |  |  |  |  |  |  |  |  |  |  |  |
| 11 | Pc.c001303489.Contig3 | 2709 | - | 332037 | 334714 |  |  |  |  |  |  |  |  |  |  |  |  |  |  |
| 11 | MALPY03169 | 1044 | - | 333275 | 334271 |  |  |  |  |  | 197 | 229 | 1300 |  |  |  |  |  |  |
| 11 | Pc.c001304215.Contig1 | 1901 | - | 335217 | 337127 | cRMPc09020 | 13697 | 944 | 324208 | 337905 |  |  |  |  |  |  |  |  |  |
| man11 | Pb.c000201297.Contig1 | 4988 | + | 338849 | 349408 |  |  |  |  |  |  |  |  |  |  |  |  |  |  |
| JC11 | MALPY03285 | 753 | - | 339767 | 340520 |  |  |  |  |  |  | 229 | 350 |  |  |  |  |  |  |
| 11 | MALPY01580 | 5037 | - | 340997 | 346070 |  |  |  |  |  |  | 229 |  |  |  |  |  |  |  |
| 11 | Pb.c001803898.Contig1 | 1274 | + | 344440 | 345708 |  |  |  |  |  |  |  |  |  |  |  |  |  |  |
| 11 | MALPY01878 | 4378 | + | 345840 | 350218 |  |  |  |  |  |  |  |  |  |  |  |  |  |  |
| 11 | Pc.c001002723.Contig1 | 6158 | + | 346050 | 352304 | cRMPc09021 | 13455 | 0 | 338849 | 352304 |  |  |  | cRMPs0908 | 61374 | 0 | 290930 | 352304 |  |
| 11 | MALPY01413 | 2497 | + | 352304 | 354753 |  |  |  |  |  |  |  |  |  |  |  |  |  |  |
| 11 | MALPY03033 | 745 | + | 354537 | 355285 |  |  |  |  |  |  |  |  |  |  |  |  |  |  |
| 11 | Pc.c000903287.Contig1 | 2709 | - | 355068 | 358050 |  |  |  |  |  |  |  |  |  |  |  |  |  |  |
| 11 | MALPY01751 | 15962 | + | 355131 | 370812 |  |  |  |  |  | 426 |  |  |  |  |  |  |  |  |
| 11 | Pb.c002112907.Contig1 | 636 | + | 355985 | 356621 |  |  |  |  |  |  |  |  |  |  |  |  |  |  |
| 11 | Pb.c001803374.Contig1 | 2076 | + | 356695 | 359059 |  |  |  |  |  |  |  |  |  |  |  |  |  |  |
| 11 | MALPY03167 | 195 | - | 357525 | 357720 |  |  |  |  |  |  |  |  |  |  |  |  |  |  |
| 11 | Pb.c001301846.Contig1 | 2672 | - | 363543 | 366227 |  |  |  |  |  |  |  |  |  |  |  |  |  |  |
| 11 | MALPY01825 | 6364 | + | 370667 | 377031 |  |  |  |  |  | 426 |  |  |  |  |  |  |  |  |
| 11 | Pb.c002112096.Contig1 | 2034 | - | 371999 | 374033 | cRMPc09022 | 24727 | 1505 | 352304 | 377031 |  |  |  | cRMPs0909 | 24727 | 1505 | 352304 | 377031 |  |
| 11 | MALPY02352 | 6386 | - | 378536 | 385051 |  |  |  |  |  | 299 |  |  |  |  |  |  |  |  |
| 11 | Pb.c001201983.Contig1 | 848 | + | 381570 | 382418 |  |  |  |  |  |  |  |  |  |  |  |  |  |  |
| 11 | Pb.c000001343.Contig2 | 7187 | + | 382410 | 390840 |  |  |  |  |  |  |  |  |  |  |  |  |  |  |
| 11 | Pc.c000803721.Contig1 | 5079 | - | 386264 | 391343 |  |  |  |  |  |  |  |  |  |  |  |  |  |  |
| 11 | MALPY01787 | 6025 | + | 386297 | 392322 | cRMPc09023 | 13786 | 1503 | 378536 | 392322 | 299 |  |  |  |  |  |  |  |  |
| 11 | MALPY01215 | 7064 | + | 393825 | 401083 |  |  |  |  |  | 299 |  |  |  |  |  |  |  |  |
| 11 | Pb.c000301400.Contig1 | 2872 | - | 394757 | 397602 |  |  |  |  |  |  |  |  |  |  |  |  |  |  |
| 11 | PcContig101 | 2529 | - | 395747 | 398273 |  |  |  |  |  |  |  |  |  |  |  |  |  |  |
| 11 | Pb.c000301346.Contig1 | 8508 | - | 397552 | 406186 |  |  |  |  |  |  |  |  |  |  |  |  |  |  |
| 11 | PcContig8 | 3754 | - | 398764 | 402532 |  |  |  |  |  |  |  |  |  |  |  |  |  |  |
| 11 | MALPY00196 | 30629 | - | 400997 | 431606 |  |  |  |  |  | 299 |  |  |  |  |  |  |  |  |
| 11 | Pc.c001405169.Contig1 | 1275 | - | 402402 | 403653 |  |  |  |  |  |  |  |  |  |  |  |  |  |  |
| 11 | Pc.c001303500.Contig2 | 4687 | - | 404013 | 408659 |  |  |  |  |  |  |  |  |  |  |  |  |  |  |
| 11 | Pb.c001702797.Contig1 | 3275 | - | 406400 | 409344 |  |  |  |  |  |  |  |  |  |  |  |  |  |  |
| 11 | Pb.c000201551.Contig1 | 4919 | - | 409464 | 414386 |  |  |  |  |  |  |  |  |  |  |  |  |  |  |
| 11 | PcContig733 | 3134 | - | 411372 | 414497 |  |  |  |  |  |  |  |  |  |  |  |  |  |  |
| 11 | Pb.c000101063.Contig1 | 8283 | + | 413564 | 422509 |  |  |  |  |  |  |  |  |  |  |  |  |  |  |
| 11 | Pc.c000402713.Contig1 | 3324 | - | 415831 | 419667 |  |  |  |  |  |  |  |  |  |  |  |  |  |  |
| 11 | Pc.c000602326.Contig2 | 4954 | + | 424334 | 429288 |  |  |  |  |  |  |  |  |  |  |  |  |  |  |
| 11 | MALPY00826 | 6003 | - | 431474 | 437583 |  |  |  |  |  | 204 |  |  |  |  |  |  |  |  |
| 11 | Pb.c001401702.Contig1 | 6786 | + | 432241 | 438944 |  |  |  |  |  |  |  |  |  |  |  |  |  |  |
| 11 | Pc.c001605884.Contig1 | 564 | - | 433495 | 434059 |  |  |  |  |  |  |  |  |  |  |  |  |  |  |
| 11 | Pc.c001204447.Contig1 | 658 | + | 433800 | 434413 |  |  |  |  |  |  |  |  |  |  |  |  |  |  |
| 11 | Pc.c001204120.Contig1 | 3914 | - | 435131 | 439098 |  |  |  |  |  |  |  |  |  |  |  |  |  |  |
| man11 | MALPY00211 - Exp | 27430 | - | 437094 | 465547 |  |  |  |  |  | 204 | 47 - Exp | 500 |  |  |  |  |  |  |
| man11 | PbContig10 - Exp | 25041 | + | 438669 | 465601 |  |  |  |  |  |  |  |  |  |  |  |  |  |  |
| 11 | Pc.c001405274.Contig1 | 1013 | + | 445668 | 446777 |  |  |  |  |  |  |  |  |  |  |  |  |  |  |
| 11 | Pc.c001404747.Contig1 | 1798 | + | 448186 | 449938 |  |  |  |  |  |  |  |  |  |  |  |  |  |  |
| 11 | Pc.c001003700.Contig1 | 1280 | - | 455419 | 456772 |  |  |  |  |  |  |  |  |  |  |  |  |  |  |
| 11 | Pc.c001304222.Contig1 | 3970 | - | 462268 | 466098 |  |  |  |  |  |  |  |  |  |  |  |  |  |  |
| 11 | MALPY00592 | 28459 | - | 466047 | 498518 |  |  |  |  |  |  | 47 - Exp |  |  |  |  |  |  |  |
| 11 | Pb.c001802851.Contig1 | 2415 | - | 467092 | 469507 |  |  |  |  |  |  |  |  |  |  |  |  |  |  |
| 11 | Pc.c001304159.Contig1 | 1873 | - | 467457 | 469399 |  |  |  |  |  |  |  |  |  |  |  |  |  |  |
| 11 | PbContig124 | 2871 | + | 469638 | 472533 |  |  |  |  |  |  |  |  |  |  |  |  |  |  |
| 11 | Pc.c000403319.Contig1 | 1523 | - | 470670 | 472163 |  |  |  |  |  |  |  |  |  |  |  |  |  |  |
| 11 | Pb.c000601553.Contig1 | 3885 | + | 473169 | 477801 |  |  |  |  |  |  |  |  |  |  |  |  |  |  |
| 11 | PcContig5 | 1983 | - | 473280 | 475263 |  |  |  |  |  |  |  |  |  |  |  |  |  |  |
| 11 | Pc.c001203885.Contig1 | 2790 | - | 477288 | 480060 |  |  |  |  |  |  |  |  |  |  |  |  |  |  |
| 11 | Pb.c002006861.Contig1 | 593 | + | 478153 | 478770 |  |  |  |  |  |  |  |  |  |  |  |  |  |  |
| 11 | Pb.c002006862.Contig1 | 1651 | + | 479332 | 481070 |  |  |  |  |  |  |  |  |  |  |  |  |  |  |
| 11 | Pb.c001402106.Contig1 | 4420 | + | 482668 | 487124 |  |  |  |  |  |  |  |  |  |  |  |  |  |  |
| 11 | Pb.c000501120.Contig1 | 6084 | - | 487198 | 494323 |  |  |  |  |  |  |  |  |  |  |  |  |  |  |
| 11 | Pc.c001505777.Contig1 | 421 | + | 491606 | 492027 |  |  |  |  |  |  |  |  |  |  |  |  |  |  |
| 11 | Pc.c001505709.Contig1 | 822 | + | 494095 | 495136 |  |  |  |  |  |  |  |  |  |  |  |  |  |  |
| 11 | Pb.c000601221.Contig2 | 6420 | - | 494360 | 501251 |  |  |  |  |  |  |  |  |  |  |  |  |  |  |
| 11 | Pc.c001204464.Contig1 | 616 | + | 495179 | 495795 |  |  |  |  |  |  |  |  |  |  |  |  |  |  |
| 11 | Pc.c000803777.Contig1 | 739 | - | 495932 | 496668 |  |  |  |  |  |  |  |  |  |  |  |  |  |  |
| 11 | MALPY01700 | 9493 | - | 497151 | 507126 |  |  |  |  |  |  |  |  |  |  |  |  |  |  |
| 11 | Pc.c000603502.Contig1 | 1346 | + | 497422 | 498780 |  |  |  |  |  |  |  |  |  |  |  |  |  |  |
| 11 | PcContig627 | 3781 | + | 498527 | 502317 |  |  |  |  |  |  |  |  |  |  |  |  |  |  |
| 11 | Pc.c001003439.Contig1 | 2436 | - | 503183 | 505616 |  |  |  |  |  |  |  |  |  |  |  |  |  |  |
| 11 | PbContig86 | 3898 | + | 503246 | 507144 | cRMPc09024 | 113319 | 3227 | 393825 | 507144 |  |  |  | cRMPs0910 | 128608 | 3227 | 378536 | 507144 |  |
| 11 | MALPY01152 | 5108 | + | 510371 | 515422 |  |  |  |  |  |  |  |  |  |  |  |  |  |  |
| 11 | Pc.c001812704.Contig1 | 734 | - | 514549 | 515292 | cRMPc09025 | 5051 | 1322 | 510371 | 515422 |  |  |  | cRMPs0911 | 5051 | 1322 | 510371 | 515422 |  |
| 11 | MALPY01338 | 7525 | - | 516744 | 524009 |  |  |  |  |  | 711 |  |  |  |  |  |  |  |  |
| 11 | Pc.c001003303.Contig1 | 3258 | - | 517478 | 520172 |  |  |  |  |  |  |  |  |  |  |  |  |  |  |
| 11 | Pb.c002006801.Contig1 | 1505 | - | 519258 | 520784 |  |  |  |  |  |  |  |  |  |  |  |  |  |  |
| 11 | PcContig353 | 1951 | - | 520053 | 521995 | cRMPc09026 | 7265 | 967 | 516744 | 524009 |  |  |  |  |  |  |  |  |  |
| 11 | MALPY02113 | 8813 | + | 524976 | 534202 |  |  |  |  |  | 711 |  |  |  |  |  |  |  |  |
| 11 | Pb.c000301343.Contig1 | 8067 | + | 530233 | 539460 |  |  |  |  |  |  |  |  |  |  |  |  |  |  |
| 11 | Pc.c000303524.Contig1 | 1571 | + | 531841 | 534014 |  |  |  |  |  |  |  |  |  |  |  |  |  |  |
| 11 | PcContig147 | 7362 | + | 534236 | 542419 |  |  |  |  |  |  |  |  |  |  |  |  |  |  |
| 11 | MALPY01280 | 4644 | - | 534371 | 539458 |  |  |  |  |  | 711 | 65 | 1600 |  |  |  |  |  |  |
| man11 | MALPY01648 - Ins | 14663 | + | 539748 | 555712 |  |  |  |  |  | 157B | 65 | 1200 |  |  |  |  |  |  |
| 11 | Pb.c002112134.Contig1 | 1442 | + | 540673 | 542082 |  |  |  |  |  |  |  |  |  |  |  |  |  |  |
| 11 | Pc.c001304789.Contig1 | 620 | - | 542040 | 542828 |  |  |  |  |  |  |  |  |  |  |  |  |  |  |
| 11 | Pc.c001505838.Contig1 | 595 | + | 542086 | 542849 |  |  |  |  |  |  |  |  |  |  |  |  |  |  |
| 11 | Pc.c000303404.Contig1 | 696 | + | 542144 | 543008 |  |  |  |  |  |  |  |  |  |  |  |  |  |  |
| 11 | Pb.c000801700.Contig1 | 3740 | - | 542361 | 546488 |  |  |  |  |  |  |  |  |  |  |  |  |  |  |
| 11 | MALPY03330 | 842 | + | 542515 | 543333 |  |  |  |  |  |  | 65 | 1400 |  |  |  |  |  |  |
| 11 | Pc.c000703343.Contig1 | 5026 | + | 543074 | 548355 |  |  |  |  |  |  |  |  |  |  |  |  |  |  |
| 11 | Pb.c001702343.Contig2 | 5925 | + | 549539 | 556180 |  |  |  |  |  |  |  |  |  |  |  |  |  |  |
| 11 | Pc.c001304923.Contig1 | 1443 | + | 549959 | 551402 |  |  |  |  |  |  |  |  |  |  |  |  |  |  |
| 11 | PcContig47 | 3936 | + | 551484 | 555956 | cRMPc09027 | 31204 | 782 | 524976 | 556180 |  |  |  |  |  |  |  |  |  |
| 11 | MALPY01274 | 7299 | - | 556962 | 565183 |  |  |  |  |  | 157B | 65 | 4300 |  |  |  |  |  |  |
| 11 | Pc.c000402952.Contig1 | 4375 | - | 558126 | 563175 |  |  |  |  |  |  |  |  |  |  |  |  |  |  |
| 11 | Pb.c001202318.Contig1 | 1082 | + | 558761 | 559900 |  |  |  |  |  |  |  |  |  |  |  |  |  |  |
| 11 | Pb.c001102214.Contig1 | 1137 | + | 560161 | 561394 |  |  |  |  |  |  |  |  |  |  |  |  |  |  |
| 11 | Pb.c000801735.Contig1 | 2603 | + | 561903 | 564739 |  |  |  |  |  |  |  |  |  |  |  |  |  |  |
| 11 | Pc.c001204528.Contig1 | 1074 | - | 563657 | 564731 |  |  |  |  |  |  |  |  |  |  |  |  |  |  |
| man11 | MALPY01327 | 2262 | - | 565017 | 567279 |  |  |  |  |  | 345 |  |  |  |  |  |  |  | Pb.c000301154.Contig2 |
| 11 | Pb.c000301154.Contig2 | 10441 | + | 565232 | 575416 |  |  |  |  |  |  |  |  |  |  |  |  |  |  |
| 11 | Pc.c001505815.Contig1 | 1010 | - | 566939 | 567949 |  |  |  |  |  |  |  |  |  |  |  |  |  |  |
| 11 | MALPY00243 | 10748 | + | 567397 | 577550 |  |  |  |  |  | 345 | 65 | 1700 |  |  |  |  |  |  |
| 11 | Pc.c000903728.Contig1 | 1631 | - | 568259 | 569905 |  |  |  |  |  |  |  |  |  |  |  |  |  |  |
| 11 | PcContig757 | 4826 | - | 569543 | 574387 |  |  |  |  |  |  |  |  |  |  |  |  |  |  |
| 11 | Pb.c000401503.Contig1 | 2446 | + | 577526 | 580156 |  |  |  |  |  |  |  |  |  |  |  |  |  |  |
| 11 | PcContig306 | 2922 | - | 578137 | 581298 |  |  |  |  |  |  |  |  |  |  |  |  |  |  |
| 11 | MALPY01437 | 13630 | + | 578699 | 592753 |  |  |  |  |  |  | 65 |  |  |  |  |  |  |  |
| 11 | PcContig649 | 6188 | - | 583989 | 590610 |  |  |  |  |  |  |  |  |  |  |  |  |  |  |
| 11 | Pb.c001001757.Contig1 | 5040 | - | 585843 | 590874 |  |  |  |  |  |  |  |  |  |  |  |  |  |  |
| 11 | MALPY03065 | 267 | + | 589621 | 589885 | cRMPc09028 | 35791 | 1899 | 556962 | 592753 |  | 137 | 1800 |  |  |  |  |  |  |
| 11 | MALPY01916 | 2273 | + | 594652 | 596961 |  |  |  |  |  | 388 | 137 | 1800 |  |  |  |  |  |  |
| 11 | Pc.c001304263.Contig1 | 1772 | - | 596482 | 598467 |  |  |  |  |  |  |  |  |  |  |  |  |  |  |
| 11 | MALPY01915 | 8344 | - | 597375 | 606399 |  |  |  |  |  | 388 | 137 - NS | 4000 |  |  |  |  |  |  |
| 11 | Pb.c000801685.Contig1 | 6220 | - | 597938 | 605249 |  |  |  |  |  |  |  |  |  |  |  |  |  |  |
| 11 | PcContig447 | 3783 | - | 599619 | 603566 |  |  |  |  |  |  |  |  |  |  |  |  |  |  |
| 11 | Pc.c000403264.Contig1 | 2334 | - | 603716 | 606008 | cRMPc09029 | 11747 | 1679 | 594652 | 606399 |  |  |  |  |  |  |  |  |  |
| 11 | PcContig507 | 6809 | + | 608078 | 615169 |  |  |  |  |  |  |  |  |  |  |  |  |  |  |
| TK11 | MALPY01385 | 2409 | + | 608870 | 610867 |  |  |  |  |  | 18 |  |  |  |  |  |  |  |  |
| 11 | MALPY00857 | 9655 | - | 610399 | 619967 |  |  |  |  |  | 18 | 137 - NS |  |  |  |  |  |  |  |
| 11 | Pb.c000601543.Contig1 | 3859 | + | 611083 | 614930 |  |  |  |  |  |  |  |  |  |  |  |  |  |  |
| 11 | Pb.c000901737.Contig1 | 7852 | - | 618424 | 626662 |  |  |  |  |  |  |  |  |  |  |  |  |  |  |
| 11 | MALPY03008 | 1228 | - | 621658 | 622880 |  |  |  |  |  | 18 |  |  |  |  |  |  |  |  |
| 11 | PcContig791 | 2135 | - | 621717 | 623923 |  |  |  |  |  |  |  |  |  |  |  |  |  |  |
| 11 | MALPY03209 | 405 | + | 622803 | 623208 |  |  |  |  |  |  | 133 | 1800 |  |  |  |  |  |  |
| 11 | MALPY03082 | 1092 | - | 622858 | 623950 |  |  |  |  |  | 18 |  |  |  |  |  |  |  |  |
| man11 | MALPY00240 - Ins | 16868 | + | 624267 | 641376 |  |  |  |  |  | 18 | 133 | 1800 |  |  |  |  |  |  |
| man11 | Pc.c000202660.Contig1 - Ins | 8025 | - | 625922 | 634350 |  |  |  |  |  |  |  |  |  |  |  |  |  |  |
| 11 | Pb.c000701587.Contig1 | 3191 | - | 627297 | 630773 |  |  |  |  |  |  |  |  |  |  |  |  |  |  |
| 11 | Pb.c000701777.Contig1 | 890 | - | 630375 | 631265 |  |  |  |  |  |  |  |  |  |  |  |  |  |  |
| 11 | Pb.c000801832.Contig1 | 3456 | - | 632182 | 635705 | cRMPc09030 | 33298 | 2365 | 608078 | 641376 |  |  |  |  |  |  |  |  |  |
| man11 | MALPY00553 | 2362 | - | 643741 | 646322 | cRMPc09031 | 2581 | 702 | 643741 | 646322 | 1213 |  |  |  |  |  |  |  |  |
| 11 | MALPY01041 | 11057 | + | 647024 | 658378 |  |  |  |  |  | 1213 | 133 |  |  |  |  |  |  |  |
| 11 | PbContig7 | 3264 | + | 649585 | 652975 |  |  |  |  |  |  |  |  |  |  |  |  |  |  |
| 11 | Pc.c001103109.Contig1 | 3049 | + | 649856 | 653100 |  |  |  |  |  |  |  |  |  |  |  |  |  |  |
| 11 | Pb.c001402170.Contig1 | 1501 | + | 655935 | 657418 |  |  |  |  |  |  |  |  |  |  |  |  |  |  |
| 11 | Pc.c000303487.Contig1 | 1035 | - | 656553 | 657588 |  |  |  |  |  |  |  |  |  |  |  |  |  |  |
| 11 | Pb.c001502063.Contig1 | 3392 | + | 657604 | 660982 |  |  |  |  |  |  |  |  |  |  |  |  |  |  |
| 11 | MALPY02365 | 2284 | + | 658943 | 661230 | cRMPc09032 | 14206 | 783 | 647024 | 661230 |  |  |  | cRMPs0912 | 144486 | 783 | 516744 | 661230 |  |
| 11 | MALPY02632 | 3652 | - | 662013 | 665688 | cRMPc09033 | 3675 | 1382 | 662013 | 665688 |  | 236 | 1200 |  |  |  |  |  |  |
| 11 | MALPY01178 | 5242 | - | 667070 | 672621 |  |  |  |  |  | 580 | 236 |  |  |  |  |  |  |  |
| 11 | Pc.c001812313.Contig1 | 622 | - | 667377 | 667999 |  |  |  |  |  |  |  |  |  |  |  |  |  |  |
| 11 | Pb.c000801708.Contig1 | 2716 | - | 667543 | 670566 |  |  |  |  |  |  |  |  |  |  |  |  |  |  |
| 11 | Pc.c000603496.Contig1 | 857 | - | 668322 | 669281 |  |  |  |  |  |  |  |  |  |  |  |  |  |  |
| 11 | Pc.c000503589.Contig1 | 711 | - | 669414 | 670131 |  |  |  |  |  |  |  |  |  |  |  |  |  |  |
| 11 | Pc.c001203918.Contig1 | 1601 | - | 670738 | 672387 |  |  |  |  |  |  |  |  |  |  |  |  |  |  |
| 11 | Pb.c000601601.Contig1 | 1979 | - | 671339 | 673363 |  |  |  |  |  |  |  |  |  |  |  |  |  |  |
| TK11 | MALPY01345 | 2463 | + | 673091 | 675063 | cRMPc09034 | 7993 | 1885 | 667070 | 675063 | 580 |  |  | cRMPs0913 | 13050 | 1885 | 662013 | 675063 |  |
| 11 | MALPY02015 | 8423 | + | 676948 | 686238 |  |  |  |  |  |  | 168 | 1400 |  |  |  |  |  |  |
| 11 | Pb.c000101378.Contig1 | 1259 | + | 677301 | 678590 |  |  |  |  |  |  |  |  |  |  |  |  |  |  |
| 11 | Pb.c000101604.Contig1 | 1576 | + | 678809 | 680385 |  |  |  |  |  |  |  |  |  |  |  |  |  |  |
| 11 | MALPY03081 | 126 | - | 679462 | 679588 | cRMPc09035 | 9290 | 2000 | 676948 | 686238 |  | 168 | 2000 |  |  |  |  |  |  |
| Centromere - PCR 2000 bp | |  |  | 680000 |  |  |  |  |  |  |  |  |  |  |  |  |  |  |  |
| 11 | MALPY02459 | 7664 | - | 688238 | 696027 |  |  |  |  |  | 424 | 168 |  |  |  |  |  |  |  |
| 11 | Pb.c001402188.Contig1 | 3011 | + | 688382 | 691393 |  |  |  |  |  |  |  |  |  |  |  |  |  |  |
| 11 | Pc.c000202733.Contig1 | 2947 | - | 689041 | 692205 |  |  |  |  |  |  |  |  |  |  |  |  |  |  |
| 11 | Pc.c000203132.Contig1 | 1022 | - | 692095 | 693123 |  |  |  |  |  |  |  |  |  |  |  |  |  |  |
| 11 | Pb.c001904424.Contig1 | 1287 | + | 694435 | 695725 |  |  |  |  |  |  |  |  |  |  |  |  |  |  |
| 11 | MALPY01802 | 3320 | - | 695730 | 699050 |  |  |  |  |  | 424 |  |  |  |  |  |  |  |  |
| 11 | Pb.c001001725.Contig1 | 2981 | + | 695797 | 698778 | cRMPc09036 | 10812 | 612 | 688238 | 699050 |  |  |  | cRMPs0914 | 22102 | 612 | 676948 | 699050 |  |
| 11 | MALPY03041 | 921 | - | 699662 | 700601 | cRMPc09037 | 939 | 1323 | 699662 | 700601 |  |  |  | cRMPs0915 | 939 | 1323 | 699662 | 700601 |  |
| 11 | Pb.c001301890.Contig1 | 8858 | - | 701924 | 710823 |  |  |  |  |  |  |  |  |  |  |  |  |  |  |
| 11 | MALPY00923 | 14665 | - | 704740 | 720540 |  |  |  |  |  | 473 |  |  |  |  |  |  |  |  |
| 11 | Pc.c000503451.Contig1 | 668 | - | 706823 | 707506 |  |  |  |  |  |  |  |  |  |  |  |  |  |  |
| 11 | Pb.c001101841.Contig1 | 3462 | - | 711610 | 715248 |  |  |  |  |  |  |  |  |  |  |  |  |  |  |
| 11 | Pc.c001404838.Contig1 | 3595 | + | 714326 | 717863 |  |  |  |  |  |  |  |  |  |  |  |  |  |  |
| 11 | Pb.c000101384.Contig1 | 3843 | - | 715244 | 719252 |  |  |  |  |  |  |  |  |  |  |  |  |  |  |
| 11 | Pb.c001803395.Contig1 | 2036 | - | 719332 | 721368 |  |  |  |  |  |  |  |  |  |  |  |  |  |  |
| TK11 | MALPY02053 | 3104 | - | 720717 | 723761 | cRMPc09038 | 21837 | 1224 | 701924 | 723761 | 473 |  |  |  |  |  |  |  |  |
| 11 | MALPY01286 | 6027 | + | 724985 | 731097 |  |  |  |  |  | 473 |  |  |  |  |  |  |  |  |
| 11 | Pb.c001502085.Contig2 | 8081 | - | 725097 | 733382 |  |  |  |  |  |  |  |  |  |  |  |  |  |  |
| 11 | Pc.c001813118.Contig1 | 2496 | - | 729211 | 731750 |  |  |  |  |  |  |  |  |  |  |  |  |  |  |
| 11 | MALPY00022 | 20677 | - | 731287 | 753388 |  |  |  |  |  | 473 |  |  |  |  |  |  |  |  |
| 11 | Pc.c001812187.Contig1 | 1703 | - | 732679 | 734355 |  |  |  |  |  |  |  |  |  |  |  |  |  |  |
| 11 | MALPY03466 | 775 | - | 732740 | 733518 |  |  |  |  |  |  |  |  |  |  |  |  |  |  |
| 11 | MALPY03527 | 502 | - | 732840 | 733345 |  |  |  |  |  |  |  |  |  |  |  |  |  |  |
| 11 | Pb.c000201527.Contig1 | 7596 | - | 733426 | 741164 |  |  |  |  |  |  |  |  |  |  |  |  |  |  |
| 11 | Pc.c001812431.Contig1 | 833 | - | 736795 | 737628 |  |  |  |  |  |  |  |  |  |  |  |  |  |  |
| 11 | Pc.c001811707.Contig1 | 1848 | - | 737992 | 739792 |  |  |  |  |  |  |  |  |  |  |  |  |  |  |
| 11 | PbContig201 | 12685 | - | 742268 | 756179 |  |  |  |  |  |  |  |  |  |  |  |  |  |  |
| 11 | Pc.c001405283.Contig1 | 627 | + | 743474 | 744101 |  |  |  |  |  |  |  |  |  |  |  |  |  |  |
| 11 | Pc.c001405284.Contig1 | 1608 | + | 744057 | 746034 |  |  |  |  |  |  |  |  |  |  |  |  |  |  |
| 11 | MALPY03130 | 374 | - | 746282 | 746656 |  |  |  |  |  |  | 32 | 400 |  |  |  |  |  |  |
| 11 | Pc.c000303426.Contig1 | 3156 | + | 746365 | 749557 | cRMPc09039 | 31194 | 4700 | 724985 | 756179 |  |  |  |  |  |  |  |  |  |
| 11 | MALPY00006 | 9214 | + | 760879 | 771128 |  |  |  |  |  |  | 32 | 350 |  |  |  |  |  |  |
| 11 | Pb.c000401537.Contig1 | 2163 | + | 761771 | 763931 |  |  |  |  |  |  |  |  |  |  |  |  |  |  |
| 11 | PcContig534 | 5018 | - | 762872 | 769004 |  |  |  |  |  |  |  |  |  |  |  |  |  |  |
| 11 | Pb.c001001686.Contig1 | 5382 | + | 764961 | 771273 | cRMPc09040 | 10394 | 491 | 760879 | 771273 |  |  |  |  |  |  |  |  |  |
| 11 | Pc.c000903256.Contig1 | 2260 | - | 771764 | 773838 |  |  |  |  |  |  |  |  |  |  |  |  |  |  |
| 11 | MALPY02125 | 5481 | - | 772104 | 777914 |  |  |  |  |  |  | 32 |  |  |  |  |  |  |  |
| 11 | Pb.c001301734.Contig1 | 4851 | - | 772169 | 777473 |  |  |  |  |  |  |  |  |  |  |  |  |  |  |
| 11 | Pc.c001304268.Contig1 | 1998 | - | 774686 | 776639 |  |  |  |  |  |  |  |  |  |  |  |  |  |  |
| 11 | Pc.c000703384.Contig1 | 2851 | - | 776822 | 779743 |  |  |  |  |  |  |  |  |  |  |  |  |  |  |
| 11 | Pb.c001301918.Contig1 | 3095 | - | 777442 | 780523 |  |  |  |  |  |  |  |  |  |  |  |  |  |  |
| 11 | MALPY01333 | 3755 | - | 778247 | 781976 |  |  |  |  |  | 84 |  |  |  |  |  |  |  |  |
| 11 | Pb.c000100759.Contig1 | 13186 | - | 781177 | 795173 |  |  |  |  |  |  |  |  |  |  |  |  |  |  |
| 11 | MALPY00386 | 5039 | + | 782566 | 787644 |  |  |  |  |  | 84 |  |  |  |  |  |  |  |  |
| 11 | Pc.c000703302.Contig1 | 1866 | + | 783880 | 785821 |  |  |  |  |  |  |  |  |  |  |  |  |  |  |
| 11 | MALPY00387 | 7358 | - | 788839 | 795686 |  |  |  |  |  | 84 |  |  |  |  |  |  |  |  |
| 11 | Pc.c001606400.Contig1 | 854 | + | 790195 | 791055 |  |  |  |  |  |  |  |  |  |  |  |  |  |  |
| 11 | Pc.c001002762.Contig2 | 3413 | + | 791110 | 794163 | cRMPc09041 | 23922 | 214 | 771764 | 795686 |  |  |  |  |  |  |  |  |  |
| 11 | Pc.c000302944.Contig1 | 3458 | + | 795900 | 799599 |  |  |  |  |  |  |  |  |  |  |  |  |  |  |
| 11 | MALPY00887 | 3021 | + | 796100 | 799183 |  |  |  |  |  | 84 | 231 | 750 |  |  |  |  |  |  |
| 11 | Pb.c001001813.Contig1 | 3531 | + | 796406 | 799888 |  |  |  |  |  |  |  |  |  |  |  |  |  |  |
| 11 | MALPY03102 | 604 | - | 799028 | 799641 | cRMPc09042 | 3988 | 1069 | 795900 | 799888 |  | 231 | 6500 |  |  |  |  |  |  |
| 11 | MALPY02488 | 2091 | + | 800957 | 803174 |  |  |  |  |  | 281 | 231 |  |  |  |  |  |  |  |
| 11 | Pb.c000201535.Contig1 | 2448 | + | 801633 | 804355 |  |  |  |  |  |  |  |  |  |  |  |  |  |  |
| 11 | MALPY03084 | 983 | - | 802502 | 803529 |  |  |  |  |  |  |  |  |  |  |  |  |  |  |
| 11 | Pc.c001707846.Contig1 | 831 | + | 802922 | 803753 |  |  |  |  |  |  |  |  |  |  |  |  |  |  |
| 11 | MALPY03099 | 553 | - | 803273 | 803883 |  |  |  |  |  |  |  |  |  |  |  |  |  |  |
| 11 | MALPY00305 | 5737 | - | 803436 | 809502 |  |  |  |  |  | 281 | 197 | 4000 |  |  |  |  |  |  |
| 11 | PcContig550 | 4770 | - | 807251 | 813261 | cRMPc09043 | 12304 | 696 | 800957 | 813261 |  |  |  |  |  |  |  |  |  |
| JC11 | MALPY01659 | 3941 | + | 813957 | 817901 |  |  |  |  |  |  | 197 | 1400 |  |  |  |  |  |  |
| 11 | Pc.c000602861.Contig1 | 3462 | + | 815048 | 818510 |  |  |  |  |  |  |  |  |  |  |  |  |  |  |
| 11 | Pb.c001904830.Contig1 | 753 | - | 818074 | 818793 |  |  |  |  |  |  |  |  |  |  |  |  |  |  |
| 11 | MALPY00219 | 12207 | - | 818148 | 833094 |  |  |  |  |  | 219 | 197 |  |  |  |  |  |  |  |
| 11 | Pc.c000903318.Contig1 | 4818 | + | 820721 | 827995 |  |  |  |  |  |  |  |  |  |  |  |  |  |  |
| 11 | Pc.c000903368.Contig1 | 5448 | + | 830558 | 836006 |  |  |  |  |  |  |  |  |  |  |  |  |  |  |
| 11 | Pb.c000101302.Contig1 | 5012 | - | 830816 | 835822 |  |  |  |  |  |  |  |  |  |  |  |  |  |  |
| 11 | MALPY01017 | 7567 | + | 835199 | 843217 |  |  |  |  |  | 219 |  |  |  |  |  |  |  |  |
| 11 | Pb.c001702336.Contig1 | 2328 | - | 836323 | 838665 |  |  |  |  |  |  |  |  |  |  |  |  |  |  |
| 11 | Pc.c000802656.Contig1 | 6474 | - | 837352 | 844028 |  |  |  |  |  |  |  |  |  |  |  |  |  |  |
| 11 | Pb.c000301426.Contig1 | 2416 | + | 838882 | 841307 |  |  |  |  |  |  |  |  |  |  |  |  |  |  |
| man11 | MALPY02754 | 2388 | - | 842409 | 844797 | cRMPc09044 | 30840 | 535 | 813957 | 844797 | 219 |  |  |  |  |  |  |  | MALPY01017 |
| 11 | MALPY02239 | 6760 | - | 845332 | 853215 |  |  |  |  |  | 219 | 92 | 1200 |  |  |  |  |  |  |
| 11 | Pb.c002007800.Contig1 | 634 | + | 845788 | 846614 |  |  |  |  |  |  |  |  |  |  |  |  |  |  |
| 11 | Pc.c001707794.Contig1 | 1142 | + | 846318 | 847583 |  |  |  |  |  |  |  |  |  |  |  |  |  |  |
| 11 | Pb.c001402177.Contig1 | 4646 | + | 847074 | 852713 |  |  |  |  |  |  |  |  |  |  |  |  |  |  |
| 11 | Pc.c001707802.Contig1 | 1457 | + | 847731 | 849293 |  |  |  |  |  |  |  |  |  |  |  |  |  |  |
| 11 | Pc.c000302983.Contig1 | 2432 | + | 850425 | 852935 | cRMPc09045 | 7883 | 32 | 845332 | 853215 |  |  |  |  |  |  |  |  |  |
| 11 | Pc.c000602916.Contig1 | 4294 | + | 853247 | 857506 |  |  |  |  |  |  |  |  |  |  |  |  |  |  |
| 11 | Pb.c002007072.Contig1 | 1882 | + | 853609 | 855500 |  |  |  |  |  |  |  |  |  |  |  |  |  |  |
| 11 | MALPY00078 | 11183 | + | 853900 | 865190 |  |  |  |  |  | 265 | 92 |  |  |  |  |  |  |  |
| 11 | Pb.c002007062.Contig1 | 1810 | + | 855434 | 857223 |  |  |  |  |  |  |  |  |  |  |  |  |  |  |
| 11 | Pb.c001702871.Contig1 | 1223 | - | 858053 | 859255 |  |  |  |  |  |  |  |  |  |  |  |  |  |  |
| 11 | Pb.c001702921.Contig1 | 1346 | - | 859150 | 860385 |  |  |  |  |  |  |  |  |  |  |  |  |  |  |
| 11 | Pc.c001606390.Contig1 | 1209 | + | 859678 | 860768 |  |  |  |  |  |  |  |  |  |  |  |  |  |  |
| 11 | Pb.c001904326.Contig1 | 3777 | - | 860717 | 864610 |  |  |  |  |  |  |  |  |  |  |  |  |  |  |
| 11 | Pc.c000402246.Contig3 | 6365 | + | 861965 | 869136 |  |  |  |  |  |  |  |  |  |  |  |  |  |  |
| 11 | MALPY03316 | 285 | - | 863223 | 863517 |  |  |  |  |  |  | 194 | 2000 |  |  |  |  |  |  |
| 11 | Pb.c000101292.Contig1 | 4948 | - | 865159 | 870479 |  |  |  |  |  |  |  |  |  |  |  |  |  |  |
| 11 | MALPY02455 | 5273 | - | 865320 | 870965 |  |  |  |  |  | 265 | 194 |  |  |  |  |  |  |  |
| 11 | Pc.c000703232.Contig1 | 1539 | + | 869240 | 870779 |  |  |  |  |  |  |  |  |  |  |  |  |  |  |
| 11 | MALPY02197 | 5072 | - | 870188 | 875257 |  |  |  |  |  | 265 |  |  |  |  |  |  |  |  |
| 11 | Pb.c001402085.Contig1 | 2231 | + | 871269 | 873554 |  |  |  |  |  |  |  |  |  |  |  |  |  |  |
| 11 | Pc.c000903485.Contig1 | 2328 | + | 871549 | 873910 |  |  |  |  |  |  |  |  |  |  |  |  |  |  |
| 11 | Pb.c000501453.Contig1 | 4186 | + | 874409 | 878620 |  |  |  |  |  |  |  |  |  |  |  |  |  |  |
| 11 | Pc.c001104100.Contig1 | 2455 | + | 875606 | 878112 |  |  |  |  |  |  |  |  |  |  |  |  |  |  |
| 11 | MALPY02785 | 2253 | + | 875708 | 877991 | cRMPc09046 | 25373 | 2983 | 853247 | 878620 | 265 | 156 | 3000 |  |  |  |  |  |  |
| 11 | MALPY03077 | 228 | + | 881603 | 881822 | cRMPc09047 | 219 | 3791 | 881603 | 881822 |  | 156 | 2200 |  |  |  |  |  |  |
| 11 | MALPY01946 | 4497 | + | 885613 | 890161 |  |  |  |  |  | 499 | 156 |  |  |  |  |  |  |  |
| 11 | Pc.c001103797.Contig1 | 1433 | - | 887954 | 889399 | cRMPc09048 | 4548 | 362 | 885613 | 890161 |  |  |  |  |  |  |  |  |  |
| TK11 | MALPY01542 | 8505 | - | 890523 | 892811 | cRMPc09049 | 2288 | 1469 | 890523 | 892811 | 499 |  |  | cRMPs0916 | 190887 | 1469 | 701924 | 892811 |  |
| 11 | Pc.c000402944.Contig1 | 1529 | - | 894280 | 895800 | cRMPc09050 | 1520 | 4120 | 894280 | 895800 |  |  |  | cRMPs0917 | 1520 | 4120 | 894280 | 895800 |  |
| 11 | MALPY00584 | 10092 | - | 899920 | 909998 |  |  |  |  |  |  | 24 | 1600 |  |  |  |  |  |  |
| 11 | Pb.c000201234.Contig1 | 2521 | + | 901863 | 904390 |  |  |  |  |  |  |  |  |  |  |  |  |  |  |
| 11 | Pc.c000002554.Contig1 | 2177 | + | 903038 | 905194 |  |  |  |  |  |  |  |  |  |  |  |  |  |  |
| 11 | Pb.c000901880.Contig1 | 3651 | + | 904744 | 908395 |  |  |  |  |  |  |  |  |  |  |  |  |  |  |
| 11 | Pc.c000503501.Contig1 | 992 | + | 906792 | 907784 |  |  |  |  |  |  |  |  |  |  |  |  |  |  |
| 11 | PcContig327 | 5943 | + | 909217 | 915148 |  |  |  |  |  |  |  |  |  |  |  |  |  |  |
| 11 | MALPY01776 | 3377 | + | 912341 | 915691 |  |  |  |  |  |  | 24 | 2000 |  |  |  |  |  |  |
| 11 | Pb.c001302124.Contig1 | 1279 | - | 913146 | 914392 |  |  |  |  |  |  |  |  |  |  |  |  |  |  |
| 11 | PbContig4 | 3973 | - | 914143 | 918170 |  |  |  |  |  |  |  |  |  |  |  |  |  |  |
| 11 | MALPY01905 | 3989 | - | 916570 | 920405 | cRMPc09051 | 20485 | 1440 | 899920 | 920405 |  | 24 | 5000 |  |  |  |  |  |  |
| TK11 | MALPY01033 | 2767 | + | 921845 | 924085 | cRMPc09052 | 2240 | 517 | 921845 | 924085 | 102 |  |  |  |  |  |  |  |  |
| 11 | MALPY01032 | 9084 | + | 924602 | 933790 |  |  |  |  |  | 102 | 24 |  |  |  |  |  |  |  |
| 11 | Pb.c001302021.Contig1 | 6089 | - | 924677 | 931132 |  |  |  |  |  |  |  |  |  |  |  |  |  |  |
| 11 | Pc.c001204064.Contig1 | 1721 | - | 924967 | 926688 |  |  |  |  |  |  |  |  |  |  |  |  |  |  |
| 11 | PcContig264 | 4531 | - | 928650 | 933151 | cRMPc09053 | 9188 | 2026 | 924602 | 933790 |  |  |  |  |  |  |  |  |  |
| 11 | Pc.c001003886.Contig1 | 4912 | - | 935816 | 940932 |  |  |  |  |  |  |  |  |  |  |  |  |  |  |
| 11 | MALPY00593 | 2804 | + | 936110 | 938902 |  |  |  |  |  | 102 |  |  |  |  |  |  |  |  |
| 11 | Pb.c001402090.Contig1 | 2108 | - | 936646 | 938724 |  |  |  |  |  |  |  |  |  |  |  |  |  |  |
| 11 | MALPY00762 | 11724 | + | 939460 | 951235 |  |  |  |  |  |  |  |  |  |  |  |  |  |  |
| 11 | Pc.c000002104.Contig2 | 3436 | - | 941022 | 944452 |  |  |  |  |  |  |  |  |  |  |  |  |  |  |
| 11 | Pb.c002006878.Contig1 | 2120 | + | 941232 | 943193 |  |  |  |  |  |  |  |  |  |  |  |  |  |  |
| 11 | Pc.c000102673.Contig1 | 8644 | - | 946731 | 956202 |  |  |  |  |  |  |  |  |  |  |  |  |  |  |
| 11 | Pb.c000501196.Contig1 | 6388 | + | 948124 | 954876 |  |  |  |  |  |  |  |  |  |  |  |  |  |  |
| 11 | MALPY03160 | 922 | - | 948468 | 949390 |  |  |  |  |  |  | 325 | 1000 |  |  |  |  |  |  |
| 11 | MALPY01949 | 9951 | + | 950047 | 960329 |  |  |  |  |  | 122 | 325 |  |  |  |  |  |  |  |
| 11 | Pb.c000801671.Contig1 | 6227 | + | 955116 | 962035 |  |  |  |  |  |  |  |  |  |  |  |  |  |  |
| 11 | Pc.c001203899.Contig1 | 1616 | - | 958210 | 959826 |  |  |  |  |  |  |  |  |  |  |  |  |  |  |
| 11 | MALPY03575 | 705 | + | 958900 | 959555 |  |  |  |  |  |  |  |  |  |  |  |  |  |  |
| 11 | Pc.c000703907.Contig1 | 928 | - | 960535 | 961463 |  |  |  |  |  |  |  |  |  |  |  |  |  |  |
| 11 | MALPY01002 | 4579 | + | 960937 | 965054 |  |  |  |  |  | 122 |  |  |  |  |  |  |  |  |
| 11 | Pc.c000703367.Contig1 | 1217 | - | 961178 | 962512 |  |  |  |  |  |  |  |  |  |  |  |  |  |  |
| 11 | Pb.c000001773.Contig1 | 2302 | - | 962299 | 964059 |  |  |  |  |  |  |  |  |  |  |  |  |  |  |
| 11 | MALPY01003 | 3957 | - | 963981 | 967938 | cRMPc09054 | 32122 | 1500 | 935816 | 967938 | 122 | 119 - NS | 1500 |  |  |  |  |  |  |
| 11 | MALPY00801 | 2003 | + | 969438 | 971438 |  |  |  |  |  |  | 119 - NS | 500 |  |  |  |  |  |  |
| 11 | Pb.c001301825.Contig1 | 1953 | + | 970624 | 972803 |  |  |  |  |  |  |  |  |  |  |  |  |  |  |
| 11 | MALPY03059 | 215 | - | 971696 | 971911 |  |  |  |  |  |  | 119 | 1400 |  |  |  |  |  |  |
| 11 | MALPY01556 | 9004 | - | 972686 | 981657 |  |  |  |  |  | 428 | 119 | 3000 |  |  |  |  |  |  |
| 11 | Pb.c001101558.Contig1 | 4672 | + | 972921 | 977614 |  |  |  |  |  |  |  |  |  |  |  |  |  |  |
| 11 | Pc.c000402910.Contig1 | 3737 | - | 973987 | 977698 | cRMPc09055 | 12219 | 3000 | 969438 | 981657 |  |  |  |  |  |  |  |  |  |
| JC11 | MALPY00609 | 4444 | xxx | 980000 |  |  |  |  |  |  | 428 |  |  |  |  |  |  |  |  |
| 11 | MALPY02438 | 8937 | - | 984657 | 993454 |  |  |  |  |  |  | 119 | 1000 |  |  |  |  |  |  |
| 11 | Pb.c000801822.Contig1 | 4084 | + | 984967 | 989116 |  |  |  |  |  |  |  |  |  |  |  |  |  |  |
| 11 | Pc.c000802664.Contig2 | 3052 | + | 986863 | 989974 |  |  |  |  |  |  |  |  |  |  |  |  |  |  |
| 11 | Pb.c001803455.Contig1 | 3618 | + | 989346 | 992604 |  |  |  |  |  |  |  |  |  |  |  |  |  |  |
| 11 | PcContig51 | 3826 | + | 990261 | 993760 | cRMPc09056 | 9103 | 437 | 984657 | 993760 |  |  |  |  |  |  |  |  |  |
| 11 | MALPY01832 | 9192 | - | 994197 | 1003068 |  |  |  |  |  |  | 119 | 1200 |  |  |  |  |  |  |
| 11 | Pb.c001702331.Contig1 | 3411 | + | 996988 | 1000396 |  |  |  |  |  |  |  |  |  |  |  |  |  |  |
| 11 | Pc.c000402782.Contig1 | 2855 | + | 997122 | 999965 |  |  |  |  |  |  |  |  |  |  |  |  |  |  |
| 11 | Pc.c000503453.Contig1 | 918 | - | 1000993 | 1001824 |  |  |  |  |  |  |  |  |  |  |  |  |  |  |
| 11 | Pb.c001301492.Contig2 | 5716 | - | 1001792 | 1007520 |  |  |  |  |  |  |  |  |  |  |  |  |  |  |
| 11 | MALPY03301 | 1033 | + | 1004049 | 1005082 |  |  |  |  |  | 313 | 119 |  |  |  |  |  |  |  |
| 11 | MALPY01632 | 7224 | + | 1005592 | 1012819 |  |  |  |  |  | 313 | 25 | 1400 |  |  |  |  |  |  |
| 11 | Pc.c000603075.Contig1 | 1787 | + | 1007339 | 1009114 |  |  |  |  |  |  |  |  |  |  |  |  |  |  |
| 11 | Pb.c001904327.Contig1 | 1922 | - | 1008295 | 1010223 | cRMPc09057 | 18622 | 1654 | 994197 | 1012819 |  |  |  |  |  |  |  |  |  |
| 11 | MALPY00586 | 7757 | + | 1014473 | 1022404 |  |  |  |  |  | 199 | 25 |  |  |  |  |  |  |  |
| 11 | Pb.c001803313.Contig1 | 11925 | - | 1015034 | 1027400 |  |  |  |  |  |  |  |  |  |  |  |  |  |  |
| 11 | Pc.c001404772.Contig1 | 2500 | - | 1022052 | 1024552 |  |  |  |  |  |  |  |  |  |  |  |  |  |  |
| 11 | MALPY00181 | 16600 | - | 1022634 | 1040686 |  |  |  |  |  | 199 |  |  |  |  |  |  |  |  |
| 11 | Pc.c000803322.Contig1 | 5672 | - | 1024911 | 1030547 |  |  |  |  |  |  |  |  |  |  |  |  |  |  |
| 11 | Pb.c000301445.Contig1 | 1138 | - | 1027577 | 1028679 |  |  |  |  |  |  |  |  |  |  |  |  |  |  |
| 11 | PbContig55 | 7222 | - | 1031261 | 1039472 |  |  |  |  |  |  |  |  |  |  |  |  |  |  |
| 11 | PcContig643 | 7103 | - | 1034781 | 1042157 | cRMPc09058 | 27684 | 668 | 1014473 | 1042157 |  |  |  | cRMPs0918 | 142237 | 668 | 899920 | 1042157 |  |
| 11 | MALPY02199 | 3082 | + | 1042825 | 1045871 |  |  |  |  |  |  | 13 | 1200 |  |  |  |  |  |  |
| 11 | Pc.c001404685.Contig1 | 3486 | - | 1045526 | 1049010 |  |  |  |  |  |  |  |  |  |  |  |  |  |  |
| 11 | Pb.c002005814.Contig1 | 3566 | + | 1045557 | 1049120 |  |  |  |  |  |  |  |  |  |  |  |  |  |  |
| 11 | MALPY00307 | 7616 | + | 1045963 | 1053576 | cRMPc09059 | 10751 | 3822 | 1042825 | 1053576 |  | 13 | 3500 |  |  |  |  |  |  |
| 11 | MALPY02027 | 9474 | - | 1057398 | 1068347 |  |  |  |  |  | 263 | 13 - Ins | 4000 |  |  |  |  |  |  |
| 11 | Pc.c000002687.Contig1 | 2756 | - | 1066611 | 1069382 |  |  |  |  |  |  |  |  |  |  |  |  |  |  |
| TK11 | MALPY02611 | 2938 | - | 1068386 | 1071249 | cRMPc09060 | 13851 | 118 | 1057398 | 1071249 | 263 |  |  |  |  |  |  |  |  |
| 11 | Pb.c001402059.Contig1 | 7218 | - | 1071367 | 1079642 |  |  |  |  |  |  |  |  |  |  |  |  |  |  |
| 11 | MALPY01253 | 5143 | + | 1072347 | 1078549 |  |  |  |  |  |  | 13 - Ins | 5000 |  |  |  |  |  |  |
| 11 | Pc.c000502426.Contig1 | 2458 | + | 1073574 | 1076266 |  |  |  |  |  |  |  |  |  |  |  |  |  |  |
| 11 | Pc.c001505080.Contig1 | 1432 | - | 1078220 | 1079649 | cRMPc09061 | 8282 | 2749 | 1071367 | 1079649 |  |  |  |  |  |  |  |  |  |
| TK11 | MALPY02274 | 2691 | + | 1082398 | 1085023 | cRMPc09062 | 2625 | 630 | 1082398 | 1085023 | 120 |  |  |  |  |  |  |  |  |
| 11 | MALPY01066 | 10614 | - | 1085653 | 1096597 |  |  |  |  |  | 120 | 13 |  |  |  |  |  |  |  |
| 11 | Pb.c001001409.Contig3 | 5292 | + | 1086227 | 1091434 |  |  |  |  |  |  |  |  |  |  |  |  |  |  |
| 11 | Pc.c000103097.Contig1 | 1162 | + | 1087565 | 1088739 |  |  |  |  |  |  |  |  |  |  |  |  |  |  |
| 11 | PcContig705 | 6376 | + | 1088708 | 1095792 |  |  |  |  |  |  |  |  |  |  |  |  |  |  |
| 11 | Pb.c001202132.Contig1 | 2955 | + | 1094146 | 1097255 |  |  |  |  |  |  |  |  |  |  |  |  |  |  |
| 11 | MALPY03444 | 824 | + | 1095015 | 1095842 |  |  |  |  |  |  | 205 | 700 |  |  |  |  |  |  |
| 11 | MALPY02633 | 2154 | + | 1095768 | 1098045 |  |  |  |  |  | 120 | 205 | 1400 |  |  |  |  |  |  |
| 11 | Pc.c000803376.Contig1 | 4064 | - | 1095794 | 1100381 |  |  |  |  |  |  |  |  |  |  |  |  |  |  |
| 11 | MALPY02590 | 6204 | - | 1099353 | 1106807 |  |  |  |  |  | 896 | 205 | 2800 |  |  |  |  |  |  |
| 11 | Pc.c001606637.Contig1 | 614 | - | 1103692 | 1104306 |  |  |  |  |  |  |  |  |  |  |  |  |  |  |
| 11 | PcContig755 | 1800 | - | 1104293 | 1106093 |  |  |  |  |  |  |  |  |  |  |  |  |  |  |
| 11 | Pb.c000001711.Contig1 | 2864 | + | 1104447 | 1107311 | cRMPc09063 | 21658 | 580 | 1085653 | 1107311 |  |  |  |  |  |  |  |  |  |
| JC11 | MALPY02282 | 2436 | xxx | 1105000 |  |  |  |  |  |  | 896 |  |  |  |  |  |  |  |  |
| JC11 | MALPY02284 | 2737 | xxx | 1106000 |  |  |  |  |  |  | 896 |  |  |  |  |  |  |  |  |
| JC11 | MALPY02677 | 3131 | xxx | 1107000 |  |  |  |  |  |  | 896 |  |  |  |  |  |  |  |  |
| 11 | Pc.c001103850.Contig1 | 2172 | - | 1107891 | 1110147 | cRMPc09064 | 2256 | 467 | 1107891 | 1110147 |  |  |  |  |  |  |  |  |  |
| 11 | MALPY01963 | 9567 | - | 1110614 | 1121199 |  |  |  |  |  |  | 205 | 1500 |  |  |  |  |  |  |
| 11 | Pb.c000401517.Contig1 | 1902 | + | 1112453 | 1114368 |  |  |  |  |  |  |  |  |  |  |  |  |  |  |
| 11 | Pb.c002112205.Contig1 | 895 | - | 1115012 | 1115899 |  |  |  |  |  |  |  |  |  |  |  |  |  |  |
| 11 | Pb.c000901434.Contig1 | 2793 | - | 1117308 | 1120092 |  |  |  |  |  |  |  |  |  |  |  |  |  |  |
| 11 | Pc.c001203870.Contig1 | 1299 | + | 1120000 | 1121299 |  |  |  |  |  |  |  |  |  |  |  |  |  |  |
| 11 | Pb.c000901783.Contig1 | 4418 | - | 1120360 | 1124778 |  |  |  |  |  |  |  |  |  |  |  |  |  |  |
| 11 | MALPY00275 | 9444 | + | 1122131 | 1131658 |  |  |  |  |  | 773 | 205 | 1000 |  |  |  |  |  |  |
| 11 | Pb.c001001715.Contig1 | 3952 | - | 1125774 | 1129886 |  |  |  |  |  |  |  |  |  |  |  |  |  |  |
| 11 | PcContig145 | 3164 | - | 1127104 | 1130449 | cRMPc09065 | 21044 | 2498 | 1110614 | 1131658 |  |  |  |  |  |  |  |  |  |
| JC11 | MALPY00882 | 2224 | xxx | 1130000 |  |  |  |  |  |  | 773 |  |  |  |  |  |  |  |  |
| 11 | MALPY00214 | 3841 | + | 1134156 | 1137994 |  |  |  |  |  |  | 205 | 1700 |  |  |  |  |  |  |
| 11 | Pb.c001301913.Contig1 | 6440 | + | 1136017 | 1143127 |  |  |  |  |  |  |  |  |  |  |  |  |  |  |
| 11 | MALPY00425 | 4801 | + | 1139861 | 1144124 |  |  |  |  |  |  | 205 | 4500 |  |  |  |  |  |  |
| 11 | Pc.c001404757.Contig1 | 1128 | + | 1142893 | 1144021 |  |  |  |  |  |  |  |  |  |  |  |  |  |  |
| 11 | Pb.c001502846.Contig1 | 951 | + | 1144065 | 1145016 | cRMPc09066 | 10860 | 3259 | 1134156 | 1145016 |  |  |  |  |  |  |  |  |  |
| TK11 | MALPY00069 | 4733 | - | 1148275 | 1153056 |  |  |  |  |  | 910B |  |  |  |  |  |  |  |  |
| 11 | Pc.c000202797.Contig1 | 11236 | - | 1151726 | 1163225 |  |  |  |  |  |  |  |  |  |  |  |  |  |  |
| 11 | Pb.c001702730.Contig1 | 4294 | + | 1155761 | 1160286 |  |  |  |  |  |  |  |  |  |  |  |  |  |  |
| 11 | MALPY01681 | 8664 | - | 1155852 | 1164539 |  |  |  |  |  | 910B | 205 | 2000 |  |  |  |  |  |  |
| 11 | Pb.c001803670.Contig1 | 2093 | + | 1160615 | 1162693 |  |  |  |  |  |  |  |  |  |  |  |  |  |  |
| 11 | Pb.c001802788.Contig1 | 1638 | - | 1161072 | 1162695 |  |  |  |  |  |  |  |  |  |  |  |  |  |  |
| 11 | Pb.c001402156.Contig1 | 2125 | - | 1162261 | 1164470 |  |  |  |  |  |  |  |  |  |  |  |  |  |  |
| 11 | Pc.c000402788.Contig1 | 1531 | - | 1162793 | 1164357 | cRMPc09067 | 16264 | 1001 | 1148275 | 1164539 |  |  |  |  |  |  |  |  |  |
| 11 | Pb.c001803362.Contig1 | 1838 | - | 1165540 | 1167378 |  |  |  |  |  |  |  |  |  |  |  |  |  |  |
| 11 | PcContig465 | 1542 | - | 1165699 | 1167241 |  |  |  |  |  |  |  |  |  |  |  |  |  |  |
| 11 | MALPY03291 | 1630 | + | 1166131 | 1167746 |  |  |  |  |  |  |  |  |  |  |  |  |  |  |
| 11 | MALPY02466 | 5427 | - | 1166159 | 1171654 |  |  |  |  |  | 358 | 205 |  |  |  |  |  |  |  |
| 11 | Pc.c001708477.Contig1 | 583 | - | 1167467 | 1168050 |  |  |  |  |  |  |  |  |  |  |  |  |  |  |
| 11 | Pb.c000301490.Contig1 | 936 | + | 1167597 | 1168533 |  |  |  |  |  |  |  |  |  |  |  |  |  |  |
| 11 | PcContig65 | 1925 | - | 1168210 | 1170249 |  |  |  |  |  |  |  |  |  |  |  |  |  |  |
| 11 | Pb.c000301171.Contig2 | 7074 | + | 1168487 | 1175481 |  |  |  |  |  |  |  |  |  |  |  |  |  |  |
| 11 | Pc.c001003371.Contig1 | 2786 | + | 1170841 | 1173636 |  |  |  |  |  |  |  |  |  |  |  |  |  |  |
| 11 | MALPY00526 | 19188 | + | 1171600 | 1190776 |  |  |  |  |  | 358 | 261 | 1500 |  |  |  |  |  |  |
| 11 | Pb.c001201698.Contig1 | 5503 | + | 1184267 | 1189761 |  |  |  |  |  |  |  |  |  |  |  |  |  |  |
| 11 | Pc.c000202822.Contig1 | 4677 | + | 1186691 | 1191384 | cRMPc09068 | 25844 | 1181 | 1165540 | 1191384 |  |  |  |  |  |  |  |  |  |
| man11 | Pb.c000501195.Contig2 - Ins | 14799 | + | 1192565 | 1209653 |  |  |  |  |  |  |  |  |  |  |  |  |  |  |
| 11 | MALPY01868 | 3092 | - | 1193515 | 1196640 |  |  |  |  |  | 226 | 261 |  |  |  |  |  |  |  |
| 11 | Pc.c001304296.Contig1 | 1412 | + | 1194771 | 1196216 |  |  |  |  |  |  |  |  |  |  |  |  |  |  |
| 11 | MALPY01567 | 6363 | - | 1196152 | 1202788 |  |  |  |  |  | 226 | 326 | 3000 |  |  |  |  |  |  |
| 11 | Pc.c000402780.Contig1 | 1575 | + | 1196675 | 1198247 |  |  |  |  |  |  |  |  |  |  |  |  |  |  |
| 11 | MALPY03361 | 217 | + | 1197758 | 1197972 |  |  |  |  |  |  | 326 |  |  |  |  |  |  |  |
| 11 | Pc.c000602849.Contig1 | 6229 | + | 1198427 | 1206407 |  |  |  |  |  |  |  |  |  |  |  |  |  |  |
| 11 | MALPY03341 | 446 | - | 1199009 | 1199440 |  |  |  |  |  |  |  |  |  |  |  |  |  |  |
| 11 | MALPY00132 | 7294 | + | 1204595 | 1211868 | cRMPc09069 | 19303 | 326 | 1192565 | 1211868 |  |  |  | cRMPs0919 | 169043 | 326 | 1042825 | 1211868 |  |
| man11 | MALPY00553 | 10433 | - | 1212194 | 1221861 | cRMPc09070 | 9667 | 2703 | 1212194 | 1221861 |  |  |  | cRMPs0920 | 9667 | 2703 | 1212194 | 1221861 |  |
| 11 | PbContig259 | 8705 | - | 1224564 | 1234142 |  |  |  |  |  |  |  |  |  |  |  |  |  |  |
| 11 | MALPY00851 | 15028 | + | 1225377 | 1240641 |  |  |  |  |  |  |  |  |  |  |  |  |  |  |
| 11 | MALPY02992 | 623 | + | 1225970 | 1226593 |  |  |  |  |  |  |  |  |  |  |  |  |  |  |
| 11 | Pc.c001103701.Contig1 | 3218 | + | 1229590 | 1232790 |  |  |  |  |  |  |  |  |  |  |  |  |  |  |
| 11 | Pc.c000102666.Contig1 | 1472 | + | 1233526 | 1234986 |  |  |  |  |  |  |  |  |  |  |  |  |  |  |
| 11 | PbContig257 | 4851 | - | 1233723 | 1238493 |  |  |  |  |  |  |  |  |  |  |  |  |  |  |
| 11 | Pc.c001811692.Contig1 | 1053 | + | 1236251 | 1237304 |  |  |  |  |  |  |  |  |  |  |  |  |  |  |
| 11 | Pc.c001812284.Contig1 | 787 | + | 1237500 | 1238228 | cRMPc09071 | 16077 | 1556 | 1224564 | 1240641 |  |  |  | cRMPs0921 | 16077 | 1556 | 1224564 | 1240641 |  |
| 11 | MALPY00321 | 25736 | + | 1242197 | 1268385 |  |  |  |  |  |  | 347 | 700 |  |  |  |  |  |  |
| 11 | Pb.c001502024.Contig1.1 | 7517 | - | 1250188 | 1257581 |  |  |  |  |  |  |  |  |  |  |  |  |  |  |
| 11 | PcContig585 | 7263 | - | 1259220 | 1266763 |  |  |  |  |  |  |  |  |  |  |  |  |  |  |
| 11 | PbContig405 | 7316 | - | 1260443 | 1268018 | cRMPc09072 | 26188 | 1115 | 1242197 | 1268385 |  |  |  |  |  |  |  |  |  |
| 11 | MALPY01993 | 8135 | + | 1269500 | 1277606 |  |  |  |  |  | 1348 | 347 |  |  |  |  |  |  |  |
| 11 | Pc.c001505587.Contig1 | 617 | - | 1271017 | 1271645 |  |  |  |  |  |  |  |  |  |  |  |  |  |  |
| 11 | Pb.c001402091.Contig1 | 2251 | + | 1271963 | 1274147 |  |  |  |  |  |  |  |  |  |  |  |  |  |  |
| 11 | Pc.c001812519.Contig1 | 901 | - | 1271969 | 1272764 | cRMPc09073 | 8106 | 2199 | 1269500 | 1277606 |  |  |  |  |  |  |  |  |  |
| 11 | MALPY01872 | 13244 | + | 1279805 | 1293078 |  |  |  |  |  | 1348 | 265 | 700 |  |  |  |  |  |  |
| 11 | Pc.c000002109.Contig3 | 5936 | + | 1280888 | 1286824 | cRMPc09074 | 13273 | 1082 | 1279805 | 1293078 |  |  |  |  |  |  |  |  |  |
| 11 | MALPY00319 | 17087 | - | 1294160 | 1311952 |  |  |  |  |  | 159 | 265 |  |  |  |  |  |  |  |
| 11 | Pb.c000101348.Contig1 | 2905 | + | 1294291 | 1297253 |  |  |  |  |  |  |  |  |  |  |  |  |  |  |
| 11 | Pc.c000503493.Contig1 | 1193 | - | 1296315 | 1297503 |  |  |  |  |  |  |  |  |  |  |  |  |  |  |
| 11 | Pc.c000402840.Contig1 | 5065 | - | 1298416 | 1303785 |  |  |  |  |  |  |  |  |  |  |  |  |  |  |
| 11 | PbContig160 | 7061 | + | 1299932 | 1306257 |  |  |  |  |  |  |  |  |  |  |  |  |  |  |
| 11 | Pc.c001104336.Contig1.1 | 332 | - | 1304957 | 1305289 |  |  |  |  |  |  |  |  |  |  |  |  |  |  |
| 11 | Pb.c001401800.Contig2 | 7425 | + | 1307087 | 1315113 |  |  |  |  |  |  |  |  |  |  |  |  |  |  |
| 11 | Pc.c000002115.Contig2 | 4294 | + | 1308068 | 1312972 |  |  |  |  |  |  |  |  |  |  |  |  |  |  |
| 11 | MALPY00670 | 8881 | - | 1310809 | 1320369 |  |  |  |  |  | 159 |  |  |  |  |  |  |  |  |
| 11 | PcContig544 | 4424 | + | 1313526 | 1317968 |  |  |  |  |  |  |  |  |  |  |  |  |  |  |
| 11 | Pb.c000901745.Contig1 | 1615 | + | 1315916 | 1317579 | cRMPc09075 | 26209 | 737 | 1294160 | 1320369 |  |  |  |  |  |  |  |  |  |
| TK11 | MALPY01575 | 3974 | + | 1321106 | 1324628 | cRMPc09076 | 3522 | 987 | 1321106 | 1324628 | 159 |  |  |  |  |  |  |  |  |
| 11 | MALPY02179 | 11861 | - | 1325615 | 1338473 |  |  |  |  |  | 159 | 312 | 1400 |  |  |  |  |  |  |
| 11 | Pb.c000401193.Contig1 | 5593 | + | 1332259 | 1337855 |  |  |  |  |  |  |  |  |  |  |  |  |  |  |
| 11 | Pc.c001605948.Contig1 | 1965 | + | 1335212 | 1337180 |  |  |  |  |  |  |  |  |  |  |  |  |  |  |
| 11 | PbContig99 | 6944 | - | 1338281 | 1345614 |  |  |  |  |  |  |  |  |  |  |  |  |  |  |
| 11 | MALPY01360 | 4714 | - | 1338880 | 1343875 |  |  |  |  |  | 707 | 312 |  |  |  |  |  |  |  |
| 11 | MALPY03264 | 640 | - | 1340377 | 1341008 |  |  |  |  |  |  | 202 | 3000 |  |  |  |  |  |  |
| 11 | Pc.c001405298.Contig1 | 995 | + | 1340462 | 1341457 |  |  |  |  |  |  |  |  |  |  |  |  |  |  |
| 11 | Pc.c001405373.Contig1 | 983 | + | 1341598 | 1342842 |  |  |  |  |  |  |  |  |  |  |  |  |  |  |
| 11 | Pc.c001813169.Contig1 | 774 | + | 1342834 | 1343608 |  |  |  |  |  |  |  |  |  |  |  |  |  |  |
| 11 | MALPY01363 | 10072 | - | 1344120 | 1354948 |  |  |  |  |  | 707 | 202 |  |  |  |  |  |  |  |
| 11 | MALPY03376 | 763 | - | 1345293 | 1346056 |  |  |  |  |  |  | 163 | 1300 |  |  |  |  |  |  |
| 11 | Pb.c000301447.Contig1 | 4809 | + | 1345626 | 1351161 |  |  |  |  |  |  |  |  |  |  |  |  |  |  |
| JC11 | MALPY03221 | 766 | + | 1346782 | 1347551 |  |  |  |  |  |  | 163 | 6500 |  |  |  |  |  |  |
| 11 | Pc.c001304759.Contig1 | 626 | - | 1349290 | 1349832 | cRMPc09077 | 29333 | 176 | 1325615 | 1354948 |  |  |  |  |  |  |  |  |  |
| 11 | MALPY02393 | 8445 | - | 1355124 | 1364010 |  |  |  |  |  | 45 | 163 | 6500 |  |  |  |  |  |  |
| 11 | Pc.c001405208.Contig1 | 1630 | + | 1355359 | 1356989 |  |  |  |  |  |  |  |  |  |  |  |  |  |  |
| 11 | Pb.c001502569.Contig1 | 3858 | - | 1355934 | 1360203 |  |  |  |  |  |  |  |  |  |  |  |  |  |  |
| 11 | Pc.c000903914.Contig1 | 1010 | + | 1357011 | 1358021 |  |  |  |  |  |  |  |  |  |  |  |  |  |  |
| 11 | Pc.c001606274.Contig1 | 674 | + | 1358453 | 1359090 |  |  |  |  |  |  |  |  |  |  |  |  |  |  |
| 11 | Pc.c001505160.Contig1 | 1194 | + | 1359122 | 1360313 | cRMPc09078 | 8886 | 317 | 1355124 | 1364010 |  |  |  |  |  |  |  |  |  |
| TK11 | MALPY00750 | 4465 | - | 1364327 | 1369015 | cRMPc09079 | 4688 | 1803 | 1364327 | 1369015 | 45 |  |  |  |  |  |  |  |  |
| 11 | Pb.c001101518.Contig1 | 6552 | - | 1370818 | 1377291 |  |  |  |  |  |  |  |  |  |  |  |  |  |  |
| 11 | MALPY02594 | 2859 | - | 1371480 | 1374362 | cRMPc09080 | 6473 | 5664 | 1370818 | 1377291 |  | 163 | 4000 |  |  |  |  |  |  |
| 11 | Pb.c001001649.Contig1 | 9895 | - | 1382955 | 1392970 |  |  |  |  |  |  |  |  |  |  |  |  |  |  |
| 11 | MALPY01928 | 11069 | - | 1383328 | 1394514 |  |  |  |  |  |  | 163 |  |  |  |  |  |  |  |
| 11 | PcContig119 | 3823 | + | 1388960 | 1392913 |  |  |  |  |  |  |  |  |  |  |  |  |  |  |
| 11 | MALPY03544 | 794 | - | 1389353 | 1390174 |  |  |  |  |  |  | 5 | 4300 |  |  |  |  |  |  |
| 11 | Pb.c001301779.Contig1 | 4841 | - | 1393441 | 1398654 |  |  |  |  |  |  |  |  |  |  |  |  |  |  |
| 11 | MALPY00208 | 8340 | - | 1394748 | 1403906 |  |  |  |  |  |  | 5 | 4300 |  |  |  |  |  |  |
| 11 | Pc.c001304778.Contig1 | 1070 | - | 1398678 | 1399649 |  |  |  |  |  |  |  |  |  |  |  |  |  |  |
| 11 | Pb.c000801792.Contig1 | 4004 | - | 1398729 | 1403203 |  |  |  |  |  |  |  |  |  |  |  |  |  |  |
| 11 | Pc.c001812240.Contig1 | 1002 | - | 1401062 | 1402067 | cRMPc09081 | 20951 | 8604 | 1382955 | 1403906 |  |  |  |  |  |  |  |  |  |
| 11 | MALPY00570 | 12667 | + | 1412510 | 1425088 |  |  |  |  |  |  | 5 | 2100 |  |  |  |  |  |  |
| 11 | Pb.c000301467.Contig1 | 4507 | - | 1412847 | 1417791 |  |  |  |  |  |  |  |  |  |  |  |  |  |  |
| 11 | Pc.c000402897.Contig1 | 1686 | + | 1413727 | 1415395 |  |  |  |  |  |  |  |  |  |  |  |  |  |  |
| 11 | Pc.c001203861.Contig1 | 2415 | + | 1415563 | 1418277 |  |  |  |  |  |  |  |  |  |  |  |  |  |  |
| 11 | Pb.c000401466.Contig1 | 8150 | + | 1418658 | 1426787 |  |  |  |  |  |  |  |  |  |  |  |  |  |  |
| 11 | Pc.c001103718.Contig1 | 1901 | - | 1421424 | 1423304 |  |  |  |  |  |  |  |  |  |  |  |  |  |  |
| 11 | PcContig376 | 6866 | - | 1423193 | 1430564 |  |  |  |  |  |  |  |  |  |  |  |  |  |  |
| JC11 | MALPY03031 | 803 | + | 1426349 | 1427152 |  |  |  |  |  |  | 5 |  |  |  |  |  |  |  |
| 11 | Pb.c000601501.Contig1 | 3667 | + | 1426921 | 1430853 |  |  |  |  |  |  |  |  |  |  |  |  |  |  |
| 11 | MALPY01728 | 4226 | - | 1427452 | 1432458 |  |  |  |  |  |  |  |  |  |  |  |  |  |  |
| 11 | MALPY00199 | 4880 | - | 1432169 | 1437107 |  |  |  |  |  |  | 88 | 200 |  |  |  |  |  |  |
| 11 | PcContig561 | 3377 | - | 1432372 | 1435782 |  |  |  |  |  |  |  |  |  |  |  |  |  |  |
| 11 | Pb.c000301476.Contig1 | 4190 | + | 1432788 | 1436981 |  |  |  |  |  |  |  |  |  |  |  |  |  |  |
| 11 | Pc.c000503099.Contig1 | 9776 | - | 1436992 | 1448408 |  |  |  |  |  |  |  |  |  |  |  |  |  |  |
| 11 | Pb.c000301372.Contig1 | 11870 | + | 1437033 | 1450911 |  |  |  |  |  |  |  |  |  |  |  |  |  |  |
| 11 | MALPY03037 | 713 | + | 1437155 | 1437877 |  |  |  |  |  |  | 88 | 1200 |  |  |  |  |  |  |
| 11 | MALPY00148 | 11338 | - | 1439464 | 1451280 |  |  |  |  |  | 356 | 88 |  |  |  |  |  |  |  |
| 11 | PcContig516 | 1019 | - | 1448649 | 1449770 |  |  |  |  |  |  |  |  |  |  |  |  |  |  |
| 11 | PcContig127 | 2913 | - | 1449957 | 1453027 |  |  |  |  |  |  |  |  |  |  |  |  |  |  |
| 11 | Pb.c001904920.Contig1 | 1218 | + | 1451211 | 1452405 |  |  |  |  |  |  |  |  |  |  |  |  |  |  |
| 11 | MALPY01481 | 8945 | + | 1451648 | 1460590 | cRMPc09082 | 48080 | 538 | 1412510 | 1460590 | 356 | 172 | 4100 |  |  |  |  |  |  |
| TK11 | MALPY01357 | 3071 | - | 1461128 | 1463950 |  |  |  |  |  | 356 |  |  |  |  |  |  |  |  |
| 11 | Pb.c000401513.Contig1 | 7202 | - | 1462737 | 1469954 |  |  |  |  |  |  |  |  |  |  |  |  |  |  |
| 11 | Pc.c001103046.Contig2 | 5437 | + | 1464377 | 1469814 |  |  |  |  |  |  |  |  |  |  |  |  |  |  |
| 11 | MALPY01065 | 4760 | + | 1465039 | 1469811 | cRMPc09083 | 8826 | 2485 | 1461128 | 1469954 |  | 172 | 1700 |  |  |  |  |  |  |
| 11 | MALPY01773 | 6602 | - | 1472439 | 1479381 |  |  |  |  |  |  | 172 |  |  |  |  |  |  |  |
| 11 | Pb.c001301889.Contig1 | 2860 | + | 1473776 | 1476966 |  |  |  |  |  |  |  |  |  |  |  |  |  |  |
| 11 | PcContig422 | 2505 | + | 1474189 | 1476694 |  |  |  |  |  |  |  |  |  |  |  |  |  |  |
| 11 | Pc.c001204061.Contig1 | 5563 | + | 1477273 | 1483060 |  |  |  |  |  |  |  |  |  |  |  |  |  |  |
| 11 | Pb.c001602469.Contig1 | 1998 | - | 1477484 | 1479575 |  |  |  |  |  |  |  |  |  |  |  |  |  |  |
| 11 | Pb.c000301437.Contig1 | 2286 | - | 1479684 | 1481958 | cRMPc09084 | 10621 | 8613 | 1472439 | 1483060 |  |  |  | cRMPs0922 | 240863 | 8613 | 1242197 | 1483060 |  |
| 11 | Pb.c000301436.Contig1 | 7190 | + | 1491673 | 1498958 |  |  |  |  |  |  |  |  |  |  |  |  |  |  |
| 11 | PcContig58 | 5635 | - | 1493686 | 1499511 |  |  |  |  |  |  |  |  |  |  |  |  |  |  |
| 11 | MALPY03042 | 849 | - | 1495071 | 1495920 |  |  |  |  |  |  | 96 | 800 |  |  |  |  |  |  |
| 11 | MALPY02097 | 3995 | + | 1496163 | 1500148 | cRMPc09085 | 8475 | 4500 | 1491673 | 1500148 |  | 96 - NS | 4500 |  |  |  |  |  |  |
| 11 | MALPY00254 | 2945 | + | 1504648 | 1507567 | cRMPc09086 | 2919 | 332 | 1504648 | 1507567 |  | 96 - NS | 1800 |  |  |  |  |  |  |
| 11 | Pc.c001204021.Contig1 | 1389 | + | 1507899 | 1509288 |  |  |  |  |  |  |  |  |  |  |  |  |  |  |
| 11 | MALPY00982 | 5770 | - | 1509141 | 1514401 |  |  |  |  |  |  | 96 | 800 |  |  |  |  |  |  |
| 11 | Pc.c000101999.Contig1 | 3263 | + | 1510019 | 1513318 |  |  |  |  |  |  |  |  |  |  |  |  |  |  |
| 11 | Pb.c001402179.Contig1 | 1952 | - | 1510521 | 1512340 |  |  |  |  |  |  |  |  |  |  |  |  |  |  |
| 11 | Pb.c001402178.Contig1 | 718 | - | 1512423 | 1513132 |  |  |  |  |  |  |  |  |  |  |  |  |  |  |
| 11 | Pb.c001101900.Contig1 | 2760 | + | 1513183 | 1515991 |  |  |  |  |  |  |  |  |  |  |  |  |  |  |
| 11 | MALPY01028 | 10099 | + | 1513791 | 1524565 |  |  |  |  |  | 940 | 96 |  |  |  |  |  |  |  |
| 11 | Pc.c000903507.Contig1 | 1582 | + | 1516796 | 1518354 |  |  |  |  |  |  |  |  |  |  |  |  |  |  |
| 11 | Pb.c001101543.Contig1 | 3763 | + | 1522598 | 1526656 |  |  |  |  |  |  |  |  |  |  |  |  |  |  |
| 11 | Pc.c001505622.Contig1 | 589 | + | 1523745 | 1524334 |  |  |  |  |  |  |  |  |  |  |  |  |  |  |
| 11 | MALPY00767 | 4713 | + | 1524573 | 1529400 |  |  |  |  |  | 940 | 101 | 2000 |  |  |  |  |  |  |
| 11 | Pc.c000603029.Contig1 | 3299 | + | 1525113 | 1528412 | cRMPc09087 | 21501 | 730 | 1507899 | 1529400 |  |  |  |  |  |  |  |  |  |
| 11 | MALPY01761 | 10940 | + | 1530130 | 1542143 |  |  |  |  |  |  | 101 | 4000 |  |  |  |  |  |  |
| 11 | Pc.c000002575.Contig1 | 2943 | - | 1531133 | 1534076 |  |  |  |  |  |  |  |  |  |  |  |  |  |  |
| 11 | Pb.c000101352.Contig1 | 2220 | + | 1532152 | 1534371 |  |  |  |  |  |  |  |  |  |  |  |  |  |  |
| 11 | Pb.c000101559.Contig1 | 2850 | + | 1534635 | 1537485 |  |  |  |  |  |  |  |  |  |  |  |  |  |  |
| 11 | Pb.c001904335.Contig1 | 1444 | + | 1538087 | 1539567 |  |  |  |  |  |  |  |  |  |  |  |  |  |  |
| 11 | Pb.c002007516.Contig1 | 2841 | + | 1540572 | 1543365 | cRMPc09088 | 13235 | 950 | 1530130 | 1543365 |  |  |  |  |  |  |  |  |  |
| 11 | PbContig242 | 10666 | + | 1544315 | 1555601 |  |  |  |  |  |  |  |  |  |  |  |  |  |  |
| 11 | MALPY01863 | 2725 | - | 1545671 | 1548283 |  |  |  |  |  |  | 101 |  |  |  |  |  |  |  |
| 11 | Pc.c001505048.Contig1 | 1373 | + | 1545878 | 1547145 |  |  |  |  |  |  |  |  |  |  |  |  |  |  |
| 11 | Pc.c000602350.Contig1 | 5279 | + | 1548101 | 1553374 |  |  |  |  |  |  |  |  |  |  |  |  |  |  |
| 11 | MALPY03229 | 810 | - | 1548941 | 1549757 |  |  |  |  |  |  | 170 | 1000 |  |  |  |  |  |  |
| 11 | MALPY01228 | 5419 | + | 1550322 | 1555638 | cRMPc09089 | 11323 | 1380 | 1544315 | 1555638 | 481 | 170 |  |  |  |  |  |  |  |
| 11 | MALPY01227 | 2764 | + | 1557018 | 1559781 |  |  |  |  |  | 481 |  |  |  |  |  |  |  |  |
| 11 | Pb.c001502005.Contig1 | 3860 | - | 1557213 | 1561146 |  |  |  |  |  |  |  |  |  |  |  |  |  |  |
| 11 | Pc.c001708959.Contig1 | 981 | - | 1559108 | 1560173 | cRMPc09090 | 4128 | 5443 | 1557018 | 1561146 |  |  |  | cRMPs0923 | 69473 | 5443 | 1491673 | 1561146 |  |
| 11 | MALPY00542 | 11949 | + | 1566589 | 1579498 |  |  |  |  |  | 162 | 9 | 4100 |  |  |  |  |  |  |
| 11 | PcContig572 | 1926 | - | 1568611 | 1570537 |  |  |  |  |  |  |  |  |  |  |  |  |  |  |
| 11 | Pb.c001301851.Contig1 | 4114 | - | 1568933 | 1573206 |  |  |  |  |  |  |  |  |  |  |  |  |  |  |
| 11 | PcContig453 | 2295 | - | 1570487 | 1572785 |  |  |  |  |  |  |  |  |  |  |  |  |  |  |
| 11 | Pc.c000703911.Contig1 | 5410 | - | 1573447 | 1579327 |  |  |  |  |  |  |  |  |  |  |  |  |  |  |
| 11 | Pb.c001502548.Contig1 | 1565 | - | 1573861 | 1575601 |  |  |  |  |  |  |  |  |  |  |  |  |  |  |
| man11 | PbContig6164 | 2330 | + | 1579319 | 1584145 |  |  |  |  |  | 162 |  |  |  |  |  |  |  | MALPY00542/MALPY00719 |
| man11 | MALPY00719 | 3937 | - | 1582089 | 1586026 |  |  |  |  |  | 162 |  |  |  |  |  |  |  | PbContig6164/MALPY00718 |
| 11 | Pb.c001101908.Contig1 | 3553 | - | 1584436 | 1587974 |  |  |  |  |  |  |  |  |  |  |  |  |  |  |
| 11 | MALPY00718 | 7385 | - | 1585963 | 1593969 |  |  |  |  |  | 162 | 9 |  |  |  |  |  |  |  |
| 11 | PbContig15 | 7148 | - | 1587982 | 1596450 |  |  |  |  |  |  |  |  |  |  |  |  |  |  |
| 11 | PcContig730 | 1826 | - | 1588136 | 1589965 |  |  |  |  |  |  |  |  |  |  |  |  |  |  |
| 11 | PcContig300 | 6258 | - | 1590275 | 1597689 |  |  |  |  |  |  |  |  |  |  |  |  |  |  |
| 11 | MALPY02454 | 4064 | + | 1594352 | 1598419 |  |  |  |  |  |  | 193 | 800 |  |  |  |  |  |  |
| 11 | Pb.c001803389.Contig1 | 1741 | + | 1597464 | 1599453 |  |  |  |  |  |  |  |  |  |  |  |  |  |  |
| 11 | Pc.c000903430.Contig1 | 1766 | - | 1598139 | 1599905 | cRMPc09091 | 33316 | 775 | 1566589 | 1599905 |  |  |  |  |  |  |  |  |  |
| 11 | MALPY00083 | 8225 | - | 1600680 | 1608794 |  |  |  |  |  | 55 | 193 |  |  |  |  |  |  |  |
| 11 | Pb.c000201531.Contig1 | 2710 | - | 1605909 | 1608544 |  |  |  |  |  |  |  |  |  |  |  |  |  |  |
| 11 | PcContig343 | 2173 | - | 1606731 | 1608840 | cRMPc09092 | 8160 | 50 | 1600680 | 1608840 |  |  |  |  |  |  |  |  |  |
| 11 | MALPY01001 | 3050 | + | 1608890 | 1612058 |  |  |  |  |  | 55 |  |  |  |  |  |  |  |  |
| 11 | Pb.c002113501.Contig1 | 791 | - | 1609186 | 1609995 | cRMPc09093 | 3168 | 448 | 1608890 | 1612058 |  |  |  |  |  |  |  |  |  |
| 11 | MALPY00195 | 26275 | - | 1612506 | 1642008 |  |  |  |  |  | 55 |  |  |  |  |  |  |  |  |
| 11 | PcContig236 | 3694 | - | 1613807 | 1617739 |  |  |  |  |  |  |  |  |  |  |  |  |  |  |
| 11 | PcContig589 | 5851 | - | 1618309 | 1624181 |  |  |  |  |  |  |  |  |  |  |  |  |  |  |
| 11 | MALPY03546 | 849 | + | 1624483 | 1625332 |  |  |  |  |  |  |  |  |  |  |  |  |  |  |
| 11 | Pb.c001803883.Contig1 | 1646 | + | 1624546 | 1626192 |  |  |  |  |  |  |  |  |  |  |  |  |  |  |
| 11 | Pc.c001204407.Contig1 | 904 | - | 1624566 | 1625470 |  |  |  |  |  |  |  |  |  |  |  |  |  |  |
| 11 | Pc.c000803276.Contig1 | 4560 | - | 1629395 | 1633916 |  |  |  |  |  |  |  |  |  |  |  |  |  |  |
| 11 | Pb.c001402137.Contig1 | 3744 | + | 1630858 | 1634611 |  |  |  |  |  |  |  |  |  |  |  |  |  |  |
| 11 | Pb.c000401203.Contig1 | 7263 | + | 1635104 | 1642394 |  |  |  |  |  |  |  |  |  |  |  |  |  |  |
| 11 | PcContig779 | 7519 | - | 1637722 | 1645270 |  |  |  |  |  |  |  |  |  |  |  |  |  |  |
| 11 | MALPY02857 | 2939 | + | 1640813 | 1643752 | cRMPc09094 | 32764 | 31 | 1612506 | 1645270 | 55 |  |  |  |  |  |  |  |  |
| 11 | MALPY02230 | 4756 | - | 1645301 | 1649926 |  |  |  |  |  | 55 |  |  |  |  |  |  |  |  |
| 11 | Pb.c001904443.Contig1 | 2406 | - | 1646547 | 1648971 |  |  |  |  |  |  |  |  |  |  |  |  |  |  |
| 11 | Pc.c001505144.Contig1 | 2926 | - | 1647662 | 1650627 |  |  |  |  |  |  |  |  |  |  |  |  |  |  |
| 11 | MALPY02631 | 2483 | - | 1648668 | 1651151 |  |  |  |  |  | 55 | 234 | 2500 |  |  |  |  |  |  |
| 11 | Pb.c001602537.Contig1 | 1758 | - | 1649057 | 1650812 | cRMPc09095 | 5850 | 260 | 1645301 | 1651151 |  |  |  |  |  |  |  |  |  |
| 11 | MALPY00714 | 13200 | + | 1651411 | 1663985 |  |  |  |  |  | 756 | 234 |  |  |  |  |  |  |  |
| 11 | Pb.c001904492.Contig1 | 1992 | + | 1652775 | 1654770 |  |  |  |  |  |  |  |  |  |  |  |  |  |  |
| 11 | Pc.c000803399.Contig1 | 1139 | - | 1652869 | 1654008 |  |  |  |  |  |  |  |  |  |  |  |  |  |  |
| 11 | Pb.c002113027.Contig1 | 626 | + | 1655771 | 1656397 |  |  |  |  |  |  |  |  |  |  |  |  |  |  |
| 11 | Pc.c000202696.Contig1 | 2924 | - | 1657477 | 1660389 |  |  |  |  |  |  |  |  |  |  |  |  |  |  |
| 11 | Pb.c001502520.Contig1 | 3674 | + | 1658682 | 1662287 |  |  |  |  |  |  |  |  |  |  |  |  |  |  |
| 11 | Pc.c001708671.Contig1 | 754 | - | 1660494 | 1661251 | cRMPc09096 | 12574 | 582 | 1651411 | 1663985 |  |  |  |  |  |  |  |  |  |
| TK11 | MALPY01016 | 2916 | + | 1664567 | 1665922 | cRMPc09097 | 1355 | 1230 | 1664567 | 1665922 | 756 |  |  | cRMPs0924 | 99333 | 1230 | 1566589 | 1665922 |  |
| man11 | MALPY02860 | 2362 | + | 1667152 | 1669552 | cRMPc09098 | 2400 | 2822 | 1667152 | 1669552 |  |  |  | cRMPs0925 | 2400 | 2822 | 1667152 | 1669552 |  |
| 11 | MALPY01664 | 7839 | - | 1672374 | 1680569 |  |  |  |  |  | 4 |  |  |  |  |  |  |  |  |
| 11 | Pc.c001104083.Contig1 | 2390 | + | 1674336 | 1676679 |  |  |  |  |  |  |  |  |  |  |  |  |  |  |
| 11 | Pb.c002006906.Contig1 | 1984 | - | 1674576 | 1676623 |  |  |  |  |  |  |  |  |  |  |  |  |  |  |
| JC11 | MALPY03025 | 469 | + | 1676409 | 1676887 |  |  |  |  |  |  |  |  |  |  |  |  |  |  |
| 11 | Pb.c001904377.Contig1 | 4815 | + | 1677339 | 1682180 |  |  |  |  |  |  |  |  |  |  |  |  |  |  |
| 11 | PcContig654 | 2320 | - | 1678002 | 1680316 |  |  |  |  |  |  |  |  |  |  |  |  |  |  |
| 11 | MALPY00868 | 11426 | - | 1680322 | 1693294 |  |  |  |  |  | 4 | 145 | 2100 |  |  |  |  |  |  |
| 11 | Pb.c001702766.Contig1 | 3180 | + | 1683083 | 1686263 |  |  |  |  |  |  |  |  |  |  |  |  |  |  |
| 11 | PcContig457 | 1269 | - | 1689324 | 1690593 |  |  |  |  |  |  |  |  |  |  |  |  |  |  |
| 11 | PbContig390 | 4058 | + | 1689866 | 1693943 | cRMPc09099 | 21569 | 1788 | 1672374 | 1693943 |  |  |  |  |  |  |  |  |  |
| 11 | MALPY00932 | 9721 | - | 1695731 | 1705382 |  |  |  |  |  | 46B | 145 |  |  |  |  |  |  |  |
| 11 | Pb.c001702888.Contig1 | 1468 | + | 1696836 | 1698298 |  |  |  |  |  |  |  |  |  |  |  |  |  |  |
| 11 | Pb.c001301869.Contig1 | 2439 | + | 1703530 | 1705972 |  |  |  |  |  |  |  |  |  |  |  |  |  |  |
| 11 | MALPY01173 | 4359 | + | 1703837 | 1708199 |  |  |  |  |  | 46B |  |  |  |  |  |  |  |  |
| 11 | Pc.c000903245.Contig1 | 2636 | + | 1704280 | 1706919 | cRMPc09100 | 12468 | 2006 | 1695731 | 1708199 |  |  |  |  |  |  |  |  |  |
| 11 | MALPY00664 | 11591 | + | 1710205 | 1722004 |  |  |  |  |  | 46B |  |  |  |  |  |  |  |  |
| 11 | Pb.c001101889.Contig1 | 5378 | - | 1711637 | 1716973 |  |  |  |  |  |  |  |  |  |  |  |  |  |  |
| 11 | Pc.c000403369.Contig1 | 1513 | - | 1715482 | 1716995 |  |  |  |  |  |  |  |  |  |  |  |  |  |  |
| 11 | Pc.c001204025.Contig1 | 1205 | - | 1717381 | 1718607 |  |  |  |  |  |  |  |  |  |  |  |  |  |  |
| 11 | Pc.c000903884.Contig1 | 958 | - | 1719134 | 1720092 | cRMPc09101 | 11799 | 1155 | 1710205 | 1722004 |  |  |  |  |  |  |  |  |  |
| 11 | Pb.c000701593.Contig1 | 2304 | + | 1723159 | 1725568 |  |  |  |  |  |  |  |  |  |  |  |  |  |  |
| 11 | MALPY00663 | 10939 | - | 1723334 | 1734075 |  |  |  |  |  | 46B |  |  |  |  |  |  |  |  |
| 11 | Pc.c001813365.Contig1 | 583 | + | 1724407 | 1724984 |  |  |  |  |  |  |  |  |  |  |  |  |  |  |
| 11 | PbContig150 | 24068 | + | 1726199 | 1752069 |  |  |  |  |  |  |  |  |  |  |  |  |  |  |
| 11 | Pc.c001505122.Contig1 | 3338 | + | 1726573 | 1730122 |  |  |  |  |  |  |  |  |  |  |  |  |  |  |
| 11 | Pc.c001708763.Contig1 | 608 | + | 1726905 | 1727513 |  |  |  |  |  |  |  |  |  |  |  |  |  |  |
| 11 | MALPY01975 | 14166 | - | 1734357 | 1750185 |  |  |  |  |  | 46B |  |  |  |  |  |  |  |  |
| 11 | Pc.c000803792.Contig1 | 1024 | - | 1734987 | 1736001 |  |  |  |  |  |  |  |  |  |  |  |  |  |  |
| 11 | PcContig474 | 997 | - | 1735973 | 1736970 |  |  |  |  |  |  |  |  |  |  |  |  |  |  |
| 11 | Pc.c000703973.Contig1 | 1522 | - | 1737743 | 1739256 |  |  |  |  |  |  |  |  |  |  |  |  |  |  |
| 11 | Pc.c000703298.Contig1 | 1678 | - | 1739457 | 1741170 |  |  |  |  |  |  |  |  |  |  |  |  |  |  |
| 11 | Pc.c001303495.Contig1 | 2209 | - | 1742161 | 1744370 |  |  |  |  |  |  |  |  |  |  |  |  |  |  |
| 11 | Pc.c001505025.Contig1 | 1144 | + | 1744888 | 1746039 |  |  |  |  |  |  |  |  |  |  |  |  |  |  |
| 11 | PcContig382 | 2621 | + | 1747040 | 1749661 |  |  |  |  |  |  |  |  |  |  |  |  |  |  |
| 11 | Pc.c001505400.Contig1 | 737 | + | 1749688 | 1750437 |  |  |  |  |  |  |  |  |  |  |  |  |  |  |
| 11 | MALPY01537 | 15956 | + | 1750092 | 1766069 | cRMPc09102 | 42910 | 500 | 1723159 | 1766069 | 46B |  |  |  |  |  |  |  |  |
| JC3/11 | MALPY02465 | 3479 | xxx | 1766569 | 1770048 | cRMPc09103 | 3479 | 500 | 1766569 | 1770048 | 46A/B |  |  |  |  |  |  |  |  |
| JC3/11 | MALPY02599 | 3919 | xxx | 1770548 | 1774467 | cRMPc09104 | 3919 |  | 1770548 | 1774467 | 46A/B |  |  | cRMPs0926 | 102093 |  | 1672374 | 1774467 |  |
| Telomere | |  |  |  |  |  |  |  |  |  |  |  |  |  |  |  |  |  |  |
|  |  | Pb Pc Py contig |  |  |  |  | cRMP contig | Gap to next cRMP contig |  |  |  |  |  |  | cRMP scaffold | Gap to next cRMP scaffold |  |  |  |
|  | Number | 740 |  |  |  | Number | 104 | 103 |  |  |  |  |  | Number | 26 | 25 |  |  |  |
|  | Average size (bp) | 4239 |  |  |  | Average size (bp) | 15451 | 1627 |  |  |  |  |  | Average size (bp) | 66291 | 2036 |  |  |  |
|  | Minimum size (bp) | 126 |  |  |  | Minimum size (bp) | 219 | 0 |  |  |  |  |  | Minimum size (bp) | 939 | 0 |  |  |  |
|  | Maximum size (bp) | 30629 |  |  |  | Maximum size (bp) | 113319 | 8613 |  |  |  |  |  | Maximum size (bp) | 240863 | 8613 |  |  |  |
|  | Total size (bp) | 3137098 |  |  |  | Total size (bp) | 1606937 | 167530 |  |  |  |  |  | Total size (bp) | 1723563 | 50904 |  |  |  |
|  |  |  |  |  |  |  |  |  |  |  |  |  |  |  |  |  |  |  |  |
