# Supplementary material for: A Plasmodium Whole-Genome Synteny Map: Indels and Synteny Breakpoints as Foci for Species-Specific Genes
Source: PLoS Pathog. 2005 Dec 23;1(4):e44. doi: 10.1371/journal.ppat.0010044 (PMC1317653; doi:10.1371/journal.ppat.0010044)
Supplement: Supplementary file 10 [file ppat.0010044.st026.doc]

Table S26 - RMPchr10


# Table S26 - RMPchr10

|  |  |  |  |  |  |  |  |  |  |  |  |  |  |  |  |  |  |  |  |  |
| --- | --- | --- | --- | --- | --- | --- | --- | --- | --- | --- | --- | --- | --- | --- | --- | --- | --- | --- | --- | --- |
| Table S26: Tiling path of RMP contigs combined to form cRMP chromosome 10. | | | | | | |  |  |  |  |  |  |  |  |  |  |  |  |  |  |
|  |  |  |  |  |  |  |  |  |  |  |  |  |  |  |  |  |  |  |  |  |
| Tiling paths of P. berghei (Pb), P. c. chabaudi (Pc) and P. y. yoelii (Py) contigs combined into composite rodent malaria parasite (cRMP) | | | | | | | | | |  |  |  |  |  |  |  |  |  |  |  |
| contigs aligned against parts of the 14 chromosomes of P. falciparum (Pf). The cRMP contigs are further linked by physical linkage | | | | | | | | | |  |  |  |  |  |  |  |  |  |  |  |
| and PCR (see Methods) to form cRMP scaffolds. Different synteny blocks with homology to different P. falciparum chromosomes | | | | | | | | | |  |  |  |  |  |  |  |  |  |  |  |
| together form the cRMP chromosomes. The tables also show gaps in the alignment (black lines), cRMP telomeres (white bars) and | | | | | | | | | |  |  |  |  |  |  |  |  |  |  |  |
| predicted centromeres (plum/black bars). Contigs, physical groups or PCR groups interrupted by inter- or intrasyntenic regions are | | | | | | | | | |  |  |  |  |  |  |  |  |  |  |  |
| printed in bold. A summary of the number of RMP contigs, cRMP contigs and cRMP scaffolds as well as their average, minimum, | | | | | | | | | |  |  |  |  |  |  |  |  |  |  |  |
| maximum and total sizes is provided. Column headings are as follows: | | | | |  |  |  |  |  |  |  |  |  |  |  |  |  |  |  |  |
|  |  |  |  |  |  |  |  |  |  |  |  |  |  |  |  |  |  |  |  |  |
| Pf chr.: P. falciparum chromosome to which the RMP contigs are aligned | | | | | |  |  |  |  |  |  |  |  |  |  |  |  |  |  |  |
| (#: PROmer analysis� from this study; JC# and TK#: Carlton et al.; man#: manual BLAST analysis; | | | | | | |  |  |  |  |  |  |  |  |  |  |  |  |  |  |
| i: inversed orientation of the synteny block; colours refer to those used in Fig. 2 of the paper); | | | | | | |  |  |  |  |  |  |  |  |  |  |  |  |  |  |
| Pb Pc Py contig: names of the aligned RMP contigs; | | |  |  |  |  |  |  |  |  |  |  |  |  |  |  |  |  |  |  |
| Size: size (bp) of the aligned RMP contigs; | |  |  |  |  |  |  |  |  |  |  |  |  |  |  |  |  |  |  |  |
| +/-: orientation of the aligned RMP contigs; | |  |  |  |  |  |  |  |  |  |  |  |  |  |  |  |  |  |  |  |
| Start contig & End contig: estimated start & end coordinates of alignment of the RMP contigs based on the coordinates | | | | | | | | |  |  |  |  |  |  |  |  |  |  |  |  |
| of PROmer hits corrected for the contig�s size and orientation and presence of inter- and intrasyntenic regions | | | | | | | |  |  |  |  |  |  |  |  |  |  |  |  |  |
| (the start position of the first RMP contig in the alignment was set at zero); | | | | | |  |  |  |  |  |  |  |  |  |  |  |  |  |  |  |
| cRMP contig: names of cRMP contigs | |  |  |  |  |  |  |  |  |  |  |  |  |  |  |  |  |  |  |  |
| (the first 2 digits refer to the cRMP chromosome number and the following 3 digits designate the order of the contigs); | | | | | | | | |  |  |  |  |  |  |  |  |  |  |  |  |
| cRMP contig size: size of cRMP contigs based on the corrected coordinates of alignment; | | | | | | |  |  |  |  |  |  |  |  |  |  |  |  |  |  |
| Gap size to next cRMP contig: size of gaps between neighbouring cRMP contigs; | | | | | | |  |  |  |  |  |  |  |  |  |  |  |  |  |  |
| Start cRMP contig & End cRMP contig: estimated start & end coordinates of alignment of the cRMP contigs; | | | | | | | |  |  |  |  |  |  |  |  |  |  |  |  |  |
| Phys. group (Py): physical linkage of neighbouring P. y. yoelii contigs; | | | | | |  |  |  |  |  |  |  |  |  |  |  |  |  |  |  |
| PCR group (Py): neighbouring P. y. yoelii contigs were linked previously or for this study by PCR; | | | | | | |  |  |  |  |  |  |  |  |  |  |  |  |  |  |
| PCR size (Py): size of the PCR product; | |  |  |  |  |  |  |  |  |  |  |  |  |  |  |  |  |  |  |  |
| cRMP scaffold: names of cRMP scaffolds | |  |  |  |  |  |  |  |  |  |  |  |  |  |  |  |  |  |  |  |
| (the first 2 digits refer to the cRMP chromosome number and the following 2 digits designate the order of the scaffolds); | | | | | | | | |  |  |  |  |  |  |  |  |  |  |  |  |
| cRMP scaffold size: size of cRMP scaffolds; | | |  |  |  |  |  |  |  |  |  |  |  |  |  |  |  |  |  |  |
| Gap size to next cRMP scaffold: size of gaps between neighbouring cRMP scaffolds; | | | | | | |  |  |  |  |  |  |  |  |  |  |  |  |  |  |
| Start cRMP scaffold & End cRMP scaffold: estimated start & end coordinates of alignment of the cRMP scaffolds; | | | | | | | | |  |  |  |  |  |  |  |  |  |  |  |  |
| Overlap: Linkage between RMP contigs established through manual BLAST analysis. | | | | | | |  |  |  |  |  |  |  |  |  |  |  |  |  |  |
|  |  |  |  |  |  |  |  |  |  |  |  |  |  |  |  |  |  |  |  |  |
| Pf chr. | Pb Pc Py contig | Size | +/- | Start contig | End contig | cRMP contig | cRMP contig size | Gap size to next cRMP contig | Start cRMP contig | End cRMP contig | Phys. group (Py) | PCR group (Py) | PCR size (Py) | cRMP scaffold | cRMP scaffold size | Gap size to next cRMP scaffold | Start cRMP scaffold | End cRMP scaffold | Overlap (manual) |  |
| Telomere | |  |  |  |  |  |  |  |  |  |  |  |  |  |  |  |  |  |  |  |
| man4 | Pb\_1024h07q1c | 13639 | + | 0 | 13639 |  |  |  |  |  |  |  |  |  |  |  |  |  | \| |  |
| 4 | PbContig198 | 11825 | + | 1793 | 13495 |  |  |  |  |  |  |  |  |  |  |  |  |  | \| |  |
| man4 | PbContig5734 | 10523 | + | 3118 | 13641 |  |  |  |  |  |  |  |  |  |  |  |  |  | MALPY00673 |  |
| 4 | MALPY00673 | 44972 | + | 4464 | 47793 |  |  |  |  |  |  | 308 | 2500 |  |  |  |  |  |  |  |
| 4 | Pc.c000403267.Contig1 | 2254 | + | 7076 | 9076 |  |  |  |  |  |  |  |  |  |  |  |  |  |  |  |
| 4 | Pc.c001104353.Contig1 | 1315 | + | 9543 | 10855 |  |  |  |  |  |  |  |  |  |  |  |  |  |  |  |
| 4 | PbContig392 | 14869 | - | 14949 | 30584 |  |  |  |  |  |  |  |  |  |  |  |  |  |  |  |
| 4 | Pc.c000903252.Contig1 | 4647 | + | 15872 | 20546 |  |  |  |  |  |  |  |  |  |  |  |  |  |  |  |
| 4 | PbContig229 | 3777 | - | 30613 | 34384 |  |  |  |  |  |  |  |  |  |  |  |  |  |  |  |
| 4 | Pb.c002006825.Contig1 | 9062 | - | 36245 | 45462 |  |  |  |  |  |  |  |  |  |  |  |  |  |  |  |
| 4 | Pc.c000302367.Contig1 | 7108 | - | 37365 | 44632 |  |  |  |  |  |  |  |  |  |  |  |  |  |  |  |
| 4 | Pb.c001202047.Contig1 | 1772 | + | 45363 | 47132 |  |  |  |  |  |  |  |  |  |  |  |  |  |  |  |
| 4 | Pc.c001003367.Contig1 | 2422 | - | 45733 | 48152 | cRMPc10001 | 48152 | 3029 | 0 | 48152 |  |  |  |  |  |  |  |  |  |  |
| 4 | MALPY00354 | 45695 | - | 51181 | 92152 |  |  |  |  |  | 43 | 308 |  |  |  |  |  |  |  |  |
| 4 | Pb.c000701633.Contig1 | 3477 | - | 52767 | 56253 |  |  |  |  |  |  |  |  |  |  |  |  |  |  |  |
| 4 | PcContig698 | 8226 | - | 52961 | 61196 |  |  |  |  |  |  |  |  |  |  |  |  |  |  |  |
| 4 | PbContig407 | 11515 | - | 55875 | 65276 |  |  |  |  |  |  |  |  |  |  |  |  |  |  |  |
| 4 | Pc.c000503111.Contig1 | 5227 | + | 60071 | 65026 |  |  |  |  |  |  |  |  |  |  |  |  |  |  |  |
| 4 | Pb.c000201534.Contig1 | 14842 | + | 64659 | 79047 |  |  |  |  |  |  |  |  |  |  |  |  |  |  |  |
| 4 | PbContig249 | 6389 | - | 83586 | 90437 |  |  |  |  |  |  |  |  |  |  |  |  |  |  |  |
| 4 | Pc.c001204611.Contig1 | 1903 | - | 86318 | 88213 |  |  |  |  |  |  |  |  |  |  |  |  |  |  |  |
| 4 | PbContig189 | 7171 | - | 90317 | 97891 |  |  |  |  |  |  |  |  |  |  |  |  |  |  |  |
| 4 | MALPY00281 | 9476 | - | 92374 | 102012 |  |  |  |  |  | 43 |  |  |  |  |  |  |  |  |  |
| 4 | PcContig169 | 2140 | + | 92522 | 94851 |  |  |  |  |  |  |  |  |  |  |  |  |  |  |  |
| 4 | Pc.c000603014.Contig1 | 3119 | + | 95109 | 98201 |  |  |  |  |  |  |  |  |  |  |  |  |  |  |  |
| 4 | Pb.c001201599.Contig1 | 7125 | - | 97019 | 104156 |  |  |  |  |  |  |  |  |  |  |  |  |  |  |  |
| 4 | MALPY01162 | 10465 | - | 100416 | 110894 |  |  |  |  |  | 43 | 240 | 700 |  |  |  |  |  |  |  |
| 4 | PcContig631 | 8155 | + | 102847 | 111046 |  |  |  |  |  |  |  |  |  |  |  |  |  |  |  |
| 4 | PbContig148 | 6082 | + | 104449 | 110630 |  |  |  |  |  |  |  |  |  |  |  |  |  |  |  |
| 4 | MALPY02493 | 2791 | - | 110301 | 113092 |  |  |  |  |  |  | 240 | 100 |  |  |  |  |  |  |  |
| 4 | Pb.c000501167.Contig3 | 7006 | + | 112316 | 119271 |  |  |  |  |  |  |  |  |  |  |  |  |  |  |  |
| 4 | MALPY01334 | 11971 | - | 112933 | 124883 |  |  |  |  |  | 68 | 240 | 4500 |  |  |  |  |  |  |  |
| 4 | Pc.c000303066.Contig1 | 4065 | - | 113237 | 117285 |  |  |  |  |  |  |  |  |  |  |  |  |  |  |  |
| 4 | Pb.c001001363.Contig2 | 3605 | - | 123629 | 127199 |  |  |  |  |  |  |  |  |  |  |  |  |  |  |  |
| man4 | MALPY01612 | 4598 | - | 124504 | 129102 |  |  |  |  |  | 68 |  |  |  |  |  |  |  | MALPY01334 |  |
| man4 | PbGSS AZ524097 | 623 | - | 128697 | 129320 |  |  |  |  |  |  |  |  |  |  |  |  |  | \| |  |
| 4 | MALPY00450 | 17745 | - | 128813 | 146379 |  |  |  |  |  | 47A | 240 |  |  |  |  |  |  | \| |  |
| man4 | PbContig5707 | 7395 | + | 129069 | 136464 |  |  |  |  |  |  |  |  |  |  |  |  |  | MALPY00450 |  |
| 4 | Pc.c001004003.Contig1 | 1271 | + | 129841 | 131076 |  |  |  |  |  |  |  |  |  |  |  |  |  |  |  |
| 4 | Pb.c001001363.Contig1 | 578 | - | 131565 | 132143 |  |  |  |  |  |  |  |  |  |  |  |  |  |  |  |
| 4 | MALPY03197 | 1633 | - | 131988 | 133602 |  |  |  |  |  |  |  |  |  |  |  |  |  |  |  |
| 4 | MALPY03547 | 686 | + | 132551 | 133237 |  |  |  |  |  |  |  |  |  |  |  |  |  |  |  |
| 4 | Pc.c000402902.Contig1 | 2133 | - | 132592 | 134725 |  |  |  |  |  |  |  |  |  |  |  |  |  |  |  |
| 4 | Pb.c001903730.Contig2 | 551 | + | 133001 | 133552 |  |  |  |  |  |  |  |  |  |  |  |  |  |  |  |
| 4 | Pb.c001903730.Contig3 | 2323 | + | 133630 | 136160 |  |  |  |  |  |  |  |  |  |  |  |  |  |  |  |
| 4 | MALPY03255 | 681 | - | 133705 | 134389 |  |  |  |  |  |  |  |  |  |  |  |  |  |  |  |
| 4 | Pc.c001708534.Contig1 | 1116 | + | 135136 | 136255 |  |  |  |  |  |  |  |  |  |  |  |  |  |  |  |
| 4 | MALPY03050 | 289 | - | 135531 | 135820 |  |  |  |  |  |  |  |  |  |  |  |  |  |  |  |
| 4 | PbContig238 | 10997 | - | 136361 | 147058 |  |  |  |  |  |  |  |  |  |  |  |  |  |  |  |
| 4 | MALPY03286 | 711 | + | 137181 | 137894 |  |  |  |  |  |  |  |  |  |  |  |  |  |  |  |
| 4 | Pc.c000502919.Contig1 | 2893 | - | 137875 | 140897 |  |  |  |  |  |  |  |  |  |  |  |  |  |  |  |
| 4 | MALPY00101 | 7707 | - | 145785 | 153741 |  |  |  |  |  | 47A | 176 | 100 |  |  |  |  |  |  |  |
| 4 | Pc.c001708108.Contig1 | 1758 | + | 146332 | 148297 |  |  |  |  |  |  |  |  |  |  |  |  |  |  |  |
| 4 | MALPY03571 | 1500 | - | 146484 | 147969 |  |  |  |  |  |  |  |  |  |  |  |  |  |  |  |
| 4 | Pb.c000901693.Contig1 | 769 | - | 146555 | 147309 |  |  |  |  |  |  |  |  |  |  |  |  |  |  |  |
| 4 | PbContig68 | 2173 | - | 147282 | 149425 |  |  |  |  |  |  |  |  |  |  |  |  |  |  |  |
| 4 | Pc.c000703244.Contig1 | 3271 | + | 148784 | 152139 |  |  |  |  |  |  |  |  |  |  |  |  |  |  |  |
| 4 | MALPY02285 | 2214 | - | 153409 | 155623 |  |  |  |  |  | 557 | 176 |  |  |  |  |  |  |  |  |
| 4 | PcContig575 | 2262 | - | 153713 | 155975 |  |  |  |  |  |  |  |  |  |  |  |  |  |  |  |
| 4 | PbContig288 | 11558 | + | 153809 | 165093 |  |  |  |  |  |  |  |  |  |  |  |  |  |  |  |
| TK4 | MALPY02638 | 3261 | - | 154732 | 157452 |  |  |  |  |  | 557 |  |  |  |  |  |  |  |  |  |
| 4 | MALPY01604 | 15600 | - | 158179 | 175898 |  |  |  |  |  | 396 |  |  |  |  |  |  |  |  |  |
| 4 | Pc.c001104222.Contig1 | 1967 | - | 158352 | 160293 |  |  |  |  |  |  |  |  |  |  |  |  |  |  |  |
| 4 | Pc.c000703467.Contig1 | 4452 | - | 160431 | 164706 |  |  |  |  |  |  |  |  |  |  |  |  |  |  |  |
| 4 | Pb.c000801391.Contig2 | 8087 | + | 167413 | 175506 |  |  |  |  |  |  |  |  |  |  |  |  |  |  |  |
| 4 | Pc.c000402215.Contig1 | 3877 | - | 167563 | 171440 |  |  |  |  |  |  |  |  |  |  |  |  |  |  |  |
| 4 | PcContig391 | 3006 | - | 171291 | 174335 | cRMPc10002 | 124717 | 7221 | 51181 | 175898 |  |  |  |  |  |  |  |  |  |  |
| 4 | MALPY02007 | 4246 | - | 183119 | 187786 |  |  |  |  |  | 396 |  |  |  |  |  |  |  |  |  |
| 4 | PbContig16 | 10066 | - | 185168 | 195255 |  |  |  |  |  |  |  |  |  |  |  |  |  |  |  |
| 4 | MALPY00854 | 13172 | - | 187751 | 200986 |  |  |  |  |  | 423 |  |  |  |  |  |  |  |  |  |
| 4 | Pc.c000402735.Contig1 | 2375 | - | 190486 | 192878 |  |  |  |  |  |  |  |  |  |  |  |  |  |  |  |
| 4 | Pb.c001803309.Contig1 | 7308 | - | 196894 | 204411 |  |  |  |  |  |  |  |  |  |  |  |  |  |  |  |
| 4 | MALPY01845 | 8226 | - | 198417 | 206542 |  |  |  |  |  | 423 | 190 | 2500 |  |  |  |  |  |  |  |
| 4 | Pc.c001505416.Contig1 | 1351 | - | 201590 | 203164 |  |  |  |  |  |  |  |  |  |  |  |  |  |  |  |
| 4 | Pb.c000401520.Contig1 | 2807 | - | 204681 | 207488 |  |  |  |  |  |  |  |  |  |  |  |  |  |  |  |
| TK4 | MALPY01815 | 2057 | - | 205964 | 207994 |  |  |  |  |  | 423 |  |  |  |  |  |  |  |  |  |
| 4 | Pb.c001502017.Contig1 | 4215 | + | 207830 | 212153 |  |  |  |  |  |  |  |  |  |  |  |  |  |  |  |
| 4 | MALPY02852 | 2028 | - | 208692 | 210729 |  |  |  |  |  |  | 190 | 300 |  |  |  |  |  |  |  |
| 4 | MALPY00977 | 10607 | - | 211039 | 222374 |  |  |  |  |  |  | 190 | 6000 |  |  |  |  |  |  |  |
| 4 | Pc.c000802689.Contig2 | 4867 | - | 212582 | 217449 |  |  |  |  |  |  |  |  |  |  |  |  |  |  |  |
| 4 | Pb.c001202125.Contig1 | 4316 | - | 213805 | 218121 |  |  |  |  |  |  |  |  |  |  |  |  |  |  |  |
| 4 | Pc.c000903346.Contig1 | 2763 | - | 217675 | 220420 |  |  |  |  |  |  |  |  |  |  |  |  |  |  |  |
| 4 | Pb.c001502096.Contig1 | 2219 | - | 219020 | 221287 | cRMPc10003 | 39255 | 6147 | 183119 | 222374 |  |  |  |  |  |  |  |  |  |  |
| 4 | MALPY00613 | 22676 | + | 228521 | 250827 |  |  |  |  |  |  | 190 | 800 |  |  |  |  |  |  |  |
| 4 | PbContig230 | 10640 | + | 229550 | 240191 |  |  |  |  |  |  |  |  |  |  |  |  |  |  |  |
| 4 | Pb.c001201653.Contig2 | 8744 | + | 242451 | 250806 |  |  |  |  |  |  |  |  |  |  |  |  |  |  |  |
| 4 | Pc.c001606009.Contig1 | 2081 | - | 247259 | 249351 |  |  |  |  |  |  |  |  |  |  |  |  |  |  |  |
| 4 | MALPY03207 | 376 | - | 248926 | 249302 | cRMPc10004 | 22306 | 415 | 228521 | 250827 |  |  |  |  |  |  |  |  |  |  |
| 4 | MALPY00127 | 17650 | - | 251242 | 268952 |  |  |  |  |  | 246 | 190 | 4000 |  |  |  |  |  |  |  |
| 4 | Pb.c001401732.Contig1 | 7624 | - | 251488 | 258912 |  |  |  |  |  |  |  |  |  |  |  |  |  |  |  |
| 4 | Pc.c000002519.Contig1 | 3935 | + | 251748 | 255662 |  |  |  |  |  |  |  |  |  |  |  |  |  |  |  |
| 4 | MALPY03089 | 1893 | - | 251947 | 253834 |  |  |  |  |  |  |  |  |  |  |  |  |  |  |  |
| 4 | MALPY03469 | 485 | + | 253018 | 253503 |  |  |  |  |  |  |  |  |  |  |  |  |  |  |  |
| 4 | PcContig620 | 7032 | - | 255235 | 262282 |  |  |  |  |  |  |  |  |  |  |  |  |  |  |  |
| 4 | PbContig95 | 9247 | + | 258983 | 268474 |  |  |  |  |  |  |  |  |  |  |  |  |  |  |  |
| 4 | Pc.c001605903.Contig1 | 1199 | - | 263657 | 265000 |  |  |  |  |  |  |  |  |  |  |  |  |  |  |  |
| 4 | PbContig313 | 28378 | - | 268694 | 299094 |  |  |  |  |  |  |  |  |  |  |  |  |  |  |  |
| TK4 | MALPY01375 | 3707 | + | 269915 | 272523 |  |  |  |  |  | 246 |  |  |  |  |  |  |  |  |  |
| 4 | MALPY01231 | 30629 | - | 273844 | 306672 |  |  |  |  |  |  | 190 | 1200 |  |  |  |  |  |  |  |
| 4 | Pc.c000403184.Contig1 | 1634 | - | 273983 | 275617 |  |  |  |  |  |  |  |  |  |  |  |  |  |  |  |
| 4 | Pc.c001708785.Contig1 | 832 | - | 275989 | 276827 |  |  |  |  |  |  |  |  |  |  |  |  |  |  |  |
| 4 | Pc.c001002689.Contig1 | 4237 | - | 281498 | 286329 |  |  |  |  |  |  |  |  |  |  |  |  |  |  |  |
| 4 | Pc.c001811686.Contig1 | 1397 | - | 286895 | 288292 |  |  |  |  |  |  |  |  |  |  |  |  |  |  |  |
| 4 | PcContig272 | 1358 | - | 290071 | 291420 |  |  |  |  |  |  |  |  |  |  |  |  |  |  |  |
| 4 | Pc.c001404818.Contig1 | 1961 | - | 291444 | 293405 |  |  |  |  |  |  |  |  |  |  |  |  |  |  |  |
| 4 | Pc.c001003414.Contig1 | 1976 | - | 293518 | 295486 |  |  |  |  |  |  |  |  |  |  |  |  |  |  |  |
| 4 | Pb.c001502734.Contig1 | 4122 | + | 299547 | 303961 |  |  |  |  |  |  |  |  |  |  |  |  |  |  |  |
| 4 | Pb.c001702812.Contig1 | 2740 | - | 305160 | 307900 |  |  |  |  |  |  |  |  |  |  |  |  |  |  |  |
| 4 | Pc.c000502928.Contig1 | 3440 | - | 305817 | 309192 |  |  |  |  |  |  |  |  |  |  |  |  |  |  |  |
| 4 | MALPY00633 - BP | 30803 | - | 308039 | 338323 |  |  |  |  |  |  | 190 |  |  |  |  |  |  |  |  |
| 4 | PbContig128 | 7857 | + | 308989 | 315258 |  |  |  |  |  |  |  |  |  |  |  |  |  |  |  |
| 4 | Pc.c000703963.Contig1 | 692 | - | 309209 | 309910 |  |  |  |  |  |  |  |  |  |  |  |  |  |  |  |
| 4 | Pc.c000703334.Contig1 | 1126 | + | 312934 | 314060 |  |  |  |  |  |  |  |  |  |  |  |  |  |  |  |
| 4 | Pc.c001304223.Contig1 | 3521 | - | 316199 | 320153 |  |  |  |  |  |  |  |  |  |  |  |  |  |  |  |
| 4 | PbContig126 - BP | 20297 | + | 316621 | 338337 |  |  |  |  |  |  |  |  |  |  |  |  |  |  |  |
| 4 | MALPY03394 | 653 | - | 317858 | 318502 |  |  |  |  |  |  |  |  |  |  |  |  |  |  |  |
| i14 | Pc.c000803423.Contig1 | 2089 | - | 324202 | 326457 |  |  |  |  |  |  |  |  |  |  |  |  |  |  |  |
| i14 | MALPY03185 | 630 | - | 326437 | 327070 |  |  |  |  |  |  | 421 |  |  |  |  |  |  |  |  |
| i14 | Pc.c001404695.Contig1 | 4525 | - | 326603 | 331118 |  |  |  |  |  |  |  |  |  |  |  |  |  |  |  |
| i14 | Pc.c001203223.Contig3 | 3960 | + | 335237 | 339197 | cRMPc10005 | 87955 | 2612 | 251242 | 339197 |  |  |  |  |  |  |  |  |  |  |
| i14 | PbContig231 | 16577 | - | 341809 | 359151 |  |  |  |  |  |  |  |  |  |  |  |  |  |  |  |
| i14 | MALPY00168 | 29373 | - | 342323 | 372903 |  |  |  |  |  |  | 190 |  |  |  |  |  |  |  |  |
| i14 | Pc.c000903431.Contig1 | 3501 | + | 354388 | 357892 |  |  |  |  |  |  |  |  |  |  |  |  |  |  |  |
| i14 | PbContig91 | 7515 | - | 366058 | 373564 | cRMPc10006 | 31755 | 337 | 341809 | 373564 |  |  |  | cRMPs1001 | 373564 | 337 | 0 | 373564 |  |  |
| i14 | Pb.c000901779.Contig1 | 3266 | - | 373901 | 377158 |  |  |  |  |  |  |  |  |  |  |  |  |  |  |  |
| i14 | MALPY01793 | 6909 | + | 374215 | 381275 |  |  |  |  |  |  | 272 | 9000 |  |  |  |  |  |  |  |
| i14 | Pc.c001505026.Contig1 | 3029 | - | 374923 | 378129 |  |  |  |  |  |  |  |  |  |  |  |  |  |  |  |
| i14 | Pb.c000901778.Contig1 | 3373 | - | 377295 | 380643 | cRMPc10007 | 7374 | 3399 | 373901 | 381275 |  |  |  |  |  |  |  |  |  |  |
| i14 | Pc.c000202668.Contig1 | 3767 | - | 384674 | 388455 |  |  |  |  |  |  |  |  |  |  |  |  |  |  |  |
| i14 | MALPY01095 | 12697 | - | 384819 | 397900 |  |  |  |  |  |  | 458 | 9500 |  |  |  |  |  |  |  |
| i14 | Pb.c000501119.Contig1 | 11892 | + | 386440 | 398744 |  |  |  |  |  |  |  |  |  |  |  |  |  |  |  |
| i14 | Pc.c000002665.Contig1 | 1568 | - | 388549 | 390063 |  |  |  |  |  |  |  |  |  |  |  |  |  |  |  |
| i14 | Pc.c001304339.Contig1 | 1667 | - | 390123 | 392135 |  |  |  |  |  |  |  |  |  |  |  |  |  |  |  |
| i14 | MALPY03406 | 834 | + | 390126 | 390960 |  |  |  |  |  |  | 272 |  |  |  |  |  |  |  |  |
| i14 | Pc.c001103822.Contig1 | 4143 | - | 392367 | 396706 |  |  |  |  |  |  |  |  |  |  |  |  |  |  |  |
| i14 | MALPY00702 | 15368 | + | 398304 | 413748 |  |  |  |  |  |  | 114 | 1400 |  |  |  |  |  |  |  |
| i14 | Pb.c000901423.Contig1 | 12681 | - | 401182 | 413888 |  |  |  |  |  |  |  |  |  |  |  |  |  |  |  |
| i14 | PcContig142 | 1798 | + | 401502 | 403294 |  |  |  |  |  |  |  |  |  |  |  |  |  |  |  |
| i14 | Pc.c001204048.Contig1 | 3009 | + | 404547 | 407559 |  |  |  |  |  |  |  |  |  |  |  |  |  |  |  |
| i14 | MALPY03555 | 670 | - | 406918 | 407588 |  |  |  |  |  |  | 458 | 1600 |  |  |  |  |  |  |  |
| i14 | MALPY03370 | 610 | + | 409236 | 409846 |  |  |  |  |  |  | 458 | 1400 |  |  |  |  |  |  |  |
| iJC14 | MALPY03347 | 1251 | + | 410173 | 411424 |  |  |  |  |  |  | 458 | 600 |  |  |  |  |  |  |  |
| i14 | Pc.c001003864.Contig1 | 856 | - | 410685 | 411541 |  |  |  |  |  |  |  |  |  |  |  |  |  |  |  |
| i14 | Pc.c001003865.Contig1 | 655 | - | 411648 | 412303 |  |  |  |  |  |  |  |  |  |  |  |  |  |  |  |
| i14 | MALPY03388 | 739 | + | 411786 | 412537 | cRMPc10008 | 29214 | 302 | 384674 | 413888 |  | 458 |  |  |  |  |  |  |  |  |
| i14 | MALPY00172 | 17437 | + | 414190 | 432272 |  |  |  |  |  |  | 114 | 1200 |  |  |  |  |  |  |  |
| i14 | PbContig202 | 2764 | - | 416533 | 419412 |  |  |  |  |  |  |  |  |  |  |  |  |  |  |  |
| i14 | PcContig146 | 2748 | - | 417439 | 420275 |  |  |  |  |  |  |  |  |  |  |  |  |  |  |  |
| i14 | Pb.c001501963.Contig1 | 2337 | - | 419533 | 421943 |  |  |  |  |  |  |  |  |  |  |  |  |  |  |  |
| i14 | Pb.c001202136.Contig1 | 2885 | - | 422351 | 425560 |  |  |  |  |  |  |  |  |  |  |  |  |  |  |  |
| i14 | Pc.c001304956.Contig1 | 638 | - | 422759 | 423397 | cRMPc10009 | 18082 | 2222 | 414190 | 432272 |  |  |  |  |  |  |  |  |  |  |
| i14 | Pb.c001904283.Contig1 | 3020 | + | 434494 | 437565 |  |  |  |  |  |  |  |  |  |  |  |  |  |  |  |
| i14 | MALPY00889 | 10990 | - | 434577 | 446015 |  |  |  |  |  |  | 114 | 2000 |  |  |  |  |  |  |  |
| i14 | Pc.c001707805.Contig1 | 1597 | - | 437029 | 438601 |  |  |  |  |  |  |  |  |  |  |  |  |  |  |  |
| i14 | Pb.c001001977.Contig1 | 884 | + | 438128 | 439018 |  |  |  |  |  |  |  |  |  |  |  |  |  |  |  |
| i14 | PcContig625 | 6817 | - | 438636 | 445787 |  |  |  |  |  |  |  |  |  |  |  |  |  |  |  |
| i14 | Pb.c000301365.Contig1 | 5423 | + | 439853 | 445597 | cRMPc10010 | 11521 | 437 | 434494 | 446015 |  |  |  |  |  |  |  |  |  |  |
| i14 | PcContig709 | 2699 | - | 446452 | 449360 |  |  |  |  |  |  |  |  |  |  |  |  |  |  |  |
| i14 | Pb.c001703057.Contig1 | 2917 | + | 446742 | 449889 |  |  |  |  |  |  |  |  |  |  |  |  |  |  |  |
| i14 | MALPY02431 | 5768 | + | 447279 | 453051 |  |  |  |  |  | 581 | 114 | 6500 |  |  |  |  |  |  |  |
| i14 | Pb.c000201578.Contig1 | 3972 | + | 450058 | 454030 |  |  |  |  |  |  |  |  |  |  |  |  |  |  |  |
| i14 | Pc.c001606267.Contig1 | 1161 | - | 451059 | 452220 |  |  |  |  |  |  |  |  |  |  |  |  |  |  |  |
| iman14 | MALPY02797 | 5698 | - | 451603 | 457519 | cRMPc10011 | 11067 | 207 | 446452 | 457519 | 581 |  |  |  |  |  |  |  |  |  |
| i14 | PcContig103 | 4969 | + | 457726 | 462948 |  |  |  |  |  |  |  |  |  |  |  |  |  |  |  |
| i14 | MALPY03083 | 1940 | + | 459889 | 461829 |  |  |  |  |  |  | 114 | 700 |  |  |  |  |  |  |  |
| i14 | MALPY03039 | 805 | + | 462137 | 462942 | cRMPc10012 | 5222 | 2439 | 457726 | 462948 |  | 114 | 4000 |  |  |  |  |  |  |  |
| i14 | MALPY03118 | 622 | + | 465387 | 466015 | cRMPc10013 | 628 | 13054 | 465387 | 466015 |  | 114 |  | cRMPs1002 | 92114 | 13054 | 373901 | 466015 |  |  |
| i14 | MALPY00976 | 7040 | - | 479069 | 486094 |  |  |  |  |  |  | 260 | 10000 |  |  |  |  |  |  |  |
| i14 | Pb.c001702293.Contig3 | 4028 | + | 479471 | 483482 |  |  |  |  |  |  |  |  |  |  |  |  |  |  |  |
| i14 | Pc.c001404828.Contig1 | 3426 | - | 481744 | 485173 | cRMPc10014 | 7025 | 4964 | 479069 | 486094 |  |  |  |  |  |  |  |  |  |  |
| i14 | Pc.c000502444.Contig3 | 8188 | - | 491058 | 499246 |  |  |  |  |  |  |  |  |  |  |  |  |  |  |  |
| i14 | MALPY03115 | 1922 | + | 497632 | 499651 |  |  |  |  |  | 77 | 260 |  |  |  |  |  |  |  |  |
| i14 | PcContig451 | 2044 | - | 499641 | 501841 |  |  |  |  |  |  |  |  |  |  |  |  |  |  |  |
| i14 | MALPY01839 | 6708 | - | 499819 | 507691 |  |  |  |  |  | 77 | 282 | 3000 |  |  |  |  |  |  |  |
| i14 | Pb.c002007662.Contig1 | 618 | + | 501064 | 501682 |  |  |  |  |  |  |  |  |  |  |  |  |  |  |  |
| i14 | Pc.c001304727.Contig1 | 789 | - | 502556 | 503354 |  |  |  |  |  |  |  |  |  |  |  |  |  |  |  |
| i14 | Pb.c000501100.Contig4 | 4795 | + | 502654 | 508028 |  |  |  |  |  |  |  |  |  |  |  |  |  |  |  |
| i14 | Pc.c000102605.Contig1 | 7002 | - | 505953 | 512955 |  |  |  |  |  |  |  |  |  |  |  |  |  |  |  |
| iman14 | MALPY02879 | 2465 | - | 508284 | 510793 |  |  |  |  |  | 77 |  |  |  |  |  |  |  |  |  |
| i14 | MALPY01665 | 7736 | + | 511493 | 519617 |  |  |  |  |  |  | 282 | 700 |  |  |  |  |  |  |  |
| i14 | Pb.c001101909.Contig1 | 2923 | - | 513390 | 516328 |  |  |  |  |  |  |  |  |  |  |  |  |  |  |  |
| i14 | Pc.c000803789.Contig1 | 820 | - | 514439 | 515277 |  |  |  |  |  |  |  |  |  |  |  |  |  |  |  |
| i14 | Pc.c000102546.Contig1 | 6122 | - | 516043 | 522488 |  |  |  |  |  |  |  |  |  |  |  |  |  |  |  |
| i14 | Pb.c001201637.Contig2 | 7545 | - | 517153 | 524929 |  |  |  |  |  |  |  |  |  |  |  |  |  |  |  |
| i14 | MALPY01572 | 8166 | + | 520272 | 528363 |  |  |  |  |  | 609 | 282 |  |  |  |  |  |  |  |  |
| i14 | Pc.c000202221.Contig3 | 5248 | - | 522805 | 528032 |  |  |  |  |  |  |  |  |  |  |  |  |  |  |  |
| iJC14 | MALPY02672 | 2313 | xxx | 525000 |  |  |  |  |  |  | 609 |  |  |  |  |  |  |  |  |  |
| i14 | Pb.c000001630.Contig1 | 2286 | - | 525275 | 527532 | cRMPc10015 | 37305 | 682 | 491058 | 528363 |  |  |  | cRMPs1003 | 49294 | 682 | 479069 | 528363 |  |  |
| i14 | Pb.c002007461.Contig1 | 1124 | - | 529045 | 530178 |  |  |  |  |  |  |  |  |  |  |  |  |  |  |  |
| i14 | PcContig74 | 4632 | + | 529499 | 534757 |  |  |  |  |  |  |  |  |  |  |  |  |  |  |  |
| i14 | MALPY01522 | 4553 | + | 530556 | 535712 |  |  |  |  |  |  |  |  |  |  |  |  |  |  |  |
| i14 | Pb.c001904285.Contig1 | 3095 | - | 530777 | 534455 | cRMPc10016 | 6667 | 1510 | 529045 | 535712 |  |  |  | cRMPs1004 | 6667 | 1510 | 529045 | 535712 |  |  |
| i14 | PcContig77 | 16622 | + | 537222 | 555558 |  |  |  |  |  |  |  |  |  |  |  |  |  |  |  |
| i14 | MALPY01969 | 4965 | - | 537789 | 542698 |  |  |  |  |  | 362 |  |  |  |  |  |  |  |  |  |
| i14 | Pb.c000701498.Contig1 | 4986 | - | 541270 | 546385 |  |  |  |  |  |  |  |  |  |  |  |  |  |  |  |
| i14 | MALPY01221 | 20201 | + | 542413 | 563818 |  |  |  |  |  | 362 |  |  |  |  |  |  |  |  |  |
| i14 | Pb.c001401755.Contig1 | 3819 | + | 554957 | 559485 |  |  |  |  |  |  |  |  |  |  |  |  |  |  |  |
| i14 | Pc.c001708451.Contig1 | 626 | + | 555930 | 556556 |  |  |  |  |  |  |  |  |  |  |  |  |  |  |  |
| i14 | Pc.c000903394.Contig1 | 2892 | + | 559137 | 562029 |  |  |  |  |  |  |  |  |  |  |  |  |  |  |  |
| iman14 | PcContig50 - Ins | 6086 | + | 562105 | 568994 |  |  |  |  |  |  |  |  |  |  |  |  |  |  |  |
| i14 | Pb.c001001706.Contig1 | 6208 | - | 565118 | 571338 |  |  |  |  |  |  |  |  |  |  |  |  |  |  |  |
| i14 | MALPY00598 | 9702 | + | 565172 | 575212 |  |  |  |  |  | 147 |  |  |  |  |  |  |  |  |  |
| i14 | MALPY03289 | 665 | + | 567882 | 568547 |  |  |  |  |  |  |  |  |  |  |  |  |  |  |  |
| i14 | Pc.c000603147.Contig1 | 635 | + | 568311 | 568937 |  |  |  |  |  |  |  |  |  |  |  |  |  |  |  |
| i14 | Pc.c001504209.Contig1 | 1554 | + | 568774 | 570340 |  |  |  |  |  |  |  |  |  |  |  |  |  |  |  |
| i14 | Pc.c000202782.Contig1 | 1250 | + | 571389 | 572630 |  |  |  |  |  |  |  |  |  |  |  |  |  |  |  |
| i14 | Pb.c001101940.Contig1 | 2280 | - | 571723 | 573979 | cRMPc10017 | 37990 | 1247 | 537222 | 575212 |  |  |  |  |  |  |  |  |  |  |
| i14 | MALPY02069 | 6774 | - | 576459 | 583279 |  |  |  |  |  | 147 | 56 | 6500 |  |  |  |  |  |  |  |
| i14 | Pb.c002112819.Contig1 | 3415 | - | 578381 | 581664 |  |  |  |  |  |  |  |  |  |  |  |  |  |  |  |
| iman14 | Pc.c000603059.Contig1 - Ins | 7965 | - | 579582 | 590289 |  |  |  |  |  |  |  |  |  |  |  |  |  |  |  |
| i14 | Pb.c001904325.Contig1 | 2014 | + | 581067 | 583084 |  |  |  |  |  |  |  |  |  |  |  |  |  |  |  |
| i14 | MALPY00332 | 46765 | + | 585995 | 639469 |  |  |  |  |  | 147 |  |  |  |  |  |  |  |  |  |
| i14 | Pb.c001602392.Contig1 | 8691 | + | 586036 | 596268 |  |  |  |  |  |  |  |  |  |  |  |  |  |  |  |
| i14 | Pc.c001505429.Contig1 | 3811 | - | 593062 | 596879 |  |  |  |  |  |  |  |  |  |  |  |  |  |  |  |
| i14 | MALPY03493 | 878 | + | 594217 | 595095 |  |  |  |  |  |  | 56 |  |  |  |  |  |  |  |  |
| i14 | Pb.c001803857.Contig1 | 1724 | + | 596692 | 598419 |  |  |  |  |  |  |  |  |  |  |  |  |  |  |  |
| i14 | Pc.c001504937.Contig1 | 2157 | - | 597014 | 599174 |  |  |  |  |  |  |  |  |  |  |  |  |  |  |  |
| i14 | Pc.c000102529.Contig1 | 4182 | - | 599593 | 604317 |  |  |  |  |  |  |  |  |  |  |  |  |  |  |  |
| i14 | Pb.c001904282.Contig1 | 1639 | + | 600561 | 602203 |  |  |  |  |  |  |  |  |  |  |  |  |  |  |  |
| i14 | MALPY03105 | 545 | + | 601128 | 601676 |  |  |  |  |  |  |  |  |  |  |  |  |  |  |  |
| i14 | PbContig92 | 20205 | + | 603139 | 627742 |  |  |  |  |  |  |  |  |  |  |  |  |  |  |  |
| i14 | PcContig768 | 6666 | - | 605837 | 613632 |  |  |  |  |  |  |  |  |  |  |  |  |  |  |  |
| i14 | PcContig367 | 1996 | - | 614920 | 617077 |  |  |  |  |  |  |  |  |  |  |  |  |  |  |  |
| i14 | PcContig361 | 4094 | - | 617421 | 622520 |  |  |  |  |  |  |  |  |  |  |  |  |  |  |  |
| i14 | PcContig671 | 11529 | + | 624103 | 636552 |  |  |  |  |  |  |  |  |  |  |  |  |  |  |  |
| i14 | Pb.c000601605.Contig1 | 4687 | - | 628156 | 633183 |  |  |  |  |  |  |  |  |  |  |  |  |  |  |  |
| i14 | Pb.c000401421.Contig1 | 9921 | - | 635331 | 645059 |  |  |  |  |  |  |  |  |  |  |  |  |  |  |  |
| i14 | Pc.c000803445.Contig1 | 3178 | + | 637545 | 640768 |  |  |  |  |  |  |  |  |  |  |  |  |  |  |  |
| i14 | Pc.c000803444.Contig1 | 1826 | + | 640406 | 642313 |  |  |  |  |  |  |  |  |  |  |  |  |  |  |  |
| i14 | MALPY02190 | 4544 | - | 641072 | 645931 |  |  |  |  |  |  | 438B | 1500 |  |  |  |  |  |  |  |
| i14 | Pc.c000803237.Contig1 | 2656 | + | 643471 | 646145 | cRMPc10018 | 69686 | 2275 | 576459 | 646145 |  |  |  |  |  |  |  |  |  |  |
| i14 | MALPY00189 | 24143 | - | 648420 | 674433 |  |  |  |  |  | 104 | 438B |  |  |  |  |  |  |  |  |
| i14 | Pc.c001103088.Contig1 | 10495 | + | 649727 | 660223 |  |  |  |  |  |  |  |  |  |  |  |  |  |  |  |
| i14 | PcContig477 | 7987 | + | 661641 | 669931 |  |  |  |  |  |  |  |  |  |  |  |  |  |  |  |
| i14 | Pb.c000001368.Contig1 | 17239 | - | 662006 | 683014 |  |  |  |  |  |  |  |  |  |  |  |  |  |  |  |
| i14 | Pc.c001405330.Contig1 | 937 | - | 670300 | 671690 |  |  |  |  |  |  |  |  |  |  |  |  |  |  |  |
| i14 | MALPY01368 | 11218 | - | 676007 | 688586 |  |  |  |  |  | 104 | 136 - Ins | 2500 |  |  |  |  |  |  |  |
| i14 | PcContig10 | 5470 | + | 676352 | 682002 |  |  |  |  |  |  |  |  |  |  |  |  |  |  |  |
| i14 | Pc.c000603053.Contig1 | 4633 | + | 683493 | 688309 |  |  |  |  |  |  |  |  |  |  |  |  |  |  |  |
| i14 | Pb.c002006936.Contig1 | 2797 | - | 684143 | 686940 | cRMPc10019 | 40166 | 2500 | 648420 | 688586 |  |  |  |  |  |  |  |  |  |  |
| i14 | MALPY03046 | 1480 | + | 691086 | 692565 | cRMPc10020 | 1479 | 326 | 691086 | 692565 | 97 | 136 - Ins |  |  |  |  |  |  |  |  |
| i14 | MALPY01877 | 3221 | + | 692891 | 696295 | cRMPc10021 | 3404 | 661 | 692891 | 696295 | 97 | 363 | 1000 |  |  |  |  |  |  |  |
| i14 | Pc.c000903260.Contig1 | 4125 | + | 696956 | 701075 |  |  |  |  |  |  |  |  |  |  |  |  |  |  |  |
| i14 | MALPY02326 | 4693 | - | 697570 | 702260 | cRMPc10022 | 5304 | 854 | 696956 | 702260 |  | 363 | 2000 |  |  |  |  |  |  |  |
| i14 | MALPY02391 | 5265 | - | 703114 | 708373 |  |  |  |  |  |  | 363 | 1400 |  |  |  |  |  |  |  |
| i14 | Pb.c001702339.Contig1 | 14026 | + | 706870 | 720771 |  |  |  |  |  |  |  |  |  |  |  |  |  |  |  |
| i14 | Pb.c000701490.Contig1 | 2281 | - | 707888 | 710169 |  |  |  |  |  |  |  |  |  |  |  |  |  |  |  |
| i14 | MALPY02567 | 4234 | - | 708808 | 712719 |  |  |  |  |  | 135 | 363 |  |  |  |  |  |  |  |  |
| i14 | Pc.c000002611.Contig1 | 618 | - | 710351 | 710969 |  |  |  |  |  |  |  |  |  |  |  |  |  |  |  |
| i14 | Pc.c001203944.Contig1 | 4832 | + | 710643 | 715413 |  |  |  |  |  |  |  |  |  |  |  |  |  |  |  |
| i14 | MALPY02587 | 3487 | - | 711917 | 715401 |  |  |  |  |  | 135 | 27 | 1900 |  |  |  |  |  |  |  |
| i14 | MALPY00583 | 4316 | + | 716973 | 721371 | cRMPc10023 | 18257 | 9000 | 703114 | 721371 |  | 27 | 9000 |  |  |  |  |  |  |  |
| Centromere - PCR 9000 bp | |  |  | 720000 |  |  |  |  |  |  |  |  |  |  |  |  |  |  |  |  |
| i14 | MALPY01896 | 2104 | - | 730371 | 732510 | cRMPc10024 | 2139 | 5 | 730371 | 732510 | 254 | 27 |  |  |  |  |  |  |  |  |
| i14 | MALPY03048 | 1501 | - | 732515 | 734022 | cRMPc10025 | 1507 | 512 | 732515 | 734022 | 254 |  |  |  |  |  |  |  |  |  |
| iJC14 | MALPY02554 | 5959 | + | 734534 | 740493 | cRMPc10026 | 5959 | 2489 | 734534 | 740493 | 254 |  |  |  |  |  |  |  |  |  |
| i14 | MALPY00953 | 3781 | - | 742982 | 746976 |  |  |  |  |  | 254 |  |  |  |  |  |  |  |  |  |
| i14 | Pb.c001301879.Contig1 | 2742 | - | 744486 | 747231 |  |  |  |  |  |  |  |  |  |  |  |  |  |  |  |
| i14 | MALPY00280 | 9607 | + | 746811 | 756951 |  |  |  |  |  |  |  |  |  |  |  |  |  |  |  |
| i14 | Pb.c000301461.Contig1 | 4276 | - | 747123 | 751262 |  |  |  |  |  |  |  |  |  |  |  |  |  |  |  |
| i14 | Pc.c000102700.Contig1 | 3419 | + | 747311 | 750727 |  |  |  |  |  |  |  |  |  |  |  |  |  |  |  |
| i14 | MALPY03310 | 218 | - | 748121 | 748333 |  |  |  |  |  |  |  |  |  |  |  |  |  |  |  |
| i14 | Pc.c001605994.Contig1 | 1633 | + | 750602 | 752112 |  |  |  |  |  |  |  |  |  |  |  |  |  |  |  |
| i14 | Pc.c001103935.Contig1 | 1986 | + | 754408 | 756394 | cRMPc10027 | 13969 | 1234 | 742982 | 756951 |  |  |  | cRMPs1005 | 219729 | 1234 | 537222 | 756951 |  |  |
| i14 | MALPY00056 | 18465 | + | 758185 | 777108 |  |  |  |  |  | 167 |  |  |  |  |  |  |  |  |  |
| i14 | Pb.c001502594.Contig1 | 5492 | - | 758704 | 764238 |  |  |  |  |  |  |  |  |  |  |  |  |  |  |  |
| i14 | PcContig402 | 4680 | + | 759666 | 764401 |  |  |  |  |  |  |  |  |  |  |  |  |  |  |  |
| i14 | MALPY03298 | 827 | + | 760622 | 761449 |  |  |  |  |  |  |  |  |  |  |  |  |  |  |  |
| i14 | PcContig253 | 8514 | + | 767099 | 775926 |  |  |  |  |  |  |  |  |  |  |  |  |  |  |  |
| i14 | Pb.c001402112.Contig1 | 1823 | + | 768005 | 769849 |  |  |  |  |  |  |  |  |  |  |  |  |  |  |  |
| i14 | Pb.c001101897.Contig1 | 4340 | - | 772504 | 776567 |  |  |  |  |  |  |  |  |  |  |  |  |  |  |  |
| i14 | MALPY01620 | 6668 | + | 775671 | 782784 |  |  |  |  |  | 167 |  |  |  |  |  |  |  |  |  |
| i14 | Pc.c001708076.Contig1 | 4361 | - | 775740 | 780104 |  |  |  |  |  |  |  |  |  |  |  |  |  |  |  |
| i14 | MALPY03564 | 212 | - | 775878 | 776090 |  |  |  |  |  |  |  |  |  |  |  |  |  |  |  |
| iman14 | Pb.c001802849.Contig2 - Ins | 14216 | - | 777110 | 793333 |  |  |  |  |  |  |  |  |  |  |  |  |  |  |  |
| i14 | MALPY02279 | 7219 | + | 784020 | 791257 |  |  |  |  |  |  |  |  |  |  |  |  |  |  |  |
| i14 | Pb.c001202346.Contig1 | 901 | + | 788367 | 789265 | cRMPc10028 | 35148 | 494 | 758185 | 793333 |  |  |  | cRMPs1006 | 35148 | 494 | 758185 | 793333 |  |  |
| i14 | Pc.c000602347.Contig1 | 3460 | - | 793827 | 798074 |  |  |  |  |  |  |  |  |  |  |  |  |  |  |  |
| i14 | MALPY02383 | 10086 | + | 793959 | 805807 |  |  |  |  |  |  |  |  |  |  |  |  |  |  |  |
| i14 | Pb.c000901827.Contig1 | 4678 | + | 794412 | 799870 |  |  |  |  |  |  |  |  |  |  |  |  |  |  |  |
| i14 | Pc.c000503090.Contig1 | 4660 | - | 799359 | 804031 |  |  |  |  |  |  |  |  |  |  |  |  |  |  |  |
| i14 | Pb.c000401467.Contig1 | 4510 | + | 801107 | 805602 | cRMPc10029 | 11980 | 621 | 793827 | 805807 |  |  |  | cRMPs1007 | 11980 | 621 | 793827 | 805807 |  |  |
| i14 | MALPY02194 | 9178 | - | 806428 | 816283 |  |  |  |  |  | 420 | 364 |  |  |  |  |  |  |  |  |
| i14 | Pb.c001001828.Contig1 | 1776 | + | 806560 | 808336 |  |  |  |  |  |  |  |  |  |  |  |  |  |  |  |
| i14 | Pc.c001003833.Contig1 | 793 | - | 807278 | 808071 |  |  |  |  |  |  |  |  |  |  |  |  |  |  |  |
| i14 | PcContig234 | 5003 | - | 808115 | 813881 |  |  |  |  |  |  |  |  |  |  |  |  |  |  |  |
| i14 | Pb.c000101304.Contig1 | 4716 | - | 810534 | 815286 |  |  |  |  |  |  |  |  |  |  |  |  |  |  |  |
| i14 | MALPY03482 | 835 | + | 811274 | 812109 | cRMPc10030 | 9855 | 132 | 806428 | 816283 |  | 364 | 3000 |  |  |  |  |  |  |  |
| i14 | MALPY02530 | 3490 | - | 816415 | 819896 |  |  |  |  |  | 420 |  |  |  |  |  |  |  |  |  |
| i14 | PcContig586 | 2656 | + | 817872 | 820522 | cRMPc10031 | 4107 | 4116 | 816415 | 820522 |  |  |  | cRMPs1008 | 14094 | 4116 | 806428 | 820522 |  |  |
| i14 | MALPY03141 | 1428 | - | 824638 | 826162 |  |  |  |  |  | 132 |  |  |  |  |  |  |  |  |  |
| i14 | PbContig106 | 4571 | - | 824796 | 830387 |  |  |  |  |  |  |  |  |  |  |  |  |  |  |  |
| i14 | MALPY02588 | 3030 | + | 826397 | 829743 |  |  |  |  |  | 132 |  |  |  |  |  |  |  |  |  |
| i14 | Pc.c001605835.Contig1 | 3750 | + | 827792 | 831682 |  |  |  |  |  |  |  |  |  |  |  |  |  |  |  |
| i14 | MALPY02705 | 2133 | - | 829483 | 831607 | cRMPc10032 | 7044 | 1560 | 824638 | 831682 | 132 |  |  | cRMPs1009 | 7044 | 1560 | 824638 | 831682 |  |  |
| iman14 | MALPY01836 | 3984 | + | 833242 | 837913 | cRMPc10033 | 4671 | 1269 | 833242 | 837913 | 301 |  |  |  |  |  |  |  |  |  |
| i14 | MALPY00005 | 47128 | + | 839182 | 890751 |  |  |  |  |  | 301 | 38 |  |  |  |  |  |  |  |  |
| i14 | Pb.c001102005.Contig1 | 7307 | - | 842302 | 850223 |  |  |  |  |  |  |  |  |  |  |  |  |  |  |  |
| i14 | PcContig307 | 15734 | - | 844061 | 862067 |  |  |  |  |  |  |  |  |  |  |  |  |  |  |  |
| i14 | Pb.c001201624.Contig1 | 10049 | + | 851479 | 863054 |  |  |  |  |  |  |  |  |  |  |  |  |  |  |  |
| i14 | Pb.c001602556.Contig1 | 6318 | + | 863699 | 870581 |  |  |  |  |  |  |  |  |  |  |  |  |  |  |  |
| i14 | PcContig461 | 6094 | + | 866814 | 873876 |  |  |  |  |  |  |  |  |  |  |  |  |  |  |  |
| i14 | Pb.c001602849.Contig1 | 1508 | + | 870940 | 872640 |  |  |  |  |  |  |  |  |  |  |  |  |  |  |  |
| i14 | Pc.c001404015.Contig2 | 3765 | - | 874996 | 878764 |  |  |  |  |  |  |  |  |  |  |  |  |  |  |  |
| i14 | PbContig298 | 8453 | - | 875380 | 884221 |  |  |  |  |  |  |  |  |  |  |  |  |  |  |  |
| i14 | MALPY03140 | 199 | + | 879040 | 879239 |  |  |  |  |  |  | 38 | 1700 |  |  |  |  |  |  |  |
| i14 | Pc.c001606396.Contig1 | 662 | - | 879247 | 879871 |  |  |  |  |  |  |  |  |  |  |  |  |  |  |  |
| i14 | PbContig219 | 3764 | - | 889403 | 893245 |  |  |  |  |  |  |  |  |  |  |  |  |  |  |  |
| i14 | Pc.c001605882.Contig1 | 4179 | + | 890469 | 894801 |  |  |  |  |  |  |  |  |  |  |  |  |  |  |  |
| i14 | MALPY01373 | 4833 | - | 891074 | 896611 | cRMPc10034 | 57429 | 299 | 839182 | 896611 | 301 |  |  |  |  |  |  |  |  |  |
| iman14 | MALPY01646 | 3075 | - | 896910 | 899896 |  |  |  |  |  | 301 |  |  |  |  |  |  |  |  |  |
| i14 | Pb.c001803479.Contig1 | 3220 | + | 899471 | 902694 |  |  |  |  |  |  |  |  |  |  |  |  |  |  |  |
| i14 | MALPY02623 | 4825 | - | 900066 | 905125 |  |  |  |  |  | 301 |  |  |  |  |  |  |  |  |  |
| iman14 | Pb.c001803317.Contig1 - Ins | 6127 | + | 903086 | 910304 |  |  |  |  |  |  |  |  |  |  |  |  |  |  |  |
| i14 | MALPY01189 | 2688 | - | 907101 | 909926 | cRMPc10035 | 13394 | 200 | 896910 | 910304 |  |  |  | cRMPs1010 | 77062 | 200 | 833242 | 910304 |  |  |
| i14 | PcContig475 | 6838 | + | 910504 | 917735 |  |  |  |  |  |  |  |  |  |  |  |  |  |  |  |
| i14 | MALPY01855 | 9132 | - | 913994 | 923364 |  |  |  |  |  |  | 211 | 1200 |  |  |  |  |  |  |  |
| i14 | Pb.c000901489.Contig2 | 6693 | + | 916308 | 923116 |  |  |  |  |  |  |  |  |  |  |  |  |  |  |  |
| i14 | MALPY03519 | 399 | + | 917697 | 918093 |  |  |  |  |  |  |  |  |  |  |  |  |  |  |  |
| i14 | PcContig415 | 3036 | + | 917974 | 920959 | cRMPc10036 | 12860 | 4464 | 910504 | 923364 |  |  |  |  |  |  |  |  |  |  |
| i14 | Pb.c000901739.Contig1 | 3561 | + | 927828 | 930990 |  |  |  |  |  |  |  |  |  |  |  |  |  |  |  |
| iman14 | MALPY02366 | 2703 | - | 928165 | 931380 |  |  |  |  |  | 1184 |  |  |  |  |  |  |  |  |  |
| i14 | MALPY03078 | 1210 | + | 930586 | 931951 | cRMPc10037 | 4123 | 637 | 927828 | 931951 | 1184 | 211 |  |  |  |  |  |  |  |  |
| i14 | Pc.c000803383.Contig1 | 2882 | - | 932588 | 935772 |  |  |  |  |  |  |  |  |  |  |  |  |  |  |  |
| i14 | MALPY00317 | 33648 | + | 933004 | 969385 |  |  |  |  |  | 1184 | 455 | 2500 |  |  |  |  |  |  |  |
| i14 | Pb.c001502409.Contig1 | 1534 | - | 933875 | 935756 |  |  |  |  |  |  |  |  |  |  |  |  |  |  |  |
| i14 | Pc.c001204638.Contig1 | 984 | - | 935799 | 936954 |  |  |  |  |  |  |  |  |  |  |  |  |  |  |  |
| i14 | PbContig103 | 3711 | - | 935821 | 940650 |  |  |  |  |  |  |  |  |  |  |  |  |  |  |  |
| i14 | Pc.c000803211.Contig1 | 1951 | - | 938032 | 939977 |  |  |  |  |  |  |  |  |  |  |  |  |  |  |  |
| i14 | Pb.c002112865.Contig1 | 978 | - | 941368 | 942346 |  |  |  |  |  |  |  |  |  |  |  |  |  |  |  |
| i14 | Pb.c000501480.Contig1 | 3237 | - | 942720 | 945984 |  |  |  |  |  |  |  |  |  |  |  |  |  |  |  |
| i14 | PcContig648 | 944 | + | 943698 | 944681 |  |  |  |  |  |  |  |  |  |  |  |  |  |  |  |
| i14 | Pc.c001203921.Contig1 | 1186 | + | 944739 | 945928 |  |  |  |  |  |  |  |  |  |  |  |  |  |  |  |
| i14 | Pb.c001401783.Contig2 | 8203 | + | 947889 | 956616 |  |  |  |  |  |  |  |  |  |  |  |  |  |  |  |
| i14 | Pc.c000703220.Contig1 | 2486 | + | 949951 | 952413 |  |  |  |  |  |  |  |  |  |  |  |  |  |  |  |
| i14 | Pc.c001103042.Contig1 | 3819 | + | 953113 | 957382 |  |  |  |  |  |  |  |  |  |  |  |  |  |  |  |
| i14 | PbContig332 | 8895 | + | 956807 | 966128 |  |  |  |  |  |  |  |  |  |  |  |  |  |  |  |
| i14 | PcContig94 | 2635 | - | 957470 | 960516 |  |  |  |  |  |  |  |  |  |  |  |  |  |  |  |
| i14 | PcContig710 | 7443 | + | 960472 | 968078 | cRMPc10038 | 36797 | 3118 | 932588 | 969385 |  |  |  |  |  |  |  |  |  |  |
| i14 | MALPY01208 | 4460 | + | 972503 | 977616 | cRMPc10039 | 5113 | 109 | 972503 | 977616 |  | 455 | 1200 |  |  |  |  |  |  |  |
| i14 | Pc.c000602907.Contig1 | 1784 | + | 977725 | 979509 |  |  |  |  |  |  |  |  |  |  |  |  |  |  |  |
| i14 | MALPY01597 | 3119 | + | 978024 | 981263 |  |  |  |  |  |  | 455 | 2800 |  |  |  |  |  |  |  |
| i14 | Pb.c002005909.Contig1 | 1609 | + | 978724 | 980333 |  |  |  |  |  |  |  |  |  |  |  |  |  |  |  |
| i14 | Pc.c000503504.Contig1 | 897 | + | 979923 | 980838 | cRMPc10040 | 3538 | 1227 | 977725 | 981263 |  |  |  |  |  |  |  |  |  |  |
| i14 | PcContig394 | 4699 | + | 982490 | 987319 |  |  |  |  |  |  |  |  |  |  |  |  |  |  |  |
| i14 | Pb.c001301741.Contig1 | 2888 | + | 982731 | 985619 |  |  |  |  |  |  |  |  |  |  |  |  |  |  |  |
| i14 | MALPY01040 | 5623 | - | 984343 | 990222 |  |  |  |  |  | 503B | 455 |  |  |  |  |  |  |  |  |
| i14 | Pc.c000003051.Contig1 | 848 | + | 987571 | 988410 |  |  |  |  |  |  |  |  |  |  |  |  |  |  |  |
| i14 | Pb.c002007078.Contig1 | 1078 | - | 987836 | 989082 |  |  |  |  |  |  |  |  |  |  |  |  |  |  |  |
| i14 | PcContig693 | 2240 | + | 988753 | 990990 |  |  |  |  |  |  |  |  |  |  |  |  |  |  |  |
| iman14 | PbContig5886 | 1972 | - | 989088 | 991060 |  |  |  |  |  |  |  |  |  |  |  |  |  | PcContig693 |  |
| iman14 | MALPY02529 | 3228 | - | 990200 | 993428 |  |  |  |  |  | 503B |  |  |  |  |  |  |  | \| |  |
| iman14 | PbContig4015 | 3728 | + | 992971 | 996699 |  |  |  |  |  |  |  |  |  |  |  |  |  | MALPY00710 |  |
| i14 | MALPY00710 | 3259 | + | 993940 | 997298 | cRMPc10041 | 14808 | 31 | 982490 | 997298 | 503B | 214 | 1600 |  |  |  |  |  |  |  |
| i14 | Pb.c000001760.Contig1 | 3239 | + | 997329 | 1000572 |  |  |  |  |  |  |  |  |  |  |  |  |  |  |  |
| i14 | MALPY02277 | 7902 | + | 998010 | 1004822 |  |  |  |  |  | 897 | 214 |  |  |  |  |  |  |  |  |
| i14 | Pc.c000402756.Contig1 | 1736 | - | 998940 | 1000679 |  |  |  |  |  |  |  |  |  |  |  |  |  |  |  |
| i14 | Pc.c001003247.Contig1 | 3293 | + | 1000903 | 1003286 |  |  |  |  |  |  |  |  |  |  |  |  |  |  |  |
| i14 | Pb.c000601219.Contig1 | 12327 | + | 1002847 | 1015773 |  |  |  |  |  |  |  |  |  |  |  |  |  |  |  |
| i14 | Pc.c000502380.Contig1 | 3257 | + | 1004746 | 1008003 |  |  |  |  |  |  |  |  |  |  |  |  |  |  |  |
| iman14 | MALPY01641 | 3877 | + | 1005971 | 1009848 |  |  |  |  |  | 897 |  |  |  |  |  |  |  |  |  |
| iman14 | MALPY01376 | 6466 | + | 1009738 | 1016284 | cRMPc10042 | 18955 | 1025 | 997329 | 1016284 | 897 |  |  | cRMPs1011 | 105780 | 1025 | 910504 | 1016284 |  |  |
| i14 | MALPY03148 | 1763 | + | 1017309 | 1019171 |  |  |  |  |  |  |  |  |  |  |  |  |  |  |  |
| i14 | Pc.c001504997.Contig1 | 1154 | + | 1017384 | 1018644 | cRMPc10043 | 1862 | 770 | 1017309 | 1019171 |  |  |  | cRMPs1012 | 1862 | 770 | 1017309 | 1019171 |  |  |
| i14 | MALPY00442 | 15025 | + | 1019941 | 1035924 |  |  |  |  |  |  |  |  |  |  |  |  |  |  |  |
| i14 | Pb.c000500672.Contig2 | 13666 | + | 1020364 | 1034963 |  |  |  |  |  |  |  |  |  |  |  |  |  |  |  |
| i14 | Pc.c000002491.Contig1 | 6266 | + | 1021028 | 1028237 |  |  |  |  |  |  |  |  |  |  |  |  |  |  |  |
| i14 | Pc.c000002712.Contig1 | 2718 | + | 1028458 | 1031177 | cRMPc10044 | 15983 | 3927 | 1019941 | 1035924 |  |  |  | cRMPs1013 | 15983 | 3927 | 1019941 | 1035924 |  |  |
| i14 | MALPY01736 | 22506 | + | 1039851 | 1065103 |  |  |  |  |  | 247 |  |  |  |  |  |  |  |  |  |
| i14 | Pb.c001702340.Contig1 | 7683 | + | 1040002 | 1048546 |  |  |  |  |  |  |  |  |  |  |  |  |  |  |  |
| i14 | PcContig289 | 1342 | + | 1041915 | 1043254 |  |  |  |  |  |  |  |  |  |  |  |  |  |  |  |
| i14 | PcContig62 | 7046 | + | 1047121 | 1054176 |  |  |  |  |  |  |  |  |  |  |  |  |  |  |  |
| i14 | Pb.c001001756.Contig1 | 3488 | + | 1054749 | 1058208 |  |  |  |  |  |  |  |  |  |  |  |  |  |  |  |
| i14 | Pc.c000503565.Contig1 | 1101 | + | 1056496 | 1057566 |  |  |  |  |  |  |  |  |  |  |  |  |  |  |  |
| i14 | MALPY03251 | 829 | - | 1058273 | 1059096 | cRMPc10045 | 25252 | 524 | 1039851 | 1065103 |  |  |  |  |  |  |  |  |  |  |
| iman14 | MALPY01647 | 4031 | + | 1065627 | 1069777 |  |  |  |  |  | 247 |  |  |  |  |  |  |  |  |  |
| i14 | MALPY00869 | 9433 | - | 1069583 | 1079391 |  |  |  |  |  | 247 |  |  |  |  |  |  |  |  |  |
| i14 | Pb.c000501383.Contig1 | 7720 | - | 1075366 | 1083505 |  |  |  |  |  |  |  |  |  |  |  |  |  |  |  |
| i14 | MALPY00117 | 10336 | + | 1079670 | 1090207 |  |  |  |  |  | 247 | 103\_alt | 3800 |  |  |  |  |  |  |  |
| i14 | Pc.c001304930.Contig1 | 754 | - | 1084675 | 1085396 |  |  |  |  |  |  |  |  |  |  |  |  |  |  |  |
| i14 | Pb.c001803338.Contig1 | 4853 | - | 1085161 | 1090014 | cRMPc10046 | 24580 | 3681 | 1065627 | 1090207 |  |  |  |  |  |  |  |  |  |  |
| i14 | Pb.c001602532.Contig1 | 3704 | - | 1093888 | 1097592 |  |  |  |  |  |  |  |  |  |  |  |  |  |  |  |
| i14 | MALPY00412 | 2493 | + | 1094225 | 1096718 |  |  |  |  |  |  | 103\_alt | 1400 |  |  |  |  |  |  |  |
| i14 | MALPY01829 | 8500 | - | 1096538 | 1105139 |  |  |  |  |  |  | 103\_alt | 2500 |  |  |  |  |  |  |  |
| i14 | PbContig51 | 5930 | - | 1097573 | 1103682 |  |  |  |  |  |  |  |  |  |  |  |  |  |  |  |
| i14 | Pc.c001708239.Contig1 | 738 | + | 1100238 | 1100973 |  |  |  |  |  |  |  |  |  |  |  |  |  |  |  |
| i14 | Pc.c001303470.Contig2 | 1551 | + | 1101228 | 1102779 | cRMPc10047 | 11251 | 2196 | 1093888 | 1105139 |  |  |  |  |  |  |  |  |  |  |
| i14 | MALPY01284 | 10832 | - | 1107335 | 1118164 |  |  |  |  |  | 956\_alt | 103\_alt |  |  |  |  |  |  |  |  |
| i14 | PcContig235 | 9754 | + | 1110401 | 1121053 |  |  |  |  |  |  |  |  |  |  |  |  |  |  |  |
| i14 | MALPY02266 | 6369 | + | 1118684 | 1125158 |  |  |  |  |  | 956\_alt | 184 | 900 |  |  |  |  |  |  |  |
| i14 | Pb.c001904404.Contig1 | 2605 | + | 1118700 | 1121299 |  |  |  |  |  |  |  |  |  |  |  |  |  |  |  |
| i14 | Pb.c002006836.Contig1 | 3425 | + | 1121805 | 1125308 |  |  |  |  |  |  |  |  |  |  |  |  |  |  |  |
| i14 | Pc.c001104309.Contig1 | 695 | - | 1123084 | 1123892 |  |  |  |  |  |  |  |  |  |  |  |  |  |  |  |
| i14 | Pc.c000602946.Contig1 | 1406 | - | 1124205 | 1125611 | cRMPc10048 | 18276 | 3284 | 1107335 | 1125611 |  |  |  |  |  |  |  |  |  |  |
| i14 | MALPY01453 | 5323 | - | 1128895 | 1134323 |  |  |  |  |  |  | 153 | 2200 |  |  |  |  |  |  |  |
| i14 | Pb.c000301135.Contig1 | 3879 | - | 1129591 | 1133443 |  |  |  |  |  |  |  |  |  |  |  |  |  |  |  |
| i14 | Pc.c000303473.Contig1 | 975 | + | 1131483 | 1132458 |  |  |  |  |  |  |  |  |  |  |  |  |  |  |  |
| i14 | MALPY03456 | 829 | + | 1131949 | 1132787 |  |  |  |  |  |  | 184 |  |  |  |  |  |  |  |  |
| i14 | Pc.c000203274.Contig1 | 854 | + | 1132645 | 1133562 | cRMPc10049 | 5428 | 414 | 1128895 | 1134323 |  |  |  |  |  |  |  |  |  |  |
| i14 | MALPY02118 | 2841 | - | 1134737 | 1137554 |  |  |  |  |  |  | 273 | 1000 |  |  |  |  |  |  |  |
| i14 | Pc.c000402843.Contig1 | 4658 | - | 1135013 | 1139740 |  |  |  |  |  |  |  |  |  |  |  |  |  |  |  |
| i14 | Pb.c000301497.Contig1 | 2790 | - | 1135272 | 1138035 |  |  |  |  |  |  |  |  |  |  |  |  |  |  |  |
| i14 | MALPY03373 | 827 | - | 1136034 | 1136834 |  |  |  |  |  |  | 153 |  |  |  |  |  |  |  |  |
| i14 | MALPY02460 | 6503 | + | 1137652 | 1144171 |  |  |  |  |  | 1197 | 273 |  |  |  |  |  |  |  |  |
| i14 | Pb.c000301131.Contig2 | 5742 | - | 1138186 | 1143987 |  |  |  |  |  |  |  |  |  |  |  |  |  |  |  |
| i14 | PcContig86 | 8508 | - | 1139617 | 1150987 |  |  |  |  |  |  |  |  |  |  |  |  |  |  |  |
| i14 | MALPY00108 | 8813 | + | 1146270 | 1155080 |  |  |  |  |  | 1197 |  |  |  |  |  |  |  |  |  |
| i14 | Pb.c001001807.Contig1 | 4447 | - | 1147178 | 1151622 | cRMPc10050 | 20343 | 4317 | 1134737 | 1155080 |  |  |  | cRMPs1014 | 115229 | 4317 | 1039851 | 1155080 |  |  |
| iman14 | MALPY00100 | 5764 | + | 1159397 | 1166001 | cRMPc10051 | 6604 | 765 | 1159397 | 1166001 |  |  |  | cRMPs1015 | 6604 | 765 | 1159397 | 1166001 |  |  |
| i14 | PbContig223 | 5707 | - | 1166766 | 1173757 |  |  |  |  |  |  |  |  |  |  |  |  |  |  |  |
| i14 | Pc.c001505700.Contig1 | 1065 | + | 1167350 | 1168415 |  |  |  |  |  |  |  |  |  |  |  |  |  |  |  |
| i14 | MALPY01362 | 12749 | + | 1167654 | 1181199 |  |  |  |  |  | 708 |  |  |  |  |  |  |  |  |  |
| i14 | PcContig313 | 4122 | - | 1168438 | 1173952 |  |  |  |  |  |  |  |  |  |  |  |  |  |  |  |
| i14 | Pc.c000102466.Contig1 | 2924 | - | 1175045 | 1177969 |  |  |  |  |  |  |  |  |  |  |  |  |  |  |  |
| i14 | Pb.c000901755.Contig1 | 4136 | - | 1175815 | 1179951 |  |  |  |  |  |  |  |  |  |  |  |  |  |  |  |
| i14 | Pc.c000602870.Contig1 | 3663 | - | 1178035 | 1181698 | cRMPc10052 | 14932 | 132 | 1166766 | 1181698 |  |  |  |  |  |  |  |  |  |  |
| i14 | MALPY02171 | 2852 | + | 1181830 | 1184992 |  |  |  |  |  | 708 |  |  |  |  |  |  |  |  |  |
| i14 | Pc.c000403273.Contig1 | 1377 | - | 1182764 | 1184357 |  |  |  |  |  |  |  |  |  |  |  |  |  |  |  |
| i14 | Pb.c001602458.Contig1 | 2133 | - | 1183122 | 1185435 | cRMPc10053 | 3605 | 99 | 1181830 | 1185435 |  |  |  | cRMPs1016 | 18669 | 99 | 1166766 | 1185435 |  |  |
| i14 | Pc.c000102938.Contig1 | 6176 | - | 1185534 | 1191836 |  |  |  |  |  |  |  |  |  |  |  |  |  |  |  |
| i14 | MALPY01886 | 3644 | + | 1187888 | 1191688 |  |  |  |  |  | 560 |  |  |  |  |  |  |  |  |  |
| i14 | Pb.c000701546.Contig1 | 4208 | - | 1190965 | 1195025 |  |  |  |  |  |  |  |  |  |  |  |  |  |  |  |
| i14 | MALPY01602 | 2431 | + | 1191315 | 1193974 | cRMPc10054 | 9491 | 321 | 1185534 | 1195025 | 560 |  |  |  |  |  |  |  |  |  |
| i14 | MALPY01317 | 4811 | - | 1195346 | 1201606 |  |  |  |  |  | 560 |  |  |  |  |  |  |  |  |  |
| i14 | Pb.c002112195.Contig1 | 2185 | - | 1196296 | 1198550 |  |  |  |  |  |  |  |  |  |  |  |  |  |  |  |
| i14 | PcContig721 | 1955 | + | 1198884 | 1200854 |  |  |  |  |  |  |  |  |  |  |  |  |  |  |  |
| i14 | Pb.c001202078.Contig1 | 1308 | - | 1199235 | 1200550 | cRMPc10055 | 6260 | 838 | 1195346 | 1201606 |  |  |  |  |  |  |  |  |  |  |
| i14 | MALPY02565 | 6654 | + | 1202444 | 1210004 |  |  |  |  |  | 560 | 467 | 2000 |  |  |  |  |  |  |  |
| i14 | Pb.c001502935.Contig1 | 2142 | - | 1204181 | 1206317 |  |  |  |  |  |  |  |  |  |  |  |  |  |  |  |
| i14 | Pc.c001812942.Contig1 | 687 | - | 1206794 | 1207481 |  |  |  |  |  |  |  |  |  |  |  |  |  |  |  |
| i14 | Pb.c001904506.Contig1 | 1178 | - | 1207724 | 1209025 | cRMPc10056 | 7560 | 478 | 1202444 | 1210004 |  |  |  |  |  |  |  |  |  |  |
| i14 | Pc.c001003457.Contig1 | 4540 | - | 1210482 | 1215023 |  |  |  |  |  |  |  |  |  |  |  |  |  |  |  |
| i14 | Pb.c001703089.Contig1 | 2643 | + | 1211499 | 1214142 |  |  |  |  |  |  |  |  |  |  |  |  |  |  |  |
| i14 | MALPY02734 | 3235 | - | 1211846 | 1215081 | cRMPc10057 | 4599 | 4474 | 1210482 | 1215081 |  | 467 |  | cRMPs1017 | 29547 | 4474 | 1185534 | 1215081 |  |  |
| i14 | MALPY00937 | 3545 | - | 1219555 | 1223100 |  |  |  |  |  | 94 |  |  |  |  |  |  |  |  |  |
| i14 | Pc.c001304173.Contig1 | 4839 | + | 1219875 | 1224771 |  |  |  |  |  |  |  |  |  |  |  |  |  |  |  |
| i14 | MALPY00938 | 2175 | + | 1222484 | 1224686 |  |  |  |  |  | 94 | 194 | 2800 |  |  |  |  |  |  |  |
| i14 | Pb.c002007027.Contig1 | 1699 | + | 1222768 | 1224539 | cRMPc10058 | 5216 | 2977 | 1219555 | 1224771 |  |  |  |  |  |  |  |  |  |  |
| i14 | MALPY01804 | 6029 | - | 1227748 | 1233780 |  |  |  |  |  | 249 | 194 |  |  |  |  |  |  |  |  |
| i14 | Pb.c000801799.Contig1 | 5572 | + | 1229563 | 1235822 |  |  |  |  |  |  |  |  |  |  |  |  |  |  |  |
| i14 | PcContig209 | 3211 | - | 1229702 | 1232913 |  |  |  |  |  |  |  |  |  |  |  |  |  |  |  |
| i14 | Pc.c001605864.Contig1 | 3416 | - | 1234041 | 1237464 |  |  |  |  |  |  |  |  |  |  |  |  |  |  |  |
| i14 | MALPY00369 | 10776 | + | 1234146 | 1244953 | cRMPc10059 | 17205 | 796 | 1227748 | 1244953 | 249 |  |  |  |  |  |  |  |  |  |
| i14 | Pb.c000201631.Contig1 | 4670 | - | 1245749 | 1250671 |  |  |  |  |  |  |  |  |  |  |  |  |  |  |  |
| i14 | MALPY03192 | 1068 | + | 1246484 | 1247567 |  |  |  |  |  | 249 |  |  |  |  |  |  |  |  |  |
| i14 | Pc.c001103786.Contig1 | 4028 | - | 1246993 | 1251139 |  |  |  |  |  |  |  |  |  |  |  |  |  |  |  |
| i14 | MALPY02572 | 4953 | - | 1247621 | 1252565 | cRMPc10060 | 6816 | 25 | 1245749 | 1252565 | 249 |  |  | cRMPs1018 | 33010 | 25 | 1219555 | 1252565 |  |  |
| i14 | Pc.c001405450.Contig1 | 1042 | - | 1252590 | 1253644 |  |  |  |  |  |  |  |  |  |  |  |  |  |  |  |
| i14 | MALPY03453 | 858 | + | 1252778 | 1253648 | cRMPc10061 | 1058 | 4752 | 1252590 | 1253648 |  | 311 | 6000 |  |  |  |  |  |  |  |
| i14 | MALPY01822 | 4792 | + | 1258400 | 1263096 |  |  |  |  |  |  | 311 |  |  |  |  |  |  |  |  |
| i14 | Pb.c000501412.Contig1 | 4494 | - | 1259553 | 1263968 | cRMPc10062 | 5568 | 1621 | 1258400 | 1263968 |  |  |  | cRMPs1019 | 11378 | 1621 | 1252590 | 1263968 |  |  |
| i14 | MALPY00411 | 19789 | - | 1265589 | 1287000 |  |  |  |  |  | 777 |  |  |  |  |  |  |  |  |  |
| i14 | PbContig241 | 16842 | - | 1265969 | 1284537 |  |  |  |  |  |  |  |  |  |  |  |  |  |  |  |
| i14 | Pc.c001811713.Contig1 | 1882 | + | 1271552 | 1273443 |  |  |  |  |  |  |  |  |  |  |  |  |  |  |  |
| i14 | Pb.c001202053.Contig1 | 1691 | - | 1285886 | 1287616 | cRMPc10063 | 22027 | 592 | 1265589 | 1287616 |  |  |  |  |  |  |  |  |  |  |
| i14 | MALPY02614 | 3110 | - | 1288208 | 1291291 |  |  |  |  |  | 777 | 292 | 800 |  |  |  |  |  |  |  |
| i14 | Pc.c001708903.Contig1 | 640 | - | 1288539 | 1289182 |  |  |  |  |  |  |  |  |  |  |  |  |  |  |  |
| i14 | Pb.c002007709.Contig1 | 1130 | - | 1288709 | 1289793 | cRMPc10064 | 3083 | 111 | 1288208 | 1291291 |  |  |  |  |  |  |  |  |  |  |
| i14 | Pb.c002006983.Contig1 | 2014 | - | 1291402 | 1293355 |  |  |  |  |  |  |  |  |  |  |  |  |  |  |  |
| iman14 | MALPY00119 - Exp | 11825 | + | 1291429 | 1300625 |  |  |  |  |  |  | 292 |  |  |  |  |  |  |  |  |
| i14 | Pc.c001813139.Contig1 | 607 | - | 1291857 | 1292427 |  |  |  |  |  |  |  |  |  |  |  |  |  |  |  |
| i14 | Pc.c001504222.Contig1 | 4104 | - | 1293405 | 1297470 |  |  |  |  |  |  |  |  |  |  |  |  |  |  |  |
| i14 | Pb.c000801834.Contig1 | 3726 | - | 1293762 | 1297482 |  |  |  |  |  |  |  |  |  |  |  |  |  |  |  |
| i14 | Pc.c001505152.Contig1 | 1438 | - | 1297436 | 1298892 |  |  |  |  |  |  |  |  |  |  |  |  |  |  |  |
| iman14 | PbContig79 - Exp | 5569 | + | 1297631 | 1299392 |  |  |  |  |  |  |  |  |  |  |  |  |  |  |  |
| i14 | Pc.c000803662.Contig1 | 1600 | - | 1298432 | 1300038 | cRMPc10065 | 9223 | 822 | 1291402 | 1300625 |  |  |  |  |  |  |  |  |  |  |
| i14 | Pb.c001402328.Contig1 | 9721 | - | 1301447 | 1311984 |  |  |  |  |  |  |  |  |  |  |  |  |  |  |  |
| i14 | MALPY00044 | 26757 | + | 1302469 | 1331852 |  |  |  |  |  |  | 292 | 1500 |  |  |  |  |  |  |  |
| i14 | Pc.c001103808.Contig1 | 2151 | - | 1305103 | 1307257 |  |  |  |  |  |  |  |  |  |  |  |  |  |  |  |
| i14 | PcContig413 | 9503 | - | 1307750 | 1317843 |  |  |  |  |  |  |  |  |  |  |  |  |  |  |  |
| i14 | PbContig134 | 11859 | - | 1312067 | 1324258 |  |  |  |  |  |  |  |  |  |  |  |  |  |  |  |
| i14 | Pb.c001803419.Contig1 | 3251 | - | 1325522 | 1328717 |  |  |  |  |  |  |  |  |  |  |  |  |  |  |  |
| i14 | PbContig394 | 3720 | - | 1329350 | 1333085 |  |  |  |  |  |  |  |  |  |  |  |  |  |  |  |
| i14 | MALPY01212 | 22131 | - | 1332569 | 1354192 |  |  |  |  |  | 1309 | 292 |  |  |  |  |  |  |  |  |
| i14 | Pc.c001707874.Contig1 | 1358 | + | 1336994 | 1338454 |  |  |  |  |  |  |  |  |  |  |  |  |  |  |  |
| i14 | PbContig250 | 2847 | - | 1337048 | 1339646 |  |  |  |  |  |  |  |  |  |  |  |  |  |  |  |
| i14 | Pb.c001802873.Contig1 | 2235 | - | 1340491 | 1342745 |  |  |  |  |  |  |  |  |  |  |  |  |  |  |  |
| i14 | PcContig185 | 1783 | - | 1340571 | 1342426 |  |  |  |  |  |  |  |  |  |  |  |  |  |  |  |
| i14 | Pc.c001103735.Contig1 | 1804 | - | 1342423 | 1344208 |  |  |  |  |  |  |  |  |  |  |  |  |  |  |  |
| i14 | Pb.c001001633.Contig1 | 5304 | - | 1342771 | 1348422 |  |  |  |  |  |  |  |  |  |  |  |  |  |  |  |
| i14 | Pc.c000303412.Contig1 | 4452 | + | 1348410 | 1352600 |  |  |  |  |  |  |  |  |  |  |  |  |  |  |  |
| i14 | Pb.c000601173.Contig1 | 15829 | + | 1348866 | 1366962 |  |  |  |  |  |  |  |  |  |  |  |  |  |  |  |
| i14 | Pc.c000703430.Contig1 | 1509 | + | 1352778 | 1354275 |  |  |  |  |  |  |  |  |  |  |  |  |  |  |  |
| i14 | MALPY00169 | 23337 | + | 1356373 | 1381682 |  |  |  |  |  | 1309 |  |  |  |  |  |  |  |  |  |
| i14 | Pc.c001203946.Contig1 | 2458 | - | 1364158 | 1366619 |  |  |  |  |  |  |  |  |  |  |  |  |  |  |  |
| i14 | PbContig299 | 15823 | - | 1375747 | 1392242 |  |  |  |  |  |  |  |  |  |  |  |  |  |  |  |
| i14 | Pc.c001404701.Contig1 | 1617 | - | 1376977 | 1379053 |  |  |  |  |  |  |  |  |  |  |  |  |  |  |  |
| i14 | Pc.c000703476.Contig1 | 1707 | - | 1379216 | 1380923 |  |  |  |  |  |  |  |  |  |  |  |  |  |  |  |
| i14 | MALPY03276 | 833 | - | 1379452 | 1380285 |  |  |  |  |  |  | 298 | 1800 |  |  |  |  |  |  |  |
| i14 | MALPY00493 | 14082 | - | 1382061 | 1398588 |  |  |  |  |  | 151 | 298 |  |  |  |  |  |  |  |  |
| i14 | Pc.c000803837.Contig1 | 1443 | - | 1386124 | 1387567 |  |  |  |  |  |  |  |  |  |  |  |  |  |  |  |
| i14 | Pc.c001605851.Contig1 | 1319 | - | 1388006 | 1389322 |  |  |  |  |  |  |  |  |  |  |  |  |  |  |  |
| i14 | Pc.c001404744.Contig1 | 2027 | - | 1389585 | 1391693 |  |  |  |  |  |  |  |  |  |  |  |  |  |  |  |
| i14 | Pb.c001802906.Contig1 | 3997 | - | 1394662 | 1398750 |  |  |  |  |  |  |  |  |  |  |  |  |  |  |  |
| i14 | MALPY02748 | 3421 | - | 1398442 | 1401893 |  |  |  |  |  | 151 |  |  |  |  |  |  |  |  |  |
| i14 | Pb.c000201630.Contig1 | 4736 | - | 1400367 | 1405436 |  |  |  |  |  |  |  |  |  |  |  |  |  |  |  |
| i14 | MALPY01187 | 9289 | + | 1402152 | 1411450 |  |  |  |  |  | 151 | 332 | 2500 |  |  |  |  |  |  |  |
| i14 | Pc.c001606738.Contig1 | 2376 | + | 1402310 | 1404737 | cRMPc10066 | 110003 | 85 | 1301447 | 1411450 |  |  |  |  |  |  |  |  |  |  |
| i14 | MALPY02299 | 6742 | - | 1411535 | 1418469 |  |  |  |  |  | 151 | 251 | 2400 |  |  |  |  |  |  |  |
| i14 | Pb.c000801378.Contig1 | 18084 | + | 1413225 | 1432024 |  |  |  |  |  |  |  |  |  |  |  |  |  |  |  |
| i14 | MALPY03441 | 678 | + | 1414229 | 1414892 |  |  |  |  |  |  | 332 |  |  |  |  |  |  |  |  |
| i14 | Pc.c001304084.Contig1 | 3186 | + | 1414755 | 1418017 |  |  |  |  |  |  |  |  |  |  |  |  |  |  |  |
| i14 | MALPY03208 | 352 | + | 1416967 | 1417322 |  |  |  |  |  |  |  |  |  |  |  |  |  |  |  |
| i14 | Pc.c000503076.Contig1 | 4104 | + | 1417934 | 1422041 |  |  |  |  |  |  |  |  |  |  |  |  |  |  |  |
| i14 | MALPY00963 | 4091 | - | 1418196 | 1422294 |  |  |  |  |  | 151 |  |  |  |  |  |  |  |  |  |
| i14 | MALPY03548 | 301 | - | 1420459 | 1420757 |  |  |  |  |  |  | 251 |  |  |  |  |  |  |  |  |
| i14 | MALPY00363 | 12558 | - | 1421688 | 1435626 |  |  |  |  |  | 151 |  |  |  |  |  |  |  |  |  |
| i14 | PcContig73 | 952 | + | 1427766 | 1428718 |  |  |  |  |  |  |  |  |  |  |  |  |  |  |  |
| i14 | Pb.c000801668.Contig1 | 2812 | + | 1432439 | 1435252 |  |  |  |  |  |  |  |  |  |  |  |  |  |  |  |
| i14 | Pc.c000002956.Contig1 | 1450 | - | 1433429 | 1434876 |  |  |  |  |  |  |  |  |  |  |  |  |  |  |  |
| i14 | Pb.c001602555.Contig1 | 5575 | + | 1435408 | 1441574 |  |  |  |  |  |  |  |  |  |  |  |  |  |  |  |
| i14 | MALPY00453 | 20812 | + | 1435769 | 1458088 |  |  |  |  |  |  | 226 | 4000 |  |  |  |  |  |  |  |
| i14 | PcContig563 | 3226 | - | 1437201 | 1440748 |  |  |  |  |  |  |  |  |  |  |  |  |  |  |  |
| i14 | Pb.c001602553.Contig1 | 1145 | + | 1441755 | 1442939 |  |  |  |  |  |  |  |  |  |  |  |  |  |  |  |
| i14 | Pb.c000501379.Contig1 | 9995 | + | 1445211 | 1456076 |  |  |  |  |  |  |  |  |  |  |  |  |  |  |  |
| i14 | Pc.c000703275.Contig1 | 2452 | + | 1453410 | 1456177 |  |  |  |  |  |  |  |  |  |  |  |  |  |  |  |
| i14 | MALPY03199 | 331 | - | 1453633 | 1453964 |  |  |  |  |  |  |  |  |  |  |  |  |  |  |  |
| i14 | PbContig274 | 4243 | - | 1455825 | 1459936 |  |  |  |  |  |  |  |  |  |  |  |  |  |  |  |
| i14 | Pc.c000002630.Contig1 | 3373 | + | 1456205 | 1459431 | cRMPc10067 | 48401 | 1120 | 1411535 | 1459936 |  |  |  |  |  |  |  |  |  |  |
| i14 | MALPY02287 | 7424 | - | 1461056 | 1468750 |  |  |  |  |  |  | 226 | 4300 |  |  |  |  |  |  |  |
| i14 | Pb.c001101968.Contig1 | 4575 | - | 1463278 | 1468120 | cRMPc10068 | 7694 | 4891 | 1461056 | 1468750 |  |  |  |  |  |  |  |  |  |  |
| iman14 | MALPY02425 - Exp | 9236 | - | 1473641 | 1490394 |  |  |  |  |  |  | 226 | 1600 |  |  |  |  |  |  |  |
| i14 | PcContig109 | 8013 | + | 1474025 | 1482933 |  |  |  |  |  |  |  |  |  |  |  |  |  |  |  |
| i14 | Pb.c000201526.Contig1 | 8109 | + | 1475212 | 1484077 | cRMPc10069 | 16753 | 295 | 1473641 | 1490394 |  |  |  |  |  |  |  |  |  |  |
| i14 | MALPY01239 | 5164 | - | 1490689 | 1495841 |  |  |  |  |  |  | 226 |  |  |  |  |  |  |  |  |
| i14 | Pc.c001003480.Contig1 | 1347 | + | 1491734 | 1493081 |  |  |  |  |  |  |  |  |  |  |  |  |  |  |  |
| i14 | Pb.c001903680.Contig1 | 1809 | - | 1492312 | 1494109 |  |  |  |  |  |  |  |  |  |  |  |  |  |  |  |
| i14 | Pc.c001003670.Contig1 | 1171 | + | 1493054 | 1494228 |  |  |  |  |  |  |  |  |  |  |  |  |  |  |  |
| i14 | MALPY01666 | 8003 | - | 1495709 | 1503732 | cRMPc10070 | 13043 | 270 | 1490689 | 1503732 | 479 | 283 | 1800 |  |  |  |  |  |  |  |
| i14 | MALPY00680 | 5704 | + | 1504002 | 1509788 |  |  |  |  |  | 479 | 326 | 8000 |  |  |  |  |  |  |  |
| i14 | Pb.c000901744.Contig1 | 4147 | - | 1504176 | 1508575 |  |  |  |  |  |  |  |  |  |  |  |  |  |  |  |
| i14 | Pc.c000602335.Contig1 | 7081 | - | 1505153 | 1513524 |  |  |  |  |  |  |  |  |  |  |  |  |  |  |  |
| i14 | MALPY03460 | 687 | + | 1505508 | 1506213 |  |  |  |  |  |  | 283 |  |  |  |  |  |  |  |  |
| i14 | MALPY00282 | 13925 | + | 1510029 | 1523555 |  |  |  |  |  | 479 |  |  |  |  |  |  |  |  |  |
| i14 | Pb.c001201975.Contig1 | 2398 | + | 1510987 | 1513489 |  |  |  |  |  |  |  |  |  |  |  |  |  |  |  |
| i14 | PbContig220 | 8793 | + | 1513565 | 1522004 |  |  |  |  |  |  |  |  |  |  |  |  |  |  |  |
| i14 | MALPY03295 | 1771 | + | 1515948 | 1517716 |  |  |  |  |  |  | 326 | 1300 |  |  |  |  |  |  |  |
| i14 | Pc.c000703295.Contig1 | 2053 | + | 1517593 | 1519637 |  |  |  |  |  |  |  |  |  |  |  |  |  |  |  |
| iJC14 | MALPY03470 | 791 | + | 1518423 | 1519205 |  |  |  |  |  |  | 326 |  |  |  |  |  |  |  |  |
| i14 | Pb.c000001624.Contig1 | 5445 | - | 1523235 | 1528418 |  |  |  |  |  |  |  |  |  |  |  |  |  |  |  |
| i14 | MALPY02185 | 9263 | - | 1523912 | 1533324 |  |  |  |  |  |  | 434 | 1500 |  |  |  |  |  |  |  |
| i14 | Pc.c001003305.Contig1 | 4776 | + | 1524066 | 1528573 |  |  |  |  |  |  |  |  |  |  |  |  |  |  |  |
| i14 | Pc.c000402792.Contig1 | 3507 | + | 1529114 | 1533301 |  |  |  |  |  |  |  |  |  |  |  |  |  |  |  |
| i14 | Pb.c000301411.Contig1 | 8514 | - | 1532642 | 1541772 |  |  |  |  |  |  |  |  |  |  |  |  |  |  |  |
| i14 | PcContig450 | 3904 | + | 1533974 | 1537927 |  |  |  |  |  |  |  |  |  |  |  |  |  |  |  |
| i14 | MALPY02510 | 5025 | - | 1534388 | 1539499 |  |  |  |  |  | 555 | 434 |  |  |  |  |  |  |  |  |
| i14 | Pc.c001504929.Contig1 | 3221 | - | 1538254 | 1541459 |  |  |  |  |  |  |  |  |  |  |  |  |  |  |  |
| i14 | MALPY00920 | 10625 | - | 1539799 | 1550412 |  |  |  |  |  | 555 |  |  |  |  |  |  |  |  |  |
| i14 | Pc.c001707834.Contig1 | 2362 | - | 1542800 | 1545204 |  |  |  |  |  |  |  |  |  |  |  |  |  |  |  |
| i14 | Pb.c000801682.Contig1 | 3110 | - | 1546544 | 1550053 |  |  |  |  |  |  |  |  |  |  |  |  |  |  |  |
| i14 | Pc.c001304698.Contig1 | 892 | + | 1548562 | 1549565 |  |  |  |  |  |  |  |  |  |  |  |  |  |  |  |
| i14 | Pc.c000902727.Contig1 | 3658 | + | 1549994 | 1553820 |  |  |  |  |  |  |  |  |  |  |  |  |  |  |  |
| i14 | MALPY01151 | 2076 | - | 1551175 | 1553248 |  |  |  |  |  | 20 |  |  |  |  |  |  |  |  |  |
| i14 | Pb.c002007832.Contig1 | 1005 | + | 1553052 | 1553994 |  |  |  |  |  |  |  |  |  |  |  |  |  |  |  |
| i14 | MALPY01235 | 9126 | - | 1553142 | 1564097 |  |  |  |  |  | 20 |  |  |  |  |  |  |  |  |  |
| i14 | PcContig590 | 2716 | + | 1558621 | 1561448 |  |  |  |  |  |  |  |  |  |  |  |  |  |  |  |
| i14 | Pb.c000902050.Contig1 | 1619 | - | 1560270 | 1561889 |  |  |  |  |  |  |  |  |  |  |  |  |  |  |  |
| i14 | Pc.c001505375.Contig1 | 831 | + | 1561947 | 1562778 | cRMPc10071 | 60095 | 411 | 1504002 | 1564097 |  |  |  | cRMPs1020 | 298508 | 411 | 1265589 | 1564097 |  |  |
| i14 | MALPY01672 | 8324 | + | 1564508 | 1573427 |  |  |  |  |  |  | 299 | 3800 |  |  |  |  |  |  |  |
| i14 | PcContig116 | 2103 | - | 1567611 | 1569717 |  |  |  |  |  |  |  |  |  |  |  |  |  |  |  |
| i14 | Pb.c001101838.Contig1 | 2273 | - | 1569131 | 1571464 | cRMPc10072 | 8919 | 423 | 1564508 | 1573427 |  |  |  |  |  |  |  |  |  |  |
| iman14 | MALPY02469 | 3140 | - | 1573850 | 1577503 | cRMPc10073 | 3653 | 261 | 1573850 | 1577503 | 142 |  |  |  |  |  |  |  |  |  |
| i14 | MALPY01079 | 9661 | + | 1577764 | 1587360 |  |  |  |  |  | 142 | 299 |  |  |  |  |  |  |  |  |
| i14 | Pb.c002113780.Contig1 | 854 | - | 1577789 | 1578643 |  |  |  |  |  |  |  |  |  |  |  |  |  |  |  |
| i14 | Pc.c000302880.Contig1 | 1345 | - | 1578414 | 1579759 |  |  |  |  |  |  |  |  |  |  |  |  |  |  |  |
| i14 | Pc.c000203281.Contig1 | 638 | + | 1578954 | 1579592 |  |  |  |  |  |  |  |  |  |  |  |  |  |  |  |
| i14 | Pb.c000601547.Contig1 | 4471 | - | 1579018 | 1583261 |  |  |  |  |  |  |  |  |  |  |  |  |  |  |  |
| i14 | Pc.c000303011.Contig1 | 1212 | + | 1580144 | 1581356 |  |  |  |  |  |  |  |  |  |  |  |  |  |  |  |
| i14 | PcContig97 | 6749 | - | 1580797 | 1587576 |  |  |  |  |  |  |  |  |  |  |  |  |  |  |  |
| i14 | Pb.c000902097.Contig1 | 935 | - | 1583621 | 1584712 |  |  |  |  |  |  |  |  |  |  |  |  |  |  |  |
| i14 | Pb.c000901855.Contig1 | 7178 | - | 1584680 | 1592331 |  |  |  |  |  |  |  |  |  |  |  |  |  |  |  |
| i14 | MALPY02518 | 7748 | - | 1587888 | 1595453 |  |  |  |  |  | 142 |  |  |  |  |  |  |  |  |  |
| i14 | Pc.c000102464.Contig1 | 3160 | + | 1589992 | 1593138 | cRMPc10074 | 17689 | 593 | 1577764 | 1595453 |  |  |  |  |  |  |  |  |  |  |
| i14 | MALPY01799 | 4463 | + | 1596046 | 1600520 |  |  |  |  |  | 142 | 87 | 2000 |  |  |  |  |  |  |  |
| i14 | Pc.c000803859.Contig1 | 644 | + | 1596202 | 1596846 |  |  |  |  |  |  |  |  |  |  |  |  |  |  |  |
| i14 | Pc.c001204523.Contig1 | 2233 | + | 1596934 | 1599167 |  |  |  |  |  |  |  |  |  |  |  |  |  |  |  |
| i14 | Pb.c001301886.Contig1 | 2502 | - | 1597794 | 1600302 | cRMPc10075 | 4474 | 509 | 1596046 | 1600520 |  |  |  |  |  |  |  |  |  |  |
| iJC14 | MALPY02470 | 2104 | xxx | 1600000 |  |  |  |  |  |  | 142 |  |  |  |  |  |  |  |  |  |
| i14 | MALPY03022 | 1875 | - | 1601029 | 1602904 | cRMPc10076 | 1875 | 6501 | 1601029 | 1602904 |  | 87 |  | cRMPs1021 | 38396 | 6501 | 1564508 | 1602904 |  |  |
| i14 | MALPY02350 | 2794 | + | 1609405 | 1612178 |  |  |  |  |  | 385 |  |  |  |  |  |  |  |  |  |
| i14 | Pb.c000902056.Contig1 | 1862 | - | 1609679 | 1611544 |  |  |  |  |  |  |  |  |  |  |  |  |  |  |  |
| i14 | MALPY00269 | 10338 | + | 1612136 | 1622607 |  |  |  |  |  | 385 |  |  |  |  |  |  |  |  |  |
| i14 | PbContig335 | 17875 | - | 1612376 | 1630550 |  |  |  |  |  |  |  |  |  |  |  |  |  |  |  |
| i14 | Pc.c000102609.Contig1 | 3166 | - | 1615224 | 1618271 |  |  |  |  |  |  |  |  |  |  |  |  |  |  |  |
| i14 | Pc.c000102975.Contig1 | 692 | - | 1618479 | 1619171 |  |  |  |  |  |  |  |  |  |  |  |  |  |  |  |
| i14 | Pc.c000102615.Contig1 | 10748 | - | 1619222 | 1630624 |  |  |  |  |  |  |  |  |  |  |  |  |  |  |  |
| i14 | MALPY01726 | 7684 | + | 1623216 | 1630764 | cRMPc10077 | 21359 | 500 | 1609405 | 1630764 | 50B - Ins |  |  |  |  |  |  |  |  |  |
| iJC14 | MALPY00217 | 7745 | + | 1631264 | 1638982 |  |  |  |  |  | 50B - Ins |  |  |  |  |  |  |  |  |  |
| i14 | Pb.c001001795.Contig1 | 4821 | - | 1631503 | 1636327 | cRMPc10078 | 7718 | 105 | 1631264 | 1638982 |  |  |  |  |  |  |  |  |  |  |
| i14 | MALPY02298 | 3736 | - | 1639087 | 1642823 |  |  |  |  |  | 50B |  |  |  |  |  |  |  |  |  |
| i14 | Pb.c001402055.Contig1 | 14488 | - | 1642044 | 1656198 |  |  |  |  |  |  |  |  |  |  |  |  |  |  |  |
| i14 | MALPY01669 | 4507 | - | 1642328 | 1647057 |  |  |  |  |  | 50B |  |  |  |  |  |  |  |  |  |
| i14 | MALPY00036 | 51480 | - | 1646738 | 1702081 |  |  |  |  |  | 50B |  |  |  |  |  |  |  |  |  |
| i14 | Pc.c001606306.Contig1 | 638 | + | 1655909 | 1656529 |  |  |  |  |  |  |  |  |  |  |  |  |  |  |  |
| i14 | Pb.c001402193.Contig1 | 5937 | + | 1656352 | 1662388 |  |  |  |  |  |  |  |  |  |  |  |  |  |  |  |
| i14 | Pc.c001606702.Contig1 | 1282 | - | 1657570 | 1658990 |  |  |  |  |  |  |  |  |  |  |  |  |  |  |  |
| i14 | PbContig401 | 17300 | - | 1667513 | 1685605 |  |  |  |  |  |  |  |  |  |  |  |  |  |  |  |
| i14 | PcContig158 | 4508 | + | 1672643 | 1677089 |  |  |  |  |  |  |  |  |  |  |  |  |  |  |  |
| i14 | Pb.c001904440.Contig1 | 3190 | + | 1685687 | 1688837 |  |  |  |  |  |  |  |  |  |  |  |  |  |  |  |
| i14 | Pc.c001707749.Contig1 | 2408 | - | 1686026 | 1688259 |  |  |  |  |  |  |  |  |  |  |  |  |  |  |  |
| i14 | Pc.c001104236.Contig1 | 1333 | - | 1687959 | 1689286 |  |  |  |  |  |  |  |  |  |  |  |  |  |  |  |
| i14 | Pb.c001101982.Contig1 | 6586 | + | 1688634 | 1695217 |  |  |  |  |  |  |  |  |  |  |  |  |  |  |  |
| i14 | Pb.c001702803.Contig1 | 2341 | + | 1698886 | 1701221 |  |  |  |  |  |  |  |  |  |  |  |  |  |  |  |
| i14 | Pc.c000603484.Contig1 | 730 | + | 1700080 | 1700810 |  |  |  |  |  |  |  |  |  |  |  |  |  |  |  |
| iman14 | PbContig4844 | 6451 | - | 1701671 | 1708122 | cRMPc10079 | 69035 |  | 1639087 | 1708122 |  |  |  | cRMPs1022 | 98717 |  | 1609405 | 1708122 | MALPY00036 |  |
| Telomere | |  |  |  |  |  |  |  |  |  |  |  |  |  |  |  |  |  |  |  |
|  |  | Pb Pc Py contig |  |  |  |  | cRMP contig | Gap to next cRMP contig |  |  |  |  |  |  | cRMP scaffold | Gap to next cRMP scaffold |  |  |  |  |
|  | Number | 594 |  |  |  | Number | 79 | 78 |  |  |  |  |  | Number | 22 | 21 |  |  |  |  |
|  | Average size (bp) | 5358 |  |  |  | Average size (bp) | 19808 | 1837 |  |  |  |  |  | Average size (bp) | 75472 | 2273 |  |  |  |  |
|  | Minimum size (bp) | 199 |  |  |  | Minimum size (bp) | 628 | 5 |  |  |  |  |  | Minimum size (bp) | 1862 | 25 |  |  |  |  |
|  | Maximum size (bp) | 51480 |  |  |  | Maximum size (bp) | 124717 | 13054 |  |  |  |  |  | Maximum size (bp) | 373564 | 13054 |  |  |  |  |
|  | Total size (bp) | 3182760 |  |  |  | Total size (bp) | 1564860 | 143262 |  |  |  |  |  | Total size (bp) | 1660379 | 47743 |  |  |  |  |
|  |  |  |  |  |  |  |  |  |  |  |  |  |  |  |  |  |  |  |  |  |
