# Supplementary material for: A Plasmodium Whole-Genome Synteny Map: Indels and Synteny Breakpoints as Foci for Species-Specific Genes
Source: PLoS Pathog. 2005 Dec 23;1(4):e44. doi: 10.1371/journal.ppat.0010044 (PMC1317653; doi:10.1371/journal.ppat.0010044)
Supplement: Supplementary file 11 [file ppat.0010044.st027.doc]

Table S27 - RMPchr11


# Table S27 - RMPchr11

|  |  |  |  |  |  |  |  |  |  |  |  |  |  |  |  |  |  |  |  |
| --- | --- | --- | --- | --- | --- | --- | --- | --- | --- | --- | --- | --- | --- | --- | --- | --- | --- | --- | --- |
| Table S27: Tiling path of RMP contigs combined to form cRMP chromosome 11. | | | | | | |  |  |  |  |  |  |  |  |  |  |  |  |  |
|  |  |  |  |  |  |  |  |  |  |  |  |  |  |  |  |  |  |  |  |
| Tiling paths of P. berghei (Pb), P. c. chabaudi (Pc) and P. y. yoelii (Py) contigs combined into composite rodent malaria parasite (cRMP) | | | | | | | | | |  |  |  |  |  |  |  |  |  |  |
| contigs aligned against parts of the 14 chromosomes of P. falciparum (Pf). The cRMP contigs are further linked by physical linkage | | | | | | | | | |  |  |  |  |  |  |  |  |  |  |
| and PCR (see Methods) to form cRMP scaffolds. Different synteny blocks with homology to different P. falciparum chromosomes | | | | | | | | | |  |  |  |  |  |  |  |  |  |  |
| together form the cRMP chromosomes. The tables also show gaps in the alignment (black lines), cRMP telomeres (white bars) and | | | | | | | | | |  |  |  |  |  |  |  |  |  |  |
| predicted centromeres (plum/black bars). Contigs, physical groups or PCR groups interrupted by inter- or intrasyntenic regions are | | | | | | | | | |  |  |  |  |  |  |  |  |  |  |
| printed in bold. A summary of the number of RMP contigs, cRMP contigs and cRMP scaffolds as well as their average, minimum, | | | | | | | | | |  |  |  |  |  |  |  |  |  |  |
| maximum and total sizes is provided. Column headings are as follows: | | | | |  |  |  |  |  |  |  |  |  |  |  |  |  |  |  |
|  |  |  |  |  |  |  |  |  |  |  |  |  |  |  |  |  |  |  |  |
| Pf chr.: P. falciparum chromosome to which the RMP contigs are aligned | | | | | |  |  |  |  |  |  |  |  |  |  |  |  |  |  |
| (#: PROmer analysis� from this study; JC# and TK#: Carlton et al.; man#: manual BLAST analysis; | | | | | | |  |  |  |  |  |  |  |  |  |  |  |  |  |
| i: inversed orientation of the synteny block; colours refer to those used in Fig. 2 of the paper); | | | | | | |  |  |  |  |  |  |  |  |  |  |  |  |  |
| Pb Pc Py contig: names of the aligned RMP contigs; | | |  |  |  |  |  |  |  |  |  |  |  |  |  |  |  |  |  |
| Size: size (bp) of the aligned RMP contigs; | |  |  |  |  |  |  |  |  |  |  |  |  |  |  |  |  |  |  |
| +/-: orientation of the aligned RMP contigs; | |  |  |  |  |  |  |  |  |  |  |  |  |  |  |  |  |  |  |
| Start contig & End contig: estimated start & end coordinates of alignment of the RMP contigs based on the coordinates | | | | | | | | |  |  |  |  |  |  |  |  |  |  |  |
| of PROmer hits corrected for the contig�s size and orientation and presence of inter- and intrasyntenic regions | | | | | | | |  |  |  |  |  |  |  |  |  |  |  |  |
| (the start position of the first RMP contig in the alignment was set at zero); | | | | | |  |  |  |  |  |  |  |  |  |  |  |  |  |  |
| cRMP contig: names of cRMP contigs | |  |  |  |  |  |  |  |  |  |  |  |  |  |  |  |  |  |  |
| (the first 2 digits refer to the cRMP chromosome number and the following 3 digits designate the order of the contigs); | | | | | | | | |  |  |  |  |  |  |  |  |  |  |  |
| cRMP contig size: size of cRMP contigs based on the corrected coordinates of alignment; | | | | | | |  |  |  |  |  |  |  |  |  |  |  |  |  |
| Gap size to next cRMP contig: size of gaps between neighbouring cRMP contigs; | | | | | | |  |  |  |  |  |  |  |  |  |  |  |  |  |
| Start cRMP contig & End cRMP contig: estimated start & end coordinates of alignment of the cRMP contigs; | | | | | | | |  |  |  |  |  |  |  |  |  |  |  |  |
| Phys. group (Py): physical linkage of neighbouring P. y. yoelii contigs; | | | | | |  |  |  |  |  |  |  |  |  |  |  |  |  |  |
| PCR group (Py): neighbouring P. y. yoelii contigs were linked previously or for this study by PCR; | | | | | | |  |  |  |  |  |  |  |  |  |  |  |  |  |
| PCR size (Py): size of the PCR product; | |  |  |  |  |  |  |  |  |  |  |  |  |  |  |  |  |  |  |
| cRMP scaffold: names of cRMP scaffolds | |  |  |  |  |  |  |  |  |  |  |  |  |  |  |  |  |  |  |
| (the first 2 digits refer to the cRMP chromosome number and the following 2 digits designate the order of the scaffolds); | | | | | | | | |  |  |  |  |  |  |  |  |  |  |  |
| cRMP scaffold size: size of cRMP scaffolds; | | |  |  |  |  |  |  |  |  |  |  |  |  |  |  |  |  |  |
| Gap size to next cRMP scaffold: size of gaps between neighbouring cRMP scaffolds; | | | | | | |  |  |  |  |  |  |  |  |  |  |  |  |  |
| Start cRMP scaffold & End cRMP scaffold: estimated start & end coordinates of alignment of the cRMP scaffolds; | | | | | | | | |  |  |  |  |  |  |  |  |  |  |  |
| Overlap: Linkage between RMP contigs established through manual BLAST analysis. | | | | | | |  |  |  |  |  |  |  |  |  |  |  |  |  |
|  |  |  |  |  |  |  |  |  |  |  |  |  |  |  |  |  |  |  |  |
| Pf chr. | Pb Pc Py contig | Size | +/- | Start contig | End contig | cRMP contig | cRMP contig size | Gap size to next cRMP contig | Start cRMP contig | End cRMP contig | Phys. group (Py) | PCR group (Py) | PCR size (Py) | cRMP scaffold | cRMP scaffold size | Gap size to next cRMP scaffold | Start cRMP scaffold | End cRMP scaffold | Overlap (manual) |
| Telomere | |  |  |  |  |  |  |  |  |  |  |  |  |  |  |  |  |  |  |
| man5 | PcContig4630 | 1475 | + | 0 | 1475 |  |  |  |  |  |  |  |  |  |  |  |  |  |  |
| man5 | MALPY01171 - Exp | 14670 | + | 525 | 16545 |  |  |  |  |  |  |  |  |  |  |  |  |  |  |
| 5 | Pc.c000402913.Contig1 | 4481 | + | 10903 | 15945 |  |  |  |  |  |  |  |  |  |  |  |  |  |  |
| 5 | Pb.c000501395.Contig1 | 5387 | + | 12647 | 18499 |  |  |  |  |  |  |  |  |  |  |  |  |  |  |
| 5 | MALPY03321 | 820 | + | 13725 | 14746 |  |  |  |  |  |  | 20 | 3000 |  |  |  |  |  |  |
| 5 | MALPY01328 | 7132 | - | 18260 | 25384 |  |  |  |  |  | 570 | 20 |  |  |  |  |  |  |  |
| 5 | MALPY01901 | 4899 | + | 24762 | 29652 |  |  |  |  |  | 570 | 243 - Ins | 2000 |  |  |  |  |  |  |
| 5 | Pb.c001602426.Contig1 | 1909 | - | 26195 | 28143 |  |  |  |  |  |  |  |  |  |  |  |  |  |  |
| 5 | Pc.c001812834.Contig1 | 944 | - | 26789 | 27733 | cRMPc11001 | 29652 | 516 | 0 | 29652 |  |  |  |  |  |  |  |  |  |
| TK5 | MALPY02177 | 2338 | - | 30168 | 32481 |  |  |  |  |  | 570 |  |  |  |  |  |  |  |  |
| 5 | Pb.c001502094.Contig2 | 6626 | + | 31610 | 39028 |  |  |  |  |  |  |  |  |  |  |  |  |  |  |
| 5 | MALPY02001 | 2103 | - | 31652 | 33928 |  |  |  |  |  | 551 | 243 - Ins |  |  |  |  |  |  |  |
| 5 | PcContig337 | 1016 | + | 31965 | 33119 |  |  |  |  |  |  |  |  |  |  |  |  |  |  |
| 5 | MALPY01432 | 6993 | + | 34015 | 41307 |  |  |  |  |  | 551 | 112 | 1000 |  |  |  |  |  |  |
| 5 | Pb.c001502481.Contig1 | 3837 | - | 41270 | 45315 |  |  |  |  |  |  |  |  |  |  |  |  |  |  |
| 5 | MALPY02289 | 4612 | + | 41293 | 46105 |  |  |  |  |  | 170 | 112 |  |  |  |  |  |  |  |
| TK5 | MALPY02353 | 2632 | - | 44717 | 47346 |  |  |  |  |  | 170 |  |  |  |  |  |  |  |  |
| 5 | Pb.c001301827.Contig1 | 1811 | - | 46666 | 48435 |  |  |  |  |  |  |  |  |  |  |  |  |  |  |
| 5 | MALPY01446 | 6619 | + | 47160 | 55631 |  |  |  |  |  | 170 |  |  |  |  |  |  |  |  |
| 5 | Pb.c000801754.Contig1 | 2234 | - | 51987 | 54428 |  |  |  |  |  |  |  |  |  |  |  |  |  |  |
| 5 | Pc.c000102620.Contig1 | 4136 | + | 54016 | 58146 |  |  |  |  |  |  |  |  |  |  |  |  |  |  |
| 5 | Pb.c001602557.Contig1 | 1656 | - | 55940 | 57590 |  |  |  |  |  |  |  |  |  |  |  |  |  |  |
| 5 | MALPY00099 | 10193 | - | 56784 | 67465 |  |  |  |  |  |  | 277 | 1400 |  |  |  |  |  |  |
| 5 | Pb.c001702295.Contig1 | 6398 | - | 57613 | 64488 |  |  |  |  |  |  |  |  |  |  |  |  |  |  |
| 5 | PcContig366 | 5086 | + | 58194 | 63697 |  |  |  |  |  |  |  |  |  |  |  |  |  |  |
| 5 | PcContig605 | 4150 | - | 65217 | 69862 |  |  |  |  |  |  |  |  |  |  |  |  |  |  |
| 5 | Pb.c000101608.Contig1 | 1703 | + | 65780 | 67441 |  |  |  |  |  |  |  |  |  |  |  |  |  |  |
| 5 | MALPY01217 | 8625 | + | 69670 | 79435 |  |  |  |  |  |  | 277 |  |  |  |  |  |  |  |
| 5 | PcContig152 | 1672 | - | 70315 | 72328 |  |  |  |  |  |  |  |  |  |  |  |  |  |  |
| 5 | Pb.c002007601.Contig1 | 646 | + | 71892 | 72562 |  |  |  |  |  |  |  |  |  |  |  |  |  |  |
| 5 | PcContig121 | 3703 | + | 73554 | 77503 |  |  |  |  |  |  |  |  |  |  |  |  |  |  |
| 5 | Pb.c000701566.Contig1 | 3788 | + | 74090 | 78166 | cRMPc11002 | 49267 | 3693 | 30168 | 79435 |  |  |  | cRMPs1101 | 79435 | 3693 | 0 | 79435 |  |
| 5 | MALPY01142 | 16974 | + | 83128 | 99896 |  |  |  |  |  |  |  |  |  |  |  |  |  |  |
| 5 | Pb.c000101338.Contig1 | 1711 | + | 84064 | 85832 |  |  |  |  |  |  |  |  |  |  |  |  |  |  |
| 5 | PcContig657 | 5311 | + | 86321 | 91809 |  |  |  |  |  |  |  |  |  |  |  |  |  |  |
| 5 | Pb.c000901820.Contig1 | 4583 | + | 88248 | 92829 |  |  |  |  |  |  |  |  |  |  |  |  |  |  |
| 5 | MALPY03420 | 828 | - | 88961 | 89774 | cRMPc11003 | 16768 | 8021 | 83128 | 99896 |  |  |  | cRMPs1102 | 16768 | 8021 | 83128 | 99896 |  |
| JC5 | MALPY00781 | 7963 | - | 107917 | 115880 | cRMPc11004 | 7963 | 4475 | 107917 | 115880 | 703 |  |  |  |  |  |  |  |  |
| 5 | MALPY01515 | 10184 | + | 120355 | 131043 |  |  |  |  |  | 703 |  |  |  |  |  |  |  |  |
| 5 | Pc.c000302832.Contig1 | 6687 | - | 125138 | 132312 |  |  |  |  |  |  |  |  |  |  |  |  |  |  |
| 5 | Pb.c000301405.Contig1 | 3348 | - | 127145 | 131006 | cRMPc11005 | 11957 | 293 | 120355 | 132312 |  |  |  | cRMPs1103 | 24395 | 293 | 107917 | 132312 |  |
| 5 | MALPY00604 | 6393 | + | 132605 | 139556 |  |  |  |  |  |  |  |  |  |  |  |  |  |  |
| 5 | Pb.c001502523.Contig1 | 2006 | - | 133018 | 135444 |  |  |  |  |  |  |  |  |  |  |  |  |  |  |
| 5 | Pc.c001003489.Contig1 | 1858 | - | 133797 | 135646 |  |  |  |  |  |  |  |  |  |  |  |  |  |  |
| 5 | Pb.c001904880.Contig1 | 611 | - | 136071 | 136684 |  |  |  |  |  |  |  |  |  |  |  |  |  |  |
| 5 | Pc.c001104305.Contig1 | 467 | - | 136134 | 136604 | cRMPc11006 | 6951 | 785 | 132605 | 139556 |  |  |  | cRMPs1104 | 6951 | 785 | 132605 | 139556 |  |
| 5 | Pc.c000102029.Contig2 | 2611 | - | 140341 | 142937 |  |  |  |  |  |  |  |  |  |  |  |  |  |  |
| 5 | MALPY00235 | 3944 | - | 140975 | 144945 |  |  |  |  |  |  |  |  |  |  |  |  |  |  |
| 5 | Pb.c001904423.Contig1 | 1388 | + | 141696 | 143075 |  |  |  |  |  |  |  |  |  |  |  |  |  |  |
| 5 | Pc.c000302890.Contig1 | 1777 | - | 143126 | 144879 |  |  |  |  |  |  |  |  |  |  |  |  |  |  |
| 5 | Pb.c000301398.Contig1 | 2244 | + | 143459 | 145709 | cRMPc11007 | 5368 | 387 | 140341 | 145709 |  |  |  | cRMPs1105 | 5368 | 387 | 140341 | 145709 |  |
| 5 | MALPY00831 | 8866 | + | 146096 | 155420 |  |  |  |  |  |  |  |  |  |  |  |  |  |  |
| 5 | Pb.c000301399.Contig1 | 3769 | + | 146155 | 150032 |  |  |  |  |  |  |  |  |  |  |  |  |  |  |
| 5 | Pc.c000002685.Contig1 | 4025 | - | 146285 | 150430 |  |  |  |  |  |  |  |  |  |  |  |  |  |  |
| 5 | PbContig326 | 6742 | - | 150507 | 157651 |  |  |  |  |  |  |  |  |  |  |  |  |  |  |
| 5 | PcContig538 | 9244 | - | 150553 | 160637 |  |  |  |  |  |  |  |  |  |  |  |  |  |  |
| 5 | Pb.c001803539.Contig1 | 2005 | - | 157819 | 159887 | cRMPc11008 | 14541 | 281 | 146096 | 160637 |  |  |  | cRMPs1106 | 14541 | 281 | 146096 | 160637 |  |
| 5 | Pc.c000903457.Contig1 | 3038 | - | 160918 | 163956 |  |  |  |  |  |  |  |  |  |  |  |  |  |  |
| 5 | Pb.c001402357.Contig1 | 859 | - | 163066 | 163925 |  |  |  |  |  |  |  |  |  |  |  |  |  |  |
| 5 | MALPY03411 | 587 | + | 163212 | 163799 |  |  |  |  |  |  | 215 | 500 |  |  |  |  |  |  |
| 5 | MALPY03067 | 776 | + | 163901 | 164674 |  |  |  |  |  |  | 164/215 | 800 |  |  |  |  |  |  |
| 5 | Pb.c001402105.Contig1 | 3430 | - | 163928 | 167361 |  |  |  |  |  |  |  |  |  |  |  |  |  |  |
| 5 | MALPY00194 | 9590 | + | 165531 | 175083 |  |  |  |  |  |  | 164 | 6500 |  |  |  |  |  |  |
| 5 | PbContig172 | 8617 | - | 169349 | 178105 |  |  |  |  |  |  |  |  |  |  |  |  |  |  |
| 5 | Pc.c001708751.Contig1 | 1134 | - | 172279 | 173275 |  |  |  |  |  |  |  |  |  |  |  |  |  |  |
| man5 | MALPY00894 | 3928 | - | 177174 | 180331 | cRMPc11009 | 19413 | 3449 | 160918 | 180331 | 138 |  |  |  |  |  |  |  |  |
| 5 | MALPY02100 | 10817 | - | 183780 | 194994 |  |  |  |  |  | 138 | 164 |  |  |  |  |  |  |  |
| 5 | PcContig645 | 2129 | - | 185001 | 187193 |  |  |  |  |  |  |  |  |  |  |  |  |  |  |
| 5 | MALPY03309 | 1343 | - | 186382 | 187698 |  |  |  |  |  |  | 200 | 7500 |  |  |  |  |  |  |
| 5 | Pb.c001202056.Contig1 | 4209 | + | 186676 | 190969 |  |  |  |  |  |  |  |  |  |  |  |  |  |  |
| 5 | Pb.c000401527.Contig1 | 3506 | + | 193668 | 197791 |  |  |  |  |  |  |  |  |  |  |  |  |  |  |
| 5 | MALPY01318 | 7759 | - | 195445 | 203423 |  |  |  |  |  | 138 | 200 |  |  |  |  |  |  |  |
| 5 | Pc.c000203299.Contig1 | 815 | + | 196699 | 197532 |  |  |  |  |  |  |  |  |  |  |  |  |  |  |
| 5 | Pc.c000203300.Contig1 | 1247 | + | 197626 | 198891 |  |  |  |  |  |  |  |  |  |  |  |  |  |  |
| 5 | PbContig369 | 5669 | + | 197891 | 203858 |  |  |  |  |  |  |  |  |  |  |  |  |  |  |
| 5 | Pc.c001708232.Contig1 | 729 | - | 200079 | 200811 |  |  |  |  |  |  |  |  |  |  |  |  |  |  |
| 5 | Pc.c000402732.Contig1 | 6674 | - | 201723 | 209668 |  |  |  |  |  |  |  |  |  |  |  |  |  |  |
| 5 | MALPY02088 | 3941 | - | 203935 | 207876 |  |  |  |  |  | 138 | 98 | 4500 |  |  |  |  |  |  |
| 5 | Pb.c001502489.Contig1 | 3802 | + | 205191 | 208993 |  |  |  |  |  |  |  |  |  |  |  |  |  |  |
| 5 | Pb.c001402136.Contig1 | 3915 | + | 209284 | 213196 |  |  |  |  |  |  |  |  |  |  |  |  |  |  |
| 5 | PbContig114 | 1702 | - | 209747 | 211686 |  |  |  |  |  |  |  |  |  |  |  |  |  |  |
| 5 | PcContig16 | 9493 | - | 210677 | 221152 |  |  |  |  |  |  |  |  |  |  |  |  |  |  |
| 5 | PbContig323 | 9125 | - | 213415 | 222549 |  |  |  |  |  |  |  |  |  |  |  |  |  |  |
| 5 | MALPY01310 | 8383 | - | 213495 | 221863 | cRMPc11010 | 38769 | 1169 | 183780 | 222549 |  | 98 |  | cRMPs1107 | 61631 | 1169 | 160918 | 222549 |  |
| 5 | MALPY01611 | 7668 | - | 223718 | 231986 |  |  |  |  |  |  |  |  |  |  |  |  |  |  |
| 5 | Pb.c001202027.Contig1 | 6092 | - | 224211 | 230595 |  |  |  |  |  |  |  |  |  |  |  |  |  |  |
| 5 | Pc.c001812914.Contig1 | 856 | - | 224825 | 225624 |  |  |  |  |  |  |  |  |  |  |  |  |  |  |
| 5 | Pc.c000802694.Contig2 | 6298 | - | 227056 | 233345 | cRMPc11011 | 9627 | 746 | 223718 | 233345 |  |  |  | cRMPs1108 | 9627 | 746 | 223718 | 233345 |  |
| 5 | Pb.c001001700.Contig1 | 5691 | + | 234091 | 239776 |  |  |  |  |  |  |  |  |  |  |  |  |  |  |
| 5 | MALPY00761 | 16664 | + | 234610 | 251869 |  |  |  |  |  | 305 |  |  |  |  |  |  |  |  |
| 5 | PcContig360 | 5019 | - | 235626 | 240645 |  |  |  |  |  |  |  |  |  |  |  |  |  |  |
| 5 | MALPY03318 | 842 | + | 237551 | 238392 |  |  |  |  |  |  |  |  |  |  |  |  |  |  |
| 5 | Pb.c000501173.Contig2 | 9187 | - | 241727 | 251372 |  |  |  |  |  |  |  |  |  |  |  |  |  |  |
| 5 | Pc.c000002678.Contig1 | 3676 | - | 242021 | 245707 |  |  |  |  |  |  |  |  |  |  |  |  |  |  |
| 5 | PcContig149 | 8955 | + | 246429 | 256970 |  |  |  |  |  |  |  |  |  |  |  |  |  |  |
| 5 | MALPY00109 | 7610 | - | 252187 | 260085 |  |  |  |  |  | 305 |  |  |  |  |  |  |  |  |
| 5 | Pb.c000701219.Contig1 | 5595 | - | 253803 | 259967 |  |  |  |  |  |  |  |  |  |  |  |  |  |  |
| 5 | MALPY03166 | 266 | + | 254616 | 254882 | cRMPc11012 | 25994 | 297 | 234091 | 260085 |  | 203 | 5000 |  |  |  |  |  |  |
| 5 | Pc.c001504954.Contig1 | 1441 | - | 260382 | 261802 |  |  |  |  |  |  |  |  |  |  |  |  |  |  |
| 5 | MALPY02442 | 2432 | - | 261131 | 263608 |  |  |  |  |  | 973 | 203 |  |  |  |  |  |  |  |
| 5 | Pc.c001505204.Contig1 | 956 | - | 262270 | 263262 | cRMPc11013 | 3226 | 26 | 260382 | 263608 |  |  |  |  |  |  |  |  |  |
| 5 | MALPY02540 | 5578 | - | 263634 | 269171 |  |  |  |  |  | 973 |  |  |  |  |  |  |  |  |
| 5 | Pc.c001708595.Contig1 | 806 | - | 264665 | 265468 |  |  |  |  |  |  |  |  |  |  |  |  |  |  |
| 5 | Pb.c000301336.Contig1 | 11976 | + | 264913 | 277237 |  |  |  |  |  |  |  |  |  |  |  |  |  |  |
| 5 | Pc.c000502977.Contig1 | 6053 | - | 266051 | 272309 |  |  |  |  |  |  |  |  |  |  |  |  |  |  |
| 5 | MALPY01655 | 8747 | - | 269086 | 277751 |  |  |  |  |  | 973 | 125 | 3800 |  |  |  |  |  |  |
| 5 | Pc.c000703469.Contig1 | 7089 | - | 273408 | 280573 | cRMPc11014 | 16939 | 935 | 263634 | 280573 |  |  |  |  |  |  |  |  |  |
| 5 | Pb.c000801387.Contig1 | 11870 | + | 281508 | 293774 |  |  |  |  |  |  |  |  |  |  |  |  |  |  |
| 5 | MALPY01299 | 7828 | + | 282554 | 290466 |  |  |  |  |  | 134 | 125 |  |  |  |  |  |  |  |
| 5 | Pc.c000502392.Contig4 | 7362 | + | 285941 | 293996 |  |  |  |  |  |  |  |  |  |  |  |  |  |  |
| 5 | MALPY00928 | 28505 | - | 290153 | 319494 |  |  |  |  |  | 134 |  |  |  |  |  |  |  |  |
| 5 | Pb.c001502532.Contig1 | 1702 | + | 293870 | 295638 |  |  |  |  |  |  |  |  |  |  |  |  |  |  |
| 5 | Pc.c000002603.Contig1 | 1671 | + | 294036 | 295773 |  |  |  |  |  |  |  |  |  |  |  |  |  |  |
| 5 | Pb.c001502533.Contig1 | 1479 | + | 295670 | 297497 |  |  |  |  |  |  |  |  |  |  |  |  |  |  |
| 5 | Pc.c001605901.Contig1 | 1256 | + | 295775 | 297064 |  |  |  |  |  |  |  |  |  |  |  |  |  |  |
| 5 | Pb.c002112328.Contig1 | 897 | + | 297504 | 298401 |  |  |  |  |  |  |  |  |  |  |  |  |  |  |
| 5 | Pc.c000003131.Contig1 | 1224 | + | 297647 | 299056 |  |  |  |  |  |  |  |  |  |  |  |  |  |  |
| 5 | Pb.c001502486.Contig1 | 1836 | + | 298637 | 300437 |  |  |  |  |  |  |  |  |  |  |  |  |  |  |
| 5 | Pb.c001001428.Contig1 | 17746 | - | 300253 | 319202 |  |  |  |  |  |  |  |  |  |  |  |  |  |  |
| 5 | Pc.c000302954.Contig1 | 1417 | - | 300486 | 301903 |  |  |  |  |  |  |  |  |  |  |  |  |  |  |
| 5 | Pc.c000602852.Contig1 | 3213 | - | 302101 | 305630 |  |  |  |  |  |  |  |  |  |  |  |  |  |  |
| 5 | MALPY02990 | 750 | - | 303985 | 304702 |  |  |  |  |  |  |  |  |  |  |  |  |  |  |
| 5 | Pc.c000503354.Contig1 | 630 | + | 306715 | 307345 |  |  |  |  |  |  |  |  |  |  |  |  |  |  |
| 5 | Pc.c000302827.Contig1 | 2933 | + | 308142 | 311384 |  |  |  |  |  |  |  |  |  |  |  |  |  |  |
| 5 | Pc.c001104332.Contig1 | 1675 | + | 313247 | 314922 |  |  |  |  |  |  |  |  |  |  |  |  |  |  |
| 5 | Pc.c001103078.Contig1 | 2938 | + | 314588 | 317790 | cRMPc11015 | 37986 | 1432 | 281508 | 319494 |  |  |  | cRMPs1109 | 85403 | 1432 | 234091 | 319494 |  |
| 5 | MALPY01213 | 19274 | - | 320926 | 340756 |  |  |  |  |  |  |  |  |  |  |  |  |  |  |
| 5 | PbContig291 | 4152 | - | 320941 | 325091 |  |  |  |  |  |  |  |  |  |  |  |  |  |  |
| 5 | PcContig470 | 5528 | - | 324280 | 329646 |  |  |  |  |  |  |  |  |  |  |  |  |  |  |
| 5 | PbContig287 | 7618 | - | 324866 | 332427 |  |  |  |  |  |  |  |  |  |  |  |  |  |  |
| 5 | Pc.c001708333.Contig1 | 855 | + | 325124 | 325979 |  |  |  |  |  |  |  |  |  |  |  |  |  |  |
| 5 | MALPY03534 | 241 | - | 326122 | 326363 |  |  |  |  |  |  |  |  |  |  |  |  |  |  |
| 5 | Pb.c000301494.Contig1 | 5203 | - | 333540 | 338740 | cRMPc11016 | 19830 | 4273 | 320926 | 340756 |  |  |  | cRMPs1110 | 19830 | 4273 | 320926 | 340756 |  |
| JC5 | MALPY01351 | 4517 | xxx | 340000 |  |  |  |  |  |  | 352 |  |  |  |  |  |  |  |  |
| 5 | MALPY01164 | 6642 | + | 345029 | 351650 |  |  |  |  |  | 352 |  |  |  |  |  |  |  |  |
| 5 | PbContig171 | 3278 | - | 348044 | 351292 |  |  |  |  |  |  |  |  |  |  |  |  |  |  |
| 5 | Pc.c001204000.Contig1 | 2677 | + | 349674 | 352330 |  |  |  |  |  |  |  |  |  |  |  |  |  |  |
| 5 | Pb.c000101342.Contig1 | 2584 | - | 351256 | 354136 |  |  |  |  |  |  |  |  |  |  |  |  |  |  |
| 5 | MALPY01652 | 9288 | - | 351831 | 361505 |  |  |  |  |  | 352 |  |  |  |  |  |  |  |  |
| 5 | Pc.c001203998.Contig1 | 992 | + | 352575 | 353604 |  |  |  |  |  |  |  |  |  |  |  |  |  |  |
| 5 | Pb.c001201619.Contig1 | 5421 | + | 357698 | 364286 |  |  |  |  |  |  |  |  |  |  |  |  |  |  |
| 5 | Pc.c001606560.Contig1 | 1684 | - | 358934 | 360618 |  |  |  |  |  |  |  |  |  |  |  |  |  |  |
| 5 | PcContig485 | 1894 | - | 362426 | 364325 |  |  |  |  |  |  |  |  |  |  |  |  |  |  |
| 5 | MALPY02997 | 1799 | - | 362500 | 364338 | cRMPc11017 | 19309 | 4000 | 345029 | 364338 | 352 | 26 | 4000 |  |  |  |  |  |  |
| Centromere - PCR 4000 bp | |  |  | 365000 |  |  |  |  |  |  |  |  |  |  |  |  |  |  |  |
| 5 | MALPY01520 | 17325 | - | 368338 | 386263 |  |  |  |  |  |  | 26 | 1600 |  |  |  |  |  |  |
| 5 | Pb.c000701212.Contig2 | 11356 | - | 371913 | 384258 |  |  |  |  |  |  |  |  |  |  |  |  |  |  |
| 5 | PcContig17 | 1980 | + | 372829 | 374776 |  |  |  |  |  |  |  |  |  |  |  |  |  |  |
| 5 | Pc.c001104313.Contig1 | 628 | + | 378090 | 378712 |  |  |  |  |  |  |  |  |  |  |  |  |  |  |
| 5 | PcContig267 | 4753 | - | 379131 | 383896 | cRMPc11018 | 17925 | 1591 | 368338 | 386263 |  |  |  |  |  |  |  |  |  |
| 5 | MALPY00244 | 10506 | - | 387854 | 398363 |  |  |  |  |  | 241 | 26 |  |  |  |  |  |  |  |
| 5 | Pb.c001803367.Contig1 | 1890 | - | 387856 | 389749 |  |  |  |  |  |  |  |  |  |  |  |  |  |  |
| 5 | Pb.c000101328.Contig1 | 3816 | - | 395344 | 399223 | cRMPc11019 | 11369 | 423 | 387854 | 399223 |  |  |  |  |  |  |  |  |  |
| 5 | MALPY00597 | 6821 | + | 399646 | 406035 |  |  |  |  |  | 241 | 62 | 2000 |  |  |  |  |  |  |
| 5 | Pb.c002112316.Contig1 | 704 | - | 400506 | 401171 |  |  |  |  |  |  |  |  |  |  |  |  |  |  |
| 5 | Pc.c001505597.Contig1 | 696 | - | 400762 | 401449 |  |  |  |  |  |  |  |  |  |  |  |  |  |  |
| 5 | Pb.c001602421.Contig1 | 2795 | - | 401607 | 404357 |  |  |  |  |  |  |  |  |  |  |  |  |  |  |
| 5 | Pc.c000402878.Contig1 | 3362 | - | 401996 | 405221 | cRMPc11020 | 6389 | 1562 | 399646 | 406035 |  |  |  |  |  |  |  |  |  |
| JC5 | MALPY00944 | 4850 | + | 407597 | 412444 |  |  |  |  |  | 505 | 62 |  |  |  |  |  |  |  |
| 5 | Pc.c001405418.Contig1 | 1704 | - | 410049 | 411750 |  |  |  |  |  |  |  |  |  |  |  |  |  |  |
| 5 | Pc.c001606585.Contig1 | 779 | - | 412062 | 412841 |  |  |  |  |  |  |  |  |  |  |  |  |  |  |
| 5 | MALPY02522 | 2347 | + | 412395 | 414748 | cRMPc11021 | 7151 | 610 | 407597 | 414748 | 505 | 94 | 2500 |  |  |  |  |  |  |
| 5 | PcContig99 | 2467 | + | 415358 | 417852 |  |  |  |  |  |  |  |  |  |  |  |  |  |  |
| 5 | MALPY01282 | 3797 | - | 417534 | 421098 |  |  |  |  |  |  | 94 | 6500 |  |  |  |  |  |  |
| 5 | Pc.c001203236.Contig1 | 747 | + | 417588 | 418338 |  |  |  |  |  |  |  |  |  |  |  |  |  |  |
| 5 | Pc.c000503070.Contig1 | 3647 | + | 417832 | 421410 |  |  |  |  |  |  |  |  |  |  |  |  |  |  |
| 5 | Pb.c000501509.Contig1 | 2785 | + | 417955 | 420624 | cRMPc11022 | 6052 | 5149 | 415358 | 421410 |  |  |  |  |  |  |  |  |  |
| 5 | Pb.c000101343.Contig1 | 5527 | + | 426559 | 432099 |  |  |  |  |  |  |  |  |  |  |  |  |  |  |
| 5 | MALPY01201 | 6861 | + | 428165 | 435010 |  |  |  |  |  |  | 94 |  |  |  |  |  |  |  |
| 5 | Pc.c000802700.Contig2 | 3745 | - | 430744 | 434479 |  |  |  |  |  |  |  |  |  |  |  |  |  |  |
| 5 | Pc.c000403145.Contig1 | 607 | + | 430763 | 431364 |  |  |  |  |  |  |  |  |  |  |  |  |  |  |
| 5 | Pc.c000802648.Contig1 | 909 | - | 431042 | 431948 |  |  |  |  |  |  |  |  |  |  |  |  |  |  |
| 5 | MALPY03365 | 208 | + | 431478 | 431689 | cRMPc11023 | 8451 | 934 | 426559 | 435010 |  |  |  | cRMPs1111 | 89981 | 934 | 345029 | 435010 |  |
| 5 | Pc.c001103803.Contig1 | 5589 | - | 435944 | 441533 |  |  |  |  |  |  |  |  |  |  |  |  |  |  |
| 5 | MALPY02620 | 5682 | - | 435993 | 441672 | cRMPc11024 | 5728 | 592 | 435944 | 441672 |  | 157 | 2000 |  |  |  |  |  |  |
| 5 | Pb.c001803522.Contig1 | 2661 | - | 442264 | 444934 |  |  |  |  |  |  |  |  |  |  |  |  |  |  |
| 5 | Pc.c000802670.Contig3 | 2142 | + | 442377 | 444519 |  |  |  |  |  |  |  |  |  |  |  |  |  |  |
| 5 | MALPY00906 | 8487 | - | 443334 | 451833 |  |  |  |  |  |  |  |  |  |  |  |  |  |  |
| 5 | MALPY03179 | 639 | + | 443559 | 444207 |  |  |  |  |  |  | 157 | 600 |  |  |  |  |  |  |
| 5 | MALPY03136 | 443 | - | 444165 | 444608 | cRMPc11025 | 9569 | 2130 | 442264 | 451833 |  | 157 |  | cRMPs1112 | 15889 | 2130 | 435944 | 451833 |  |
| 5 | MALPY01418 | 6039 | + | 453963 | 459669 |  |  |  |  |  |  | 73 | 3000 |  |  |  |  |  |  |
| 5 | Pb.c001101554.Contig2 | 2294 | - | 454678 | 456857 |  |  |  |  |  |  |  |  |  |  |  |  |  |  |
| 5 | Pb.c002007602.Contig1 | 495 | - | 456972 | 457482 | cRMPc11026 | 5706 | 3884 | 453963 | 459669 |  |  |  |  |  |  |  |  |  |
| 5 | MALPY01425 | 17821 | - | 463553 | 482769 |  |  |  |  |  | 73 | 73 |  |  |  |  |  |  |  |
| 5 | Pc.c001707714.Contig1 | 1211 | - | 465994 | 467360 |  |  |  |  |  |  |  |  |  |  |  |  |  |  |
| 5 | Pb.c000901871.Contig1 | 6070 | - | 466934 | 472906 |  |  |  |  |  |  |  |  |  |  |  |  |  |  |
| 5 | PcContig611 | 4638 | - | 467570 | 516530 |  |  |  |  |  |  |  |  |  |  |  |  |  |  |
| 5 | Pc.c000203269.Contig1 | 969 | - | 476024 | 477002 |  |  |  |  |  |  |  |  |  |  |  |  |  |  |
| 5 | Pb.c001301514.Contig1 | 14190 | - | 478453 | 495983 |  |  |  |  |  |  |  |  |  |  |  |  |  |  |
| 5 | Pc.c000503086.Contig1 | 5815 | + | 479386 | 485123 |  |  |  |  |  |  |  |  |  |  |  |  |  |  |
| 5 | MALPY03400 | 839 | - | 479387 | 480226 |  |  |  |  |  |  |  |  |  |  |  |  |  |  |
| 5 | MALPY00787 | 16040 | - | 482820 | 501994 |  |  |  |  |  | 73 |  |  |  |  |  |  |  |  |
| 5 | Pc.c000703406.Contig1 | 1191 | + | 485426 | 486757 |  |  |  |  |  |  |  |  |  |  |  |  |  |  |
| 5 | PcContig104 | 8145 | + | 493840 | 502644 |  |  |  |  |  |  |  |  |  |  |  |  |  |  |
| 5 | Pb.c001602443.Contig1 | 1655 | - | 495948 | 497600 |  |  |  |  |  |  |  |  |  |  |  |  |  |  |
| 5 | Pb.c001301531.Contig1 | 4246 | - | 498643 | 502658 |  |  |  |  |  |  |  |  |  |  |  |  |  |  |
| JC5 | MALPY03110 | 1776 | + | 502323 | 504123 |  |  |  |  |  | 73 |  |  |  |  |  |  |  |  |
| 5 | Pc.c000002094.Contig2 | 4693 | - | 503616 | 508598 |  |  |  |  |  |  |  |  |  |  |  |  |  |  |
| 5 | MALPY00549 | 8739 | - | 504473 | 513507 |  |  |  |  |  | 73 | 149 | 1800 |  |  |  |  |  |  |
| 5 | Pb.c001602422.Contig1 | 3012 | + | 505093 | 508096 |  |  |  |  |  |  |  |  |  |  |  |  |  |  |
| 5 | Pb.c001301442.Contig2 | 4983 | - | 511265 | 516620 |  |  |  |  |  |  |  |  |  |  |  |  |  |  |
| 5 | Pc.c000302369.Contig2 | 7927 | - | 511471 | 520347 |  |  |  |  |  |  |  |  |  |  |  |  |  |  |
| 5 | MALPY02584 | 4770 | - | 514452 | 519460 |  |  |  |  |  |  | 149 | 6000 |  |  |  |  |  |  |
| 5 | Pb.c001802810.Contig1 | 5649 | + | 516924 | 522573 | cRMPc11027 | 59020 | 2620 | 463553 | 522573 |  |  |  |  |  |  |  |  |  |
| 5 | MALPY00922 | 8101 | - | 525193 | 533330 |  |  |  |  |  | 141 | 149 |  |  |  |  |  |  |  |
| 5 | Pc.c000703226.Contig1 | 9472 | - | 527830 | 537266 |  |  |  |  |  |  |  |  |  |  |  |  |  |  |
| 5 | Pb.c000401205.Contig1 | 6701 | - | 529728 | 536399 |  |  |  |  |  |  |  |  |  |  |  |  |  |  |
| man5 | MALPY00921 | 4050 | + | 534325 | 536518 | cRMPc11028 | 12073 | 3064 | 525193 | 537266 | 141 |  |  |  |  |  |  |  |  |
| 5 | MALPY00094 | 6725 | + | 540330 | 547262 |  |  |  |  |  | 141 |  |  |  |  |  |  |  |  |
| 5 | Pb.c000601548.Contig1 | 6195 | + | 541179 | 547581 |  |  |  |  |  |  |  |  |  |  |  |  |  |  |
| 5 | Pc.c001003391.Contig1 | 5298 | - | 541675 | 547163 |  |  |  |  |  |  |  |  |  |  |  |  |  |  |
| 5 | MALPY00595 | 8096 | + | 547041 | 555299 |  |  |  |  |  | 141 | 61 | 1900 |  |  |  |  |  |  |
| 5 | Pc.c001403998.Contig1 | 3370 | - | 547403 | 550943 |  |  |  |  |  |  |  |  |  |  |  |  |  |  |
| 5 | PcContig181 | 2028 | - | 551653 | 553866 | cRMPc11029 | 14969 | 1241 | 540330 | 555299 |  |  |  |  |  |  |  |  |  |
| 5 | MALPY00555 | 5704 | - | 556540 | 562961 |  |  |  |  |  | 1 | 61 |  |  |  |  |  |  |  |
| 5 | Pb.c001502617.Contig1 | 816 | - | 557493 | 558424 |  |  |  |  |  |  |  |  |  |  |  |  |  |  |
| 5 | Pc.c001104452.Contig1 | 979 | - | 557796 | 559153 |  |  |  |  |  |  |  |  |  |  |  |  |  |  |
| 5 | Pb.c000201284.Contig1 | 10651 | + | 558657 | 571250 |  |  |  |  |  |  |  |  |  |  |  |  |  |  |
| 5 | PcContig118 | 9238 | - | 559593 | 570890 |  |  |  |  |  |  |  |  |  |  |  |  |  |  |
| 5 | MALPY00065 - BP | 8294 | - | 563526 | 572142 |  |  |  |  |  | 1 | 355 - BP | 1300 |  |  |  |  |  |  |
| 5 | MALPY03348 | 752 | - | 564202 | 564954 | cRMPc11030 | 15602 | 1300 | 556540 | 572142 |  |  |  |  |  |  |  |  |  |
| 6 | MALPY00537 - BP | 9784 | + | 573442 | 583229 |  |  |  |  |  |  | 355 - BP | 700 |  |  |  |  |  |  |
| 6 | Pb.c001802858.Contig1 | 6057 | + | 573992 | 580055 |  |  |  |  |  |  |  |  |  |  |  |  |  |  |
| 6 | Pc.c001605970.Contig1 | 1948 | - | 575503 | 577454 | cRMPc11031 | 9787 | 1261 | 573442 | 583229 |  |  |  |  |  |  |  |  |  |
| 6 | MALPY00399 | 12041 | + | 584490 | 596638 |  |  |  |  |  | 750 | 355 |  |  |  |  |  |  |  |
| 6 | PbContig166 | 4344 | + | 588778 | 593113 | cRMPc11032 | 12148 | 79 | 584490 | 596638 |  |  |  |  |  |  |  |  |  |
| 6 | Pb.c000701168.Contig1 | 12856 | - | 596717 | 610297 |  |  |  |  |  |  |  |  |  |  |  |  |  |  |
| 6 | PcContig76 | 2713 | + | 596982 | 599698 |  |  |  |  |  |  |  |  |  |  |  |  |  |  |
| 6 | MALPY01653 | 10961 | - | 597581 | 608636 |  |  |  |  |  | 750 |  |  |  |  |  |  |  |  |
| 6 | Pc.c000902735.Contig3 | 9796 | + | 600366 | 610212 |  |  |  |  |  |  |  |  |  |  |  |  |  |  |
| 6 | MALPY03252 | 1246 | - | 604576 | 605822 |  |  |  |  |  |  |  |  |  |  |  |  |  |  |
| 6 | MALPY03353 | 240 | - | 605667 | 605904 |  |  |  |  |  |  |  |  |  |  |  |  |  |  |
| 6 | MALPY03425 | 751 | - | 605770 | 606524 |  |  |  |  |  |  | 64 | 2300 |  |  |  |  |  |  |
| 6 | MALPY03412 | 1450 | - | 609033 | 610489 | cRMPc11033 | 13772 | 96 | 596717 | 610489 | 750 | 64 | 750 |  |  |  |  |  |  |
| 6 | MALPY01273 | 3814 | + | 610585 | 614441 |  |  |  |  |  |  | 64 | 3000 |  |  |  |  |  |  |
| 6 | Pc.c000303044.Contig1 | 6274 | - | 611252 | 617563 |  |  |  |  |  |  |  |  |  |  |  |  |  |  |
| 6 | Pb.c001702881.Contig1 | 3284 | - | 611706 | 615087 |  |  |  |  |  |  |  |  |  |  |  |  |  |  |
| 6 | MALPY03040 | 1682 | - | 616566 | 618300 |  |  |  |  |  | 227 | 64 |  |  |  |  |  |  |  |
| 6 | Pb.c002006935.Contig1 | 1364 | + | 617509 | 618864 |  |  |  |  |  |  |  |  |  |  |  |  |  |  |
| 6 | Pc.c000202808.Contig1 | 1975 | - | 617777 | 619743 |  |  |  |  |  |  |  |  |  |  |  |  |  |  |
| man6 | MALPY01592 - Ins | 17034 | - | 618225 | 637549 |  |  |  |  |  | 227 | 66B |  |  |  |  |  |  |  |
| man6 | Pb.c000701496.Contig1 - Ins | 4473 | + | 618926 | 623976 |  |  |  |  |  |  |  |  |  |  |  |  |  |  |
| 6 | Pc.c001813589.Contig1 | 2587 | - | 623996 | 626583 |  |  |  |  |  |  |  |  |  |  |  |  |  |  |
| 6 | Pb.c001803530.Contig1 | 2168 | + | 624052 | 626220 |  |  |  |  |  |  |  |  |  |  |  |  |  |  |
| 6 | Pc.c001605828.Contig1 | 911 | + | 628979 | 629935 |  |  |  |  |  |  |  |  |  |  |  |  |  |  |
| 6 | Pb.c000701750.Contig1 | 2026 | + | 630135 | 632125 |  |  |  |  |  |  |  |  |  |  |  |  |  |  |
| 6 | Pb.c001803529.Contig1 | 2945 | + | 635519 | 638868 |  |  |  |  |  |  |  |  |  |  |  |  |  |  |
| 6 | Pc.c001505062.Contig1 | 1973 | + | 636217 | 638196 |  |  |  |  |  |  |  |  |  |  |  |  |  |  |
| 6 | MALPY03108 | 737 | - | 638383 | 639126 | cRMPc11034 | 28541 | 196 | 610585 | 639126 |  | 189\_alt | 600 |  |  |  |  |  |  |
| 6 | MALPY01749 | 2473 | - | 639322 | 641789 |  |  |  |  |  |  | 189\_alt | 1100 |  |  |  |  |  |  |
| 6 | PcContig35 | 2582 | - | 639695 | 642265 |  |  |  |  |  |  |  |  |  |  |  |  |  |  |
| 6 | Pb.c001802908.Contig1 | 5280 | + | 642259 | 647589 |  |  |  |  |  |  |  |  |  |  |  |  |  |  |
| 6 | MALPY00972 | 25453 | + | 643258 | 671208 |  |  |  |  |  |  | 189\_alt | 900 |  |  |  |  |  |  |
| 6 | Pb.c001402147.Contig1 | 2494 | + | 649304 | 651798 |  |  |  |  |  |  |  |  |  |  |  |  |  |  |
| 6 | Pc.c001811889.Contig1 | 3702 | + | 654014 | 657609 |  |  |  |  |  |  |  |  |  |  |  |  |  |  |
| 6 | PcContig160 | 4327 | + | 657532 | 662642 |  |  |  |  |  |  |  |  |  |  |  |  |  |  |
| 6 | Pc.c001505389.Contig1 | 876 | + | 663999 | 664914 |  |  |  |  |  |  |  |  |  |  |  |  |  |  |
| 6 | Pc.c000703285.Contig1 | 3793 | + | 665285 | 670312 |  |  |  |  |  |  |  |  |  |  |  |  |  |  |
| 6 | Pb.c001803546.Contig1 | 2196 | - | 667678 | 670048 |  |  |  |  |  |  |  |  |  |  |  |  |  |  |
| 6 | Pb.c001001778.Contig1 | 3743 | - | 670409 | 674416 |  |  |  |  |  |  |  |  |  |  |  |  |  |  |
| 6 | Pc.c001812454.Contig1 | 613 | + | 670540 | 671153 |  |  |  |  |  |  |  |  |  |  |  |  |  |  |
| 6 | MALPY01561 | 2376 | + | 671745 | 674136 |  |  |  |  |  | 728 | 189\_alt | 3200 |  |  |  |  |  |  |
| 6 | PcContig38 | 6784 | + | 674155 | 681595 |  |  |  |  |  |  |  |  |  |  |  |  |  |  |
| 6 | MALPY01422 | 4112 | + | 674290 | 678339 |  |  |  |  |  | 728 |  |  |  |  |  |  |  |  |
| 6 | Pb.c001002007.Contig1 | 1541 | - | 674431 | 675942 |  |  |  |  |  |  |  |  |  |  |  |  |  |  |
| 6 | MALPY00840 | 11900 | - | 676960 | 687925 |  |  |  |  |  | 109 | 189\_alt |  |  |  |  |  |  |  |
| 6 | Pb.c000201579.Contig1 | 2351 | + | 678277 | 680628 |  |  |  |  |  |  |  |  |  |  |  |  |  |  |
| 6 | Pb.c000201581.Contig1 | 4585 | + | 679522 | 684282 |  |  |  |  |  |  |  |  |  |  |  |  |  |  |
| 6 | Pc.c001204239.Contig1 | 5443 | + | 680727 | 686319 |  |  |  |  |  |  |  |  |  |  |  |  |  |  |
| 6 | Pb.c001402196.Contig1 | 3843 | + | 686529 | 690405 |  |  |  |  |  |  |  |  |  |  |  |  |  |  |
| 6 | MALPY01071 | 5449 | - | 687738 | 693208 |  |  |  |  |  | 109 |  |  |  |  |  |  |  |  |
| 6 | Pc.c001606526.Contig1 | 662 | + | 689774 | 690469 |  |  |  |  |  |  |  |  |  |  |  |  |  |  |
| 6 | Pb.c000201605.Contig1 | 3805 | + | 690587 | 694576 |  |  |  |  |  |  |  |  |  |  |  |  |  |  |
| 6 | Pc.c001304085.Contig1 | 4248 | + | 691334 | 695859 |  |  |  |  |  |  |  |  |  |  |  |  |  |  |
| 6 | MALPY01847 | 7204 | + | 693237 | 700492 |  |  |  |  |  | 109 |  |  |  |  |  |  |  |  |
| 6 | PbContig381 | 2795 | - | 695092 | 698001 |  |  |  |  |  |  |  |  |  |  |  |  |  |  |
| 6 | Pb.c000701193.Contig3 | 3344 | - | 699901 | 703340 |  |  |  |  |  |  |  |  |  |  |  |  |  |  |
| 6 | PcContig357 | 3393 | - | 700149 | 703640 |  |  |  |  |  |  |  |  |  |  |  |  |  |  |
| 6 | MALPY02608 | 3140 | - | 700880 | 704063 | cRMPc11035 | 64741 | 3709 | 639322 | 704063 | 109 | 61 | 3000 |  |  |  |  |  |  |
| 6 | MALPY00926 | 14442 | - | 707772 | 722895 |  |  |  |  |  |  | 61 | 1500 |  |  |  |  |  |  |
| 6 | Pc.c000402863.Contig1 | 2802 | - | 712568 | 715379 |  |  |  |  |  |  |  |  |  |  |  |  |  |  |
| 6 | PbContig339 | 4286 | - | 712638 | 716983 |  |  |  |  |  |  |  |  |  |  |  |  |  |  |
| 6 | Pb.c000701590.Contig1 | 3985 | - | 717778 | 721763 |  |  |  |  |  |  |  |  |  |  |  |  |  |  |
| 6 | Pc.c001304190.Contig1 | 3510 | - | 718406 | 721916 | cRMPc11036 | 15123 | 1588 | 707772 | 722895 |  |  |  |  |  |  |  |  |  |
| 6 | MALPY01271 | 10095 | - | 724483 | 734738 |  |  |  |  |  | 186 | 61 |  |  |  |  |  |  |  |
| 6 | Pb.c001905045.Contig1 | 732 | + | 725024 | 725756 |  |  |  |  |  |  |  |  |  |  |  |  |  |  |
| 6 | PcContig724 | 5609 | + | 732864 | 739155 |  |  |  |  |  |  |  |  |  |  |  |  |  |  |
| 6 | Pb.c000501185.Contig1 | 3885 | + | 733029 | 737340 |  |  |  |  |  |  |  |  |  |  |  |  |  |  |
| 6 | Pc.c001204491.Contig1 | 858 | + | 734553 | 735411 |  |  |  |  |  |  |  |  |  |  |  |  |  |  |
| 6 | MALPY01369 | 14201 | - | 734706 | 749693 |  |  |  |  |  | 186 |  |  |  |  |  |  |  |  |
| 6 | Pc.c000003001.Contig1 | 481 | + | 738523 | 739001 |  |  |  |  |  |  |  |  |  |  |  |  |  |  |
| 6 | Pb.c001903654.Contig1 | 2142 | + | 738852 | 741089 |  |  |  |  |  |  |  |  |  |  |  |  |  |  |
| 6 | Pb.c002005844.Contig1 | 2142 | + | 738852 | 741089 |  |  |  |  |  |  |  |  |  |  |  |  |  |  |
| 6 | PcContig609 | 2168 | + | 739152 | 741326 |  |  |  |  |  |  |  |  |  |  |  |  |  |  |
| 6 | Pb.c000401423.Contig1 | 6564 | + | 741562 | 747739 |  |  |  |  |  |  |  |  |  |  |  |  |  |  |
| 6 | Pc.c001104123.Contig1 | 2462 | + | 742153 | 744613 |  |  |  |  |  |  |  |  |  |  |  |  |  |  |
| 6 | Pc.c001103813.Contig1 | 2596 | + | 744747 | 747343 |  |  |  |  |  |  |  |  |  |  |  |  |  |  |
| 6 | MALPY03268 | 834 | + | 745607 | 746441 | cRMPc11037 | 25210 | 2136 | 724483 | 749693 |  | 78 | 6000 |  |  |  |  |  |  |
| 6 | MALPY01307 | 8017 | + | 751829 | 759867 |  |  |  |  |  |  | 78 | 900 |  |  |  |  |  |  |
| 6 | PcContig564 | 6975 | - | 752501 | 759652 |  |  |  |  |  |  |  |  |  |  |  |  |  |  |
| 6 | PbContig284 | 6170 | - | 753644 | 759569 | cRMPc11038 | 8038 | 491 | 751829 | 759867 |  |  |  |  |  |  |  |  |  |
| 6 | Pc.c000502984.Contig1 | 3085 | - | 760358 | 763413 |  |  |  |  |  |  |  |  |  |  |  |  |  |  |
| 6 | MALPY02083 | 6559 | - | 760386 | 767165 |  |  |  |  |  |  | 78 |  |  |  |  |  |  |  |
| 6 | Pb.c000801838.Contig1 | 2954 | + | 761725 | 764742 |  |  |  |  |  |  |  |  |  |  |  |  |  |  |
| 6 | Pc.c000002036.Contig1 | 6332 | + | 764755 | 771083 |  |  |  |  |  |  |  |  |  |  |  |  |  |  |
| 6 | Pb.c000801837.Contig1 | 1762 | + | 765277 | 767035 |  |  |  |  |  |  |  |  |  |  |  |  |  |  |
| 6 | MALPY00392 | 12555 | + | 765773 | 777991 |  |  |  |  |  | 1101 |  |  |  |  |  |  |  |  |
| 6 | PcContig481 | 2982 | + | 772270 | 775252 |  |  |  |  |  |  |  |  |  |  |  |  |  |  |
| 6 | Pb.c001803311.Contig1 | 3562 | - | 774515 | 778077 |  |  |  |  |  |  |  |  |  |  |  |  |  |  |
| 6 | Pc.c001304259.Contig1 | 1608 | - | 776914 | 778522 |  |  |  |  |  |  |  |  |  |  |  |  |  |  |
| 6 | MALPY00339 | 5128 | + | 777331 | 783030 |  |  |  |  |  | 1101 |  |  |  |  |  |  |  |  |
| 6 | PbContig213 | 3942 | - | 777477 | 781587 |  |  |  |  |  |  |  |  |  |  |  |  |  |  |
| 6 | Pc.c001304261.Contig1 | 1707 | - | 778500 | 780378 |  |  |  |  |  |  |  |  |  |  |  |  |  |  |
| 6 | PcContig675 | 3559 | + | 781271 | 785004 |  |  |  |  |  |  |  |  |  |  |  |  |  |  |
| 6 | Pb.c001602820.Contig1 | 890 | - | 782221 | 783132 |  |  |  |  |  |  |  |  |  |  |  |  |  |  |
| 6 | MALPY00607 | 5799 | + | 782927 | 788800 |  |  |  |  |  | 1101 | 284 | 500 |  |  |  |  |  |  |
| 6 | Pb.c000001357.Contig1 | 2598 | + | 783695 | 786354 |  |  |  |  |  |  |  |  |  |  |  |  |  |  |
| 6 | PbContig396 | 3248 | - | 788392 | 791760 |  |  |  |  |  |  |  |  |  |  |  |  |  |  |
| 6 | MALPY00608 | 3337 | - | 789044 | 792492 |  |  |  |  |  | 1067 | 284 |  |  |  |  |  |  |  |
| 6 | PcContig37 | 8513 | + | 789170 | 798798 |  |  |  |  |  |  |  |  |  |  |  |  |  |  |
| 6 | MALPY00600 | 11247 | - | 792558 | 804264 |  |  |  |  |  | 1067 |  |  |  |  |  |  |  |  |
| 6 | Pb.c000601600.Contig1 | 2381 | - | 793088 | 795703 |  |  |  |  |  |  |  |  |  |  |  |  |  |  |
| 6 | MALPY03194 | 398 | - | 797446 | 797844 |  |  |  |  |  |  |  |  |  |  |  |  |  |  |
| 6 | Pb.c001202088.Contig1 | 3225 | - | 797485 | 800710 |  |  |  |  |  |  |  |  |  |  |  |  |  |  |
| 6 | Pb.c001402098.Contig1 | 2629 | - | 801453 | 803950 |  |  |  |  |  |  |  |  |  |  |  |  |  |  |
| 6 | Pc.c000703399.Contig1 | 1436 | - | 801519 | 802955 | cRMPc11039 | 43906 | 5147 | 760358 | 804264 |  |  |  | cRMPs1113 | 350301 | 5147 | 453963 | 804264 |  |
| 6 | Pc.c001505181.Contig1 | 3804 | - | 809411 | 813320 |  |  |  |  |  |  |  |  |  |  |  |  |  |  |
| 6 | MALPY01224 | 3243 | - | 809886 | 813180 |  |  |  |  |  | 858B | 350 | 600 |  |  |  |  |  |  |
| 6 | Pb.c001001702.Contig1 | 8469 | + | 810403 | 818724 |  |  |  |  |  |  |  |  |  |  |  |  |  |  |
| 6 | MALPY01011 | 3575 | - | 813224 | 816833 |  |  |  |  |  | 236 | 350 |  |  |  |  |  |  |  |
| 6 | PcContig501 | 4658 | + | 813652 | 818447 |  |  |  |  |  |  |  |  |  |  |  |  |  |  |
| 6 | MALPY02106 | 7396 | + | 816402 | 824178 |  |  |  |  |  | 236 | 175 | 350 |  |  |  |  |  |  |
| 6 | Pc.c000302914.Contig1 | 1671 | + | 819253 | 820963 |  |  |  |  |  |  |  |  |  |  |  |  |  |  |
| 6 | Pb.c001502848.Contig1 | 1073 | - | 819998 | 821077 |  |  |  |  |  |  |  |  |  |  |  |  |  |  |
| 6 | Pc.c000302364.Contig3 | 6676 | + | 820939 | 827732 |  |  |  |  |  |  |  |  |  |  |  |  |  |  |
| 6 | PbContig319 | 3849 | - | 821245 | 825132 |  |  |  |  |  |  |  |  |  |  |  |  |  |  |
| 6 | MALPY01519 | 6302 | + | 822727 | 829077 |  |  |  |  |  | 236 |  |  |  |  |  |  |  |  |
| 6 | MALPY03087 | 392 | + | 824015 | 824413 |  |  |  |  |  |  | 175 |  |  |  |  |  |  |  |
| 6 | Pb.c000401211.Contig2 | 12639 | - | 825697 | 839114 |  |  |  |  |  |  |  |  |  |  |  |  |  |  |
| 6 | MALPY01257 | 12383 | - | 829039 | 842567 |  |  |  |  |  | 236 |  |  |  |  |  |  |  |  |
| 6 | PcContig591 | 3603 | - | 830049 | 833709 |  |  |  |  |  |  |  |  |  |  |  |  |  |  |
| 6 | MALPY03426 | 175 | - | 831477 | 831652 |  |  |  |  |  |  |  |  |  |  |  |  |  |  |
| 6 | Pc.c001504206.Contig2 | 2668 | + | 834772 | 837794 |  |  |  |  |  |  |  |  |  |  |  |  |  |  |
| 6 | Pb.c000501691.Contig1 | 1692 | + | 839561 | 841289 |  |  |  |  |  |  |  |  |  |  |  |  |  |  |
| 6 | Pc.c000102510.Contig1 | 1773 | - | 841303 | 843058 |  |  |  |  |  |  |  |  |  |  |  |  |  |  |
| 6 | Pb.c000501399.Contig1 | 1582 | + | 841545 | 843145 | cRMPc11040 | 33734 | 5538 | 809411 | 843145 |  |  |  | cRMPs1114 | 33734 | 5538 | 809411 | 843145 |  |
| 6 | Pb.c000501455.Contig1 | 3398 | - | 848683 | 852081 |  |  |  |  |  |  |  |  |  |  |  |  |  |  |
| 6 | MALPY02240 | 3043 | - | 849344 | 852387 |  |  |  |  |  |  | 102 | 700 |  |  |  |  |  |  |
| 6 | Pc.c000403315.Contig1 | 774 | + | 850266 | 851040 | cRMPc11041 | 3704 | 844 | 848683 | 852387 |  |  |  |  |  |  |  |  |  |
| 6 | Pb.c000501156.Contig1 | 8559 | - | 853231 | 861783 |  |  |  |  |  |  |  |  |  |  |  |  |  |  |
| 6 | MALPY00559 | 6831 | + | 853532 | 860251 |  |  |  |  |  | 764 | 102 |  |  |  |  |  |  |  |
| 6 | Pc.c000402920.Contig1 | 5126 | - | 855643 | 861748 |  |  |  |  |  |  |  |  |  |  |  |  |  |  |
| 6 | MALPY01356 | 8817 | - | 860573 | 870511 |  |  |  |  |  | 764 | 225 | 1400 |  |  |  |  |  |  |
| 6 | Pc.c000603378.Contig1 | 1416 | - | 863024 | 864419 |  |  |  |  |  |  |  |  |  |  |  |  |  |  |
| 6 | Pb.c001001391.Contig2 | 5539 | - | 863809 | 869354 |  |  |  |  |  |  |  |  |  |  |  |  |  |  |
| 6 | Pc.c001504944.Contig1 | 2575 | - | 866929 | 869504 | cRMPc11042 | 17280 | 1262 | 853231 | 870511 |  |  |  |  |  |  |  |  |  |
| 6 | MALPY01821 | 5269 | - | 871773 | 876898 |  |  |  |  |  | 292 | 225 |  |  |  |  |  |  |  |
| 6 | Pb.c001702743.Contig1 | 4187 | - | 872346 | 876482 |  |  |  |  |  |  |  |  |  |  |  |  |  |  |
| 6 | PcContig503 | 2161 | + | 872658 | 874840 |  |  |  |  |  |  |  |  |  |  |  |  |  |  |
| 6 | MALPY03182 | 346 | - | 873840 | 874186 |  |  |  |  |  |  | 18 | 2200 |  |  |  |  |  |  |
| 6 | MALPY02057 | 4755 | - | 876114 | 880836 |  |  |  |  |  | 292 | 18 |  |  |  |  |  |  |  |
| 6 | Pb.c001702742.Contig1 | 2579 | - | 876583 | 879174 |  |  |  |  |  |  |  |  |  |  |  |  |  |  |
| 6 | Pc.c001812280.Contig1 | 6750 | - | 876586 | 883452 |  |  |  |  |  |  |  |  |  |  |  |  |  |  |
| man6 | MALPY02154 | 5456 | - | 883183 | 888056 |  |  |  |  |  |  |  |  |  |  |  |  |  |  |
| man6 | MALPY02324 | 2226 | + | 887200 | 889426 | cRMPc11043 | 17653 | 1710 | 871773 | 889426 |  |  |  | cRMPs1115 | 40743 | 1710 | 848683 | 889426 | MALPY02154 |
| 6 | MALPY00639 | 15985 | - | 891136 | 906545 |  |  |  |  |  |  | 174 - NS | 4000 |  |  |  |  |  |  |
| 6 | Pb.c000101068.Contig1 | 6071 | - | 892304 | 898360 |  |  |  |  |  |  |  |  |  |  |  |  |  |  |
| 6 | Pc.c001505435.Contig1 | 1348 | + | 896126 | 897468 |  |  |  |  |  |  |  |  |  |  |  |  |  |  |
| 6 | Pc.c000002069.Contig1 | 4310 | + | 899664 | 904058 |  |  |  |  |  |  |  |  |  |  |  |  |  |  |
| 6 | Pb.c001301508.Contig1 | 4552 | - | 900998 | 905550 | cRMPc11044 | 15409 | 3868 | 891136 | 906545 |  |  |  |  |  |  |  |  |  |
| 6 | Pb.c001201937.Contig1 | 4785 | - | 910413 | 915672 |  |  |  |  |  |  |  |  |  |  |  |  |  |  |
| man6 | MALPY00563 - Ins | 7638 | + | 910545 | 918702 |  |  |  |  |  |  | 174 - NS | 5000 |  |  |  |  |  |  |
| 6 | Pc.c001003993.Contig1 | 602 | - | 912074 | 912679 | cRMPc11045 | 8289 | 87 | 910413 | 918702 |  |  |  |  |  |  |  |  |  |
| 6 | MALPY02441 | 3624 | + | 918789 | 922419 | cRMPc11046 | 3630 | 912 | 918789 | 922419 |  |  |  |  |  |  |  |  |  |
| 6 | PbContig214 | 5103 | - | 923331 | 928434 |  |  |  |  |  |  |  |  |  |  |  |  |  |  |
| JC6 | MALPY01559 | 4896 | - | 924981 | 929982 |  |  |  |  |  |  | 174 | 1400 |  |  |  |  |  |  |
| 6 | Pb.c002006999.Contig1 | 3306 | + | 929771 | 933080 |  |  |  |  |  |  |  |  |  |  |  |  |  |  |
| 6 | Pc.c001204563.Contig1 | 1255 | - | 930084 | 931339 |  |  |  |  |  |  |  |  |  |  |  |  |  |  |
| 6 | MALPY01361 | 4560 | + | 930824 | 935384 |  |  |  |  |  |  | 174 | 1200 |  |  |  |  |  |  |
| 6 | Pc.c000803291.Contig1 | 2139 | - | 931468 | 933607 |  |  |  |  |  |  |  |  |  |  |  |  |  |  |
| 6 | Pb.c001702853.Contig1 | 1121 | - | 934327 | 935448 |  |  |  |  |  |  |  |  |  |  |  |  |  |  |
| 6 | MALPY03086 | 835 | + | 935189 | 936062 | cRMPc11047 | 12731 | 2677 | 923331 | 936062 |  | 174 | 3200 |  |  |  |  |  |  |
| 6 | Pb.c001001367.Contig1 | 3457 | - | 938739 | 942196 |  |  |  |  |  |  |  |  |  |  |  |  |  |  |
| 6 | MALPY02800 | 6711 | + | 939880 | 947324 |  |  |  |  |  | 782 | 174 |  |  |  |  |  |  |  |
| 6 | Pb.c001001671.Contig1 | 1448 | - | 942259 | 944127 |  |  |  |  |  |  |  |  |  |  |  |  |  |  |
| 6 | Pb.c001101836.Contig1 | 1911 | + | 944794 | 946723 |  |  |  |  |  |  |  |  |  |  |  |  |  |  |
| 6 | Pc.c001708678.Contig1 | 733 | + | 945154 | 945887 |  |  |  |  |  |  |  |  |  |  |  |  |  |  |
| 6 | PcContig543 | 5511 | - | 946996 | 952608 |  |  |  |  |  |  |  |  |  |  |  |  |  |  |
| man6 | MALPY01429 - Ins | 24847 | - | 947459 | 974278 |  |  |  |  |  | 782 | new | 2000 |  |  |  |  |  |  |
| man6 | No Centromere |  |  | 947459 | 1023912 |  |  |  |  |  |  |  |  |  |  |  |  |  |  |
| 6 | Pb.c000901399.Contig1 | 4317 | + | 948448 | 952834 |  |  |  |  |  |  |  |  |  |  |  |  |  |  |
| 6 | PcContig788 | 2978 | - | 952891 | 955845 |  |  |  |  |  |  |  |  |  |  |  |  |  |  |
| 6 | Pb.c000901732.Contig1 | 1366 | - | 953070 | 954412 |  |  |  |  |  |  |  |  |  |  |  |  |  |  |
| man6 | Pc.c000203144.Contig1 - Ins | 6412 | - | 960989 | 967244 |  |  |  |  |  |  |  |  |  |  |  |  |  |  |
| man6 | Pb.c001202023.Contig1 - Ins | 5320 | - | 961187 | 966351 |  |  |  |  |  |  |  |  |  |  |  |  |  |  |
| 6 | Pb.c001904302.Contig1 | 4149 | - | 966302 | 970588 |  |  |  |  |  |  |  |  |  |  |  |  |  |  |
| 6 | Pc.c001708490.Contig1 | 826 | - | 968063 | 968889 |  |  |  |  |  |  |  |  |  |  |  |  |  |  |
| 6 | Pc.c001303451.Contig2 | 3021 | - | 968978 | 972182 |  |  |  |  |  |  |  |  |  |  |  |  |  |  |
| 6 | Pb.c002006934.Contig1 | 3523 | - | 971006 | 974526 |  |  |  |  |  |  |  |  |  |  |  |  |  |  |
| 6 | MALPY00748 | 42524 | + | 975728 | 1023912 |  |  |  |  |  |  | new |  |  |  |  |  |  |  |
| 6 | Pb.c000101402.Contig1 | 8868 | + | 975997 | 985785 |  |  |  |  |  |  |  |  |  |  |  |  |  |  |
| 6 | Pc.c000402784.Contig1 | 4445 | - | 976704 | 981059 |  |  |  |  |  |  |  |  |  |  |  |  |  |  |
| 6 | Pc.c000202677.Contig1 | 2967 | - | 981890 | 985127 |  |  |  |  |  |  |  |  |  |  |  |  |  |  |
| 6 | Pb.c001301769.Contig1 | 6101 | + | 987629 | 994320 |  |  |  |  |  |  |  |  |  |  |  |  |  |  |
| 6 | Pc.c000502460.Contig1 | 3942 | + | 988284 | 992451 |  |  |  |  |  |  |  |  |  |  |  |  |  |  |
| 6 | MALPY03423 | 936 | + | 992704 | 994057 |  |  |  |  |  |  |  |  |  |  |  |  |  |  |
| 6 | Pc.c001304269.Contig1 | 1596 | + | 993220 | 994861 |  |  |  |  |  |  |  |  |  |  |  |  |  |  |
| 6 | Pb.c001602045.Contig3 | 2866 | - | 995839 | 999620 |  |  |  |  |  |  |  |  |  |  |  |  |  |  |
| 6 | Pc.c000403314.Contig1 | 648 | + | 998878 | 999517 |  |  |  |  |  |  |  |  |  |  |  |  |  |  |
| 6 | Pc.c001505503.Contig1 | 1050 | - | 1002493 | 1003540 |  |  |  |  |  |  |  |  |  |  |  |  |  |  |
| 6 | Pb.c001001772.Contig1 | 3782 | + | 1002792 | 1006863 |  |  |  |  |  |  |  |  |  |  |  |  |  |  |
| 6 | Pc.c001707810.Contig1 | 1644 | - | 1004132 | 1005791 |  |  |  |  |  |  |  |  |  |  |  |  |  |  |
| 6 | PbContig159 | 4303 | + | 1007728 | 1012651 |  |  |  |  |  |  |  |  |  |  |  |  |  |  |
| 6 | Pc.c000803371.Contig1 | 3015 | + | 1009611 | 1012830 |  |  |  |  |  |  |  |  |  |  |  |  |  |  |
| 6 | Pb.c001001810.Contig1 | 2799 | - | 1012979 | 1016075 |  |  |  |  |  |  |  |  |  |  |  |  |  |  |
| 6 | Pc.c000903341.Contig1 | 1310 | + | 1013002 | 1014405 |  |  |  |  |  |  |  |  |  |  |  |  |  |  |
| 6 | MALPY03303 | 849 | - | 1014965 | 1015814 |  |  |  |  |  |  |  |  |  |  |  |  |  |  |
| 6 | Pb.c001904448.Contig1 | 1551 | - | 1016108 | 1017842 |  |  |  |  |  |  |  |  |  |  |  |  |  |  |
| 6 | Pb.c001904958.Contig1 | 1180 | - | 1017901 | 1019072 |  |  |  |  |  |  |  |  |  |  |  |  |  |  |
| 6 | MALPY03240 | 804 | - | 1018904 | 1019729 |  |  |  |  |  |  | 185 | 8000 |  |  |  |  |  |  |
| 6 | Pc.c001606005.Contig1 | 1013 | - | 1019543 | 1020676 |  |  |  |  |  |  |  |  |  |  |  |  |  |  |
| 6 | Pb.c002006820.Contig1 | 2757 | - | 1020274 | 1023076 |  |  |  |  |  |  |  |  |  |  |  |  |  |  |
| 6 | Pc.c001304146.Contig1 | 2505 | - | 1020732 | 1023282 | cRMPc11048 | 85173 | 4238 | 938739 | 1023912 |  |  |  |  |  |  |  |  |  |
| 6 | MALPY01779 | 12024 | - | 1028150 | 1041383 |  |  |  |  |  |  | 185 | 3000 |  |  |  |  |  |  |
| 6 | Pb.c000201562.Contig1 | 5663 | - | 1028429 | 1034680 |  |  |  |  |  |  |  |  |  |  |  |  |  |  |
| 6 | PcContig440 | 3277 | - | 1032669 | 1036528 |  |  |  |  |  |  |  |  |  |  |  |  |  |  |
| 6 | Pb.c001101500.Contig2 | 5247 | - | 1035308 | 1040600 |  |  |  |  |  |  |  |  |  |  |  |  |  |  |
| 6 | Pc.c000603526.Contig1 | 1195 | + | 1036530 | 1037737 | cRMPc11049 | 13233 | 4055 | 1028150 | 1041383 |  |  |  |  |  |  |  |  |  |
| 6 | MALPY02147 | 5760 | + | 1045438 | 1051074 |  |  |  |  |  | 242 | 185 |  |  |  |  |  |  |  |
| 6 | Pb.c001904293.Contig1 | 2319 | - | 1046132 | 1048493 |  |  |  |  |  |  |  |  |  |  |  |  |  |  |
| 6 | Pc.c001505078.Contig1 | 1834 | - | 1046982 | 1048819 | cRMPc11050 | 5636 | 552 | 1045438 | 1051074 |  |  |  |  |  |  |  |  |  |
| 6 | MALPY01031 | 13451 | - | 1051626 | 1070289 |  |  |  |  |  | 242 |  |  |  |  |  |  |  |  |
| 6 | Pc.c000402934.Contig1 | 3572 | + | 1053742 | 1057263 |  |  |  |  |  |  |  |  |  |  |  |  |  |  |
| 6 | Pb.c001602845.Contig1 | 2658 | - | 1053897 | 1056507 |  |  |  |  |  |  |  |  |  |  |  |  |  |  |
| 6 | Pc.c000603381.Contig1 | 1170 | + | 1062096 | 1063272 |  |  |  |  |  |  |  |  |  |  |  |  |  |  |
| 6 | Pc.c001707765.Contig1 | 1513 | + | 1063842 | 1065355 |  |  |  |  |  |  |  |  |  |  |  |  |  |  |
| 6 | PbContig384 | 6387 | - | 1064185 | 1070820 |  |  |  |  |  |  |  |  |  |  |  |  |  |  |
| 6 | Pc.c000903932.Contig1 | 1070 | + | 1065373 | 1066440 |  |  |  |  |  |  |  |  |  |  |  |  |  |  |
| 6 | MALPY01249 | 15435 | - | 1067724 | 1083994 |  |  |  |  |  |  |  |  |  |  |  |  |  |  |
| 6 | Pc.c000602344.Contig1 | 2510 | + | 1080698 | 1083208 |  |  |  |  |  |  |  |  |  |  |  |  |  |  |
| 6 | Pb.c001402185.Contig1 | 4137 | + | 1081284 | 1085445 | cRMPc11051 | 33819 | 5094 | 1051626 | 1085445 |  |  |  | cRMPs1116 | 194309 | 5094 | 891136 | 1085445 |  |
| 6 | MALPY00188 | 14505 | + | 1090539 | 1105185 |  |  |  |  |  |  | 20 | 800 |  |  |  |  |  |  |
| 6 | Pb.c000501133.Contig1 | 5221 | - | 1090839 | 1096192 |  |  |  |  |  |  |  |  |  |  |  |  |  |  |
| 6 | Pc.c001812429.Contig1 | 577 | + | 1094625 | 1095202 | cRMPc11052 | 14646 | 197 | 1090539 | 1105185 |  |  |  |  |  |  |  |  |  |
| 6 | PbContig318 | 5485 | - | 1105382 | 1111411 |  |  |  |  |  |  |  |  |  |  |  |  |  |  |
| 6 | MALPY00612 | 8337 | - | 1105928 | 1115089 |  |  |  |  |  | 384B | 20 | 1600 |  |  |  |  |  |  |
| 6 | Pc.c000703236.Contig1 | 4591 | + | 1107121 | 1111955 |  |  |  |  |  |  |  |  |  |  |  |  |  |  |
| 6 | Pb.c000701596.Contig1 | 3365 | - | 1111666 | 1115151 |  |  |  |  |  |  |  |  |  |  |  |  |  |  |
| 6 | Pc.c000602903.Contig1 | 553 | + | 1112262 | 1112809 | cRMPc11053 | 9769 | 1908 | 1105382 | 1115151 |  |  |  |  |  |  |  |  |  |
| 6 | PbContig108 | 6183 | + | 1117059 | 1123267 |  |  |  |  |  |  |  |  |  |  |  |  |  |  |
| 6 | MALPY01258 | 4914 | + | 1117276 | 1122201 |  |  |  |  |  |  | 20 | 700 |  |  |  |  |  |  |
| 6 | MALPY02101 | 2819 | - | 1122204 | 1125173 |  |  |  |  |  | 931 | 20 |  |  |  |  |  |  |  |
| 6 | Pc.c000703324.Contig1 | 2356 | - | 1122935 | 1125468 |  |  |  |  |  |  |  |  |  |  |  |  |  |  |
| 6 | MALPY03416 | 773 | - | 1123857 | 1124711 | cRMPc11054 | 8409 | 418 | 1117059 | 1125468 |  | 213 | 1700 |  |  |  |  |  |  |
| 6 | MALPY02028 | 4830 | - | 1125886 | 1130752 |  |  |  |  |  | 931 | 213 |  |  |  |  |  |  |  |
| 6 | Pb.c000601561.Contig1 | 2975 | - | 1125893 | 1128772 |  |  |  |  |  |  |  |  |  |  |  |  |  |  |
| 6 | Pc.c001104293.Contig1 | 815 | + | 1126412 | 1127227 |  |  |  |  |  |  |  |  |  |  |  |  |  |  |
| 6 | Pc.c001405119.Contig1 | 855 | + | 1127595 | 1128450 |  |  |  |  |  |  |  |  |  |  |  |  |  |  |
| 6 | Pc.c001204520.Contig1 | 2106 | - | 1129802 | 1132097 |  |  |  |  |  |  |  |  |  |  |  |  |  |  |
| 6 | MALPY01694 | 6850 | + | 1130858 | 1137795 |  |  |  |  |  | 931 |  |  |  |  |  |  |  |  |
| 6 | Pb.c001602455.Contig1 | 3918 | + | 1132007 | 1136015 |  |  |  |  |  |  |  |  |  |  |  |  |  |  |
| 6 | Pc.c001405354.Contig1 | 1512 | - | 1132524 | 1134036 |  |  |  |  |  |  |  |  |  |  |  |  |  |  |
| 6 | MALPY03541 | 396 | - | 1133092 | 1133485 |  |  |  |  |  |  | 332 | 2800 |  |  |  |  |  |  |
| 6 | Pc.c000403357.Contig1 | 1241 | - | 1134073 | 1135416 |  |  |  |  |  |  |  |  |  |  |  |  |  |  |
| 6 | Pc.c000303057.Contig1 | 3036 | - | 1135565 | 1138559 |  |  |  |  |  |  |  |  |  |  |  |  |  |  |
| 6 | MALPY03343 | 151 | - | 1136027 | 1136178 |  |  |  |  |  |  | 332 |  |  |  |  |  |  |  |
| 6 | Pb.c001904519.Contig1 | 2163 | + | 1136171 | 1138261 | cRMPc11055 | 12673 | 6119 | 1125886 | 1138559 |  |  |  | cRMPs1117 | 48020 | 6119 | 1090539 | 1138559 |  |
| 6 | MALPY01020 | 2731 | - | 1144678 | 1147412 |  |  |  |  |  | 504 |  |  |  |  |  |  |  |  |
| 6 | Pb.c001502540.Contig1 | 1795 | - | 1145607 | 1147408 | cRMPc11056 | 2734 | 231 | 1144678 | 1147412 |  |  |  |  |  |  |  |  |  |
| 6 | MALPY01384 | 5189 | + | 1147643 | 1153210 |  |  |  |  |  | 504 | 241 | 6000 |  |  |  |  |  |  |
| 6 | Pb.c001702784.Contig1 | 1603 | - | 1147998 | 1149580 |  |  |  |  |  |  |  |  |  |  |  |  |  |  |
| 6 | Pc.c001812275.Contig1 | 907 | - | 1149700 | 1150619 |  |  |  |  |  |  |  |  |  |  |  |  |  |  |
| 6 | Pb.c000001786.Contig1 | 5912 | - | 1150088 | 1156015 |  |  |  |  |  |  |  |  |  |  |  |  |  |  |
| 6 | Pc.c001404787.Contig1 | 2887 | - | 1150908 | 1153795 | cRMPc11057 | 8372 | 1025 | 1147643 | 1156015 |  |  |  |  |  |  |  |  |  |
| 6 | Pb.c001001441.Contig1 | 5422 | - | 1157040 | 1162438 |  |  |  |  |  |  |  |  |  |  |  |  |  |  |
| 6 | Pc.c000002563.Contig1 | 7925 | + | 1157298 | 1165225 |  |  |  |  |  |  |  |  |  |  |  |  |  |  |
| 6 | MALPY00225 - BP | 10163 | + | 1158410 | 1168477 |  |  |  |  |  |  | 241 |  |  |  |  |  |  |  |
| 13 | PcContig652 | 7429 | + | 1166693 | 1175352 |  |  |  |  |  |  |  |  |  |  |  |  |  |  |
| 13 | MALPY03352 | 690 | - | 1170613 | 1171309 |  |  |  |  |  |  | 85 | 2500 |  |  |  |  |  |  |
| 13 | MALPY01431 | 4776 | + | 1174599 | 1179366 |  |  |  |  |  | 81 | 85 | 800 |  |  |  |  |  |  |
| 13 | Pb.c001602385.Contig1.0 | 1760 | + | 1175790 | 1177547 |  |  |  |  |  |  |  |  |  |  |  |  |  |  |
| 13 | PcContig488 | 9301 | + | 1177149 | 1187607 |  |  |  |  |  |  |  |  |  |  |  |  |  |  |
| 13 | PbContig35 | 2506 | + | 1177904 | 1180284 |  |  |  |  |  |  |  |  |  |  |  |  |  |  |
| 13 | MALPY03106 | 422 | + | 1179747 | 1180160 |  |  |  |  |  |  | 85 |  |  |  |  |  |  |  |
| 13 | MALPY01781 | 3965 | - | 1179824 | 1184075 |  |  |  |  |  | 81 |  |  |  |  |  |  |  |  |
| 13 | Pb.c000801782.Contig1 | 2731 | + | 1182746 | 1185725 |  |  |  |  |  |  |  |  |  |  |  |  |  |  |
| 13 | MALPY00643 | 15915 | - | 1183374 | 1200137 |  |  |  |  |  | 81 |  |  |  |  |  |  |  |  |
| 13 | Pb.c001502458.Contig1 | 2462 | + | 1186866 | 1189361 |  |  |  |  |  |  |  |  |  |  |  |  |  |  |
| 13 | Pb.c000002057.Contig1 | 1057 | + | 1192859 | 1193916 |  |  |  |  |  |  |  |  |  |  |  |  |  |  |
| 13 | Pb.c000001809.Contig1 | 8879 | + | 1193889 | 1203772 |  |  |  |  |  |  |  |  |  |  |  |  |  |  |
| 13 | Pc.c001304782.Contig1 | 780 | + | 1194124 | 1194904 |  |  |  |  |  |  |  |  |  |  |  |  |  |  |
| 13 | Pc.c001505042.Contig1 | 1878 | - | 1197632 | 1199567 |  |  |  |  |  |  |  |  |  |  |  |  |  |  |
| 13 | Pc.c000002640.Contig1 | 2600 | - | 1199621 | 1202673 |  |  |  |  |  |  |  |  |  |  |  |  |  |  |
| 13 | MALPY01942 | 3682 | + | 1200111 | 1204038 |  |  |  |  |  |  | 158 | 1900 |  |  |  |  |  |  |
| 13 | Pc.c000403227.Contig1 | 2230 | - | 1202821 | 1205090 | cRMPc11058 | 48050 | 1541 | 1157040 | 1205090 |  |  |  |  |  |  |  |  |  |
| 13 | MALPY00941 | 6880 | + | 1206631 | 1213582 |  |  |  |  |  |  | 158 |  |  |  |  |  |  |  |
| 13 | PbContig251 | 10945 | + | 1206686 | 1218054 |  |  |  |  |  |  |  |  |  |  |  |  |  |  |
| 13 | Pc.c001103820.Contig1 | 3121 | - | 1206717 | 1209837 |  |  |  |  |  |  |  |  |  |  |  |  |  |  |
| 13 | MALPY03202 | 472 | - | 1208051 | 1208523 |  |  |  |  |  |  | 127 | 6500 |  |  |  |  |  |  |
| 13 | Pc.c000102498.Contig1 | 3336 | - | 1212066 | 1215414 |  |  |  |  |  |  |  |  |  |  |  |  |  |  |
| 13 | MALPY01518 | 15480 | - | 1214750 | 1231334 |  |  |  |  |  | 95 | 127 |  |  |  |  |  |  |  |
| 13 | Pb.c000501450.Contig1 | 8788 | + | 1218774 | 1228937 |  |  |  |  |  |  |  |  |  |  |  |  |  |  |
| 13 | PcContig685 | 3263 | - | 1221755 | 1225678 |  |  |  |  |  |  |  |  |  |  |  |  |  |  |
| 13 | Pc.c000203339.Contig1 | 619 | - | 1228365 | 1228984 |  |  |  |  |  |  |  |  |  |  |  |  |  |  |
| 13 | Pb.c000501149.Contig1 | 5267 | + | 1228710 | 1233969 |  |  |  |  |  |  |  |  |  |  |  |  |  |  |
| 13 | PcContig686 | 3593 | - | 1228991 | 1232482 |  |  |  |  |  |  |  |  |  |  |  |  |  |  |
| man13 | MALPY01445 - Ins | 9356 | + | 1231391 | 1240705 |  |  |  |  |  | 95 | 109 |  |  |  |  |  |  |  |
| 13 | Pb.c000002059.Contig1 | 1245 | + | 1232789 | 1234046 |  |  |  |  |  |  |  |  |  |  |  |  |  |  |
| man13 | Pc.c001304195.Contig1 - Ins | 2845 | - | 1233933 | 1236921 |  |  |  |  |  |  |  |  |  |  |  |  |  |  |
| 13 | Pb.c000001765.Contig1 | 1578 | + | 1234024 | 1235602 |  |  |  |  |  |  |  |  |  |  |  |  |  |  |
| 13 | Pb.c001001775.Contig1 | 3767 | - | 1235902 | 1239684 |  |  |  |  |  |  |  |  |  |  |  |  |  |  |
| 13 | Pb.c000802049.Contig1 | 3608 | + | 1239091 | 1242659 |  |  |  |  |  |  |  |  |  |  |  |  |  |  |
| 13 | PcContig610 | 3983 | + | 1239574 | 1243955 |  |  |  |  |  |  |  |  |  |  |  |  |  |  |
| 13 | MALPY02355 | 2601 | + | 1240545 | 1243099 |  |  |  |  |  |  | 109 | 700 |  |  |  |  |  |  |
| 13 | MALPY03359 | 921 | - | 1243459 | 1244377 | cRMPc11059 | 37746 | 362 | 1206631 | 1244377 |  | 109 | 6500 |  |  |  |  |  |  |
| man13 | MALPY01426 | 3053 | - | 1244739 | 1247786 | cRMPc11060 | 3047 | 3972 | 1244739 | 1247786 |  |  |  |  |  |  |  |  |  |
| 13 | Pb.c001001822.Contig1 | 1371 | - | 1251758 | 1253096 |  |  |  |  |  |  |  |  |  |  |  |  |  |  |
| 13 | MALPY01532 | 4870 | + | 1252209 | 1257077 |  |  |  |  |  |  | 109 | 1400 |  |  |  |  |  |  |
| 13 | Pc.c001811701.Contig1 | 1794 | + | 1252242 | 1254036 |  |  |  |  |  |  |  |  |  |  |  |  |  |  |
| 13 | Pb.c000201566.Contig1 | 3118 | - | 1253416 | 1256582 |  |  |  |  |  |  |  |  |  |  |  |  |  |  |
| 13 | PcContig398 | 1829 | + | 1254313 | 1256190 | cRMPc11061 | 5319 | 331 | 1251758 | 1257077 |  |  |  |  |  |  |  |  |  |
| 13 | MALPY00435 | 8381 | - | 1257408 | 1265961 |  |  |  |  |  |  | 109 | 800 |  |  |  |  |  |  |
| 13 | Pb.c000801733.Contig1 | 2561 | - | 1257665 | 1260143 |  |  |  |  |  |  |  |  |  |  |  |  |  |  |
| 13 | Pc.c001204079.Contig1 | 1044 | + | 1258894 | 1259869 |  |  |  |  |  |  |  |  |  |  |  |  |  |  |
| 13 | Pc.c001204080.Contig1 | 1314 | + | 1259883 | 1261164 |  |  |  |  |  |  |  |  |  |  |  |  |  |  |
| 13 | Pc.c001002760.Contig2 | 2050 | - | 1263226 | 1265306 |  |  |  |  |  |  |  |  |  |  |  |  |  |  |
| 13 | Pb.c000201603.Contig1 | 6297 | - | 1263376 | 1269866 |  |  |  |  |  |  |  |  |  |  |  |  |  |  |
| 13 | MALPY00830 | 7080 | - | 1266112 | 1273633 |  |  |  |  |  |  | 109 |  |  |  |  |  |  |  |
| 13 | Pb.c001301433.Contig2 | 1967 | - | 1270627 | 1272594 | cRMPc11062 | 16225 | 2673 | 1257408 | 1273633 |  |  |  | cRMPs1118 | 128955 | 2673 | 1144678 | 1273633 |  |
| 13 | Pb.c001301753.Contig1 | 7570 | - | 1276306 | 1283879 |  |  |  |  |  |  |  |  |  |  |  |  |  |  |
| 13 | MALPY00641 | 15964 | - | 1277016 | 1294007 |  |  |  |  |  |  |  |  |  |  |  |  |  |  |
| 13 | Pc.c000202861.Contig1 | 1729 | + | 1279265 | 1280985 |  |  |  |  |  |  |  |  |  |  |  |  |  |  |
| 13 | Pb.c001703024.Contig1 | 562 | + | 1280176 | 1280737 |  |  |  |  |  |  |  |  |  |  |  |  |  |  |
| 13 | Pb.c001401787.Contig1 | 5026 | - | 1286745 | 1291777 |  |  |  |  |  |  |  |  |  |  |  |  |  |  |
| 13 | Pc.c000502398.Contig1 | 3397 | + | 1288468 | 1291897 |  |  |  |  |  |  |  |  |  |  |  |  |  |  |
| 13 | MALPY00104 | 13794 | + | 1292160 | 1307144 |  |  |  |  |  | 804 |  |  |  |  |  |  |  |  |
| 13 | MALPY03249 | 959 | - | 1292500 | 1293459 |  |  |  |  |  |  |  |  |  |  |  |  |  |  |
| 13 | Pb.c001201917.Contig1 | 6208 | - | 1293442 | 1300764 |  |  |  |  |  |  |  |  |  |  |  |  |  |  |
| 13 | Pc.c000902719.Contig1 | 5347 | - | 1296084 | 1301438 |  |  |  |  |  |  |  |  |  |  |  |  |  |  |
| 13 | PbContig46 | 23164 | + | 1301298 | 1326165 |  |  |  |  |  |  |  |  |  |  |  |  |  |  |
| 13 | MALPY01935 | 10554 | + | 1307222 | 1317391 |  |  |  |  |  | 804 |  |  |  |  |  |  |  |  |
| 13 | Pc.c001405504.Contig1 | 631 | + | 1308939 | 1309570 |  |  |  |  |  |  |  |  |  |  |  |  |  |  |
| 13 | Pc.c001003266.Contig1 | 2639 | + | 1309671 | 1312067 |  |  |  |  |  |  |  |  |  |  |  |  |  |  |
| 13 | Pc.c001404739.Contig1 | 1743 | + | 1313761 | 1315411 |  |  |  |  |  |  |  |  |  |  |  |  |  |  |
| 13 | PcContig89 | 4257 | - | 1316224 | 1320540 |  |  |  |  |  |  |  |  |  |  |  |  |  |  |
| 13 | MALPY00832 | 7183 | + | 1317985 | 1325521 |  |  |  |  |  | 804 |  |  |  |  |  |  |  |  |
| 13 | MALPY03467 | 177 | + | 1319495 | 1319672 |  |  |  |  |  |  | 76 | 800 |  |  |  |  |  |  |
| 13 | MALPY03340 | 505 | + | 1320137 | 1320642 |  |  |  |  |  |  | 76 | 6500 |  |  |  |  |  |  |
| 13 | Pc.c000703423.Contig1 | 2139 | - | 1320665 | 1322804 |  |  |  |  |  |  |  |  |  |  |  |  |  |  |
| 13 | Pc.c001104455.Contig1 | 652 | + | 1323363 | 1324015 | cRMPc11063 | 49859 | 225 | 1276306 | 1326165 |  |  |  |  |  |  |  |  |  |
| 13 | MALPY03029 | 554 | - | 1326390 | 1326944 | cRMPc11064 | 554 | 453 | 1326390 | 1326944 |  | 76 | 1900 |  |  |  |  |  |  |
| 13 | Pb.c002113480.Contig1 | 919 | - | 1327397 | 1328313 |  |  |  |  |  |  |  |  |  |  |  |  |  |  |
| 13 | MALPY02254 | 3343 | - | 1328226 | 1331197 |  |  |  |  |  |  | 76 | 500 |  |  |  |  |  |  |
| 13 | Pb.c001001676.Contig1 | 4018 | - | 1330669 | 1334757 |  |  |  |  |  |  |  |  |  |  |  |  |  |  |
| 13 | MALPY01995 | 2910 | + | 1331618 | 1334516 |  |  |  |  |  |  | 76 |  |  |  |  |  |  |  |
| 13 | Pc.c001605843.Contig1 | 2282 | - | 1332560 | 1334797 | cRMPc11065 | 7400 | 4026 | 1327397 | 1334797 |  |  |  | cRMPs1119 | 58491 | 4026 | 1276306 | 1334797 |  |
| JC13 | MALPY00418 | 3935 | - | 1338823 | 1342761 | cRMPc11066 | 3938 | 358 | 1338823 | 1342761 | 1142 |  |  |  |  |  |  |  |  |
| 13 | MALPY00312 | 9225 | + | 1343119 | 1352047 |  |  |  |  |  | 1142 |  |  |  |  |  |  |  |  |
| 13 | PbContig312 | 7563 | - | 1345420 | 1353367 |  |  |  |  |  |  |  |  |  |  |  |  |  |  |
| 13 | Pc.c001605977.Contig1 | 2004 | - | 1348469 | 1350266 |  |  |  |  |  |  |  |  |  |  |  |  |  |  |
| 13 | MALPY02361 | 2094 | - | 1351249 | 1353343 | cRMPc11067 | 10248 | 1907 | 1343119 | 1353367 |  |  |  | cRMPs1120 | 14544 | 1907 | 1338823 | 1353367 |  |
| i13 | Pc.c000603008.Contig1 | 1581 | - | 1355274 | 1356855 |  |  |  |  |  |  |  |  |  |  |  |  |  |  |
| i13 | Pb.c001602000.Contig1 | 7771 | + | 1355590 | 1363451 |  |  |  |  |  |  |  |  |  |  |  |  |  |  |
| i13 | MALPY01175 | 7582 | + | 1355987 | 1363408 |  |  |  |  |  |  | 182 | 4000 |  |  |  |  |  |  |
| i13 | Pc.c000603535.Contig1 | 1793 | - | 1357039 | 1358967 |  |  |  |  |  |  |  |  |  |  |  |  |  |  |
| i13 | Pc.c001809966.Contig1 | 2135 | + | 1359546 | 1361684 | cRMPc11068 | 8177 | 4654 | 1355274 | 1363451 |  |  |  |  |  |  |  |  |  |
| i13 | Pb.c000101429.Contig1 | 9630 | + | 1368105 | 1377846 |  |  |  |  |  |  |  |  |  |  |  |  |  |  |
| i13 | MALPY02290 | 3334 | - | 1368163 | 1371491 |  |  |  |  |  | 403 | 182 |  |  |  |  |  |  |  |
| i13 | Pc.c000002537.Contig1 | 3852 | + | 1368411 | 1372257 |  |  |  |  |  |  |  |  |  |  |  |  |  |  |
| i13 | MALPY00075 | 4006 | - | 1371865 | 1375818 |  |  |  |  |  | 403 |  |  |  |  |  |  |  |  |
| i13 | Pc.c000002533.Contig1 | 2111 | + | 1372680 | 1374796 |  |  |  |  |  |  |  |  |  |  |  |  |  |  |
| i13 | MALPY03111 | 1586 | + | 1375583 | 1377169 | cRMPc11069 | 9741 | 798 | 1368105 | 1377846 | 403 | 177 | 2300 |  |  |  |  |  |  |
| i13 | MALPY02288 | 2637 | - | 1378644 | 1381428 |  |  |  |  |  | 76 | 177 |  |  |  |  |  |  |  |
| i13 | Pb.c000701247.Contig1 | 6414 | - | 1378810 | 1386227 |  |  |  |  |  |  |  |  |  |  |  |  |  |  |
| i13 | Pc.c001505123.Contig1 | 2079 | + | 1379665 | 1381759 |  |  |  |  |  |  |  |  |  |  |  |  |  |  |
| i13 | MALPY00812 | 4415 | - | 1381353 | 1386593 |  |  |  |  |  | 76 |  |  |  |  |  |  |  |  |
| i13 | Pc.c000503036.Contig1 | 2922 | + | 1381772 | 1385259 |  |  |  |  |  |  |  |  |  |  |  |  |  |  |
| iman13 | MALPY01389 - Ins | 14817 | - | 1385204 | 1401178 |  |  |  |  |  | 76 |  |  |  |  |  |  |  |  |
| i13 | Pc.c000502989.Contig1 | 1627 | + | 1386059 | 1387686 |  |  |  |  |  |  |  |  |  |  |  |  |  |  |
| i13 | Pb.c002006841.Contig1 | 1033 | - | 1386348 | 1387378 |  |  |  |  |  |  |  |  |  |  |  |  |  |  |
| 13 | Pb.c001001773.Contig1 | 4777 | + | 1395434 | 1400249 | cRMPc11070 | 22534 | 650 | 1378644 | 1401178 |  |  |  |  |  |  |  |  |  |
| 13 | Pb.c000501389.Contig1 | 2787 | - | 1401828 | 1404765 |  |  |  |  |  |  |  |  |  |  |  |  |  |  |
| 13 | MALPY03191 | 1779 | - | 1402977 | 1404917 | cRMPc11071 | 3089 | 639 | 1401828 | 1404917 | 76 |  |  |  |  |  |  |  |  |
| 13 | MALPY00255 | 23991 | + | 1405556 | 1430539 |  |  |  |  |  | 76 |  |  |  |  |  |  |  |  |
| 13 | Pb.c000901398.Contig1 | 9045 | + | 1407022 | 1417066 |  |  |  |  |  |  |  |  |  |  |  |  |  |  |
| 13 | PcContig3 | 9828 | + | 1407196 | 1418080 |  |  |  |  |  |  |  |  |  |  |  |  |  |  |
| 13 | Pb.c000401512.Contig1 | 2601 | + | 1417340 | 1419961 |  |  |  |  |  |  |  |  |  |  |  |  |  |  |
| 13 | Pc.c000203292.Contig1 | 1042 | + | 1418094 | 1419160 |  |  |  |  |  |  |  |  |  |  |  |  |  |  |
| 13 | Pb.c002112085.Contig1 | 5891 | + | 1423770 | 1429531 |  |  |  |  |  |  |  |  |  |  |  |  |  |  |
| 13 | Pc.c001404906.Contig1 | 1727 | + | 1424311 | 1426077 |  |  |  |  |  |  |  |  |  |  |  |  |  |  |
| 13 | Pc.c000402848.Contig1 | 5337 | + | 1426121 | 1431475 |  |  |  |  |  |  |  |  |  |  |  |  |  |  |
| 13 | PbContig244 | 10732 | + | 1429795 | 1442139 |  |  |  |  |  |  |  |  |  |  |  |  |  |  |
| 13 | MALPY00849 | 5318 | - | 1430529 | 1436327 |  |  |  |  |  | 76 |  |  |  |  |  |  |  |  |
| 13 | Pc.c000403324.Contig1 | 1291 | - | 1432613 | 1433883 |  |  |  |  |  |  |  |  |  |  |  |  |  |  |
| 13 | MALPY03364 | 893 | - | 1433365 | 1434243 |  |  |  |  |  |  | 83 | 2500 |  |  |  |  |  |  |
| 13 | Pc.c000503020.Contig1 | 2317 | - | 1434033 | 1436717 |  |  |  |  |  |  |  |  |  |  |  |  |  |  |
| 13 | MALPY00754 | 4926 | - | 1437399 | 1442290 |  |  |  |  |  | 76 | 83 | 2000 |  |  |  |  |  |  |
| 13 | Pc.c001304338.Contig1 | 4130 | - | 1437996 | 1442120 | cRMPc11072 | 36734 | 1387 | 1405556 | 1442290 |  |  |  |  |  |  |  |  |  |
| man13 | MALPY02501 - Ins | 9700 | - | 1443677 | 1453314 |  |  |  |  |  | 1342 | 83 |  |  |  |  |  |  |  |
| man13 | PbContig129 - Ins | 7944 | - | 1444582 | 1452448 |  |  |  |  |  |  |  |  |  |  |  |  |  |  |
| 13 | Pc.c001304661.Contig1 | 658 | - | 1445452 | 1446107 |  |  |  |  |  |  |  |  |  |  |  |  |  |  |
| 13 | Pc.c001304744.Contig1 | 1265 | - | 1446234 | 1447568 |  |  |  |  |  |  |  |  |  |  |  |  |  |  |
| man13 | Pc.c001103097.Contig2 - Ins | 3418 | - | 1447783 | 1450993 |  |  |  |  |  |  |  |  |  |  |  |  |  |  |
| 13 | MALPY03517 | 336 | - | 1448757 | 1449093 |  |  |  |  |  |  | 274 | 5000 |  |  |  |  |  |  |
| 13 | Pc.c001104023.Contig1 | 585 | - | 1451094 | 1451643 |  |  |  |  |  |  |  |  |  |  |  |  |  |  |
| 13 | PbContig351 | 18094 | + | 1453179 | 1470946 |  |  |  |  |  |  |  |  |  |  |  |  |  |  |
| man13 | MALPY00289 - Ins | 21027 | - | 1453535 | 1474162 |  |  |  |  |  | 1342 | 274 |  |  |  |  |  |  |  |
| 13 | MALPY03193 | 583 | - | 1455727 | 1456310 |  |  |  |  |  |  |  |  |  |  |  |  |  |  |
| 13 | Pc.c000002556.Contig1 | 2347 | - | 1457295 | 1459520 |  |  |  |  |  |  |  |  |  |  |  |  |  |  |
| 13 | Pc.c001404775.Contig1 | 2779 | + | 1467653 | 1470428 |  |  |  |  |  |  |  |  |  |  |  |  |  |  |
| 13 | Pb.c000101112.Contig1 | 19184 | + | 1472116 | 1491915 |  |  |  |  |  |  |  |  |  |  |  |  |  |  |
| 13 | Pc.c001204524.Contig1 | 1096 | + | 1472727 | 1473820 |  |  |  |  |  |  |  |  |  |  |  |  |  |  |
| 13 | Pc.c001204003.Contig1 | 2018 | + | 1473953 | 1475971 |  |  |  |  |  |  |  |  |  |  |  |  |  |  |
| 13 | MALPY00474 | 11538 | - | 1474721 | 1486411 |  |  |  |  |  | 103 |  |  |  |  |  |  |  |  |
| 13 | PcContig741 | 10504 | + | 1476007 | 1486664 |  |  |  |  |  |  |  |  |  |  |  |  |  |  |
| 13 | MALPY03213 | 347 | - | 1481293 | 1481640 |  |  |  |  |  |  | 152 | 4300 |  |  |  |  |  |  |
| 13 | MALPY02319 | 5450 | + | 1486922 | 1492327 |  |  |  |  |  | 103 | 152 |  |  |  |  |  |  |  |
| 13 | Pc.c001604984.Contig2 | 2777 | + | 1488270 | 1491054 |  |  |  |  |  |  |  |  |  |  |  |  |  |  |
| 13 | MALPY01689 | 4242 | - | 1491021 | 1495263 |  |  |  |  |  | 103 |  |  |  |  |  |  |  |  |
| 13 | Pc.c000602964.Contig1 | 1183 | - | 1491964 | 1493127 |  |  |  |  |  |  |  |  |  |  |  |  |  |  |
| 13 | Pb.c000501714.Contig1 | 923 | - | 1492488 | 1493411 |  |  |  |  |  |  |  |  |  |  |  |  |  |  |
| 13 | MALPY03322 | 125 | + | 1493061 | 1493186 |  |  |  |  |  |  | 56 | 2300 |  |  |  |  |  |  |
| 13 | Pc.c000703835.Contig1 | 1263 | - | 1493145 | 1494408 |  |  |  |  |  |  |  |  |  |  |  |  |  |  |
| 13 | PbContig133 | 4700 | - | 1493526 | 1497994 |  |  |  |  |  |  |  |  |  |  |  |  |  |  |
| 13 | PcContig737 | 2581 | - | 1494066 | 1496665 |  |  |  |  |  |  |  |  |  |  |  |  |  |  |
| 13 | MALPY00263 | 15580 | + | 1494995 | 1510786 |  |  |  |  |  | 103 | 56 - NS | 9000 |  |  |  |  |  |  |
| 13 | PbContig32 | 9588 | - | 1498549 | 1508371 |  |  |  |  |  |  |  |  |  |  |  |  |  |  |
| 13 | Pc.c000202890.Contig1 | 2003 | - | 1500348 | 1502369 |  |  |  |  |  |  |  |  |  |  |  |  |  |  |
| 13 | Pc.c000503513.Contig1 | 833 | - | 1502352 | 1503395 |  |  |  |  |  |  |  |  |  |  |  |  |  |  |
| 13 | Pc.c001505368.Contig1 | 576 | - | 1503498 | 1504071 |  |  |  |  |  |  |  |  |  |  |  |  |  |  |
| 13 | Pb.c001904428.Contig1 | 2572 | - | 1508216 | 1510788 |  |  |  |  |  |  |  |  |  |  |  |  |  |  |
| 13 | Pc.c000402890.Contig1 | 2136 | - | 1508665 | 1510801 | cRMPc11073 | 67124 | 8985 | 1443677 | 1510801 |  |  |  |  |  |  |  |  |  |
| 13 | MALPY03158 | 1766 | - | 1519786 | 1521552 | cRMPc11074 | 1766 | 1095 | 1519786 | 1521552 |  | 56 - NS | 1000 |  |  |  |  |  |  |
| 13 | MALPY01411 | 14185 | + | 1522647 | 1537532 |  |  |  |  |  |  | 56 | 1200 |  |  |  |  |  |  |
| 13 | Pb.c001702848.Contig1 | 2515 | + | 1523746 | 1526318 |  |  |  |  |  |  |  |  |  |  |  |  |  |  |
| 13 | Pb.c000801802.Contig1 | 3639 | - | 1531771 | 1535446 | cRMPc11075 | 14885 | 487 | 1522647 | 1537532 |  |  |  |  |  |  |  |  |  |
| 13 | MALPY01900 | 4707 | - | 1538019 | 1542891 |  |  |  |  |  | 928 | 56 |  |  |  |  |  |  |  |
| 13 | Pb.c001301902.Contig1 | 1574 | + | 1542282 | 1543853 |  |  |  |  |  |  |  |  |  |  |  |  |  |  |
| 13 | MALPY01899 | 4718 | + | 1543011 | 1547747 |  |  |  |  |  | 928 | 242 | 600 |  |  |  |  |  |  |
| 13 | Pc.c001504938.Contig1 | 2297 | + | 1544111 | 1546429 | cRMPc11076 | 9728 | 773 | 1538019 | 1547747 |  |  |  |  |  |  |  |  |  |
| 13 | MALPY02305 | 2494 | - | 1548520 | 1551109 | cRMPc11077 | 2589 | 310 | 1548520 | 1551109 |  | 242 | 1300 |  |  |  |  |  |  |
| man13 | MALPY00226 - Ins | 32431 | - | 1551419 | 1584644 |  |  |  |  |  | 198 | 242 |  |  |  |  |  |  |  |
| 13 | Pb.c001702844.Contig1 | 2013 | - | 1552184 | 1554281 |  |  |  |  |  |  |  |  |  |  |  |  |  |  |
| 13 | Pc.c001812528.Contig1 | 991 | - | 1553810 | 1554795 |  |  |  |  |  |  |  |  |  |  |  |  |  |  |
| 13 | Pb.c000401481.Contig1 | 2851 | + | 1554690 | 1557503 |  |  |  |  |  |  |  |  |  |  |  |  |  |  |
| 13 | Pc.c001204583.Contig1 | 1654 | - | 1556091 | 1557717 |  |  |  |  |  |  |  |  |  |  |  |  |  |  |
| man13 | Pb.c000901860.Contig1 - Ins | 8990 | + | 1562415 | 1571070 |  |  |  |  |  |  |  |  |  |  |  |  |  |  |
| 13 | Pc.c001605845.Contig1 | 3771 | - | 1563204 | 1567071 |  |  |  |  |  |  |  |  |  |  |  |  |  |  |
| 13 | Pb.c000801791.Contig1 | 9162 | + | 1571601 | 1580736 |  |  |  |  |  |  |  |  |  |  |  |  |  |  |
| 13 | Pc.c000802675.Contig1 | 4164 | - | 1571920 | 1576036 |  |  |  |  |  |  |  |  |  |  |  |  |  |  |
| 13 | Pc.c001708455.Contig1 | 1031 | + | 1576354 | 1577412 |  |  |  |  |  |  |  |  |  |  |  |  |  |  |
| 13 | Pb.c002006842.Contig1 | 2196 | + | 1583146 | 1585237 |  |  |  |  |  |  |  |  |  |  |  |  |  |  |
| TK13 | MALPY00860 | 4523 | + | 1584995 | 1589821 | cRMPc11078 | 38402 | 407 | 1551419 | 1589821 | 198 |  |  |  |  |  |  |  |  |
| 13 | MALPY02329 | 2784 | - | 1590228 | 1593015 |  |  |  |  |  | 198 |  |  |  |  |  |  |  |  |
| man13 | Pb.c000301396.Contig1 - Ins | 10435 | - | 1591493 | 1602938 |  |  |  |  |  |  |  |  |  |  |  |  |  |  |
| 13 | Pc.c001103869.Contig1 | 3871 | - | 1591980 | 1596007 |  |  |  |  |  |  |  |  |  |  |  |  |  |  |
| man13 | MALPY00775 - Ins | 8858 | - | 1593067 | 1602754 |  |  |  |  |  | 198 |  |  |  |  |  |  |  |  |
| man13 | Pc.c001404780.Contig1 - Ins | 1571 | - | 1596394 | 1598549 |  |  |  |  |  |  |  |  |  |  |  |  |  |  |
| 13 | Pc.c000903267.Contig1 | 1467 | + | 1600597 | 1602198 |  |  |  |  |  |  |  |  |  |  |  |  |  |  |
| 13 | MALPY02689 | 4320 | + | 1602895 | 1607203 |  |  |  |  |  | 198 | 150 | 2000 |  |  |  |  |  |  |
| 13 | PcContig566 | 2788 | + | 1602959 | 1605824 | cRMPc11079 | 16975 | 2275 | 1590228 | 1607203 |  |  |  |  |  |  |  |  |  |
| 13 | MALPY00797 | 11239 | - | 1609478 | 1620789 |  |  |  |  |  |  | 150 | 1400 |  |  |  |  |  |  |
| 13 | PcContig473 | 2343 | + | 1609893 | 1612200 |  |  |  |  |  |  |  |  |  |  |  |  |  |  |
| 13 | Pc.c001605940.Contig1 | 2958 | + | 1612891 | 1615527 |  |  |  |  |  |  |  |  |  |  |  |  |  |  |
| 13 | Pb.c000601242.Contig2 | 6579 | + | 1615134 | 1622351 |  |  |  |  |  |  |  |  |  |  |  |  |  |  |
| 13 | Pc.c001605906.Contig1 | 4365 | + | 1615622 | 1620544 | cRMPc11080 | 12873 | 121 | 1609478 | 1622351 |  |  |  |  |  |  |  |  |  |
| 13 | MALPY00956 | 3887 | + | 1622472 | 1626344 |  |  |  |  |  | 1112 | 150 |  |  |  |  |  |  |  |
| 13 | Pb.c001602858.Contig1 | 1475 | - | 1623544 | 1625013 |  |  |  |  |  |  |  |  |  |  |  |  |  |  |
| 13 | Pb.c001402163.Contig1 | 1132 | - | 1626156 | 1627288 |  |  |  |  |  |  |  |  |  |  |  |  |  |  |
| 13 | MALPY00955 | 3794 | + | 1626431 | 1630129 |  |  |  |  |  | 1112 |  |  |  |  |  |  |  |  |
| 13 | Pc.c000203326.Contig1 | 1153 | + | 1626678 | 1627817 |  |  |  |  |  |  |  |  |  |  |  |  |  |  |
| 13 | Pb.c001402161.Contig1 | 1290 | - | 1627231 | 1628512 |  |  |  |  |  |  |  |  |  |  |  |  |  |  |
| 13 | MALPY01579 | 6673 | - | 1630055 | 1637258 |  |  |  |  |  |  |  |  |  |  |  |  |  |  |
| 13 | Pc.c001204011.Contig1 | 2768 | - | 1631212 | 1634342 |  |  |  |  |  |  |  |  |  |  |  |  |  |  |
| 13 | Pc.c000402280.Contig1 | 2064 | - | 1635109 | 1637206 | cRMPc11081 | 14786 | 1063 | 1622472 | 1637258 |  |  |  | cRMPs1121 | 281984 | 1063 | 1355274 | 1637258 |  |
| 13 | MALPY01649 | 7981 | - | 1638321 | 1646952 |  |  |  |  |  | 449 |  |  |  |  |  |  |  |  |
| 13 | Pb.c001904331.Contig1 | 2200 | - | 1638416 | 1640631 |  |  |  |  |  |  |  |  |  |  |  |  |  |  |
| 13 | Pc.c000402829.Contig1 | 1804 | - | 1639345 | 1641227 |  |  |  |  |  |  |  |  |  |  |  |  |  |  |
| 13 | MALPY00892 | 5905 | + | 1646893 | 1652804 | cRMPc11082 | 14483 | 34 | 1638321 | 1652804 | 449 | 142 | 500 |  |  |  |  |  |  |
| 13 | MALPY01527 | 6199 | + | 1652838 | 1659248 |  |  |  |  |  | 192 | 142 | 6500 |  |  |  |  |  |  |
| 13 | Pc.c001304189.Contig1 | 2787 | + | 1653337 | 1656124 |  |  |  |  |  |  |  |  |  |  |  |  |  |  |
| 13 | Pb.c001301833.Contig1 | 6324 | + | 1655343 | 1662011 |  |  |  |  |  |  |  |  |  |  |  |  |  |  |
| 13 | PcContig168 | 5240 | + | 1656619 | 1662006 |  |  |  |  |  |  |  |  |  |  |  |  |  |  |
| TK13 | MALPY02033 | 5584 | + | 1659101 | 1664090 | cRMPc11083 | 11252 | 2393 | 1652838 | 1664090 | 192 |  |  |  |  |  |  |  |  |
| 13 | Pc.c001604986.Contig2 | 8933 | - | 1666483 | 1675777 |  |  |  |  |  |  |  |  |  |  |  |  |  |  |
| 13 | MALPY00023 | 10984 | - | 1667798 | 1678775 |  |  |  |  |  |  | 142 | 2300 |  |  |  |  |  |  |
| 13 | Pb.c000101364.Contig1 | 6287 | + | 1667931 | 1674639 |  |  |  |  |  |  |  |  |  |  |  |  |  |  |
| 13 | Pb.c000501424.Contig1 | 1989 | + | 1674504 | 1676526 |  |  |  |  |  |  |  |  |  |  |  |  |  |  |
| 13 | Pb.c000301427.Contig1 | 2368 | + | 1678636 | 1681022 |  |  |  |  |  |  |  |  |  |  |  |  |  |  |
| 13 | MALPY03068 | 972 | - | 1680086 | 1681076 |  |  |  |  |  |  | 142 | 3500 |  |  |  |  |  |  |
| 13 | MALPY02562 | 2594 | + | 1681027 | 1683678 |  |  |  |  |  |  |  |  |  |  |  |  |  |  |
| 13 | Pb.c001402084.Contig1 | 1945 | - | 1681233 | 1683202 |  |  |  |  |  |  |  |  |  |  |  |  |  |  |
| 13 | Pc.c000502942.Contig1 | 2761 | - | 1681327 | 1684184 | cRMPc11084 | 17701 | 471 | 1666483 | 1684184 |  |  |  |  |  |  |  |  |  |
| 13 | Pc.c000903963.Contig1 | 1040 | - | 1684655 | 1685689 |  |  |  |  |  |  |  |  |  |  |  |  |  |  |
| 13 | MALPY00874 | 19596 | - | 1684979 | 1705148 |  |  |  |  |  |  | 142 |  |  |  |  |  |  |  |
| 13 | Pb.c000501416.Contig1 | 2695 | + | 1686484 | 1689185 |  |  |  |  |  |  |  |  |  |  |  |  |  |  |
| 13 | MALPY03311 | 1245 | - | 1689230 | 1690514 |  |  |  |  |  |  |  |  |  |  |  |  |  |  |
| 13 | PbContig52 | 9709 | + | 1689358 | 1699083 |  |  |  |  |  |  |  |  |  |  |  |  |  |  |
| 13 | MALPY03060 | 1778 | - | 1689784 | 1691526 |  |  |  |  |  |  |  |  |  |  |  |  |  |  |
| 13 | PcContig238 | 3113 | + | 1690152 | 1693268 |  |  |  |  |  |  |  |  |  |  |  |  |  |  |
| 13 | PcContig483 | 2876 | + | 1693900 | 1696776 |  |  |  |  |  |  |  |  |  |  |  |  |  |  |
| 13 | Pb.c000801790.Contig1 | 4501 | + | 1702393 | 1706845 |  |  |  |  |  |  |  |  |  |  |  |  |  |  |
| 13 | MALPY01268 | 7880 | - | 1705481 | 1713330 | cRMPc11085 | 28675 | 456 | 1684655 | 1713330 | 208 |  |  |  |  |  |  |  |  |
| 13 | MALPY01851 | 5710 | - | 1713786 | 1719496 |  |  |  |  |  | 208 |  |  |  |  |  |  |  |  |
| 13 | Pb.c001702819.Contig1 | 3079 | + | 1716946 | 1720025 |  |  |  |  |  |  |  |  |  |  |  |  |  |  |
| 13 | Pc.c001103714.Contig1 | 1202 | - | 1718696 | 1719898 |  |  |  |  |  |  |  |  |  |  |  |  |  |  |
| 13 | MALPY01295 | 7987 | - | 1719782 | 1728896 |  |  |  |  |  | 208 |  |  |  |  |  |  |  |  |
| 13 | PbContig350 | 2589 | + | 1720265 | 1723100 |  |  |  |  |  |  |  |  |  |  |  |  |  |  |
| 13 | Pb.c001301814.Contig1 | 1311 | + | 1723902 | 1725330 |  |  |  |  |  |  |  |  |  |  |  |  |  |  |
| 13 | PbContig157 | 4583 | + | 1725616 | 1730471 |  |  |  |  |  |  |  |  |  |  |  |  |  |  |
| 13 | Pc.c000302999.Contig1 | 1320 | + | 1728302 | 1729838 | cRMPc11086 | 16685 | 315 | 1713786 | 1730471 |  |  |  |  |  |  |  |  |  |
| 13 | MALPY01279 | 6708 | + | 1730786 | 1737807 |  |  |  |  |  | 208 |  |  |  |  |  |  |  |  |
| 13 | Pb.c001802819.Contig1 | 5068 | + | 1731743 | 1737183 |  |  |  |  |  |  |  |  |  |  |  |  |  |  |
| 13 | Pc.c001505391.Contig1 | 625 | + | 1732645 | 1733309 |  |  |  |  |  |  |  |  |  |  |  |  |  |  |
| 13 | Pc.c000402783.Contig1 | 4010 | + | 1733637 | 1737690 | cRMPc11087 | 7021 | 1176 | 1730786 | 1737807 |  |  |  | cRMPs1122 | 99486 | 1176 | 1638321 | 1737807 |  |
| TK13 | MALPY01393 | 6364 | + | 1738983 | 1745309 |  |  |  |  |  | 735 |  |  |  |  |  |  |  |  |
| JC13 | MALPY02189 | 6289 | + | 1745265 | 1751554 |  |  |  |  |  | 735 |  |  |  |  |  |  |  |  |
| 13 | MALPY00081 | 16091 | + | 1749999 | 1766168 |  |  |  |  |  | 12 |  |  |  |  |  |  |  |  |
| 13 | Pb.c000701737.Contig1 | 2158 | + | 1762940 | 1765176 |  |  |  |  |  |  |  |  |  |  |  |  |  |  |
| 13 | MALPY03507 | 771 | + | 1763640 | 1764528 |  |  |  |  |  |  | 335 | 2400 |  |  |  |  |  |  |
| 13 | PbContig57 | 11406 | - | 1765305 | 1776539 |  |  |  |  |  |  |  |  |  |  |  |  |  |  |
| 13 | MALPY00303 | 15303 | + | 1766394 | 1781727 |  |  |  |  |  | 12 | 335 |  |  |  |  |  |  |  |
| 13 | Pc.c000302899.Contig1 | 3490 | - | 1767134 | 1770681 |  |  |  |  |  |  |  |  |  |  |  |  |  |  |
| man13 | MALPY01112 | 5069 | + | 1781427 | 1786496 |  |  |  |  |  | 12 |  |  |  |  |  |  |  | MALPY00303 |
| man13 | PbContig4630 | 3335 | + | 1783176 | 1786511 | cRMPc11088 | 47528 |  | 1738983 | 1786511 |  |  |  | cRMPs1123 | 47528 |  | 1738983 | 1786511 | \| |
| Telomere | |  |  |  |  |  |  |  |  |  |  |  |  |  |  |  |  |  |  |
|  |  | Pb Pc Py contig |  |  |  |  | cRMP contig | Gap to next cRMP contig |  |  |  |  |  |  | cRMP scaffold | Gap to next cRMP scaffold |  |  |  |
|  | Number | 707 |  |  |  | Number | 88 | 87 |  |  |  |  |  | Number | 23 | 22 |  |  |  |
|  | Average size (bp) | 4449 |  |  |  | Average size (bp) | 18487 | 1835 |  |  |  |  |  | Average size (bp) | 75127 | 2664 |  |  |  |
|  | Minimum size (bp) | 125 |  |  |  | Minimum size (bp) | 554 | 26 |  |  |  |  |  | Minimum size (bp) | 5368 | 281 |  |  |  |
|  | Maximum size (bp) | 42524 |  |  |  | Maximum size (bp) | 85173 | 8985 |  |  |  |  |  | Maximum size (bp) | 350301 | 8021 |  |  |  |
|  | Total size (bp) | 3145576 |  |  |  | Total size (bp) | 1626858 | 159653 |  |  |  |  |  | Total size (bp) | 1727914 | 58597 |  |  |  |
|  |  |  |  |  |  |  |  |  |  |  |  |  |  |  |  |  |  |  |  |
