# Supplementary material for: A Plasmodium Whole-Genome Synteny Map: Indels and Synteny Breakpoints as Foci for Species-Specific Genes
Source: PLoS Pathog. 2005 Dec 23;1(4):e44. doi: 10.1371/journal.ppat.0010044 (PMC1317653; doi:10.1371/journal.ppat.0010044)
Supplement: Supplementary file 12 [file ppat.0010044.st028.doc]

Table S28 - RMPchr12


# Table S28 - RMPchr12

|  |  |  |  |  |  |  |  |  |  |  |  |  |  |  |  |  |  |  |  |
| --- | --- | --- | --- | --- | --- | --- | --- | --- | --- | --- | --- | --- | --- | --- | --- | --- | --- | --- | --- |
| Table S28: Tiling path of RMP contigs combined to form cRMP chromosome 12. | | | | | | |  |  |  |  |  |  |  |  |  |  |  |  |  |
|  |  |  |  |  |  |  |  |  |  |  |  |  |  |  |  |  |  |  |  |
| Tiling paths of P. berghei (Pb), P. c. chabaudi (Pc) and P. y. yoelii (Py) contigs combined into composite rodent malaria parasite (cRMP) | | | | | | | | | |  |  |  |  |  |  |  |  |  |  |
| contigs aligned against parts of the 14 chromosomes of P. falciparum (Pf). The cRMP contigs are further linked by physical linkage | | | | | | | | | |  |  |  |  |  |  |  |  |  |  |
| and PCR (see Methods) to form cRMP scaffolds. Different synteny blocks with homology to different P. falciparum chromosomes | | | | | | | | | |  |  |  |  |  |  |  |  |  |  |
| together form the cRMP chromosomes. The tables also show gaps in the alignment (black lines), cRMP telomeres (white bars) and | | | | | | | | | |  |  |  |  |  |  |  |  |  |  |
| predicted centromeres (plum/black bars). Contigs, physical groups or PCR groups interrupted by inter- or intrasyntenic regions are | | | | | | | | | |  |  |  |  |  |  |  |  |  |  |
| printed in bold. A summary of the number of RMP contigs, cRMP contigs and cRMP scaffolds as well as their average, minimum, | | | | | | | | | |  |  |  |  |  |  |  |  |  |  |
| maximum and total sizes is provided. Column headings are as follows: | | | | |  |  |  |  |  |  |  |  |  |  |  |  |  |  |  |
|  |  |  |  |  |  |  |  |  |  |  |  |  |  |  |  |  |  |  |  |
| Pf chr.: P. falciparum chromosome to which the RMP contigs are aligned | | | | | |  |  |  |  |  |  |  |  |  |  |  |  |  |  |
| (#: PROmer analysis� from this study; JC# and TK#: Carlton et al.; man#: manual BLAST analysis; | | | | | | |  |  |  |  |  |  |  |  |  |  |  |  |  |
| i: inversed orientation of the synteny block; colours refer to those used in Fig. 2 of the paper); | | | | | | |  |  |  |  |  |  |  |  |  |  |  |  |  |
| Pb Pc Py contig: names of the aligned RMP contigs; | | |  |  |  |  |  |  |  |  |  |  |  |  |  |  |  |  |  |
| Size: size (bp) of the aligned RMP contigs; | |  |  |  |  |  |  |  |  |  |  |  |  |  |  |  |  |  |  |
| +/-: orientation of the aligned RMP contigs; | |  |  |  |  |  |  |  |  |  |  |  |  |  |  |  |  |  |  |
| Start contig & End contig: estimated start & end coordinates of alignment of the RMP contigs based on the coordinates | | | | | | | | |  |  |  |  |  |  |  |  |  |  |  |
| of PROmer hits corrected for the contig�s size and orientation and presence of inter- and intrasyntenic regions | | | | | | | |  |  |  |  |  |  |  |  |  |  |  |  |
| (the start position of the first RMP contig in the alignment was set at zero); | | | | | |  |  |  |  |  |  |  |  |  |  |  |  |  |  |
| cRMP contig: names of cRMP contigs | |  |  |  |  |  |  |  |  |  |  |  |  |  |  |  |  |  |  |
| (the first 2 digits refer to the cRMP chromosome number and the following 3 digits designate the order of the contigs); | | | | | | | | |  |  |  |  |  |  |  |  |  |  |  |
| cRMP contig size: size of cRMP contigs based on the corrected coordinates of alignment; | | | | | | |  |  |  |  |  |  |  |  |  |  |  |  |  |
| Gap size to next cRMP contig: size of gaps between neighbouring cRMP contigs; | | | | | | |  |  |  |  |  |  |  |  |  |  |  |  |  |
| Start cRMP contig & End cRMP contig: estimated start & end coordinates of alignment of the cRMP contigs; | | | | | | | |  |  |  |  |  |  |  |  |  |  |  |  |
| Phys. group (Py): physical linkage of neighbouring P. y. yoelii contigs; | | | | | |  |  |  |  |  |  |  |  |  |  |  |  |  |  |
| PCR group (Py): neighbouring P. y. yoelii contigs were linked previously or for this study by PCR; | | | | | | |  |  |  |  |  |  |  |  |  |  |  |  |  |
| PCR size (Py): size of the PCR product; | |  |  |  |  |  |  |  |  |  |  |  |  |  |  |  |  |  |  |
| cRMP scaffold: names of cRMP scaffolds | |  |  |  |  |  |  |  |  |  |  |  |  |  |  |  |  |  |  |
| (the first 2 digits refer to the cRMP chromosome number and the following 2 digits designate the order of the scaffolds); | | | | | | | | |  |  |  |  |  |  |  |  |  |  |  |
| cRMP scaffold size: size of cRMP scaffolds; | | |  |  |  |  |  |  |  |  |  |  |  |  |  |  |  |  |  |
| Gap size to next cRMP scaffold: size of gaps between neighbouring cRMP scaffolds; | | | | | | |  |  |  |  |  |  |  |  |  |  |  |  |  |
| Start cRMP scaffold & End cRMP scaffold: estimated start & end coordinates of alignment of the cRMP scaffolds; | | | | | | | | |  |  |  |  |  |  |  |  |  |  |  |
| Overlap: Linkage between RMP contigs established through manual BLAST analysis. | | | | | | |  |  |  |  |  |  |  |  |  |  |  |  |  |
|  |  |  |  |  |  |  |  |  |  |  |  |  |  |  |  |  |  |  |  |
| Pf chr. | Pb Pc Py contig | Size | +/- | Start contig | End contig | cRMP contig | cRMP contig size | Gap size to next cRMP contig | Start cRMP contig | End cRMP contig | Phys. group (Py) | PCR group (Py) | PCR size (Py) | cRMP scaffold | cRMP scaffold size | Gap size to next cRMP scaffold | Start cRMP scaffold | End cRMP scaffold | Overlap (manual) |
| Telomere | |  |  |  |  |  |  |  |  |  |  |  |  |  |  |  |  |  |  |
| man10 | MALPY01864 | 6451 | - | 0 | 6451 |  |  |  |  |  |  |  |  |  |  |  |  |  | \| |
| man10 | PbContig5452 | 6284 | - | 4765 | 11049 |  |  |  |  |  |  |  |  |  |  |  |  |  | MALPY00998 |
| 10 | MALPY00998 | 8707 | - | 6379 | 15198 |  |  |  |  |  | 577 |  |  |  |  |  |  |  |  |
| 10 | PcContig280 | 4455 | - | 6447 | 10902 |  |  |  |  |  |  |  |  |  |  |  |  |  |  |
| 10 | Pb.c000601615.Contig1 | 5210 | + | 11197 | 16428 |  |  |  |  |  |  |  |  |  |  |  |  |  |  |
| 10 | Pc.c001203983.Contig1 | 5521 | + | 11525 | 16927 |  |  |  |  |  |  |  |  |  |  |  |  |  |  |
| 10 | MALPY01823 | 6873 | + | 15156 | 22180 |  |  |  |  |  | 577 |  |  |  |  |  |  |  |  |
| 10 | Pb.c001101948.Contig1 | 1601 | + | 16284 | 18074 |  |  |  |  |  |  |  |  |  |  |  |  |  |  |
| 10 | PbContig104 | 4806 | + | 18138 | 22962 |  |  |  |  |  |  |  |  |  |  |  |  |  |  |
| 10 | Pc.c000803314.Contig1 | 1729 | + | 18970 | 20678 |  |  |  |  |  |  |  |  |  |  |  |  |  |  |
| 10 | MALPY01331 | 7583 | + | 20478 | 27832 |  |  |  |  |  |  | 98 | 3500 |  |  |  |  |  |  |
| 10 | Pc.c000803850.Contig1 | 1031 | + | 20709 | 21713 | cRMPc12001 | 27832 | 2165 | 0 | 27832 |  |  |  |  |  |  |  |  |  |
| 10 | Pb.c001502498.Contig1 | 5009 | - | 29997 | 35006 |  |  |  |  |  |  |  |  |  |  |  |  |  |  |
| 10 | MALPY01296 | 4630 | + | 31848 | 37069 | cRMPc12002 | 7072 | 31 | 29997 | 37069 |  | 98 | 1200 |  |  |  |  |  |  |
| 10 | MALPY00253 | 14876 | + | 37100 | 52555 |  |  |  |  |  | 583 | 98 |  |  |  |  |  |  |  |
| 10 | Pb.c000701600.Contig1 | 5912 | - | 39841 | 45321 |  |  |  |  |  |  |  |  |  |  |  |  |  |  |
| 10 | Pc.c001304170.Contig1 | 2224 | - | 40961 | 43179 |  |  |  |  |  |  |  |  |  |  |  |  |  |  |
| 10 | Pb.c001702870.Contig1 | 3838 | - | 49371 | 52970 |  |  |  |  |  |  |  |  |  |  |  |  |  |  |
| 10 | PcContig188 | 1512 | - | 50793 | 52073 | cRMPc12003 | 15870 | 1186 | 37100 | 52970 |  |  |  |  |  |  |  |  |  |
| 10 | MALPY01314 | 6121 | + | 54156 | 60399 |  |  |  |  |  | 583 |  |  |  |  |  |  |  |  |
| 10 | Pc.c000503045.Contig1 | 2084 | + | 55021 | 57114 |  |  |  |  |  |  |  |  |  |  |  |  |  |  |
| 10 | Pb.c001702830.Contig1 | 2644 | + | 55086 | 57739 |  |  |  |  |  |  |  |  |  |  |  |  |  |  |
| 10 | Pc.c001606516.Contig1 | 981 | + | 57025 | 58006 |  |  |  |  |  |  |  |  |  |  |  |  |  |  |
| 10 | Pb.c001904473.Contig1 | 3817 | + | 58367 | 62562 |  |  |  |  |  |  |  |  |  |  |  |  |  |  |
| 10 | MALPY02463 | 2252 | - | 60505 | 62757 |  |  |  |  |  | 583 |  |  |  |  |  |  |  |  |
| 10 | MALPY01720 | 6454 | + | 62029 | 68573 |  |  |  |  |  |  | 187 | 1300 |  |  |  |  |  |  |
| 10 | Pb.c000501512.Contig1 | 2131 | + | 62655 | 64822 |  |  |  |  |  |  |  |  |  |  |  |  |  |  |
| 10 | Pc.c000803255.Contig1 | 2938 | - | 63071 | 66078 |  |  |  |  |  |  |  |  |  |  |  |  |  |  |
| 10 | MALPY03520 | 549 | + | 64968 | 65517 |  |  |  |  |  |  |  |  |  |  |  |  |  |  |
| 10 | Pb.c002007428.Contig1 | 824 | - | 65149 | 65973 |  |  |  |  |  |  |  |  |  |  |  |  |  |  |
| 10 | Pc.c000803516.Contig1 | 1321 | - | 66221 | 67569 |  |  |  |  |  |  |  |  |  |  |  |  |  |  |
| 10 | Pb.c002112830.Contig1 | 1133 | - | 66293 | 67444 |  |  |  |  |  |  |  |  |  |  |  |  |  |  |
| 10 | PcContig767 | 5365 | + | 68297 | 78999 |  |  |  |  |  |  |  |  |  |  |  |  |  |  |
| 10 | Pb.c000301414.Contig1 | 5042 | - | 68786 | 79008 |  |  |  |  |  |  |  |  |  |  |  |  |  |  |
| 10 | MALPY02693 | 2325 | + | 69553 | 71878 |  |  |  |  |  |  | 187 | 6000 |  |  |  |  |  |  |
| 10 | Pc.c000403258.Contig1 | 620 | + | 70292 | 70912 | cRMPc12004 | 24852 | 2328 | 54156 | 79008 |  |  |  |  |  |  |  |  |  |
| 10 | PcContig344 | 6022 | + | 81336 | 87677 |  |  |  |  |  |  |  |  |  |  |  |  |  |  |
| 10 | PbContig398 | 3272 | - | 83421 | 86794 |  |  |  |  |  |  |  |  |  |  |  |  |  |  |
| 10 | MALPY01640 | 6098 | - | 83613 | 90238 |  |  |  |  |  | 91 | 187 |  |  |  |  |  |  |  |
| 10 | Pc.c000303049.Contig1 | 3743 | - | 83914 | 87663 |  |  |  |  |  |  |  |  |  |  |  |  |  |  |
| 10 | PbContig60 | 816 | - | 87140 | 87992 |  |  |  |  |  |  |  |  |  |  |  |  |  |  |
| 10 | Pb.c000701551.Contig1 | 4906 | + | 89339 | 94305 |  |  |  |  |  |  |  |  |  |  |  |  |  |  |
| 10 | PcContig588 | 5342 | + | 90077 | 95530 |  |  |  |  |  |  |  |  |  |  |  |  |  |  |
| 10 | MALPY02291 | 4193 | - | 90599 | 94840 | cRMPc12005 | 14194 | 1393 | 81336 | 95530 | 91 | 183 | 1000 |  |  |  |  |  |  |
| 10 | Pb.c001001799.Contig1 | 5500 | - | 96923 | 102321 |  |  |  |  |  |  |  |  |  |  |  |  |  |  |
| 10 | MALPY01777 | 4255 | - | 96955 | 101198 |  |  |  |  |  |  | 183 |  |  |  |  |  |  |  |
| 10 | Pc.c000903270.Contig1 | 2435 | + | 97304 | 99763 | cRMPc12006 | 5398 | 763 | 96923 | 102321 |  |  |  | cRMPs1201 | 102321 | 763 | 0 | 102321 |  |
| 10 | PcContig682 | 2909 | - | 103084 | 105996 |  |  |  |  |  |  |  |  |  |  |  |  |  |  |
| 10 | Pb.c001101535.Contig2 | 5481 | - | 104553 | 110046 |  |  |  |  |  |  |  |  |  |  |  |  |  |  |
| 10 | MALPY02269 | 5660 | - | 105050 | 110716 |  |  |  |  |  | 776 |  |  |  |  |  |  |  |  |
| 10 | Pc.c001304735.Contig1 | 1408 | - | 109489 | 110897 |  |  |  |  |  |  |  |  |  |  |  |  |  |  |
| 10 | MALPY00475 | 2826 | + | 110118 | 112944 |  |  |  |  |  | 776 |  |  |  |  |  |  |  |  |
| 10 | MALPY02780 | 2099 | + | 112567 | 114642 |  |  |  |  |  | 776 | 164 | 100 |  |  |  |  |  |  |
| 10 | PcContig416 | 4112 | - | 114162 | 118375 |  |  |  |  |  |  |  |  |  |  |  |  |  |  |
| 10 | MALPY02420 | 3425 | + | 114938 | 118222 |  |  |  |  |  |  | 164 |  |  |  |  |  |  |  |
| 10 | Pb.c001602399.Contig1 | 1382 | + | 114955 | 116343 |  |  |  |  |  |  |  |  |  |  |  |  |  |  |
| 10 | Pb.c002007779.Contig1 | 904 | + | 116352 | 117310 | cRMPc12007 | 15291 | 2397 | 103084 | 118375 |  |  |  | cRMPs1202 | 15291 | 2397 | 103084 | 118375 |  |
| 10 | Pb.c001904333.Contig1 | 2885 | - | 120772 | 123708 |  |  |  |  |  |  |  |  |  |  |  |  |  |  |
| 10 | MALPY00844 | 4386 | + | 121118 | 125559 |  |  |  |  |  | 23 |  |  |  |  |  |  |  |  |
| 10 | Pc.c001505657.Contig1 | 610 | + | 122423 | 123030 |  |  |  |  |  |  |  |  |  |  |  |  |  |  |
| 10 | Pb.c001202050.Contig1 | 5295 | - | 124752 | 130050 |  |  |  |  |  |  |  |  |  |  |  |  |  |  |
| 10 | MALPY00337 | 12236 | - | 126104 | 139099 |  |  |  |  |  | 23 | 36 | 900 |  |  |  |  |  |  |
| 10 | PcContig664 | 4093 | - | 126721 | 130728 |  |  |  |  |  |  |  |  |  |  |  |  |  |  |
| 10 | PcContig210 | 5168 | - | 127131 | 132264 |  |  |  |  |  |  |  |  |  |  |  |  |  |  |
| 10 | PcContig70 | 8542 | + | 138384 | 147371 |  |  |  |  |  |  |  |  |  |  |  |  |  |  |
| 10 | MALPY02261 | 14263 | - | 139604 | 153578 |  |  |  |  |  |  | 36 | 3000 |  |  |  |  |  |  |
| 10 | Pb.c001803464.Contig1 | 3690 | + | 142511 | 146180 |  |  |  |  |  |  |  |  |  |  |  |  |  |  |
| 10 | Pb.c002112394.Contig1 | 597 | + | 147088 | 147685 |  |  |  |  |  |  |  |  |  |  |  |  |  |  |
| 10 | Pb.c001904414.Contig1 | 2705 | + | 147803 | 150508 |  |  |  |  |  |  |  |  |  |  |  |  |  |  |
| 10 | Pc.c001203243.Contig2 | 2978 | + | 147826 | 150750 |  |  |  |  |  |  |  |  |  |  |  |  |  |  |
| 10 | Pc.c001303442.Contig2 | 4319 | + | 150496 | 154547 |  |  |  |  |  |  |  |  |  |  |  |  |  |  |
| 10 | PbContig186 | 3578 | + | 150881 | 154477 |  |  |  |  |  |  |  |  |  |  |  |  |  |  |
| 10 | MALPY03417 | 882 | - | 152947 | 153826 | cRMPc12008 | 33775 | 1331 | 120772 | 154547 |  |  |  |  |  |  |  |  |  |
| 10 | MALPY00009 | 10873 | + | 155878 | 167371 |  |  |  |  |  | 348 | 36 |  |  |  |  |  |  |  |
| 10 | Pb.c001904329.Contig1 | 2079 | - | 158947 | 161074 |  |  |  |  |  |  |  |  |  |  |  |  |  |  |
| 10 | Pb.c001502493.Contig1 | 2329 | - | 161697 | 164026 |  |  |  |  |  |  |  |  |  |  |  |  |  |  |
| 10 | PcContig75 | 13565 | + | 164183 | 180696 |  |  |  |  |  |  |  |  |  |  |  |  |  |  |
| 10 | Pb.c001502901.Contig1 | 819 | - | 164770 | 165583 |  |  |  |  |  |  |  |  |  |  |  |  |  |  |
| 10 | Pb.c001803538.Contig1 | 3493 | - | 167107 | 170819 |  |  |  |  |  |  |  |  |  |  |  |  |  |  |
| 10 | MALPY00965 | 2733 | + | 168092 | 171024 |  |  |  |  |  | 348 |  |  |  |  |  |  |  |  |
| 10 | Pb.c001803537.Contig1 | 1972 | - | 171677 | 173649 |  |  |  |  |  |  |  |  |  |  |  |  |  |  |
| 10 | MALPY02821 | 2635 | + | 171714 | 174349 |  |  |  |  |  | 348 | 28 | 2000 |  |  |  |  |  |  |
| 10 | PbContig121 | 5973 | + | 175012 | 181250 |  |  |  |  |  |  |  |  |  |  |  |  |  |  |
| 10 | MALPY00513 | 7053 | - | 176483 | 183617 |  |  |  |  |  |  | 28 | 900 |  |  |  |  |  |  |
| 10 | Pc.c000603463.Contig1 | 1141 | - | 180853 | 181885 | cRMPc12009 | 27739 | 957 | 155878 | 183617 |  |  |  |  |  |  |  |  |  |
| 10 | MALPY00002 | 12818 | + | 184574 | 197620 |  |  |  |  |  |  | 28 | 8000 |  |  |  |  |  |  |
| 10 | Pb.c001201946.Contig1 | 3847 | - | 185690 | 189480 |  |  |  |  |  |  |  |  |  |  |  |  |  |  |
| 10 | Pc.c000603061.Contig1 | 4980 | + | 185849 | 190552 |  |  |  |  |  |  |  |  |  |  |  |  |  |  |
| 10 | Pb.c000500709.Contig1 | 5177 | + | 191386 | 196819 |  |  |  |  |  |  |  |  |  |  |  |  |  |  |
| 10 | Pc.c000402946.Contig1 | 3134 | + | 192635 | 195826 |  |  |  |  |  |  |  |  |  |  |  |  |  |  |
| 10 | Pc.c000903904.Contig1 | 577 | + | 196308 | 196894 | cRMPc12010 | 13046 | 7572 | 184574 | 197620 |  |  |  |  |  |  |  |  |  |
| 10 | Pb.c000901795.Contig1 | 3208 | + | 205192 | 208415 |  |  |  |  |  |  |  |  |  |  |  |  |  |  |
| 10 | MALPY01288 | 4145 | - | 206644 | 210777 |  |  |  |  |  |  | 28 | 1700 |  |  |  |  |  |  |
| 10 | MALPY03543 | 775 | - | 207592 | 208385 |  |  |  |  |  |  |  |  |  |  |  |  |  |  |
| 10 | Pb.c000902070.Contig1 | 1063 | + | 208421 | 209484 | cRMPc12011 | 5585 | 326 | 205192 | 210777 |  |  |  |  |  |  |  |  |  |
| 10 | PbContig218 | 9450 | + | 211103 | 221198 |  |  |  |  |  |  |  |  |  |  |  |  |  |  |
| 10 | MALPY02579 | 6600 | + | 211912 | 219046 |  |  |  |  |  |  | 28 | 2000 |  |  |  |  |  |  |
| 10 | Pc.c000203252.Contig1 | 808 | - | 212936 | 213786 |  |  |  |  |  |  |  |  |  |  |  |  |  |  |
| 10 | Pc.c001405125.Contig1 | 808 | - | 214500 | 215350 |  |  |  |  |  |  |  |  |  |  |  |  |  |  |
| 10 | Pc.c000503056.Contig1 | 3879 | - | 216500 | 220154 |  |  |  |  |  |  |  |  |  |  |  |  |  |  |
| 10 | MALPY00207 | 20339 | + | 220415 | 241048 |  |  |  |  |  |  | 28 | 1200 |  |  |  |  |  |  |
| 10 | PbContig72 | 14976 | + | 220944 | 237023 |  |  |  |  |  |  |  |  |  |  |  |  |  |  |
| 10 | Pc.c000102660.Contig1 | 1370 | + | 225535 | 226897 |  |  |  |  |  |  |  |  |  |  |  |  |  |  |
| 10 | PcContig80 | 5381 | + | 228519 | 234252 |  |  |  |  |  |  |  |  |  |  |  |  |  |  |
| 10 | PcContig452 | 2360 | + | 236380 | 238740 |  |  |  |  |  |  |  |  |  |  |  |  |  |  |
| 10 | Pb.c001702931.Contig1 | 1543 | + | 236737 | 238265 |  |  |  |  |  |  |  |  |  |  |  |  |  |  |
| 10 | MALPY03418 | 736 | - | 238018 | 238757 |  |  |  |  |  |  |  |  |  |  |  |  |  |  |
| 10 | Pb.c001702932.Contig1 | 1416 | + | 238314 | 239730 | cRMPc12012 | 29945 | 1312 | 211103 | 241048 |  |  |  |  |  |  |  |  |  |
| 10 | MALPY01854 | 14222 | - | 242360 | 258020 |  |  |  |  |  |  | 28 |  |  |  |  |  |  |  |
| 10 | Pb.c000501529.Contig1 | 2381 | + | 242411 | 244807 |  |  |  |  |  |  |  |  |  |  |  |  |  |  |
| 10 | Pc.c000503534.Contig1 | 542 | + | 242588 | 243133 |  |  |  |  |  |  |  |  |  |  |  |  |  |  |
| 10 | Pb.c001201690.Contig1 | 4212 | - | 246783 | 251443 |  |  |  |  |  |  |  |  |  |  |  |  |  |  |
| 10 | Pb.c001401765.Contig1 | 3003 | - | 251685 | 254407 |  |  |  |  |  |  |  |  |  |  |  |  |  |  |
| 10 | Pc.c000203333.Contig1 | 1342 | + | 252307 | 253371 |  |  |  |  |  |  |  |  |  |  |  |  |  |  |
| 10 | PcContig578 | 2384 | + | 254913 | 257297 |  |  |  |  |  |  |  |  |  |  |  |  |  |  |
| 10 | Pb.c000001276.Contig1 | 5888 | - | 255599 | 261486 |  |  |  |  |  |  |  |  |  |  |  |  |  |  |
| 10 | MALPY00417 | 8778 | - | 256596 | 263783 |  |  |  |  |  | 205 |  |  |  |  |  |  |  |  |
| 10 | MALPY02994 | 554 | + | 257645 | 258199 |  |  |  |  |  |  |  |  |  |  |  |  |  |  |
| 10 | Pc.c001103833.Contig1 | 1210 | - | 259312 | 260555 |  |  |  |  |  |  |  |  |  |  |  |  |  |  |
| 10 | PbContig370 | 10688 | - | 259633 | 270398 |  |  |  |  |  |  |  |  |  |  |  |  |  |  |
| 10 | Pc.c001708403.Contig1 | 2284 | - | 260705 | 263066 |  |  |  |  |  |  |  |  |  |  |  |  |  |  |
| 10 | MALPY00696 | 20234 | - | 263896 | 285471 |  |  |  |  |  | 205 |  |  |  |  |  |  |  |  |
| 10 | Pb.c001301764.Contig1 | 4720 | - | 270829 | 275912 |  |  |  |  |  |  |  |  |  |  |  |  |  |  |
| 10 | Pc.c000603069.Contig1 | 2388 | + | 272003 | 274954 |  |  |  |  |  |  |  |  |  |  |  |  |  |  |
| 10 | PcContig302 | 6106 | - | 274838 | 282284 |  |  |  |  |  |  |  |  |  |  |  |  |  |  |
| 10 | Pb.c001702845.Contig1 | 3641 | - | 279946 | 283581 | cRMPc12013 | 43111 | 919 | 242360 | 285471 |  |  |  | cRMPs1203 | 164699 | 919 | 120772 | 285471 |  |
| 10 | Pb.c001201927.Contig1 | 6721 | + | 286390 | 293410 |  |  |  |  |  |  |  |  |  |  |  |  |  |  |
| 10 | MALPY01205 | 9815 | + | 287797 | 297623 |  |  |  |  |  | 809 |  |  |  |  |  |  |  |  |
| 10 | Pc.c000202769.Contig1 | 4635 | + | 287844 | 292750 |  |  |  |  |  |  |  |  |  |  |  |  |  |  |
| 10 | Pc.c000203275.Contig1 | 602 | + | 292835 | 293437 |  |  |  |  |  |  |  |  |  |  |  |  |  |  |
| 10 | Pb.c000701580.Contig1 | 4343 | + | 293512 | 297749 |  |  |  |  |  |  |  |  |  |  |  |  |  |  |
| 10 | PcContig232 | 861 | + | 296069 | 296930 |  |  |  |  |  |  |  |  |  |  |  |  |  |  |
| 10 | Pc.c000102493.Contig1 | 1059 | + | 297307 | 298366 |  |  |  |  |  |  |  |  |  |  |  |  |  |  |
| 10 | MALPY02073 | 3405 | - | 297602 | 301572 |  |  |  |  |  | 809 | 11 | 7000 |  |  |  |  |  |  |
| 10 | Pc.c000602908.Contig1 | 3530 | + | 298535 | 302420 |  |  |  |  |  |  |  |  |  |  |  |  |  |  |
| 10 | Pb.c001702831.Contig1 | 1649 | + | 299728 | 301672 | cRMPc12014 | 16030 | 6694 | 286390 | 302420 |  |  |  |  |  |  |  |  |  |
| 10 | MALPY00574 | 11953 | - | 309114 | 321240 |  |  |  |  |  | 284B | 11 | 1000 |  |  |  |  |  |  |
| 10 | Pb.c001602568.Contig1 | 2240 | - | 310289 | 312647 |  |  |  |  |  |  |  |  |  |  |  |  |  |  |
| 10 | PcContig312 | 1288 | - | 313177 | 314465 |  |  |  |  |  |  |  |  |  |  |  |  |  |  |
| 10 | Pb.c001702898.Contig1 | 4355 | + | 313207 | 317571 |  |  |  |  |  |  |  |  |  |  |  |  |  |  |
| 10 | Pc.c001203938.Contig1 | 2513 | - | 314833 | 317307 | cRMPc12015 | 12126 | 1645 | 309114 | 321240 |  |  |  |  |  |  |  |  |  |
| 10 | MALPY00751 | 6182 | - | 322885 | 329064 |  |  |  |  |  |  | 11 | 1200 |  |  |  |  |  |  |
| 10 | Pb.c000801852.Contig1 | 5137 | - | 325492 | 330614 |  |  |  |  |  |  |  |  |  |  |  |  |  |  |
| 10 | MALPY02351 | 5583 | - | 330181 | 336008 |  |  |  |  |  |  | 11 | 300 |  |  |  |  |  |  |
| 10 | Pb.c000601582.Contig1 | 4521 | + | 331357 | 335892 |  |  |  |  |  |  |  |  |  |  |  |  |  |  |
| 10 | Pc.c001811714.Contig1 | 670 | - | 331738 | 332411 | cRMPc12016 | 13123 | 772 | 322885 | 336008 |  |  |  |  |  |  |  |  |  |
| 10 | MALPY02487 | 2174 | - | 336780 | 338984 | cRMPc12017 | 2204 | 310 | 336780 | 338984 |  | 11 | 600 |  |  |  |  |  |  |
| 10 | Pb.c001402219.Contig1 | 2744 | - | 339294 | 342095 |  |  |  |  |  |  |  |  |  |  |  |  |  |  |
| 10 | MALPY00403 | 3418 | + | 339472 | 342941 |  |  |  |  |  |  | 11 | 1500 |  |  |  |  |  |  |
| 10 | MALPY03114 | 106 | - | 340812 | 340918 | cRMPc12018 | 3647 | 1237 | 339294 | 342941 |  |  |  |  |  |  |  |  |  |
| 10 | Pb.c001301806.Contig1 | 7411 | - | 344178 | 352267 |  |  |  |  |  |  |  |  |  |  |  |  |  |  |
| 10 | MALPY01022 | 4840 | - | 345729 | 350683 |  |  |  |  |  |  | 11 | 2500 |  |  |  |  |  |  |
| 10 | Pc.c000303082.Contig1 | 4874 | + | 346487 | 352147 | cRMPc12019 | 8089 | 178 | 344178 | 352267 |  |  |  |  |  |  |  |  |  |
| 10 | Pb.c001301877.Contig1 | 4890 | - | 352445 | 357305 |  |  |  |  |  |  |  |  |  |  |  |  |  |  |
| 10 | MALPY01072 | 7503 | + | 353635 | 361310 |  |  |  |  |  |  | 11 | 200 |  |  |  |  |  |  |
| 10 | Pc.c000903401.Contig1 | 1756 | + | 354292 | 356018 |  |  |  |  |  |  |  |  |  |  |  |  |  |  |
| 10 | Pb.c001904888.Contig1 | 1403 | + | 358694 | 360046 |  |  |  |  |  |  |  |  |  |  |  |  |  |  |
| 10 | Pc.c001505124.Contig1 | 2328 | - | 360931 | 363259 |  |  |  |  |  |  |  |  |  |  |  |  |  |  |
| 10 | MALPY01005 | 19721 | + | 361358 | 382989 |  |  |  |  |  | 201 | 11 |  |  |  |  |  |  |  |
| 10 | Pb.c001502562.Contig1 | 2644 | + | 362269 | 364833 |  |  |  |  |  |  |  |  |  |  |  |  |  |  |
| 10 | PcContig56 | 930 | - | 362938 | 363868 |  |  |  |  |  |  |  |  |  |  |  |  |  |  |
| 10 | PcContig48 | 3273 | - | 363770 | 367120 |  |  |  |  |  |  |  |  |  |  |  |  |  |  |
| 10 | Pb.c001803391.Contig1 | 4491 | + | 365646 | 370784 |  |  |  |  |  |  |  |  |  |  |  |  |  |  |
| 10 | PcContig7 | 4655 | - | 367739 | 372394 |  |  |  |  |  |  |  |  |  |  |  |  |  |  |
| 10 | Pb.c001502571.Contig1 | 4089 | + | 371310 | 375433 |  |  |  |  |  |  |  |  |  |  |  |  |  |  |
| 10 | Pc.c001505373.Contig1 | 1238 | + | 372611 | 373828 |  |  |  |  |  |  |  |  |  |  |  |  |  |  |
| 10 | Pc.c000703352.Contig1 | 1792 | + | 374007 | 375832 |  |  |  |  |  |  |  |  |  |  |  |  |  |  |
| 10 | Pb.c001602009.Contig2 | 9804 | + | 377775 | 388521 |  |  |  |  |  |  |  |  |  |  |  |  |  |  |
| 10 | PcContig576 | 6087 | + | 377806 | 384378 |  |  |  |  |  |  |  |  |  |  |  |  |  |  |
| 10 | MALPY00358 | 14556 | - | 382857 | 398052 |  |  |  |  |  | 201 | 144 | 1800 |  |  |  |  |  |  |
| 10 | Pc.c000903470.Contig1 | 1196 | + | 384786 | 385982 |  |  |  |  |  |  |  |  |  |  |  |  |  |  |
| 10 | Pc.c001405552.Contig1 | 861 | + | 386247 | 387108 |  |  |  |  |  |  |  |  |  |  |  |  |  |  |
| 10 | Pb.c000201564.Contig1 | 4037 | + | 389108 | 393064 |  |  |  |  |  |  |  |  |  |  |  |  |  |  |
| 10 | Pc.c001505570.Contig1 | 1636 | + | 389834 | 391470 |  |  |  |  |  |  |  |  |  |  |  |  |  |  |
| 10 | Pc.c001505629.Contig1 | 1182 | + | 391592 | 392783 |  |  |  |  |  |  |  |  |  |  |  |  |  |  |
| 10 | Pb.c002007524.Contig1 | 854 | - | 393012 | 393860 |  |  |  |  |  |  |  |  |  |  |  |  |  |  |
| 10 | Pc.c001505824.Contig1 | 905 | + | 393567 | 394505 |  |  |  |  |  |  |  |  |  |  |  |  |  |  |
| 10 | PbContig56 | 13561 | - | 394354 | 409198 |  |  |  |  |  |  |  |  |  |  |  |  |  |  |
| 10 | PcContig690 | 1463 | + | 394695 | 396206 |  |  |  |  |  |  |  |  |  |  |  |  |  |  |
| 10 | Pc.c001504933.Contig1 | 1037 | + | 396381 | 397436 |  |  |  |  |  |  |  |  |  |  |  |  |  |  |
| 10 | MALPY01626 | 5588 | + | 400503 | 406298 |  |  |  |  |  | 223 | 144 | 10000 |  |  |  |  |  |  |
| 10 | Pc.c000202740.Contig1 | 2794 | - | 400710 | 403663 |  |  |  |  |  |  |  |  |  |  |  |  |  |  |
| TK10 | MALPY02354 | 7182 | - | 407405 | 414534 | cRMPc12020 | 62089 | 1766 | 352445 | 414534 | 223 |  |  |  |  |  |  |  |  |
| 10 | MALPY01616 | 2341 | - | 416300 | 418665 |  |  |  |  |  | 80\_alt | 144 | 300 |  |  |  |  |  |  |
| 10 | Pb.c000901856.Contig1 | 8882 | + | 416543 | 425495 |  |  |  |  |  |  |  |  |  |  |  |  |  |  |
| 10 | MALPY01496 | 13076 | - | 418843 | 431807 |  |  |  |  |  |  | 144 | 500 |  |  |  |  |  |  |
| 10 | Pc.c001103698.Contig1 | 3671 | + | 424065 | 428051 | cRMPc12021 | 15507 | 1108 | 416300 | 431807 |  |  |  |  |  |  |  |  |  |
| 10 | MALPY00525 | 9992 | + | 432915 | 443282 |  |  |  |  |  |  | 144 | 1700 |  |  |  |  |  |  |
| 10 | PbContig40 | 6922 | + | 434873 | 441744 |  |  |  |  |  |  |  |  |  |  |  |  |  |  |
| 10 | PcContig351 | 3174 | - | 436810 | 439912 |  |  |  |  |  |  |  |  |  |  |  |  |  |  |
| 10 | Pb.c000401229.Contig1 | 1987 | + | 442219 | 444114 | cRMPc12022 | 11199 | 2199 | 432915 | 444114 |  |  |  |  |  |  |  |  |  |
| 10 | MALPY01464 | 17045 | + | 446313 | 464514 |  |  |  |  |  | 273 | 144 |  |  |  |  |  |  |  |
| 10 | Pb.c001502537.Contig1 | 4629 | + | 449488 | 454138 |  |  |  |  |  |  |  |  |  |  |  |  |  |  |
| 10 | Pc.c000103080.Contig1 | 687 | + | 452069 | 452750 |  |  |  |  |  |  |  |  |  |  |  |  |  |  |
| 10 | Pc.c000202885.Contig1 | 601 | + | 452309 | 452904 |  |  |  |  |  |  |  |  |  |  |  |  |  |  |
| 10 | PbContig246 | 7588 | - | 457585 | 465226 |  |  |  |  |  |  |  |  |  |  |  |  |  |  |
| 10 | Pc.c001003269.Contig1 | 3682 | + | 460113 | 463648 |  |  |  |  |  |  |  |  |  |  |  |  |  |  |
| 10 | MALPY01619 | 2842 | - | 464762 | 467604 |  |  |  |  |  | 273 | 157 | 4000 |  |  |  |  |  |  |
| 10 | Pb.c000401553.Contig1 | 1956 | - | 465243 | 467199 | cRMPc12023 | 21291 | 4422 | 446313 | 467604 |  |  |  |  |  |  |  |  |  |
| 10 | MALPY01127 | 6941 | + | 472026 | 478798 |  |  |  |  |  |  | 157 | 6000 |  |  |  |  |  |  |
| 10 | Pb.c002006918.Contig1 | 991 | - | 473022 | 474013 |  |  |  |  |  |  |  |  |  |  |  |  |  |  |
| 10 | Pc.c000003018.Contig1 | 917 | + | 473495 | 474463 |  |  |  |  |  |  |  |  |  |  |  |  |  |  |
| 10 | Pb.c001904373.Contig1 | 2210 | - | 474452 | 476576 |  |  |  |  |  |  |  |  |  |  |  |  |  |  |
| 10 | Pc.c000302894.Contig1 | 3249 | + | 474508 | 477743 | cRMPc12024 | 6772 | 4251 | 472026 | 478798 |  |  |  |  |  |  |  |  |  |
| man10 | MALPY02722 | 2098 | - | 483049 | 485147 |  |  |  |  |  | 11 |  |  |  |  |  |  |  | Pb.c002007041.Contig1 |
| 10 | Pb.c002007041.Contig1 | 2342 | + | 484622 | 486973 |  |  |  |  |  |  |  |  |  |  |  |  |  |  |
| 10 | MALPY02740 | 4032 | - | 485264 | 488894 |  |  |  |  |  | 11 | 157 |  |  |  |  |  |  |  |
| 10 | Pc.c000202771.Contig1 | 3579 | - | 486995 | 490557 |  |  |  |  |  |  |  |  |  |  |  |  |  |  |
| TK10 | MALPY01979 | 2265 | + | 488767 | 491151 |  |  |  |  |  | 11 |  |  |  |  |  |  |  |  |
| TK10 | MALPY02743 | 2476 | + | 491145 | 493666 |  |  |  |  |  | 11 |  |  |  |  |  |  |  |  |
| 10 | MALPY00875 | 7383 | + | 493348 | 500749 |  |  |  |  |  | 11 |  |  |  |  |  |  |  |  |
| 10 | Pb.c001803569.Contig1 | 2898 | + | 495957 | 498870 | cRMPc12025 | 17700 | 1023 | 483049 | 500749 |  |  |  |  |  |  |  |  |  |
| 10 | MALPY02739 | 3455 | + | 501772 | 504801 |  |  |  |  |  | 11 |  |  |  |  |  |  |  |  |
| 10 | MALPY01423 | 16727 | - | 504226 | 522966 |  |  |  |  |  | 11 |  |  |  |  |  |  |  |  |
| 10 | PcContig190 | 7595 | - | 505093 | 513173 |  |  |  |  |  |  |  |  |  |  |  |  |  |  |
| 10 | Pb.c000401157.Contig1 | 6874 | + | 505223 | 512411 |  |  |  |  |  |  |  |  |  |  |  |  |  |  |
| 10 | MALPY03518 | 345 | + | 510475 | 510820 |  |  |  |  |  |  |  |  |  |  |  |  |  |  |
| 10 | Pb.c000401159.Contig1 | 6295 | - | 514231 | 520880 |  |  |  |  |  |  |  |  |  |  |  |  |  |  |
| 10 | PcContig407 | 2827 | - | 514816 | 517971 |  |  |  |  |  |  |  |  |  |  |  |  |  |  |
| 10 | MALPY03266 | 707 | + | 517564 | 518292 |  |  |  |  |  |  |  |  |  |  |  |  |  |  |
| 10 | Pc.c001002736.Contig2 | 3460 | - | 518031 | 521492 |  |  |  |  |  |  |  |  |  |  |  |  |  |  |
| 10 | MALPY03267 | 846 | - | 518416 | 519259 | cRMPc12026 | 21194 | 299 | 501772 | 522966 |  |  |  |  |  |  |  |  |  |
| 10 | MALPY02564 | 2646 | - | 523265 | 525979 |  |  |  |  |  | 11 |  |  |  |  |  |  |  |  |
| 10 | Pb.c001401778.Contig2 | 3837 | + | 523609 | 527446 |  |  |  |  |  |  |  |  |  |  |  |  |  |  |
| 10 | Pc.c000803948.Contig1 | 833 | - | 524136 | 524969 |  |  |  |  |  |  |  |  |  |  |  |  |  |  |
| 10 | PcContig117 | 3030 | - | 525177 | 528735 |  |  |  |  |  |  |  |  |  |  |  |  |  |  |
| 10 | MALPY02281 | 8231 | + | 525893 | 534174 |  |  |  |  |  | 11 | 171 | 600 |  |  |  |  |  |  |
| JC10 | MALPY02941 | 2733 | + | 526882 | 529615 |  |  |  |  |  |  |  |  |  |  |  |  |  |  |
| 10 | Pb.c001502545.Contig1 | 4684 | + | 527845 | 532822 |  |  |  |  |  |  |  |  |  |  |  |  |  |  |
| 10 | Pc.c000703967.Contig1 | 700 | - | 528713 | 529413 |  |  |  |  |  |  |  |  |  |  |  |  |  |  |
| 10 | MALPY03419 | 695 | - | 529125 | 529820 |  |  |  |  |  |  |  |  |  |  |  |  |  |  |
| 10 | Pc.c000402851.Contig1 | 5356 | - | 530205 | 535519 |  |  |  |  |  |  |  |  |  |  |  |  |  |  |
| 10 | MALPY00409 - BP | 8908 | - | 534643 | 543470 |  |  |  |  |  | 216 | 171 |  |  |  |  |  |  |  |
| 10 | Pb.c001702793.Contig1 - BP | 4831 | + | 536725 | 541653 |  |  |  |  |  |  |  |  |  |  |  |  |  |  |
| 10 | PcContig202 | 2141 | - | 537023 | 539119 |  |  |  |  |  |  |  |  |  |  |  |  |  |  |
| i3 | Pc.c000503109.Contig1 | 1713 | - | 539438 | 541103 | cRMPc12027 | 20205 | 260 | 523265 | 543470 |  |  |  |  |  |  |  |  |  |
| i3 | MALPY00103 | 22975 | - | 543730 | 567877 |  |  |  |  |  | 216 | 293 | 6500 |  |  |  |  |  |  |
| i3 | Pc.c000202828.Contig1 | 5550 | - | 547476 | 553131 |  |  |  |  |  |  |  |  |  |  |  |  |  |  |
| i3 | Pb.c001803406.Contig1 | 2101 | + | 549182 | 551395 |  |  |  |  |  |  |  |  |  |  |  |  |  |  |
| i3 | Pb.c000501525.Contig1 | 3244 | + | 553562 | 556752 |  |  |  |  |  |  |  |  |  |  |  |  |  |  |
| i3 | Pc.c000902707.Contig1 | 4726 | + | 566465 | 571133 |  |  |  |  |  |  |  |  |  |  |  |  |  |  |
| i3 | Pb.c001803549.Contig1 | 1685 | + | 566697 | 568502 |  |  |  |  |  |  |  |  |  |  |  |  |  |  |
| i3 | MALPY02397 | 5482 | - | 568166 | 573460 |  |  |  |  |  |  |  |  |  |  |  |  |  |  |
| i3 | Pb.c002006961.Contig1 | 1433 | - | 571530 | 572960 | cRMPc12028 | 29730 | 546 | 543730 | 573460 |  |  |  |  |  |  |  |  |  |
| i3 | MALPY00630 | 3683 | + | 574006 | 577360 |  |  |  |  |  |  | 293 | 4000 |  |  |  |  |  |  |
| i3 | Pb.c000301409.Contig1 | 1083 | - | 574414 | 575497 |  |  |  |  |  |  |  |  |  |  |  |  |  |  |
| i3 | Pc.c000703898.Contig1 | 796 | - | 574866 | 575662 | cRMPc12029 | 3354 | 3980 | 574006 | 577360 |  |  |  |  |  |  |  |  |  |
| i3 | Pb.c002006782.Contig1 | 1738 | + | 581340 | 583048 |  |  |  |  |  |  |  |  |  |  |  |  |  |  |
| i3 | MALPY00175 | 10856 | + | 582773 | 594097 |  |  |  |  |  | 251 | 293 |  |  |  |  |  |  |  |
| i3 | PcContig61 | 7386 | - | 583012 | 590437 |  |  |  |  |  |  |  |  |  |  |  |  |  |  |
| i3 | Pb.c000401442.Contig1 | 2728 | - | 586711 | 589478 | cRMPc12030 | 12757 | 283 | 581340 | 594097 |  |  |  |  |  |  |  |  |  |
| i3 | MALPY00331 | 8921 | - | 594380 | 603921 |  |  |  |  |  | 251 |  |  |  |  |  |  |  |  |
| i3 | Pb.c000701230.Contig1 | 7886 | - | 595339 | 603969 |  |  |  |  |  |  |  |  |  |  |  |  |  |  |
| i3 | Pc.c000302916.Contig1 | 3441 | + | 595658 | 599096 |  |  |  |  |  |  |  |  |  |  |  |  |  |  |
| i3 | MALPY01078 | 24452 | - | 603462 | 628346 |  |  |  |  |  | 251 |  |  |  |  |  |  |  |  |
| i3 | Pb.c001001672.Contig1 | 5097 | - | 604098 | 609192 |  |  |  |  |  |  |  |  |  |  |  |  |  |  |
| i3 | PcContig773 | 4277 | + | 607760 | 612288 |  |  |  |  |  |  |  |  |  |  |  |  |  |  |
| i3 | PbContig265 | 3892 | - | 608912 | 612807 |  |  |  |  |  |  |  |  |  |  |  |  |  |  |
| i3 | PcContig640 | 3531 | + | 612391 | 616317 |  |  |  |  |  |  |  |  |  |  |  |  |  |  |
| i3 | Pb.c000501374.Contig1 | 4634 | + | 612682 | 617598 |  |  |  |  |  |  |  |  |  |  |  |  |  |  |
| i3 | Pb.c000001776.Contig1 | 1884 | - | 619562 | 621485 |  |  |  |  |  |  |  |  |  |  |  |  |  |  |
| i3 | Pc.c001003232.Contig1 | 3085 | - | 620612 | 624463 |  |  |  |  |  |  |  |  |  |  |  |  |  |  |
| i3 | Pb.c000001814.Contig1 | 6284 | - | 621330 | 628742 |  |  |  |  |  |  |  |  |  |  |  |  |  |  |
| i3 | Pc.c000403320.Contig1 | 832 | - | 624513 | 625345 |  |  |  |  |  |  |  |  |  |  |  |  |  |  |
| i3 | Pc.c001605841.Contig1 | 2864 | - | 625513 | 628644 |  |  |  |  |  |  |  |  |  |  |  |  |  |  |
| iman3 | MALPY00098 | 2702 | - | 628119 | 630821 |  |  |  |  |  | 251 |  |  |  |  |  |  |  | Pb.c000001814.Contig1 |
| iTK3 | MALPY00097 | 3498 | + | 630628 | 634085 |  |  |  |  |  | 251 |  |  |  |  |  |  |  |  |
| i3 | Pb.c001602578.Contig1 | 3272 | + | 632243 | 635515 |  |  |  |  |  |  |  |  |  |  |  |  |  |  |
| i3 | MALPY01109 | 8305 | + | 634454 | 642550 |  |  |  |  |  | 922 |  |  |  |  |  |  |  |  |
| i3 | PbContig170 | 4639 | + | 635972 | 640635 |  |  |  |  |  |  |  |  |  |  |  |  |  |  |
| i3 | Pc.c000402801.Contig1 | 1857 | + | 636157 | 638014 |  |  |  |  |  |  |  |  |  |  |  |  |  |  |
| i3 | Pc.c000903325.Contig1 | 2367 | + | 638136 | 640590 |  |  |  |  |  |  |  |  |  |  |  |  |  |  |
| i3 | Pb.c000101607.Contig1 | 2375 | + | 640894 | 643269 |  |  |  |  |  |  |  |  |  |  |  |  |  |  |
| i3 | MALPY00741 | 4624 | - | 642920 | 647727 |  |  |  |  |  | 922 | 66A | 1100 |  |  |  |  |  |  |
| i3 | Pb.c000401156.Contig4 | 4622 | - | 643836 | 648314 |  |  |  |  |  |  |  |  |  |  |  |  |  |  |
| i3 | MALPY00121 | 17518 | + | 647696 | 666179 |  |  |  |  |  |  |  |  |  |  |  |  |  |  |
| i3 | Pb.c000401452.Contig1 | 6490 | - | 648048 | 654475 |  |  |  |  |  |  |  |  |  |  |  |  |  |  |
| i3 | PcContig571 | 7138 | + | 655566 | 663075 |  |  |  |  |  |  |  |  |  |  |  |  |  |  |
| i3 | PbContig258 | 10504 | - | 655592 | 666326 |  |  |  |  |  |  |  |  |  |  |  |  |  |  |
| i3 | Pc.c000403307.Contig1 | 769 | + | 664219 | 664988 |  |  |  |  |  |  |  |  |  |  |  |  |  |  |
| i3 | Pc.c001707812.Contig1 | 1412 | + | 665079 | 666572 | cRMPc12031 | 72192 | 498 | 594380 | 666572 |  |  |  | cRMPs1204 | 380182 | 498 | 286390 | 666572 |  |
| i3 | Pb.c000001635.Contig1 | 2810 | - | 667070 | 669932 |  |  |  |  |  |  |  |  |  |  |  |  |  |  |
| i3 | MALPY00510 | 3944 | + | 667088 | 671051 |  |  |  |  |  |  | 206 | 1500 |  |  |  |  |  |  |
| i3 | Pc.c000602856.Contig1 | 2845 | - | 667342 | 670200 | cRMPc12032 | 3981 | 1794 | 667070 | 671051 |  |  |  |  |  |  |  |  |  |
| i3 | MALPY01667 | 9744 | + | 672845 | 682584 |  |  |  |  |  |  | 206 |  |  |  |  |  |  |  |
| i3 | MALPY00163 | 17915 | + | 681454 | 698540 |  |  |  |  |  | 944 |  |  |  |  |  |  |  |  |
| i3 | Pb.c000201600.Contig1 | 10522 | - | 688424 | 698381 |  |  |  |  |  |  |  |  |  |  |  |  |  |  |
| i3 | Pc.c001404010.Contig1 | 4255 | - | 688426 | 692639 |  |  |  |  |  |  |  |  |  |  |  |  |  |  |
| i3 | Pc.c000503439.Contig1 | 2350 | + | 692789 | 695139 |  |  |  |  |  |  |  |  |  |  |  |  |  |  |
| i3 | MALPY03508 | 874 | + | 693689 | 694563 | cRMPc12033 | 25695 | 559 | 672845 | 698540 |  |  |  |  |  |  |  |  |  |
| i3 | MALPY00494 | 2474 | + | 699099 | 701350 |  |  |  |  |  | 944 | 295 | 1200 |  |  |  |  |  |  |
| i3 | Pb.c001904902.Contig1 | 793 | + | 699276 | 700068 |  |  |  |  |  |  |  |  |  |  |  |  |  |  |
| i3 | Pb.c001904405.Contig1 | 1930 | + | 700043 | 701826 | cRMPc12034 | 2727 | 216 | 699099 | 701826 |  |  |  |  |  |  |  |  |  |
| i3 | MALPY02155 | 2264 | - | 702042 | 704021 |  |  |  |  |  |  | 295 | 1000 |  |  |  |  |  |  |
| i3 | Pb.c002112256.Contig1 | 2628 | + | 702219 | 704593 |  |  |  |  |  |  |  |  |  |  |  |  |  |  |
| i3 | PcContig60 | 2117 | + | 702821 | 704938 |  |  |  |  |  |  |  |  |  |  |  |  |  |  |
| i3 | MALPY00045 | 7530 | + | 704159 | 712435 |  |  |  |  |  | 888 | 295 |  |  |  |  |  |  |  |
| i3 | Pb.c000901430.Contig1 | 3208 | - | 706218 | 709543 |  |  |  |  |  |  |  |  |  |  |  |  |  |  |
| i3 | PcContig687 | 2332 | - | 710856 | 713188 |  |  |  |  |  |  |  |  |  |  |  |  |  |  |
| i3 | Pb.c001904328.Contig1 | 1592 | - | 711911 | 713629 |  |  |  |  |  |  |  |  |  |  |  |  |  |  |
| i3 | MALPY00863 | 3235 | + | 712711 | 716315 | cRMPc12035 | 14273 | 1985 | 702042 | 716315 | 888 |  |  | cRMPs1205 | 49245 | 1985 | 667070 | 716315 |  |
| i3 | MALPY03471 | 781 | + | 718300 | 719087 | cRMPc12036 | 787 | 2204 | 718300 | 719087 |  | 264 | 2300 |  |  |  |  |  |  |
| i3 | Pc.c000603034.Contig1 - BP | 3157 | + | 721291 | 724540 |  |  |  |  |  |  |  |  |  |  |  |  |  |  |
| i3 | Pb.c000001756.Contig1 - BP | 5898 | + | 721577 | 727447 |  |  |  |  |  |  |  |  |  |  |  |  |  |  |
| i3 | MALPY03554 | 689 | + | 722558 | 723244 |  |  |  |  |  |  | 264 |  |  |  |  |  |  |  |
| 7 | MALPY01588 | 25526 | - | 723189 | 751220 |  |  |  |  |  | 322 |  |  |  |  |  |  |  |  |
| 7 | PcContig646 | 3560 | + | 724976 | 728530 |  |  |  |  |  |  |  |  |  |  |  |  |  |  |
| 7 | PcContig524 | 4058 | + | 728068 | 732399 |  |  |  |  |  |  |  |  |  |  |  |  |  |  |
| 7 | Pb.c002005907.Contig1 | 2718 | - | 728395 | 731375 |  |  |  |  |  |  |  |  |  |  |  |  |  |  |
| 7 | Pb.c001602100.Contig1 | 5331 | - | 731509 | 736885 |  |  |  |  |  |  |  |  |  |  |  |  |  |  |
| 7 | Pc.c001505139.Contig1 | 1381 | + | 732808 | 734189 |  |  |  |  |  |  |  |  |  |  |  |  |  |  |
| 7 | Pc.c001003248.Contig1 | 2229 | + | 736838 | 739079 |  |  |  |  |  |  |  |  |  |  |  |  |  |  |
| 7 | Pb.c001602580.Contig1 | 3979 | - | 741714 | 745828 |  |  |  |  |  |  |  |  |  |  |  |  |  |  |
| 7 | Pc.c001505057.Contig1 | 1726 | + | 742864 | 744593 |  |  |  |  |  |  |  |  |  |  |  |  |  |  |
| 7 | Pc.c000102484.Contig1 | 4238 | + | 745593 | 749814 |  |  |  |  |  |  |  |  |  |  |  |  |  |  |
| 7 | Pb.c001904338.Contig1 | 2020 | - | 746009 | 748224 |  |  |  |  |  |  |  |  |  |  |  |  |  |  |
| 7 | MALPY02825 | 3021 | - | 750722 | 753737 |  |  |  |  |  | 322 |  |  |  |  |  |  |  |  |
| 7 | Pc.c000703821.Contig1 | 2203 | - | 750971 | 753171 | cRMPc12037 | 32446 | 1961 | 721291 | 753737 |  |  |  | cRMPs1206 | 35437 | 1961 | 718300 | 753737 |  |
| 7 | Pc.c001103755.Contig1 | 4460 | - | 755698 | 760116 | cRMPc12038 | 4418 | 2677 | 755698 | 760116 |  |  |  | cRMPs1207 | 4418 | 2677 | 755698 | 760116 |  |
| 7 | Pc.c000803490.Contig1 | 5440 | - | 762793 | 768233 |  |  |  |  |  |  |  |  |  |  |  |  |  |  |
| 7 | MALPY00552 | 10389 | + | 766361 | 776967 |  |  |  |  |  |  | 309 | 1400 |  |  |  |  |  |  |
| 7 | Pb.c001702314.Contig1 | 2417 | + | 767999 | 770531 |  |  |  |  |  |  |  |  |  |  |  |  |  |  |
| 7 | Pc.c000803772.Contig1 | 1278 | - | 768289 | 769567 |  |  |  |  |  |  |  |  |  |  |  |  |  |  |
| 7 | Pc.c000803909.Contig1 | 1436 | - | 769873 | 771375 |  |  |  |  |  |  |  |  |  |  |  |  |  |  |
| 7 | PcContig263 | 1714 | - | 771576 | 773293 |  |  |  |  |  |  |  |  |  |  |  |  |  |  |
| 7 | Pb.c000201241.Contig1 | 8719 | - | 771792 | 780520 |  |  |  |  |  |  |  |  |  |  |  |  |  |  |
| 7 | MALPY00824 | 11370 | - | 772514 | 783596 |  |  |  |  |  |  | 309 |  |  |  |  |  |  |  |
| 7 | Pc.c001304114.Contig1 | 2727 | - | 774728 | 777455 |  |  |  |  |  |  |  |  |  |  |  |  |  |  |
| 7 | Pb.c000701506.Contig1 | 858 | + | 776098 | 776956 |  |  |  |  |  |  |  |  |  |  |  |  |  |  |
| 7 | Pb.c000701507.Contig1 | 5420 | + | 776310 | 782449 |  |  |  |  |  |  |  |  |  |  |  |  |  |  |
| 7 | Pc.c000602340.Contig1 | 2036 | - | 776962 | 779070 |  |  |  |  |  |  |  |  |  |  |  |  |  |  |
| 7 | Pc.c000703800.Contig1 | 606 | - | 780360 | 780930 |  |  |  |  |  |  |  |  |  |  |  |  |  |  |
| 7 | Pc.c000402898.Contig1 | 4419 | - | 781544 | 786041 | cRMPc12039 | 23248 | 15816 | 762793 | 786041 |  |  |  | cRMPs1208 | 23248 | 15816 | 762793 | 786041 |  |
| 7 | MALPY01442 | 12204 | - | 801857 | 814269 |  |  |  |  |  | 114 |  |  |  |  |  |  |  |  |
| 7 | Pb.c001101517.Contig1 | 10684 | - | 810912 | 821613 |  |  |  |  |  |  |  |  |  |  |  |  |  |  |
| 7 | MALPY01508 | 7365 | + | 813858 | 821067 |  |  |  |  |  | 114 |  |  |  |  |  |  |  |  |
| 7 | Pc.c000503365.Contig1 | 2106 | + | 814036 | 816112 |  |  |  |  |  |  |  |  |  |  |  |  |  |  |
| 7 | Pc.c000503080.Contig1 | 1662 | + | 815979 | 817513 |  |  |  |  |  |  |  |  |  |  |  |  |  |  |
| 7 | PcContig341 | 6236 | + | 819723 | 826058 |  |  |  |  |  |  |  |  |  |  |  |  |  |  |
| 7 | MALPY01892 | 9330 | + | 821484 | 831216 |  |  |  |  |  | 114 |  |  |  |  |  |  |  |  |
| 7 | Pb.c000801719.Contig1 | 2265 | - | 822924 | 825267 |  |  |  |  |  |  |  |  |  |  |  |  |  |  |
| 7 | Pb.c000801720.Contig1 | 3595 | - | 825397 | 829154 |  |  |  |  |  |  |  |  |  |  |  |  |  |  |
| 7 | Pc.c001002707.Contig2.1 | 664 | + | 825583 | 826247 |  |  |  |  |  |  |  |  |  |  |  |  |  |  |
| 7 | Pc.c001505063.Contig1 | 4619 | + | 826464 | 831269 | cRMPc12040 | 29412 | 3308 | 801857 | 831269 |  |  |  | cRMPs1209 | 29412 | 3308 | 801857 | 831269 |  |
| 7 | Pc.c001604992.Contig1 | 1667 | + | 834577 | 836226 |  |  |  |  |  |  |  |  |  |  |  |  |  |  |
| 7 | PbContig221 | 10423 | - | 835411 | 846212 |  |  |  |  |  |  |  |  |  |  |  |  |  |  |
| 7 | MALPY00519 - BP | 16931 | - | 835576 | 851951 |  |  |  |  |  | 331 |  |  |  |  |  |  |  |  |
| 7 | Pc.c001103824.Contig1 | 1955 | - | 837195 | 839147 |  |  |  |  |  |  |  |  |  |  |  |  |  |  |
| 7 | Pc.c001104367.Contig1 | 1333 | - | 843731 | 845094 |  |  |  |  |  |  |  |  |  |  |  |  |  |  |
| 8 | Pb.c002007588.Contig1 | 586 | - | 847125 | 847711 |  |  |  |  |  |  |  |  |  |  |  |  |  |  |
| 8 | Pb.c001202033.Contig1 | 1372 | - | 849573 | 850936 |  |  |  |  |  |  |  |  |  |  |  |  |  |  |
| 8 | Pc.c001606366.Contig1 | 964 | - | 849867 | 850819 |  |  |  |  |  |  |  |  |  |  |  |  |  |  |
| 8 | Pc.c001203271.Contig1 | 8039 | - | 850871 | 858968 |  |  |  |  |  |  |  |  |  |  |  |  |  |  |
| 8 | MALPY02310 | 5796 | + | 852233 | 858077 |  |  |  |  |  | 331 |  |  |  |  |  |  |  |  |
| 8 | PbContig324 | 5737 | + | 852567 | 858289 |  |  |  |  |  |  |  |  |  |  |  |  |  |  |
| 8 | MALPY03431 | 1519 | - | 853548 | 855067 |  |  |  |  |  |  |  |  |  |  |  |  |  |  |
| 8 | MALPY03132 | 626 | - | 854705 | 855331 | cRMPc12041 | 24391 | 446 | 834577 | 858968 |  | 17 | 7000 |  |  |  |  |  |  |
| 8 | MALPY00365 | 4841 | - | 859414 | 863989 |  |  |  |  |  | 331 | 17 | 2500 |  |  |  |  |  |  |
| 8 | PcContig275 | 2657 | - | 859784 | 862441 |  |  |  |  |  |  |  |  |  |  |  |  |  |  |
| 8 | Pc.c000303423.Contig1 | 1006 | - | 862706 | 863730 | cRMPc12042 | 4575 | 2752 | 859414 | 863989 |  |  |  |  |  |  |  |  |  |
| 8 | Pb.c000901715.Contig1 | 6084 | + | 866741 | 873359 |  |  |  |  |  |  |  |  |  |  |  |  |  |  |
| man8 | MALPY01255 - Ins | 12238 | - | 867044 | 880668 |  |  |  |  |  | 206 | 17 |  |  |  |  |  |  |  |
| 8 | Pc.c000602357.Contig2 | 4523 | - | 867253 | 872246 |  |  |  |  |  |  |  |  |  |  |  |  |  |  |
| 8 | PbContig93 | 12013 | + | 874098 | 887063 |  |  |  |  |  |  |  |  |  |  |  |  |  |  |
| 8 | PcContig410 | 3856 | + | 876901 | 881147 |  |  |  |  |  |  |  |  |  |  |  |  |  |  |
| 8 | MALPY00283 | 8349 | - | 880902 | 889168 |  |  |  |  |  | 206 | 224 | 3500 |  |  |  |  |  |  |
| 8 | PcContig297 | 3374 | + | 883202 | 886767 |  |  |  |  |  |  |  |  |  |  |  |  |  |  |
| 8 | Pb.c000601581.Contig1 | 4466 | - | 887103 | 891563 |  |  |  |  |  |  |  |  |  |  |  |  |  |  |
| 8 | PcContig265 | 3999 | + | 890687 | 894698 |  |  |  |  |  |  |  |  |  |  |  |  |  |  |
| 8 | Pb.c001802857.Contig2 | 3214 | - | 892746 | 896200 |  |  |  |  |  |  |  |  |  |  |  |  |  |  |
| 8 | MALPY02622 | 7334 | + | 892747 | 900722 |  |  |  |  |  | 28 | 224 |  |  |  |  |  |  |  |
| 8 | MALPY02985 | 912 | + | 893203 | 894142 |  |  |  |  |  |  | 328 | 10000 |  |  |  |  |  |  |
| 8 | PcContig770 | 2945 | + | 895077 | 898221 |  |  |  |  |  |  |  |  |  |  |  |  |  |  |
| 8 | Pb.c001903726.Contig1 | 3303 | + | 896276 | 899973 |  |  |  |  |  |  |  |  |  |  |  |  |  |  |
| 8 | Pb.c001802857.Contig1 | 565 | - | 896371 | 897000 |  |  |  |  |  |  |  |  |  |  |  |  |  |  |
| 8 | Pb.c001201990.Contig1 | 5865 | - | 900407 | 906572 |  |  |  |  |  |  |  |  |  |  |  |  |  |  |
| 8 | MALPY01722 | 6206 | + | 900670 | 907167 |  |  |  |  |  | 28 | 328 |  |  |  |  |  |  |  |
| 8 | MALPY03382 | 843 | - | 902816 | 903626 | cRMPc12043 | 40426 | 675 | 866741 | 907167 |  | 106 | 4000 |  |  |  |  |  |  |
| 8 | PcContig175 | 10857 | - | 907842 | 918799 |  |  |  |  |  |  |  |  |  |  |  |  |  |  |
| 8 | Pb.c000901710.Contig1 | 11187 | - | 908056 | 919305 |  |  |  |  |  |  |  |  |  |  |  |  |  |  |
| 8 | MALPY00804 | 15594 | - | 909692 | 925264 |  |  |  |  |  |  | 106 | 900 |  |  |  |  |  |  |
| 8 | PbContig64 | 11649 | + | 919390 | 929909 |  |  |  |  |  |  |  |  |  |  |  |  |  |  |
| 8 | MALPY01443 | 10853 | - | 923745 | 935647 |  |  |  |  |  |  | 106 | 1700 |  |  |  |  |  |  |
| 8 | Pc.c001204652.Contig1 | 1287 | - | 927293 | 928577 |  |  |  |  |  |  |  |  |  |  |  |  |  |  |
| 8 | PcContig266 | 4136 | - | 929155 | 934222 |  |  |  |  |  |  |  |  |  |  |  |  |  |  |
| 8 | PbContig49 | 5415 | + | 930262 | 936567 | cRMPc12044 | 28725 | 1424 | 907842 | 936567 |  |  |  |  |  |  |  |  |  |
| 8 | MALPY01625 | 8132 | - | 937991 | 946105 |  |  |  |  |  |  | 106/new | 3000 |  |  |  |  |  |  |
| 8 | PcContig623 | 2012 | - | 942823 | 944835 |  |  |  |  |  |  |  |  |  |  |  |  |  |  |
| 8 | Pb.c001202073.Contig1 | 2670 | - | 942931 | 945443 |  |  |  |  |  |  |  |  |  |  |  |  |  |  |
| 8 | Pc.c000803884.Contig1 | 660 | - | 944925 | 945585 | cRMPc12045 | 8114 | 3000 | 937991 | 946105 |  |  |  |  |  |  |  |  |  |
| man8 | MALPY00951 | 7042 | + | 949105 | 956147 | cRMPc12046 | 7042 | 1500 | 949105 | 956147 |  | new | 1500 |  |  |  |  |  |  |
| man8 | MALPY01569 | 6003 | - | 957647 | 963650 | cRMPc12047 | 6003 | 3095 | 957647 | 963650 |  | new |  | cRMPs1210 | 129073 | 3095 | 834577 | 963650 |  |
| Centromere - gap 3095 bp | |  |  | 960000 |  |  |  |  |  |  |  |  |  |  |  |  |  |  |  |
| 8 | MALPY01784 | 4943 | + | 966745 | 971733 |  |  |  |  |  |  | new | 3000 |  |  |  |  |  |  |
| 8 | Pb.c000601506.Contig1 | 2121 | + | 966824 | 968912 |  |  |  |  |  |  |  |  |  |  |  |  |  |  |
| 8 | Pb.c000601800.Contig1 | 999 | + | 969029 | 970112 | cRMPc12048 | 4988 | 3971 | 966745 | 971733 |  |  |  |  |  |  |  |  |  |
| 8 | MALPY02595 | 3050 | - | 975704 | 978742 |  |  |  |  |  |  | 327/new | 4500 |  |  |  |  |  |  |
| 8 | Pb.c001702782.Contig1 | 3172 | + | 976408 | 979571 | cRMPc12049 | 3867 | 1317 | 975704 | 979571 |  |  |  |  |  |  |  |  |  |
| man8 | MALPY00258 | 3037 | - | 980888 | 983925 |  |  |  |  |  | 518 |  |  |  |  |  |  |  | Pb.c001803432.Contig1 |
| 8 | Pb.c001803432.Contig1 | 6206 | - | 981910 | 988285 |  |  |  |  |  |  |  |  |  |  |  |  |  |  |
| 8 | Pc.c001103770.Contig1 | 2067 | + | 983601 | 985833 |  |  |  |  |  |  |  |  |  |  |  |  |  |  |
| 8 | MALPY00107 | 6913 | + | 984067 | 991174 |  |  |  |  |  | 518 | 327 |  |  |  |  |  |  |  |
| 8 | Pc.c001304088.Contig1 | 6996 | + | 990078 | 997101 |  |  |  |  |  |  |  |  |  |  |  |  |  |  |
| 8 | MALPY01769 | 8454 | - | 990353 | 998885 |  |  |  |  |  | 518 |  |  |  |  |  |  |  |  |
| 8 | Pb.c001904316.Contig1 | 2316 | - | 996510 | 998784 |  |  |  |  |  |  |  |  |  |  |  |  |  |  |
| 8 | Pc.c000603072.Contig1 | 2942 | + | 997103 | 1000069 |  |  |  |  |  |  |  |  |  |  |  |  |  |  |
| 8 | MALPY02512 | 3865 | - | 998745 | 1002895 |  |  |  |  |  |  | 212 | 3500 |  |  |  |  |  |  |
| 8 | Pb.c001402183.Contig1 | 3049 | + | 1000275 | 1003651 |  |  |  |  |  |  |  |  |  |  |  |  |  |  |
| 8 | Pc.c001504961.Contig1 | 2319 | + | 1000362 | 1002681 |  |  |  |  |  |  |  |  |  |  |  |  |  |  |
| 8 | Pc.c001504960.Contig1 | 1563 | + | 1003162 | 1004707 |  |  |  |  |  |  |  |  |  |  |  |  |  |  |
| 8 | MALPY02149 | 3305 | + | 1003218 | 1006451 |  |  |  |  |  |  |  |  |  |  |  |  |  |  |
| 8 | Pb.c001502448.Contig1 | 6065 | + | 1004460 | 1010525 |  |  |  |  |  |  |  |  |  |  |  |  |  |  |
| 8 | MALPY00216 | 38432 | + | 1007110 | 1057706 |  |  |  |  |  |  | 212 | 1400 |  |  |  |  |  |  |
| 8 | PcContig228 | 4620 | + | 1008241 | 1012897 |  |  |  |  |  |  |  |  |  |  |  |  |  |  |
| 8 | Pb.c001502863.Contig1 | 1142 | + | 1011149 | 1012345 |  |  |  |  |  |  |  |  |  |  |  |  |  |  |
| 8 | PbContig185 | 19227 | - | 1012728 | 1034300 |  |  |  |  |  |  |  |  |  |  |  |  |  |  |
| 8 | PcContig780 | 962 | + | 1013269 | 1014305 |  |  |  |  |  |  |  |  |  |  |  |  |  |  |
| 8 | Pc.c000102744.Contig1 | 7692 | - | 1015822 | 1023627 |  |  |  |  |  |  |  |  |  |  |  |  |  |  |
| 8 | Pc.c000202842.Contig1 | 1278 | - | 1024606 | 1025881 |  |  |  |  |  |  |  |  |  |  |  |  |  |  |
| 8 | Pc.c000303049.Contig1 | 3743 | - | 1026326 | 1030075 |  |  |  |  |  |  |  |  |  |  |  |  |  |  |
| 8 | PcContig434 | 5438 | - | 1030255 | 1035976 |  |  |  |  |  |  |  |  |  |  |  |  |  |  |
| 8 | Pc.c000602836.Contig1 | 4685 | - | 1044130 | 1048785 |  |  |  |  |  |  |  |  |  |  |  |  |  |  |
| 8 | Pb.c000501533.Contig1 | 4712 | + | 1044852 | 1049537 |  |  |  |  |  |  |  |  |  |  |  |  |  |  |
| 8 | Pc.c000503026.Contig1 | 5919 | - | 1049563 | 1056592 |  |  |  |  |  |  |  |  |  |  |  |  |  |  |
| 8 | Pb.c001201914.Contig1 | 6492 | + | 1050243 | 1057732 | cRMPc12050 | 76844 | 568 | 980888 | 1057732 |  |  |  |  |  |  |  |  |  |
| 8 | MALPY01811 | 3517 | + | 1058300 | 1061582 |  |  |  |  |  | 368 | 212 |  |  |  |  |  |  |  |
| 8 | Pb.c000401449.Contig1 | 2520 | - | 1060412 | 1062770 |  |  |  |  |  |  |  |  |  |  |  |  |  |  |
| TK8 | MALPY02731 | 3662 | + | 1061196 | 1065089 |  |  |  |  |  | 368 |  |  |  |  |  |  |  |  |
| 8 | PbContig308 | 6380 | - | 1063801 | 1070399 |  |  |  |  |  |  |  |  |  |  |  |  |  |  |
| 8 | MALPY01816 | 3247 | - | 1065747 | 1068994 |  |  |  |  |  | 368 | 214 | 500 |  |  |  |  |  |  |
| 8 | Pc.c001304867.Contig1 | 646 | + | 1065796 | 1066439 |  |  |  |  |  |  |  |  |  |  |  |  |  |  |
| 8 | PcContig100 | 5665 | + | 1067402 | 1073790 |  |  |  |  |  |  |  |  |  |  |  |  |  |  |
| 8 | MALPY00635 | 4598 | + | 1069678 | 1074519 |  |  |  |  |  |  | 214 | 5000 |  |  |  |  |  |  |
| 8 | Pb.c000401479.Contig1 | 2882 | - | 1071157 | 1074258 | cRMPc12051 | 16219 | 614 | 1058300 | 1074519 |  |  |  |  |  |  |  |  |  |
| 8 | MALPY02076 | 4104 | - | 1075133 | 1079476 |  |  |  |  |  | 253B |  |  |  |  |  |  |  |  |
| 8 | Pb.c001001717.Contig1 | 5970 | - | 1077036 | 1084945 |  |  |  |  |  |  |  |  |  |  |  |  |  |  |
| 8 | MALPY02580 | 3486 | - | 1080815 | 1085074 |  |  |  |  |  | 38A | 214 |  |  |  |  |  |  |  |
| 8 | PcContig123 | 2121 | + | 1081587 | 1084376 |  |  |  |  |  |  |  |  |  |  |  |  |  |  |
| 8 | MALPY01293 | 20280 | + | 1085038 | 1106407 |  |  |  |  |  | 38A |  |  |  |  |  |  |  |  |
| 8 | Pb.c001301438.Contig1 | 9990 | + | 1086161 | 1097083 |  |  |  |  |  |  |  |  |  |  |  |  |  |  |
| 8 | Pc.c001404719.Contig1 | 1705 | - | 1091286 | 1093117 |  |  |  |  |  |  |  |  |  |  |  |  |  |  |
| 8 | MALPY03537 | 658 | - | 1096029 | 1096687 |  |  |  |  |  |  | 6 | 4600 |  |  |  |  |  |  |
| 8 | Pc.c000703335.Contig1 | 1783 | - | 1096511 | 1098291 |  |  |  |  |  |  |  |  |  |  |  |  |  |  |
| 8 | Pb.c000301179.Contig2 | 6350 | + | 1097079 | 1104025 |  |  |  |  |  |  |  |  |  |  |  |  |  |  |
| 8 | Pc.c000503228.Contig1 | 634 | - | 1099329 | 1099963 |  |  |  |  |  |  |  |  |  |  |  |  |  |  |
| man8 | Pb.c000401455.Contig1 | 3859 | + | 1104957 | 1111495 |  |  |  |  |  |  |  |  |  |  |  |  |  | MALPY01293/MALPY00136 |
| man8 | MALPY00136 | 2768 | - | 1109372 | 1112140 |  |  |  |  |  | 815 |  |  |  |  |  |  |  | Pb.c000401455.Contig1/Pb.c001402192.Contig1 |
| 8 | Pb.c001402192.Contig1 | 11042 | - | 1111569 | 1124040 |  |  |  |  |  |  |  |  |  |  |  |  |  |  |
| 8 | MALPY01089 | 6646 | - | 1111989 | 1119941 |  |  |  |  |  | 815 | 6 | 1000 |  |  |  |  |  |  |
| 8 | Pc.c000202741.Contig1 | 1662 | - | 1112157 | 1113822 |  |  |  |  |  |  |  |  |  |  |  |  |  |  |
| 8 | Pc.c001304498.Contig1 | 3945 | - | 1114588 | 1119041 |  |  |  |  |  |  |  |  |  |  |  |  |  |  |
| 8 | MALPY00717 | 18704 | + | 1121637 | 1140431 |  |  |  |  |  | 266 - Ins | 6 |  |  |  |  |  |  |  |
| 8 | PbContig137 | 11543 | - | 1125955 | 1137605 |  |  |  |  |  |  |  |  |  |  |  |  |  |  |
| 8 | PcContig378 | 5010 | - | 1127975 | 1133465 |  |  |  |  |  |  |  |  |  |  |  |  |  |  |
| 8 | Pc.c000102690.Contig1 | 1475 | + | 1133146 | 1134582 |  |  |  |  |  |  |  |  |  |  |  |  |  |  |
| 8 | Pc.c000102031.Contig2 | 3014 | + | 1139809 | 1142853 |  |  |  |  |  |  |  |  |  |  |  |  |  |  |
| 8 | Pb.c000701503.Contig1 | 2760 | - | 1140474 | 1143300 |  |  |  |  |  |  |  |  |  |  |  |  |  |  |
| 8 | MALPY00999 | 7231 | + | 1140931 | 1148985 |  |  |  |  |  | 266 - Ins |  |  |  |  |  |  |  |  |
| 8 | MALPY03540 | 170 | + | 1142332 | 1142499 |  |  |  |  |  |  |  |  |  |  |  |  |  |  |
| 8 | PcContig217 | 3778 | + | 1143649 | 1147744 |  |  |  |  |  |  |  |  |  |  |  |  |  |  |
| 8 | Pb.c000701505.Contig1 | 5908 | - | 1143856 | 1150117 | cRMPc12052 | 74984 | 3118 | 1075133 | 1150117 |  |  |  | cRMPs1211 | 183372 | 3118 | 966745 | 1150117 |  |
| 8 | Pb.c000101414.Contig1 | 3191 | - | 1153235 | 1156194 |  |  |  |  |  |  |  |  |  |  |  |  |  |  |
| 8 | MALPY03091 | 1772 | + | 1153336 | 1155111 |  |  |  |  |  | 1230 |  |  |  |  |  |  |  |  |
| 8 | MALPY01254 | 2015 | + | 1154721 | 1156664 |  |  |  |  |  | 1230 | 14 | 250 |  |  |  |  |  |  |
| man8 | MALPY01502 | 4457 | - | 1155843 | 1160300 |  |  |  |  |  | 1157 |  |  |  |  |  |  |  |  |
| 8 | Pb.c001301804.Contig1 | 6996 | - | 1156159 | 1163155 |  |  |  |  |  |  |  |  |  |  |  |  |  |  |
| 8 | Pb.c002112582.Contig1 | 1057 | - | 1156488 | 1157548 |  |  |  |  |  |  |  |  |  |  |  |  |  |  |
| 8 | MALPY03486 | 758 | - | 1156571 | 1157317 |  |  |  |  |  |  | 14 | 5000 |  |  |  |  |  |  |
| 8 | PcContig140 - BP | 5583 | - | 1160055 | 1165629 |  |  |  |  |  |  |  |  |  |  |  |  |  |  |
| 8 | MALPY02878 | 2221 | + | 1160216 | 1162437 |  |  |  |  |  | 1157 | 14 |  |  |  |  |  |  |  |
| 6 | MALPY00483 - BP | 7246 | - | 1162228 | 1169486 |  |  |  |  |  | 280 |  |  |  |  |  |  |  |  |
| 6 | Pb.c002006894.Contig1 | 4026 | - | 1168258 | 1172464 |  |  |  |  |  |  |  |  |  |  |  |  |  |  |
| 6 | Pc.c001708413.Contig1 | 1926 | + | 1168990 | 1171065 |  |  |  |  |  |  |  |  |  |  |  |  |  |  |
| 6 | MALPY03350 | 561 | + | 1169469 | 1170024 |  |  |  |  |  |  |  |  |  |  |  |  |  |  |
| 6 | MALPY01753 | 3510 | - | 1170075 | 1173585 |  |  |  |  |  | 280 | 139 | 1800 |  |  |  |  |  |  |
| 6 | Pc.c001204579.Contig1 | 2115 | + | 1171301 | 1173416 | cRMPc12053 | 20350 | 1267 | 1153235 | 1173585 |  |  |  |  |  |  |  |  |  |
| 6 | MALPY01838 | 5827 | - | 1174852 | 1180643 |  |  |  |  |  |  | 139 | 1900 |  |  |  |  |  |  |
| 6 | Pb.c001702911.Contig1 | 3408 | - | 1176526 | 1179981 |  |  |  |  |  |  |  |  |  |  |  |  |  |  |
| 6 | Pc.c001104385.Contig1 | 647 | - | 1176979 | 1177632 |  |  |  |  |  |  |  |  |  |  |  |  |  |  |
| 6 | Pc.c000302975.Contig1 | 1156 | - | 1178312 | 1179465 |  |  |  |  |  |  |  |  |  |  |  |  |  |  |
| 6 | Pb.c001001992.Contig1 | 1689 | - | 1180349 | 1182038 | cRMPc12054 | 7186 | 307 | 1174852 | 1182038 |  |  |  |  |  |  |  |  |  |
| 6 | Pc.c001103894.Contig1 | 5990 | + | 1182345 | 1188110 |  |  |  |  |  |  |  |  |  |  |  |  |  |  |
| 6 | MALPY01462 | 3004 | - | 1182521 | 1185525 |  |  |  |  |  | 238 | 139 |  |  |  |  |  |  |  |
| 6 | Pb.c001101878.Contig1 | 7015 | - | 1183542 | 1190343 |  |  |  |  |  |  |  |  |  |  |  |  |  |  |
| 6 | MALPY01143 | 3916 | - | 1184556 | 1188556 |  |  |  |  |  | 238 |  |  |  |  |  |  |  |  |
| 6 | MALPY03509 | 550 | - | 1187145 | 1187692 |  |  |  |  |  |  | 74 | 5000 |  |  |  |  |  |  |
| man6 | MALPY00766 | 3265 | - | 1188583 | 1191974 |  |  |  |  |  | 238 |  |  |  |  |  |  |  |  |
| 6 | Pb.c001702953.Contig1 | 5180 | - | 1190604 | 1195784 |  |  |  |  |  |  |  |  |  |  |  |  |  |  |
| 6 | MALPY01085 | 10371 | - | 1193694 | 1204371 |  |  |  |  |  |  | 74 | 2800 |  |  |  |  |  |  |
| 6 | PcContig219 | 3050 | - | 1194086 | 1197212 |  |  |  |  |  |  |  |  |  |  |  |  |  |  |
| 6 | Pb.c000701598.Contig1 | 3221 | - | 1196510 | 1199956 | cRMPc12055 | 22026 | 2174 | 1182345 | 1204371 |  |  |  |  |  |  |  |  |  |
| 6 | MALPY02226 | 5708 | + | 1206545 | 1211878 |  |  |  |  |  | 27 | 74 |  |  |  |  |  |  |  |
| 6 | PcContig49 | 3097 | + | 1206554 | 1209597 |  |  |  |  |  |  |  |  |  |  |  |  |  |  |
| 6 | Pb.c000201622.Contig1 | 7966 | - | 1206707 | 1214523 |  |  |  |  |  |  |  |  |  |  |  |  |  |  |
| 6 | Pc.c001404904.Contig1 | 1584 | + | 1209512 | 1211076 |  |  |  |  |  |  |  |  |  |  |  |  |  |  |
| TK6 | MALPY01775 | 4972 | + | 1214372 | 1217676 |  |  |  |  |  |  |  |  |  |  |  |  |  |  |
| 6 | PcContig224 | 5757 | - | 1217272 | 1223261 |  |  |  |  |  |  |  |  |  |  |  |  |  |  |
| 6 | PbContig94 | 3588 | + | 1218280 | 1221867 |  |  |  |  |  |  |  |  |  |  |  |  |  |  |
| JC6 | MALPY01774 | 2917 | + | 1219966 | 1222889 |  |  |  |  |  | 27 |  |  |  |  |  |  |  |  |
| 6 | MALPY02805 | 2435 | + | 1220108 | 1222728 | cRMPc12056 | 16716 | 1104 | 1206545 | 1223261 | 27 |  |  | cRMPs1212 | 70026 | 1104 | 1153235 | 1223261 |  |
| 6 | PcContig222 | 3564 | - | 1224365 | 1227971 | cRMPc12057 | 3606 | 4161 | 1224365 | 1227971 |  |  |  | cRMPs1213 | 3606 | 4161 | 1224365 | 1227971 |  |
| 6 | Pc.c001813126.Contig1 | 1201 | - | 1232132 | 1233333 |  |  |  |  |  |  |  |  |  |  |  |  |  |  |
| 6 | MALPY00440 - BP | 8339 | + | 1232808 | 1241940 |  |  |  |  |  | 816 |  |  |  |  |  |  |  |  |
| 6 | Pb.c001301860.Contig1 | 1301 | - | 1232830 | 1234125 |  |  |  |  |  |  |  |  |  |  |  |  |  |  |
| 5 | Pb.c000901466.Contig2 - Ins | 6852 | - | 1235338 | 1244153 |  |  |  |  |  |  |  |  |  |  |  |  |  |  |
| 5 | Pc.c000803784.Contig1 | 2001 | + | 1236690 | 1238667 |  |  |  |  |  |  |  |  |  |  |  |  |  |  |
| 5 | PcContig113 | 1508 | + | 1239886 | 1241433 |  |  |  |  |  |  |  |  |  |  |  |  |  |  |
| 5 | Pc.c000503137.Contig1 | 2005 | + | 1242478 | 1244873 |  |  |  |  |  |  |  |  |  |  |  |  |  |  |
| 5 | MALPY00946 | 10425 | - | 1242787 | 1254089 |  |  |  |  |  | 816 |  |  |  |  |  |  |  |  |
| 5 | Pb.c001803809.Contig1 | 977 | + | 1244520 | 1245742 |  |  |  |  |  |  |  |  |  |  |  |  |  |  |
| 5 | Pc.c001504910.Contig1 | 4241 | + | 1245189 | 1249310 |  |  |  |  |  |  |  |  |  |  |  |  |  |  |
| 5 | Pb.c001803333.Contig1 | 1838 | + | 1245754 | 1247592 |  |  |  |  |  |  |  |  |  |  |  |  |  |  |
| 5 | Pb.c001904270.Contig1 | 3872 | + | 1247348 | 1251107 |  |  |  |  |  |  |  |  |  |  |  |  |  |  |
| 5 | MALPY03147 | 841 | + | 1248218 | 1249047 |  |  |  |  |  |  | 173 | 2500 |  |  |  |  |  |  |
| 5 | Pc.c000402938.Contig1 | 9035 | + | 1249820 | 1260885 |  |  |  |  |  |  |  |  |  |  |  |  |  |  |
| 5 | MALPY03427 | 513 | - | 1252229 | 1252718 |  |  |  |  |  |  | 173 |  |  |  |  |  |  |  |
| 5 | MALPY01125 | 13962 | + | 1257245 | 1271853 |  |  |  |  |  |  | 272 | 1200 |  |  |  |  |  |  |
| 5 | Pb.c001101993.Contig1 | 1933 | - | 1258588 | 1260629 |  |  |  |  |  |  |  |  |  |  |  |  |  |  |
| 5 | Pb.c000101294.Contig1 | 5600 | - | 1260928 | 1266746 |  |  |  |  |  |  |  |  |  |  |  |  |  |  |
| 5 | PcContig223 | 5550 | - | 1261750 | 1268255 | cRMPc12058 | 39721 | 2014 | 1232132 | 1271853 |  |  |  |  |  |  |  |  |  |
| 5 | MALPY01316 | 15147 | + | 1273867 | 1290166 |  |  |  |  |  |  | 272 |  |  |  |  |  |  |  |
| 5 | Pb.c000801422.Contig1 | 12625 | - | 1276702 | 1290532 |  |  |  |  |  |  |  |  |  |  |  |  |  |  |
| 5 | Pc.c000102518.Contig1 | 8197 | + | 1276840 | 1286049 |  |  |  |  |  |  |  |  |  |  |  |  |  |  |
| 5 | Pc.c000703779.Contig1 | 3623 | + | 1287273 | 1290710 | cRMPc12059 | 16843 | 3810 | 1273867 | 1290710 |  |  |  | cRMPs1214 | 58578 | 3810 | 1232132 | 1290710 |  |
| 5 | Pb.c000901877.Contig1 | 4890 | + | 1294520 | 1299658 |  |  |  |  |  |  |  |  |  |  |  |  |  |  |
| 5 | MALPY01706 | 6628 | - | 1294743 | 1301561 |  |  |  |  |  |  |  |  |  |  |  |  |  |  |
| 5 | Pc.c001708874.Contig1 | 594 | - | 1296402 | 1296996 |  |  |  |  |  |  |  |  |  |  |  |  |  |  |
| 5 | Pc.c001003271.Contig1 | 2226 | - | 1297458 | 1299801 |  |  |  |  |  |  |  |  |  |  |  |  |  |  |
| 5 | Pb.c001703275.Contig1 | 805 | + | 1299984 | 1300915 |  |  |  |  |  |  |  |  |  |  |  |  |  |  |
| 5 | Pb.c001702783.Contig1 | 1488 | + | 1301270 | 1302779 | cRMPc12060 | 8259 | 1369 | 1294520 | 1302779 |  |  |  | cRMPs1215 | 8259 | 1369 | 1294520 | 1302779 |  |
| 5 | Pb.c000601477.Contig1 | 13088 | + | 1304148 | 1316780 |  |  |  |  |  |  |  |  |  |  |  |  |  |  |
| 5 | MALPY01159 | 2486 | + | 1304163 | 1306646 |  |  |  |  |  |  |  |  |  |  |  |  |  |  |
| 5 | MALPY00437 | 15487 | - | 1306182 | 1322193 |  |  |  |  |  | 277 |  |  |  |  |  |  |  |  |
| 5 | Pc.c001002687.Contig1 | 2422 | - | 1306469 | 1308891 |  |  |  |  |  |  |  |  |  |  |  |  |  |  |
| 5 | Pc.c001405452.Contig1 | 2117 | - | 1310097 | 1312208 |  |  |  |  |  |  |  |  |  |  |  |  |  |  |
| 5 | MALPY03164 | 318 | - | 1311504 | 1311828 |  |  |  |  |  |  |  |  |  |  |  |  |  |  |
| 5 | Pc.c000302386.Contig2 | 12385 | - | 1312198 | 1325654 |  |  |  |  |  |  |  |  |  |  |  |  |  |  |
| 5 | PbContig235 | 3362 | + | 1319760 | 1323158 |  |  |  |  |  |  |  |  |  |  |  |  |  |  |
| 5 | MALPY00743 | 4923 | + | 1322339 | 1327135 |  |  |  |  |  | 277 |  |  |  |  |  |  |  |  |
| 5 | Pb.c001903631.Contig1 | 2611 | + | 1323353 | 1325988 |  |  |  |  |  |  |  |  |  |  |  |  |  |  |
| 5 | Pc.c001606145.Contig1 | 4090 | - | 1325871 | 1329551 |  |  |  |  |  |  |  |  |  |  |  |  |  |  |
| 5 | Pb.c001301536.Contig1 | 9033 | + | 1326108 | 1334915 |  |  |  |  |  |  |  |  |  |  |  |  |  |  |
| 5 | MALPY01219 | 7137 | - | 1326441 | 1333736 |  |  |  |  |  | 277 | 278 | 500 |  |  |  |  |  |  |
| 5 | Pc.c000803459.Contig1 | 3480 | - | 1331172 | 1334697 |  |  |  |  |  |  |  |  |  |  |  |  |  |  |
| 5 | MALPY00558 | 5312 | - | 1334169 | 1340175 |  |  |  |  |  |  | 278 | 500 |  |  |  |  |  |  |
| 5 | Pc.c000202165.Contig3 | 2472 | - | 1335366 | 1337793 |  |  |  |  |  |  |  |  |  |  |  |  |  |  |
| 5 | Pb.c001301864.Contig1 | 3321 | + | 1335638 | 1338905 |  |  |  |  |  |  |  |  |  |  |  |  |  |  |
| 5 | Pb.c000701187.Contig2 | 7503 | + | 1339385 | 1348888 |  |  |  |  |  |  |  |  |  |  |  |  |  |  |
| 5 | Pc.c001707839.Contig1 | 682 | - | 1339972 | 1340666 |  |  |  |  |  |  |  |  |  |  |  |  |  |  |
| 5 | MALPY01225 | 4570 | + | 1340175 | 1344879 |  |  |  |  |  | 484 | 278 |  |  |  |  |  |  |  |
| 5 | Pc.c000903337.Contig1 | 1326 | - | 1341010 | 1342336 |  |  |  |  |  |  |  |  |  |  |  |  |  |  |
| 5 | Pc.c000202662.Contig1 | 5936 | + | 1346008 | 1351893 |  |  |  |  |  |  |  |  |  |  |  |  |  |  |
| 5 | MALPY00975 | 5415 | - | 1346528 | 1351964 |  |  |  |  |  | 484 |  |  |  |  |  |  |  |  |
| 5 | Pb.c001702301.Contig1 | 3086 | - | 1350679 | 1353780 |  |  |  |  |  |  |  |  |  |  |  |  |  |  |
| 5 | MALPY02021 | 6395 | + | 1351399 | 1357896 |  |  |  |  |  | 484 |  |  |  |  |  |  |  |  |
| 5 | Pc.c001405415.Contig1 | 1477 | + | 1352213 | 1353708 |  |  |  |  |  |  |  |  |  |  |  |  |  |  |
| 5 | Pb.c001702731.Contig1 | 3819 | + | 1353799 | 1358035 |  |  |  |  |  |  |  |  |  |  |  |  |  |  |
| 5 | Pc.c001404886.Contig1 | 2742 | + | 1353921 | 1357062 | cRMPc12061 | 53887 | 380 | 1304148 | 1358035 |  |  |  |  |  |  |  |  |  |
| 5 | MALPY02084 | 4799 | - | 1358415 | 1363120 |  |  |  |  |  | 484 | 93 | 600 |  |  |  |  |  |  |
| 5 | Pb.c000601216.Contig1 | 12753 | + | 1358710 | 1372668 |  |  |  |  |  |  |  |  |  |  |  |  |  |  |
| 5 | Pc.c000202216.Contig1 | 3431 | + | 1360367 | 1363910 |  |  |  |  |  |  |  |  |  |  |  |  |  |  |
| 5 | MALPY01456 | 6726 | + | 1363495 | 1370683 |  |  |  |  |  |  | 93 | 7000 |  |  |  |  |  |  |
| 5 | Pc.c000202752.Contig1 | 2895 | - | 1364690 | 1368444 | cRMPc12062 | 14253 | 3233 | 1358415 | 1372668 |  |  |  |  |  |  |  |  |  |
| 5 | MALPY00192 | 7354 | - | 1375901 | 1383552 |  |  |  |  |  | 556A | 93 | 400 |  |  |  |  |  |  |
| 5 | Pc.c001708709.Contig1 | 631 | + | 1376254 | 1376882 |  |  |  |  |  |  |  |  |  |  |  |  |  |  |
| 5 | Pb.c001301871.Contig1 | 3633 | - | 1380488 | 1384127 |  |  |  |  |  |  |  |  |  |  |  |  |  |  |
| 5 | PcContig166 | 5352 | + | 1383049 | 1388398 |  |  |  |  |  |  |  |  |  |  |  |  |  |  |
| 5 | MALPY00945 | 2664 | + | 1383559 | 1386220 |  |  |  |  |  |  | 93 |  |  |  |  |  |  |  |
| 5 | Pb.c001301872.Contig1 | 2652 | - | 1384218 | 1386867 |  |  |  |  |  |  |  |  |  |  |  |  |  |  |
| 5 | Pb.c002113175.Contig1 | 606 | - | 1384684 | 1385290 | cRMPc12063 | 12497 | 1896 | 1375901 | 1388398 |  |  |  | cRMPs1216 | 84250 | 1896 | 1304148 | 1388398 |  |
| 5 | PbContig43 | 4319 | - | 1390294 | 1394621 |  |  |  |  |  |  |  |  |  |  |  |  |  |  |
| 5 | MALPY00603 | 6709 | + | 1391443 | 1397887 |  |  |  |  |  | 86 |  |  |  |  |  |  |  |  |
| 5 | Pc.c001504981.Contig1 | 1762 | + | 1392686 | 1394468 |  |  |  |  |  |  |  |  |  |  |  |  |  |  |
| 5 | Pb.c001102004.Contig1 | 3686 | + | 1396465 | 1400514 |  |  |  |  |  |  |  |  |  |  |  |  |  |  |
| 5 | MALPY01372 | 9014 | - | 1397637 | 1409187 |  |  |  |  |  | 86 | 78 | 1800 |  |  |  |  |  |  |
| 5 | Pc.c001103900.Contig1 | 3672 | - | 1398303 | 1402327 |  |  |  |  |  |  |  |  |  |  |  |  |  |  |
| 5 | Pb.c001502062.Contig1 | 2603 | + | 1401065 | 1403766 |  |  |  |  |  |  |  |  |  |  |  |  |  |  |
| 5 | Pc.c001103899.Contig1 | 608 | - | 1402666 | 1403274 |  |  |  |  |  |  |  |  |  |  |  |  |  |  |
| 5 | PcContig396 | 12212 | - | 1408962 | 1422373 |  |  |  |  |  |  |  |  |  |  |  |  |  |  |
| 5 | MALPY02450 | 7172 | - | 1410747 | 1418295 |  |  |  |  |  |  | 78 | 700 |  |  |  |  |  |  |
| 5 | Pb.c002006953.Contig1 | 2314 | - | 1412268 | 1414802 |  |  |  |  |  |  |  |  |  |  |  |  |  |  |
| man5 | MALPY00814 - Ins | 11660 | + | 1418450 | 1430808 |  |  |  |  |  | 179 | 78 | 1300 |  |  |  |  |  |  |
| 5 | Pb.c001904303.Contig1 | 2158 | + | 1420010 | 1422168 |  |  |  |  |  |  |  |  |  |  |  |  |  |  |
| 5 | MALPY03072 | 517 | + | 1420997 | 1421474 |  |  |  |  |  |  | 78 |  |  |  |  |  |  |  |
| 5 | Pc.c001003805.Contig1 | 833 | - | 1425335 | 1426168 |  |  |  |  |  |  |  |  |  |  |  |  |  |  |
| 5 | Pc.c000002731.Contig1 | 1921 | - | 1426550 | 1428420 |  |  |  |  |  |  |  |  |  |  |  |  |  |  |
| 5 | PcContig673 | 1602 | + | 1429437 | 1431036 |  |  |  |  |  |  |  |  |  |  |  |  |  |  |
| 5 | MALPY01123 | 2025 | - | 1430841 | 1432923 |  |  |  |  |  | 179 | 258 | 3000 |  |  |  |  |  |  |
| 5 | Pc.c000302910.Contig1 | 4880 | + | 1431715 | 1436478 |  |  |  |  |  |  |  |  |  |  |  |  |  |  |
| 5 | Pb.c000601215.Contig1 | 3118 | + | 1434050 | 1437216 |  |  |  |  |  |  |  |  |  |  |  |  |  |  |
| 5 | MALPY00234 | 6499 | + | 1436100 | 1442511 |  |  |  |  |  |  | 258 |  |  |  |  |  |  |  |
| 5 | Pc.c001505783.Contig1 | 1222 | + | 1437485 | 1438698 |  |  |  |  |  |  |  |  |  |  |  |  |  |  |
| 5 | MALPY03461 | 745 | - | 1437970 | 1438703 |  |  |  |  |  |  | 129 | 3500 |  |  |  |  |  |  |
| 5 | Pb.c000901797.Contig1 | 3385 | + | 1439031 | 1442499 |  |  |  |  |  |  |  |  |  |  |  |  |  |  |
| 5 | MALPY00361 | 7516 | + | 1442070 | 1449270 |  |  |  |  |  |  | 129 | 8000 |  |  |  |  |  |  |
| 5 | Pb.c000901733.Contig1 | 2774 | + | 1443390 | 1446269 |  |  |  |  |  |  |  |  |  |  |  |  |  |  |
| 5 | PcContig689 | 1476 | - | 1443480 | 1444977 |  |  |  |  |  |  |  |  |  |  |  |  |  |  |
| 5 | Pc.c000803858.Contig1 | 943 | - | 1445352 | 1446337 |  |  |  |  |  |  |  |  |  |  |  |  |  |  |
| 5 | Pb.c001602517.Contig1 | 2863 | + | 1446343 | 1449194 |  |  |  |  |  |  |  |  |  |  |  |  |  |  |
| 5 | Pc.c000903393.Contig1 | 6872 | + | 1446520 | 1453393 |  |  |  |  |  |  |  |  |  |  |  |  |  |  |
| 5 | PbContig346 | 2801 | + | 1449146 | 1451956 |  |  |  |  |  |  |  |  |  |  |  |  |  |  |
| 5 | Pc.c000002612.Contig1 | 2298 | + | 1450094 | 1452377 | cRMPc12064 | 63099 | 4110 | 1390294 | 1453393 |  |  |  |  |  |  |  |  |  |
| 5 | MALPY02006 | 4667 | + | 1457503 | 1462155 |  |  |  |  |  |  | 129 | 2400 |  |  |  |  |  |  |
| 5 | Pc.c001203948.Contig1 | 1400 | + | 1459248 | 1460660 | cRMPc12065 | 4652 | 2100 | 1457503 | 1462155 |  |  |  |  |  |  |  |  |  |
| 5 | Pc.c001504943.Contig1 | 2221 | - | 1464255 | 1466598 |  |  |  |  |  |  |  |  |  |  |  |  |  |  |
| 5 | MALPY03113 | 1917 | - | 1464597 | 1466530 |  |  |  |  |  |  | 129 |  |  |  |  |  |  |  |
| 5 | Pb.c001702288.Contig1 | 3853 | + | 1464620 | 1468552 | cRMPc12066 | 4297 | 793 | 1464255 | 1468552 |  |  |  | cRMPs1217 | 78258 | 793 | 1390294 | 1468552 |  |
| 5 | MALPY02186 | 11544 | - | 1469345 | 1481122 |  |  |  |  |  | 998 |  |  |  |  |  |  |  |  |
| 5 | Pc.c001606456.Contig1 | 625 | + | 1469583 | 1470208 |  |  |  |  |  |  |  |  |  |  |  |  |  |  |
| 5 | Pb.c001101882.Contig1 | 1988 | - | 1469750 | 1471729 |  |  |  |  |  |  |  |  |  |  |  |  |  |  |
| 5 | Pc.c000302970.Contig1 | 2110 | + | 1470356 | 1472498 |  |  |  |  |  |  |  |  |  |  |  |  |  |  |
| 5 | Pb.c001301810.Contig1 | 3667 | + | 1475238 | 1478836 | cRMPc12067 | 11777 | 834 | 1469345 | 1481122 |  |  |  |  |  |  |  |  |  |
| 5 | MALPY00008 | 25321 | + | 1481956 | 1507748 |  |  |  |  |  | 998 |  |  |  |  |  |  |  |  |
| 5 | Pb.c001001824.Contig1 | 3983 | - | 1482901 | 1486881 |  |  |  |  |  |  |  |  |  |  |  |  |  |  |
| 5 | Pb.c002007815.Contig1 | 2175 | - | 1489476 | 1491763 |  |  |  |  |  |  |  |  |  |  |  |  |  |  |
| 5 | Pb.c001201697.Contig2 | 4125 | - | 1496396 | 1500647 |  |  |  |  |  |  |  |  |  |  |  |  |  |  |
| 5 | Pc.c000603436.Contig1 | 811 | - | 1497740 | 1498551 |  |  |  |  |  |  |  |  |  |  |  |  |  |  |
| 5 | MALPY03434 | 757 | - | 1498826 | 1499604 |  |  |  |  |  |  | 220 | 2300 |  |  |  |  |  |  |
| 5 | PcContig392 | 3580 | - | 1498972 | 1502777 |  |  |  |  |  |  |  |  |  |  |  |  |  |  |
| 5 | Pb.c000401490.Contig1 | 4920 | + | 1502184 | 1507104 |  |  |  |  |  |  |  |  |  |  |  |  |  |  |
| 5 | MALPY03429 | 407 | + | 1502733 | 1503140 |  |  |  |  |  |  | 220 | 8000 |  |  |  |  |  |  |
| 5 | PcContig368 | 1167 | - | 1502820 | 1503977 | cRMPc12068 | 25792 | 1587 | 1481956 | 1507748 |  |  |  |  |  |  |  |  |  |
| 5 | MALPY02452 | 4733 | - | 1509335 | 1514065 |  |  |  |  |  | 523 | 220 |  |  |  |  |  |  |  |
| 5 | Pb.c000201556.Contig1 | 2272 | - | 1511391 | 1513648 |  |  |  |  |  |  |  |  |  |  |  |  |  |  |
| 5 | Pc.c000302850.Contig1 | 1629 | - | 1511541 | 1513161 | cRMPc12069 | 4730 | 1725 | 1509335 | 1514065 |  |  |  |  |  |  |  |  |  |
| 5 | MALPY00070 | 17265 | - | 1515790 | 1533203 |  |  |  |  |  | 523 |  |  |  |  |  |  |  |  |
| 5 | Pb.c000101375.Contig1 | 13778 | - | 1519562 | 1533521 |  |  |  |  |  |  |  |  |  |  |  |  |  |  |
| 5 | Pc.c000102650.Contig1 | 1642 | + | 1519829 | 1521471 |  |  |  |  |  |  |  |  |  |  |  |  |  |  |
| 5 | Pc.c000103113.Contig1 | 937 | + | 1520999 | 1521951 |  |  |  |  |  |  |  |  |  |  |  |  |  |  |
| 5 | Pc.c000603487.Contig1 | 618 | + | 1522995 | 1523601 |  |  |  |  |  |  |  |  |  |  |  |  |  |  |
| 5 | MALPY03212 | 597 | + | 1523000 | 1523585 |  |  |  |  |  |  |  |  |  |  |  |  |  |  |
| 5 | Pc.c000902695.Contig3 | 4998 | + | 1523729 | 1528599 |  |  |  |  |  |  |  |  |  |  |  |  |  |  |
| 5 | PcContig552 | 1800 | + | 1531943 | 1533743 |  |  |  |  |  |  |  |  |  |  |  |  |  |  |
| 5 | MALPY01092 | 7981 | + | 1533626 | 1542161 |  |  |  |  |  | 523 | 34 | 600 |  |  |  |  |  |  |
| 5 | Pb.c000101370.Contig1 | 2599 | + | 1534390 | 1536983 |  |  |  |  |  |  |  |  |  |  |  |  |  |  |
| 5 | Pc.c001203994.Contig1 | 3299 | - | 1535396 | 1538685 |  |  |  |  |  |  |  |  |  |  |  |  |  |  |
| 5 | MALPY03336 | 358 | + | 1536304 | 1536653 |  |  |  |  |  |  | 34 | 4300 |  |  |  |  |  |  |
| 5 | Pb.c000101369.Contig1 | 1081 | + | 1536944 | 1538028 |  |  |  |  |  |  |  |  |  |  |  |  |  |  |
| 5 | Pb.c001201998.Contig1 | 4371 | + | 1538819 | 1543219 |  |  |  |  |  |  |  |  |  |  |  |  |  |  |
| 5 | Pc.c000102574.Contig1 | 3880 | - | 1539504 | 1543277 |  |  |  |  |  |  |  |  |  |  |  |  |  |  |
| 5 | MALPY03001 | 1470 | + | 1540775 | 1542230 |  |  |  |  |  |  | 34 |  |  |  |  |  |  |  |
| 5 | MALPY00786 | 2721 | + | 1541432 | 1544153 | cRMPc12070 | 28363 | 1515 | 1515790 | 1544153 | 523 | 146 | 9000 |  |  |  |  |  |  |
| 5 | Pb.c001502598.Contig1 | 3055 | + | 1545668 | 1548732 | cRMPc12071 | 3064 | 2669 | 1545668 | 1548732 |  |  |  |  |  |  |  |  |  |
| 5 | MALPY01454 | 5795 | - | 1551401 | 1557145 |  |  |  |  |  |  | 146 | 2000 |  |  |  |  |  |  |
| 5 | Pb.c001904446.Contig1 | 1834 | - | 1552399 | 1554218 |  |  |  |  |  |  |  |  |  |  |  |  |  |  |
| 5 | Pc.c000002035.Contig1 | 5174 | - | 1553200 | 1558374 |  |  |  |  |  |  |  |  |  |  |  |  |  |  |
| 5 | Pb.c000301466.Contig1 | 8857 | - | 1558256 | 1567104 |  |  |  |  |  |  |  |  |  |  |  |  |  |  |
| 5 | MALPY00042 | 15446 | + | 1558839 | 1575178 |  |  |  |  |  | 49 | 146 |  |  |  |  |  |  |  |
| 5 | Pc.c000402725.Contig1 | 6510 | + | 1559402 | 1565903 |  |  |  |  |  |  |  |  |  |  |  |  |  |  |
| 5 | Pb.c001904855.Contig1 | 575 | - | 1568929 | 1569507 |  |  |  |  |  |  |  |  |  |  |  |  |  |  |
| 5 | Pb.c001201994.Contig1 | 3295 | - | 1574369 | 1577664 |  |  |  |  |  |  |  |  |  |  |  |  |  |  |
| 5 | MALPY02165 | 2617 | + | 1574698 | 1577306 | cRMPc12072 | 26263 | 2216 | 1551401 | 1577664 | 49 | 116 | 3000 |  |  |  |  |  |  |
| 5 | Pb.c001101506.Contig1 | 2530 | + | 1579880 | 1582440 |  |  |  |  |  |  |  |  |  |  |  |  |  |  |
| 5 | MALPY03396 | 760 | + | 1579968 | 1580728 |  |  |  |  |  |  | 116 | 1000 |  |  |  |  |  |  |
| 5 | Pc.c000202821.Contig1 | 2595 | + | 1579998 | 1582689 |  |  |  |  |  |  |  |  |  |  |  |  |  |  |
| 5 | MALPY02410 | 2120 | + | 1580843 | 1582963 | cRMPc12073 | 3083 | 1253 | 1579880 | 1582963 |  | 116 | 2000 |  |  |  |  |  |  |
| 5 | MALPY02708 | 3390 | - | 1584216 | 1587910 |  |  |  |  |  | 904 | 116 |  |  |  |  |  |  |  |
| 5 | Pc.c000803903.Contig1 | 1435 | - | 1584835 | 1586554 | cRMPc12074 | 3694 | 32 | 1584216 | 1587910 |  |  |  |  |  |  |  |  |  |
| 5 | PbContig101 | 5500 | + | 1587942 | 1593649 |  |  |  |  |  |  |  |  |  |  |  |  |  |  |
| 5 | MALPY01978 | 4666 | - | 1589058 | 1593988 |  |  |  |  |  | 904 | 137 | 2300 |  |  |  |  |  |  |
| 5 | Pc.c001708417.Contig1 | 952 | - | 1589582 | 1590519 |  |  |  |  |  |  |  |  |  |  |  |  |  |  |
| 5 | Pc.c001404921.Contig1 | 1754 | - | 1590870 | 1592610 | cRMPc12075 | 6046 | 2518 | 1587942 | 1593988 |  |  |  |  |  |  |  |  |  |
| 5 | MALPY02175 | 4996 | + | 1596506 | 1601496 |  |  |  |  |  | 541 | 137 |  |  |  |  |  |  |  |
| 5 | Pc.c000702676.Contig1 | 4370 | - | 1596639 | 1601097 |  |  |  |  |  |  |  |  |  |  |  |  |  |  |
| 5 | Pb.c001001691.Contig1 | 4414 | - | 1597353 | 1601770 | cRMPc12076 | 5264 | 531 | 1596506 | 1601770 |  |  |  |  |  |  |  |  |  |
| 5 | MALPY00326 | 4088 | + | 1602301 | 1606389 |  |  |  |  |  | 541 | 2 | 1400 |  |  |  |  |  |  |
| 5 | Pb.c001001692.Contig1 | 6535 | - | 1602535 | 1609274 |  |  |  |  |  |  |  |  |  |  |  |  |  |  |
| 5 | Pc.c000503068.Contig1 | 4472 | - | 1603040 | 1607512 |  |  |  |  |  |  |  |  |  |  |  |  |  |  |
| 5 | MALPY02987 | 1993 | - | 1607122 | 1609463 |  |  |  |  |  |  | 2 | 2000 |  |  |  |  |  |  |
| 5 | Pc.c000202816.Contig1 | 2296 | - | 1607531 | 1609827 | cRMPc12077 | 7526 | 461 | 1602301 | 1609827 |  |  |  |  |  |  |  |  |  |
| 5 | PcContig157 | 2913 | - | 1610288 | 1613522 |  |  |  |  |  |  |  |  |  |  |  |  |  |  |
| 5 | MALPY02363 | 4828 | + | 1610970 | 1616152 |  |  |  |  |  |  | 2 | 4000 |  |  |  |  |  |  |
| 5 | Pb.c001904540.Contig1 | 3491 | - | 1611102 | 1614806 |  |  |  |  |  |  |  |  |  |  |  |  |  |  |
| 5 | Pb.c002007750.Contig1 | 910 | - | 1615473 | 1616383 | cRMPc12078 | 6095 | 2789 | 1610288 | 1616383 |  |  |  |  |  |  |  |  |  |
| 5 | PcContig237 | 2289 | + | 1619172 | 1621449 |  |  |  |  |  |  |  |  |  |  |  |  |  |  |
| 5 | MALPY00581 | 6883 | + | 1620080 | 1628740 |  |  |  |  |  | 196 | 2 |  |  |  |  |  |  |  |
| 5 | PcContig295 | 1671 | - | 1622494 | 1624435 |  |  |  |  |  |  |  |  |  |  |  |  |  |  |
| 5 | Pb.c001803350.Contig1 | 1564 | + | 1623700 | 1625294 |  |  |  |  |  |  |  |  |  |  |  |  |  |  |
| 5 | MALPY01075 | 5468 | + | 1628534 | 1634513 |  |  |  |  |  | 196 |  |  |  |  |  |  |  |  |
| 5 | Pc.c000703401.Contig1 | 1706 | + | 1629498 | 1631204 |  |  |  |  |  |  |  |  |  |  |  |  |  |  |
| 5 | Pb.c000301475.Contig1 | 7468 | + | 1629754 | 1638195 |  |  |  |  |  |  |  |  |  |  |  |  |  |  |
| 5 | Pc.c001304118.Contig1 | 1440 | + | 1631848 | 1633288 |  |  |  |  |  |  |  |  |  |  |  |  |  |  |
| 5 | Pc.c000603056.Contig1 | 1061 | + | 1633369 | 1634430 |  |  |  |  |  |  |  |  |  |  |  |  |  |  |
| 5 | MALPY02328 | 2626 | - | 1635482 | 1638058 |  |  |  |  |  | 196 | 144 | 1300 |  |  |  |  |  |  |
| 5 | Pc.c000402765.Contig1 | 1193 | + | 1635513 | 1636715 |  |  |  |  |  |  |  |  |  |  |  |  |  |  |
| 5 | MALPY01150 | 11877 | - | 1638163 | 1650016 |  |  |  |  |  | 437 | 144 |  |  |  |  |  |  |  |
| 5 | Pc.c000903265.Contig1 | 3127 | - | 1643178 | 1646305 |  |  |  |  |  |  |  |  |  |  |  |  |  |  |
| 5 | Pb.c001202134.Contig1 | 6634 | + | 1645321 | 1651955 |  |  |  |  |  |  |  |  |  |  |  |  |  |  |
| TK5 | MALPY02606 | 2417 | + | 1651050 | 1653346 |  |  |  |  |  | 437 |  |  |  |  |  |  |  |  |
| 5 | Pb.c000501477.Contig1 | 3889 | + | 1653056 | 1657527 |  |  |  |  |  |  |  |  |  |  |  |  |  |  |
| 5 | MALPY03009 | 1194 | - | 1653327 | 1654554 |  |  |  |  |  | 437 |  |  |  |  |  |  |  |  |
| 5 | Pc.c000303318.Contig1 | 1001 | - | 1653939 | 1655462 |  |  |  |  |  |  |  |  |  |  |  |  |  |  |
| 5 | Pc.c001304783.Contig1 | 742 | - | 1653939 | 1654690 |  |  |  |  |  |  |  |  |  |  |  |  |  |  |
| 5 | MALPY02685 | 3751 | + | 1654972 | 1658738 |  |  |  |  |  | 437 - Ins |  |  |  |  |  |  |  |  |
| 5 | PcContig535 | 2578 | - | 1656286 | 1658897 | cRMPc12079 | 39725 | 341 | 1619172 | 1658897 |  |  |  |  |  |  |  |  |  |
| 5 | MALPY01101 | 17056 | + | 1659238 | 1678298 |  |  |  |  |  | 437 - Ins |  |  |  |  |  |  |  |  |
| 5 | PbContig190 | 4764 | - | 1659976 | 1665092 |  |  |  |  |  |  |  |  |  |  |  |  |  |  |
| 5 | PcContig569 | 9618 | + | 1660750 | 1672104 |  |  |  |  |  |  |  |  |  |  |  |  |  |  |
| 5 | Pb.c001202090.Contig1 | 1581 | + | 1666498 | 1668079 |  |  |  |  |  |  |  |  |  |  |  |  |  |  |
| 5 | Pb.c001202044.Contig1 | 1668 | + | 1667863 | 1669650 |  |  |  |  |  |  |  |  |  |  |  |  |  |  |
| 5 | Pb.c002112407.Contig1 | 863 | + | 1668112 | 1669097 |  |  |  |  |  |  |  |  |  |  |  |  |  |  |
| 5 | PbContig342 | 12500 | + | 1670145 | 1682721 |  |  |  |  |  |  |  |  |  |  |  |  |  |  |
| 5 | Pc.c000103036.Contig1 | 1062 | + | 1672373 | 1673435 |  |  |  |  |  |  |  |  |  |  |  |  |  |  |
| 5 | Pc.c000402737.Contig1 | 1271 | + | 1673831 | 1675099 |  |  |  |  |  |  |  |  |  |  |  |  |  |  |
| 5 | Pc.c001505031.Contig1 | 1236 | + | 1675853 | 1677155 |  |  |  |  |  |  |  |  |  |  |  |  |  |  |
| 5 | Pc.c001505604.Contig1 | 1139 | + | 1677371 | 1678510 |  |  |  |  |  |  |  |  |  |  |  |  |  |  |
| 5 | PcContig696 | 1280 | + | 1678186 | 1679463 |  |  |  |  |  |  |  |  |  |  |  |  |  |  |
| 5 | MALPY00447 | 3181 | + | 1678647 | 1681848 | cRMPc12080 | 23483 | 1087 | 1659238 | 1682721 | 344 |  |  |  |  |  |  |  |  |
| 5 | MALPY00113 | 8111 | + | 1683808 | 1691922 |  |  |  |  |  | 344 |  |  |  |  |  |  |  |  |
| 5 | Pb.c000601195.Contig2 | 6539 | + | 1684907 | 1691497 |  |  |  |  |  |  |  |  |  |  |  |  |  |  |
| 5 | Pc.c001203821.Contig1 | 2569 | + | 1685497 | 1688066 |  |  |  |  |  |  |  |  |  |  |  |  |  |  |
| 5 | Pc.c000904035.Contig1 | 1285 | + | 1686675 | 1687960 |  |  |  |  |  |  |  |  |  |  |  |  |  |  |
| 5 | PcContig252 | 2505 | + | 1687610 | 1690172 |  |  |  |  |  |  |  |  |  |  |  |  |  |  |
| 5 | Pb.c000701427.Contig1 | 16952 | + | 1690431 | 1709825 |  |  |  |  |  |  |  |  |  |  |  |  |  |  |
| 5 | Pc.c000503088.Contig1 | 2708 | + | 1690968 | 1693676 |  |  |  |  |  |  |  |  |  |  |  |  |  |  |
| 5 | MALPY00336 | 15685 | - | 1693576 | 1712051 |  |  |  |  |  | 148A |  |  |  |  |  |  |  |  |
| 5 | PcContig191 | 3873 | + | 1693686 | 1699714 |  |  |  |  |  |  |  |  |  |  |  |  |  |  |
| 5 | Pc.c000703453.Contig1 | 2032 | - | 1702458 | 1704487 |  |  |  |  |  |  |  |  |  |  |  |  |  |  |
| 5 | Pc.c000602372.Contig2 | 5117 | - | 1704484 | 1709581 |  |  |  |  |  |  |  |  |  |  |  |  |  |  |
| 5 | Pc.c001812688.Contig1 | 639 | - | 1710230 | 1710923 | cRMPc12081 | 28243 | 1055 | 1683808 | 1712051 |  |  |  | cRMPs1218 | 242706 | 1055 | 1469345 | 1712051 |  |
| JC5 | MALPY01589 | 2639 | xxx | 1712000 |  |  |  |  |  |  | 448 |  |  |  |  |  |  |  |  |
| 5 | MALPY02260 | 6358 | - | 1713106 | 1719565 |  |  |  |  |  | 448 |  |  |  |  |  |  |  |  |
| 5 | Pc.c001504907.Contig1 | 4793 | - | 1713510 | 1718422 |  |  |  |  |  |  |  |  |  |  |  |  |  |  |
| 5 | Pb.c001702379.Contig1 | 3439 | + | 1713672 | 1717253 |  |  |  |  |  |  |  |  |  |  |  |  |  |  |
| 5 | Pb.c001502414.Contig1 | 4769 | + | 1718000 | 1722775 |  |  |  |  |  |  |  |  |  |  |  |  |  |  |
| 5 | Pc.c000903509.Contig1 | 4307 | - | 1719720 | 1724030 |  |  |  |  |  |  |  |  |  |  |  |  |  |  |
| man5 | MALPY00190 - Ins | 16324 | + | 1719771 | 1737559 |  |  |  |  |  | 448 |  |  |  |  |  |  |  |  |
| 5 | PbContig227 | 6852 | - | 1724716 | 1731583 |  |  |  |  |  |  |  |  |  |  |  |  |  |  |
| 5 | Pb.c000601510.Contig1 | 907 | - | 1731646 | 1732598 |  |  |  |  |  |  |  |  |  |  |  |  |  |  |
| 5 | Pc.c000703951.Contig1 | 959 | + | 1731783 | 1732778 |  |  |  |  |  |  |  |  |  |  |  |  |  |  |
| 5 | Pb.c000001791.Contig1 | 3576 | - | 1733064 | 1736637 |  |  |  |  |  |  |  |  |  |  |  |  |  |  |
| man5 | Pc.c000401444.Contig1 | 4109 | - | 1734188 | 1738297 |  |  |  |  |  |  |  |  |  |  |  |  |  | MALPY00190/MALPY00518 |
| man5 | MALPY00518 | 4193 | + | 1737650 | 1741843 |  |  |  |  |  | 448 |  |  |  |  |  |  |  | Pc.c000401444.Contig1/PbContig372 |
| 5 | PbContig372 | 11351 | - | 1741197 | 1753202 |  |  |  |  |  |  |  |  |  |  |  |  |  |  |
| 5 | MALPY00777 | 7291 | - | 1741880 | 1749611 |  |  |  |  |  | 448 |  |  |  |  |  |  |  |  |
| 5 | MALPY01627 | 13962 | + | 1749406 | 1762885 |  |  |  |  |  | 448 | 190 | 2500 |  |  |  |  |  |  |
| 5 | Pc.c001304145.Contig1 | 1419 | - | 1749785 | 1751516 |  |  |  |  |  |  |  |  |  |  |  |  |  |  |
| 5 | Pb.c000501158.Contig2 | 11463 | + | 1754931 | 1766211 |  |  |  |  |  |  |  |  |  |  |  |  |  |  |
| 5 | Pc.c000402847.Contig1 | 1944 | + | 1760891 | 1762822 |  |  |  |  |  |  |  |  |  |  |  |  |  |  |
| 5 | MALPY00492 | 16006 | + | 1765301 | 1781934 |  |  |  |  |  | 536 | 190 |  |  |  |  |  |  |  |
| 5 | Pb.c001502018.Contig1 | 4768 | + | 1767524 | 1772170 |  |  |  |  |  |  |  |  |  |  |  |  |  |  |
| 5 | Pc.c001605904.Contig1 | 1244 | - | 1768188 | 1769432 |  |  |  |  |  |  |  |  |  |  |  |  |  |  |
| 5 | Pb.c001703164.Contig1 | 1468 | + | 1773787 | 1775234 |  |  |  |  |  |  |  |  |  |  |  |  |  |  |
| 5 | Pb.c001101567.Contig1 | 9483 | + | 1775873 | 1786407 |  |  |  |  |  |  |  |  |  |  |  |  |  |  |
| 5 | Pc.c001203230.Contig1 | 2184 | - | 1776980 | 1779164 |  |  |  |  |  |  |  |  |  |  |  |  |  |  |
| 5 | MALPY02377 | 4816 | - | 1782970 | 1787780 |  |  |  |  |  | 536 |  |  |  |  |  |  |  |  |
| 5 | Pb.c001402180.Contig1 | 3404 | - | 1786817 | 1790403 |  |  |  |  |  |  |  |  |  |  |  |  |  |  |
| 5 | MALPY00784 | 4177 | + | 1787779 | 1792127 |  |  |  |  |  | 536 |  |  |  |  |  |  |  |  |
| 5 | Pc.c001707698.Contig1 | 1541 | + | 1787908 | 1789521 |  |  |  |  |  |  |  |  |  |  |  |  |  |  |
| 5 | Pc.c001812662.Contig1 | 983 | + | 1789964 | 1790950 |  |  |  |  |  |  |  |  |  |  |  |  |  |  |
| 5 | Pb.c000201757.Contig1 | 2672 | + | 1790723 | 1793395 |  |  |  |  |  |  |  |  |  |  |  |  |  |  |
| 5 | MALPY02545 | 6620 | - | 1792448 | 1798815 |  |  |  |  |  | 536 |  |  |  |  |  |  |  |  |
| 5 | PbContig29 | 3722 | - | 1793503 | 1797269 |  |  |  |  |  |  |  |  |  |  |  |  |  |  |
| 5 | MALPY00340 | 10056 | - | 1797087 | 1807413 |  |  |  |  |  | 402 |  |  |  |  |  |  |  |  |
| 5 | PcContig613 | 5500 | - | 1797897 | 1803395 |  |  |  |  |  |  |  |  |  |  |  |  |  |  |
| 5 | Pb.c001402200.Contig1 | 3865 | - | 1798064 | 1801896 |  |  |  |  |  |  |  |  |  |  |  |  |  |  |
| 5 | MALPY03344 | 1054 | - | 1798672 | 1799725 |  |  |  |  |  |  |  |  |  |  |  |  |  |  |
| 5 | Pb.c001001974.Contig1 | 1050 | + | 1803356 | 1804391 |  |  |  |  |  |  |  |  |  |  |  |  |  |  |
| 5 | MALPY03245 | 821 | + | 1803988 | 1804803 |  |  |  |  |  |  | 85 | 1100 |  |  |  |  |  |  |
| 5 | MALPY03026 | 394 | - | 1805540 | 1805934 |  |  |  |  |  |  | 85 |  |  |  |  |  |  |  |
| 5 | MALPY02697 | 4510 | - | 1806869 | 1811591 | cRMPc12082 | 98485 | 293 | 1713106 | 1811591 | 402 | 234 | 700 |  |  |  |  |  |  |
| 5 | MALPY02544 | 4633 | - | 1811884 | 1816508 |  |  |  |  |  | 181 | 234 |  |  |  |  |  |  |  |
| 5 | Pb.c001002012.Contig1 | 1434 | + | 1812959 | 1814375 |  |  |  |  |  |  |  |  |  |  |  |  |  |  |
| 5 | MALPY00841 | 10888 | - | 1816439 | 1827340 |  |  |  |  |  | 181 | new/new2 | 500/3000 |  |  |  |  |  |  |
| 5 | Pc.c001405259.Contig1 | 622 | + | 1820558 | 1821180 |  |  |  |  |  |  |  |  |  |  |  |  |  |  |
| 5 | Pb.c001803975.Contig1 | 1440 | + | 1822526 | 1823969 |  |  |  |  |  |  |  |  |  |  |  |  |  |  |
| 5 | Pb.c002007520.Contig1 | 1243 | + | 1825696 | 1826960 | cRMPc12083 | 15456 | 0 | 1811884 | 1827340 |  |  |  |  |  |  |  |  |  |
| man5 | MALPY01992 | 3015 | + | 1827340 | 1828825 | cRMPc12084 | 1485 | 0 | 1827340 | 1828825 |  | new |  |  |  |  |  |  |  |
| 5 | MALPY01430 | 15070 | - | 1828825 | 1843845 |  |  |  |  |  |  | new2 |  |  |  |  |  |  |  |
| man5 | PcContig6404 | 4366 | - | 1840909 | 1845275 |  |  |  |  |  |  |  |  |  |  |  |  |  | MALPY01430 |
| man5 | PbContig3666 | 2523 | - | 1842495 | 1845018 | cRMPc12085 | 16450 |  | 1828825 | 1845275 |  |  |  | cRMPs1219 | 132169 |  | 1713106 | 1845275 | \| |
| Telomere | |  |  |  |  |  |  |  |  |  |  |  |  |  |  |  |  |  |  |
|  |  | Pb Pc Py contig |  |  |  |  | cRMP contig | Gap to next cRMP contig |  |  |  |  |  |  | cRMP scaffold | Gap to next cRMP scaffold |  |  |  |
|  | Number | 762 |  |  |  | Number | 85 | 84 |  |  |  |  |  | Number | 19 | 18 |  |  |  |
|  | Average size (bp) | 4504 |  |  |  | Average size (bp) | 19865 | 1866 |  |  |  |  |  | Average size (bp) | 94450 | 2818 |  |  |  |
|  | Minimum size (bp) | 106 |  |  |  | Minimum size (bp) | 787 | 0 |  |  |  |  |  | Minimum size (bp) | 3606 | 498 |  |  |  |
|  | Maximum size (bp) | 38432 |  |  |  | Maximum size (bp) | 98485 | 15816 |  |  |  |  |  | Maximum size (bp) | 380182 | 15816 |  |  |  |
|  | Total size (bp) | 3432229 |  |  |  | Total size (bp) | 1688500 | 156775 |  |  |  |  |  | Total size (bp) | 1794550 | 50725 |  |  |  |
|  |  |  |  |  |  |  |  |  |  |  |  |  |  |  |  |  |  |  |  |
