# Supplementary material for: A Plasmodium Whole-Genome Synteny Map: Indels and Synteny Breakpoints as Foci for Species-Specific Genes
Source: PLoS Pathog. 2005 Dec 23;1(4):e44. doi: 10.1371/journal.ppat.0010044 (PMC1317653; doi:10.1371/journal.ppat.0010044)
Supplement: Supplementary file 13 [file ppat.0010044.st029.doc]

Table S29 - RMPchr13


# Table S29 - RMPchr13

|  |  |  |  |  |  |  |  |  |  |  |  |  |  |  |  |  |  |  |  |
| --- | --- | --- | --- | --- | --- | --- | --- | --- | --- | --- | --- | --- | --- | --- | --- | --- | --- | --- | --- |
| Table S29: Tiling path of RMP contigs combined to form cRMP chromosome 13. | | | | | | |  |  |  |  |  |  |  |  |  |  |  |  |  |
|  |  |  |  |  |  |  |  |  |  |  |  |  |  |  |  |  |  |  |  |
| Tiling paths of P. berghei (Pb), P. c. chabaudi (Pc) and P. y. yoelii (Py) contigs combined into composite rodent malaria parasite (cRMP) | | | | | | | | | |  |  |  |  |  |  |  |  |  |  |
| contigs aligned against parts of the 14 chromosomes of P. falciparum (Pf). The cRMP contigs are further linked by physical linkage | | | | | | | | | |  |  |  |  |  |  |  |  |  |  |
| and PCR (see Methods) to form cRMP scaffolds. Different synteny blocks with homology to different P. falciparum chromosomes | | | | | | | | | |  |  |  |  |  |  |  |  |  |  |
| together form the cRMP chromosomes. The tables also show gaps in the alignment (black lines), cRMP telomeres (white bars) and | | | | | | | | | |  |  |  |  |  |  |  |  |  |  |
| predicted centromeres (plum/black bars). Contigs, physical groups or PCR groups interrupted by inter- or intrasyntenic regions are | | | | | | | | | |  |  |  |  |  |  |  |  |  |  |
| printed in bold. A summary of the number of RMP contigs, cRMP contigs and cRMP scaffolds as well as their average, minimum, | | | | | | | | | |  |  |  |  |  |  |  |  |  |  |
| maximum and total sizes is provided. Column headings are as follows: | | | | |  |  |  |  |  |  |  |  |  |  |  |  |  |  |  |
|  |  |  |  |  |  |  |  |  |  |  |  |  |  |  |  |  |  |  |  |
| Pf chr.: P. falciparum chromosome to which the RMP contigs are aligned | | | | | |  |  |  |  |  |  |  |  |  |  |  |  |  |  |
| (#: PROmer analysis� from this study; JC# and TK#: Carlton et al.; man#: manual BLAST analysis; | | | | | | |  |  |  |  |  |  |  |  |  |  |  |  |  |
| i: inversed orientation of the synteny block; colours refer to those used in Fig. 2 of the paper); | | | | | | |  |  |  |  |  |  |  |  |  |  |  |  |  |
| Pb Pc Py contig: names of the aligned RMP contigs; | | |  |  |  |  |  |  |  |  |  |  |  |  |  |  |  |  |  |
| Size: size (bp) of the aligned RMP contigs; | |  |  |  |  |  |  |  |  |  |  |  |  |  |  |  |  |  |  |
| +/-: orientation of the aligned RMP contigs; | |  |  |  |  |  |  |  |  |  |  |  |  |  |  |  |  |  |  |
| Start contig & End contig: estimated start & end coordinates of alignment of the RMP contigs based on the coordinates | | | | | | | | |  |  |  |  |  |  |  |  |  |  |  |
| of PROmer hits corrected for the contig�s size and orientation and presence of inter- and intrasyntenic regions | | | | | | | |  |  |  |  |  |  |  |  |  |  |  |  |
| (the start position of the first RMP contig in the alignment was set at zero); | | | | | |  |  |  |  |  |  |  |  |  |  |  |  |  |  |
| cRMP contig: names of cRMP contigs | |  |  |  |  |  |  |  |  |  |  |  |  |  |  |  |  |  |  |
| (the first 2 digits refer to the cRMP chromosome number and the following 3 digits designate the order of the contigs); | | | | | | | | |  |  |  |  |  |  |  |  |  |  |  |
| cRMP contig size: size of cRMP contigs based on the corrected coordinates of alignment; | | | | | | |  |  |  |  |  |  |  |  |  |  |  |  |  |
| Gap size to next cRMP contig: size of gaps between neighbouring cRMP contigs; | | | | | | |  |  |  |  |  |  |  |  |  |  |  |  |  |
| Start cRMP contig & End cRMP contig: estimated start & end coordinates of alignment of the cRMP contigs; | | | | | | | |  |  |  |  |  |  |  |  |  |  |  |  |
| Phys. group (Py): physical linkage of neighbouring P. y. yoelii contigs; | | | | | |  |  |  |  |  |  |  |  |  |  |  |  |  |  |
| PCR group (Py): neighbouring P. y. yoelii contigs were linked previously or for this study by PCR; | | | | | | |  |  |  |  |  |  |  |  |  |  |  |  |  |
| PCR size (Py): size of the PCR product; | |  |  |  |  |  |  |  |  |  |  |  |  |  |  |  |  |  |  |
| cRMP scaffold: names of cRMP scaffolds | |  |  |  |  |  |  |  |  |  |  |  |  |  |  |  |  |  |  |
| (the first 2 digits refer to the cRMP chromosome number and the following 2 digits designate the order of the scaffolds); | | | | | | | | |  |  |  |  |  |  |  |  |  |  |  |
| cRMP scaffold size: size of cRMP scaffolds; | | |  |  |  |  |  |  |  |  |  |  |  |  |  |  |  |  |  |
| Gap size to next cRMP scaffold: size of gaps between neighbouring cRMP scaffolds; | | | | | | |  |  |  |  |  |  |  |  |  |  |  |  |  |
| Start cRMP scaffold & End cRMP scaffold: estimated start & end coordinates of alignment of the cRMP scaffolds; | | | | | | | | |  |  |  |  |  |  |  |  |  |  |  |
| Overlap: Linkage between RMP contigs established through manual BLAST analysis. | | | | | | |  |  |  |  |  |  |  |  |  |  |  |  |  |
|  |  |  |  |  |  |  |  |  |  |  |  |  |  |  |  |  |  |  |  |
| Pf chr. | Pb Pc Py contig | Size | +/- | Start contig | End contig | cRMP contig | cRMP contig size | Gap size to next cRMP contig | Start cRMP contig | End cRMP contig | Phys. group (Py) | PCR group (Py) | PCR size (Py) | cRMP scaffold | cRMP scaffold size | Gap size to next cRMP scaffold | Start cRMP scaffold | End cRMP scaffold | Overlap (manual) |
| Telomere | |  |  |  |  |  |  |  |  |  |  |  |  |  |  |  |  |  |  |
| iman14 | MALPY01324 | 5857 | + | 0 | 5857 |  |  |  |  |  | 300 |  |  |  |  |  |  |  | \| |
| iman14 | PbContig5641 | 7560 | - | 3536 | 11096 |  |  |  |  |  |  |  |  |  |  |  |  |  | MALPY01888 |
| i14 | PbContig322 | 6532 | - | 4562 | 10926 |  |  |  |  |  |  |  |  |  |  |  |  |  |  |
| i14 | Pc.c001304090.Contig1 | 2509 | + | 5800 | 8366 |  |  |  |  |  |  |  |  |  |  |  |  |  |  |
| i14 | MALPY01888 | 3096 | - | 5867 | 9020 |  |  |  |  |  | 300 | 205 | 8500 |  |  |  |  |  |  |
| i14 | PcContig446 | 4707 | - | 7044 | 11955 |  |  |  |  |  |  |  |  |  |  |  |  |  |  |
| i14 | MALPY01782 | 11203 | + | 9189 | 20337 |  |  |  |  |  | 300 |  |  |  |  |  |  |  |  |
| i14 | Pb.c001001457.Contig1 | 6223 | + | 12500 | 18751 |  |  |  |  |  |  |  |  |  |  |  |  |  |  |
| i14 | Pc.c001708342.Contig1 | 1197 | - | 12911 | 14111 |  |  |  |  |  |  |  |  |  |  |  |  |  |  |
| i14 | PcContig435 | 4891 | - | 14112 | 19081 |  |  |  |  |  |  |  |  |  |  |  |  |  |  |
| i14 | MALPY03073 | 1197 | + | 17516 | 18713 |  |  |  |  |  |  | 205 |  |  |  |  |  |  |  |
| i14 | Pb.c000601204.Contig1 | 985 | + | 17826 | 18807 | cRMPc13001 | 20337 | 1985 | 0 | 20337 |  |  |  |  |  |  |  |  |  |
| i14 | MALPY00040 | 14840 | - | 22322 | 37336 |  |  |  |  |  | 300 |  |  |  |  |  |  |  |  |
| i14 | Pb.c000901789.Contig1 | 2564 | + | 27178 | 29742 |  |  |  |  |  |  |  |  |  |  |  |  |  |  |
| i14 | Pc.c000803188.Contig1 | 9633 | - | 28781 | 38562 |  |  |  |  |  |  |  |  |  |  |  |  |  |  |
| i14 | Pb.c000701484.Contig1 | 5950 | + | 29797 | 35899 |  |  |  |  |  |  |  |  |  |  |  |  |  |  |
| i14 | Pb.c001301874.Contig1 | 6625 | + | 36919 | 43340 |  |  |  |  |  |  |  |  |  |  |  |  |  |  |
| i14 | MALPY00193 | 14757 | - | 38136 | 54064 |  |  |  |  |  | 300 | 483 | 2000 |  |  |  |  |  |  |
| i14 | Pc.c000402957.Contig1 | 1346 | - | 39266 | 40615 |  |  |  |  |  |  |  |  |  |  |  |  |  |  |
| i14 | PcContig292 | 2251 | - | 40555 | 42797 |  |  |  |  |  |  |  |  |  |  |  |  |  |  |
| i14 | Pb.c001502014.Contig2 | 5882 | + | 47490 | 54340 |  |  |  |  |  |  |  |  |  |  |  |  |  |  |
| i14 | Pc.c000302987.Contig1 | 5510 | - | 48256 | 54755 | cRMPc13002 | 32433 | 28 | 22322 | 54755 |  |  |  |  |  |  |  |  |  |
| i14 | MALPY00076 | 9680 | - | 54783 | 64909 |  |  |  |  |  | 790 | 483 |  |  |  |  |  |  |  |
| i14 | Pb.c001803524.Contig1 | 4064 | - | 55278 | 59345 |  |  |  |  |  |  |  |  |  |  |  |  |  |  |
| i14 | PcContig243 | 3205 | - | 55609 | 58814 |  |  |  |  |  |  |  |  |  |  |  |  |  |  |
| i14 | Pb.c000501524.Contig1 | 4825 | - | 60959 | 65683 |  |  |  |  |  |  |  |  |  |  |  |  |  |  |
| i14 | PcContig512 | 1055 | - | 61491 | 62528 |  |  |  |  |  |  |  |  |  |  |  |  |  |  |
| i14 | PcContig789 | 2478 | - | 62879 | 65285 | cRMPc13003 | 10900 | 694 | 54783 | 65683 |  |  |  |  |  |  |  |  |  |
| i14 | MALPY00727 | 17833 | - | 66377 | 85171 |  |  |  |  |  | 790 |  |  |  |  |  |  |  |  |
| i14 | PbContig215 | 3873 | - | 67057 | 71158 |  |  |  |  |  |  |  |  |  |  |  |  |  |  |
| i14 | Pc.c001304322.Contig1 | 2458 | - | 80979 | 83386 |  |  |  |  |  |  |  |  |  |  |  |  |  |  |
| i14 | Pc.c001405138.Contig1 | 702 | - | 83830 | 84532 | cRMPc13004 | 18794 | 13294 | 66377 | 85171 |  |  |  | cRMPs1301 | 85171 | 13294 | 0 | 85171 |  |
| i14 | MALPY02494 | 2195 | - | 98465 | 100684 |  |  |  |  |  | 966 |  |  |  |  |  |  |  |  |
| i14 | PbContig125 - BP | 9496 | + | 98847 | 108576 |  |  |  |  |  |  |  |  |  |  |  |  |  |  |
| i14 | PcContig596 - BP | 3782 | - | 99861 | 103831 |  |  |  |  |  |  |  |  |  |  |  |  |  |  |
| i14 | MALPY00618 - BP | 8197 | - | 100883 | 109172 |  |  |  |  |  | 966 | 395\_alt | 1000 |  |  |  |  |  |  |
| i14 | MALPY03112 | 599 | - | 102606 | 103205 |  |  |  |  |  |  |  |  |  |  |  |  |  |  |
| 14 | Pc.c001304730.Contig1 | 826 | - | 103923 | 104749 |  |  |  |  |  |  |  |  |  |  |  |  |  |  |
| 14 | Pc.c001605016.Contig1 | 4607 | - | 105015 | 109610 |  |  |  |  |  |  |  |  |  |  |  |  |  |  |
| 14 | MALPY00183 | 22997 | + | 105838 | 130236 |  |  |  |  |  |  | 395\_alt | 3000 |  |  |  |  |  |  |
| 14 | Pb.c001001662.Contig1 | 18768 | + | 108905 | 129181 |  |  |  |  |  |  |  |  |  |  |  |  |  |  |
| 14 | Pc.c000703456.Contig1 | 4216 | - | 115757 | 120186 |  |  |  |  |  |  |  |  |  |  |  |  |  |  |
| 14 | Pc.c000703978.Contig1 | 637 | + | 117367 | 117992 |  |  |  |  |  |  |  |  |  |  |  |  |  |  |
| 14 | Pc.c001404756.Contig1 | 2802 | - | 120245 | 123041 |  |  |  |  |  |  |  |  |  |  |  |  |  |  |
| 14 | Pc.c000003055.Contig1 | 772 | - | 123853 | 124625 |  |  |  |  |  |  |  |  |  |  |  |  |  |  |
| 14 | Pc.c001003923.Contig1 | 917 | - | 127382 | 128336 |  |  |  |  |  |  |  |  |  |  |  |  |  |  |
| man14 | MALPY01964 | 2754 | + | 129811 | 132565 |  |  |  |  |  | 350 |  |  |  |  |  |  |  |  |
| 14 | MALPY00116 | 29133 | - | 130585 | 159318 |  |  |  |  |  | 350 | 395\_alt |  |  |  |  |  |  |  |
| 14 | Pc.c001708765.Contig1 | 570 | - | 133743 | 134313 |  |  |  |  |  |  |  |  |  |  |  |  |  |  |
| 14 | Pb.c001702386.Contig2 | 12338 | + | 134067 | 146723 |  |  |  |  |  |  |  |  |  |  |  |  |  |  |
| 14 | Pc.c000803400.Contig1 | 2209 | + | 136074 | 138283 |  |  |  |  |  |  |  |  |  |  |  |  |  |  |
| 14 | PcContig514 | 8631 | + | 140138 | 149130 |  |  |  |  |  |  |  |  |  |  |  |  |  |  |
| 14 | Pb.c001202005.Contig1 | 1511 | + | 146694 | 148525 |  |  |  |  |  |  |  |  |  |  |  |  |  |  |
| 14 | Pb.c001702880.Contig1 | 1743 | + | 148674 | 150417 |  |  |  |  |  |  |  |  |  |  |  |  |  |  |
| 14 | Pc.c001708614.Contig1 | 1029 | + | 149261 | 150290 |  |  |  |  |  |  |  |  |  |  |  |  |  |  |
| 14 | Pb.c001201612.Contig1 | 2222 | - | 155592 | 157730 |  |  |  |  |  |  |  |  |  |  |  |  |  |  |
| 14 | Pb.c000801825.Contig1 | 3182 | + | 158355 | 161543 |  |  |  |  |  |  |  |  |  |  |  |  |  |  |
| 14 | MALPY01817 | 4679 | + | 159873 | 164460 |  |  |  |  |  | 350 | 202 | 10000 |  |  |  |  |  |  |
| 14 | Pb.c001903684.Contig3 | 3238 | + | 161423 | 164656 |  |  |  |  |  |  |  |  |  |  |  |  |  |  |
| 14 | Pc.c000803455.Contig1 | 4178 | + | 162545 | 166711 | cRMPc13005 | 68246 | 2475 | 98465 | 166711 |  |  |  |  |  |  |  |  |  |
| 14 | MALPY00224 | 9595 | + | 169186 | 178407 |  |  |  |  |  |  | 202 | 1500 |  |  |  |  |  |  |
| 14 | PbContig143 | 7683 | + | 169819 | 177414 |  |  |  |  |  |  |  |  |  |  |  |  |  |  |
| 14 | PcContig745 | 1972 | - | 173482 | 175469 |  |  |  |  |  |  |  |  |  |  |  |  |  |  |
| 14 | PcContig772 | 2482 | - | 175376 | 177858 | cRMPc13006 | 9221 | 1352 | 169186 | 178407 |  |  |  |  |  |  |  |  |  |
| 14 | PbContig75 | 7170 | - | 179759 | 187380 |  |  |  |  |  |  |  |  |  |  |  |  |  |  |
| 14 | MALPY02414 | 3209 | - | 179765 | 183010 |  |  |  |  |  |  | 202 | 1800 |  |  |  |  |  |  |
| 14 | Pc.c001204013.Contig1 | 4414 | - | 183100 | 187346 |  |  |  |  |  |  |  |  |  |  |  |  |  |  |
| 14 | MALPY01300 | 29211 | - | 184402 | 215181 |  |  |  |  |  |  | 202 |  |  |  |  |  |  |  |
| 14 | Pc.c000703827.Contig1 | 1833 | + | 191308 | 193132 |  |  |  |  |  |  |  |  |  |  |  |  |  |  |
| 14 | Pb.c001702410.Contig2 | 5901 | - | 193565 | 199560 |  |  |  |  |  |  |  |  |  |  |  |  |  |  |
| 14 | PcContig639 | 5056 | + | 194820 | 199969 |  |  |  |  |  |  |  |  |  |  |  |  |  |  |
| 14 | Pb.c000801725.Contig1 | 6243 | + | 201584 | 208053 |  |  |  |  |  |  |  |  |  |  |  |  |  |  |
| 14 | Pb.c001502439.Contig1 | 3350 | + | 208175 | 211737 |  |  |  |  |  |  |  |  |  |  |  |  |  |  |
| 14 | Pc.c001103890.Contig1 | 2552 | + | 208880 | 211636 |  |  |  |  |  |  |  |  |  |  |  |  |  |  |
| 14 | Pc.c001606568.Contig1 | 1021 | + | 212063 | 213099 |  |  |  |  |  |  |  |  |  |  |  |  |  |  |
| 14 | MALPY01024 | 5281 | + | 214882 | 219874 |  |  |  |  |  |  | 471 | 1000 |  |  |  |  |  |  |
| 14 | Pb.c000501481.Contig1 | 1962 | - | 215334 | 217521 |  |  |  |  |  |  |  |  |  |  |  |  |  |  |
| 14 | Pb.c001602466.Contig1 | 1511 | - | 217668 | 219219 |  |  |  |  |  |  |  |  |  |  |  |  |  |  |
| 14 | Pb.c001602448.Contig1 | 2083 | - | 218995 | 221032 |  |  |  |  |  |  |  |  |  |  |  |  |  |  |
| 14 | MALPY00712 | 11291 | + | 219852 | 232869 |  |  |  |  |  | 193 | 471 | 7000 |  |  |  |  |  |  |
| 14 | PbContig267 | 5302 | - | 220972 | 227891 |  |  |  |  |  |  |  |  |  |  |  |  |  |  |
| 14 | Pc.c001606393.Contig1 | 1077 | + | 221942 | 222959 |  |  |  |  |  |  |  |  |  |  |  |  |  |  |
| 14 | Pc.c001104359.Contig1 | 1249 | + | 226354 | 227603 |  |  |  |  |  |  |  |  |  |  |  |  |  |  |
| 14 | Pb.c000401770.Contig1 | 1601 | - | 228307 | 229876 |  |  |  |  |  |  |  |  |  |  |  |  |  |  |
| 14 | Pb.c000101440.Contig1 | 3938 | - | 230610 | 234611 |  |  |  |  |  |  |  |  |  |  |  |  |  |  |
| 14 | MALPY01820 | 3827 | - | 232730 | 236620 | cRMPc13007 | 56861 | 1682 | 179759 | 236620 | 193 |  |  |  |  |  |  |  |  |
| 14 | PcContig126 | 8371 | + | 238302 | 246433 |  |  |  |  |  |  |  |  |  |  |  |  |  |  |
| 14 | PbContig260 | 22387 | - | 239029 | 261401 |  |  |  |  |  |  |  |  |  |  |  |  |  |  |
| 14 | MALPY01770 | 2062 | + | 239062 | 241124 |  |  |  |  |  | 52 | 471 |  |  |  |  |  |  |  |
| 14 | MALPY01686 | 8759 | + | 240631 | 249078 |  |  |  |  |  | 52 |  |  |  |  |  |  |  |  |
| man14 | MALPY01927 | 3046 | - | 247985 | 251031 |  |  |  |  |  | 52 |  |  |  |  |  |  |  | PbContig260 |
| 14 | MALPY01994 | 2917 | - | 252377 | 255281 |  |  |  |  |  | 52 |  |  |  |  |  |  |  |  |
| 14 | Pc.c001812947.Contig1 | 819 | - | 253090 | 253909 |  |  |  |  |  |  |  |  |  |  |  |  |  |  |
| 14 | MALPY02227 | 3144 | - | 253637 | 256861 |  |  |  |  |  | 684 |  |  |  |  |  |  |  |  |
| 14 | Pc.c001706590.Contig2 | 2635 | - | 254010 | 256703 |  |  |  |  |  |  |  |  |  |  |  |  |  |  |
| 14 | MALPY00341 | 8385 | - | 257314 | 265871 |  |  |  |  |  | 684 |  |  |  |  |  |  |  |  |
| 14 | Pb.c000901800.Contig1 | 4972 | - | 261838 | 266811 |  |  |  |  |  |  |  |  |  |  |  |  |  |  |
| 14 | Pc.c001504213.Contig1 | 2395 | - | 262365 | 264763 |  |  |  |  |  |  |  |  |  |  |  |  |  |  |
| 14 | MALPY00238 | 3955 | - | 266058 | 269980 |  |  |  |  |  | 684 |  |  |  |  |  |  |  |  |
| 14 | PbContig25 | 8987 | - | 267096 | 276550 |  |  |  |  |  |  |  |  |  |  |  |  |  |  |
| 14 | MALPY00847 | 6445 | - | 269986 | 276884 |  |  |  |  |  | 684 |  |  |  |  |  |  |  |  |
| 14 | Pc.c000903248.Contig1 | 4237 | - | 270343 | 275012 |  |  |  |  |  |  |  |  |  |  |  |  |  |  |
| JC14 | MALPY03122 | 658 | - | 275001 | 275626 | cRMPc13008 | 38582 | 1865 | 238302 | 276884 |  | 420 | 2100 |  |  |  |  |  |  |
| 14 | MALPY00675 | 11316 | - | 278749 | 290128 |  |  |  |  |  |  | 420 |  |  |  |  |  |  |  |
| 14 | Pb.c001602573.Contig1 | 1920 | - | 287631 | 289593 | cRMPc13009 | 11379 | 14913 | 278749 | 290128 |  |  |  | cRMPs1302 | 191663 | 14913 | 98465 | 290128 |  |
| man14 | MALPY01576 | 10413 | - | 305041 | 314900 | cRMPc13010 | 9859 | 53 | 305041 | 314900 | 775 |  |  |  |  |  |  |  |  |
| 14 | PcContig786 | 1471 | - | 314953 | 316442 |  |  |  |  |  |  |  |  |  |  |  |  |  |  |
| 14 | MALPY00028 | 16588 | + | 315339 | 333391 |  |  |  |  |  | 775 | 248 | 800 |  |  |  |  |  |  |
| 14 | Pc.c000402956.Contig1 | 2963 | - | 316731 | 319985 |  |  |  |  |  |  |  |  |  |  |  |  |  |  |
| 14 | Pb.c000301128.Contig1 | 9853 | - | 317076 | 328019 |  |  |  |  |  |  |  |  |  |  |  |  |  |  |
| 14 | Pc.c000403251.Contig1 | 1622 | - | 320320 | 322002 |  |  |  |  |  |  |  |  |  |  |  |  |  |  |
| 14 | Pc.c001203993.Contig1 | 4767 | - | 322382 | 328006 |  |  |  |  |  |  |  |  |  |  |  |  |  |  |
| 14 | Pb.c000601493.Contig1 | 2127 | - | 331254 | 333346 | cRMPc13011 | 18438 | 750 | 314953 | 333391 |  |  |  |  |  |  |  |  |  |
| 14 | MALPY03097 | 1358 | + | 334141 | 335493 |  |  |  |  |  | 435 | 248 |  |  |  |  |  |  |  |
| 14 | Pc.c000603528.Contig1 | 937 | + | 334595 | 335526 |  |  |  |  |  |  |  |  |  |  |  |  |  |  |
| 14 | Pb.c000101403.Contig1 | 9934 | - | 335133 | 345360 |  |  |  |  |  |  |  |  |  |  |  |  |  |  |
| 14 | MALPY00523 | 27939 | - | 335434 | 364779 |  |  |  |  |  | 435 |  |  |  |  |  |  |  |  |
| 14 | PcContig547 | 663 | + | 336009 | 336747 |  |  |  |  |  |  |  |  |  |  |  |  |  |  |
| 14 | Pc.c000303371.Contig1 | 836 | + | 338238 | 339074 |  |  |  |  |  |  |  |  |  |  |  |  |  |  |
| 14 | Pc.c001505159.Contig1 | 5390 | + | 339640 | 345608 |  |  |  |  |  |  |  |  |  |  |  |  |  |  |
| 14 | Pb.c000601545.Contig1 | 6090 | - | 346710 | 352848 |  |  |  |  |  |  |  |  |  |  |  |  |  |  |
| 14 | Pc.c000703900.Contig1 | 598 | + | 347893 | 348515 |  |  |  |  |  |  |  |  |  |  |  |  |  |  |
| 14 | Pc.c000903630.Contig1 | 1020 | + | 347957 | 349002 |  |  |  |  |  |  |  |  |  |  |  |  |  |  |
| 14 | Pc.c000903418.Contig1 | 2109 | + | 349568 | 351634 |  |  |  |  |  |  |  |  |  |  |  |  |  |  |
| 14 | Pc.c001003483.Contig1 | 1915 | + | 351750 | 354023 |  |  |  |  |  |  |  |  |  |  |  |  |  |  |
| 14 | Pb.c001102003.Contig1 | 2582 | - | 353295 | 356009 |  |  |  |  |  |  |  |  |  |  |  |  |  |  |
| 14 | Pc.c001003989.Contig1 | 739 | + | 354150 | 354895 |  |  |  |  |  |  |  |  |  |  |  |  |  |  |
| 14 | MALPY03497 | 884 | + | 354690 | 355580 |  |  |  |  |  |  | 376 | 2000 |  |  |  |  |  |  |
| 14 | Pc.c001003358.Contig1 | 2473 | + | 355144 | 357635 |  |  |  |  |  |  |  |  |  |  |  |  |  |  |
| 14 | Pb.c000301432.Contig1 | 2454 | - | 356244 | 358710 |  |  |  |  |  |  |  |  |  |  |  |  |  |  |
| 14 | MALPY03308 | 624 | - | 356636 | 357278 |  |  |  |  |  |  | 376 | 3000 |  |  |  |  |  |  |
| 14 | Pb.c001001399.Contig2 | 4211 | + | 358224 | 362446 |  |  |  |  |  |  |  |  |  |  |  |  |  |  |
| 14 | Pc.c000803750.Contig1 | 630 | + | 360727 | 361357 |  |  |  |  |  |  |  |  |  |  |  |  |  |  |
| 14 | MALPY03387 | 636 | - | 360917 | 361553 |  |  |  |  |  |  | 376 | 3500 |  |  |  |  |  |  |
| 14 | Pb.c000901760.Contig1 | 2646 | + | 362576 | 365231 |  |  |  |  |  |  |  |  |  |  |  |  |  |  |
| 14 | MALPY01748 | 13418 | + | 364820 | 378520 |  |  |  |  |  | 435 | 376 |  |  |  |  |  |  |  |
| 14 | Pb.c000901761.Contig1 | 3076 | - | 365152 | 368210 |  |  |  |  |  |  |  |  |  |  |  |  |  |  |
| 14 | Pb.c002006821.Contig1 | 1505 | - | 368490 | 369995 |  |  |  |  |  |  |  |  |  |  |  |  |  |  |
| 14 | Pb.c001202012.Contig1 | 1674 | - | 374577 | 376248 | cRMPc13012 | 44379 | 1198 | 334141 | 378520 |  |  |  |  |  |  |  |  |  |
| 14 | MALPY00452 | 13073 | + | 379718 | 393185 |  |  |  |  |  | 435 | 217 | 2000 |  |  |  |  |  |  |
| 14 | Pb.c001803858.Contig1 | 917 | + | 383174 | 384100 |  |  |  |  |  |  |  |  |  |  |  |  |  |  |
| 14 | Pc.c000003071.Contig1 | 3370 | - | 389618 | 392988 |  |  |  |  |  |  |  |  |  |  |  |  |  |  |
| 14 | PbContig199 | 3186 | - | 390096 | 393420 | cRMPc13013 | 13702 | 635 | 379718 | 393420 |  |  |  |  |  |  |  |  |  |
| 14 | MALPY00949 | 7436 | - | 394055 | 401714 |  |  |  |  |  |  | 217 |  |  |  |  |  |  |  |
| 14 | Pb.c000001790.Contig1 | 3216 | + | 397377 | 400698 |  |  |  |  |  |  |  |  |  |  |  |  |  |  |
| 14 | MALPY03524 | 557 | - | 398339 | 398896 | cRMPc13014 | 7659 | 2249 | 394055 | 401714 |  |  |  | cRMPs1303 | 96673 | 2249 | 305041 | 401714 |  |
| 14 | PbContig136 | 4776 | - | 403963 | 408739 |  |  |  |  |  |  |  |  |  |  |  |  |  |  |
| 14 | MALPY01091 | 12761 | - | 404293 | 417543 |  |  |  |  |  |  | 199 | 2500 |  |  |  |  |  |  |
| 14 | PcContig82 | 4184 | - | 404416 | 408591 |  |  |  |  |  |  |  |  |  |  |  |  |  |  |
| 14 | Pb.c000501409.Contig1 | 3816 | - | 410010 | 414295 |  |  |  |  |  |  |  |  |  |  |  |  |  |  |
| 14 | Pc.c001505755.Contig1 | 814 | - | 411406 | 412226 |  |  |  |  |  |  |  |  |  |  |  |  |  |  |
| 14 | Pc.c000203334.Contig1 | 749 | - | 413729 | 414478 |  |  |  |  |  |  |  |  |  |  |  |  |  |  |
| 14 | Pb.c001602418.Contig1 | 3110 | - | 414645 | 417884 |  |  |  |  |  |  |  |  |  |  |  |  |  |  |
| 14 | Pc.c000403137.Contig1 | 1302 | - | 414680 | 415934 |  |  |  |  |  |  |  |  |  |  |  |  |  |  |
| 14 | PcContig342 | 10056 | - | 417009 | 427069 |  |  |  |  |  |  |  |  |  |  |  |  |  |  |
| 14 | Pb.c002006887.Contig1 | 2010 | - | 418855 | 420841 |  |  |  |  |  |  |  |  |  |  |  |  |  |  |
| 14 | MALPY02695 | 2508 | + | 419149 | 421615 |  |  |  |  |  | 1239B | 199 | 800 |  |  |  |  |  |  |
| 14 | MALPY03225 | 837 | + | 421648 | 422491 |  |  |  |  |  |  | 199 | 1800 |  |  |  |  |  |  |
| 14 | Pb.c002007514.Contig1 | 624 | - | 422406 | 423057 |  |  |  |  |  |  |  |  |  |  |  |  |  |  |
| 14 | MALPY02411 | 4055 | + | 423018 | 426817 |  |  |  |  |  | 482B | 199 | 3000 |  |  |  |  |  |  |
| 14 | Pb.c000601549.Contig1 | 3029 | - | 423234 | 426104 | cRMPc13015 | 23106 | 4903 | 403963 | 427069 |  |  |  |  |  |  |  |  |  |
| 14 | MALPY01783 | 7269 | - | 431972 | 439699 |  |  |  |  |  | 425 | 199 | 8000 |  |  |  |  |  |  |
| 14 | PcContig404 | 8227 | + | 432595 | 441347 |  |  |  |  |  |  |  |  |  |  |  |  |  |  |
| 14 | Pb.c001102200.Contig1 | 1073 | - | 432885 | 433958 |  |  |  |  |  |  |  |  |  |  |  |  |  |  |
| 14 | Pb.c001101475.Contig1 | 4564 | - | 434585 | 438970 |  |  |  |  |  |  |  |  |  |  |  |  |  |  |
| man14 | MALPY01959 | 2780 | + | 439560 | 442340 | cRMPc13016 | 10368 | 3822 | 431972 | 442340 | 425 |  |  |  |  |  |  |  |  |
| 14 | MALPY00521 | 16299 | - | 446162 | 461880 |  |  |  |  |  |  | 199 |  |  |  |  |  |  |  |
| 14 | Pb.c001903656.Contig1 | 7225 | - | 446580 | 453307 |  |  |  |  |  |  |  |  |  |  |  |  |  |  |
| 14 | Pc.c000502412.Contig2 | 4344 | + | 448306 | 452162 |  |  |  |  |  |  |  |  |  |  |  |  |  |  |
| 14 | Pb.c001301773.Contig1 | 6407 | + | 453781 | 460170 |  |  |  |  |  |  |  |  |  |  |  |  |  |  |
| 14 | Pc.c000202757.Contig1 | 4047 | + | 454071 | 458094 |  |  |  |  |  |  |  |  |  |  |  |  |  |  |
| 14 | Pc.c000202753.Contig1 | 1721 | - | 454817 | 456517 |  |  |  |  |  |  |  |  |  |  |  |  |  |  |
| 14 | PcContig184 | 4367 | + | 454934 | 459247 | cRMPc13017 | 15718 | 808 | 446162 | 461880 |  |  |  | cRMPs1304 | 57917 | 808 | 403963 | 461880 |  |
| 14 | Pc.c001404814.Contig1 | 2365 | - | 462688 | 465053 |  |  |  |  |  |  |  |  |  |  |  |  |  |  |
| 14 | MALPY02519 | 2258 | - | 462924 | 465842 |  |  |  |  |  | 90 |  |  |  |  |  |  |  |  |
| 14 | Pb.c001301748.Contig1 | 7369 | + | 463251 | 471213 |  |  |  |  |  |  |  |  |  |  |  |  |  |  |
| 14 | MALPY03135 | 1737 | - | 465233 | 466988 |  |  |  |  |  | 90 |  |  |  |  |  |  |  |  |
| 14 | MALPY01420 | 7269 | - | 468272 | 476015 |  |  |  |  |  | 90 | 91 | 1500 |  |  |  |  |  |  |
| 14 | Pc.c000802643.Contig1 | 5497 | - | 469871 | 476518 |  |  |  |  |  |  |  |  |  |  |  |  |  |  |
| 14 | Pb.c000101363.Contig1 | 5281 | + | 474761 | 480394 |  |  |  |  |  |  |  |  |  |  |  |  |  |  |
| 14 | MALPY03332 | 1304 | + | 476633 | 477961 |  |  |  |  |  | 553 | 91 |  |  |  |  |  |  |  |
| 14 | PcContig174 | 2966 | - | 476728 | 479719 |  |  |  |  |  |  |  |  |  |  |  |  |  |  |
| 14 | MALPY02497 | 2738 | - | 477796 | 480605 |  |  |  |  |  | 553 | 223 | 950 |  |  |  |  |  |  |
| 14 | MALPY00413 | 3737 | + | 480479 | 484216 |  |  |  |  |  |  | 223 | 900 |  |  |  |  |  |  |
| 14 | Pb.c001602521.Contig1 | 5114 | - | 481321 | 486396 |  |  |  |  |  |  |  |  |  |  |  |  |  |  |
| 14 | PcContig301 | 7769 | + | 481322 | 489076 |  |  |  |  |  |  |  |  |  |  |  |  |  |  |
| 14 | MALPY02423 | 4676 | + | 484222 | 488732 | cRMPc13018 | 26388 | 1569 | 462688 | 489076 |  | 223 | 2700 |  |  |  |  |  |  |
| 14 | PcContig345 | 2015 | + | 490645 | 492663 |  |  |  |  |  |  |  |  |  |  |  |  |  |  |
| 14 | PbContig345 | 3193 | - | 490808 | 494056 |  |  |  |  |  |  |  |  |  |  |  |  |  |  |
| 14 | MALPY03502 | 671 | - | 492114 | 492797 |  |  |  |  |  |  | 425 | 2500 |  |  |  |  |  |  |
| 14 | MALPY02318 | 2919 | + | 492123 | 495010 | cRMPc13019 | 4365 | 146 | 490645 | 495010 | 360 | 223 |  |  |  |  |  |  |  |
| 14 | Pb.c001101536.Contig1 | 6088 | + | 495156 | 501422 |  |  |  |  |  |  |  |  |  |  |  |  |  |  |
| 14 | MALPY01503 | 3171 | - | 495647 | 498794 |  |  |  |  |  | 360 | 425 |  |  |  |  |  |  |  |
| 14 | Pc.c001812062.Contig1 | 1285 | + | 496399 | 497687 |  |  |  |  |  |  |  |  |  |  |  |  |  |  |
| 14 | Pc.c000503529.Contig1 | 1114 | + | 498075 | 499189 |  |  |  |  |  |  |  |  |  |  |  |  |  |  |
| 14 | MALPY00112 | 3269 | + | 498688 | 501844 |  |  |  |  |  | 360 | 450 | 1400 |  |  |  |  |  |  |
| 14 | PcContig555 | 1959 | + | 499385 | 501344 | cRMPc13020 | 6688 | 581 | 495156 | 501844 |  |  |  |  |  |  |  |  |  |
| 14 | Pc.c000403086.Contig1 | 4140 | + | 502425 | 506604 |  |  |  |  |  |  |  |  |  |  |  |  |  |  |
| 14 | MALPY01989 | 5073 | + | 502448 | 507287 |  |  |  |  |  |  | 450 |  |  |  |  |  |  |  |
| 14 | PbContig3 | 5316 | + | 502448 | 507564 |  |  |  |  |  |  |  |  |  |  |  |  |  |  |
| 14 | PcContig607 | 5726 | + | 506559 | 513076 | cRMPc13021 | 10651 | 965 | 502425 | 513076 |  |  |  | cRMPs1305 | 50388 | 965 | 462688 | 513076 |  |
| 14 | MALPY02017 | 5395 | - | 514041 | 519442 |  |  |  |  |  |  |  |  |  |  |  |  |  |  |
| 14 | Pb.c001502544.Contig1 | 2717 | + | 515393 | 518110 |  |  |  |  |  |  |  |  |  |  |  |  |  |  |
| 14 | Pc.c001002694.Contig1 | 2073 | - | 515615 | 517688 |  |  |  |  |  |  |  |  |  |  |  |  |  |  |
| 14 | Pc.c001003849.Contig1 | 627 | - | 517636 | 518263 | cRMPc13022 | 5401 | 1309 | 514041 | 519442 |  |  |  | cRMPs1306 | 5401 | 1309 | 514041 | 519442 |  |
| 14 | Pc.c000102677.Contig1 | 4977 | - | 520751 | 526103 |  |  |  |  |  |  |  |  |  |  |  |  |  |  |
| 14 | Pb.c001001990.Contig1 | 1256 | + | 521129 | 522385 |  |  |  |  |  |  |  |  |  |  |  |  |  |  |
| 14 | MALPY00293 | 6013 | - | 521255 | 527571 |  |  |  |  |  |  |  |  |  |  |  |  |  |  |
| 14 | Pb.c001001743.Contig1 | 2050 | + | 522420 | 524461 |  |  |  |  |  |  |  |  |  |  |  |  |  |  |
| 14 | Pb.c002112289.Contig1 | 701 | + | 524846 | 525541 | cRMPc13023 | 6820 | 1541 | 520751 | 527571 |  |  |  | cRMPs1307 | 6820 | 1541 | 520751 | 527571 |  |
| 14 | MALPY00897 | 6086 | - | 529112 | 535507 |  |  |  |  |  |  |  |  |  |  |  |  |  |  |
| 14 | Pb.c001702839.Contig1 | 2623 | - | 529362 | 532003 |  |  |  |  |  |  |  |  |  |  |  |  |  |  |
| 14 | PcContig731 | 1148 | + | 529770 | 530918 |  |  |  |  |  |  |  |  |  |  |  |  |  |  |
| 14 | MALPY03159 | 610 | + | 530321 | 530949 |  |  |  |  |  |  |  |  |  |  |  |  |  |  |
| 14 | Pb.c002113272.Contig1 | 567 | + | 535191 | 535758 |  |  |  |  |  |  |  |  |  |  |  |  |  |  |
| 14 | Pc.c001708078.Contig1 | 1065 | + | 535323 | 536388 | cRMPc13024 | 7276 | 180 | 529112 | 536388 |  |  |  | cRMPs1308 | 7276 | 180 | 529112 | 536388 |  |
| 14 | Pc.c000903772.Contig1 | 616 | + | 536568 | 537175 |  |  |  |  |  |  |  |  |  |  |  |  |  |  |
| 14 | Pc.c000603501.Contig1 | 1496 | - | 536734 | 538446 | cRMPc13025 | 1878 | 1897 | 536568 | 538446 |  |  |  | cRMPs1309 | 1878 | 1897 | 536568 | 538446 |  |
| 14 | PcContig411 | 3816 | - | 540343 | 544243 |  |  |  |  |  |  |  |  |  |  |  |  |  |  |
| 14 | PbContig155 | 4602 | + | 541315 | 546019 |  |  |  |  |  |  |  |  |  |  |  |  |  |  |
| man14 | MALPY01607 - Ins | 7814 | - | 542279 | 552471 |  |  |  |  |  | 952 | 177 | 4000 |  |  |  |  |  |  |
| man14 | MALPY01970 | 4093 | + | 552093 | 556118 | cRMPc13026 | 15775 | 1968 | 540343 | 556118 | 952 |  |  |  |  |  |  |  |  |
| 14 | MALPY02173 | 5437 | - | 558086 | 563485 |  |  |  |  |  | 1049 | 177 |  |  |  |  |  |  |  |
| 14 | Pc.c001003256.Contig1 | 8819 | + | 560485 | 570215 |  |  |  |  |  |  |  |  |  |  |  |  |  |  |
| 14 | Pb.c001502485.Contig1 | 2303 | - | 561363 | 563633 |  |  |  |  |  |  |  |  |  |  |  |  |  |  |
| man14 | MALPY02117 | 2382 | + | 563835 | 565602 |  |  |  |  |  | 1049 |  |  |  |  |  |  |  |  |
| 14 | Pb.c000501193.Contig2 | 6556 | - | 565591 | 571996 |  |  |  |  |  |  |  |  |  |  |  |  |  |  |
| 14 | MALPY02658 | 4118 | - | 566675 | 570725 |  |  |  |  |  | 1049 |  |  |  |  |  |  |  |  |
| 14 | MALPY01106 | 6958 | - | 568702 | 575523 |  |  |  |  |  |  |  |  |  |  |  |  |  |  |
| 14 | Pc.c001505074.Contig1 | 1727 | + | 570020 | 571996 | cRMPc13027 | 17437 | 3758 | 558086 | 575523 |  |  |  | cRMPs1310 | 35180 | 3758 | 540343 | 575523 |  |
| 14 | MALPY02826 | 3367 | - | 579281 | 582651 |  |  |  |  |  | 318 |  |  |  |  |  |  |  |  |
| 14 | PbContig196 | 3573 | + | 579465 | 583497 |  |  |  |  |  |  |  |  |  |  |  |  |  |  |
| 14 | Pc.c000102471.Contig1 | 6762 | + | 580988 | 588522 |  |  |  |  |  |  |  |  |  |  |  |  |  |  |
| 14 | MALPY03034 | 1398 | - | 583212 | 584610 |  |  |  |  |  | 318 |  |  |  |  |  |  |  |  |
| 14 | MALPY03035 | 1675 | - | 584777 | 586494 | cRMPc13028 | 9241 | 3805 | 579281 | 588522 | 318 | 108\_alt |  | cRMPs1311 | 9241 | 3805 | 579281 | 588522 |  |
| 14 | MALPY02676 | 3037 | + | 592327 | 595190 | cRMPc13029 | 2863 | 1223 | 592327 | 595190 |  | 361 | 2500 |  |  |  |  |  |  |
| 14 | Pb.c001602487.Contig1 | 6903 | - | 596413 | 603666 |  |  |  |  |  |  |  |  |  |  |  |  |  |  |
| 14 | MALPY00284 | 2785 | + | 597844 | 600584 |  |  |  |  |  |  | 361 |  |  |  |  |  |  |  |
| 14 | Pc.c001606454.Contig1 | 1256 | + | 598459 | 599809 |  |  |  |  |  |  |  |  |  |  |  |  |  |  |
| 14 | Pc.c001605832.Contig1 | 2350 | + | 600125 | 602475 |  |  |  |  |  |  |  |  |  |  |  |  |  |  |
| 14 | MALPY02138 | 5491 | + | 601176 | 607547 |  |  |  |  |  |  |  |  |  |  |  |  |  |  |
| 14 | MALPY03049 | 204 | - | 601913 | 602117 |  |  |  |  |  |  | 140 | 4200 |  |  |  |  |  |  |
| 14 | Pc.c001605862.Contig1 | 2496 | + | 602533 | 605029 |  |  |  |  |  |  |  |  |  |  |  |  |  |  |
| 14 | Pc.c001304130.Contig1 | 1858 | + | 605998 | 607961 |  |  |  |  |  |  |  |  |  |  |  |  |  |  |
| 14 | Pb.c000701440.Contig1 | 6011 | + | 607678 | 614480 |  |  |  |  |  |  |  |  |  |  |  |  |  |  |
| 14 | MALPY01237 | 4438 | + | 608089 | 612572 |  |  |  |  |  |  | 140 | 1500 |  |  |  |  |  |  |
| 14 | Pc.c001708187.Contig1 | 1157 | - | 610513 | 611670 |  |  |  |  |  |  |  |  |  |  |  |  |  |  |
| 14 | Pc.c001812763.Contig1 | 644 | - | 612612 | 613256 |  |  |  |  |  |  |  |  |  |  |  |  |  |  |
| 14 | MALPY00547 | 22426 | + | 613028 | 637002 |  |  |  |  |  | 220 | 140 | 1300 |  |  |  |  |  |  |
| 14 | Pc.c000602845.Contig1 | 4997 | - | 613867 | 618987 |  |  |  |  |  |  |  |  |  |  |  |  |  |  |
| 14 | Pc.c000202153.Contig2 | 8207 | - | 619871 | 629352 |  |  |  |  |  |  |  |  |  |  |  |  |  |  |
| 14 | Pb.c000801413.Contig2 | 13542 | + | 621538 | 635788 |  |  |  |  |  |  |  |  |  |  |  |  |  |  |
| 14 | Pc.c001304651.Contig1 | 644 | - | 633584 | 634228 |  |  |  |  |  |  |  |  |  |  |  |  |  |  |
| 14 | Pc.c001103778.Contig1 | 2042 | - | 634411 | 636453 | cRMPc13030 | 40589 | 379 | 596413 | 637002 |  |  |  |  |  |  |  |  |  |
| 14 | MALPY01695 | 14054 | - | 637381 | 651545 |  |  |  |  |  | 220 | 140 |  |  |  |  |  |  |  |
| 14 | Pc.c001003262.Contig1 | 2284 | - | 639751 | 642035 |  |  |  |  |  |  |  |  |  |  |  |  |  |  |
| 14 | Pb.c000001338.Contig1 | 6537 | + | 639988 | 646552 |  |  |  |  |  |  |  |  |  |  |  |  |  |  |
| 14 | Pc.c001003260.Contig1 | 673 | - | 642090 | 642763 |  |  |  |  |  |  |  |  |  |  |  |  |  |  |
| 14 | Pc.c001304150.Contig1 | 2017 | - | 642822 | 644837 | cRMPc13031 | 14164 | 2623 | 637381 | 651545 |  |  |  | cRMPs1312 | 59218 | 2623 | 592327 | 651545 |  |
| 14 | MALPY00438 | 26831 | - | 654168 | 682123 |  |  |  |  |  |  |  |  |  |  |  |  |  |  |
| 14 | Pb.c000401491.Contig1 | 5832 | + | 655000 | 661354 |  |  |  |  |  |  |  |  |  |  |  |  |  |  |
| 14 | Pc.c000402796.Contig1 | 1559 | + | 655734 | 657293 |  |  |  |  |  |  |  |  |  |  |  |  |  |  |
| 14 | PcContig554 | 5924 | + | 657715 | 664476 |  |  |  |  |  |  |  |  |  |  |  |  |  |  |
| 14 | PbContig47 | 16896 | - | 661763 | 679134 |  |  |  |  |  |  |  |  |  |  |  |  |  |  |
| 14 | Pc.c000402808.Contig1 | 3607 | + | 665206 | 668799 |  |  |  |  |  |  |  |  |  |  |  |  |  |  |
| 14 | Pc.c001104245.Contig1 | 1032 | + | 668693 | 669753 |  |  |  |  |  |  |  |  |  |  |  |  |  |  |
| 14 | Pc.c000402818.Contig1 | 2723 | + | 672701 | 675439 |  |  |  |  |  |  |  |  |  |  |  |  |  |  |
| 14 | Pc.c000002624.Contig1 | 4043 | + | 675508 | 680028 |  |  |  |  |  |  |  |  |  |  |  |  |  |  |
| 14 | MALPY03569 | 862 | - | 678147 | 679093 |  |  |  |  |  |  | 254 | 1600 |  |  |  |  |  |  |
| 14 | Pb.c001001767.Contig1 | 1525 | - | 679227 | 680779 |  |  |  |  |  |  |  |  |  |  |  |  |  |  |
| 14 | MALPY03243 | 1201 | - | 679956 | 681085 | cRMPc13032 | 27955 | 391 | 654168 | 682123 |  | 254 |  | cRMPs1313 | 27955 | 391 | 654168 | 682123 |  |
| 14 | MALPY02376 | 14819 | - | 682514 | 696929 |  |  |  |  |  |  |  |  |  |  |  |  |  |  |
| 14 | Pb.c000501442.Contig1 | 7496 | - | 685661 | 693087 |  |  |  |  |  |  |  |  |  |  |  |  |  |  |
| 14 | Pc.c000903253.Contig1 | 4772 | - | 686032 | 690822 |  |  |  |  |  |  |  |  |  |  |  |  |  |  |
| 14 | MALPY03044 | 499 | + | 689679 | 690178 |  |  |  |  |  |  |  |  |  |  |  |  |  |  |
| 14 | Pb.c000601805.Contig1 | 1253 | - | 694655 | 695908 |  |  |  |  |  |  |  |  |  |  |  |  |  |  |
| 14 | MALPY03247 | 770 | - | 694958 | 695728 | cRMPc13033 | 14415 | 1244 | 682514 | 696929 |  | 246 | 2100 |  |  |  |  |  |  |
| 14 | Pb.c000001808.Contig1 | 4961 | + | 698173 | 703155 |  |  |  |  |  |  |  |  |  |  |  |  |  |  |
| 14 | MALPY01636 | 6181 | - | 698365 | 704546 |  |  |  |  |  |  | 246 | 1700 |  |  |  |  |  |  |
| 14 | MALPY00019 | 9574 | + | 704507 | 714395 |  |  |  |  |  |  | 246 | 1400 |  |  |  |  |  |  |
| 14 | Pb.c001803936.Contig1 | 2382 | + | 711690 | 714150 |  |  |  |  |  |  |  |  |  |  |  |  |  |  |
| 14 | Pb.c001803488.Contig1 | 2339 | + | 714280 | 716606 |  |  |  |  |  |  |  |  |  |  |  |  |  |  |
| 14 | Pc.c000402889.Contig1 | 640 | + | 714528 | 715165 |  |  |  |  |  |  |  |  |  |  |  |  |  |  |
| 14 | MALPY01545 | 17213 | + | 714734 | 732292 |  |  |  |  |  | 1012 | 246 |  |  |  |  |  |  |  |
| 14 | Pc.c001304230.Contig1 | 2984 | - | 716378 | 719392 |  |  |  |  |  |  |  |  |  |  |  |  |  |  |
| 14 | PbContig366 | 2504 | - | 721789 | 724305 |  |  |  |  |  |  |  |  |  |  |  |  |  |  |
| 14 | Pb.c000901462.Contig3 | 7001 | - | 724707 | 731526 |  |  |  |  |  |  |  |  |  |  |  |  |  |  |
| 14 | Pc.c000602875.Contig1 | 6109 | - | 725194 | 731108 | cRMPc13034 | 34119 | 160 | 698173 | 732292 |  |  |  |  |  |  |  |  |  |
| 14 | MALPY00073 | 8418 | + | 732452 | 741022 |  |  |  |  |  | 1012 |  |  |  |  |  |  |  |  |
| 14 | PbContig70 | 12911 | + | 736161 | 749253 |  |  |  |  |  |  |  |  |  |  |  |  |  |  |
| 14 | Pc.c001303490.Contig1 | 3201 | - | 737370 | 740718 |  |  |  |  |  |  |  |  |  |  |  |  |  |  |
| 14 | MALPY01944 | 13873 | - | 741177 | 755148 |  |  |  |  |  | 1012 |  |  |  |  |  |  |  |  |
| 14 | Pc.c000303411.Contig1 | 1774 | - | 744235 | 746017 |  |  |  |  |  |  |  |  |  |  |  |  |  |  |
| 14 | MALPY03069 | 524 | + | 745417 | 745956 |  |  |  |  |  |  | 188 | 1800 |  |  |  |  |  |  |
| 14 | MALPY03445 | 855 | - | 746396 | 747257 |  |  |  |  |  |  | 188 | 10000 |  |  |  |  |  |  |
| 14 | Pc.c001606712.Contig1 | 1081 | - | 747664 | 748742 |  |  |  |  |  |  |  |  |  |  |  |  |  |  |
| 14 | Pb.c000701583.Contig1 | 7234 | - | 749384 | 756862 |  |  |  |  |  |  |  |  |  |  |  |  |  |  |
| 14 | Pc.c000002571.Contig1 | 1169 | + | 752446 | 753612 | cRMPc13035 | 24410 | 222 | 732452 | 756862 |  |  |  |  |  |  |  |  |  |
| 14 | PbContig330 | 18151 | + | 757084 | 777811 |  |  |  |  |  |  |  |  |  |  |  |  |  |  |
| 14 | MALPY00213 | 17662 | + | 757199 | 777398 |  |  |  |  |  |  | 188 | 2200 |  |  |  |  |  |  |
| 14 | Pc.c000402864.Contig1 | 1308 | + | 758941 | 760243 |  |  |  |  |  |  |  |  |  |  |  |  |  |  |
| 14 | Pc.c000302989.Contig1 | 4527 | + | 760393 | 765864 |  |  |  |  |  |  |  |  |  |  |  |  |  |  |
| 14 | Pc.c001605858.Contig1 | 1285 | + | 766109 | 767397 |  |  |  |  |  |  |  |  |  |  |  |  |  |  |
| 14 | Pc.c000302857.Contig1 | 2410 | - | 769616 | 771972 |  |  |  |  |  |  |  |  |  |  |  |  |  |  |
| 14 | Pc.c001605837.Contig1 | 3374 | + | 774028 | 777390 | cRMPc13036 | 20727 | 662 | 757084 | 777811 |  |  |  |  |  |  |  |  |  |
| 14 | Pb.c001301525.Contig1 | 4332 | - | 778473 | 782805 |  |  |  |  |  |  |  |  |  |  |  |  |  |  |
| 14 | MALPY02417 | 3361 | - | 778608 | 781969 | cRMPc13037 | 4332 | 374 | 778473 | 782805 |  | 188 |  | cRMPs1314 | 100291 | 374 | 682514 | 782805 |  |
| 14 | MALPY00232 | 23119 | + | 783179 | 807499 |  |  |  |  |  | 343 |  |  |  |  |  |  |  |  |
| 14 | Pc.c001304119.Contig1 | 1708 | - | 783339 | 785056 |  |  |  |  |  |  |  |  |  |  |  |  |  |  |
| 14 | Pb.c001502386.Contig1 | 16816 | - | 783908 | 801141 |  |  |  |  |  |  |  |  |  |  |  |  |  |  |
| 14 | PcContig233 | 3905 | - | 785185 | 789752 |  |  |  |  |  |  |  |  |  |  |  |  |  |  |
| 14 | PcContig230 | 7908 | - | 789764 | 797687 |  |  |  |  |  |  |  |  |  |  |  |  |  |  |
| 14 | Pc.c001606022.Contig1 | 4336 | + | 797686 | 802081 |  |  |  |  |  |  |  |  |  |  |  |  |  |  |
| 14 | Pc.c000503114.Contig1 | 2672 | - | 804446 | 807109 |  |  |  |  |  |  |  |  |  |  |  |  |  |  |
| 14 | Pb.c000801358.Contig1 | 3598 | - | 804604 | 808262 |  |  |  |  |  |  |  |  |  |  |  |  |  |  |
| 14 | Pb.c001903624.Contig1 | 1423 | - | 804604 | 806087 |  |  |  |  |  |  |  |  |  |  |  |  |  |  |
| 14 | Pb.c001903718.Contig1 | 611 | - | 805673 | 806272 |  |  |  |  |  |  |  |  |  |  |  |  |  |  |
| 14 | MALPY02111 | 4971 | + | 807974 | 812924 |  |  |  |  |  | 343 | 355 | 500 |  |  |  |  |  |  |
| 14 | Pc.c000202867.Contig1 | 3457 | - | 808183 | 811724 |  |  |  |  |  |  |  |  |  |  |  |  |  |  |
| 14 | PbContig23 | 3449 | - | 809082 | 812528 |  |  |  |  |  |  |  |  |  |  |  |  |  |  |
| 14 | PbContig233 | 7714 | - | 812463 | 821158 |  |  |  |  |  |  |  |  |  |  |  |  |  |  |
| 14 | MALPY03566 | 710 | + | 813030 | 813743 |  |  |  |  |  |  | 355 | 2300 |  |  |  |  |  |  |
| 14 | MALPY01869 | 18280 | - | 816157 | 834490 |  |  |  |  |  | 378 | 355 |  |  |  |  |  |  |  |
| 14 | Pc.c001103675.Contig1 | 6978 | - | 816622 | 823653 |  |  |  |  |  |  |  |  |  |  |  |  |  |  |
| 14 | MALPY03561 | 803 | - | 817041 | 817835 |  |  |  |  |  |  |  |  |  |  |  |  |  |  |
| 14 | Pb.c000401165.Contig3 | 20955 | + | 821217 | 842178 |  |  |  |  |  |  |  |  |  |  |  |  |  |  |
| 14 | Pc.c000803487.Contig1 | 6848 | + | 823901 | 830932 |  |  |  |  |  |  |  |  |  |  |  |  |  |  |
| 14 | Pc.c001103777.Contig1 | 2278 | + | 833232 | 835501 |  |  |  |  |  |  |  |  |  |  |  |  |  |  |
| 14 | MALPY01599 | 2616 | - | 834109 | 836721 |  |  |  |  |  | 378 |  |  |  |  |  |  |  |  |
| 14 | MALPY00424 | 7411 | - | 835411 | 842915 |  |  |  |  |  | 378 |  |  |  |  |  |  |  |  |
| 14 | Pc.c001505412.Contig1 | 1493 | + | 835791 | 837278 |  |  |  |  |  |  |  |  |  |  |  |  |  |  |
| 14 | Pc.c000904006.Contig1 | 1276 | + | 837769 | 839060 |  |  |  |  |  |  |  |  |  |  |  |  |  |  |
| 14 | Pc.c001304349.Contig1 | 1760 | + | 839131 | 840846 |  |  |  |  |  |  |  |  |  |  |  |  |  |  |
| 14 | MALPY03363 | 786 | - | 839348 | 840095 | cRMPc13038 | 59736 | 2805 | 783179 | 842915 |  |  |  |  |  |  |  |  |  |
| 14 | MALPY00184 | 10924 | + | 845720 | 856860 |  |  |  |  |  | 378 |  |  |  |  |  |  |  |  |
| 14 | Pc.c000402884.Contig1 | 4513 | + | 847638 | 852151 |  |  |  |  |  |  |  |  |  |  |  |  |  |  |
| 14 | Pb.c000101428.Contig1 | 5658 | + | 849120 | 854749 |  |  |  |  |  |  |  |  |  |  |  |  |  |  |
| 14 | Pc.c001304271.Contig1 | 2314 | + | 854293 | 856787 |  |  |  |  |  |  |  |  |  |  |  |  |  |  |
| 14 | Pb.c001301923.Contig1 | 5575 | - | 855204 | 860747 |  |  |  |  |  |  |  |  |  |  |  |  |  |  |
| 14 | MALPY00772 | 6999 | + | 855389 | 862893 |  |  |  |  |  | 378 | 144 | 2500 |  |  |  |  |  |  |
| 14 | Pb.c000101405.Contig1 | 1681 | - | 861625 | 863303 |  |  |  |  |  |  |  |  |  |  |  |  |  |  |
| 14 | MALPY00653 | 14119 | + | 863102 | 877199 |  |  |  |  |  |  | 144 | 4000 |  |  |  |  |  |  |
| 14 | Pb.c001702756.Contig1 | 4901 | - | 863204 | 868426 |  |  |  |  |  |  |  |  |  |  |  |  |  |  |
| 14 | PcContig756 | 4918 | - | 863903 | 869079 |  |  |  |  |  |  |  |  |  |  |  |  |  |  |
| 14 | Pb.c001101578.Contig3 | 6602 | - | 868457 | 874830 |  |  |  |  |  |  |  |  |  |  |  |  |  |  |
| 14 | MALPY03210 | 596 | - | 869288 | 869911 |  |  |  |  |  |  | 144 |  |  |  |  |  |  |  |
| 14 | Pc.c001708533.Contig1 | 555 | + | 873421 | 873976 |  |  |  |  |  |  |  |  |  |  |  |  |  |  |
| 14 | PcContig739 | 1921 | + | 874402 | 876290 |  |  |  |  |  |  |  |  |  |  |  |  |  |  |
| 14 | Pb.c001402155.Contig1 | 2323 | - | 875385 | 877690 | cRMPc13039 | 31970 | 1527 | 845720 | 877690 |  |  |  | cRMPs1315 | 94511 | 1527 | 783179 | 877690 |  |
| 14 | MALPY02402 | 3700 | + | 879217 | 882920 |  |  |  |  |  |  | 13 | 2000 |  |  |  |  |  |  |
| 14 | Pb.c000501502.Contig1 | 3838 | - | 879271 | 883112 |  |  |  |  |  |  |  |  |  |  |  |  |  |  |
| 14 | Pc.c000402870.Contig1 | 1593 | + | 879543 | 881136 |  |  |  |  |  |  |  |  |  |  |  |  |  |  |
| 14 | Pc.c000703853.Contig1 | 890 | + | 881322 | 882218 | cRMPc13040 | 3895 | 689 | 879217 | 883112 |  |  |  |  |  |  |  |  |  |
| 14 | PbContig88 | 9437 | - | 883801 | 892824 |  |  |  |  |  |  |  |  |  |  |  |  |  |  |
| 14 | MALPY02213 | 5312 | + | 884671 | 889564 |  |  |  |  |  |  | 13 | 2000 |  |  |  |  |  |  |
| 14 | MALPY01252 | 13819 | + | 891218 | 905578 |  |  |  |  |  |  | 13 |  |  |  |  |  |  |  |
| 14 | Pb.c001402078.Contig1 | 3667 | - | 892476 | 896575 |  |  |  |  |  |  |  |  |  |  |  |  |  |  |
| 14 | Pb.c001402077.Contig1 | 1703 | - | 896611 | 898461 | cRMPc13041 | 21777 | 923 | 883801 | 905578 |  |  |  | cRMPs1316 | 26361 | 923 | 879217 | 905578 |  |
| 14 | MALPY00185 | 21070 | + | 906501 | 928146 |  |  |  |  |  |  | 432 | 2400 |  |  |  |  |  |  |
| 14 | PcContig303 | 5234 | - | 908562 | 913763 |  |  |  |  |  |  |  |  |  |  |  |  |  |  |
| 14 | Pb.c001301763.Contig1 | 3056 | - | 909739 | 912762 |  |  |  |  |  |  |  |  |  |  |  |  |  |  |
| 14 | Pb.c001402168.Contig1 | 2419 | - | 913411 | 915899 |  |  |  |  |  |  |  |  |  |  |  |  |  |  |
| 14 | Pb.c001702309.Contig1 | 5004 | - | 918551 | 923549 |  |  |  |  |  |  |  |  |  |  |  |  |  |  |
| 14 | Pc.c000303017.Contig1 | 1201 | + | 920058 | 921259 | cRMPc13042 | 21645 | 2987 | 906501 | 928146 |  |  |  |  |  |  |  |  |  |
| 14 | MALPY03126 | 1975 | - | 931133 | 933252 |  |  |  |  |  |  | 432 | 4200 |  |  |  |  |  |  |
| 14 | Pb.c001301826.Contig1 | 4343 | + | 931479 | 935822 | cRMPc13043 | 4689 | 814 | 931133 | 935822 |  |  |  |  |  |  |  |  |  |
| 14 | Pc.c000602866.Contig1 | 3389 | - | 936636 | 940214 |  |  |  |  |  |  |  |  |  |  |  |  |  |  |
| 14 | MALPY00034 | 6199 | - | 937363 | 943425 |  |  |  |  |  |  | 432 | 850 |  |  |  |  |  |  |
| 14 | Pb.c000801347.Contig2 | 5724 | - | 937692 | 943426 | cRMPc13044 | 6790 | 60 | 936636 | 943426 |  |  |  |  |  |  |  |  |  |
| 14 | Pb.c001602770.Contig1 | 1230 | - | 943486 | 944665 |  |  |  |  |  |  |  |  |  |  |  |  |  |  |
| 14 | MALPY03386 | 842 | + | 943513 | 944376 |  |  |  |  |  |  | 432 | 800 |  |  |  |  |  |  |
| 14 | MALPY03385 | 724 | - | 944245 | 944998 |  |  |  |  |  |  | 432 | 700 |  |  |  |  |  |  |
| 14 | MALPY02002 | 3843 | - | 944978 | 949141 |  |  |  |  |  |  | 432 | 1500 |  |  |  |  |  |  |
| 14 | Pb.c001301750.Contig1 | 8103 | + | 947026 | 955961 |  |  |  |  |  |  |  |  |  |  |  |  |  |  |
| 14 | Pc.c001811943.Contig1 | 5984 | - | 947307 | 954292 |  |  |  |  |  |  |  |  |  |  |  |  |  |  |
| 14 | MALPY01987 | 4886 | + | 950713 | 955422 |  |  |  |  |  | 769 | 432 |  |  |  |  |  |  |  |
| 14 | MALPY01737 | 7639 | + | 954330 | 962076 |  |  |  |  |  | 769 |  |  |  |  |  |  |  |  |
| 14 | Pc.c001405471.Contig1 | 1170 | - | 954411 | 955540 |  |  |  |  |  |  |  |  |  |  |  |  |  |  |
| 14 | Pb.c002112995.Contig1 | 963 | + | 956282 | 957260 |  |  |  |  |  |  |  |  |  |  |  |  |  |  |
| 14 | Pb.c001602464.Contig1 | 3411 | + | 957941 | 961547 | cRMPc13045 | 18590 | 1243 | 943486 | 962076 |  |  |  |  |  |  |  |  |  |
| 14 | MALPY03088 | 1954 | + | 963319 | 965273 |  |  |  |  |  | 769 |  |  |  |  |  |  |  |  |
| 14 | MALPY01439 | 4885 | - | 965182 | 969875 |  |  |  |  |  | 769 | 90 | 1400 |  |  |  |  |  |  |
| 14 | Pb.c001702800.Contig1 | 8616 | + | 966269 | 974225 |  |  |  |  |  |  |  |  |  |  |  |  |  |  |
| 14 | PcContig278 | 3027 | - | 969195 | 972204 |  |  |  |  |  |  |  |  |  |  |  |  |  |  |
| 14 | Pc.c001003842.Contig1 | 1011 | - | 969370 | 970387 |  |  |  |  |  |  |  |  |  |  |  |  |  |  |
| 14 | MALPY02222 | 2711 | - | 970043 | 972736 |  |  |  |  |  | 742 | 90 |  |  |  |  |  |  |  |
| 14 | Pc.c001003293.Contig1 | 1487 | - | 972904 | 974391 |  |  |  |  |  |  |  |  |  |  |  |  |  |  |
| man14 | MALPY02223 - Ins | 9853 | + | 972939 | 982400 |  |  |  |  |  | 742 | 92 | 4000 |  |  |  |  |  |  |
| 14 | Pb.c002007792.Contig1 | 939 | + | 977339 | 978248 |  |  |  |  |  |  |  |  |  |  |  |  |  |  |
| 14 | Pb.c001302106.Contig1 | 1433 | + | 978837 | 980276 | cRMPc13046 | 19081 | 1350 | 963319 | 982400 |  |  |  |  |  |  |  |  |  |
| man14 | MALPY02513 | 2201 | - | 983750 | 985972 |  |  |  |  |  | 730 |  |  |  |  |  |  |  |  |
| 14 | Pc.c000903851.Contig1 | 1979 | + | 985579 | 987597 |  |  |  |  |  |  |  |  |  |  |  |  |  |  |
| 14 | MALPY01767 | 6963 | - | 986018 | 993221 |  |  |  |  |  | 730 | 92 |  |  |  |  |  |  |  |
| 14 | Pb.c001702759.Contig1 | 5514 | + | 989136 | 995539 |  |  |  |  |  |  |  |  |  |  |  |  |  |  |
| 14 | Pc.c000903322.Contig1 | 1670 | + | 990564 | 992668 |  |  |  |  |  |  |  |  |  |  |  |  |  |  |
| 14 | Pc.c000903732.Contig1 | 2019 | + | 992766 | 995457 |  |  |  |  |  |  |  |  |  |  |  |  |  |  |
| 14 | MALPY00524 | 7488 | - | 993942 | 1002402 |  |  |  |  |  | 730 |  |  |  |  |  |  |  |  |
| 14 | Pb.c000201259.Contig2 | 8439 | + | 997776 | 1004072 |  |  |  |  |  |  |  |  |  |  |  |  |  |  |
| 14 | Pc.c000803335.Contig1 | 1879 | + | 998270 | 1000152 |  |  |  |  |  |  |  |  |  |  |  |  |  |  |
| 14 | MALPY00473 | 19753 | - | 999291 | 1022299 |  |  |  |  |  | 730 |  |  |  |  |  |  |  |  |
| 14 | Pc.c000703975.Contig1 | 1322 | + | 1000292 | 1001623 |  |  |  |  |  |  |  |  |  |  |  |  |  |  |
| 14 | Pc.c000903855.Contig1 | 905 | + | 1002222 | 1003160 |  |  |  |  |  |  |  |  |  |  |  |  |  |  |
| 14 | Pc.c001605931.Contig1 | 2549 | + | 1003385 | 1005895 |  |  |  |  |  |  |  |  |  |  |  |  |  |  |
| 14 | Pb.c001802864.Contig1 | 2721 | + | 1006348 | 1009069 |  |  |  |  |  |  |  |  |  |  |  |  |  |  |
| 14 | PbContig334 | 14149 | - | 1012836 | 1027335 |  |  |  |  |  |  |  |  |  |  |  |  |  |  |
| 14 | PcContig40 | 1563 | - | 1017876 | 1019433 |  |  |  |  |  |  |  |  |  |  |  |  |  |  |
| 14 | Pc.c000303069.Contig1 | 2616 | - | 1022206 | 1024801 |  |  |  |  |  |  |  |  |  |  |  |  |  |  |
| 14 | MALPY01629 | 10917 | - | 1022390 | 1034113 |  |  |  |  |  | 730 |  |  |  |  |  |  |  |  |
| 14 | Pb.c001904330.Contig1 | 2263 | - | 1030758 | 1033186 | cRMPc13047 | 50363 | 376 | 983750 | 1034113 |  |  |  |  |  |  |  |  |  |
| 14 | Pb.c001001444.Contig1 | 5114 | - | 1034489 | 1040073 |  |  |  |  |  |  |  |  |  |  |  |  |  |  |
| 14 | MALPY01241 | 13343 | - | 1034586 | 1048750 |  |  |  |  |  | 730 | 460 | 1400 |  |  |  |  |  |  |
| 14 | Pc.c001505801.Contig1 | 690 | - | 1037075 | 1037768 |  |  |  |  |  |  |  |  |  |  |  |  |  |  |
| 14 | Pc.c001103882.Contig1 | 2596 | - | 1037965 | 1040561 |  |  |  |  |  |  |  |  |  |  |  |  |  |  |
| 14 | Pb.c001101895.Contig1 | 2575 | - | 1042404 | 1044994 | cRMPc13048 | 14261 | 5954 | 1034489 | 1048750 |  |  |  |  |  |  |  |  |  |
| 14 | MALPY00144 | 8022 | - | 1054704 | 1062795 |  |  |  |  |  | 72 | 460 |  |  |  |  |  |  |  |
| 14 | Pb.c001702716.Contig1 | 5755 | - | 1059433 | 1065142 |  |  |  |  |  |  |  |  |  |  |  |  |  |  |
| 14 | Pc.c000903437.Contig1 | 593 | + | 1061126 | 1061758 |  |  |  |  |  |  |  |  |  |  |  |  |  |  |
| 14 | Pc.c000503357.Contig1 | 644 | + | 1061381 | 1062025 |  |  |  |  |  |  |  |  |  |  |  |  |  |  |
| 14 | Pc.c001304123.Contig1 | 623 | + | 1061401 | 1062033 |  |  |  |  |  |  |  |  |  |  |  |  |  |  |
| 14 | MALPY03265 | 397 | - | 1061486 | 1061877 |  |  |  |  |  |  | 278 | 350 |  |  |  |  |  |  |
| 14 | PcContig439 | 2025 | + | 1061696 | 1063666 |  |  |  |  |  |  |  |  |  |  |  |  |  |  |
| 14 | MALPY03144 | 1442 | - | 1061744 | 1063210 |  |  |  |  |  |  | 278 |  |  |  |  |  |  |  |
| 14 | MALPY02627 | 3757 | - | 1062159 | 1065792 | cRMPc13049 | 11088 | 1869 | 1054704 | 1065792 | 72 | 108\_alt | 3000 |  |  |  |  |  |  |
| 14 | Pb.c001001817.Contig1 | 3782 | + | 1067661 | 1071437 |  |  |  |  |  |  |  |  |  |  |  |  |  |  |
| 14 | Pc.c001405273.Contig1 | 1065 | - | 1068780 | 1069845 |  |  |  |  |  |  |  |  |  |  |  |  |  |  |
| 14 | MALPY01930 | 5513 | + | 1068853 | 1074439 |  |  |  |  |  |  | 108\_alt |  |  |  |  |  |  |  |
| 14 | Pc.c001405198.Contig1 | 687 | - | 1070268 | 1070955 |  |  |  |  |  |  |  |  |  |  |  |  |  |  |
| 14 | Pc.c000302977.Contig1 | 1982 | - | 1072266 | 1074407 |  |  |  |  |  |  |  |  |  |  |  |  |  |  |
| 14 | Pb.c001502597.Contig1 | 1752 | + | 1073030 | 1074890 |  |  |  |  |  |  |  |  |  |  |  |  |  |  |
| 14 | MALPY03522 | 331 | - | 1073309 | 1073604 | cRMPc13050 | 7229 | 1914 | 1067661 | 1074890 |  | 192 - Ins | 3200 |  |  |  |  |  |  |
| 14 | MALPY00935 | 24874 | + | 1076804 | 1102923 |  |  |  |  |  |  | 192 - Ins |  |  |  |  |  |  |  |
| 14 | Pb.c000101054.Contig1 | 10588 | + | 1078518 | 1089273 |  |  |  |  |  |  |  |  |  |  |  |  |  |  |
| 14 | Pb.c001702973.Contig1 | 723 | + | 1081794 | 1082517 |  |  |  |  |  |  |  |  |  |  |  |  |  |  |
| 14 | Pc.c000202738.Contig1 | 1470 | - | 1083210 | 1084680 |  |  |  |  |  |  |  |  |  |  |  |  |  |  |
| 14 | Pc.c000002561.Contig1 | 1370 | - | 1084894 | 1086264 |  |  |  |  |  |  |  |  |  |  |  |  |  |  |
| 14 | MALPY03244 | 280 | + | 1085633 | 1085912 |  |  |  |  |  |  |  |  |  |  |  |  |  |  |
| 14 | Pb.c001802803.Contig1 | 3278 | + | 1090045 | 1093337 |  |  |  |  |  |  |  |  |  |  |  |  |  |  |
| 14 | Pc.c001605992.Contig1 | 1499 | + | 1090184 | 1091666 |  |  |  |  |  |  |  |  |  |  |  |  |  |  |
| 14 | Pc.c000903970.Contig1 | 994 | + | 1091748 | 1092742 |  |  |  |  |  |  |  |  |  |  |  |  |  |  |
| 14 | Pc.c000703305.Contig1 | 1805 | + | 1096865 | 1098667 |  |  |  |  |  |  |  |  |  |  |  |  |  |  |
| 14 | MALPY01281 | 6024 | + | 1100913 | 1106767 |  |  |  |  |  | 984 |  |  |  |  |  |  |  |  |
| man14 | PbContig5429 | 5909 | - | 1103988 | 1109897 |  |  |  |  |  |  |  |  |  |  |  |  |  | MALPY01281 |
| man14 | Pb.c000101053.Contig3 - Ins | 10928 | + | 1104004 | 1115371 |  |  |  |  |  |  |  |  |  |  |  |  |  |  |
| man14 | MALPY01371 - Ins | 16955 | + | 1104541 | 1122702 |  |  |  |  |  | 115 | 80 | 5000 |  |  |  |  |  |  |
| man14 | PcContig3616 | 1135 | - | 1106078 | 1107213 |  |  |  |  |  |  |  |  |  |  |  |  |  | MALPY03052 |
| 14 | MALPY03052 | 1611 | - | 1106615 | 1108205 |  |  |  |  |  | 984 | 146 |  |  |  |  |  |  |  |
| man14 | PbContig2865 | 1394 | + | 1122458 | 1123852 |  |  |  |  |  |  |  |  |  |  |  |  |  | MALPY01371 |
| man14 | MALPY02556 | 2231 | + | 1123029 | 1125208 |  |  |  |  |  | 115 |  |  |  |  |  |  |  | \| |
| man14 | PbContig2662 | 3197 | - | 1123491 | 1126688 | cRMPc13051 | 49884 | 2092 | 1076804 | 1126688 |  |  |  |  |  |  |  |  | \| |
| 14 | Pb.c000902066.Contig1 | 952 | + | 1128780 | 1129726 |  |  |  |  |  |  |  |  |  |  |  |  |  |  |
| 14 | Pc.c001811737.Contig1 | 1429 | + | 1129049 | 1130478 |  |  |  |  |  |  |  |  |  |  |  |  |  |  |
| 14 | MALPY02077 | 2435 | + | 1129099 | 1131804 |  |  |  |  |  |  | 80 |  |  |  |  |  |  |  |
| 14 | Pb.c000601556.Contig1 | 4867 | + | 1130216 | 1135059 | cRMPc13052 | 6279 | 2661 | 1128780 | 1135059 |  |  |  | cRMPs1317 | 228558 | 2661 | 906501 | 1135059 |  |
| 14 | Pc.c000502991.Contig1 | 3551 | - | 1137720 | 1141472 |  |  |  |  |  |  |  |  |  |  |  |  |  |  |
| 14 | MALPY01339 | 14942 | - | 1138324 | 1153367 |  |  |  |  |  |  |  |  |  |  |  |  |  |  |
| 14 | Pb.c001101587.Contig1 | 4616 | + | 1140473 | 1145329 |  |  |  |  |  |  |  |  |  |  |  |  |  |  |
| 14 | MALPY03337 | 402 | - | 1141693 | 1142098 |  |  |  |  |  |  | 451 | 9000 |  |  |  |  |  |  |
| 14 | Pc.c001606483.Contig1 | 604 | + | 1145444 | 1146048 |  |  |  |  |  |  |  |  |  |  |  |  |  |  |
| 14 | Pc.c001505607.Contig1 | 913 | + | 1146080 | 1146996 |  |  |  |  |  |  |  |  |  |  |  |  |  |  |
| 14 | Pb.c001201962.Contig1 | 2091 | - | 1149421 | 1151512 |  |  |  |  |  |  |  |  |  |  |  |  |  |  |
| 14 | MALPY03358 | 112 | + | 1150580 | 1150692 |  |  |  |  |  |  | 451 | 6000 |  |  |  |  |  |  |
| 14 | Pb.c000601567.Contig1 | 1945 | - | 1151553 | 1153507 | cRMPc13053 | 15787 | 1149 | 1137720 | 1153507 |  |  |  |  |  |  |  |  |  |
| 14 | Pc.c000402859.Contig1 | 2740 | + | 1154656 | 1157414 |  |  |  |  |  |  |  |  |  |  |  |  |  |  |
| 14 | Pb.c001502592.Contig1 | 3328 | + | 1154684 | 1157965 |  |  |  |  |  |  |  |  |  |  |  |  |  |  |
| 14 | MALPY00362 | 11028 | - | 1155708 | 1166825 |  |  |  |  |  |  | 451 |  |  |  |  |  |  |  |
| 14 | Pb.c000002013.Contig1 | 1759 | + | 1158515 | 1160280 |  |  |  |  |  |  |  |  |  |  |  |  |  |  |
| 14 | Pc.c001708008.Contig1 | 1551 | + | 1159749 | 1161279 |  |  |  |  |  |  |  |  |  |  |  |  |  |  |
| 14 | Pb.c000001272.Contig2 | 8088 | + | 1160393 | 1168292 |  |  |  |  |  |  |  |  |  |  |  |  |  |  |
| 14 | MALPY03570 | 782 | - | 1160686 | 1161477 |  |  |  |  |  |  | 487 | 9000 |  |  |  |  |  |  |
| 14 | Pc.c001707733.Contig1 | 1885 | + | 1161228 | 1163231 |  |  |  |  |  |  |  |  |  |  |  |  |  |  |
| 14 | Pc.c001304225.Contig1 | 1715 | - | 1163959 | 1165652 |  |  |  |  |  |  |  |  |  |  |  |  |  |  |
| 14 | Pc.c001304226.Contig1 | 2718 | - | 1165301 | 1168077 | cRMPc13054 | 13636 | 481 | 1154656 | 1168292 |  |  |  |  |  |  |  |  |  |
| 14 | Pb.c001702747.Contig1 | 4222 | - | 1168773 | 1172992 |  |  |  |  |  |  |  |  |  |  |  |  |  |  |
| 14 | MALPY00551 | 20708 | - | 1168852 | 1188841 |  |  |  |  |  | 824 | 487 |  |  |  |  |  |  |  |
| 14 | Pc.c000803854.Contig1 | 2598 | + | 1171434 | 1174028 |  |  |  |  |  |  |  |  |  |  |  |  |  |  |
| 14 | Pb.c000601238.Contig1 | 11997 | - | 1173010 | 1184249 |  |  |  |  |  |  |  |  |  |  |  |  |  |  |
| 14 | Pc.c001002711.Contig1 | 4100 | - | 1175377 | 1179489 |  |  |  |  |  |  |  |  |  |  |  |  |  |  |
| 14 | Pc.c001003971.Contig1 | 811 | - | 1179656 | 1180470 |  |  |  |  |  |  |  |  |  |  |  |  |  |  |
| 14 | Pc.c000403301.Contig1 | 738 | - | 1183824 | 1184562 |  |  |  |  |  |  |  |  |  |  |  |  |  |  |
| 14 | Pb.c002007579.Contig1 | 956 | - | 1184799 | 1185755 |  |  |  |  |  |  |  |  |  |  |  |  |  |  |
| 14 | Pc.c000503496.Contig1 | 1117 | + | 1186132 | 1187249 |  |  |  |  |  |  |  |  |  |  |  |  |  |  |
| 14 | Pb.c002112858.Contig1 | 867 | - | 1186458 | 1187304 |  |  |  |  |  |  |  |  |  |  |  |  |  |  |
| 14 | Pb.c001301732.Contig1 | 10715 | - | 1187796 | 1198412 |  |  |  |  |  |  |  |  |  |  |  |  |  |  |
| 14 | Pc.c000602901.Contig1 | 3071 | + | 1187970 | 1191032 |  |  |  |  |  |  |  |  |  |  |  |  |  |  |
| 14 | MALPY01460 | 14630 | + | 1188807 | 1203393 |  |  |  |  |  | 824 |  |  |  |  |  |  |  |  |
| 14 | MALPY03093 | 630 | + | 1189969 | 1190650 |  |  |  |  |  |  | 244 | 7000 |  |  |  |  |  |  |
| 14 | Pc.c001708532.Contig1 | 1027 | + | 1192795 | 1193822 |  |  |  |  |  |  |  |  |  |  |  |  |  |  |
| 14 | Pc.c001812292.Contig1 | 986 | + | 1196080 | 1197078 |  |  |  |  |  |  |  |  |  |  |  |  |  |  |
| 14 | MALPY03124 | 113 | + | 1197008 | 1197121 |  |  |  |  |  |  | 244 | 3500 |  |  |  |  |  |  |
| 14 | Pc.c001707753.Contig1 | 1028 | + | 1197093 | 1198118 |  |  |  |  |  |  |  |  |  |  |  |  |  |  |
| 14 | Pb.c001301848.Contig1 | 1652 | - | 1198545 | 1200347 |  |  |  |  |  |  |  |  |  |  |  |  |  |  |
| 14 | PcContig621 | 2925 | + | 1199801 | 1202619 |  |  |  |  |  |  |  |  |  |  |  |  |  |  |
| 14 | PbContig164 | 5159 | - | 1200772 | 1206229 |  |  |  |  |  |  |  |  |  |  |  |  |  |  |
| 14 | MALPY03181 | 411 | + | 1200874 | 1201285 |  |  |  |  |  |  | 244 | 900 |  |  |  |  |  |  |
| 14 | MALPY03121 | 1829 | + | 1203804 | 1205698 |  |  |  |  |  | 749 | 244 |  |  |  |  |  |  |  |
| 14 | PcContig24 | 1325 | + | 1204262 | 1205640 |  |  |  |  |  |  |  |  |  |  |  |  |  |  |
| 14 | MALPY01311 | 2774 | + | 1205762 | 1208536 | cRMPc13055 | 39763 | 823 | 1168773 | 1208536 | 749 | 176 | 3000 |  |  |  |  |  |  |
| 14 | Pb.c000901450.Contig1 | 6838 | - | 1209359 | 1216275 |  |  |  |  |  |  |  |  |  |  |  |  |  |  |
| 14 | Pc.c001405100.Contig1 | 3427 | + | 1209389 | 1212840 |  |  |  |  |  |  |  |  |  |  |  |  |  |  |
| 14 | MALPY02263 | 6268 | - | 1209519 | 1215840 |  |  |  |  |  | 156 | 176 |  |  |  |  |  |  |  |
| 14 | MALPY03103 | 146 | + | 1212029 | 1212175 |  |  |  |  |  |  |  |  |  |  |  |  |  |  |
| 14 | MALPY03328 | 941 | - | 1212465 | 1213434 |  |  |  |  |  |  |  |  |  |  |  |  |  |  |
| 14 | Pc.c001003244.Contig1 | 4196 | - | 1212885 | 1217081 |  |  |  |  |  |  |  |  |  |  |  |  |  |  |
| man14 | MALPY02123 | 2886 | + | 1215622 | 1218508 | cRMPc13056 | 9149 | 10358 | 1209359 | 1218508 | 156 |  |  | cRMPs1318 | 80788 | 10358 | 1137720 | 1218508 | Pb.c000901450.Contig1 |
| 14 | MALPY00071 | 8913 | - | 1228866 | 1237407 |  |  |  |  |  |  | 122 | 5500 |  |  |  |  |  |  |
| 14 | Pb.c000901854.Contig1 | 9395 | + | 1229056 | 1238438 |  |  |  |  |  |  |  |  |  |  |  |  |  |  |
| 14 | PcContig560 | 7693 | + | 1229271 | 1236993 | cRMPc13057 | 9572 | 4928 | 1228866 | 1238438 |  |  |  |  |  |  |  |  |  |
| 14 | MALPY02096 | 4237 | + | 1243366 | 1247603 |  |  |  |  |  |  | 122 | 2500 |  |  |  |  |  |  |
| 14 | Pc.c001103884.Contig1 | 2319 | + | 1245525 | 1247844 | cRMPc13058 | 4478 | 1757 | 1243366 | 1247844 |  |  |  |  |  |  |  |  |  |
| 14 | Pb.c001502465.Contig1 | 2343 | - | 1249601 | 1251875 |  |  |  |  |  |  |  |  |  |  |  |  |  |  |
| 14 | MALPY03377 | 1359 | - | 1249633 | 1250974 | cRMPc13059 | 2274 | 4915 | 1249601 | 1251875 |  | 122 | 6000 |  |  |  |  |  |  |
| man14 | PbContig209 - Ins | 8801 | - | 1256790 | 1269429 |  |  |  |  |  |  |  |  |  |  |  |  |  |  |
| man14 | MALPY01792 - Ins | 12976 | - | 1258979 | 1274002 |  |  |  |  |  | 1104 | 122 |  |  |  |  |  |  |  |
| 14 | Pc.c001304635.Contig1 | 635 | + | 1266400 | 1267032 |  |  |  |  |  |  |  |  |  |  |  |  |  |  |
| 14 | Pc.c001404842.Contig1 | 1298 | + | 1267655 | 1268944 |  |  |  |  |  |  |  |  |  |  |  |  |  |  |
| 14 | Pb.c000801722.Contig1 | 4808 | + | 1268492 | 1273950 |  |  |  |  |  |  |  |  |  |  |  |  |  |  |
| 14 | MALPY03448 | 327 | + | 1268854 | 1269181 |  |  |  |  |  |  | 334 | 5000 |  |  |  |  |  |  |
| 14 | Pc.c001304913.Contig1 | 832 | - | 1271831 | 1272657 | cRMPc13060 | 17212 | 4193 | 1256790 | 1274002 |  |  |  |  |  |  |  |  |  |
| 14 | MALPY01082 | 15670 | + | 1278195 | 1294906 |  |  |  |  |  | 1104 | 334 |  |  |  |  |  |  |  |
| 14 | PbContig362 | 7416 | + | 1280219 | 1288662 |  |  |  |  |  |  |  |  |  |  |  |  |  |  |
| 14 | Pc.c000903345.Contig1 | 5257 | + | 1285999 | 1291171 |  |  |  |  |  |  |  |  |  |  |  |  |  |  |
| 14 | Pb.c002006833.Contig1 | 2313 | - | 1289601 | 1292315 |  |  |  |  |  |  |  |  |  |  |  |  |  |  |
| 14 | MALPY03399 | 818 | - | 1291840 | 1292646 |  |  |  |  |  |  |  |  |  |  |  |  |  |  |
| 14 | Pc.c001606291.Contig1 | 1320 | + | 1291964 | 1293272 |  |  |  |  |  |  |  |  |  |  |  |  |  |  |
| 14 | Pc.c000803639.Contig1 | 2589 | + | 1294125 | 1296771 | cRMPc13061 | 18576 | 2636 | 1278195 | 1296771 |  |  |  | cRMPs1319 | 67905 | 2636 | 1228866 | 1296771 |  |
| 14 | PcContig600 | 3706 | - | 1299407 | 1303113 |  |  |  |  |  |  |  |  |  |  |  |  |  |  |
| man14 | MALPY01766 | 2773 | - | 1299639 | 1302400 |  |  |  |  |  | 516 |  |  |  |  |  |  |  |  |
| 14 | PbContig113 | 7551 | - | 1300117 | 1307776 |  |  |  |  |  |  |  |  |  |  |  |  |  |  |
| 14 | MALPY00373 | 9482 | - | 1302071 | 1311448 |  |  |  |  |  | 516 |  |  |  |  |  |  |  |  |
| 14 | PcContig557 | 2828 | + | 1303214 | 1306009 |  |  |  |  |  |  |  |  |  |  |  |  |  |  |
| 14 | PcContig592 | 2407 | + | 1305043 | 1307414 |  |  |  |  |  |  |  |  |  |  |  |  |  |  |
| 14 | MALPY03237 | 813 | - | 1307584 | 1308397 |  |  |  |  |  |  |  |  |  |  |  |  |  |  |
| 14 | Pb.c000601491.Contig1 | 4007 | - | 1307593 | 1311717 | cRMPc13062 | 12310 | 13273 | 1299407 | 1311717 |  |  |  | cRMPs1320 | 12310 | 13273 | 1299407 | 1311717 |  |
| 14 | MALPY00805 | 6618 | + | 1324990 | 1332063 |  |  |  |  |  |  |  |  |  |  |  |  |  |  |
| 14 | Pb.c002005826.Contig1 | 3158 | + | 1325022 | 1328073 |  |  |  |  |  |  |  |  |  |  |  |  |  |  |
| 14 | PcContig694 | 2747 | + | 1325924 | 1328620 |  |  |  |  |  |  |  |  |  |  |  |  |  |  |
| 14 | Pb.c001502460.Contig1 | 2391 | + | 1329241 | 1331747 |  |  |  |  |  |  |  |  |  |  |  |  |  |  |
| 14 | MALPY03560 | 750 | + | 1330729 | 1331505 |  |  |  |  |  |  |  |  |  |  |  |  |  |  |
| 14 | MALPY03241 | 776 | + | 1330792 | 1331667 |  |  |  |  |  |  | 253 - NS | 6500 |  |  |  |  |  |  |
| 14 | Pc.c001812593.Contig1 | 598 | - | 1331224 | 1331837 | cRMPc13063 | 7073 | 3321 | 1324990 | 1332063 |  |  |  |  |  |  |  |  |  |
| man14 | MALPY02443 | 2842 | - | 1335384 | 1338226 |  |  |  |  |  | 774 |  |  |  |  |  |  |  |  |
| 14 | Pb.c001101593.Contig1 | 4078 | - | 1337372 | 1341451 |  |  |  |  |  |  |  |  |  |  |  |  |  |  |
| 14 | Pc.c001404782.Contig1 | 2041 | - | 1337861 | 1339809 |  |  |  |  |  |  |  |  |  |  |  |  |  |  |
| 14 | MALPY01322 | 10575 | + | 1338167 | 1348882 |  |  |  |  |  | 774 | 253 - NS | 1500 |  |  |  |  |  |  |
| 14 | Pb.c001602058.Contig2 | 3601 | - | 1341095 | 1344717 |  |  |  |  |  |  |  |  |  |  |  |  |  |  |
| 14 | Pc.c000202898.Contig1 | 6154 | + | 1344227 | 1349814 |  |  |  |  |  |  |  |  |  |  |  |  |  |  |
| 14 | Pb.c000301168.Contig1 | 5669 | - | 1345257 | 1350141 |  |  |  |  |  |  |  |  |  |  |  |  |  |  |
| 14 | MALPY03248 | 1896 | + | 1348090 | 1349986 |  |  |  |  |  | 1105 | 253 |  |  |  |  |  |  |  |
| 14 | MALPY01074 | 7723 | + | 1349983 | 1357463 |  |  |  |  |  | 1105 | 236 - NS | 9000 |  |  |  |  |  |  |
| 14 | Pb.c001904969.Contig1 | 1092 | - | 1350248 | 1351346 |  |  |  |  |  |  |  |  |  |  |  |  |  |  |
| 14 | Pb.c000601177.Contig1 | 2999 | - | 1351508 | 1354507 | cRMPc13064 | 22079 | 5210 | 1335384 | 1357463 |  |  |  |  |  |  |  |  |  |
| man14 | MALPY02607 | 2511 | - | 1362673 | 1365625 | cRMPc13065 | 2952 | 838 | 1362673 | 1365625 | 1105 |  |  |  |  |  |  |  |  |
| 14 | MALPY00458 | 18862 | + | 1366463 | 1386101 |  |  |  |  |  |  | 236 - NS | 2000 |  |  |  |  |  |  |
| 14 | Pb.c000801384.Contig2 | 5786 | - | 1367350 | 1373514 |  |  |  |  |  |  |  |  |  |  |  |  |  |  |
| 14 | PcContig254 | 3826 | + | 1368084 | 1372291 |  |  |  |  |  |  |  |  |  |  |  |  |  |  |
| 14 | Pc.c000703471.Contig1 | 5231 | - | 1372783 | 1377988 |  |  |  |  |  |  |  |  |  |  |  |  |  |  |
| 14 | Pb.c001401698.Contig1 | 11382 | - | 1374692 | 1386047 | cRMPc13066 | 19638 | 1345 | 1366463 | 1386101 |  |  |  |  |  |  |  |  |  |
| 14 | Pb.c001803523.Contig1 | 1991 | - | 1387446 | 1389446 |  |  |  |  |  |  |  |  |  |  |  |  |  |  |
| 14 | MALPY02286 | 7820 | - | 1388503 | 1396372 |  |  |  |  |  |  | 236 | 2800 |  |  |  |  |  |  |
| 14 | Pb.c000601498.Contig1 | 5213 | - | 1389955 | 1395126 |  |  |  |  |  |  |  |  |  |  |  |  |  |  |
| 14 | Pc.c000302936.Contig1 | 3182 | - | 1390538 | 1393717 |  |  |  |  |  |  |  |  |  |  |  |  |  |  |
| 14 | Pb.c001702734.Contig1 | 3654 | - | 1395201 | 1398873 |  |  |  |  |  |  |  |  |  |  |  |  |  |  |
| 14 | MALPY02733 | 2354 | - | 1398798 | 1401149 |  |  |  |  |  | 158 | 236 |  |  |  |  |  |  |  |
| 14 | Pb.c000301133.Contig1 | 7269 | - | 1399026 | 1406723 |  |  |  |  |  |  |  |  |  |  |  |  |  |  |
| 14 | PcContig373 | 5817 | + | 1399213 | 1404948 |  |  |  |  |  |  |  |  |  |  |  |  |  |  |
| 14 | MALPY01060 | 11475 | - | 1400395 | 1412517 |  |  |  |  |  | 158 | 247 | 1400 |  |  |  |  |  |  |
| 14 | Pc.c000803337.Contig1 | 979 | + | 1406129 | 1407106 |  |  |  |  |  |  |  |  |  |  |  |  |  |  |
| 14 | Pb.c002006823.Contig1 | 2357 | + | 1406926 | 1409287 |  |  |  |  |  |  |  |  |  |  |  |  |  |  |
| 14 | Pc.c000403290.Contig1 | 1646 | - | 1409073 | 1410773 |  |  |  |  |  |  |  |  |  |  |  |  |  |  |
| 14 | Pb.c002112900.Contig1 | 937 | + | 1410560 | 1411485 | cRMPc13067 | 25071 | 52 | 1387446 | 1412517 |  |  |  |  |  |  |  |  |  |
| 14 | Pb.c000901749.Contig1 | 2161 | + | 1412569 | 1414646 |  |  |  |  |  |  |  |  |  |  |  |  |  |  |
| 14 | MALPY02294 | 2145 | + | 1412893 | 1414942 | cRMPc13068 | 2373 | 1944 | 1412569 | 1414942 |  | 247 | 1400 |  |  |  |  |  |  |
| 14 | MALPY01939 | 5297 | + | 1416886 | 1422179 |  |  |  |  |  |  | 247 |  |  |  |  |  |  |  |
| 14 | Pc.c001103801.Contig1 | 2529 | + | 1416891 | 1419078 |  |  |  |  |  |  |  |  |  |  |  |  |  |  |
| 14 | Pb.c000801355.Contig2 | 1389 | - | 1416952 | 1418377 |  |  |  |  |  |  |  |  |  |  |  |  |  |  |
| 14 | MALPY03492 | 857 | + | 1417970 | 1418818 |  |  |  |  |  |  |  |  |  |  |  |  |  |  |
| 14 | Pb.c001702949.Contig1 | 4099 | - | 1419424 | 1423532 |  |  |  |  |  |  |  |  |  |  |  |  |  |  |
| 14 | Pc.c001103716.Contig1 | 2340 | + | 1419513 | 1421847 | cRMPc13069 | 6646 | 2698 | 1416886 | 1423532 |  |  |  | cRMPs1321 | 98542 | 2698 | 1324990 | 1423532 |  |
| 14 | MALPY02609 | 6105 | + | 1426230 | 1432707 |  |  |  |  |  | 323 |  |  |  |  |  |  |  |  |
| 14 | Pb.c000601586.Contig1 | 8525 | - | 1426844 | 1436008 |  |  |  |  |  |  |  |  |  |  |  |  |  |  |
| 14 | PcContig318 | 16829 | + | 1427008 | 1445457 |  |  |  |  |  |  |  |  |  |  |  |  |  |  |
| 14 | MALPY02144 | 6608 | - | 1432781 | 1440266 |  |  |  |  |  | 323 | 414 | 3500 |  |  |  |  |  |  |
| 14 | Pb.c001201935.Contig1 | 11064 | - | 1438665 | 1449620 |  |  |  |  |  |  |  |  |  |  |  |  |  |  |
| 14 | MALPY01087 | 6719 | - | 1440995 | 1447741 |  |  |  |  |  | 87 | 414 |  |  |  |  |  |  |  |
| man14 | MALPY02662 | 2383 | - | 1444574 | 1447317 | cRMPc13070 | 23390 | 3412 | 1426230 | 1449620 | 87 |  |  | cRMPs1322 | 23390 | 3412 | 1426230 | 1449620 |  |
| 14 | Pc.c000703412.Contig1 | 4727 | - | 1453032 | 1457759 |  |  |  |  |  |  |  |  |  |  |  |  |  |  |
| 14 | Pb.c000601576.Contig1 | 5302 | - | 1454072 | 1459349 |  |  |  |  |  |  |  |  |  |  |  |  |  |  |
| 14 | MALPY01696 | 4228 | + | 1454215 | 1458431 |  |  |  |  |  |  | 107 | 4000 |  |  |  |  |  |  |
| 14 | Pc.c001204414.Contig1 | 915 | - | 1457875 | 1458788 | cRMPc13071 | 6317 | 1060 | 1453032 | 1459349 |  |  |  |  |  |  |  |  |  |
| 14 | Pc.c001104470.Contig1 | 1015 | - | 1460409 | 1461424 | cRMPc13072 | 1015 | 3134 | 1460409 | 1461424 |  |  |  |  |  |  |  |  |  |
| 14 | MALPY03030 | 748 | + | 1464558 | 1465303 | cRMPc13073 | 745 | 364 | 1464558 | 1465303 |  | 107 | 800 |  |  |  |  |  |  |
| 14 | Pc.c001812352.Contig1 | 715 | - | 1465667 | 1466382 |  |  |  |  |  |  |  |  |  |  |  |  |  |  |
| 14 | Pb.c000801828.Contig1 | 4233 | + | 1465712 | 1470833 |  |  |  |  |  |  |  |  |  |  |  |  |  |  |
| 14 | MALPY01711 | 2453 | + | 1465727 | 1468180 |  |  |  |  |  |  | 107 |  |  |  |  |  |  |  |
| 14 | Pc.c001404745.Contig1 | 728 | - | 1466714 | 1467442 |  |  |  |  |  |  |  |  |  |  |  |  |  |  |
| 14 | MALPY01941 | 4406 | - | 1469427 | 1473783 |  |  |  |  |  |  | 127 | 4000 |  |  |  |  |  |  |
| 14 | PcContig502 | 2056 | - | 1470115 | 1472240 |  |  |  |  |  |  |  |  |  |  |  |  |  |  |
| 14 | Pb.c002006827.Contig1 | 1687 | + | 1470957 | 1472653 | cRMPc13074 | 8116 | 593 | 1465667 | 1473783 |  |  |  |  |  |  |  |  |  |
| 14 | Pb.c002007391.Contig1 | 701 | + | 1474376 | 1475077 | cRMPc13075 | 701 | 1306 | 1474376 | 1475077 |  |  |  |  |  |  |  |  |  |
| 14 | MALPY02128 | 2354 | - | 1476383 | 1478737 |  |  |  |  |  | 234 |  |  |  |  |  |  |  |  |
| 14 | Pb.c001904893.Contig1 | 920 | - | 1477066 | 1477986 |  |  |  |  |  |  |  |  |  |  |  |  |  |  |
| 14 | PbContig173 | 12059 | - | 1478193 | 1491359 |  |  |  |  |  |  |  |  |  |  |  |  |  |  |
| 14 | MALPY01297 | 12728 | - | 1478539 | 1492701 |  |  |  |  |  | 234 | 127 | 1200 |  |  |  |  |  |  |
| 14 | Pc.c001505433.Contig1 | 1205 | + | 1479040 | 1480245 |  |  |  |  |  |  |  |  |  |  |  |  |  |  |
| 14 | Pc.c000502981.Contig1 | 1765 | - | 1481224 | 1483226 |  |  |  |  |  |  |  |  |  |  |  |  |  |  |
| 14 | Pc.c001304175.Contig1 | 2051 | + | 1487821 | 1489866 |  |  |  |  |  |  |  |  |  |  |  |  |  |  |
| 14 | Pc.c000703789.Contig1 | 2816 | + | 1491161 | 1493935 |  |  |  |  |  |  |  |  |  |  |  |  |  |  |
| 14 | Pb.c001202039.Contig1 | 1222 | - | 1492101 | 1493324 |  |  |  |  |  |  |  |  |  |  |  |  |  |  |
| 14 | MALPY03047 | 656 | - | 1492905 | 1493570 | cRMPc13076 | 17552 | 2146 | 1476383 | 1493935 |  | 127 | 2500 |  |  |  |  |  |  |
| 14 | Pc.c000902736.Contig1 | 3522 | + | 1496081 | 1499597 |  |  |  |  |  |  |  |  |  |  |  |  |  |  |
| 14 | Pb.c001502511.Contig1 | 2347 | - | 1496189 | 1498530 |  |  |  |  |  |  |  |  |  |  |  |  |  |  |
| 14 | MALPY01084 | 14879 | + | 1496400 | 1513799 |  |  |  |  |  | 137 | 127 |  |  |  |  |  |  |  |
| 14 | PbContig240 | 7147 | - | 1499798 | 1507439 |  |  |  |  |  |  |  |  |  |  |  |  |  |  |
| 14 | Pc.c000402901.Contig1 | 5219 | - | 1504166 | 1509389 |  |  |  |  |  |  |  |  |  |  |  |  |  |  |
| 14 | PcContig131 | 3844 | - | 1510330 | 1514165 |  |  |  |  |  |  |  |  |  |  |  |  |  |  |
| 14 | Pb.c000801736.Contig1 | 4970 | - | 1511444 | 1516405 |  |  |  |  |  |  |  |  |  |  |  |  |  |  |
| man14 | MALPY01595 | 6520 | - | 1512900 | 1525805 |  |  |  |  |  | 137 |  |  |  |  |  |  |  |  |
| man14 | Pb.c001602050.Contig2 - BP | 8388 | - | 1522442 | 1532635 |  |  |  |  |  |  |  |  |  |  |  |  |  |  |
| 13 | MALPY01729 | 4530 | - | 1528072 | 1532675 |  |  |  |  |  |  | 129 | 1500 |  |  |  |  |  |  |
| 13 | Pc.c001505439.Contig1 | 1581 | + | 1528804 | 1530364 |  |  |  |  |  |  |  |  |  |  |  |  |  |  |
| 13 | PcContig738 | 831 | + | 1530604 | 1531435 | cRMPc13077 | 36594 | 943 | 1496081 | 1532675 |  |  |  |  |  |  |  |  |  |
| 13 | Pb.c001803409.Contig1 | 2518 | - | 1533618 | 1536136 |  |  |  |  |  |  |  |  |  |  |  |  |  |  |
| 13 | MALPY02401 | 4556 | - | 1533880 | 1538330 |  |  |  |  |  |  | 129 | 900 |  |  |  |  |  |  |
| 13 | Pb.c000901851.Contig1 | 3662 | - | 1536609 | 1543613 |  |  |  |  |  |  |  |  |  |  |  |  |  |  |
| 13 | Pc.c000803178.Contig1 | 5055 | - | 1538224 | 1543240 |  |  |  |  |  |  |  |  |  |  |  |  |  |  |
| 13 | MALPY02196 | 4686 | - | 1541815 | 1546513 |  |  |  |  |  |  | 129 - Ins | 800 |  |  |  |  |  |  |
| 13 | PcContig395 | 3896 | - | 1545652 | 1549521 |  |  |  |  |  |  |  |  |  |  |  |  |  |  |
| 13 | MALPY00279 | 16934 | - | 1547313 | 1565591 |  |  |  |  |  | 92 | 129 - Ins |  |  |  |  |  |  |  |
| 13 | Pc.c000002540.Contig1 | 4725 | + | 1551429 | 1556806 |  |  |  |  |  |  |  |  |  |  |  |  |  |  |
| 13 | Pb.c000001320.Contig1 | 5107 | - | 1559207 | 1564713 |  |  |  |  |  |  |  |  |  |  |  |  |  |  |
| 13 | Pc.c000002709.Contig1 | 1813 | + | 1560681 | 1562779 |  |  |  |  |  |  |  |  |  |  |  |  |  |  |
| 13 | PcContig296 | 1827 | - | 1563391 | 1565230 |  |  |  |  |  |  |  |  |  |  |  |  |  |  |
| JC13 | MALPY00202 | 3116 | + | 1565014 | 1568130 | cRMPc13078 | 34512 | 32 | 1533618 | 1568130 | 92 |  |  |  |  |  |  |  |  |
| man13 | MALPY00146A - Ins | 5057 | - | 1568162 | 1573551 |  |  |  |  |  | 92 |  |  |  |  |  |  |  |  |
| 13 | Pb.c002007030.Contig1 | 1326 | + | 1569143 | 1570469 |  |  |  |  |  |  |  |  |  |  |  |  |  |  |
| 13 | Pc.c001811748.Contig1 | 1360 | - | 1571030 | 1572387 |  |  |  |  |  |  |  |  |  |  |  |  |  |  |
| 13 | Pc.c001812930.Contig1 | 641 | - | 1573144 | 1573872 | cRMPc13079 | 5710 | 563 | 1568162 | 1573872 |  |  |  | cRMPs1323 | 120840 | 563 | 1453032 | 1573872 |  |
| 13 | MALPY00427 | 10375 | + | 1574435 | 1584469 |  |  |  |  |  |  | 10 | 1500 |  |  |  |  |  |  |
| 13 | PbContig295 | 6726 | - | 1575769 | 1582575 |  |  |  |  |  |  |  |  |  |  |  |  |  |  |
| 13 | Pc.c001003987.Contig1 | 684 | - | 1577125 | 1577812 |  |  |  |  |  |  |  |  |  |  |  |  |  |  |
| 13 | PcContig374 | 7432 | + | 1577403 | 1584764 |  |  |  |  |  |  |  |  |  |  |  |  |  |  |
| 13 | Pb.c001301836.Contig1 | 1900 | - | 1582163 | 1584080 | cRMPc13080 | 10329 | 221 | 1574435 | 1584764 |  |  |  |  |  |  |  |  |  |
| 13 | MALPY01416 | 2902 | + | 1584985 | 1588109 |  |  |  |  |  |  | 10 | 1800 |  |  |  |  |  |  |
| 13 | Pc.c001812632.Contig1 | 1055 | + | 1585015 | 1586133 |  |  |  |  |  |  |  |  |  |  |  |  |  |  |
| 13 | Pc.c001708051.Contig1 | 1485 | + | 1586386 | 1587923 | cRMPc13081 | 3124 | 709 | 1584985 | 1588109 |  |  |  |  |  |  |  |  |  |
| 13 | MALPY00573 | 4317 | - | 1588818 | 1593162 |  |  |  |  |  | 148B | 10 |  |  |  |  |  |  |  |
| 13 | PbContig371 | 5891 | + | 1589158 | 1595544 |  |  |  |  |  |  |  |  |  |  |  |  |  |  |
| 13 | Pc.c000503081.Contig1 | 1010 | - | 1589935 | 1590930 |  |  |  |  |  |  |  |  |  |  |  |  |  |  |
| 13 | MALPY02679 | 2294 | - | 1593367 | 1595643 |  |  |  |  |  | 148B | 147 | 900 |  |  |  |  |  |  |
| 13 | Pc.c000202199.Contig2 | 5109 | - | 1593716 | 1599091 |  |  |  |  |  |  |  |  |  |  |  |  |  |  |
| 13 | MALPY01469 | 3449 | - | 1596167 | 1599560 |  |  |  |  |  |  | 147 |  |  |  |  |  |  |  |
| 13 | Pb.c001402089.Contig1 | 3598 | + | 1596413 | 1600008 |  |  |  |  |  |  |  |  |  |  |  |  |  |  |
| 13 | MALPY02280 | 9891 | - | 1597776 | 1609160 |  |  |  |  |  |  | 169 | 2500 |  |  |  |  |  |  |
| 13 | Pc.c001203895.Contig1 | 2730 | - | 1606815 | 1609590 | cRMPc13082 | 20772 | 1142 | 1588818 | 1609590 |  |  |  |  |  |  |  |  |  |
| 13 | Pc.c001404715.Contig1 | 2524 | + | 1610732 | 1613256 |  |  |  |  |  |  |  |  |  |  |  |  |  |  |
| 13 | MALPY00441 | 5005 | + | 1611120 | 1616387 |  |  |  |  |  | 129 | 169 |  |  |  |  |  |  |  |
| 13 | Pb.c000301147.Contig2 | 4441 | - | 1611357 | 1616176 |  |  |  |  |  |  |  |  |  |  |  |  |  |  |
| 13 | Pc.c000803622.Contig1 | 614 | - | 1613035 | 1613646 |  |  |  |  |  |  |  |  |  |  |  |  |  |  |
| 13 | Pc.c001708826.Contig1 | 647 | + | 1613142 | 1613786 |  |  |  |  |  |  |  |  |  |  |  |  |  |  |
| 13 | PcContig484 | 2712 | + | 1613503 | 1616504 | cRMPc13083 | 5772 | 755 | 1610732 | 1616504 |  |  |  |  |  |  |  |  |  |
| TK13 | MALPY02647 | 5050 | - | 1617259 | 1618212 | cRMPc13084 | 953 | 17060 | 1617259 | 1618212 | 129 |  |  | cRMPs1324 | 43777 | 17060 | 1574435 | 1618212 |  |
| 13 | MALPY00752 | 3857 | + | 1635272 | 1639147 |  |  |  |  |  | 143 |  |  |  |  |  |  |  |  |
| 13 | PcContig249 | 3434 | - | 1638021 | 1641635 |  |  |  |  |  |  |  |  |  |  |  |  |  |  |
| 13 | MALPY00220 | 4583 | - | 1639224 | 1643804 |  |  |  |  |  | 143 |  |  |  |  |  |  |  |  |
| 13 | Pb.c001201601.Contig1 | 5974 | - | 1639893 | 1645924 | cRMPc13085 | 10652 | 1782 | 1635272 | 1645924 |  |  |  | cRMPs1325 | 10652 | 1782 | 1635272 | 1645924 |  |
| 13 | Pc.c000002894.Contig1 | 3219 | - | 1647706 | 1650928 |  |  |  |  |  |  |  |  |  |  |  |  |  |  |
| 13 | MALPY01158 | 8952 | - | 1648019 | 1657264 |  |  |  |  |  | 296 |  |  |  |  |  |  |  |  |
| 13 | Pb.c000401166.Contig1 | 5268 | + | 1648199 | 1653425 |  |  |  |  |  |  |  |  |  |  |  |  |  |  |
| 13 | Pc.c000803701.Contig1 | 6010 | + | 1653434 | 1659527 |  |  |  |  |  |  |  |  |  |  |  |  |  |  |
| 13 | Pb.c000101323.Contig1 | 10347 | - | 1654544 | 1664835 |  |  |  |  |  |  |  |  |  |  |  |  |  |  |
| 13 | MALPY00247 | 4141 | + | 1657227 | 1661293 |  |  |  |  |  | 296 | 255 | 1800 |  |  |  |  |  |  |
| 13 | Pc.c000302971.Contig1 | 2476 | - | 1659632 | 1662094 |  |  |  |  |  |  |  |  |  |  |  |  |  |  |
| 13 | MALPY00978 | 2261 | - | 1661362 | 1663837 |  |  |  |  |  | 296 | 23 | 500 |  |  |  |  |  |  |
| 13 | MALPY01172 | 3105 | - | 1662069 | 1665298 |  |  |  |  |  |  | 255 |  |  |  |  |  |  |  |
| 13 | PcContig72 | 3517 | + | 1662204 | 1665908 |  |  |  |  |  |  |  |  |  |  |  |  |  |  |
| 13 | MALPY02999 | 774 | - | 1663718 | 1664492 |  |  |  |  |  |  | 23 |  |  |  |  |  |  |  |
| TK13 | MALPY01304 | 10116 | + | 1664475 | 1674576 | cRMPc13086 | 26870 | 214 | 1647706 | 1674576 | 33 |  |  |  |  |  |  |  |  |
| 13 | MALPY03053 | 1957 | - | 1674790 | 1676747 |  |  |  |  |  | 33 |  |  |  |  |  |  |  |  |
| 13 | PcContig207 | 2626 | - | 1675377 | 1678280 |  |  |  |  |  |  |  |  |  |  |  |  |  |  |
| 13 | Pb.c000401228.Contig1 | 4059 | - | 1675580 | 1679940 |  |  |  |  |  |  |  |  |  |  |  |  |  |  |
| 13 | MALPY00557 | 10214 | + | 1676861 | 1686865 |  |  |  |  |  | 33 |  |  |  |  |  |  |  |  |
| 13 | Pc.c001707724.Contig1 | 1555 | + | 1678503 | 1679991 |  |  |  |  |  |  |  |  |  |  |  |  |  |  |
| 13 | Pb.c001301500.Contig2 | 5202 | - | 1679818 | 1685107 |  |  |  |  |  |  |  |  |  |  |  |  |  |  |
| 13 | PcContig463 | 3932 | + | 1680895 | 1684970 | cRMPc13087 | 12075 | 284 | 1674790 | 1686865 |  |  |  |  |  |  |  |  |  |
| 13 | MALPY00799 | 13457 | - | 1687149 | 1700606 |  |  |  |  |  | 33 |  |  |  |  |  |  |  |  |
| man13 | PcContig6403 | 3344 | - | 1697600 | 1700944 |  |  |  |  |  |  |  |  |  |  |  |  |  | MALPY00799 |
| 13 | Pc.c000202197.Contig1 | 2411 | - | 1698636 | 1701047 |  |  |  |  |  |  |  |  |  |  |  |  |  |  |
| man13 | MALPY00919 | 2765 | - | 1700600 | 1703365 |  |  |  |  |  | 33 |  |  |  |  |  |  |  | \| |
| man13 | PbContig6116 | 1644 | + | 1702081 | 1703725 |  |  |  |  |  |  |  |  |  |  |  |  |  | MALPY00469 |
| 13 | MALPY00469 | 6173 | - | 1702112 | 1707929 |  |  |  |  |  | 33 |  |  |  |  |  |  |  |  |
| 13 | Pb.c001904490.Contig1 | 2270 | + | 1703115 | 1704959 |  |  |  |  |  |  |  |  |  |  |  |  |  |  |
| 13 | Pc.c000803273.Contig1 | 1904 | + | 1703901 | 1706006 |  |  |  |  |  |  |  |  |  |  |  |  |  |  |
| 13 | Pb.c000801751.Contig1 | 7029 | + | 1705111 | 1713017 |  |  |  |  |  |  |  |  |  |  |  |  |  |  |
| 13 | PcContig155 | 9016 | - | 1706287 | 1717294 |  |  |  |  |  |  |  |  |  |  |  |  |  |  |
| 13 | MALPY01857 | 5200 | + | 1708310 | 1713683 |  |  |  |  |  |  |  |  |  |  |  |  |  |  |
| 13 | Pb.c002112784.Contig1 | 658 | + | 1713915 | 1714573 | cRMPc13088 | 30145 | 85 | 1687149 | 1717294 |  |  |  | cRMPs1326 | 69588 | 85 | 1647706 | 1717294 |  |
| 13 | Pc.c000503104.Contig1 | 2053 | - | 1717379 | 1719441 | cRMPc13089 | 2062 | 7468 | 1717379 | 1719441 |  |  |  | cRMPs1327 | 2062 | 7468 | 1717379 | 1719441 |  |
| 13 | PcContig241 | 14116 | - | 1726909 | 1741025 |  |  |  |  |  |  |  |  |  |  |  |  |  |  |
| 13 | MALPY00346 | 14387 | - | 1729802 | 1744183 |  |  |  |  |  |  |  |  |  |  |  |  |  |  |
| 13 | Pb.c000601178.Contig1 | 4538 | - | 1732637 | 1737175 |  |  |  |  |  |  |  |  |  |  |  |  |  |  |
| 13 | MALPY00276 | 27227 | - | 1738958 | 1768667 |  |  |  |  |  |  |  |  |  |  |  |  |  |  |
| 13 | PcContig347 | 3850 | - | 1739307 | 1743264 |  |  |  |  |  |  |  |  |  |  |  |  |  |  |
| 13 | Pb.c001602026.Contig2 | 15188 | + | 1739334 | 1755099 |  |  |  |  |  |  |  |  |  |  |  |  |  |  |
| 13 | MALPY03362 | 803 | - | 1740746 | 1741549 |  |  |  |  |  |  |  |  |  |  |  |  |  |  |
| 13 | MALPY02989 | 987 | - | 1740831 | 1741814 |  |  |  |  |  |  |  |  |  |  |  |  |  |  |
| 13 | Pc.c001605952.Contig1 | 1385 | - | 1744622 | 1746220 |  |  |  |  |  |  |  |  |  |  |  |  |  |  |
| 13 | Pc.c000503000.Contig1 | 1766 | - | 1752083 | 1753963 |  |  |  |  |  |  |  |  |  |  |  |  |  |  |
| 13 | Pb.c000901477.Contig1 | 3955 | + | 1755236 | 1759267 |  |  |  |  |  |  |  |  |  |  |  |  |  |  |
| 13 | Pc.c000602894.Contig1 | 4241 | + | 1756519 | 1761408 |  |  |  |  |  |  |  |  |  |  |  |  |  |  |
| 13 | Pb.c001301854.Contig1 | 4696 | - | 1760169 | 1765835 |  |  |  |  |  |  |  |  |  |  |  |  |  |  |
| 13 | PcContig28 | 1254 | - | 1761539 | 1762985 |  |  |  |  |  |  |  |  |  |  |  |  |  |  |
| 13 | MALPY03019 | 611 | - | 1762389 | 1762979 |  |  |  |  |  |  | 59 | 4300 |  |  |  |  |  |  |
| 13 | Pc.c001304902.Contig1 | 844 | - | 1765990 | 1766834 |  |  |  |  |  |  |  |  |  |  |  |  |  |  |
| 13 | Pb.c001602024.Contig1 | 2677 | - | 1766259 | 1769015 |  |  |  |  |  |  |  |  |  |  |  |  |  |  |
| 13 | Pc.c000903412.Contig1 | 3529 | + | 1767706 | 1771288 |  |  |  |  |  |  |  |  |  |  |  |  |  |  |
| man13 | MALPY00414 - Ins | 10707 | - | 1768588 | 1780106 |  |  |  |  |  |  | 59 |  |  |  |  |  |  |  |
| 13 | Pb.c001602460.Contig1 | 7391 | + | 1769082 | 1776473 |  |  |  |  |  |  |  |  |  |  |  |  |  |  |
| 13 | Pc.c001605885.Contig1 | 4957 | + | 1776442 | 1781391 |  |  |  |  |  |  |  |  |  |  |  |  |  |  |
| 13 | PbContig59 | 2801 | - | 1777479 | 1780280 |  |  |  |  |  |  |  |  |  |  |  |  |  |  |
| 13 | MALPY02644 | 4143 | - | 1780977 | 1785085 |  |  |  |  |  |  |  |  |  |  |  |  |  |  |
| 13 | PcContig283 | 1492 | + | 1783493 | 1784988 |  |  |  |  |  |  |  |  |  |  |  |  |  |  |
| 13 | PbContig377 | 13537 | - | 1783774 | 1798104 |  |  |  |  |  |  |  |  |  |  |  |  |  |  |
| 13 | MALPY01549 | 8088 | + | 1789133 | 1797221 | cRMPc13090 | 71195 | 220 | 1726909 | 1798104 |  | 310 | 1300 |  |  |  |  |  |  |
| 13 | MALPY01358 | 9199 | - | 1798324 | 1807370 |  |  |  |  |  |  | 310 | 4000 |  |  |  |  |  |  |
| 13 | Pb.c001101844.Contig1 | 5251 | - | 1801472 | 1806549 | cRMPc13091 | 9046 | 550 | 1798324 | 1807370 |  |  |  |  |  |  |  |  |  |
| Centromere \* - PCR 4000 bp | |  |  | 1807000 |  |  |  |  |  |  |  |  |  |  |  |  |  |  |  |
| 13 | Pc.c001204062.Contig1 | 3915 | - | 1807920 | 1811955 |  |  |  |  |  |  |  |  |  |  |  |  |  |  |
| 13 | Pb.c001301808.Contig1 | 4622 | + | 1808244 | 1812914 |  |  |  |  |  |  |  |  |  |  |  |  |  |  |
| 13 | MALPY00394 | 3173 | + | 1808806 | 1812036 |  |  |  |  |  |  | 310 |  |  |  |  |  |  |  |
| 13 | MALPY03551 | 512 | - | 1810766 | 1811287 |  |  |  |  |  |  | 318 | 2500 |  |  |  |  |  |  |
| 13 | MALPY02330 | 4739 | + | 1812096 | 1816542 |  |  |  |  |  |  | 318 |  |  |  |  |  |  |  |
| 13 | Pb.c002113125.Contig1 | 830 | - | 1813911 | 1814741 |  |  |  |  |  |  |  |  |  |  |  |  |  |  |
| 13 | Pb.c002110446.Contig1 | 1805 | - | 1814602 | 1816410 | cRMPc13092 | 8622 | 433 | 1807920 | 1816542 |  |  |  | cRMPs1328 | 89633 | 433 | 1726909 | 1816542 |  |
| 13 | MALPY03485 | 691 | + | 1816975 | 1817672 |  |  |  |  |  |  | 266 | 1300 |  |  |  |  |  |  |
| 13 | MALPY02500 | 3557 | - | 1817592 | 1821149 | cRMPc13093 | 4174 | 2323 | 1816975 | 1821149 |  |  |  |  |  |  |  |  |  |
| 13 | MALPY00074 | 10987 | + | 1823472 | 1834453 |  |  |  |  |  | 561 | 266 |  |  |  |  |  |  |  |
| 13 | Pb.c002007707.Contig1 | 1355 | + | 1830997 | 1832352 |  |  |  |  |  |  |  |  |  |  |  |  |  |  |
| 13 | Pb.c001602571.Contig1 | 5756 | + | 1833545 | 1839301 |  |  |  |  |  |  |  |  |  |  |  |  |  |  |
| 13 | Pc.c001204233.Contig1 | 4239 | - | 1834643 | 1838903 |  |  |  |  |  |  |  |  |  |  |  |  |  |  |
| 13 | MALPY03170 | 1635 | - | 1835455 | 1837087 |  |  |  |  |  | 561 |  |  |  |  |  |  |  |  |
| 13 | Pb.c000701207.Contig1 | 9921 | - | 1837144 | 1847230 |  |  |  |  |  |  |  |  |  |  |  |  |  |  |
| TK13 | MALPY02774 | 2413 | - | 1838020 | 1839410 |  |  |  |  |  | 561 |  |  |  |  |  |  |  |  |
| 13 | MALPY00356 | 9006 | + | 1841145 | 1850242 |  |  |  |  |  |  |  |  |  |  |  |  |  |  |
| 13 | Pc.c000602848.Contig1 | 3075 | - | 1843111 | 1846324 | cRMPc13094 | 26770 | 9973 | 1823472 | 1850242 |  |  |  | cRMPs1329 | 33267 | 9973 | 1816975 | 1850242 |  |
| 13 | MALPY01251 | 8588 | + | 1860215 | 1868617 |  |  |  |  |  | 1028 |  |  |  |  |  |  |  |  |
| 13 | PbContig217 | 11476 | + | 1861112 | 1872840 |  |  |  |  |  |  |  |  |  |  |  |  |  |  |
| 13 | Pc.c001204350.Contig1 | 1030 | - | 1861207 | 1862305 |  |  |  |  |  |  |  |  |  |  |  |  |  |  |
| 13 | Pc.c001605953.Contig1 | 3029 | - | 1864394 | 1867629 |  |  |  |  |  |  |  |  |  |  |  |  |  |  |
| 13 | Pc.c001707896.Contig1 | 1525 | - | 1868072 | 1869597 |  |  |  |  |  |  |  |  |  |  |  |  |  |  |
| 13 | MALPY02170 | 2835 | - | 1868698 | 1871565 |  |  |  |  |  | 1028 |  |  |  |  |  |  |  |  |
| 13 | PcContig787 | 6714 | - | 1870032 | 1879007 |  |  |  |  |  |  |  |  |  |  |  |  |  |  |
| 13 | MALPY02052 | 5837 | - | 1873361 | 1879466 |  |  |  |  |  |  |  |  |  |  |  |  |  |  |
| 13 | PbContig268 | 12274 | - | 1874842 | 1887114 |  |  |  |  |  |  |  |  |  |  |  |  |  |  |
| 13 | MALPY00606 | 13289 | - | 1881605 | 1894823 |  |  |  |  |  | 36 | 244B | 2500 |  |  |  |  |  |  |
| 13 | PbContig76 | 4533 | - | 1888695 | 1893325 |  |  |  |  |  |  |  |  |  |  |  |  |  |  |
| 13 | Pc.c001103907.Contig1 | 2316 | - | 1890729 | 1893177 | cRMPc13095 | 34608 | 1252 | 1860215 | 1894823 |  |  |  |  |  |  |  |  |  |
| 13 | MALPY00134 | 3298 | - | 1896075 | 1899084 |  |  |  |  |  | 36 |  |  |  |  |  |  |  |  |
| 13 | Pb.c000501174.Contig1 | 8348 | + | 1897744 | 1906113 |  |  |  |  |  |  |  |  |  |  |  |  |  |  |
| 13 | Pc.c001605920.Contig1 | 2486 | - | 1897952 | 1900438 |  |  |  |  |  |  |  |  |  |  |  |  |  |  |
| 13 | MALPY01298 | 3203 | - | 1899180 | 1902374 |  |  |  |  |  | 36 |  |  |  |  |  |  |  |  |
| 13 | Pc.c001103762.Contig1 | 6464 | - | 1900341 | 1908181 |  |  |  |  |  |  |  |  |  |  |  |  |  |  |
| 13 | MALPY00599 | 8630 | + | 1903617 | 1912192 |  |  |  |  |  | 36 |  |  |  |  |  |  |  |  |
| 13 | MALPY03204 | 598 | - | 1906088 | 1906683 |  |  |  |  |  |  | 130 | 900 |  |  |  |  |  |  |
| 13 | Pb.c001202011.Contig1 | 3669 | + | 1908180 | 1911852 |  |  |  |  |  |  |  |  |  |  |  |  |  |  |
| 13 | Pc.c000203306.Contig1 | 792 | - | 1908693 | 1909485 |  |  |  |  |  |  |  |  |  |  |  |  |  |  |
| 13 | Pc.c001605870.Contig1 | 1980 | - | 1909439 | 1911434 |  |  |  |  |  |  |  |  |  |  |  |  |  |  |
| 13 | MALPY03256 | 764 | + | 1909914 | 1910668 | cRMPc13096 | 16117 | 533 | 1896075 | 1912192 |  |  |  | cRMPs1330 | 51977 | 533 | 1860215 | 1912192 |  |
| 13 | PbContig1 | 13178 | - | 1912725 | 1926919 |  |  |  |  |  |  |  |  |  |  |  |  |  |  |
| 13 | MALPY02717 | 3811 | + | 1912829 | 1916483 |  |  |  |  |  | 26B |  |  |  |  |  |  |  |  |
| 13 | Pc.c001708936.Contig1 | 914 | - | 1913042 | 1913956 |  |  |  |  |  |  |  |  |  |  |  |  |  |  |
| 13 | Pc.c001304219.Contig1 | 2954 | - | 1914201 | 1917237 |  |  |  |  |  |  |  |  |  |  |  |  |  |  |
| 13 | MALPY02486 | 3358 | - | 1915312 | 1918575 |  |  |  |  |  | 26B |  |  |  |  |  |  |  |  |
| 13 | MALPY00706 | 4164 | - | 1919291 | 1923425 |  |  |  |  |  | 26B |  |  |  |  |  |  |  |  |
| 13 | PcContig208 | 3722 | + | 1920745 | 1924488 |  |  |  |  |  |  |  |  |  |  |  |  |  |  |
| 13 | MALPY01858 | 13213 | - | 1922764 | 1937154 |  |  |  |  |  | 26B |  |  |  |  |  |  |  |  |
| 13 | Pc.c001104159.Contig1 | 2850 | + | 1924760 | 1927755 |  |  |  |  |  |  |  |  |  |  |  |  |  |  |
| 13 | Pb.c000101344.Contig1 | 9954 | + | 1926939 | 1939127 |  |  |  |  |  |  |  |  |  |  |  |  |  |  |
| JC13 | MALPY03216 | 453 | + | 1930315 | 1930801 |  |  |  |  |  |  |  |  |  |  |  |  |  |  |
| 13 | Pc.c001405308.Contig1 | 2010 | + | 1931530 | 1933536 |  |  |  |  |  |  |  |  |  |  |  |  |  |  |
| 13 | Pc.c000303496.Contig1 | 1130 | + | 1934796 | 1935923 |  |  |  |  |  |  |  |  |  |  |  |  |  |  |
| 13 | Pc.c000303042.Contig1 | 2931 | + | 1936666 | 1939585 |  |  |  |  |  |  |  |  |  |  |  |  |  |  |
| 13 | MALPY00504 | 11558 | + | 1937731 | 1953183 |  |  |  |  |  | 26B |  |  |  |  |  |  |  |  |
| 13 | PbContig272 | 7914 | + | 1938548 | 1947327 |  |  |  |  |  |  |  |  |  |  |  |  |  |  |
| 13 | Pc.c000603393.Contig1 | 1117 | - | 1939081 | 1940198 |  |  |  |  |  |  |  |  |  |  |  |  |  |  |
| 13 | Pc.c001003874.Contig1 | 935 | - | 1940427 | 1941320 |  |  |  |  |  |  |  |  |  |  |  |  |  |  |
| 13 | Pc.c000102530.Contig1 | 3065 | - | 1941293 | 1944415 |  |  |  |  |  |  |  |  |  |  |  |  |  |  |
| 13 | Pc.c000101979.Contig1 | 2899 | - | 1945243 | 1948136 |  |  |  |  |  |  |  |  |  |  |  |  |  |  |
| 13 | Pb.c000801714.Contig1 | 2884 | + | 1952485 | 1955528 |  |  |  |  |  |  |  |  |  |  |  |  |  |  |
| 13 | MALPY02176 | 4889 | + | 1953431 | 1958696 |  |  |  |  |  | 26B |  |  |  |  |  |  |  |  |
| 13 | Pc.c000302409.Contig2 | 5406 | - | 1954658 | 1960616 |  |  |  |  |  |  |  |  |  |  |  |  |  |  |
| 13 | Pb.c000101381.Contig1 | 4317 | + | 1956745 | 1961794 |  |  |  |  |  |  |  |  |  |  |  |  |  |  |
| 13 | MALPY00576 | 3199 | + | 1959000 | 1962405 | cRMPc13097 | 49680 | 586 | 1912725 | 1962405 | 26B |  |  |  |  |  |  |  |  |
| 13 | MALPY00476 | 6737 | + | 1962991 | 1969749 |  |  |  |  |  | 26B | 46 | 700 |  |  |  |  |  |  |
| 13 | PbContig22 | 9160 | - | 1963801 | 1974755 |  |  |  |  |  |  |  |  |  |  |  |  |  |  |
| 13 | Pc.c000703445.Contig1 | 3607 | + | 1964700 | 1969058 |  |  |  |  |  |  |  |  |  |  |  |  |  |  |
| 13 | Pc.c001103719.Contig1 | 6854 | - | 1970506 | 1978259 |  |  |  |  |  |  |  |  |  |  |  |  |  |  |
| 13 | MALPY01487 | 2568 | - | 1971154 | 1973746 |  |  |  |  |  |  | 46 | 1300 |  |  |  |  |  |  |
| 13 | MALPY00372 | 9231 | - | 1975609 | 1984050 |  |  |  |  |  |  | 46 | 1100 |  |  |  |  |  |  |
| 13 | Pb.c002006958.Contig1 | 1797 | - | 1975890 | 1977642 |  |  |  |  |  |  |  |  |  |  |  |  |  |  |
| 13 | Pb.c001502853.Contig1 | 2659 | - | 1977934 | 1980793 |  |  |  |  |  |  |  |  |  |  |  |  |  |  |
| 13 | Pb.c001502431.Contig1 | 2645 | - | 1980478 | 1983210 |  |  |  |  |  |  |  |  |  |  |  |  |  |  |
| 13 | Pc.c000402753.Contig1 | 538 | - | 1982232 | 1982770 |  |  |  |  |  |  |  |  |  |  |  |  |  |  |
| 13 | Pc.c000402754.Contig1 | 5441 | - | 1983418 | 1989484 |  |  |  |  |  |  |  |  |  |  |  |  |  |  |
| man13 | MALPY00591 - Exp | 10334 | + | 1984686 | 1995759 |  |  |  |  |  |  | 46 |  |  |  |  |  |  |  |
| man13 | Pb.c001001417.Contig3 - Exp | 8745 | + | 1988349 | 1998355 |  |  |  |  |  |  |  |  |  |  |  |  |  |  |
| man13 | PcContig173 - Ins | 9532 | - | 1994511 | 2005472 |  |  |  |  |  |  |  |  |  |  |  |  |  |  |
| man13 | MALPY00867 - Ins | 19695 | + | 1996900 | 2018206 |  |  |  |  |  | 455 |  |  |  |  |  |  |  |  |
| 13 | Pb.c000101036.Contig2 | 4610 | + | 1998678 | 2003520 |  |  |  |  |  |  |  |  |  |  |  |  |  |  |
| 13 | Pb.c000101355.Contig1 | 827 | + | 2002769 | 2003581 |  |  |  |  |  |  |  |  |  |  |  |  |  |  |
| 13 | Pb.c000101353.Contig1 | 2385 | + | 2003541 | 2006106 |  |  |  |  |  |  |  |  |  |  |  |  |  |  |
| 13 | PbContig188 | 8666 | - | 2017712 | 2026928 |  |  |  |  |  |  |  |  |  |  |  |  |  |  |
| 13 | MALPY00401 | 3172 | + | 2018017 | 2021192 |  |  |  |  |  | 455 |  |  |  |  |  |  |  |  |
| 13 | PcContig638 | 2106 | - | 2019753 | 2021838 |  |  |  |  |  |  |  |  |  |  |  |  |  |  |
| 13 | MALPY00400 | 11525 | - | 2021373 | 2033042 |  |  |  |  |  | 455 | 97 | 1200 |  |  |  |  |  |  |
| 13 | Pc.c001504968.Contig1 | 2042 | - | 2025383 | 2027425 |  |  |  |  |  |  |  |  |  |  |  |  |  |  |
| 13 | Pc.c000902721.Contig1 | 5263 | - | 2028537 | 2033852 |  |  |  |  |  |  |  |  |  |  |  |  |  |  |
| 13 | Pb.c000401763.Contig1 | 3427 | - | 2029176 | 2032681 | cRMPc13098 | 70861 | 690 | 1962991 | 2033852 |  |  |  |  |  |  |  |  |  |
| 13 | MALPY02098 | 6411 | + | 2034542 | 2041508 |  |  |  |  |  |  | 97 |  |  |  |  |  |  |  |
| 13 | Pb.c000801369.Contig1 | 3117 | + | 2034836 | 2038016 |  |  |  |  |  |  |  |  |  |  |  |  |  |  |
| 13 | MALPY03532 | 830 | + | 2037806 | 2038648 |  |  |  |  |  |  |  |  |  |  |  |  |  |  |
| 13 | Pb.c000501507.Contig1 | 3491 | + | 2038016 | 2042093 |  |  |  |  |  |  |  |  |  |  |  |  |  |  |
| 13 | Pc.c000302397.Contig3 | 5619 | - | 2038049 | 2044432 |  |  |  |  |  |  |  |  |  |  |  |  |  |  |
| 13 | Pb.c000601532.Contig1 | 4266 | + | 2042667 | 2047117 |  |  |  |  |  |  |  |  |  |  |  |  |  |  |
| 13 | MALPY01555 | 16108 | - | 2043826 | 2060526 |  |  |  |  |  | 592 |  |  |  |  |  |  |  |  |
| 13 | Pc.c001604988.Contig1 | 2109 | - | 2045113 | 2047222 |  |  |  |  |  |  |  |  |  |  |  |  |  |  |
| 13 | Pb.c002007117.Contig1 | 2780 | - | 2047810 | 2050843 |  |  |  |  |  |  |  |  |  |  |  |  |  |  |
| 13 | Pc.c000303047.Contig1 | 914 | - | 2047989 | 2049047 |  |  |  |  |  |  |  |  |  |  |  |  |  |  |
| 13 | Pb.c000901930.Contig1 | 602 | + | 2048768 | 2049325 |  |  |  |  |  |  |  |  |  |  |  |  |  |  |
| 13 | Pb.c001803705.Contig1 | 624 | - | 2048773 | 2049352 |  |  |  |  |  |  |  |  |  |  |  |  |  |  |
| JC13 | MALPY03299 | 1487 | + | 2049044 | 2050531 |  |  |  |  |  |  |  |  |  |  |  |  |  |  |
| 13 | PcContig518 | 5093 | - | 2049303 | 2055146 |  |  |  |  |  |  |  |  |  |  |  |  |  |  |
| 13 | Pb.c000601558.Contig1 | 2254 | - | 2051918 | 2054190 |  |  |  |  |  |  |  |  |  |  |  |  |  |  |
| 13 | PcContig282 | 3526 | - | 2055459 | 2059009 |  |  |  |  |  |  |  |  |  |  |  |  |  |  |
| 13 | Pb.c000801423.Contig1 | 4943 | + | 2057305 | 2062278 |  |  |  |  |  |  |  |  |  |  |  |  |  |  |
| 13 | PcContig225 | 3482 | - | 2060408 | 2063890 | cRMPc13099 | 29348 | 578 | 2034542 | 2063890 |  |  |  |  |  |  |  |  |  |
| man13 | MALPY00876 | 4471 | - | 2064468 | 2069000 |  |  |  |  |  | 592 |  |  |  |  |  |  |  |  |
| 13 | MALPY00580 | 11678 | + | 2068706 | 2080892 |  |  |  |  |  | 431 |  |  |  |  |  |  |  |  |
| 13 | Pc.c001605856.Contig1 | 1225 | + | 2071150 | 2072375 |  |  |  |  |  |  |  |  |  |  |  |  |  |  |
| 13 | Pb.c000501398.Contig1 | 4426 | + | 2071568 | 2076313 |  |  |  |  |  |  |  |  |  |  |  |  |  |  |
| 13 | PcContig67 | 3071 | + | 2075045 | 2078556 |  |  |  |  |  |  |  |  |  |  |  |  |  |  |
| 13 | Pb.c001904854.Contig1 | 955 | + | 2077412 | 2078367 | cRMPc13100 | 16424 | 1521 | 2064468 | 2080892 |  |  |  |  |  |  |  |  |  |
| 13 | Pb.c001702954.Contig1 | 2390 | + | 2082413 | 2084806 |  |  |  |  |  |  |  |  |  |  |  |  |  |  |
| 13 | MALPY00753 | 12430 | + | 2082571 | 2095422 |  |  |  |  |  | 431 |  |  |  |  |  |  |  |  |
| 13 | PbContig111 | 7715 | + | 2085517 | 2094390 |  |  |  |  |  |  |  |  |  |  |  |  |  |  |
| 13 | Pc.c000602823.Contig1 | 4595 | - | 2086099 | 2090955 | cRMPc13101 | 13009 | 26 | 2082413 | 2095422 |  |  |  |  |  |  |  |  |  |
| 13 | Pb.c000300832.Contig1 | 5005 | + | 2095448 | 2100595 |  |  |  |  |  |  |  |  |  |  |  |  |  |  |
| 13 | PcContig506 | 6587 | + | 2095606 | 2102440 |  |  |  |  |  |  |  |  |  |  |  |  |  |  |
| 13 | MALPY02546 | 14348 | - | 2095709 | 2110508 |  |  |  |  |  | 431 |  |  |  |  |  |  |  |  |
| 13 | PcContig723 | 5648 | - | 2104054 | 2110526 |  |  |  |  |  |  |  |  |  |  |  |  |  |  |
| 13 | Pb.c000701755.Contig1 | 702 | - | 2105114 | 2105846 |  |  |  |  |  |  |  |  |  |  |  |  |  |  |
| 13 | Pb.c000801416.Contig2 | 6577 | - | 2106300 | 2113329 |  |  |  |  |  |  |  |  |  |  |  |  |  |  |
| 13 | MALPY01789 | 6002 | - | 2109738 | 2115737 |  |  |  |  |  | 431 |  |  |  |  |  |  |  |  |
| 13 | MALPY02440 | 6256 | - | 2112696 | 2118996 |  |  |  |  |  | 948 |  |  |  |  |  |  |  |  |
| man13 | PbContig389 - Ins | 9735 | - | 2113087 | 2123558 |  |  |  |  |  |  |  |  |  |  |  |  |  |  |
| 13 | Pc.c000202699.Contig1 | 1973 | + | 2116846 | 2118774 |  |  |  |  |  |  |  |  |  |  |  |  |  |  |
| 13 | MALPY00421 | 3008 | + | 2118951 | 2122781 |  |  |  |  |  | 948 | 271 | 3000 |  |  |  |  |  |  |
| 13 | Pc.c001707881.Contig1 | 934 | + | 2120305 | 2121190 |  |  |  |  |  |  |  |  |  |  |  |  |  |  |
| 13 | Pc.c000703896.Contig1 | 1039 | + | 2121881 | 2122920 | cRMPc13102 | 28110 | 4381 | 2095448 | 2123558 |  |  |  |  |  |  |  |  |  |
| 13 | Pb.c000101418.Contig1 | 6648 | - | 2127939 | 2134901 |  |  |  |  |  |  |  |  |  |  |  |  |  |  |
| 13 | MALPY00153 | 2143 | - | 2128655 | 2130801 |  |  |  |  |  | 741B | 271 | 2200 |  |  |  |  |  |  |
| 13 | MALPY03371 | 1000 | - | 2132350 | 2133350 |  |  |  |  |  |  | 271 |  |  |  |  |  |  |  |
| man13 | MALPY00161 | 6321 | + | 2134786 | 2141977 | cRMPc13103 | 14038 | 915 | 2127939 | 2141977 |  |  |  | cRMPs1331 | 229252 | 915 | 1912725 | 2141977 |  |
| 13 | MALPY01289 | 2784 | - | 2142892 | 2144934 |  |  |  |  |  | 329 |  |  |  |  |  |  |  |  |
| 13 | Pb.c000601247.Contig1 | 14579 | - | 2144098 | 2157944 |  |  |  |  |  |  |  |  |  |  |  |  |  |  |
| 13 | Pc.c001505787.Contig1 | 928 | - | 2144655 | 2145595 |  |  |  |  |  |  |  |  |  |  |  |  |  |  |
| 13 | MALPY00701 | 8004 | + | 2145173 | 2151957 |  |  |  |  |  | 329 |  |  |  |  |  |  |  |  |
| JC13 | MALPY03474 | 533 | + | 2146220 | 2146753 |  |  |  |  |  |  | 33 | 8000 |  |  |  |  |  |  |
| 13 | Pc.c001103105.Contig3 | 2394 | - | 2147111 | 2149505 |  |  |  |  |  |  |  |  |  |  |  |  |  |  |
| 13 | Pc.c001404730.Contig1 | 2637 | + | 2148947 | 2151673 |  |  |  |  |  |  |  |  |  |  |  |  |  |  |
| JC13 | MALPY00517 | 3331 | xxx | 2150000 |  |  |  |  |  |  | 48 |  |  |  |  |  |  |  |  |
| 13 | MALPY01401 | 11348 | + | 2152857 | 2165148 |  |  |  |  |  | 48 | 33 |  |  |  |  |  |  |  |
| 13 | PcContig377 | 7614 | + | 2160781 | 2169055 |  |  |  |  |  |  |  |  |  |  |  |  |  |  |
| 13 | PbContig336 | 6609 | - | 2162569 | 2169743 |  |  |  |  |  |  |  |  |  |  |  |  |  |  |
| 13 | MALPY00809 | 4013 | + | 2165315 | 2169446 |  |  |  |  |  | 48 |  |  |  |  |  |  |  |  |
| 13 | Pc.c001103979.Contig1 | 648 | - | 2166747 | 2167332 |  |  |  |  |  |  |  |  |  |  |  |  |  |  |
| 13 | MALPY02690 | 2956 | + | 2168401 | 2171357 |  |  |  |  |  | 48 |  |  |  |  |  |  |  |  |
| 13 | Pb.c001904410.Contig1 | 1441 | - | 2169045 | 2170486 |  |  |  |  |  |  |  |  |  |  |  |  |  |  |
| 13 | Pb.c000301140.Contig1 | 7677 | - | 2170829 | 2180460 |  |  |  |  |  |  |  |  |  |  |  |  |  |  |
| 13 | MALPY00366 | 14688 | - | 2171474 | 2188133 |  |  |  |  |  | 48 | 320 | 1400 |  |  |  |  |  |  |
| 13 | Pc.c001505744.Contig1 | 1262 | - | 2172722 | 2174332 |  |  |  |  |  |  |  |  |  |  |  |  |  |  |
| 13 | Pc.c001103938.Contig1 | 5545 | - | 2174874 | 2181701 |  |  |  |  |  |  |  |  |  |  |  |  |  |  |
| 13 | Pb.c001201681.Contig2 | 3404 | - | 2180694 | 2184083 |  |  |  |  |  |  |  |  |  |  |  |  |  |  |
| 13 | Pc.c001604994.Contig2 | 4230 | - | 2185305 | 2189532 |  |  |  |  |  |  |  |  |  |  |  |  |  |  |
| 13 | MALPY03525 | 632 | - | 2189244 | 2189894 |  |  |  |  |  |  | 320 | 1300 |  |  |  |  |  |  |
| 13 | Pc.c000402791.Contig1 | 7092 | + | 2189761 | 2197251 |  |  |  |  |  |  |  |  |  |  |  |  |  |  |
| 13 | MALPY00877 | 6107 | - | 2190499 | 2196937 |  |  |  |  |  |  | 320 |  |  |  |  |  |  |  |
| 13 | Pb.c001802834.Contig1 | 5889 | - | 2191166 | 2197443 |  |  |  |  |  |  |  |  |  |  |  |  |  |  |
| 13 | MALPY03391 | 606 | + | 2191902 | 2192556 |  |  |  |  |  |  | 272 | 600 |  |  |  |  |  |  |
| JC13 | MALPY03392 | 444 | - | 2192591 | 2193035 | cRMPc13104 | 54551 | 82 | 2142892 | 2197443 |  | 272 | 4300 |  |  |  |  |  |  |
| 13 | MALPY02166 | 2712 | + | 2197525 | 2200240 |  |  |  |  |  |  | 272 | 3000 |  |  |  |  |  |  |
| 13 | Pc.c000803348.Contig1 | 1351 | - | 2197794 | 2199145 |  |  |  |  |  |  |  |  |  |  |  |  |  |  |
| 13 | Pb.c001904466.Contig1 | 2279 | - | 2198138 | 2200417 |  |  |  |  |  |  |  |  |  |  |  |  |  |  |
| 13 | PcContig430 | 7063 | - | 2199720 | 2206783 |  |  |  |  |  |  |  |  |  |  |  |  |  |  |
| 13 | PbContig263 | 4518 | + | 2201835 | 2206353 |  |  |  |  |  |  |  |  |  |  |  |  |  |  |
| 13 | MALPY00090 | 4823 | - | 2202411 | 2207234 | cRMPc13105 | 9709 | 784 | 2197525 | 2207234 |  | 272 | 1300 |  |  |  |  |  |  |
| 13 | Pb.c002005864.Contig1 | 3111 | + | 2208018 | 2211120 |  |  |  |  |  |  |  |  |  |  |  |  |  |  |
| 13 | MALPY00422 | 6520 | + | 2208410 | 2214735 |  |  |  |  |  |  | 272 - NS | 4500 |  |  |  |  |  |  |
| 13 | PcContig608 | 1376 | + | 2209835 | 2211214 |  |  |  |  |  |  |  |  |  |  |  |  |  |  |
| 13 | Pb.c000901801.Contig1 | 2581 | + | 2211153 | 2213731 |  |  |  |  |  |  |  |  |  |  |  |  |  |  |
| 13 | Pc.c000903274.Contig1 | 1981 | - | 2211175 | 2213150 | cRMPc13106 | 6717 | 3351 | 2208018 | 2214735 |  |  |  |  |  |  |  |  |  |
| 13 | PbContig44 | 14138 | - | 2218086 | 2232782 |  |  |  |  |  |  |  |  |  |  |  |  |  |  |
| 13 | Pc.c001504932.Contig1 | 6685 | + | 2218679 | 2225869 |  |  |  |  |  |  |  |  |  |  |  |  |  |  |
| 13 | MALPY00323 | 21386 | + | 2219235 | 2240922 |  |  |  |  |  | 272 - Ins | 272 - NS |  |  |  |  |  |  |  |
| 13 | Pc.c001505798.Contig1 | 1337 | + | 2226291 | 2227617 |  |  |  |  |  |  |  |  |  |  |  |  |  |  |
| 13 | MALPY03163 | 630 | - | 2226619 | 2227248 |  |  |  |  |  |  | 200 | 2300 |  |  |  |  |  |  |
| 13 | MALPY03262 | 489 | - | 2226897 | 2227379 |  |  |  |  |  |  | 200 |  |  |  |  |  |  |  |
| 13 | Pc.c000102504.Contig1 | 2057 | - | 2227844 | 2229853 |  |  |  |  |  |  |  |  |  |  |  |  |  |  |
| 13 | Pc.c000603060.Contig1 | 2708 | - | 2229825 | 2232651 |  |  |  |  |  |  |  |  |  |  |  |  |  |  |
| 13 | Pb.c001402393.Contig1 | 944 | - | 2232696 | 2233640 |  |  |  |  |  |  |  |  |  |  |  |  |  |  |
| 13 | Pb.c001202286.Contig1 | 3395 | - | 2240094 | 2243378 |  |  |  |  |  |  |  |  |  |  |  |  |  |  |
| 13 | MALPY01756 | 10878 | - | 2241422 | 2252669 |  |  |  |  |  | 272 - Ins |  |  |  |  |  |  |  |  |
| 13 | Pb.c001502033.Contig2 | 19790 | - | 2246281 | 2268075 |  |  |  |  |  |  |  |  |  |  |  |  |  |  |
| 13 | Pc.c001204046.Contig1 | 1047 | - | 2250349 | 2251393 |  |  |  |  |  |  |  |  |  |  |  |  |  |  |
| 13 | MALPY03104 | 718 | - | 2250520 | 2251256 |  |  |  |  |  |  | 311 | 4300 |  |  |  |  |  |  |
| 13 | Pc.c001708607.Contig1 | 621 | - | 2252172 | 2252790 |  |  |  |  |  |  |  |  |  |  |  |  |  |  |
| 13 | MALPY00834 | 37332 | - | 2257047 | 2294439 |  |  |  |  |  |  | 311 |  |  |  |  |  |  |  |
| 13 | Pc.c001103866.Contig1 | 2442 | + | 2259411 | 2261895 |  |  |  |  |  |  |  |  |  |  |  |  |  |  |
| 13 | MALPY03384 | 256 | + | 2260091 | 2260347 |  |  |  |  |  |  | 333 | 6500 |  |  |  |  |  |  |
| 13 | Pc.c001304256.Contig1 | 1427 | - | 2262747 | 2264174 |  |  |  |  |  |  |  |  |  |  |  |  |  |  |
| 13 | Pc.c000002649.Contig1 | 3424 | - | 2265187 | 2268611 |  |  |  |  |  |  |  |  |  |  |  |  |  |  |
| 13 | MALPY03346 | 709 | + | 2266720 | 2267428 |  |  |  |  |  |  | 333 | 600 |  |  |  |  |  |  |
| 13 | MALPY03345 | 428 | - | 2267497 | 2267914 |  |  |  |  |  |  | 333 |  |  |  |  |  |  |  |
| 13 | Pb.c001702807.Contig1 | 1974 | - | 2268140 | 2270102 |  |  |  |  |  |  |  |  |  |  |  |  |  |  |
| 13 | Pb.c002112086.Contig1 | 6742 | - | 2269063 | 2275806 |  |  |  |  |  |  |  |  |  |  |  |  |  |  |
| 13 | MALPY02082 | 12628 | + | 2269543 | 2282625 |  |  |  |  |  |  | 77 | 1000 |  |  |  |  |  |  |
| 13 | Pc.c001003458.Contig1 | 3821 | - | 2270431 | 2274261 |  |  |  |  |  |  |  |  |  |  |  |  |  |  |
| 13 | Pc.c001505092.Contig1 | 1811 | - | 2274563 | 2276374 |  |  |  |  |  |  |  |  |  |  |  |  |  |  |
| 13 | Pb.c001803476.Contig1 | 5696 | + | 2276427 | 2282457 |  |  |  |  |  |  |  |  |  |  |  |  |  |  |
| 13 | PcContig189 | 6553 | - | 2279883 | 2286498 |  |  |  |  |  |  |  |  |  |  |  |  |  |  |
| 13 | MALPY00160 | 31093 | + | 2283905 | 2314221 |  |  |  |  |  |  | 77 |  |  |  |  |  |  |  |
| 13 | PbContig131 | 24994 | - | 2284688 | 2309636 |  |  |  |  |  |  |  |  |  |  |  |  |  |  |
| 13 | PcContig129 | 4886 | - | 2287591 | 2292699 |  |  |  |  |  |  |  |  |  |  |  |  |  |  |
| 13 | Pc.c001304359.Contig1 | 1243 | - | 2292867 | 2294110 |  |  |  |  |  |  |  |  |  |  |  |  |  |  |
| 13 | PcContig381 | 14690 | - | 2294413 | 2308645 |  |  |  |  |  |  |  |  |  |  |  |  |  |  |
| 13 | Pb.c000101447.Contig1 | 11711 | - | 2309702 | 2321768 |  |  |  |  |  |  |  |  |  |  |  |  |  |  |
| 13 | Pc.c001707715.Contig1 | 1483 | - | 2311874 | 2313342 |  |  |  |  |  |  |  |  |  |  |  |  |  |  |
| 13 | MALPY00689 | 18151 | + | 2312811 | 2330790 |  |  |  |  |  | 62 |  |  |  |  |  |  |  |  |
| 13 | Pc.c000603027.Contig1 | 1885 | - | 2313410 | 2315292 |  |  |  |  |  |  |  |  |  |  |  |  |  |  |
| 13 | Pc.c001707711.Contig1 | 3778 | + | 2318209 | 2322005 |  |  |  |  |  |  |  |  |  |  |  |  |  |  |
| 13 | Pb.c001904450.Contig1 | 2007 | - | 2321360 | 2323367 |  |  |  |  |  |  |  |  |  |  |  |  |  |  |
| 13 | Pb.c000001633.Contig1 | 3234 | - | 2323319 | 2326589 |  |  |  |  |  |  |  |  |  |  |  |  |  |  |
| 13 | Pb.c000001363.Contig1 | 4036 | - | 2326955 | 2330994 |  |  |  |  |  |  |  |  |  |  |  |  |  |  |
| 13 | PcContig151 | 4317 | - | 2327168 | 2331486 | cRMPc13107 | 113400 | 738 | 2218086 | 2331486 |  |  |  |  |  |  |  |  |  |
| 13 | MALPY00350 | 24621 | - | 2332224 | 2359530 |  |  |  |  |  | 62 |  |  |  |  |  |  |  |  |
| 13 | PbContig158 | 18306 | + | 2332280 | 2353169 |  |  |  |  |  |  |  |  |  |  |  |  |  |  |
| 13 | Pc.c001605983.Contig1 | 2768 | - | 2332954 | 2335722 |  |  |  |  |  |  |  |  |  |  |  |  |  |  |
| 13 | Pc.c000202151.Contig1 | 5673 | + | 2336846 | 2343238 |  |  |  |  |  |  |  |  |  |  |  |  |  |  |
| 13 | MALPY03150 | 1018 | - | 2341302 | 2342320 |  |  |  |  |  |  |  |  |  |  |  |  |  |  |
| 13 | Pc.c001003455.Contig1 | 1985 | + | 2343297 | 2345405 |  |  |  |  |  |  |  |  |  |  |  |  |  |  |
| 13 | PcContig720 | 3862 | + | 2345761 | 2350124 |  |  |  |  |  |  |  |  |  |  |  |  |  |  |
| 13 | PbContig388 | 12107 | + | 2353323 | 2365924 |  |  |  |  |  |  |  |  |  |  |  |  |  |  |
| 13 | PcContig359 | 6692 | + | 2358672 | 2365379 |  |  |  |  |  |  |  |  |  |  |  |  |  |  |
| 13 | MALPY01260 | 5697 | - | 2359551 | 2365254 |  |  |  |  |  | 62 |  |  |  |  |  |  |  |  |
| 13 | MALPY01336 | 3275 | - | 2363359 | 2366640 |  |  |  |  |  | 779 |  |  |  |  |  |  |  |  |
| 13 | Pc.c001203200.Contig2 | 1931 | + | 2365306 | 2367237 |  |  |  |  |  |  |  |  |  |  |  |  |  |  |
| man13 | Pb.c000301160.Contig1 - Ins | 13642 | + | 2365975 | 2379131 |  |  |  |  |  |  |  |  |  |  |  |  |  |  |
| 13 | MALPY00300 | 7684 | + | 2366406 | 2373383 |  |  |  |  |  | 779 |  |  |  |  |  |  |  |  |
| 13 | Pc.c001104247.Contig1 | 627 | + | 2367072 | 2367699 |  |  |  |  |  |  |  |  |  |  |  |  |  |  |
| 13 | Pc.c001405293.Contig1 | 617 | + | 2367077 | 2367694 |  |  |  |  |  |  |  |  |  |  |  |  |  |  |
| 13 | PcContig4 | 1191 | + | 2367615 | 2368741 |  |  |  |  |  |  |  |  |  |  |  |  |  |  |
| 13 | Pc.c000402744.Contig1 | 6590 | + | 2369747 | 2376646 |  |  |  |  |  |  |  |  |  |  |  |  |  |  |
| 13 | MALPY03178 | 1901 | - | 2373579 | 2375486 |  |  |  |  |  | 779 | 215B | 3500 |  |  |  |  |  |  |
| 13 | Pc.c001103909.Contig1 | 1952 | + | 2378826 | 2380924 |  |  |  |  |  |  |  |  |  |  |  |  |  |  |
| 13 | MALPY02767 | 3109 | - | 2378969 | 2382224 |  |  |  |  |  |  | 215B | 8000 |  |  |  |  |  |  |
| 13 | Pb.c001202068.Contig1 | 4099 | + | 2379482 | 2383725 | cRMPc13108 | 51501 | 1981 | 2332224 | 2383725 |  |  |  |  |  |  |  |  |  |
| 13 | PbContig195 | 5821 | + | 2385706 | 2391335 |  |  |  |  |  |  |  |  |  |  |  |  |  |  |
| 13 | MALPY02025 | 14916 | + | 2385720 | 2400324 |  |  |  |  |  | 215 | 215B |  |  |  |  |  |  |  |
| 13 | Pc.c001003875.Contig1 | 630 | - | 2386539 | 2387130 |  |  |  |  |  |  |  |  |  |  |  |  |  |  |
| 13 | Pc.c001003320.Contig1 | 2710 | - | 2387274 | 2389864 |  |  |  |  |  |  |  |  |  |  |  |  |  |  |
| 13 | Pb.c001904953.Contig1 | 604 | - | 2391299 | 2391897 |  |  |  |  |  |  |  |  |  |  |  |  |  |  |
| 13 | PcContig688 | 2984 | - | 2394986 | 2397955 |  |  |  |  |  |  |  |  |  |  |  |  |  |  |
| 13 | MALPY00725 | 12718 | + | 2399744 | 2412888 |  |  |  |  |  | 215 |  |  |  |  |  |  |  |  |
| 13 | Pb.c001904345.Contig1 | 2923 | + | 2402277 | 2405200 |  |  |  |  |  |  |  |  |  |  |  |  |  |  |
| 13 | Pc.c001504963.Contig1 | 2985 | - | 2402490 | 2405475 |  |  |  |  |  |  |  |  |  |  |  |  |  |  |
| 13 | Pb.c000201274.Contig2 | 4123 | + | 2405772 | 2409823 |  |  |  |  |  |  |  |  |  |  |  |  |  |  |
| 13 | Pc.c001003352.Contig1 | 1848 | - | 2406338 | 2408139 |  |  |  |  |  |  |  |  |  |  |  |  |  |  |
| 13 | Pc.c001003898.Contig1 | 1155 | - | 2408054 | 2409151 | cRMPc13109 | 27182 | 348 | 2385706 | 2412888 |  |  |  |  |  |  |  |  |  |
| 13 | MALPY02569 | 4850 | + | 2413236 | 2418200 |  |  |  |  |  | 215 |  |  |  |  |  |  |  |  |
| 13 | Pc.c001203882.Contig1 | 2716 | - | 2413304 | 2416023 | cRMPc13110 | 4964 | 2067 | 2413236 | 2418200 |  |  |  | cRMPs1332 | 275308 | 2067 | 2142892 | 2418200 |  |
| 13 | MALPY02145 | 4960 | - | 2420267 | 2425227 |  |  |  |  |  |  |  |  |  |  |  |  |  |  |
| 13 | Pc.c001003921.Contig1 | 628 | + | 2420402 | 2421030 | cRMPc13111 | 4960 | 2412 | 2420267 | 2425227 |  |  |  | cRMPs1333 | 4960 | 2412 | 2420267 | 2425227 |  |
| 13 | MALPY01608 | 8367 | - | 2427639 | 2435955 |  |  |  |  |  | 1079 |  |  |  |  |  |  |  |  |
| 13 | Pc.c001605967.Contig1 | 2150 | - | 2428109 | 2430259 |  |  |  |  |  |  |  |  |  |  |  |  |  |  |
| 13 | Pb.c000101436.Contig1 | 2780 | + | 2428211 | 2430991 |  |  |  |  |  |  |  |  |  |  |  |  |  |  |
| 13 | PcContig468 | 1019 | - | 2433080 | 2434150 |  |  |  |  |  |  |  |  |  |  |  |  |  |  |
| 13 | PcContig582 | 4856 | + | 2434184 | 2439657 |  |  |  |  |  |  |  |  |  |  |  |  |  |  |
| 13 | Pb.c000201608.Contig1 | 4651 | + | 2434333 | 2439318 |  |  |  |  |  |  |  |  |  |  |  |  |  |  |
| 13 | MALPY02150 | 3307 | - | 2436059 | 2439724 | cRMPc13112 | 12085 | 2228 | 2427639 | 2439724 | 1079 | 306 | 2500 |  |  |  |  |  |  |
| 13 | Pc.c001404033.Contig1 | 1901 | + | 2441952 | 2443847 |  |  |  |  |  |  |  |  |  |  |  |  |  |  |
| 13 | MALPY00813 | 12085 | + | 2442037 | 2453475 |  |  |  |  |  | 19 | 306 |  |  |  |  |  |  |  |
| 13 | Pc.c000002481.Contig1 | 6297 | - | 2444787 | 2451522 |  |  |  |  |  |  |  |  |  |  |  |  |  |  |
| 13 | Pb.c001101880.Contig1 | 3226 | + | 2448656 | 2451858 |  |  |  |  |  |  |  |  |  |  |  |  |  |  |
| 13 | PcContig42 | 4071 | - | 2452273 | 2456731 |  |  |  |  |  |  |  |  |  |  |  |  |  |  |
| 13 | MALPY01206 | 5855 | + | 2454262 | 2461424 |  |  |  |  |  | 19 |  |  |  |  |  |  |  |  |
| 13 | PcContig218 | 3404 | + | 2458613 | 2462223 |  |  |  |  |  |  |  |  |  |  |  |  |  |  |
| 13 | Pb.c001904292.Contig1 | 1637 | - | 2459089 | 2460786 |  |  |  |  |  |  |  |  |  |  |  |  |  |  |
| 13 | MALPY01207 | 3578 | - | 2461116 | 2464584 |  |  |  |  |  | 19 |  |  |  |  |  |  |  |  |
| 13 | Pb.c001602496.Contig1 | 1580 | - | 2461969 | 2463549 |  |  |  |  |  |  |  |  |  |  |  |  |  |  |
| 13 | Pc.c001708843.Contig1 | 617 | - | 2462173 | 2462790 |  |  |  |  |  |  |  |  |  |  |  |  |  |  |
| 13 | Pc.c001404793.Contig1 | 2042 | + | 2462479 | 2464524 |  |  |  |  |  |  |  |  |  |  |  |  |  |  |
| 13 | Pb.c002112189.Contig1 | 1499 | - | 2464270 | 2465775 |  |  |  |  |  |  |  |  |  |  |  |  |  |  |
| TK13 | MALPY02707 | 3255 | - | 2464459 | 2468862 | cRMPc13113 | 26910 | 161 | 2441952 | 2468862 | 19 |  |  |  |  |  |  |  |  |
| TK13 | MALPY02581 | 2028 | + | 2469023 | 2471211 | cRMPc13114 | 2188 | 977 | 2469023 | 2471211 | 19 |  |  |  |  |  |  |  |  |
| 13 | MALPY00035 | 13981 | + | 2472188 | 2485980 |  |  |  |  |  | 19 |  |  |  |  |  |  |  |  |
| 13 | Pc.c001708293.Contig1 | 1202 | - | 2474462 | 2475664 |  |  |  |  |  |  |  |  |  |  |  |  |  |  |
| 13 | Pb.c000601260.Contig1 | 5208 | - | 2474703 | 2480196 |  |  |  |  |  |  |  |  |  |  |  |  |  |  |
| 13 | Pc.c000202700.Contig1 | 2945 | - | 2476256 | 2479400 |  |  |  |  |  |  |  |  |  |  |  |  |  |  |
| 13 | MALPY03552 | 406 | + | 2479313 | 2479728 |  |  |  |  |  |  |  |  |  |  |  |  |  |  |
| 13 | Pc.c001813205.Contig1 | 652 | - | 2479749 | 2480401 |  |  |  |  |  |  |  |  |  |  |  |  |  |  |
| 13 | PcContig603 | 13806 | + | 2481284 | 2496552 |  |  |  |  |  |  |  |  |  |  |  |  |  |  |
| 13 | PbContig404 | 5472 | + | 2482625 | 2488500 |  |  |  |  |  |  |  |  |  |  |  |  |  |  |
| 13 | MALPY03280 | 823 | - | 2483393 | 2484216 |  |  |  |  |  |  |  |  |  |  |  |  |  |  |
| 13 | MALPY03503 | 862 | - | 2483556 | 2484415 |  |  |  |  |  |  |  |  |  |  |  |  |  |  |
| 13 | MALPY01803 | 5182 | + | 2486150 | 2491192 |  |  |  |  |  | 19 |  |  |  |  |  |  |  |  |
| 13 | Pb.c001803433.Contig1 | 2943 | + | 2492838 | 2495799 |  |  |  |  |  |  |  |  |  |  |  |  |  |  |
| 13 | MALPY02464 | 12846 | + | 2492933 | 2506793 | cRMPc13115 | 34605 | 1230 | 2472188 | 2506793 |  | 51 | 700 |  |  |  |  |  |  |
| 13 | MALPY00031 | 5649 | - | 2508023 | 2513675 |  |  |  |  |  | 127 | 51 | 8000 |  |  |  |  |  |  |
| 13 | Pb.c000501500.Contig1 | 2542 | - | 2511116 | 2513682 |  |  |  |  |  |  |  |  |  |  |  |  |  |  |
| man13 | PbContig4232 | 2623 | + | 2512573 | 2515196 |  |  |  |  |  |  |  |  |  |  |  |  |  |  |
| man13 | MALPY00032 | 3702 | + | 2515087 | 2518789 |  |  |  |  |  | 127 |  |  |  |  |  |  |  |  |
| man13 | PbContig3537 | 2266 | - | 2518319 | 2520585 |  |  |  |  |  |  |  |  |  |  |  |  |  |  |
| TK13 | MALPY02086 | 2551 | - | 2518660 | 2520982 |  |  |  |  |  | 127 |  |  |  |  |  |  |  |  |
| 13 | Pc.c001104355.Contig1 | 1540 | + | 2520360 | 2521900 |  |  |  |  |  |  |  |  |  |  |  |  |  |  |
| 13 | Pb.c001803914.Contig1 | 3369 | - | 2520558 | 2524032 |  |  |  |  |  |  |  |  |  |  |  |  |  |  |
| 13 | MALPY03012 | 1351 | + | 2521545 | 2523040 |  |  |  |  |  | 295 | 51 |  |  |  |  |  |  |  |
| 13 | Pc.c001813082.Contig1 | 575 | + | 2522488 | 2523063 |  |  |  |  |  |  |  |  |  |  |  |  |  |  |
| 13 | MALPY00251 | 11145 | + | 2522995 | 2535227 |  |  |  |  |  | 295 |  |  |  |  |  |  |  |  |
| 13 | Pb.c000501697.Contig1 | 650 | - | 2524455 | 2525093 |  |  |  |  |  |  |  |  |  |  |  |  |  |  |
| 13 | PbContig387 | 9785 | - | 2525867 | 2536022 |  |  |  |  |  |  |  |  |  |  |  |  |  |  |
| 13 | PcContig748 | 6012 | + | 2527605 | 2533950 |  |  |  |  |  |  |  |  |  |  |  |  |  |  |
| 13 | MALPY03580 | 748 | - | 2533802 | 2534499 | cRMPc13116 | 27999 | 427 | 2508023 | 2536022 |  | 91 | 2000 |  |  |  |  |  |  |
| 13 | MALPY01139 | 5650 | - | 2536449 | 2542171 |  |  |  |  |  | 295 | 91 | 2000 |  |  |  |  |  |  |
| 13 | Pb.c000501445.Contig1 | 6178 | - | 2536946 | 2543169 |  |  |  |  |  |  |  |  |  |  |  |  |  |  |
| 13 | Pc.c001304087.Contig1 | 2241 | - | 2537929 | 2540230 | cRMPc13117 | 6720 | 1584 | 2536449 | 2543169 |  |  |  |  |  |  |  |  |  |
| 13 | MALPY02575 | 7696 | + | 2544753 | 2553152 |  |  |  |  |  |  | 91 | 1300 |  |  |  |  |  |  |
| 13 | Pc.c000903497.Contig1 | 2245 | - | 2545371 | 2547616 |  |  |  |  |  |  |  |  |  |  |  |  |  |  |
| 13 | Pb.c000601571.Contig1 | 5107 | + | 2549636 | 2555040 |  |  |  |  |  |  |  |  |  |  |  |  |  |  |
| 13 | Pc.c000903893.Contig1 | 1225 | - | 2550894 | 2552134 |  |  |  |  |  |  |  |  |  |  |  |  |  |  |
| 13 | Pc.c001708557.Contig1 | 1275 | - | 2552670 | 2554143 |  |  |  |  |  |  |  |  |  |  |  |  |  |  |
| 13 | MALPY02532 | 3745 | - | 2553507 | 2557239 |  |  |  |  |  |  | 91 | 900 |  |  |  |  |  |  |
| 13 | PcContig491 | 1722 | + | 2555396 | 2557154 | cRMPc13118 | 12486 | 198 | 2544753 | 2557239 |  |  |  |  |  |  |  |  |  |
| 13 | Pc.c001604982.Contig1 | 2544 | - | 2557437 | 2559981 |  |  |  |  |  |  |  |  |  |  |  |  |  |  |
| 13 | MALPY01388 | 2367 | + | 2557960 | 2560327 | cRMPc13119 | 2890 | 2549 | 2557437 | 2560327 | 503A | 91 - Ins | 6000 |  |  |  |  |  |  |
| 13 | Pb.c001202015.Contig1 | 2719 | + | 2562876 | 2565781 | cRMPc13120 | 2905 | 546 | 2562876 | 2565781 |  |  |  |  |  |  |  |  |  |
| 13 | MALPY02092 | 3908 | + | 2566327 | 2569966 |  |  |  |  |  |  | 91 - Ins |  |  |  |  |  |  |  |
| 13 | Pb.c001201987.Contig1 | 4657 | - | 2566412 | 2571154 |  |  |  |  |  |  |  |  |  |  |  |  |  |  |
| 13 | Pc.c001003314.Contig1 | 1192 | - | 2568248 | 2569401 |  |  |  |  |  |  |  |  |  |  |  |  |  |  |
| 13 | Pc.c001003955.Contig1 | 1233 | - | 2569680 | 2570913 | cRMPc13121 | 4827 | 2044 | 2566327 | 2571154 |  |  |  | cRMPs1334 | 143515 | 2044 | 2427639 | 2571154 |  |
| 13 | Pc.c000303068.Contig1 | 4165 | - | 2573198 | 2577732 |  |  |  |  |  |  |  |  |  |  |  |  |  |  |
| 13 | MALPY00315 | 7974 | - | 2573442 | 2582127 |  |  |  |  |  |  | 136 | 1000 |  |  |  |  |  |  |
| 13 | PbContig135 | 6229 | - | 2574965 | 2581856 |  |  |  |  |  |  |  |  |  |  |  |  |  |  |
| 13 | Pc.c001304786.Contig1 | 919 | - | 2577860 | 2578941 | cRMPc13122 | 8929 | 470 | 2573198 | 2582127 |  |  |  |  |  |  |  |  |  |
| 13 | Pc.c000702649.Contig1 | 3593 | - | 2582597 | 2586194 |  |  |  |  |  |  |  |  |  |  |  |  |  |  |
| 13 | MALPY03064 | 1613 | - | 2583174 | 2584760 |  |  |  |  |  | 746 | 136 |  |  |  |  |  |  |  |
| 13 | MALPY00758 | 2866 | - | 2583929 | 2586710 |  |  |  |  |  | 746 |  |  |  |  |  |  |  |  |
| 13 | Pb.c001803509.Contig1 | 1186 | + | 2585081 | 2586249 |  |  |  |  |  |  |  |  |  |  |  |  |  |  |
| man13 | PcContig6548 | 5278 | + | 2586204 | 2591482 |  |  |  |  |  |  |  |  |  |  |  |  |  |  |
| man13 | PbContig4633 | 6079 | + | 2588269 | 2594348 |  |  |  |  |  |  |  |  |  |  |  |  |  |  |
| man13 | MALPY02646 | 4494 | + | 2589031 | 2593525 | cRMPc13123 | 11751 |  | 2582597 | 2594348 |  |  |  | cRMPs1335 | 21150 |  | 2573198 | 2594348 |  |
| Telomere | |  |  |  |  |  |  |  |  |  |  |  |  |  |  |  |  |  |  |
|  |  | Pb Pc Py contig |  |  |  |  | cRMP contig | Gap to next cRMP contig |  |  |  |  |  |  | cRMP scaffold | Gap to next cRMP scaffold |  |  |  |
|  | Number | 1042 |  |  |  | Number | 123 | 122 |  |  |  |  |  | Number | 35 | 34 |  |  |  |
|  | Average size (bp) | 4611 |  |  |  | Average size (bp) | 19008 | 2101 |  |  |  |  |  | Average size (bp) | 70383 | 3851 |  |  |  |
|  | Minimum size (bp) | 112 |  |  |  | Minimum size (bp) | 701 | 26 |  |  |  |  |  | Minimum size (bp) | 1878 | 85 |  |  |  |
|  | Maximum size (bp) | 37332 |  |  |  | Maximum size (bp) | 113400 | 17060 |  |  |  |  |  | Maximum size (bp) | 275308 | 17060 |  |  |  |
|  | Total size (bp) | 4804380 |  |  |  | Total size (bp) | 2338029 | 256319 |  |  |  |  |  | Total size (bp) | 2463418 | 130930 |  |  |  |
|  |  |  |  |  |  |  |  |  |  |  |  |  |  |  |  |  |  |  |  |
