# Supplementary material for: A Plasmodium Whole-Genome Synteny Map: Indels and Synteny Breakpoints as Foci for Species-Specific Genes
Source: PLoS Pathog. 2005 Dec 23;1(4):e44. doi: 10.1371/journal.ppat.0010044 (PMC1317653; doi:10.1371/journal.ppat.0010044)
Supplement: Supplementary file 14 [file ppat.0010044.st030.doc]

Table S30 - RMPchr14


# Table S30 - RMPchr14

|  |  |  |  |  |  |  |  |  |  |  |  |  |  |  |  |  |  |  |  |
| --- | --- | --- | --- | --- | --- | --- | --- | --- | --- | --- | --- | --- | --- | --- | --- | --- | --- | --- | --- |
| Table S30: Tiling path of RMP contigs combined to form cRMP chromosome 14. | | | | | | |  |  |  |  |  |  |  |  |  |  |  |  |  |
|  |  |  |  |  |  |  |  |  |  |  |  |  |  |  |  |  |  |  |  |
| Tiling paths of P. berghei (Pb), P. c. chabaudi (Pc) and P. y. yoelii (Py) contigs combined into composite rodent malaria parasite (cRMP) | | | | | | | | | |  |  |  |  |  |  |  |  |  |  |
| contigs aligned against parts of the 14 chromosomes of P. falciparum (Pf). The cRMP contigs are further linked by physical linkage | | | | | | | | | |  |  |  |  |  |  |  |  |  |  |
| and PCR (see Methods) to form cRMP scaffolds. Different synteny blocks with homology to different P. falciparum chromosomes | | | | | | | | | |  |  |  |  |  |  |  |  |  |  |
| together form the cRMP chromosomes. The tables also show gaps in the alignment (black lines), cRMP telomeres (white bars) and | | | | | | | | | |  |  |  |  |  |  |  |  |  |  |
| predicted centromeres (plum/black bars). Contigs, physical groups or PCR groups interrupted by inter- or intrasyntenic regions are | | | | | | | | | |  |  |  |  |  |  |  |  |  |  |
| printed in bold. A summary of the number of RMP contigs, cRMP contigs and cRMP scaffolds as well as their average, minimum, | | | | | | | | | |  |  |  |  |  |  |  |  |  |  |
| maximum and total sizes is provided. Column headings are as follows: | | | | |  |  |  |  |  |  |  |  |  |  |  |  |  |  |  |
|  |  |  |  |  |  |  |  |  |  |  |  |  |  |  |  |  |  |  |  |
| Pf chr.: P. falciparum chromosome to which the RMP contigs are aligned | | | | | |  |  |  |  |  |  |  |  |  |  |  |  |  |  |
| (#: PROmer analysis� from this study; JC# and TK#: Carlton et al.; man#: manual BLAST analysis; | | | | | | |  |  |  |  |  |  |  |  |  |  |  |  |  |
| i: inversed orientation of the synteny block; colours refer to those used in Fig. 2 of the paper); | | | | | | |  |  |  |  |  |  |  |  |  |  |  |  |  |
| Pb Pc Py contig: names of the aligned RMP contigs; | | |  |  |  |  |  |  |  |  |  |  |  |  |  |  |  |  |  |
| Size: size (bp) of the aligned RMP contigs; | |  |  |  |  |  |  |  |  |  |  |  |  |  |  |  |  |  |  |
| +/-: orientation of the aligned RMP contigs; | |  |  |  |  |  |  |  |  |  |  |  |  |  |  |  |  |  |  |
| Start contig & End contig: estimated start & end coordinates of alignment of the RMP contigs based on the coordinates | | | | | | | | |  |  |  |  |  |  |  |  |  |  |  |
| of PROmer hits corrected for the contig�s size and orientation and presence of inter- and intrasyntenic regions | | | | | | | |  |  |  |  |  |  |  |  |  |  |  |  |
| (the start position of the first RMP contig in the alignment was set at zero); | | | | | |  |  |  |  |  |  |  |  |  |  |  |  |  |  |
| cRMP contig: names of cRMP contigs | |  |  |  |  |  |  |  |  |  |  |  |  |  |  |  |  |  |  |
| (the first 2 digits refer to the cRMP chromosome number and the following 3 digits designate the order of the contigs); | | | | | | | | |  |  |  |  |  |  |  |  |  |  |  |
| cRMP contig size: size of cRMP contigs based on the corrected coordinates of alignment; | | | | | | |  |  |  |  |  |  |  |  |  |  |  |  |  |
| Gap size to next cRMP contig: size of gaps between neighbouring cRMP contigs; | | | | | | |  |  |  |  |  |  |  |  |  |  |  |  |  |
| Start cRMP contig & End cRMP contig: estimated start & end coordinates of alignment of the cRMP contigs; | | | | | | | |  |  |  |  |  |  |  |  |  |  |  |  |
| Phys. group (Py): physical linkage of neighbouring P. y. yoelii contigs; | | | | | |  |  |  |  |  |  |  |  |  |  |  |  |  |  |
| PCR group (Py): neighbouring P. y. yoelii contigs were linked previously or for this study by PCR; | | | | | | |  |  |  |  |  |  |  |  |  |  |  |  |  |
| PCR size (Py): size of the PCR product; | |  |  |  |  |  |  |  |  |  |  |  |  |  |  |  |  |  |  |
| cRMP scaffold: names of cRMP scaffolds | |  |  |  |  |  |  |  |  |  |  |  |  |  |  |  |  |  |  |
| (the first 2 digits refer to the cRMP chromosome number and the following 2 digits designate the order of the scaffolds); | | | | | | | | |  |  |  |  |  |  |  |  |  |  |  |
| cRMP scaffold size: size of cRMP scaffolds; | | |  |  |  |  |  |  |  |  |  |  |  |  |  |  |  |  |  |
| Gap size to next cRMP scaffold: size of gaps between neighbouring cRMP scaffolds; | | | | | | |  |  |  |  |  |  |  |  |  |  |  |  |  |
| Start cRMP scaffold & End cRMP scaffold: estimated start & end coordinates of alignment of the cRMP scaffolds; | | | | | | | | |  |  |  |  |  |  |  |  |  |  |  |
| Overlap: Linkage between RMP contigs established through manual BLAST analysis. | | | | | | |  |  |  |  |  |  |  |  |  |  |  |  |  |
|  |  |  |  |  |  |  |  |  |  |  |  |  |  |  |  |  |  |  |  |
| Pf chr. | Pb Pc Py contig | Size | +/- | Start contig | End contig | cRMP contig | cRMP contig size | Gap size to next cRMP contig | Start cRMP contig | End cRMP contig | Phys. group (Py) | PCR group (Py) | PCR size (Py) | cRMP scaffold | cRMP scaffold size | Gap size to next cRMP scaffold | Start cRMP scaffold | End cRMP scaffold | Overlap (manual) |
| Telomere | |  |  |  |  |  |  |  |  |  |  |  |  |  |  |  |  |  |  |
| man13 | PbContig5483 | 8495 | - | 0 | 8495 |  |  |  |  |  |  |  |  |  |  |  |  |  | \| |
| man13 | MALPY00829 | 4878 | - | 159 | 5037 |  |  |  |  |  |  |  |  |  |  |  |  |  | \| |
| man13 | PcContig6115 | 3841 | + | 5377 | 9218 |  |  |  |  |  |  |  |  |  |  |  |  |  | \| |
| man13 | PcContig7036 | 5919 | + | 7723 | 13642 |  |  |  |  |  |  |  |  |  |  |  |  |  | \| |
| man13 | MALPY00728 | 3087 | + | 10426 | 13513 |  |  |  |  |  |  |  |  |  |  |  |  |  | \| |
| man13 | PbContig4527 | 2736 | - | 11185 | 13921 |  |  |  |  |  |  |  |  |  |  |  |  |  | \| |
| man13 | MALPY00853 | 2510 | + | 13627 | 16137 |  |  |  |  |  |  |  |  |  |  |  |  |  | \| |
| man13 | PcContig5539 | 3391 | + | 13702 | 17093 |  |  |  |  |  |  |  |  |  |  |  |  |  | \| |
| man13 | PbContig3966 | 2931 | + | 16298 | 19229 |  |  |  |  |  |  |  |  |  |  |  |  |  | MALPY00800 |
| 13 | MALPY00800 | 11330 | + | 16602 | 28275 |  |  |  |  |  | 1115 |  |  |  |  |  |  |  |  |
| 13 | Pb.c002007591.Contig1 | 812 | - | 19339 | 20283 |  |  |  |  |  |  |  |  |  |  |  |  |  |  |
| 13 | Pb.c001201986.Contig1 | 3544 | - | 20355 | 24022 |  |  |  |  |  |  |  |  |  |  |  |  |  |  |
| 13 | MALPY03057 | 302 | - | 20769 | 21071 |  |  |  |  |  |  |  |  |  |  |  |  |  |  |
| 13 | MALPY03446 | 518 | - | 20771 | 21289 |  |  |  |  |  |  | 245 | 5000 |  |  |  |  |  |  |
| 13 | Pb.c001001708.Contig1 | 4645 | - | 27803 | 32577 |  |  |  |  |  |  |  |  |  |  |  |  |  |  |
| 13 | MALPY00198 | 8529 | + | 28838 | 37735 |  |  |  |  |  | 1115 | 245 |  |  |  |  |  |  |  |
| 13 | Pc.c001204512.Contig1 | 877 | + | 30145 | 31181 |  |  |  |  |  |  |  |  |  |  |  |  |  |  |
| 13 | Pc.c001707744.Contig1 | 1351 | + | 31210 | 32561 |  |  |  |  |  |  |  |  |  |  |  |  |  |  |
| 13 | Pb.c001201608.Contig1 | 7000 | - | 33012 | 39821 |  |  |  |  |  |  |  |  |  |  |  |  |  |  |
| 13 | Pc.c000702693.Contig1 | 2371 | + | 33271 | 35561 |  |  |  |  |  |  |  |  |  |  |  |  |  |  |
| 13 | MALPY03457 | 248 | - | 34136 | 34384 |  |  |  |  |  |  | 253 | 2500 |  |  |  |  |  |  |
| 13 | MALPY01165 | 11528 | + | 37525 | 49289 |  |  |  |  |  | 1115 | 253 | 2000 |  |  |  |  |  |  |
| 13 | Pc.c001103916.Contig1 | 1211 | - | 41752 | 42987 |  |  |  |  |  |  |  |  |  |  |  |  |  |  |
| 13 | Pb.c001001816.Contig1 | 12979 | - | 42545 | 56908 |  |  |  |  |  |  |  |  |  |  |  |  |  |  |
| 13 | PcContig629 | 3252 | - | 43329 | 46579 |  |  |  |  |  |  |  |  |  |  |  |  |  |  |
| 13 | Pc.c001405417.Contig1 | 2170 | - | 46717 | 48796 |  |  |  |  |  |  |  |  |  |  |  |  |  |  |
| 13 | MALPY00828 | 10569 | + | 53046 | 63820 |  |  |  |  |  | 946 | 253 |  |  |  |  |  |  |  |
| 13 | Pb.c001402114.Contig1 | 6893 | - | 56575 | 64041 |  |  |  |  |  |  |  |  |  |  |  |  |  |  |
| 13 | PcContig182 | 5910 | + | 57706 | 64204 | cRMPc14001 | 64204 | 1012 | 0 | 64204 |  |  |  |  |  |  |  |  |  |
| 13 | MALPY00532 | 15922 | - | 65216 | 81138 | cRMPc14002 | 15922 | 1085 | 65216 | 81138 | 946 |  |  |  |  |  |  |  |  |
| TK13 | MALPY01102 | 4075 | + | 82223 | 86229 | cRMPc14003 | 4006 | 271 | 82223 | 86229 | 946 |  |  |  |  |  |  |  |  |
| TK13 | MALPY00128 | 6348 | + | 86500 | 93758 | cRMPc14004 | 7258 | 7177 | 86500 | 93758 | 946 |  |  | cRMPs1401 | 93758 | 7177 | 0 | 93758 |  |
| 13 | Pc.c001304240.Contig1 | 3479 | + | 100935 | 104384 |  |  |  |  |  |  |  |  |  |  |  |  |  |  |
| 13 | PbContig18 | 7274 | + | 101013 | 108123 |  |  |  |  |  |  |  |  |  |  |  |  |  |  |
| 13 | MALPY00408 | 15448 | - | 101629 | 117457 |  |  |  |  |  | 923 |  |  |  |  |  |  |  |  |
| 13 | Pc.c001103932.Contig1 | 3588 | + | 106740 | 110500 |  |  |  |  |  |  |  |  |  |  |  |  |  |  |
| 13 | PbContig107 | 4936 | - | 108653 | 113614 |  |  |  |  |  |  |  |  |  |  |  |  |  |  |
| 13 | MALPY03281 | 870 | + | 109910 | 110780 |  |  |  |  |  |  | 226 | 4300 |  |  |  |  |  |  |
| 13 | Pc.c001505104.Contig1 | 1518 | + | 110650 | 112177 |  |  |  |  |  |  |  |  |  |  |  |  |  |  |
| 13 | Pc.c001404905.Contig1 | 1778 | + | 112919 | 114712 |  |  |  |  |  |  |  |  |  |  |  |  |  |  |
| 13 | Pb.c000901774.Contig1 | 3246 | - | 114114 | 117288 |  |  |  |  |  |  |  |  |  |  |  |  |  |  |
| 13 | Pc.c001104234.Contig1 | 1528 | + | 114707 | 116151 | cRMPc14005 | 16522 | 938 | 100935 | 117457 |  |  |  |  |  |  |  |  |  |
| 13 | MALPY02657 | 4422 | - | 118395 | 122889 |  |  |  |  |  | 923 | 226 |  |  |  |  |  |  |  |
| 13 | Pc.c000803355.Contig1 | 1772 | + | 118727 | 120523 |  |  |  |  |  |  |  |  |  |  |  |  |  |  |
| 13 | Pb.c001201621.Contig1 | 6684 | - | 118852 | 125723 |  |  |  |  |  |  |  |  |  |  |  |  |  |  |
| 13 | MALPY01628 | 9055 | + | 119762 | 128826 |  |  |  |  |  | 443 |  |  |  |  |  |  |  |  |
| 13 | Pc.c001103836.Contig1 | 3065 | + | 121562 | 124624 |  |  |  |  |  |  |  |  |  |  |  |  |  |  |
| 13 | Pb.c000201238.Contig1 | 7503 | - | 124480 | 131819 |  |  |  |  |  |  |  |  |  |  |  |  |  |  |
| 13 | Pc.c000203294.Contig1 | 887 | - | 128050 | 128949 |  |  |  |  |  |  |  |  |  |  |  |  |  |  |
| 13 | MALPY00061 | 7250 | + | 128711 | 135933 |  |  |  |  |  | 443 | 282 | 1900 |  |  |  |  |  |  |
| 13 | Pc.c000102520.Contig1 | 2744 | - | 128912 | 131682 |  |  |  |  |  |  |  |  |  |  |  |  |  |  |
| 13 | Pb.c001101881.Contig1 | 6988 | - | 130616 | 138020 |  |  |  |  |  |  |  |  |  |  |  |  |  |  |
| 13 | MALPY00602 | 19420 | + | 131093 | 151466 |  |  |  |  |  |  | 282 |  |  |  |  |  |  |  |
| 13 | Pc.c000503027.Contig1 | 3839 | - | 132275 | 136252 |  |  |  |  |  |  |  |  |  |  |  |  |  |  |
| 13 | Pc.c001304360.Contig1 | 1360 | - | 136780 | 138443 |  |  |  |  |  |  |  |  |  |  |  |  |  |  |
| 13 | Pb.c001101827.Contig1 | 8066 | - | 138456 | 146476 |  |  |  |  |  |  |  |  |  |  |  |  |  |  |
| 13 | Pc.c001404779.Contig1 | 2590 | - | 138617 | 141175 |  |  |  |  |  |  |  |  |  |  |  |  |  |  |
| 13 | MALPY03368 | 519 | - | 146480 | 147014 |  |  |  |  |  |  | 338 | 8000 |  |  |  |  |  |  |
| 13 | Pb.c001301920.Contig1 | 4777 | + | 148708 | 153597 | cRMPc14006 | 35202 | 1105 | 118395 | 153597 |  |  |  |  |  |  |  |  |  |
| 13 | Pb.c001502061.Contig2 | 4949 | + | 154702 | 159729 |  |  |  |  |  |  |  |  |  |  |  |  |  |  |
| 13 | MALPY00155 | 11188 | + | 155029 | 166359 |  |  |  |  |  |  | 338 |  |  |  |  |  |  |  |
| 13 | PbContig224 | 7898 | + | 160708 | 168441 |  |  |  |  |  |  |  |  |  |  |  |  |  |  |
| 13 | Pc.c001505212.Contig1 | 629 | - | 161260 | 161874 |  |  |  |  |  |  |  |  |  |  |  |  |  |  |
| 13 | MALPY03583 | 664 | + | 164436 | 165055 |  |  |  |  |  |  |  |  |  |  |  |  |  |  |
| 13 | MALPY03098 | 1050 | + | 164482 | 165529 |  |  |  |  |  |  | 184 | 1700 |  |  |  |  |  |  |
| 13 | MALPY01797 | 3748 | - | 165729 | 169623 |  |  |  |  |  |  | 184 |  |  |  |  |  |  |  |
| 13 | Pc.c001003284.Contig1 | 2921 | - | 167240 | 170306 |  |  |  |  |  |  |  |  |  |  |  |  |  |  |
| 13 | Pb.c000401474.Contig1 | 1416 | + | 167820 | 169372 | cRMPc14007 | 15604 | 1017 | 154702 | 170306 |  |  |  | cRMPs1402 | 69371 | 1017 | 100935 | 170306 |  |
| 13 | MALPY01547 | 4945 | - | 171323 | 176265 |  |  |  |  |  | 319 |  |  |  |  |  |  |  |  |
| 13 | Pb.c000701264.Contig1 | 8126 | + | 175702 | 183770 |  |  |  |  |  |  |  |  |  |  |  |  |  |  |
| 13 | Pc.c001606426.Contig1 | 955 | + | 175833 | 176788 |  |  |  |  |  |  |  |  |  |  |  |  |  |  |
| 13 | MALPY00713 | 22430 | + | 176419 | 208211 |  |  |  |  |  | 319 |  |  |  |  |  |  |  |  |
| 13 | Pc.c001104330.Contig1 | 1162 | + | 177035 | 178200 |  |  |  |  |  |  |  |  |  |  |  |  |  |  |
| 13 | Pc.c001003440.Contig1 | 3204 | - | 185118 | 188319 |  |  |  |  |  |  |  |  |  |  |  |  |  |  |
| 13 | PbContig211 | 7362 | + | 197142 | 204939 |  |  |  |  |  |  |  |  |  |  |  |  |  |  |
| 13 | Pc.c000803921.Contig1 | 986 | + | 198716 | 199699 |  |  |  |  |  |  |  |  |  |  |  |  |  |  |
| 13 | PcContig667 | 2591 | + | 200114 | 202954 |  |  |  |  |  |  |  |  |  |  |  |  |  |  |
| 13 | Pc.c001304302.Contig1 | 1097 | + | 202934 | 204031 |  |  |  |  |  |  |  |  |  |  |  |  |  |  |
| 13 | Pb.c001201929.Contig1 | 8989 | + | 204292 | 213901 |  |  |  |  |  |  |  |  |  |  |  |  |  |  |
| 13 | MALPY01805 | 10809 | - | 207315 | 219596 |  |  |  |  |  | 319 |  |  |  |  |  |  |  |  |
| 13 | Pc.c000101989.Contig1 | 3637 | + | 208934 | 213128 |  |  |  |  |  |  |  |  |  |  |  |  |  |  |
| 13 | Pc.c001606813.Contig1 | 662 | + | 213428 | 214090 |  |  |  |  |  |  |  |  |  |  |  |  |  |  |
| 13 | Pc.c000903460.Contig1 | 2146 | + | 214071 | 216221 |  |  |  |  |  |  |  |  |  |  |  |  |  |  |
| 13 | PbContig187 | 18565 | - | 214423 | 234425 |  |  |  |  |  |  |  |  |  |  |  |  |  |  |
| 13 | MALPY00007 | 22849 | - | 218842 | 242788 |  |  |  |  |  | 319 | 35 | 1300 |  |  |  |  |  |  |
| 13 | Pc.c001505592.Contig1 | 687 | - | 223134 | 223821 |  |  |  |  |  |  |  |  |  |  |  |  |  |  |
| 13 | PcContig304 | 1351 | + | 226238 | 227589 |  |  |  |  |  |  |  |  |  |  |  |  |  |  |
| 13 | Pc.c000602963.Contig1 | 2813 | + | 227541 | 230210 |  |  |  |  |  |  |  |  |  |  |  |  |  |  |
| 13 | PcContig487 | 3294 | + | 231937 | 235285 |  |  |  |  |  |  |  |  |  |  |  |  |  |  |
| 13 | Pb.c000001774.Contig1 | 2962 | - | 235039 | 238058 |  |  |  |  |  |  |  |  |  |  |  |  |  |  |
| 13 | Pc.c001103811.Contig1 | 2143 | + | 238234 | 240362 |  |  |  |  |  |  |  |  |  |  |  |  |  |  |
| 13 | Pb.c000001797.Contig1 | 4379 | + | 238325 | 242689 | cRMPc14008 | 71465 | 331 | 171323 | 242788 |  |  |  |  |  |  |  |  |  |
| 13 | Pb.c000901869.Contig1 | 3130 | + | 243119 | 246591 |  |  |  |  |  |  |  |  |  |  |  |  |  |  |
| 13 | MALPY02099 | 9188 | + | 243506 | 252698 |  |  |  |  |  |  | 35 |  |  |  |  |  |  |  |
| 13 | Pc.c001708878.Contig1 | 746 | + | 244405 | 245151 |  |  |  |  |  |  |  |  |  |  |  |  |  |  |
| 13 | Pb.c001402189.Contig1 | 10166 | + | 246036 | 256613 |  |  |  |  |  |  |  |  |  |  |  |  |  |  |
| 13 | Pc.c000101998.Contig1 | 8153 | - | 247740 | 256402 |  |  |  |  |  |  |  |  |  |  |  |  |  |  |
| 13 | MALPY03162 | 220 | + | 249020 | 249240 |  |  |  |  |  |  | 161 | 1900 |  |  |  |  |  |  |
| 13 | MALPY01477 | 5601 | + | 250971 | 256857 | cRMPc14009 | 13738 | 966 | 243119 | 256857 |  | 161 | 500 |  |  |  |  |  |  |
| 13 | MALPY00893 | 17536 | + | 257823 | 276639 |  |  |  |  |  |  | 161 |  |  |  |  |  |  |  |
| 13 | Pb.c001602513.Contig1 | 17732 | + | 257882 | 276890 |  |  |  |  |  |  |  |  |  |  |  |  |  |  |
| 13 | MALPY03440 | 784 | + | 267899 | 268692 |  |  |  |  |  |  |  |  |  |  |  |  |  |  |
| 13 | Pc.c000803344.Contig1 | 1403 | + | 268213 | 269613 | cRMPc14010 | 19067 | 3307 | 257823 | 276890 |  |  |  | cRMPs1403 | 105567 | 3307 | 171323 | 276890 |  |
| 13 | MALPY01524 | 5032 | - | 280197 | 285302 |  |  |  |  |  |  |  |  |  |  |  |  |  |  |
| 13 | Pb.c001201959.Contig1 | 1510 | + | 280225 | 281753 |  |  |  |  |  |  |  |  |  |  |  |  |  |  |
| 13 | Pb.c001301891.Contig1 | 769 | + | 281732 | 282453 |  |  |  |  |  |  |  |  |  |  |  |  |  |  |
| 13 | Pb.c001803842.Contig1 | 1057 | + | 283516 | 284576 | cRMPc14011 | 5105 | 1597 | 280197 | 285302 |  |  |  | cRMPs1404 | 5105 | 1597 | 280197 | 285302 |  |
| 13 | MALPY01290 | 7944 | - | 286899 | 295434 |  |  |  |  |  | 1182 |  |  |  |  |  |  |  |  |
| 13 | Pb.c000901445.Contig1 | 6985 | - | 287160 | 294889 |  |  |  |  |  |  |  |  |  |  |  |  |  |  |
| 13 | Pc.c001103919.Contig1 | 2146 | - | 288008 | 290148 |  |  |  |  |  |  |  |  |  |  |  |  |  |  |
| 13 | Pc.c001103920.Contig1 | 1576 | - | 290814 | 292346 |  |  |  |  |  |  |  |  |  |  |  |  |  |  |
| 13 | Pc.c001304680.Contig1 | 651 | - | 292794 | 293445 |  |  |  |  |  |  |  |  |  |  |  |  |  |  |
| 13 | MALPY02663 | 5511 | - | 294908 | 300464 |  |  |  |  |  | 1182 | 203 | 1600 |  |  |  |  |  |  |
| 13 | Pb.c001904323.Contig1 | 4531 | - | 295630 | 300206 | cRMPc14012 | 13565 | 1452 | 286899 | 300464 |  |  |  |  |  |  |  |  |  |
| 13 | MALPY00472 | 3156 | - | 301916 | 305063 |  |  |  |  |  |  | 203 | 850 |  |  |  |  |  |  |
| 13 | MALPY02462 | 3219 | + | 304883 | 308247 |  |  |  |  |  |  | 203 | 1100 |  |  |  |  |  |  |
| 13 | Pb.c001502446.Contig1 | 2511 | - | 305020 | 307683 |  |  |  |  |  |  |  |  |  |  |  |  |  |  |
| 13 | Pc.c001304257.Contig1 | 1424 | - | 305384 | 306941 | cRMPc14013 | 6331 | 1070 | 301916 | 308247 |  |  |  |  |  |  |  |  |  |
| 13 | MALPY00638 | 14655 | + | 309317 | 324526 |  |  |  |  |  | 152 | 203 |  |  |  |  |  |  |  |
| 13 | Pc.c001104178.Contig1 | 5077 | + | 311604 | 316653 |  |  |  |  |  |  |  |  |  |  |  |  |  |  |
| 13 | Pb.c001602433.Contig1 | 5419 | - | 311986 | 317450 |  |  |  |  |  |  |  |  |  |  |  |  |  |  |
| 13 | PbContig110 | 10222 | - | 317420 | 328350 |  |  |  |  |  |  |  |  |  |  |  |  |  |  |
| 13 | Pc.c000302403.Contig2 | 2618 | - | 317688 | 320711 |  |  |  |  |  |  |  |  |  |  |  |  |  |  |
| 13 | Pb.c001301476.Contig1 | 742 | - | 319067 | 320184 |  |  |  |  |  |  |  |  |  |  |  |  |  |  |
| 13 | MALPY00883 | 13119 | + | 324604 | 338094 |  |  |  |  |  | 152 |  |  |  |  |  |  |  |  |
| 13 | Pc.c001204615.Contig1 | 1061 | - | 324882 | 325913 |  |  |  |  |  |  |  |  |  |  |  |  |  |  |
| 13 | Pc.c000103107.Contig1 | 1085 | - | 326143 | 327228 |  |  |  |  |  |  |  |  |  |  |  |  |  |  |
| 13 | Pb.c001502089.Contig1 | 4805 | - | 329486 | 334218 |  |  |  |  |  |  |  |  |  |  |  |  |  |  |
| 13 | Pc.c001103695.Contig1 | 1399 | - | 331296 | 332710 |  |  |  |  |  |  |  |  |  |  |  |  |  |  |
| 13 | Pc.c000502925.Contig1 | 3573 | - | 333359 | 336997 | cRMPc14014 | 28777 | 7192 | 309317 | 338094 |  |  |  | cRMPs1405 | 51195 | 7192 | 286899 | 338094 |  |
| 13 | PbContig397 | 11827 | + | 345286 | 356990 |  |  |  |  |  |  |  |  |  |  |  |  |  |  |
| 13 | MALPY00783 | 31838 | + | 351843 | 386523 |  |  |  |  |  |  | 123 | 800 |  |  |  |  |  |  |
| 13 | Pc.c001104492.Contig1 | 2245 | + | 352728 | 355080 |  |  |  |  |  |  |  |  |  |  |  |  |  |  |
| 13 | Pb.c000901447.Contig1 | 534 | - | 355094 | 355628 |  |  |  |  |  |  |  |  |  |  |  |  |  |  |
| 13 | Pc.c001405467.Contig1 | 923 | + | 355095 | 356012 |  |  |  |  |  |  |  |  |  |  |  |  |  |  |
| 13 | PbContig65 | 20951 | + | 357225 | 379257 |  |  |  |  |  |  |  |  |  |  |  |  |  |  |
| 13 | Pc.c001505829.Contig1 | 632 | + | 358722 | 359354 |  |  |  |  |  |  |  |  |  |  |  |  |  |  |
| 13 | Pc.c001505830.Contig1 | 1480 | + | 359434 | 361220 |  |  |  |  |  |  |  |  |  |  |  |  |  |  |
| 13 | Pc.c000302978.Contig1 | 989 | - | 362587 | 363585 |  |  |  |  |  |  |  |  |  |  |  |  |  |  |
| 13 | Pc.c000202235.Contig2 | 2709 | - | 363659 | 366318 |  |  |  |  |  |  |  |  |  |  |  |  |  |  |
| 13 | Pc.c000902729.Contig1 | 7902 | + | 368088 | 376461 |  |  |  |  |  |  |  |  |  |  |  |  |  |  |
| 13 | PbContig175 | 16170 | + | 381944 | 399976 |  |  |  |  |  |  |  |  |  |  |  |  |  |  |
| 13 | MALPY00415 | 9546 | - | 388329 | 398451 |  |  |  |  |  | 275 | 123 |  |  |  |  |  |  |  |
| 13 | PcContig90 | 5611 | - | 389376 | 395800 |  |  |  |  |  |  |  |  |  |  |  |  |  |  |
| 13 | MALPY03304 | 772 | + | 389377 | 390147 |  |  |  |  |  |  | 228 | 850 |  |  |  |  |  |  |
| 13 | MALPY03428 | 162 | + | 390434 | 390596 |  |  |  |  |  |  | 228 |  |  |  |  |  |  |  |
| 13 | MALPY03454 | 470 | - | 394776 | 395243 |  |  |  |  |  |  |  |  |  |  |  |  |  |  |
| 13 | MALPY03152 | 1825 | - | 394854 | 396668 |  |  |  |  |  |  | 321 | 2000 |  |  |  |  |  |  |
| 13 | Pc.c000503074.Contig1 | 3280 | - | 395942 | 398936 |  |  |  |  |  |  |  |  |  |  |  |  |  |  |
| 13 | MALPY01757 | 4469 | + | 398013 | 402482 |  |  |  |  |  | 275 | 321 |  |  |  |  |  |  |  |
| 13 | Pc.c001505112.Contig1 | 789 | - | 399357 | 400146 |  |  |  |  |  |  |  |  |  |  |  |  |  |  |
| 13 | Pc.c000703450.Contig1 | 1212 | - | 401968 | 403177 |  |  |  |  |  |  |  |  |  |  |  |  |  |  |
| 13 | Pb.c001904532.Contig1 | 2015 | + | 402164 | 404186 |  |  |  |  |  |  |  |  |  |  |  |  |  |  |
| 13 | MALPY00640 | 10400 | + | 402896 | 414106 |  |  |  |  |  | 275 |  |  |  |  |  |  |  |  |
| man13 | PbContig245 - Ins | 16015 | + | 405153 | 423243 |  |  |  |  |  |  |  |  |  |  |  |  |  |  |
| 13 | Pc.c001707696.Contig1 | 2374 | + | 407915 | 410388 |  |  |  |  |  |  |  |  |  |  |  |  |  |  |
| 13 | Pc.c001505118.Contig1 | 1684 | + | 410718 | 412438 |  |  |  |  |  |  |  |  |  |  |  |  |  |  |
| 13 | Pc.c000003135.Contig1 | 5110 | + | 413158 | 418998 |  |  |  |  |  |  |  |  |  |  |  |  |  |  |
| man13 | MALPY01596 - Ins | 9625 | - | 414673 | 425168 |  |  |  |  |  | 275 |  |  |  |  |  |  |  |  |
| 13 | MALPY03127 | 714 | + | 422813 | 423527 |  |  |  |  |  |  | 95 - Ins | 6000 |  |  |  |  |  |  |
| 13 | Pb.c001402102.Contig1 | 2294 | + | 423734 | 426082 | cRMPc14015 | 80796 | 2353 | 345286 | 426082 |  |  |  |  |  |  |  |  |  |
| 13 | Pb.c000001634.Contig1 | 7732 | - | 428435 | 435836 |  |  |  |  |  |  |  |  |  |  |  |  |  |  |
| 13 | MALPY02583 | 3796 | + | 429527 | 433317 |  |  |  |  |  |  | 95 - Ins | 1800 |  |  |  |  |  |  |
| 13 | MALPY03531 | 658 | + | 434015 | 434825 |  |  |  |  |  |  | 95 | 2000 |  |  |  |  |  |  |
| 13 | MALPY01113 | 20180 | - | 435772 | 455824 |  |  |  |  |  |  | 95 | 700 |  |  |  |  |  |  |
| 13 | Pc.c000403119.Contig1 | 3542 | - | 446348 | 449770 |  |  |  |  |  |  |  |  |  |  |  |  |  |  |
| 13 | Pb.c001402122.Contig1 | 2607 | - | 448947 | 451548 |  |  |  |  |  |  |  |  |  |  |  |  |  |  |
| 13 | Pb.c002113014.Contig1 | 1089 | - | 451659 | 452739 | cRMPc14016 | 27389 | 1056 | 428435 | 455824 |  |  |  |  |  |  |  |  |  |
| 13 | MALPY00794 | 14343 | - | 456880 | 471594 |  |  |  |  |  |  | 95 | 400 |  |  |  |  |  |  |
| 13 | Pb.c000101359.Contig1 | 2008 | - | 460105 | 462095 |  |  |  |  |  |  |  |  |  |  |  |  |  |  |
| 13 | Pb.c000301374.Contig1 | 3150 | - | 466353 | 469494 |  |  |  |  |  |  |  |  |  |  |  |  |  |  |
| 13 | Pb.c000501443.Contig1 | 2158 | - | 470351 | 472509 |  |  |  |  |  |  |  |  |  |  |  |  |  |  |
| JC13 | MALPY03450 | 501 | + | 471760 | 472261 |  |  |  |  |  |  | 95 | 1200 |  |  |  |  |  |  |
| 13 | MALPY00515 | 12308 | - | 472296 | 484014 |  |  |  |  |  |  | 95 | 1300 |  |  |  |  |  |  |
| 13 | Pb.c001202049.Contig1 | 4511 | - | 478950 | 483464 |  |  |  |  |  |  |  |  |  |  |  |  |  |  |
| 13 | Pc.c000602936.Contig1 | 3906 | + | 479849 | 483755 | cRMPc14017 | 27134 | 616 | 456880 | 484014 |  |  |  |  |  |  |  |  |  |
| 13 | MALPY01294 | 3383 | - | 484630 | 488187 |  |  |  |  |  |  | 95 |  |  |  |  |  |  |  |
| 13 | Pc.c001405360.Contig1 | 974 | + | 485195 | 486181 | cRMPc14018 | 3557 | 1130 | 484630 | 488187 |  |  |  | cRMPs1406 | 142901 | 1130 | 345286 | 488187 |  |
| 13 | Pc.c000903680.Contig1 | 4323 | - | 489317 | 493652 |  |  |  |  |  |  |  |  |  |  |  |  |  |  |
| 13 | MALPY00133 | 19590 | - | 491326 | 511062 |  |  |  |  |  | 434 |  |  |  |  |  |  |  |  |
| 13 | Pb.c000401531.Contig1 | 3302 | + | 493287 | 496574 |  |  |  |  |  |  |  |  |  |  |  |  |  |  |
| 13 | Pc.c000903934.Contig1 | 1172 | + | 495047 | 496213 |  |  |  |  |  |  |  |  |  |  |  |  |  |  |
| 13 | PcContig630 | 2673 | + | 495788 | 498724 |  |  |  |  |  |  |  |  |  |  |  |  |  |  |
| 13 | Pb.c002112260.Contig1 | 2589 | - | 497435 | 499790 |  |  |  |  |  |  |  |  |  |  |  |  |  |  |
| 13 | Pc.c000903995.Contig1 | 635 | + | 499083 | 499718 |  |  |  |  |  |  |  |  |  |  |  |  |  |  |
| 13 | Pb.c002112876.Contig1 | 957 | - | 500579 | 501554 |  |  |  |  |  |  |  |  |  |  |  |  |  |  |
| 13 | PcContig193 | 1461 | - | 503149 | 504610 |  |  |  |  |  |  |  |  |  |  |  |  |  |  |
| 13 | Pb.c001101852.Contig1 | 2699 | - | 503894 | 506595 |  |  |  |  |  |  |  |  |  |  |  |  |  |  |
| 13 | Pc.c000303030.Contig1 | 1568 | + | 504631 | 506223 |  |  |  |  |  |  |  |  |  |  |  |  |  |  |
| 13 | Pb.c001001981.Contig1 | 1078 | - | 506521 | 507599 |  |  |  |  |  |  |  |  |  |  |  |  |  |  |
| 13 | Pc.c000303491.Contig1 | 775 | - | 506612 | 507387 |  |  |  |  |  |  |  |  |  |  |  |  |  |  |
| 13 | Pc.c001103743.Contig1 | 605 | + | 506715 | 507320 |  |  |  |  |  |  |  |  |  |  |  |  |  |  |
| 13 | PcContig128 | 6347 | - | 507075 | 514307 |  |  |  |  |  |  |  |  |  |  |  |  |  |  |
| 13 | Pb.c000901727.Contig1 | 3913 | - | 510352 | 514399 |  |  |  |  |  |  |  |  |  |  |  |  |  |  |
| 13 | MALPY02437 | 3738 | + | 511439 | 515230 |  |  |  |  |  | 434 |  |  |  |  |  |  |  |  |
| 13 | Pc.c000602354.Contig1 | 3880 | - | 514969 | 518738 |  |  |  |  |  |  |  |  |  |  |  |  |  |  |
| 13 | MALPY01424 | 6116 | - | 515545 | 521418 |  |  |  |  |  | 434 |  |  |  |  |  |  |  |  |
| 13 | Pb.c001602088.Contig1 | 3804 | - | 516254 | 519991 |  |  |  |  |  |  |  |  |  |  |  |  |  |  |
| 13 | Pc.c000602879.Contig1 | 1600 | - | 518704 | 520439 |  |  |  |  |  |  |  |  |  |  |  |  |  |  |
| 13 | Pb.c001904842.Contig1 | 1251 | - | 520366 | 521524 | cRMPc14019 | 32207 | 123 | 489317 | 521524 |  |  |  |  |  |  |  |  |  |
| 13 | MALPY01410 | 9076 | + | 521647 | 530750 |  |  |  |  |  | 434 | 55 | 5000 |  |  |  |  |  |  |
| 13 | Pb.c000501203.Contig1 | 5567 | - | 522745 | 528451 |  |  |  |  |  |  |  |  |  |  |  |  |  |  |
| 13 | PcContig523 | 3902 | + | 523859 | 528080 |  |  |  |  |  |  |  |  |  |  |  |  |  |  |
| 13 | Pc.c000603283.Contig1 | 704 | - | 528265 | 528975 |  |  |  |  |  |  |  |  |  |  |  |  |  |  |
| 13 | Pc.c000602906.Contig1 | 4931 | - | 529265 | 534196 | cRMPc14020 | 12549 | 546 | 521647 | 534196 |  |  |  |  |  |  |  |  |  |
| 13 | PbContig383 | 6261 | - | 534742 | 540983 |  |  |  |  |  |  |  |  |  |  |  |  |  |  |
| 13 | Pc.c000102600.Contig1 | 4622 | - | 535259 | 539716 |  |  |  |  |  |  |  |  |  |  |  |  |  |  |
| 13 | MALPY02453 | 8980 | + | 536382 | 544609 |  |  |  |  |  |  | 55 | 1300 |  |  |  |  |  |  |
| 13 | PcContig626 | 4507 | - | 541164 | 545794 |  |  |  |  |  |  |  |  |  |  |  |  |  |  |
| 13 | Pb.c000501422.Contig1 | 4172 | + | 541942 | 546288 |  |  |  |  |  |  |  |  |  |  |  |  |  |  |
| 13 | MALPY02236 | 6905 | - | 545122 | 552227 |  |  |  |  |  | 112 | 55 |  |  |  |  |  |  |  |
| 13 | Pc.c001104485.Contig1 | 633 | + | 545952 | 546573 |  |  |  |  |  |  |  |  |  |  |  |  |  |  |
| 13 | Pc.c000402728.Contig1 | 3162 | - | 548452 | 551719 |  |  |  |  |  |  |  |  |  |  |  |  |  |  |
| 13 | Pb.c000801780.Contig1 | 5770 | - | 548640 | 555511 |  |  |  |  |  |  |  |  |  |  |  |  |  |  |
| 13 | MALPY02237 | 2604 | + | 552822 | 555777 |  |  |  |  |  | 112 | 89 | 3000 |  |  |  |  |  |  |
| 13 | Pc.c001813251.Contig1 | 685 | - | 553824 | 554512 | cRMPc14021 | 21035 | 376 | 534742 | 555777 |  |  |  |  |  |  |  |  |  |
| 13 | Pc.c001003794.Contig1 | 2581 | - | 556153 | 558722 |  |  |  |  |  |  |  |  |  |  |  |  |  |  |
| 13 | MALPY00925 | 10981 | - | 558468 | 569665 |  |  |  |  |  |  | 89 | 600 |  |  |  |  |  |  |
| 13 | Pb.c001301762.Contig1 | 2667 | + | 559563 | 562236 |  |  |  |  |  |  |  |  |  |  |  |  |  |  |
| 13 | PcContig790 | 6180 | + | 561365 | 567646 |  |  |  |  |  |  |  |  |  |  |  |  |  |  |
| 13 | Pb.c001001377.Contig2 | 4612 | + | 564862 | 569499 |  |  |  |  |  |  |  |  |  |  |  |  |  |  |
| 13 | Pc.c000703444.Contig1 | 4387 | - | 568997 | 573456 |  |  |  |  |  |  |  |  |  |  |  |  |  |  |
| 13 | PbContig165 | 9308 | - | 569712 | 579428 |  |  |  |  |  |  |  |  |  |  |  |  |  |  |
| 13 | MALPY00671 | 3828 | - | 570111 | 573932 |  |  |  |  |  | 126 | 89 |  |  |  |  |  |  |  |
| TK13 | MALPY00672 | 2695 | + | 574705 | 577007 |  |  |  |  |  | 126 |  |  |  |  |  |  |  |  |
| 13 | MALPY00981 | 20524 | + | 576306 | 599794 |  |  |  |  |  | 126 |  |  |  |  |  |  |  |  |
| 13 | PbContig316 | 10679 | - | 580012 | 595615 |  |  |  |  |  |  |  |  |  |  |  |  |  |  |
| 13 | PcContig423 | 4033 | + | 580116 | 584151 |  |  |  |  |  |  |  |  |  |  |  |  |  |  |
| 13 | PbContig80 | 4599 | + | 587500 | 592265 |  |  |  |  |  |  |  |  |  |  |  |  |  |  |
| 13 | Pc.c000103116.Contig1 | 996 | - | 588501 | 589497 |  |  |  |  |  |  |  |  |  |  |  |  |  |  |
| 13 | MALPY03220 | 1398 | - | 589051 | 590626 |  |  |  |  |  |  | 162 | 6000 |  |  |  |  |  |  |
| 13 | Pc.c000303076.Contig1 | 2095 | - | 590708 | 592830 |  |  |  |  |  |  |  |  |  |  |  |  |  |  |
| 13 | Pc.c000303482.Contig1 | 1183 | + | 592951 | 594134 |  |  |  |  |  |  |  |  |  |  |  |  |  |  |
| 13 | Pc.c001708233.Contig1 | 772 | + | 595319 | 596091 |  |  |  |  |  |  |  |  |  |  |  |  |  |  |
| 13 | MALPY02596 | 4824 | - | 598775 | 603593 |  |  |  |  |  | 41 | 162 |  |  |  |  |  |  |  |
| 13 | PbContig19 | 7056 | - | 599589 | 606645 |  |  |  |  |  |  |  |  |  |  |  |  |  |  |
| 13 | MALPY00052 | 19228 | - | 603608 | 622441 |  |  |  |  |  | 41 |  |  |  |  |  |  |  |  |
| 13 | PbContig352 | 7600 | + | 607515 | 615142 |  |  |  |  |  |  |  |  |  |  |  |  |  |  |
| 13 | PcContig725 | 4366 | + | 611147 | 615513 |  |  |  |  |  |  |  |  |  |  |  |  |  |  |
| 13 | PbContig254 | 6643 | + | 617439 | 624176 |  |  |  |  |  |  |  |  |  |  |  |  |  |  |
| 13 | Pc.c000503112.Contig1 | 3003 | + | 617909 | 621007 |  |  |  |  |  |  |  |  |  |  |  |  |  |  |
| 13 | MALPY03094 | 487 | + | 618472 | 618959 |  |  |  |  |  |  | 125 | 1000 |  |  |  |  |  |  |
| 13 | MALPY03499 | 702 | + | 620022 | 620727 |  |  |  |  |  |  | 125 | 3500 |  |  |  |  |  |  |
| 13 | PcContig183 | 5799 | - | 622708 | 628701 |  |  |  |  |  |  |  |  |  |  |  |  |  |  |
| 13 | MALPY01124 | 3964 | + | 623628 | 627359 |  |  |  |  |  |  | 125 | 800 |  |  |  |  |  |  |
| 13 | PbContig341 | 9504 | + | 624752 | 634174 |  |  |  |  |  |  |  |  |  |  |  |  |  |  |
| 13 | MALPY03487 | 801 | - | 627691 | 628477 |  |  |  |  |  |  | 125 | 400 |  |  |  |  |  |  |
| 13 | MALPY03200 | 520 | + | 628595 | 629115 |  |  |  |  |  |  | 125 | 5000 |  |  |  |  |  |  |
| TK13 | MALPY00541 | 6029 | + | 630920 | 636913 |  |  |  |  |  | 164 |  |  |  |  |  |  |  |  |
| 13 | MALPY00481 | 14720 | - | 636234 | 651130 |  |  |  |  |  | 164 | 125 |  |  |  |  |  |  |  |
| 13 | Pb.c000901724.Contig1 | 3588 | - | 636683 | 640294 |  |  |  |  |  |  |  |  |  |  |  |  |  |  |
| 13 | Pc.c000602362.Contig2 | 2696 | + | 639699 | 642368 |  |  |  |  |  |  |  |  |  |  |  |  |  |  |
| 13 | Pb.c000301492.Contig1 | 2810 | - | 640386 | 643196 |  |  |  |  |  |  |  |  |  |  |  |  |  |  |
| 13 | Pb.c000401175.Contig2 | 7533 | - | 643206 | 651049 |  |  |  |  |  |  |  |  |  |  |  |  |  |  |
| 13 | Pb.c001803357.Contig1 | 4452 | - | 650479 | 654941 |  |  |  |  |  |  |  |  |  |  |  |  |  |  |
| 13 | MALPY00934 | 16005 | + | 651136 | 669607 |  |  |  |  |  | 1113 |  |  |  |  |  |  |  |  |
| 13 | Pc.c001605876.Contig1 | 2947 | + | 651798 | 654758 |  |  |  |  |  |  |  |  |  |  |  |  |  |  |
| JC13 | MALPY03462 | 232 | + | 652504 | 652736 |  |  |  |  |  |  | 258 | 1600 |  |  |  |  |  |  |
| 13 | MALPY03356 | 493 | - | 654083 | 654579 |  |  |  |  |  |  | 258 |  |  |  |  |  |  |  |
| 13 | Pb.c001001690.Contig1 | 3022 | - | 656346 | 659386 |  |  |  |  |  |  |  |  |  |  |  |  |  |  |
| 13 | Pc.c001103883.Contig1 | 2720 | + | 657194 | 659932 |  |  |  |  |  |  |  |  |  |  |  |  |  |  |
| 13 | Pb.c000201573.Contig1 | 15199 | - | 661660 | 677196 |  |  |  |  |  |  |  |  |  |  |  |  |  |  |
| 13 | Pc.c000602867.Contig1 | 2977 | + | 669484 | 672386 |  |  |  |  |  |  |  |  |  |  |  |  |  |  |
| 13 | MALPY01378 | 5481 | - | 669709 | 675336 |  |  |  |  |  | 1113 |  |  |  |  |  |  |  |  |
| 13 | Pc.c001203846.Contig1 | 1729 | + | 672671 | 674397 |  |  |  |  |  |  |  |  |  |  |  |  |  |  |
| 13 | MALPY02744 | 3009 | - | 674207 | 677133 | cRMPc14022 | 121043 | 1144 | 556153 | 677196 | 1113 |  |  | cRMPs1407 | 187879 | 1144 | 489317 | 677196 |  |
| 13 | MALPY02108 | 7059 | - | 678340 | 685076 |  |  |  |  |  | 489 |  |  |  |  |  |  |  |  |
| 13 | Pb.c001502604.Contig1 | 1206 | - | 679217 | 680429 |  |  |  |  |  |  |  |  |  |  |  |  |  |  |
| 13 | PbContig156 | 8421 | - | 680659 | 688688 |  |  |  |  |  |  |  |  |  |  |  |  |  |  |
| 13 | MALPY00531 | 10671 | - | 685259 | 696347 |  |  |  |  |  | 489 | 291 - Ins | 1800 |  |  |  |  |  |  |
| 13 | Pb.c000601624.Contig1 | 5572 | - | 689020 | 695032 | cRMPc14023 | 18007 | 1332 | 678340 | 696347 |  |  |  |  |  |  |  |  |  |
| 13 | Pb.c000401447.Contig1 | 8730 | - | 697679 | 706769 |  |  |  |  |  |  |  |  |  |  |  |  |  |  |
| 13 | MALPY01154 | 10481 | - | 698147 | 708910 |  |  |  |  |  |  | 291 - Ins |  |  |  |  |  |  |  |
| 13 | Pc.c001404785.Contig1 | 1166 | - | 701731 | 702918 |  |  |  |  |  |  |  |  |  |  |  |  |  |  |
| 13 | MALPY03478 | 840 | - | 702018 | 702873 |  |  |  |  |  |  |  |  |  |  |  |  |  |  |
| 13 | Pc.c001003287.Contig1 | 2692 | - | 703083 | 705775 |  |  |  |  |  |  |  |  |  |  |  |  |  |  |
| 13 | Pb.c000802027.Contig1 | 742 | - | 707087 | 707814 | cRMPc14024 | 11231 | 36 | 697679 | 708910 |  |  |  | cRMPs1408 | 30570 | 36 | 678340 | 708910 |  |
| 13 | Pb.c000601528.Contig1 | 2836 | - | 708946 | 711523 |  |  |  |  |  |  |  |  |  |  |  |  |  |  |
| 13 | MALPY02555 | 10030 | + | 709025 | 719343 |  |  |  |  |  | 258 |  |  |  |  |  |  |  |  |
| 13 | Pc.c000703965.Contig1 | 1195 | + | 710867 | 712065 |  |  |  |  |  |  |  |  |  |  |  |  |  |  |
| 13 | PbContig180 | 15777 | - | 712342 | 728121 |  |  |  |  |  |  |  |  |  |  |  |  |  |  |
| 13 | Pc.c001404846.Contig1 | 978 | + | 716022 | 717000 |  |  |  |  |  |  |  |  |  |  |  |  |  |  |
| 13 | MALPY01021 | 8533 | - | 719487 | 727850 |  |  |  |  |  | 258 | 70 | 1600 |  |  |  |  |  |  |
| 13 | Pc.c000402740.Contig1 | 3811 | + | 722351 | 726127 |  |  |  |  |  |  |  |  |  |  |  |  |  |  |
| 13 | Pb.c002007802.Contig1 | 827 | - | 728069 | 728896 |  |  |  |  |  |  |  |  |  |  |  |  |  |  |
| 13 | MALPY00439 | 13255 | - | 728519 | 747003 |  |  |  |  |  |  | 70 | 1700 |  |  |  |  |  |  |
| 13 | Pc.c001405155.Contig1 | 642 | + | 728680 | 729322 |  |  |  |  |  |  |  |  |  |  |  |  |  |  |
| 13 | Pb.c001602805.Contig1 | 1398 | - | 744251 | 745649 | cRMPc14025 | 38057 | 1587 | 708946 | 747003 |  |  |  |  |  |  |  |  |  |
| 13 | MALPY03020 | 813 | - | 748590 | 749406 | cRMPc14026 | 816 | 108 | 748590 | 749406 |  | 70 | 400 |  |  |  |  |  |  |
| 13 | MALPY03459 | 742 | + | 749514 | 750253 |  |  |  |  |  |  | 70 | 300 |  |  |  |  |  |  |
| 13 | MALPY02127 | 2682 | - | 750236 | 753347 |  |  |  |  |  |  | 70 | 800 |  |  |  |  |  |  |
| 13 | Pc.c001103686.Contig1 | 2189 | + | 752814 | 755213 |  |  |  |  |  |  |  |  |  |  |  |  |  |  |
| 13 | MALPY01199 | 8930 | - | 753361 | 762744 |  |  |  |  |  | 918B | 70 | 3500 |  |  |  |  |  |  |
| 13 | Pb.c001301844.Contig1 | 3105 | + | 755832 | 758937 |  |  |  |  |  |  |  |  |  |  |  |  |  |  |
| 13 | Pc.c000503115.Contig1 | 1952 | - | 756769 | 758718 |  |  |  |  |  |  |  |  |  |  |  |  |  |  |
| 13 | Pb.c002112148.Contig1 | 1339 | + | 759445 | 760798 | cRMPc14027 | 13230 | 2512 | 749514 | 762744 |  |  |  |  |  |  |  |  |  |
| 13 | MALPY00506 - BP | 17146 | + | 765256 | 782039 |  |  |  |  |  |  | 70 |  |  |  |  |  |  |  |
| 13 | Pc.c000703451.Contig1 | 3985 | + | 767167 | 771152 |  |  |  |  |  |  |  |  |  |  |  |  |  |  |
| 13 | Pb.c000301434.Contig1 | 2405 | + | 767815 | 770220 |  |  |  |  |  |  |  |  |  |  |  |  |  |  |
| 7 | Pb.c000601591.Contig1 | 5212 | + | 770635 | 775452 |  |  |  |  |  |  |  |  |  |  |  |  |  |  |
| 7 | Pc.c000303415.Contig1 | 884 | + | 771166 | 772047 |  |  |  |  |  |  |  |  |  |  |  |  |  |  |
| 7 | Pc.c000002548.Contig1 | 2348 | - | 773292 | 775625 |  |  |  |  |  |  |  |  |  |  |  |  |  |  |
| 7 | Pb.c001802842.Contig3 | 6756 | + | 775683 | 782525 |  |  |  |  |  |  |  |  |  |  |  |  |  |  |
| 7 | MALPY03424 | 791 | + | 776679 | 777470 |  |  |  |  |  |  | 223 | 1300 |  |  |  |  |  |  |
| 7 | MALPY03504 | 750 | + | 778291 | 779047 |  |  |  |  |  |  | 223 |  |  |  |  |  |  |  |
| 7 | MALPY03190 | 497 | - | 778704 | 779201 |  |  |  |  |  |  |  |  |  |  |  |  |  |  |
| 7 | MALPY03526 | 417 | - | 778795 | 779209 |  |  |  |  |  |  |  |  |  |  |  |  |  |  |
| 7 | MALPY03436 | 821 | + | 779153 | 779983 | cRMPc14028 | 17269 | 23 | 765256 | 782525 |  | 238 | 2600 |  |  |  |  |  |  |
| 7 | MALPY02343 | 3019 | - | 782548 | 785570 |  |  |  |  |  |  | 238 | 700 |  |  |  |  |  |  |
| 7 | Pb.c001904499.Contig1 | 1538 | + | 783238 | 784779 | cRMPc14029 | 3022 | 2199 | 782548 | 785570 |  |  |  |  |  |  |  |  |  |
| 7 | MALPY01523 | 8346 | - | 787769 | 796451 |  |  |  |  |  |  | 238 |  |  |  |  |  |  |  |
| 7 | Pb.c001502517.Contig1 | 4850 | + | 789412 | 794214 |  |  |  |  |  |  |  |  |  |  |  |  |  |  |
| 7 | MALPY03317 | 976 | - | 791065 | 792015 |  |  |  |  |  |  | 16 | 4200 |  |  |  |  |  |  |
| 7 | Pc.c000803833.Contig1 | 1216 | + | 791206 | 792413 | cRMPc14030 | 8682 | 875 | 787769 | 796451 |  |  |  |  |  |  |  |  |  |
| 7 | PbContig328 | 7723 | + | 797326 | 805916 |  |  |  |  |  |  |  |  |  |  |  |  |  |  |
| 7 | MALPY00722 | 21289 | + | 798196 | 822662 |  |  |  |  |  |  | 16 |  |  |  |  |  |  |  |
| 7 | Pc.c001405438.Contig1 | 976 | + | 801496 | 802472 |  |  |  |  |  |  |  |  |  |  |  |  |  |  |
| 7 | Pc.c001813008.Contig1 | 590 | + | 805088 | 805678 |  |  |  |  |  |  |  |  |  |  |  |  |  |  |
| 7 | PcContig545 | 5264 | - | 814235 | 819713 |  |  |  |  |  |  |  |  |  |  |  |  |  |  |
| 7 | PbContig301 | 4557 | + | 814706 | 819484 |  |  |  |  |  |  |  |  |  |  |  |  |  |  |
| 7 | Pc.c001103859.Contig1 | 2022 | - | 820877 | 822896 |  |  |  |  |  |  |  |  |  |  |  |  |  |  |
| 7 | Pb.c000501521.Contig1 | 6299 | + | 821308 | 828868 |  |  |  |  |  |  |  |  |  |  |  |  |  |  |
| 7 | MALPY00342 | 13998 | - | 822938 | 838886 |  |  |  |  |  |  | 305 | 2000 |  |  |  |  |  |  |
| 7 | PcContig584 | 8234 | + | 823293 | 833340 |  |  |  |  |  |  |  |  |  |  |  |  |  |  |
| 7 | PbContig139 | 6544 | + | 828978 | 836233 | cRMPc14031 | 41560 | 3616 | 797326 | 838886 |  |  |  |  |  |  |  |  |  |
| 7 | Pc.c000503181.Contig1 | 3545 | - | 842502 | 846059 |  |  |  |  |  |  |  |  |  |  |  |  |  |  |
| 7 | Pb.c001803381.Contig1 | 2961 | - | 843054 | 845988 |  |  |  |  |  |  |  |  |  |  |  |  |  |  |
| 7 | MALPY00810 | 11552 | + | 843431 | 855251 |  |  |  |  |  |  | 305 |  |  |  |  |  |  |  |
| 7 | PbContig297 | 4877 | - | 846323 | 851212 |  |  |  |  |  |  |  |  |  |  |  |  |  |  |
| 7 | Pc.c001103799.Contig1 | 2344 | - | 847912 | 850249 |  |  |  |  |  |  |  |  |  |  |  |  |  |  |
| 7 | MALPY03574 | 724 | + | 850986 | 851713 |  |  |  |  |  |  | 339 | 3300 |  |  |  |  |  |  |
| 7 | Pb.c000501139.Contig1 - BP | 15482 | - | 851130 | 868098 |  |  |  |  |  |  |  |  |  |  |  |  |  |  |
| 7 | Pc.c001203892.Contig1 | 3438 | - | 851341 | 855052 |  |  |  |  |  |  |  |  |  |  |  |  |  |  |
| 7 | MALPY02566 | 3148 | + | 856419 | 859690 |  |  |  |  |  | 237 | 339 |  |  |  |  |  |  |  |
| 7 | PcContig281 - BP | 5206 | + | 857891 | 863710 |  |  |  |  |  |  |  |  |  |  |  |  |  |  |
| 7 | MALPY03494 | 302 | + | 859110 | 859397 |  |  |  |  |  |  |  |  |  |  |  |  |  |  |
| 8 | MALPY01355 | 9879 | + | 860113 | 870368 |  |  |  |  |  | 237 |  |  |  |  |  |  |  |  |
| 8 | Pc.c000202837.Contig1 | 2555 | + | 865638 | 868193 |  |  |  |  |  |  |  |  |  |  |  |  |  |  |
| 8 | MALPY01846 | 3536 | - | 870352 | 874152 |  |  |  |  |  | 237 | 232 | 1200 |  |  |  |  |  |  |
| 8 | Pc.c000803197.Contig1 | 6016 | + | 871732 | 877958 |  |  |  |  |  |  |  |  |  |  |  |  |  |  |
| 8 | Pb.c001101931.Contig1 | 2029 | - | 872734 | 874802 |  |  |  |  |  |  |  |  |  |  |  |  |  |  |
| 8 | MALPY01723 | 4336 | + | 874901 | 879225 |  |  |  |  |  |  | 232 | 600 |  |  |  |  |  |  |
| 8 | Pb.c000501519.Contig1 | 1740 | + | 876059 | 877793 |  |  |  |  |  |  |  |  |  |  |  |  |  |  |
| 8 | Pb.c001101855.Contig1 | 5564 | + | 878922 | 884477 |  |  |  |  |  |  |  |  |  |  |  |  |  |  |
| 8 | MALPY01688 | 3306 | + | 879811 | 883111 |  |  |  |  |  |  | 232 |  |  |  |  |  |  |  |
| 8 | Pc.c000602971.Contig1 | 1732 | + | 880792 | 882524 | cRMPc14032 | 41975 | 2444 | 842502 | 884477 |  |  |  | cRMPs1409 | 175531 | 2444 | 708946 | 884477 |  |
| 8 | Pb.c000501696.Contig1 | 2034 | + | 886921 | 889016 |  |  |  |  |  |  |  |  |  |  |  |  |  |  |
| 8 | MALPY00661 | 7654 | - | 887026 | 895250 |  |  |  |  |  |  | 107 | 600 |  |  |  |  |  |  |
| 8 | Pc.c000402837.Contig1 | 7114 | - | 887272 | 895111 |  |  |  |  |  |  |  |  |  |  |  |  |  |  |
| 8 | Pb.c001201695.Contig1 | 5252 | + | 892370 | 898805 |  |  |  |  |  |  |  |  |  |  |  |  |  |  |
| 8 | MALPY02953 | 2863 | + | 896021 | 898905 |  |  |  |  |  |  | 107 - NS | 2000 |  |  |  |  |  |  |
| 8 | Pc.c001606027.Contig1 | 1902 | - | 897558 | 899454 | cRMPc14033 | 12533 | 1451 | 886921 | 899454 |  |  |  |  |  |  |  |  |  |
| 8 | MALPY02195 | 11127 | - | 900905 | 912559 |  |  |  |  |  |  | 107 - NS | 400 |  |  |  |  |  |  |
| 8 | Pc.c000903294.Contig1 | 3635 | + | 902043 | 905728 |  |  |  |  |  |  |  |  |  |  |  |  |  |  |
| 8 | Pb.c001401771.Contig2 | 7104 | + | 902503 | 910197 |  |  |  |  |  |  |  |  |  |  |  |  |  |  |
| 8 | Pc.c000803218.Contig1 | 5534 | + | 906508 | 912549 |  |  |  |  |  |  |  |  |  |  |  |  |  |  |
| 8 | PbContig374 | 3126 | + | 910723 | 913873 |  |  |  |  |  |  |  |  |  |  |  |  |  |  |
| 8 | MALPY03233 | 866 | - | 912604 | 913470 |  |  |  |  |  |  | 107 | 900 |  |  |  |  |  |  |
| 8 | PcContig785 | 4394 | - | 912855 | 917301 |  |  |  |  |  |  |  |  |  |  |  |  |  |  |
| 8 | MALPY02982 | 1968 | - | 913720 | 915744 |  |  |  |  |  |  | 107 | 1000 |  |  |  |  |  |  |
| 8 | MALPY00129 | 13944 | - | 916905 | 929821 |  |  |  |  |  |  | 107 | 800 |  |  |  |  |  |  |
| 8 | Pb.c000001367.Contig1 | 5974 | + | 917297 | 923259 |  |  |  |  |  |  |  |  |  |  |  |  |  |  |
| 8 | Pb.c001702785.Contig1 | 1913 | + | 922439 | 924355 |  |  |  |  |  |  |  |  |  |  |  |  |  |  |
| 8 | Pc.c001003492.Contig1 | 1730 | + | 922972 | 924696 |  |  |  |  |  |  |  |  |  |  |  |  |  |  |
| 8 | Pb.c000301435.Contig1 | 5426 | + | 927926 | 933334 |  |  |  |  |  |  |  |  |  |  |  |  |  |  |
| JC8 | MALPY01985 | 7299 | + | 930751 | 938029 | cRMPc14034 | 37124 | 1944 | 900905 | 938029 |  | 107 | 2000 |  |  |  |  |  |  |
| 8 | Pb.c002007838.Contig1 | 1201 | + | 939973 | 941219 |  |  |  |  |  |  |  |  |  |  |  |  |  |  |
| 8 | MALPY00910 | 2968 | - | 940281 | 943285 | cRMPc14035 | 3312 | 500 | 939973 | 943285 | 357 - Ins | 107 |  |  |  |  |  |  |  |
| 8 | MALPY00485 | 20677 | + | 943785 | 965596 |  |  |  |  |  | 357 - Ins |  |  |  |  |  |  |  |  |
| 8 | PbContig368 | 5987 | + | 944462 | 950503 |  |  |  |  |  |  |  |  |  |  |  |  |  |  |
| 8 | Pc.c001505451.Contig1 | 2367 | - | 944738 | 947069 |  |  |  |  |  |  |  |  |  |  |  |  |  |  |
| 8 | MALPY03187 | 369 | + | 947978 | 948323 |  |  |  |  |  |  |  |  |  |  |  |  |  |  |
| 8 | Pc.c001103845.Contig1 | 2927 | + | 948054 | 951020 |  |  |  |  |  |  |  |  |  |  |  |  |  |  |
| 8 | Pb.c001001736.Contig1 | 1794 | + | 951009 | 952737 |  |  |  |  |  |  |  |  |  |  |  |  |  |  |
| 8 | Pc.c001104318.Contig1 | 851 | + | 951656 | 952513 |  |  |  |  |  |  |  |  |  |  |  |  |  |  |
| 8 | PbContig42 | 14001 | - | 953718 | 968058 |  |  |  |  |  |  |  |  |  |  |  |  |  |  |
| 8 | Pc.c000002735.Contig1 | 3845 | - | 955911 | 959756 |  |  |  |  |  |  |  |  |  |  |  |  |  |  |
| 8 | Pc.c001204001.Contig1 | 1926 | - | 964919 | 966848 |  |  |  |  |  |  |  |  |  |  |  |  |  |  |
| 8 | MALPY01155 | 10088 | - | 965532 | 976236 |  |  |  |  |  | 713 |  |  |  |  |  |  |  |  |
| 8 | Pc.c001204009.Contig1 | 2994 | - | 967794 | 970989 |  |  |  |  |  |  |  |  |  |  |  |  |  |  |
| 8 | Pb.c000301507.Contig1 | 3212 | - | 969021 | 972617 | cRMPc14036 | 32451 | 173 | 943785 | 976236 |  |  |  |  |  |  |  |  |  |
| 8 | Pb.c001001684.Contig1 | 5990 | + | 976409 | 982279 |  |  |  |  |  |  |  |  |  |  |  |  |  |  |
| 8 | MALPY00200 | 8617 | + | 976906 | 985415 |  |  |  |  |  | 713 | 93 | 2500 |  |  |  |  |  |  |
| 8 | Pc.c001505069.Contig1 | 1846 | + | 977808 | 979582 |  |  |  |  |  |  |  |  |  |  |  |  |  |  |
| 8 | Pc.c000202846.Contig1 | 3012 | + | 981335 | 984452 |  |  |  |  |  |  |  |  |  |  |  |  |  |  |
| 8 | Pb.c001101972.Contig1 | 2150 | - | 982477 | 984672 | cRMPc14037 | 9006 | 2540 | 976409 | 985415 |  |  |  |  |  |  |  |  |  |
| 8 | MALPY01438 | 18720 | - | 987955 | 1008639 |  |  |  |  |  |  | 93 | 1200 |  |  |  |  |  |  |
| 8 | Pb.c000401567.Contig1 | 8590 | - | 988753 | 999046 |  |  |  |  |  |  |  |  |  |  |  |  |  |  |
| 8 | PcContig59 | 9654 | + | 995456 | 1005421 |  |  |  |  |  |  |  |  |  |  |  |  |  |  |
| 8 | Pb.c000401566.Contig1 | 5672 | - | 998998 | 1005038 |  |  |  |  |  |  |  |  |  |  |  |  |  |  |
| 8 | Pb.c001502518.Contig1 | 6780 | + | 1008044 | 1014820 |  |  |  |  |  |  |  |  |  |  |  |  |  |  |
| 8 | MALPY00308 | 5093 | - | 1009382 | 1014487 |  |  |  |  |  | 21B | 93 |  |  |  |  |  |  |  |
| 8 | PcContig199 | 3225 | - | 1010453 | 1013618 |  |  |  |  |  |  |  |  |  |  |  |  |  |  |
| 8 | MALPY00309 | 11704 | - | 1014123 | 1025445 |  |  |  |  |  | 21B |  |  |  |  |  |  |  |  |
| 8 | Pb.c000401575.Contig1 | 4169 | + | 1014680 | 1018795 |  |  |  |  |  |  |  |  |  |  |  |  |  |  |
| 8 | PbContig364 | 8036 | - | 1018925 | 1026702 |  |  |  |  |  |  |  |  |  |  |  |  |  |  |
| 8 | PcContig429 | 4340 | - | 1024527 | 1028792 |  |  |  |  |  |  |  |  |  |  |  |  |  |  |
| 8 | MALPY00611 | 13413 | - | 1025885 | 1039894 |  |  |  |  |  | 21B | 216 | 2000 |  |  |  |  |  |  |
| 8 | Pb.c001602427.Contig1 | 3080 | + | 1026690 | 1029879 |  |  |  |  |  |  |  |  |  |  |  |  |  |  |
| 8 | Pb.c002006937.Contig1 | 2915 | + | 1029958 | 1032954 |  |  |  |  |  |  |  |  |  |  |  |  |  |  |
| 8 | Pb.c001202224.Contig1 | 6234 | - | 1034451 | 1040706 |  |  |  |  |  |  |  |  |  |  |  |  |  |  |
| 8 | Pc.c000602905.Contig1 | 2846 | + | 1035584 | 1038454 | cRMPc14038 | 52751 | 2189 | 987955 | 1040706 |  |  |  |  |  |  |  |  |  |
| 8 | MALPY02037 | 5269 | - | 1042895 | 1048160 |  |  |  |  |  |  | 216 | 3500 |  |  |  |  |  |  |
| 8 | Pb.c001202105.Contig1 | 2959 | + | 1043556 | 1046515 |  |  |  |  |  |  |  |  |  |  |  |  |  |  |
| 8 | Pc.c000402899.Contig1 | 3749 | + | 1043914 | 1047649 |  |  |  |  |  |  |  |  |  |  |  |  |  |  |
| 8 | MALPY03533 | 900 | - | 1044748 | 1045666 |  |  |  |  |  |  | 216 | 900 |  |  |  |  |  |  |
| 8 | MALPY03277 | 880 | + | 1046069 | 1046949 |  |  |  |  |  |  | 216 | 3000 |  |  |  |  |  |  |
| 8 | Pb.c001202104.Contig1 | 1323 | + | 1046517 | 1047879 | cRMPc14039 | 5265 | 2720 | 1042895 | 1048160 |  |  |  |  |  |  |  |  |  |
| 8 | PbContig208 | 7717 | - | 1050880 | 1058709 |  |  |  |  |  |  |  |  |  |  |  |  |  |  |
| 8 | Pc.c001707796.Contig1 | 1585 | - | 1051861 | 1053445 |  |  |  |  |  |  |  |  |  |  |  |  |  |  |
| man8 | MALPY00448 - Ins | 17751 | + | 1052047 | 1071827 |  |  |  |  |  | 82 | 216 |  |  |  |  |  |  |  |
| 8 | Pc.c001504914.Contig1 | 3882 | - | 1053684 | 1057640 |  |  |  |  |  |  |  |  |  |  |  |  |  |  |
| 8 | MALPY03146 | 109 | - | 1056370 | 1056479 |  |  |  |  |  |  |  |  |  |  |  |  |  |  |
| 8 | Pb.c001904529.Contig1 | 3330 | - | 1059527 | 1062857 |  |  |  |  |  |  |  |  |  |  |  |  |  |  |
| 8 | Pb.c001402430.Contig1 | 751 | - | 1066618 | 1067369 |  |  |  |  |  |  |  |  |  |  |  |  |  |  |
| 8 | Pb.c000401436.Contig1 | 4594 | - | 1067259 | 1071839 |  |  |  |  |  |  |  |  |  |  |  |  |  |  |
| 8 | Pc.c000903439.Contig1 | 2046 | - | 1068229 | 1070299 |  |  |  |  |  |  |  |  |  |  |  |  |  |  |
| 8 | Pc.c001404870.Contig1 | 1531 | - | 1070555 | 1072097 |  |  |  |  |  |  |  |  |  |  |  |  |  |  |
| 8 | MALPY02238 | 2821 | + | 1071538 | 1074350 |  |  |  |  |  | 82 |  |  |  |  |  |  |  |  |
| 8 | PbContig97 | 4382 | - | 1073959 | 1078341 |  |  |  |  |  |  |  |  |  |  |  |  |  |  |
| TK8 | MALPY02755 | 2732 | - | 1075388 | 1077955 |  |  |  |  |  | 60A |  |  |  |  |  |  |  |  |
| 8 | MALPY01269 | 3435 | + | 1078043 | 1081478 |  |  |  |  |  | 60A | 45 | 2000 |  |  |  |  |  |  |
| 8 | PbContig87 | 4102 | - | 1078188 | 1082290 |  |  |  |  |  |  |  |  |  |  |  |  |  |  |
| 8 | Pc.c001003747.Contig1 | 2883 | + | 1082265 | 1085124 |  |  |  |  |  |  |  |  |  |  |  |  |  |  |
| 8 | Pb.c001201691.Contig1 | 13776 | - | 1082878 | 1096908 |  |  |  |  |  |  |  |  |  |  |  |  |  |  |
| 8 | MALPY02140 | 7420 | + | 1084653 | 1092568 |  |  |  |  |  | 985 | 45 |  |  |  |  |  |  |  |
| 8 | Pc.c001203996.Contig1 | 2460 | + | 1089115 | 1091677 |  |  |  |  |  |  |  |  |  |  |  |  |  |  |
| 8 | MALPY02412 | 8493 | - | 1092164 | 1100712 |  |  |  |  |  | 985 |  |  |  |  |  |  |  |  |
| 8 | MALPY03109 | 275 | + | 1093912 | 1094187 |  |  |  |  |  |  | 116 | 8000 |  |  |  |  |  |  |
| 8 | Pb.c001802795.Contig1 | 2845 | - | 1097879 | 1100724 |  |  |  |  |  |  |  |  |  |  |  |  |  |  |
| 8 | PcContig268 | 3296 | - | 1099303 | 1103472 |  |  |  |  |  |  |  |  |  |  |  |  |  |  |
| 8 | MALPY02256 - BP | 11354 | - | 1101560 | 1113045 |  |  |  |  |  | 544 | 116 |  |  |  |  |  |  |  |
| 8 | Pb.c000201301.Contig2 - BP | 7658 | + | 1102022 | 1109606 |  |  |  |  |  |  |  |  |  |  |  |  |  |  |
| 12 | Pc.c001404874.Contig1 | 1679 | + | 1103844 | 1105481 |  |  |  |  |  |  |  |  |  |  |  |  |  |  |
| 12 | Pb.c000701537.Contig1 | 1665 | + | 1109480 | 1111098 |  |  |  |  |  |  |  |  |  |  |  |  |  |  |
| 12 | Pc.c001103119.Contig1 | 2960 | - | 1109571 | 1112482 |  |  |  |  |  |  |  |  |  |  |  |  |  |  |
| 12 | PbContig360 | 11357 | + | 1111434 | 1122768 |  |  |  |  |  |  |  |  |  |  |  |  |  |  |
| 12 | Pc.c001203220.Contig1.2 | 1262 | - | 1112820 | 1114079 |  |  |  |  |  |  |  |  |  |  |  |  |  |  |
| 12 | MALPY03123 | 1285 | - | 1113258 | 1114540 |  |  |  |  |  | 544 |  |  |  |  |  |  |  |  |
| 12 | Pc.c001504978.Contig1 | 3179 | - | 1113987 | 1117160 |  |  |  |  |  |  |  |  |  |  |  |  |  |  |
| 12 | MALPY01996 | 7330 | + | 1114690 | 1122010 |  |  |  |  |  | 544 |  |  |  |  |  |  |  |  |
| 12 | Pc.c000503258.Contig1 | 635 | - | 1115044 | 1115679 |  |  |  |  |  |  |  |  |  |  |  |  |  |  |
| 12 | Pc.c001203220.Contig1.0 | 565 | - | 1115101 | 1115666 |  |  |  |  |  |  |  |  |  |  |  |  |  |  |
| 12 | MALPY01397 | 5297 | + | 1120442 | 1125937 |  |  |  |  |  |  | 437 | 1800 |  |  |  |  |  |  |
| 12 | Pc.c001404902.Contig1 | 3820 | + | 1121735 | 1125756 |  |  |  |  |  |  |  |  |  |  |  |  |  |  |
| 12 | Pb.c001502097.Contig1 | 4147 | + | 1122731 | 1127077 |  |  |  |  |  |  |  |  |  |  |  |  |  |  |
| 12 | Pc.c000002633.Contig1 | 3617 | + | 1126176 | 1129856 |  |  |  |  |  |  |  |  |  |  |  |  |  |  |
| 12 | MALPY01533 | 8197 | + | 1127275 | 1135487 |  |  |  |  |  |  | 437 |  |  |  |  |  |  |  |
| 12 | Pb.c000601603.Contig1 | 7186 | - | 1127945 | 1135142 |  |  |  |  |  |  |  |  |  |  |  |  |  |  |
| 12 | Pc.c001003936.Contig1 | 967 | + | 1131557 | 1132533 |  |  |  |  |  |  |  |  |  |  |  |  |  |  |
| 12 | Pc.c001707708.Contig1 | 1723 | + | 1132716 | 1134505 | cRMPc14040 | 84607 | 1577 | 1050880 | 1135487 |  |  |  | cRMPs1410 | 248566 | 1577 | 886921 | 1135487 |  |
| 12 | Pb.c000901437.Contig1 | 8529 | - | 1137064 | 1145235 |  |  |  |  |  |  |  |  |  |  |  |  |  |  |
| 12 | MALPY01951 | 7679 | - | 1137844 | 1145178 |  |  |  |  |  |  |  |  |  |  |  |  |  |  |
| 12 | Pc.c001103780.Contig1 | 3385 | - | 1137907 | 1141143 |  |  |  |  |  |  |  |  |  |  |  |  |  |  |
| 12 | Pc.c000102694.Contig1 | 2508 | + | 1142020 | 1144501 | cRMPc14041 | 8171 | 885 | 1137064 | 1145235 |  |  |  | cRMPs1411 | 8171 | 885 | 1137064 | 1145235 |  |
| 12 | MALPY00556 | 25626 | + | 1146120 | 1174163 |  |  |  |  |  | 669 |  |  |  |  |  |  |  |  |
| 12 | Pb.c000501436.Contig1 | 1069 | - | 1146378 | 1147528 |  |  |  |  |  |  |  |  |  |  |  |  |  |  |
| 12 | PbContig296 | 5820 | - | 1147532 | 1154024 |  |  |  |  |  |  |  |  |  |  |  |  |  |  |
| 12 | Pc.c001002753.Contig2 | 4768 | - | 1148508 | 1154051 |  |  |  |  |  |  |  |  |  |  |  |  |  |  |
| 12 | PbContig314 | 24304 | - | 1154081 | 1180279 |  |  |  |  |  |  |  |  |  |  |  |  |  |  |
| 12 | Pc.c000103123.Contig1 | 1211 | + | 1158151 | 1159500 |  |  |  |  |  |  |  |  |  |  |  |  |  |  |
| 12 | Pc.c000102676.Contig1 | 5398 | + | 1159305 | 1165159 |  |  |  |  |  |  |  |  |  |  |  |  |  |  |
| 12 | Pc.c001303481.Contig3 | 2621 | + | 1170826 | 1173508 |  |  |  |  |  |  |  |  |  |  |  |  |  |  |
| 12 | MALPY02759 | 3511 | + | 1173775 | 1177261 |  |  |  |  |  | 669 |  |  |  |  |  |  |  |  |
| 12 | Pc.c001708836.Contig1 | 2193 | + | 1176104 | 1178278 |  |  |  |  |  |  |  |  |  |  |  |  |  |  |
| 12 | MALPY01590 | 2226 | + | 1177205 | 1179431 |  |  |  |  |  | 669 |  |  |  |  |  |  |  |  |
| 12 | Pc.c001605871.Contig1 | 2258 | + | 1178714 | 1180972 |  |  |  |  |  |  |  |  |  |  |  |  |  |  |
| 12 | PbContig393 | 8421 | + | 1180538 | 1188910 |  |  |  |  |  |  |  |  |  |  |  |  |  |  |
| 12 | MALPY00763 | 12720 | + | 1180697 | 1193790 |  |  |  |  |  | 338 | 128 | 4000 |  |  |  |  |  |  |
| 12 | Pc.c000202825.Contig1 | 5734 | + | 1181560 | 1187242 |  |  |  |  |  |  |  |  |  |  |  |  |  |  |
| 12 | Pb.c001904294.Contig1 | 1424 | + | 1189210 | 1190607 |  |  |  |  |  |  |  |  |  |  |  |  |  |  |
| 12 | Pb.c001904295.Contig1 | 2182 | - | 1190585 | 1193020 |  |  |  |  |  |  |  |  |  |  |  |  |  |  |
| 12 | Pc.c000503054.Contig1 | 2733 | - | 1192219 | 1194952 |  |  |  |  |  |  |  |  |  |  |  |  |  |  |
| man12 | MALPY02511 | 3769 | + | 1193437 | 1197448 |  |  |  |  |  | 338 |  |  |  |  |  |  |  |  |
| 12 | MALPY00625 | 11789 | - | 1196894 | 1207859 |  |  |  |  |  |  | 128 | 1300 |  |  |  |  |  |  |
| 12 | Pb.c000201286.Contig2 | 4507 | - | 1197278 | 1201619 |  |  |  |  |  |  |  |  |  |  |  |  |  |  |
| 12 | Pc.c001103838.Contig1 | 1162 | + | 1197500 | 1198553 |  |  |  |  |  |  |  |  |  |  |  |  |  |  |
| 12 | Pb.c000101362.Contig1 | 6538 | + | 1201819 | 1208141 |  |  |  |  |  |  |  |  |  |  |  |  |  |  |
| 12 | Pc.c000703890.Contig1 | 1091 | + | 1202619 | 1203707 | cRMPc14042 | 62021 | 191 | 1146120 | 1208141 |  |  |  |  |  |  |  |  |  |
| 12 | MALPY03360 | 1012 | + | 1208332 | 1209335 | cRMPc14043 | 1003 | 1296 | 1208332 | 1209335 |  | 128 | 1900 |  |  |  |  |  |  |
| 12 | MALPY01794 | 3965 | - | 1210631 | 1214686 |  |  |  |  |  | 8 | 128 |  |  |  |  |  |  |  |
| 12 | PbContig294 | 4235 | + | 1211719 | 1216045 |  |  |  |  |  |  |  |  |  |  |  |  |  |  |
| 12 | MALPY03260 | 781 | - | 1212064 | 1212845 |  |  |  |  |  |  |  |  |  |  |  |  |  |  |
| 12 | MALPY00049 | 13808 | + | 1215573 | 1230607 |  |  |  |  |  | 8 |  |  |  |  |  |  |  |  |
| 12 | Pb.c001502595.Contig1 | 1760 | - | 1217797 | 1219578 |  |  |  |  |  |  |  |  |  |  |  |  |  |  |
| 12 | Pb.c000901786.Contig1 | 2817 | + | 1225393 | 1228327 |  |  |  |  |  |  |  |  |  |  |  |  |  |  |
| 12 | Pc.c000503274.Contig1 | 3981 | + | 1225969 | 1230167 |  |  |  |  |  |  |  |  |  |  |  |  |  |  |
| 12 | Pb.c000901787.Contig1 | 2697 | + | 1228277 | 1231082 | cRMPc14044 | 20451 | 1489 | 1210631 | 1231082 |  |  |  |  |  |  |  |  |  |
| 12 | PbContig127 | 12842 | + | 1232571 | 1246056 |  |  |  |  |  |  |  |  |  |  |  |  |  |  |
| 12 | MALPY00416 | 8872 | + | 1233089 | 1242175 |  |  |  |  |  | 8 |  |  |  |  |  |  |  |  |
| 12 | Pc.c001103107.Contig2 | 5257 | - | 1234062 | 1239731 |  |  |  |  |  |  |  |  |  |  |  |  |  |  |
| 12 | Pc.c000102569.Contig1 | 2588 | - | 1239902 | 1242661 |  |  |  |  |  |  |  |  |  |  |  |  |  |  |
| 12 | MALPY02681 | 2982 | - | 1242205 | 1245346 |  |  |  |  |  | 8 | 161 | 900 |  |  |  |  |  |  |
| 12 | Pc.c001104328.Contig1 | 895 | - | 1242709 | 1243628 |  |  |  |  |  |  |  |  |  |  |  |  |  |  |
| 12 | Pc.c000903505.Contig1 | 3026 | + | 1244088 | 1247230 |  |  |  |  |  |  |  |  |  |  |  |  |  |  |
| 12 | MALPY01702 | 7188 | + | 1245725 | 1253141 |  |  |  |  |  |  | 161 | 1900 |  |  |  |  |  |  |
| 12 | Pb.c001201940.Contig1 | 3413 | + | 1246131 | 1249506 |  |  |  |  |  |  |  |  |  |  |  |  |  |  |
| 12 | PcContig599 | 2047 | + | 1247133 | 1249189 |  |  |  |  |  |  |  |  |  |  |  |  |  |  |
| 12 | PcContig315 | 2252 | + | 1249246 | 1251756 |  |  |  |  |  |  |  |  |  |  |  |  |  |  |
| 12 | Pb.c000401549.Contig1 | 3066 | + | 1249963 | 1253032 | cRMPc14045 | 20570 | 634 | 1232571 | 1253141 |  |  |  |  |  |  |  |  |  |
| 12 | Pb.c000401548.Contig1 | 3210 | + | 1253775 | 1256994 |  |  |  |  |  |  |  |  |  |  |  |  |  |  |
| 12 | PcContig328 | 4142 | + | 1254311 | 1258459 |  |  |  |  |  |  |  |  |  |  |  |  |  |  |
| 12 | MALPY01458 | 12239 | + | 1254578 | 1268419 |  |  |  |  |  | 732 | 161 |  |  |  |  |  |  |  |
| 12 | PbContig253 | 13284 | - | 1258611 | 1273196 |  |  |  |  |  |  |  |  |  |  |  |  |  |  |
| 12 | Pc.c000102633.Contig1 | 1065 | + | 1259338 | 1260442 |  |  |  |  |  |  |  |  |  |  |  |  |  |  |
| 12 | PcContig701 | 2446 | - | 1261999 | 1264431 |  |  |  |  |  |  |  |  |  |  |  |  |  |  |
| 12 | MALPY01499 | 7691 | - | 1268415 | 1276171 |  |  |  |  |  | 732 | 73 | 9100 |  |  |  |  |  |  |
| 12 | PbContig115 | 5150 | - | 1273307 | 1278577 |  |  |  |  |  |  |  |  |  |  |  |  |  |  |
| 12 | MALPY02219 | 9244 | + | 1277873 | 1287041 |  |  |  |  |  |  | 73 | 2000 |  |  |  |  |  |  |
| 12 | Pb.c001703176.Contig1 | 1307 | - | 1279728 | 1281035 |  |  |  |  |  |  |  |  |  |  |  |  |  |  |
| 12 | Pc.c001707735.Contig1 | 620 | + | 1280384 | 1281004 |  |  |  |  |  |  |  |  |  |  |  |  |  |  |
| 12 | Pb.c000201613.Contig1 | 3947 | + | 1281984 | 1285788 |  |  |  |  |  |  |  |  |  |  |  |  |  |  |
| 12 | Pc.c001003363.Contig1 | 1022 | + | 1282208 | 1283230 | cRMPc14046 | 33266 | 1349 | 1253775 | 1287041 |  |  |  |  |  |  |  |  |  |
| 12 | Pc.c000002081.Contig2 | 4615 | + | 1288390 | 1293053 |  |  |  |  |  |  |  |  |  |  |  |  |  |  |
| 12 | MALPY00487 | 18384 | + | 1288455 | 1307342 |  |  |  |  |  |  | 73 |  |  |  |  |  |  |  |
| 12 | Pb.c000501396.Contig1 | 4740 | + | 1288462 | 1293218 |  |  |  |  |  |  |  |  |  |  |  |  |  |  |
| 12 | MALPY03578 | 1418 | + | 1291465 | 1293125 |  |  |  |  |  |  |  |  |  |  |  |  |  |  |
| 12 | Pc.c000903957.Contig1 | 6038 | + | 1295501 | 1301840 |  |  |  |  |  |  |  |  |  |  |  |  |  |  |
| 12 | Pb.c000001798.Contig1 | 5049 | + | 1295725 | 1300771 |  |  |  |  |  |  |  |  |  |  |  |  |  |  |
| 12 | Pc.c001104346.Contig1 | 869 | + | 1302113 | 1302982 |  |  |  |  |  |  |  |  |  |  |  |  |  |  |
| 12 | PbContig205 | 3448 | + | 1302114 | 1305296 |  |  |  |  |  |  |  |  |  |  |  |  |  |  |
| 12 | MALPY03475 | 228 | - | 1302818 | 1303046 | cRMPc14047 | 18952 | 517 | 1288390 | 1307342 |  | 206 | 9000 |  |  |  |  |  |  |
| 12 | Pb.c002007047.Contig1 | 2129 | + | 1307859 | 1309988 |  |  |  |  |  |  |  |  |  |  |  |  |  |  |
| man12 | MALPY00505 - Ins | 26409 | - | 1308772 | 1336093 |  |  |  |  |  |  | 206 | 500? (io 5000) | |  |  |  |  |  |
| 12 | Pb.c000401458.Contig1 | 4106 | + | 1314235 | 1318394 |  |  |  |  |  |  |  |  |  |  |  |  |  |  |
| 12 | Pc.c000002529.Contig1 | 2897 | + | 1315825 | 1318749 |  |  |  |  |  |  |  |  |  |  |  |  |  |  |
| 12 | Pb.c000401456.Contig1 | 4058 | + | 1318029 | 1322147 |  |  |  |  |  |  |  |  |  |  |  |  |  |  |
| man12 | PbContig63 - Ins | 13481 | + | 1322928 | 1336888 |  |  |  |  |  |  |  |  |  |  |  |  |  |  |
| 12 | Pc.c001812545.Contig1 | 615 | - | 1324226 | 1324868 |  |  |  |  |  |  |  |  |  |  |  |  |  |  |
| 12 | Pc.c001304874.Contig1 | 709 | - | 1325130 | 1325839 |  |  |  |  |  |  |  |  |  |  |  |  |  |  |
| 12 | Pc.c000402308.Contig2 | 2181 | - | 1326055 | 1328227 |  |  |  |  |  |  |  |  |  |  |  |  |  |  |
| 12 | Pc.c001606243.Contig1 | 710 | - | 1327966 | 1328676 |  |  |  |  |  |  |  |  |  |  |  |  |  |  |
| 12 | PcContig442 | 1534 | - | 1328506 | 1330067 |  |  |  |  |  |  |  |  |  |  |  |  |  |  |
| 12 | Pc.c000503436.Contig1 | 754 | - | 1330143 | 1330975 |  |  |  |  |  |  |  |  |  |  |  |  |  |  |
| 12 | Pc.c001002716.Contig1 | 6020 | - | 1331165 | 1337688 |  |  |  |  |  |  |  |  |  |  |  |  |  |  |
| 12 | Pc.c001404849.Contig1 | 633 | + | 1334902 | 1335523 |  |  |  |  |  |  |  |  |  |  |  |  |  |  |
| 12 | MALPY00488 | 10783 | - | 1336204 | 1347369 |  |  |  |  |  |  | 206 | 5000? (io 500) | |  |  |  |  |  |
| 12 | Pb.c000501448.Contig1 | 8695 | + | 1337132 | 1346290 |  |  |  |  |  |  |  |  |  |  |  |  |  |  |
| 12 | Pc.c001708801.Contig1 | 827 | + | 1338572 | 1339393 |  |  |  |  |  |  |  |  |  |  |  |  |  |  |
| 12 | Pc.c001003839.Contig1 | 1170 | + | 1339505 | 1340663 |  |  |  |  |  |  |  |  |  |  |  |  |  |  |
| 12 | Pc.c001812891.Contig1 | 759 | + | 1340879 | 1341596 |  |  |  |  |  |  |  |  |  |  |  |  |  |  |
| 12 | Pc.c000903527.Contig1 | 4137 | + | 1342412 | 1346549 | cRMPc14048 | 39510 | 593 | 1307859 | 1347369 |  |  |  |  |  |  |  |  |  |
| man12 | MALPY01651 | 4603 | + | 1347962 | 1351753 | cRMPc14049 | 3791 | 508 | 1347962 | 1351753 | 427 |  |  |  |  |  |  |  |  |
| 12 | MALPY02273 | 11640 | + | 1352261 | 1364205 |  |  |  |  |  | 427 | 206 |  |  |  |  |  |  |  |
| 12 | Pc.c000003133.Contig1 | 1334 | + | 1354825 | 1356294 |  |  |  |  |  |  |  |  |  |  |  |  |  |  |
| 12 | Pb.c002112997.Contig1 | 701 | - | 1355693 | 1356394 |  |  |  |  |  |  |  |  |  |  |  |  |  |  |
| 12 | Pc.c001708200.Contig1 | 1334 | + | 1356295 | 1357575 |  |  |  |  |  |  |  |  |  |  |  |  |  |  |
| 12 | Pb.c001201686.Contig1 | 4518 | - | 1356685 | 1361481 | cRMPc14050 | 11944 | 192 | 1352261 | 1364205 |  |  |  | cRMPs1412 | 218085 | 192 | 1146120 | 1364205 |  |
| 12 | MALPY02467 | 2050 | + | 1364397 | 1366444 |  |  |  |  |  | 276 |  |  |  |  |  |  |  |  |
| 12 | Pb.c001602544.Contig1 | 2155 | + | 1364538 | 1366690 | cRMPc14051 | 2293 | 2584 | 1364397 | 1366690 |  |  |  |  |  |  |  |  |  |
| 12 | MALPY01525 | 6538 | + | 1369274 | 1375782 |  |  |  |  |  | 276 |  |  |  |  |  |  |  |  |
| 12 | Pb.c001904339.Contig1 | 2625 | + | 1372099 | 1374694 |  |  |  |  |  |  |  |  |  |  |  |  |  |  |
| 12 | Pc.c001813362.Contig1 | 889 | - | 1372950 | 1373812 | cRMPc14052 | 6508 | 1667 | 1369274 | 1375782 |  |  |  |  |  |  |  |  |  |
| 12 | MALPY01009 | 9388 | - | 1377449 | 1387794 |  |  |  |  |  | 276 |  |  |  |  |  |  |  |  |
| 12 | Pb.c001202108.Contig1 | 5294 | + | 1377510 | 1383504 |  |  |  |  |  |  |  |  |  |  |  |  |  |  |
| 12 | PcContig763 | 3609 | + | 1377638 | 1381971 |  |  |  |  |  |  |  |  |  |  |  |  |  |  |
| 12 | Pc.c000703281.Contig1 | 1947 | + | 1381960 | 1383946 |  |  |  |  |  |  |  |  |  |  |  |  |  |  |
| man12 | MALPY01713 | 5982 | + | 1387433 | 1393421 |  |  |  |  |  | 276 |  |  |  |  |  |  |  |  |
| 12 | Pb.c000601253.Contig1 | 13583 | + | 1387526 | 1401160 |  |  |  |  |  |  |  |  |  |  |  |  |  |  |
| 12 | MALPY00301 | 13051 | - | 1393544 | 1407437 |  |  |  |  |  | 276 |  |  |  |  |  |  |  |  |
| 12 | Pc.c001505149.Contig1 | 2006 | - | 1397970 | 1400030 |  |  |  |  |  |  |  |  |  |  |  |  |  |  |
| 12 | Pb.c000101357.Contig1 | 7102 | - | 1403618 | 1410448 |  |  |  |  |  |  |  |  |  |  |  |  |  |  |
| 12 | MALPY00918 | 9291 | + | 1407304 | 1416880 |  |  |  |  |  | 276 - Ins |  |  |  |  |  |  |  |  |
| 12 | Pc.c001003557.Contig1 | 1094 | - | 1408246 | 1409333 |  |  |  |  |  |  |  |  |  |  |  |  |  |  |
| 12 | Pc.c001003289.Contig1 | 3440 | - | 1409564 | 1413008 |  |  |  |  |  |  |  |  |  |  |  |  |  |  |
| 12 | Pb.c000101356.Contig1 | 5207 | - | 1410859 | 1416051 | cRMPc14053 | 39431 | 500 | 1377449 | 1416880 |  |  |  |  |  |  |  |  |  |
| man12 | MALPY00159 - Ins | 11897 | + | 1417380 | 1429133 |  |  |  |  |  | 276 - Ins |  |  |  |  |  |  |  |  |
| 12 | Pb.c001101858.Contig1 | 1887 | + | 1417798 | 1419697 |  |  |  |  |  |  |  |  |  |  |  |  |  |  |
| 12 | Pc.c000603459.Contig1 | 2133 | + | 1421160 | 1423332 |  |  |  |  |  |  |  |  |  |  |  |  |  |  |
| 12 | Pb.c001301905.Contig1 | 4223 | - | 1421732 | 1425990 |  |  |  |  |  |  |  |  |  |  |  |  |  |  |
| 12 | PcContig290 | 731 | + | 1423289 | 1424020 |  |  |  |  |  |  |  |  |  |  |  |  |  |  |
| 12 | PbContig90 | 5259 | - | 1426596 | 1431634 |  |  |  |  |  |  |  |  |  |  |  |  |  |  |
| 12 | Pc.c000202721.Contig1 | 3258 | + | 1426750 | 1429945 |  |  |  |  |  |  |  |  |  |  |  |  |  |  |
| man12 | MALPY02628 | 5037 | + | 1430358 | 1435395 | cRMPc14054 | 18015 | 1200 | 1417380 | 1435395 |  |  |  | cRMPs1413 | 70998 | 1200 | 1364397 | 1435395 |  |
| JC12 | MALPY02483 | 2369 | xxx | 1435000 |  |  |  |  |  |  | 235 |  |  |  |  |  |  |  |  |
| 12 | MALPY00995 | 24364 | + | 1436595 | 1463631 |  |  |  |  |  | 235 |  |  |  |  |  |  |  |  |
| 12 | Pb.c000801727.Contig1 | 3290 | - | 1439584 | 1442874 |  |  |  |  |  |  |  |  |  |  |  |  |  |  |
| 12 | Pb.c002007118.Contig1 | 2505 | - | 1445311 | 1447876 |  |  |  |  |  |  |  |  |  |  |  |  |  |  |
| 12 | PcContig248 | 3177 | + | 1445942 | 1449814 |  |  |  |  |  |  |  |  |  |  |  |  |  |  |
| 12 | Pc.c000102603.Contig1 | 3367 | + | 1454348 | 1457714 |  |  |  |  |  |  |  |  |  |  |  |  |  |  |
| 12 | Pb.c001401707.Contig1 | 3890 | - | 1454579 | 1458472 |  |  |  |  |  |  |  |  |  |  |  |  |  |  |
| 12 | MALPY03439 | 744 | - | 1456116 | 1456863 |  |  |  |  |  |  | 40 | 8000 |  |  |  |  |  |  |
| 12 | Pb.c000301502.Contig1 | 2877 | + | 1460325 | 1463241 |  |  |  |  |  |  |  |  |  |  |  |  |  |  |
| 12 | Pc.c001304791.Contig1 | 802 | - | 1461480 | 1462312 |  |  |  |  |  |  |  |  |  |  |  |  |  |  |
| 12 | Pc.c000903387.Contig1 | 1850 | - | 1461952 | 1463760 |  |  |  |  |  |  |  |  |  |  |  |  |  |  |
| 12 | MALPY02667 | 4136 | + | 1463700 | 1467815 |  |  |  |  |  | 235 | 40 | 800 |  |  |  |  |  |  |
| 12 | PbContig117 | 3084 | - | 1464545 | 1467629 |  |  |  |  |  |  |  |  |  |  |  |  |  |  |
| 12 | Pc.c000002614.Contig1 | 1866 | - | 1465106 | 1466996 | cRMPc14055 | 31220 | 912 | 1436595 | 1467815 |  |  |  |  |  |  |  |  |  |
| 12 | MALPY03307 | 741 | + | 1468727 | 1469468 |  |  |  |  |  |  | 40 | 400 |  |  |  |  |  |  |
| 12 | Pc.c000002616.Contig1 | 1811 | - | 1469047 | 1470924 |  |  |  |  |  |  |  |  |  |  |  |  |  |  |
| 12 | MALPY02203 | 4970 | - | 1469410 | 1474055 |  |  |  |  |  | 78 | 40 |  |  |  |  |  |  |  |
| 12 | Pb.c001202025.Contig1 | 3262 | + | 1471046 | 1474239 |  |  |  |  |  |  |  |  |  |  |  |  |  |  |
| 12 | PcContig764 | 5165 | - | 1472885 | 1478093 |  |  |  |  |  |  |  |  |  |  |  |  |  |  |
| 12 | MALPY03404 | 583 | + | 1473489 | 1474032 |  |  |  |  |  |  | 270 | 1000 |  |  |  |  |  |  |
| 12 | MALPY01918 | 5895 | - | 1473976 | 1479979 |  |  |  |  |  | 78 | 270 |  |  |  |  |  |  |  |
| 12 | Pb.c001101937.Contig1 | 2530 | + | 1474311 | 1476981 |  |  |  |  |  |  |  |  |  |  |  |  |  |  |
| 12 | Pc.c001003913.Contig1 | 646 | - | 1474326 | 1475100 | cRMPc14056 | 11252 | 2208 | 1468727 | 1479979 |  |  |  | cRMPs1414 | 43384 | 2208 | 1436595 | 1479979 |  |
| man12 | MALPY02723 | 3497 | - | 1482187 | 1485661 |  |  |  |  |  | 264B |  |  |  |  |  |  |  |  |
| man12 | MALPY01264 | 3161 | + | 1485243 | 1488683 | cRMPc14057 | 6496 | 2697 | 1482187 | 1488683 | 264B |  |  | cRMPs1415 | 6496 | 2697 | 1482187 | 1488683 |  |
| 12 | MALPY02333 | 9642 | - | 1491380 | 1501200 |  |  |  |  |  |  | 208 | 1300 |  |  |  |  |  |  |
| 12 | PbContig140 | 8128 | + | 1494269 | 1502707 |  |  |  |  |  |  |  |  |  |  |  |  |  |  |
| 12 | Pc.c001605938.Contig1 | 618 | + | 1496647 | 1497265 |  |  |  |  |  |  |  |  |  |  |  |  |  |  |
| 12 | Pc.c001203816.Contig1 | 3393 | + | 1498224 | 1501724 |  |  |  |  |  |  |  |  |  |  |  |  |  |  |
| 12 | MALPY00940 | 6224 | - | 1501800 | 1508255 |  |  |  |  |  | 259 | 208 |  |  |  |  |  |  |  |
| 12 | Pc.c001605860.Contig1 | 1556 | + | 1502726 | 1504288 |  |  |  |  |  |  |  |  |  |  |  |  |  |  |
| 12 | Pb.c000801697.Contig1 | 1100 | - | 1503873 | 1504973 |  |  |  |  |  |  |  |  |  |  |  |  |  |  |
| 12 | Pc.c001405210.Contig1 | 872 | + | 1504606 | 1505498 |  |  |  |  |  |  |  |  |  |  |  |  |  |  |
| 12 | MALPY03447 | 668 | + | 1504814 | 1505482 | cRMPc14058 | 16875 | 358 | 1491380 | 1508255 |  |  |  |  |  |  |  |  |  |
| man12 | MALPY01718 | 4125 | + | 1508613 | 1512913 | cRMPc14059 | 4300 | 2195 | 1508613 | 1512913 | 259 |  |  | cRMPs1416 | 21533 | 2195 | 1491380 | 1512913 |  |
| 12 | Pc.c000302923.Contig1 | 3344 | - | 1515108 | 1518617 |  |  |  |  |  |  |  |  |  |  |  |  |  |  |
| 12 | Pb.c000901798.Contig1 | 5295 | - | 1515530 | 1520949 |  |  |  |  |  |  |  |  |  |  |  |  |  |  |
| 12 | MALPY01712 | 4598 | - | 1515629 | 1520329 |  |  |  |  |  |  |  |  |  |  |  |  |  |  |
| 12 | MALPY03521 | 623 | + | 1518672 | 1519307 |  |  |  |  |  |  | 474 | 1800 |  |  |  |  |  |  |
| 12 | Pc.c001304609.Contig1 | 2454 | - | 1518915 | 1521372 | cRMPc14060 | 6264 | 558 | 1515108 | 1521372 |  |  |  |  |  |  |  |  |  |
| 12 | MALPY01922 | 3491 | - | 1521930 | 1525418 |  |  |  |  |  | 422 | 474 |  |  |  |  |  |  |  |
| 12 | Pc.c000202766.Contig1 | 991 | - | 1523620 | 1524608 |  |  |  |  |  |  |  |  |  |  |  |  |  |  |
| 12 | Pb.c000501473.Contig1 | 2792 | - | 1524971 | 1527608 |  |  |  |  |  |  |  |  |  |  |  |  |  |  |
| 12 | MALPY01359 | 2392 | - | 1525580 | 1527795 |  |  |  |  |  | 422 | 295 | 600 |  |  |  |  |  |  |
| 12 | Pc.c000003082.Contig1 | 775 | - | 1526395 | 1527125 | cRMPc14061 | 5865 | 340 | 1521930 | 1527795 |  |  |  |  |  |  |  |  |  |
| 12 | Pb.c000501471.Contig1 | 918 | - | 1528135 | 1529053 |  |  |  |  |  |  |  |  |  |  |  |  |  |  |
| 12 | MALPY03530 | 650 | + | 1528519 | 1529169 | cRMPc14062 | 1034 | 229 | 1528135 | 1529169 |  | 295 | 6000 |  |  |  |  |  |  |
| 12 | Pb.c001402271.Contig1 | 3949 | - | 1529398 | 1533350 | cRMPc14063 | 3952 | 2848 | 1529398 | 1533350 |  |  |  |  |  |  |  |  |  |
| 12 | MALPY02615 | 3147 | + | 1536198 | 1539342 |  |  |  |  |  | 110 | 295 |  |  |  |  |  |  |  |
| 12 | Pb.c000002054.Contig1 | 1165 | - | 1536830 | 1538021 |  |  |  |  |  |  |  |  |  |  |  |  |  |  |
| 12 | Pc.c000502972.Contig1 | 5890 | - | 1536946 | 1542845 |  |  |  |  |  |  |  |  |  |  |  |  |  |  |
| 12 | Pb.c002006791.Contig1 | 2267 | + | 1538840 | 1541140 |  |  |  |  |  |  |  |  |  |  |  |  |  |  |
| man12 | MALPY02272 | 4577 | - | 1539654 | 1544340 |  |  |  |  |  | 110 |  |  |  |  |  |  |  |  |
| man12 | MALPY02732 | 2074 | - | 1544106 | 1546180 |  |  |  |  |  | 110 |  |  |  |  |  |  |  | MALPY02272 |
| 12 | MALPY02694 | 2815 | + | 1545934 | 1548803 |  |  |  |  |  | 110 | 374 | 2000 |  |  |  |  |  |  |
| 12 | Pb.c000501518.Contig1 | 933 | + | 1547353 | 1548342 |  |  |  |  |  |  |  |  |  |  |  |  |  |  |
| 12 | PcContig536 | 5789 | + | 1547716 | 1553948 |  |  |  |  |  |  |  |  |  |  |  |  |  |  |
| 12 | Pb.c000901825.Contig1 | 3846 | + | 1549638 | 1553484 |  |  |  |  |  |  |  |  |  |  |  |  |  |  |
| 12 | MALPY01893 | 9059 | + | 1549914 | 1560626 |  |  |  |  |  | 721 | 374 |  |  |  |  |  |  |  |
| 12 | Pc.c001203843.Contig1 | 2320 | + | 1557884 | 1560366 |  |  |  |  |  |  |  |  |  |  |  |  |  |  |
| 12 | Pb.c001402186.Contig1 | 2519 | + | 1558200 | 1560701 | cRMPc14064 | 24503 | 538 | 1536198 | 1560701 |  |  |  |  |  |  |  |  |  |
| 12 | MALPY01921 | 8444 | + | 1561239 | 1569287 |  |  |  |  |  | 721 |  |  |  |  |  |  |  |  |
| 12 | Pb.c001301749.Contig1 | 2151 | - | 1561474 | 1563625 |  |  |  |  |  |  |  |  |  |  |  |  |  |  |
| 12 | Pc.c000303008.Contig1 | 3866 | - | 1565044 | 1569122 |  |  |  |  |  |  |  |  |  |  |  |  |  |  |
| 12 | Pb.c001502451.Contig1 | 2600 | - | 1565058 | 1567875 | cRMPc14065 | 8048 | 85 | 1561239 | 1569287 |  |  |  | cRMPs1417 | 54179 | 85 | 1515108 | 1569287 |  |
| 12 | MALPY03529 | 794 | - | 1569372 | 1570166 |  |  |  |  |  |  | 476 | 2300 |  |  |  |  |  |  |
| 12 | Pb.c001702950.Contig1 | 1396 | - | 1569791 | 1571187 | cRMPc14066 | 1815 | 236 | 1569372 | 1571187 |  |  |  |  |  |  |  |  |  |
| 12 | Pb.c001702951.Contig1 | 1742 | - | 1571423 | 1573165 |  |  |  |  |  |  |  |  |  |  |  |  |  |  |
| 12 | MALPY00514 | 14535 | + | 1572623 | 1585682 |  |  |  |  |  | 1108 | 476 |  |  |  |  |  |  |  |
| 12 | Pc.c001606522.Contig1 | 1123 | + | 1573387 | 1574513 |  |  |  |  |  |  |  |  |  |  |  |  |  |  |
| 12 | Pb.c000601537.Contig1 | 10571 | - | 1574455 | 1583777 |  |  |  |  |  |  |  |  |  |  |  |  |  |  |
| 12 | Pc.c000402830.Contig1 | 3882 | + | 1575071 | 1579029 |  |  |  |  |  |  |  |  |  |  |  |  |  |  |
| 12 | Pc.c001504180.Contig2 | 4527 | + | 1579004 | 1582332 |  |  |  |  |  |  |  |  |  |  |  |  |  |  |
| man12 | Pb.c001401795.Contig1 - Ins | 7861 | - | 1584397 | 1592781 |  |  |  |  |  |  |  |  |  |  |  |  |  |  |
| man12 | MALPY01026 - Ins | 7917 | + | 1586231 | 1595052 |  |  |  |  |  | 1108 | 433 | 3000 |  |  |  |  |  |  |
| 12 | Pc.c001708286.Contig1 | 1015 | + | 1586252 | 1587261 |  |  |  |  |  |  |  |  |  |  |  |  |  |  |
| 12 | PcContig634 | 3343 | + | 1589422 | 1593741 | cRMPc14067 | 23629 | 416 | 1571423 | 1595052 |  |  |  |  |  |  |  |  |  |
| 12 | MALPY03154 | xxx | + | 1595468 | 1596620 |  |  |  |  |  | 1108 |  |  |  |  |  |  |  |  |
| 12 | PcContig642 | 2379 | + | 1595869 | 1598425 | cRMPc14068 | 2957 | 402 | 1595468 | 1598425 |  |  |  |  |  |  |  |  |  |
| 12 | MALPY03312 | 879 | + | 1598827 | 1599718 |  |  |  |  |  |  | 433 | 300 |  |  |  |  |  |  |
| 12 | PcContig348 | 3413 | + | 1598952 | 1602545 |  |  |  |  |  |  |  |  |  |  |  |  |  |  |
| 12 | MALPY02325 | 7593 | - | 1599678 | 1607127 |  |  |  |  |  | 579 | 433 |  |  |  |  |  |  |  |
| 12 | Pb.c001702366.Contig1 | 2167 | + | 1600324 | 1602492 |  |  |  |  |  |  |  |  |  |  |  |  |  |  |
| 12 | Pb.c002006883.Contig1 | 2902 | - | 1604145 | 1607098 |  |  |  |  |  |  |  |  |  |  |  |  |  |  |
| 12 | MALPY02479 | 3947 | - | 1607041 | 1611103 | cRMPc14069 | 12276 | 537 | 1598827 | 1611103 | 579 | 305 | 6000 |  |  |  |  |  |  |
| man12 | MALPY01326 | 2306 | + | 1611640 | 1614063 | cRMPc14070 | 2423 | 910 | 1611640 | 1614063 | 579 |  |  |  |  |  |  |  |  |
| 12 | PbContig329 | 4714 | + | 1614973 | 1619738 |  |  |  |  |  |  |  |  |  |  |  |  |  |  |
| 12 | Pc.c000503037.Contig1 | 2363 | - | 1616173 | 1618627 |  |  |  |  |  |  |  |  |  |  |  |  |  |  |
| 12 | MALPY01216 | 3555 | + | 1616312 | 1619918 |  |  |  |  |  | 508 | 305 |  |  |  |  |  |  |  |
| 12 | MALPY03095 | 211 | + | 1618034 | 1618245 | cRMPc14071 | 4945 | 472 | 1614973 | 1619918 |  | 31 | 2100 |  |  |  |  |  |  |
| 12 | MALPY01614 | 4755 | + | 1620390 | 1625358 |  |  |  |  |  | 508 | 31 | 400 |  |  |  |  |  |  |
| 12 | Pb.c000701573.Contig1 | 3615 | + | 1620810 | 1624428 |  |  |  |  |  |  |  |  |  |  |  |  |  |  |
| 12 | Pc.c001505316.Contig1 | 618 | + | 1622925 | 1623543 |  |  |  |  |  |  |  |  |  |  |  |  |  |  |
| 12 | Pc.c001505014.Contig1 | 1268 | + | 1623658 | 1624908 |  |  |  |  |  |  |  |  |  |  |  |  |  |  |
| 12 | Pb.c000601513.Contig1 | 2057 | + | 1624547 | 1626712 |  |  |  |  |  |  |  |  |  |  |  |  |  |  |
| 12 | MALPY03369 | 884 | + | 1625308 | 1626192 |  |  |  |  |  |  | 31 | 400 |  |  |  |  |  |  |
| 12 | MALPY00589 | 6034 | + | 1626274 | 1632611 |  |  |  |  |  |  | 31 |  |  |  |  |  |  |  |
| 12 | Pb.c000601511.Contig1 | 1811 | + | 1626891 | 1628699 |  |  |  |  |  |  |  |  |  |  |  |  |  |  |
| 12 | Pb.c002113556.Contig1 | 1088 | + | 1629456 | 1630496 | cRMPc14072 | 12221 | 3630 | 1620390 | 1632611 |  |  |  | cRMPs1418 | 63239 | 3630 | 1569372 | 1632611 |  |
| 12 | MALPY00020 | 12087 | - | 1636241 | 1649025 |  |  |  |  |  |  |  |  |  |  |  |  |  |  |
| 12 | Pb.c000301431.Contig1 | 2019 | - | 1636766 | 1639053 |  |  |  |  |  |  |  |  |  |  |  |  |  |  |
| 12 | Pc.c001811664.Contig1 | 3817 | + | 1638080 | 1642357 |  |  |  |  |  |  |  |  |  |  |  |  |  |  |
| 12 | PbContig271 | 12204 | - | 1639481 | 1651685 |  |  |  |  |  |  |  |  |  |  |  |  |  |  |
| 12 | MALPY00149 | 15734 | + | 1639670 | 1655132 |  |  |  |  |  |  | 461 | 1600 |  |  |  |  |  |  |
| 12 | Pb.c001402058.Contig1 | 6801 | + | 1649421 | 1655955 |  |  |  |  |  |  |  |  |  |  |  |  |  |  |
| 12 | Pc.c001708952.Contig1 | 640 | - | 1651324 | 1651964 | cRMPc14073 | 19714 | 1554 | 1636241 | 1655955 |  |  |  |  |  |  |  |  |  |
| 12 | MALPY01203 | 15757 | + | 1657509 | 1673562 |  |  |  |  |  | 144 | 461 |  |  |  |  |  |  |  |
| 12 | Pb.c001301450.Contig1 | 3340 | + | 1657850 | 1661250 |  |  |  |  |  |  |  |  |  |  |  |  |  |  |
| 12 | PbContig355 | 11865 | + | 1663081 | 1675189 |  |  |  |  |  |  |  |  |  |  |  |  |  |  |
| 12 | Pc.c000402286.Contig1 | 3979 | + | 1664452 | 1667983 |  |  |  |  |  |  |  |  |  |  |  |  |  |  |
| 12 | MALPY03222 | 1071 | + | 1671559 | 1672642 |  |  |  |  |  | 144 |  |  |  |  |  |  |  |  |
| 12 | Pc.c000502405.Contig1 | 4663 | - | 1671748 | 1676613 |  |  |  |  |  |  |  |  |  |  |  |  |  |  |
| 12 | MALPY01841 | 5686 | - | 1672197 | 1678597 |  |  |  |  |  | 144 | 203 | 700 |  |  |  |  |  |  |
| 12 | Pc.c001812548.Contig1 | 941 | - | 1677401 | 1678390 | cRMPc14074 | 21088 | 598 | 1657509 | 1678597 |  |  |  |  |  |  |  |  |  |
| 12 | MALPY03217 | 890 | - | 1679195 | 1680061 |  |  |  |  |  |  | 203 | 1000 |  |  |  |  |  |  |
| 12 | Pc.c000402914.Contig1 | 3657 | - | 1679519 | 1683226 |  |  |  |  |  |  |  |  |  |  |  |  |  |  |
| 12 | Pb.c000401715.Contig1 | 3589 | - | 1679668 | 1683234 |  |  |  |  |  |  |  |  |  |  |  |  |  |  |
| 12 | MALPY00811 | 10481 | + | 1680241 | 1690708 |  |  |  |  |  | 214 | 203 |  |  |  |  |  |  |  |
| 12 | Pb.c000401504.Contig1 | 9901 | - | 1683628 | 1693784 |  |  |  |  |  |  |  |  |  |  |  |  |  |  |
| 12 | Pc.c000603010.Contig1 | 1250 | - | 1689112 | 1690332 |  |  |  |  |  |  |  |  |  |  |  |  |  |  |
| 12 | MALPY00545 | 9901 | - | 1690775 | 1703323 |  |  |  |  |  | 214 |  |  |  |  |  |  |  |  |
| 12 | Pc.c001812807.Contig1 | 1660 | - | 1690825 | 1692672 |  |  |  |  |  |  |  |  |  |  |  |  |  |  |
| 12 | Pb.c000701195.Contig2 | 6384 | + | 1697757 | 1704417 |  |  |  |  |  |  |  |  |  |  |  |  |  |  |
| 12 | Pc.c001002727.Contig1 | 1923 | + | 1700590 | 1702678 | cRMPc14075 | 25222 | 1919 | 1679195 | 1704417 |  |  |  | cRMPs1419 | 68176 | 1919 | 1636241 | 1704417 |  |
| man12 | MALPY02650 | 2830 | - | 1706336 | 1709711 |  |  |  |  |  | 556B |  |  |  |  |  |  |  |  |
| man12 | MALPY00335 | 3444 | + | 1708905 | 1712881 |  |  |  |  |  | 556B |  |  |  |  |  |  |  |  |
| 12 | Pb.c000401759.Contig1 | 1909 | + | 1712829 | 1714765 |  |  |  |  |  |  |  |  |  |  |  |  |  |  |
| 12 | MALPY01581 | 9844 | - | 1713358 | 1723214 |  |  |  |  |  | 416 |  |  |  |  |  |  |  |  |
| 12 | MALPY03293 | 819 | - | 1716752 | 1717565 | cRMPc14076 | 16878 | 1060 | 1706336 | 1723214 |  |  |  |  |  |  |  |  |  |
| man12 | MALPY00186 | 6478 | - | 1724274 | 1731514 |  |  |  |  |  | 416 |  |  |  |  |  |  |  |  |
| 12 | Pc.c000102008.Contig1 | 8508 | - | 1727479 | 1736659 |  |  |  |  |  |  |  |  |  |  |  |  |  |  |
| 12 | Pb.c001602402.Contig1 | 3350 | + | 1730813 | 1734163 |  |  |  |  |  |  |  |  |  |  |  |  |  |  |
| 12 | MALPY01738 | 2267 | + | 1731410 | 1733677 |  |  |  |  |  | 416 |  |  |  |  |  |  |  |  |
| 12 | MALPY02674 | 4692 | - | 1734456 | 1739444 |  |  |  |  |  | 416 |  |  |  |  |  |  |  |  |
| 12 | MALPY03172 | 307 | + | 1735858 | 1736165 |  |  |  |  |  |  |  |  |  |  |  |  |  |  |
| 12 | MALPY03173 | 244 | + | 1735862 | 1736106 |  |  |  |  |  |  | 30 | 8000 |  |  |  |  |  |  |
| 12 | Pb.c000901750.Contig1 | 3428 | - | 1736039 | 1739735 |  |  |  |  |  |  |  |  |  |  |  |  |  |  |
| man12 | PbContig4827 | 3444 | - | 1736288 | 1739732 |  |  |  |  |  |  |  |  |  |  |  |  |  | MALPY02674/MALPY02713 |
| 12 | Pc.c000202813.Contig1 | 3222 | - | 1736733 | 1740158 |  |  |  |  |  |  |  |  |  |  |  |  |  |  |
| man12 | MALPY02713 | 2310 | - | 1739314 | 1741624 |  |  |  |  |  | 416 |  |  |  |  |  |  |  | PbContig4827 |
| 12 | Pb.c000601520.Contig1 | 3212 | + | 1741536 | 1744787 |  |  |  |  |  |  |  |  |  |  |  |  |  |  |
| 12 | MALPY00588 | 5382 | - | 1743914 | 1749965 |  |  |  |  |  |  | 30 | 2200 |  |  |  |  |  |  |
| 12 | Pb.c000201833.Contig1 | 1144 | + | 1747532 | 1748736 | cRMPc14077 | 25691 | 2289 | 1724274 | 1749965 |  |  |  |  |  |  |  |  |  |
| 12 | MALPY00690 | 6656 | - | 1752254 | 1758894 |  |  |  |  |  | 388\_alt | 30 |  |  |  |  |  |  |  |
| 12 | Pb.c001101821.Contig1 | 6239 | - | 1753791 | 1760023 |  |  |  |  |  |  |  |  |  |  |  |  |  |  |
| 12 | MALPY02521 | 7030 | + | 1759847 | 1767735 |  |  |  |  |  | 388\_alt | 382 | 3500 |  |  |  |  |  |  |
| 12 | Pb.c000601523.Contig1 | 4226 | + | 1763017 | 1768061 |  |  |  |  |  |  |  |  |  |  |  |  |  |  |
| 12 | Pc.c001103878.Contig1 | 1969 | + | 1766247 | 1768219 |  |  |  |  |  |  |  |  |  |  |  |  |  |  |
| 12 | Pc.c001104458.Contig1 | 627 | + | 1766375 | 1767008 |  |  |  |  |  |  |  |  |  |  |  |  |  |  |
| 12 | MALPY02152 | 2232 | - | 1767757 | 1769983 |  |  |  |  |  |  |  |  |  |  |  |  |  |  |
| 12 | Pb.c000301438.Contig1 | 3879 | + | 1768025 | 1772365 |  |  |  |  |  |  |  |  |  |  |  |  |  |  |
| 12 | Pc.c000402720.Contig1 | 5834 | + | 1768302 | 1774249 |  |  |  |  |  |  |  |  |  |  |  |  |  |  |
| 12 | MALPY01114 | 13504 | + | 1770574 | 1784212 |  |  |  |  |  | 53 | 382 |  |  |  |  |  |  |  |
| 12 | Pb.c000901839.Contig1 | 4929 | + | 1772535 | 1777503 |  |  |  |  |  |  |  |  |  |  |  |  |  |  |
| 12 | Pc.c001505691.Contig1 | 1332 | + | 1774960 | 1776249 |  |  |  |  |  |  |  |  |  |  |  |  |  |  |
| 12 | Pc.c000303079.Contig1 | 2193 | + | 1776270 | 1778661 |  |  |  |  |  |  |  |  |  |  |  |  |  |  |
| 12 | Pb.c000301486.Contig1 | 7870 | + | 1778024 | 1785955 |  |  |  |  |  |  |  |  |  |  |  |  |  |  |
| 12 | MALPY03327 | 148 | + | 1780528 | 1780676 |  |  |  |  |  |  | 372 | 2500 |  |  |  |  |  |  |
| 12 | Pc.c001708335.Contig1 | 650 | - | 1781093 | 1781743 |  |  |  |  |  |  |  |  |  |  |  |  |  |  |
| 12 | Pc.c000402877.Contig1 | 1560 | - | 1781672 | 1783232 |  |  |  |  |  |  |  |  |  |  |  |  |  |  |
| 12 | MALPY00420 | 10282 | - | 1783036 | 1794130 |  |  |  |  |  | 53 | 372 |  |  |  |  |  |  |  |
| 12 | Pc.c000402283.Contig1 | 3853 | - | 1783404 | 1787466 |  |  |  |  |  |  |  |  |  |  |  |  |  |  |
| 12 | Pb.c001602004.Contig1 | 5912 | + | 1786520 | 1792977 |  |  |  |  |  |  |  |  |  |  |  |  |  |  |
| 12 | Pc.c001304553.Contig1 | 2546 | - | 1793495 | 1796041 |  |  |  |  |  |  |  |  |  |  |  |  |  |  |
| 12 | Pb.c001702761.Contig1 | 2597 | - | 1793851 | 1796448 |  |  |  |  |  |  |  |  |  |  |  |  |  |  |
| 12 | MALPY01663 | 11140 | + | 1794182 | 1805796 |  |  |  |  |  | 53 | 280 | 3500 |  |  |  |  |  |  |
| 12 | PbContig53 | 3509 | + | 1798839 | 1802547 | cRMPc14078 | 53542 | 3500 | 1752254 | 1805796 |  |  |  |  |  |  |  |  |  |
| Centromere - PCR 3500 bp | |  |  | 1800000 |  |  |  |  |  |  |  |  |  |  |  |  |  |  |  |
| 12 | MALPY01956 | 3528 | + | 1809296 | 1812905 |  |  |  |  |  | 595 | 280 |  |  |  |  |  |  |  |
| 12 | Pb.c001602499.Contig1 | 2050 | - | 1810863 | 1813000 |  |  |  |  |  |  |  |  |  |  |  |  |  |  |
| 12 | MALPY01096 | 7978 | + | 1812876 | 1821766 |  |  |  |  |  | 595 | 366 | 1400 |  |  |  |  |  |  |
| 12 | Pb.c001702719.Contig1 | 8477 | - | 1814511 | 1824568 |  |  |  |  |  |  |  |  |  |  |  |  |  |  |
| 12 | Pc.c000803694.Contig1 | 616 | + | 1818888 | 1819504 |  |  |  |  |  |  |  |  |  |  |  |  |  |  |
| 12 | Pc.c000803341.Contig1 | 3021 | + | 1819542 | 1822791 |  |  |  |  |  |  |  |  |  |  |  |  |  |  |
| 12 | MALPY02687 | 9773 | + | 1822584 | 1832146 |  |  |  |  |  |  | 366 |  |  |  |  |  |  |  |
| 12 | MALPY03211 | 503 | - | 1824028 | 1824531 |  |  |  |  |  |  |  |  |  |  |  |  |  |  |
| 12 | Pb.c002007039.Contig1 | 2573 | - | 1824491 | 1827166 |  |  |  |  |  |  |  |  |  |  |  |  |  |  |
| 12 | MALPY03269 | 274 | + | 1825644 | 1825921 | cRMPc14079 | 22850 | 5667 | 1809296 | 1832146 |  |  |  | cRMPs1420 | 125810 | 5667 | 1706336 | 1832146 |  |
| 12 | MALPY02010 | 8651 | + | 1837813 | 1846329 |  |  |  |  |  | 930 | 191 | 2600 |  |  |  |  |  |  |
| 12 | Pb.c001101989.Contig1 | 7630 | - | 1838209 | 1845710 |  |  |  |  |  |  |  |  |  |  |  |  |  |  |
| 12 | Pc.c001505095.Contig1 | 1933 | + | 1841010 | 1842892 | cRMPc14080 | 8516 | 137 | 1837813 | 1846329 |  |  |  |  |  |  |  |  |  |
| man12 | MALPY02011 | 2574 | + | 1846466 | 1848296 | cRMPc14081 | 1830 | 1305 | 1846466 | 1848296 | 930 |  |  |  |  |  |  |  |  |
| 12 | MALPY01467 | 4842 | + | 1849601 | 1854737 |  |  |  |  |  |  | 191 |  |  |  |  |  |  |  |
| 12 | Pb.c000401430.Contig1 | 5019 | - | 1851127 | 1856588 |  |  |  |  |  |  |  |  |  |  |  |  |  |  |
| 12 | Pc.c000702658.Contig2 | 2179 | + | 1852626 | 1855259 |  |  |  |  |  |  |  |  |  |  |  |  |  |  |
| 12 | MALPY01482 | 7932 | + | 1852937 | 1861374 |  |  |  |  |  | 429 |  |  |  |  |  |  |  |  |
| 12 | Pc.c000602342.Contig2 | 2521 | + | 1855289 | 1858145 |  |  |  |  |  |  |  |  |  |  |  |  |  |  |
| 12 | Pc.c001404689.Contig1 | 2594 | - | 1856570 | 1859155 |  |  |  |  |  |  |  |  |  |  |  |  |  |  |
| 12 | PbContig216 | 7087 | - | 1856938 | 1864349 |  |  |  |  |  |  |  |  |  |  |  |  |  |  |
| 12 | MALPY01553 | 3241 | - | 1861811 | 1865140 |  |  |  |  |  | 429 | 142 | 2500 |  |  |  |  |  |  |
| 12 | Pc.c001708290.Contig1 | 1381 | + | 1862581 | 1863962 |  |  |  |  |  |  |  |  |  |  |  |  |  |  |
| 12 | Pc.c001813199.Contig1 | 627 | + | 1864527 | 1865154 | cRMPc14082 | 15553 | 3046 | 1849601 | 1865154 |  |  |  |  |  |  |  |  |  |
| 12 | MALPY01447 | 24554 | + | 1868200 | 1894985 |  |  |  |  |  |  | 142 | 3200 |  |  |  |  |  |  |
| 12 | Pc.c001204040.Contig1 | 5244 | - | 1869544 | 1875005 |  |  |  |  |  |  |  |  |  |  |  |  |  |  |
| 12 | Pb.c001904372.Contig1 | 4588 | + | 1870763 | 1875351 |  |  |  |  |  |  |  |  |  |  |  |  |  |  |
| 12 | Pc.c000602935.Contig1 | 7727 | - | 1876597 | 1884323 |  |  |  |  |  |  |  |  |  |  |  |  |  |  |
| 12 | PbContig69 | 6598 | + | 1878402 | 1884922 |  |  |  |  |  |  |  |  |  |  |  |  |  |  |
| 12 | PcContig644 | 4149 | - | 1887095 | 1891247 | cRMPc14083 | 26785 | 3832 | 1868200 | 1894985 |  |  |  |  |  |  |  |  |  |
| 12 | MALPY01541 | 9007 | - | 1898817 | 1908034 |  |  |  |  |  |  | 142 | 500 |  |  |  |  |  |  |
| 12 | Pb.c000101286.Contig1 | 5733 | - | 1900027 | 1906024 |  |  |  |  |  |  |  |  |  |  |  |  |  |  |
| 12 | Pc.c000903508.Contig1 | 3708 | - | 1901455 | 1905379 | cRMPc14084 | 9217 | 866 | 1898817 | 1908034 |  |  |  |  |  |  |  |  |  |
| 12 | MALPY01136 | 10247 | - | 1908900 | 1920558 |  |  |  |  |  |  | 142 | 500 |  |  |  |  |  |  |
| 12 | Pc.c000803392.Contig1 | 2783 | - | 1908988 | 1912041 |  |  |  |  |  |  |  |  |  |  |  |  |  |  |
| 12 | Pb.c001101519.Contig1 | 8288 | - | 1909329 | 1918273 |  |  |  |  |  |  |  |  |  |  |  |  |  |  |
| 12 | Pc.c000803393.Contig1 | 5375 | - | 1912217 | 1918447 |  |  |  |  |  |  |  |  |  |  |  |  |  |  |
| 12 | Pc.c000603422.Contig1 | 623 | - | 1920529 | 1921152 |  |  |  |  |  |  |  |  |  |  |  |  |  |  |
| 12 | MALPY03567 | 478 | - | 1920617 | 1921095 | cRMPc14085 | 12252 | 838 | 1908900 | 1921152 |  | 142 | 2000 |  |  |  |  |  |  |
| 12 | PcContig386 | 7692 | + | 1921990 | 1930230 |  |  |  |  |  |  |  |  |  |  |  |  |  |  |
| 12 | Pb.c001601978.Contig1 | 4889 | - | 1922573 | 1927458 |  |  |  |  |  |  |  |  |  |  |  |  |  |  |
| 12 | MALPY00357 | 4407 | - | 1923532 | 1927682 |  |  |  |  |  |  | 142 |  |  |  |  |  |  |  |
| 12 | MALPY03259 | 1393 | - | 1925263 | 1926396 |  |  |  |  |  |  |  |  |  |  |  |  |  |  |
| 12 | MALPY03320 | 339 | + | 1925754 | 1926093 |  |  |  |  |  |  | 448 | 2300 |  |  |  |  |  |  |
| 12 | MALPY02962 | 2043 | - | 1928286 | 1930369 |  |  |  |  |  | 1123 | 448 |  |  |  |  |  |  |  |
| 12 | Pb.c001702892.Contig1 | 1647 | - | 1929443 | 1931046 |  |  |  |  |  |  |  |  |  |  |  |  |  |  |
| 12 | MALPY00657 | 8022 | - | 1929700 | 1936971 |  |  |  |  |  | 1123 | 380 | 2200 |  |  |  |  |  |  |
| 12 | PcContig574 | 4011 | + | 1933684 | 1937862 |  |  |  |  |  |  |  |  |  |  |  |  |  |  |
| 12 | Pb.c000501457.Contig1 | 1067 | - | 1934111 | 1935223 | cRMPc14086 | 15872 | 2318 | 1921990 | 1937862 |  |  |  |  |  |  |  |  |  |
| 12 | MALPY01108 | 13118 | - | 1940180 | 1953850 |  |  |  |  |  | 1316 | 380 |  |  |  |  |  |  |  |
| 12 | Pc.c000803294.Contig1 | 4005 | + | 1941659 | 1945655 |  |  |  |  |  |  |  |  |  |  |  |  |  |  |
| 12 | Pb.c001201991.Contig1 | 3028 | - | 1945946 | 1948950 |  |  |  |  |  |  |  |  |  |  |  |  |  |  |
| 12 | PcContig708 | 12009 | - | 1948452 | 1962000 |  |  |  |  |  |  |  |  |  |  |  |  |  |  |
| 12 | Pb.c001702754.Contig1 | 2928 | - | 1949586 | 1952512 |  |  |  |  |  |  |  |  |  |  |  |  |  |  |
| 12 | MALPY02078 | 12112 | + | 1954861 | 1967324 |  |  |  |  |  | 1316 | 82 | 1000 |  |  |  |  |  |  |
| 12 | Pb.c001904349.Contig1 | 1058 | - | 1956878 | 1958226 |  |  |  |  |  |  |  |  |  |  |  |  |  |  |
| 12 | PbContig167 | 4965 | - | 1958248 | 1963356 |  |  |  |  |  |  |  |  |  |  |  |  |  |  |
| 12 | MALPY01018 | 13006 | - | 1966759 | 1981926 |  |  |  |  |  |  | 82 |  |  |  |  |  |  |  |
| 12 | PbContig306 | 2208 | - | 1966990 | 1969222 |  |  |  |  |  |  |  |  |  |  |  |  |  |  |
| 12 | Pc.c000303075.Contig1 | 1178 | + | 1967674 | 1968849 |  |  |  |  |  |  |  |  |  |  |  |  |  |  |
| 12 | Pb.c001401714.Contig2 | 12639 | - | 1970917 | 1983740 |  |  |  |  |  |  |  |  |  |  |  |  |  |  |
| 12 | Pc.c000803343.Contig1 | 6257 | - | 1972201 | 1978524 |  |  |  |  |  |  |  |  |  |  |  |  |  |  |
| 12 | PcContig255 | 12437 | - | 1979663 | 1992563 |  |  |  |  |  |  |  |  |  |  |  |  |  |  |
| 12 | MALPY03116 | 1328 | - | 1979731 | 1980961 |  |  |  |  |  |  | 429 | 2500 |  |  |  |  |  |  |
| 12 | MALPY00142 | 4405 | + | 1983075 | 1987638 |  |  |  |  |  | 9 | 429 |  |  |  |  |  |  |  |
| 12 | Pb.c001102207.Contig1 | 1526 | - | 1983646 | 1985175 |  |  |  |  |  |  |  |  |  |  |  |  |  |  |
| 12 | PbContig226 | 11927 | - | 1985502 | 1998098 |  |  |  |  |  |  |  |  |  |  |  |  |  |  |
| 12 | MALPY00143 | 13870 | - | 1987262 | 2002426 |  |  |  |  |  | 9 |  |  |  |  |  |  |  |  |
| 12 | MALPY03397 | 378 | - | 1988563 | 1988941 |  |  |  |  |  |  |  |  |  |  |  |  |  |  |
| 12 | Pc.c001605009.Contig1 | 1673 | - | 1995404 | 1997305 |  |  |  |  |  |  |  |  |  |  |  |  |  |  |
| 12 | PcContig349 | 7154 | - | 1998161 | 2005867 |  |  |  |  |  |  |  |  |  |  |  |  |  |  |
| 12 | Pb.c000501485.Contig1 | 3281 | - | 1998797 | 2002306 |  |  |  |  |  |  |  |  |  |  |  |  |  |  |
| 12 | MALPY00260 | 15631 | + | 2002780 | 2017309 |  |  |  |  |  | 9 |  |  |  |  |  |  |  |  |
| 12 | Pb.c002113245.Contig1 | 1194 | - | 2003169 | 2004381 |  |  |  |  |  |  |  |  |  |  |  |  |  |  |
| 12 | PbContig192 | 8046 | + | 2004571 | 2012259 |  |  |  |  |  |  |  |  |  |  |  |  |  |  |
| 12 | Pc.c001003475.Contig1 | 4236 | - | 2005603 | 2009984 |  |  |  |  |  |  |  |  |  |  |  |  |  |  |
| 12 | MALPY03313 | 1809 | + | 2010812 | 2012666 |  |  |  |  |  |  |  |  |  |  |  |  |  |  |
| 12 | MALPY03283 | 1000 | + | 2011891 | 2012936 |  |  |  |  |  |  |  |  |  |  |  |  |  |  |
| 12 | Pb.c001402475.Contig1 | 1605 | + | 2012519 | 2014124 |  |  |  |  |  |  |  |  |  |  |  |  |  |  |
| 12 | MALPY03236 | 190 | - | 2012819 | 2012997 |  |  |  |  |  |  | 241 | 4000 |  |  |  |  |  |  |
| 12 | PcContig492 | 8601 | + | 2012877 | 2022096 |  |  |  |  |  |  |  |  |  |  |  |  |  |  |
| 12 | Pb.c000201260.Contig1 | 6612 | + | 2014278 | 2021363 |  |  |  |  |  |  |  |  |  |  |  |  |  |  |
| 12 | MALPY00261 | 7897 | + | 2017787 | 2025012 |  |  |  |  |  | 9 | 241 |  |  |  |  |  |  |  |
| 12 | Pb.c000901771.Contig1 | 6928 | - | 2022121 | 2029519 |  |  |  |  |  |  |  |  |  |  |  |  |  |  |
| 12 | Pc.c001606800.Contig1 | 654 | - | 2024216 | 2024867 |  |  |  |  |  |  |  |  |  |  |  |  |  |  |
| 12 | MALPY01272 | 2453 | - | 2025305 | 2027758 |  |  |  |  |  | 9 |  |  |  |  |  |  |  |  |
| 12 | PcContig203 | 2464 | - | 2025349 | 2027813 | cRMPc14087 | 89339 | 303 | 1940180 | 2029519 |  |  |  | cRMPs1421 | 191706 | 303 | 1837813 | 2029519 |  |
| 12 | MALPY00901 | 3325 | - | 2029822 | 2033249 |  |  |  |  |  |  |  |  |  |  |  |  |  |  |
| 12 | PcContig258 | 4090 | - | 2030067 | 2034304 |  |  |  |  |  |  |  |  |  |  |  |  |  |  |
| 12 | Pb.c000902061.Contig1 | 604 | - | 2030919 | 2031523 |  |  |  |  |  |  |  |  |  |  |  |  |  |  |
| 12 | Pb.c000901770.Contig1 | 1742 | - | 2031503 | 2033389 | cRMPc14088 | 4482 | 8939 | 2029822 | 2034304 |  |  |  | cRMPs1422 | 4482 | 8939 | 2029822 | 2034304 |  |
| 12 | MALPY02043 | 7135 | - | 2043243 | 2050753 |  |  |  |  |  | 492 |  |  |  |  |  |  |  |  |
| 12 | Pb.c001904453.Contig1 | 2531 | + | 2043371 | 2045899 |  |  |  |  |  |  |  |  |  |  |  |  |  |  |
| 12 | Pb.c002007490.Contig1 | 1068 | + | 2049071 | 2050142 |  |  |  |  |  |  |  |  |  |  |  |  |  |  |
| 12 | Pb.c001803330.Contig1 | 2165 | + | 2050116 | 2052281 |  |  |  |  |  |  |  |  |  |  |  |  |  |  |
| 12 | MALPY01778 | 16008 | - | 2050600 | 2067275 |  |  |  |  |  | 492 |  |  |  |  |  |  |  |  |
| 12 | Pb.c000701577.Contig1 | 2476 | + | 2052361 | 2054752 |  |  |  |  |  |  |  |  |  |  |  |  |  |  |
| 12 | Pc.c000802642.Contig2 | 7300 | + | 2052638 | 2059848 |  |  |  |  |  |  |  |  |  |  |  |  |  |  |
| 12 | Pb.c002005880.Contig1 | 2638 | + | 2061744 | 2064491 |  |  |  |  |  |  |  |  |  |  |  |  |  |  |
| 12 | Pc.c001812078.Contig1 | 1517 | - | 2062309 | 2063866 |  |  |  |  |  |  |  |  |  |  |  |  |  |  |
| 12 | Pc.c000302379.Contig3 | 11226 | - | 2066296 | 2077522 |  |  |  |  |  |  |  |  |  |  |  |  |  |  |
| man12 | MALPY02745 | 3564 | - | 2068746 | 2073474 |  |  |  |  |  | 492 |  |  |  |  |  |  |  |  |
| JC12 | MALPY00085 | 5906 | xxx | 2070000 |  |  |  |  |  |  | 108 |  |  |  |  |  |  |  |  |
| man12 | MALPY02392 | 2161 | + | 2073715 | 2075816 |  |  |  |  |  | 108 |  |  |  |  |  |  |  |  |
| man12 | MALPY01929 | 3254 | + | 2076293 | 2080423 |  |  |  |  |  | 108 |  |  |  |  |  |  |  |  |
| 12 | MALPY01340 | 5130 | + | 2079973 | 2085251 |  |  |  |  |  | 108 |  |  |  |  |  |  |  |  |
| 12 | Pb.c000801739.Contig1 | 3106 | + | 2081284 | 2084861 |  |  |  |  |  |  |  |  |  |  |  |  |  |  |
| 12 | Pc.c000202160.Contig2 | 4534 | + | 2081919 | 2086590 |  |  |  |  |  |  |  |  |  |  |  |  |  |  |
| man12 | MALPY01724 | 5158 | + | 2085796 | 2091940 |  |  |  |  |  | 124 |  |  |  |  |  |  |  |  |
| 12 | MALPY01048 | 6201 | + | 2091122 | 2098049 |  |  |  |  |  | 124 |  |  |  |  |  |  |  |  |
| 12 | PbContig61 | 4263 | - | 2091384 | 2095647 |  |  |  |  |  |  |  |  |  |  |  |  |  |  |
| 12 | Pc.c001304754.Contig1 | 823 | + | 2093132 | 2093958 |  |  |  |  |  |  |  |  |  |  |  |  |  |  |
| 12 | Pc.c001707691.Contig1 | 2340 | - | 2095747 | 2098108 |  |  |  |  |  |  |  |  |  |  |  |  |  |  |
| 12 | PbContig200 | 19567 | + | 2097373 | 2121577 |  |  |  |  |  |  |  |  |  |  |  |  |  |  |
| 12 | MALPY01999 | 2873 | - | 2098616 | 2101346 |  |  |  |  |  | 124 |  |  |  |  |  |  |  |  |
| 12 | MALPY01495 | 12793 | - | 2101276 | 2118731 |  |  |  |  |  | 124 | 222 | 3500 |  |  |  |  |  |  |
| 12 | Pc.c000103059.Contig1 | 1747 | + | 2107486 | 2109230 |  |  |  |  |  |  |  |  |  |  |  |  |  |  |
| 12 | PcContig81 | 3772 | + | 2113575 | 2117418 |  |  |  |  |  |  |  |  |  |  |  |  |  |  |
| 12 | Pc.c000303031.Contig1 | 5795 | - | 2119761 | 2125520 |  |  |  |  |  |  |  |  |  |  |  |  |  |  |
| 12 | Pb.c001301444.Contig1 | 5304 | - | 2123139 | 2128659 |  |  |  |  |  |  |  |  |  |  |  |  |  |  |
| 12 | MALPY02422 | 4686 | + | 2124087 | 2128767 | cRMPc14089 | 85524 | 1157 | 2043243 | 2128767 |  | 222 | 2200 |  |  |  |  |  |  |
| 12 | MALPY01968 | 2459 | - | 2129924 | 2132458 |  |  |  |  |  |  | 222 | 2000 |  |  |  |  |  |  |
| 12 | PcContig726 | 2425 | + | 2129927 | 2132472 | cRMPc14090 | 2548 | 1010 | 2129924 | 2132472 |  |  |  |  |  |  |  |  |  |
| 12 | Pb.c000201576.Contig1 | 3775 | - | 2133482 | 2137365 |  |  |  |  |  |  |  |  |  |  |  |  |  |  |
| 12 | MALPY00212 | 7670 | - | 2133859 | 2142602 |  |  |  |  |  |  | 222 |  |  |  |  |  |  |  |
| 12 | Pc.c001505384.Contig1 | 1228 | - | 2135057 | 2136447 |  |  |  |  |  |  |  |  |  |  |  |  |  |  |
| 12 | PcContig775 | 4989 | - | 2136467 | 2142590 |  |  |  |  |  |  |  |  |  |  |  |  |  |  |
| 12 | Pb.c001702367.Contig1 | 3663 | - | 2138518 | 2142196 |  |  |  |  |  |  |  |  |  |  |  |  |  |  |
| 12 | MALPY03413 | 354 | - | 2140218 | 2140572 | cRMPc14091 | 9120 | 1114 | 2133482 | 2142602 |  | 289 | 3000 |  |  |  |  |  |  |
| 12 | PbContig399 | 8111 | - | 2143716 | 2153246 |  |  |  |  |  |  |  |  |  |  |  |  |  |  |
| 12 | Pc.c000203091.Contig1 | 2732 | - | 2143776 | 2146509 |  |  |  |  |  |  |  |  |  |  |  |  |  |  |
| 12 | MALPY01923 | 4079 | - | 2144025 | 2148092 |  |  |  |  |  | 369 | 289 |  |  |  |  |  |  |  |
| 12 | Pc.c001812722.Contig1 | 622 | - | 2146787 | 2147400 |  |  |  |  |  |  |  |  |  |  |  |  |  |  |
| 12 | MALPY00818 | 3198 | + | 2149322 | 2152701 |  |  |  |  |  | 369 | 418 | 900 |  |  |  |  |  |  |
| 12 | Pc.c000503082.Contig1 | 3329 | + | 2149557 | 2153060 | cRMPc14092 | 9530 | 8323 | 2143716 | 2153246 |  |  |  |  |  |  |  |  |  |
| 12 | Pb.c001202102.Contig1 | 8013 | + | 2161569 | 2170761 |  |  |  |  |  |  |  |  |  |  |  |  |  |  |
| 12 | MALPY02364 | 4918 | - | 2163309 | 2168395 |  |  |  |  |  |  | 418 | 3000 |  |  |  |  |  |  |
| 12 | Pc.c000202712.Contig1 | 3294 | - | 2164620 | 2168157 |  |  |  |  |  |  |  |  |  |  |  |  |  |  |
| 12 | Pb.c001703173.Contig1 | 592 | + | 2166531 | 2167123 |  |  |  |  |  |  |  |  |  |  |  |  |  |  |
| 12 | MALPY03331 | 846 | + | 2169281 | 2170109 | cRMPc14093 | 9192 | 532 | 2161569 | 2170761 |  | 42 | 1800 |  |  |  |  |  |  |
| 12 | MALPY01210 | 5591 | - | 2171293 | 2178391 |  |  |  |  |  |  | 42 |  |  |  |  |  |  |  |
| 12 | Pb.c001803298.Contig1 | 9207 | + | 2171909 | 2182581 |  |  |  |  |  |  |  |  |  |  |  |  |  |  |
| 12 | PcContig677 | 4348 | + | 2172358 | 2176928 |  |  |  |  |  |  |  |  |  |  |  |  |  |  |
| 12 | Pc.c000903843.Contig1 | 684 | + | 2173885 | 2174731 |  |  |  |  |  |  |  |  |  |  |  |  |  |  |
| 12 | MALPY03505 | 780 | - | 2174534 | 2175380 |  |  |  |  |  |  |  |  |  |  |  |  |  |  |
| 12 | Pc.c000903355.Contig1 | 2163 | - | 2174780 | 2176955 |  |  |  |  |  |  |  |  |  |  |  |  |  |  |
| 12 | MALPY03287 | 1082 | - | 2174794 | 2175879 |  |  |  |  |  |  |  |  |  |  |  |  |  |  |
| 12 | MALPY01591 | 4757 | + | 2178409 | 2183253 |  |  |  |  |  |  |  |  |  |  |  |  |  |  |
| 12 | Pc.c000502436.Contig1 | 3920 | - | 2178860 | 2182867 |  |  |  |  |  |  |  |  |  |  |  |  |  |  |
| 12 | MALPY03021 | 647 | - | 2180959 | 2181584 | cRMPc14094 | 11960 | 1984 | 2171293 | 2183253 |  | 86 | 3500 |  |  |  |  |  |  |
| 12 | MALPY00969 | 6364 | + | 2185237 | 2191748 |  |  |  |  |  |  | 86 |  |  |  |  |  |  |  |
| 12 | Pb.c001703214.Contig1 | 777 | + | 2188084 | 2188861 |  |  |  |  |  |  |  |  |  |  |  |  |  |  |
| 12 | Pb.c001202119.Contig1 | 3095 | + | 2189288 | 2192413 |  |  |  |  |  |  |  |  |  |  |  |  |  |  |
| 12 | MALPY03349 | 799 | + | 2190109 | 2190905 | cRMPc14095 | 7176 | 1180 | 2185237 | 2192413 |  |  |  | cRMPs1423 | 149170 | 1180 | 2043243 | 2192413 |  |
| man12 | MALPY00819 | 3358 | + | 2193593 | 2196957 |  |  |  |  |  | 44 |  |  |  |  |  |  |  |  |
| 12 | MALPY01671 | 5009 | + | 2195932 | 2200851 |  |  |  |  |  | 44 |  |  |  |  |  |  |  |  |
| 12 | PbContig239 | 12291 | + | 2196031 | 2208152 |  |  |  |  |  |  |  |  |  |  |  |  |  |  |
| man12 | MALPY00610 - Ins | 19346 | + | 2200279 | 2219033 |  |  |  |  |  | 44 | 351 | 3000 |  |  |  |  |  |  |
| 12 | Pc.c000402856.Contig1 | 1502 | - | 2201628 | 2203109 |  |  |  |  |  |  |  |  |  |  |  |  |  |  |
| 12 | Pc.c000803396.Contig1 | 1427 | - | 2202853 | 2204394 |  |  |  |  |  |  |  |  |  |  |  |  |  |  |
| 12 | Pc.c000303506.Contig1 | 899 | - | 2204500 | 2205489 |  |  |  |  |  |  |  |  |  |  |  |  |  |  |
| 12 | Pb.c000301155.Contig1 | 5506 | - | 2208096 | 2212978 |  |  |  |  |  |  |  |  |  |  |  |  |  |  |
| 12 | Pc.c000202691.Contig1 | 4848 | + | 2208433 | 2212479 |  |  |  |  |  |  |  |  |  |  |  |  |  |  |
| 12 | MALPY03393 | 1353 | + | 2208756 | 2210081 |  |  |  |  |  |  |  |  |  |  |  |  |  |  |
| 12 | MALPY03168 | 471 | - | 2209194 | 2209644 |  |  |  |  |  |  | 351 | 6500 |  |  |  |  |  |  |
| 12 | MALPY03465 | 846 | + | 2211549 | 2212329 |  |  |  |  |  |  | 351 |  |  |  |  |  |  |  |
| 12 | Pb.c001602361.Contig1 | 4105 | + | 2213276 | 2217625 |  |  |  |  |  |  |  |  |  |  |  |  |  |  |
| 12 | Pc.c001606307.Contig1 | 1104 | - | 2216721 | 2217825 | cRMPc14096 | 25440 | 31 | 2193593 | 2219033 |  |  |  | cRMPs1424 | 25440 | 31 | 2193593 | 2219033 |  |
| 12 | MALPY01000 | 11093 | + | 2219064 | 2230184 |  |  |  |  |  |  |  |  |  |  |  |  |  |  |
| 12 | Pb.c002006803.Contig1 | 2458 | - | 2219703 | 2222089 |  |  |  |  |  |  |  |  |  |  |  |  |  |  |
| 12 | MALPY03511 | 859 | - | 2220474 | 2221264 |  |  |  |  |  |  |  |  |  |  |  |  |  |  |
| 12 | Pc.c001813156.Contig1 | 718 | - | 2220501 | 2221159 |  |  |  |  |  |  |  |  |  |  |  |  |  |  |
| 12 | Pb.c001001661.Contig1 | 5641 | - | 2224863 | 2230513 |  |  |  |  |  |  |  |  |  |  |  |  |  |  |
| 12 | Pc.c001504946.Contig1 | 1104 | - | 2225701 | 2226820 | cRMPc14097 | 11449 | 3422 | 2219064 | 2230513 |  |  |  | cRMPs1425 | 11449 | 3422 | 2219064 | 2230513 |  |
| 12 | MALPY00398 | 26406 | - | 2233935 | 2262022 |  |  |  |  |  |  |  |  |  |  |  |  |  |  |
| 12 | Pb.c001402142.Contig1 | 21022 | - | 2235306 | 2257757 |  |  |  |  |  |  |  |  |  |  |  |  |  |  |
| 12 | PcContig107 | 2028 | - | 2237123 | 2239148 |  |  |  |  |  |  |  |  |  |  |  |  |  |  |
| 12 | PcContig769 | 6032 | - | 2240286 | 2246466 |  |  |  |  |  |  |  |  |  |  |  |  |  |  |
| 12 | MALPY03510 | 539 | - | 2244574 | 2245113 |  |  |  |  |  |  |  |  |  |  |  |  |  |  |
| 12 | PcContig389 | 7790 | - | 2247445 | 2255721 |  |  |  |  |  |  |  |  |  |  |  |  |  |  |
| 12 | Pb.c000501179.Contig2 | 10278 | - | 2259185 | 2269805 |  |  |  |  |  |  |  |  |  |  |  |  |  |  |
| 12 | MALPY01233 | 11394 | - | 2260741 | 2272840 |  |  |  |  |  | 364 |  |  |  |  |  |  |  |  |
| 12 | Pc.c001505098.Contig1 | 1553 | - | 2263053 | 2264594 |  |  |  |  |  |  |  |  |  |  |  |  |  |  |
| 12 | MALPY03319 | 249 | - | 2267900 | 2268149 |  |  |  |  |  |  | 293 | 1000 |  |  |  |  |  |  |
| 12 | MALPY03270 | 207 | - | 2268907 | 2269117 |  |  |  |  |  |  | 293 | 3500 |  |  |  |  |  |  |
| 12 | Pb.c000501482.Contig1 | 6474 | - | 2270215 | 2277021 |  |  |  |  |  |  |  |  |  |  |  |  |  |  |
| 12 | Pc.c001505638.Contig1 | 692 | + | 2271620 | 2272312 |  |  |  |  |  |  |  |  |  |  |  |  |  |  |
| 12 | MALPY01039 | 3917 | + | 2273195 | 2277183 |  |  |  |  |  | 364 | 293 |  |  |  |  |  |  |  |
| 12 | Pc.c001204426.Contig1 | 1596 | + | 2274732 | 2276259 |  |  |  |  |  |  |  |  |  |  |  |  |  |  |
| man12 | MALPY00333 - Ins | 7446 | - | 2277042 | 2284436 |  |  |  |  |  | 364 | 479 | 1200 |  |  |  |  |  |  |
| 12 | PbContig5 | 5802 | - | 2278028 | 2283774 | cRMPc14098 | 50501 | 99 | 2233935 | 2284436 |  |  |  |  |  |  |  |  |  |
| 12 | Pb.c000601461.Contig1 | 5549 | - | 2284535 | 2291352 |  |  |  |  |  |  |  |  |  |  |  |  |  |  |
| 12 | MALPY00318 | 20734 | + | 2285241 | 2308840 |  |  |  |  |  | 1361 | 479 |  |  |  |  |  |  |  |
| 12 | Pc.c000702680.Contig2 | 7283 | - | 2288749 | 2296052 |  |  |  |  |  |  |  |  |  |  |  |  |  |  |
| JC12 | MALPY03333 | 581 | - | 2289690 | 2290268 |  |  |  |  |  |  | 349 | 2000 |  |  |  |  |  |  |
| 12 | MALPY03464 | 756 | - | 2291997 | 2292725 |  |  |  |  |  |  | 349 |  |  |  |  |  |  |  |
| 12 | Pb.c002005836.Contig2 | 9731 | + | 2296749 | 2306465 |  |  |  |  |  |  |  |  |  |  |  |  |  |  |
| 12 | Pc.c001706596.Contig2 | 2793 | - | 2297840 | 2300514 |  |  |  |  |  |  |  |  |  |  |  |  |  |  |
| 12 | PcContig170 | 3136 | - | 2302128 | 2305424 |  |  |  |  |  |  |  |  |  |  |  |  |  |  |
| 12 | MALPY03288 | 1028 | + | 2304075 | 2305103 |  |  |  |  |  |  |  |  |  |  |  |  |  |  |
| 12 | Pc.c001304228.Contig1 | 1725 | - | 2306415 | 2308107 | cRMPc14099 | 24305 | 68 | 2284535 | 2308840 |  |  |  |  |  |  |  |  |  |
| 12 | MALPY01991 | 7692 | + | 2308908 | 2317048 |  |  |  |  |  | 1361 | 110 | 3200 |  |  |  |  |  |  |
| 12 | Pc.c000202778.Contig1 | 5101 | - | 2310960 | 2316127 |  |  |  |  |  |  |  |  |  |  |  |  |  |  |
| 12 | Pb.c000301478.Contig1 | 5138 | - | 2311224 | 2316407 | cRMPc14100 | 8140 | 259 | 2308908 | 2317048 |  |  |  |  |  |  |  |  |  |
| 12 | Pb.c001301888.Contig1 | 1181 | - | 2317307 | 2318488 | cRMPc14101 | 1181 | 98 | 2317307 | 2318488 |  |  |  |  |  |  |  |  |  |
| 12 | Pb.c002007051.Contig1 | 1841 | - | 2318586 | 2320535 |  |  |  |  |  |  |  |  |  |  |  |  |  |  |
| 12 | MALPY02234 | 2121 | - | 2319684 | 2321880 | cRMPc14102 | 3294 | 469 | 2318586 | 2321880 | 264A | 110 |  |  |  |  |  |  |  |
| 12 | MALPY02172 | 4976 | - | 2322349 | 2327313 |  |  |  |  |  | 264A |  |  |  |  |  |  |  |  |
| 12 | Pb.c000901714.Contig1 | 8354 | - | 2324012 | 2332690 |  |  |  |  |  |  |  |  |  |  |  |  |  |  |
| 12 | Pc.c000102696.Contig1 | 4345 | + | 2324506 | 2328806 |  |  |  |  |  |  |  |  |  |  |  |  |  |  |
| 12 | MALPY01134 | 2767 | - | 2327679 | 2330419 |  |  |  |  |  | 264A |  |  |  |  |  |  |  |  |
| 12 | Pc.c001304281.Contig1 | 1380 | + | 2329143 | 2330523 |  |  |  |  |  |  |  |  |  |  |  |  |  |  |
| 12 | MALPY00266 | 6088 | + | 2330834 | 2336898 |  |  |  |  |  | 264A | 348 | 5000 |  |  |  |  |  |  |
| 12 | Pb.c002112294.Contig1 | 999 | - | 2332859 | 2333891 |  |  |  |  |  |  |  |  |  |  |  |  |  |  |
| 12 | PcContig532 | 2059 | + | 2333255 | 2335342 |  |  |  |  |  |  |  |  |  |  |  |  |  |  |
| 12 | Pb.c001803536.Contig1 | 1613 | - | 2334004 | 2335650 | cRMPc14103 | 14549 | 3959 | 2322349 | 2336898 |  |  |  |  |  |  |  |  |  |
| 12 | Pc.c000703477.Contig1 | 6166 | - | 2340857 | 2347983 |  |  |  |  |  |  |  |  |  |  |  |  |  |  |
| 12 | MALPY02661 | 2429 | + | 2341578 | 2344007 |  |  |  |  |  | 60B | 348 |  |  |  |  |  |  |  |
| 12 | MALPY00902 | 5343 | + | 2343021 | 2349255 |  |  |  |  |  | 60B | 98 | 1100 |  |  |  |  |  |  |
| 12 | Pb.c001802806.Contig1 | 4258 | - | 2343531 | 2348674 |  |  |  |  |  |  |  |  |  |  |  |  |  |  |
| 12 | MALPY03535 | 828 | + | 2343849 | 2344671 | cRMPc14104 | 8398 | 3246 | 2340857 | 2349255 |  | 98 | 6000 |  |  |  |  |  |  |
| 12 | MALPY01491 | 3186 | - | 2352501 | 2356073 |  |  |  |  |  |  | 98 | 4000 |  |  |  |  |  |  |
| 12 | Pc.c000102678.Contig1 | 1442 | - | 2353094 | 2354536 |  |  |  |  |  |  |  |  |  |  |  |  |  |  |
| 12 | Pb.c001802804.Contig1 | 2986 | + | 2354094 | 2357080 |  |  |  |  |  |  |  |  |  |  |  |  |  |  |
| 12 | PcContig39 | 5501 | + | 2355205 | 2360706 |  |  |  |  |  |  |  |  |  |  |  |  |  |  |
| 12 | MALPY02229 | 7506 | - | 2359308 | 2367222 |  |  |  |  |  | 1280 | 98 |  |  |  |  |  |  |  |
| 12 | Pc.c001812752.Contig1 | 632 | + | 2361064 | 2361642 |  |  |  |  |  |  |  |  |  |  |  |  |  |  |
| 12 | Pc.c001813060.Contig1 | 621 | + | 2363435 | 2364020 |  |  |  |  |  |  |  |  |  |  |  |  |  |  |
| 12 | Pb.c001702324.Contig1 | 3566 | + | 2363467 | 2367087 |  |  |  |  |  |  |  |  |  |  |  |  |  |  |
| 12 | Pc.c001707844.Contig1 | 1983 | + | 2366817 | 2368788 |  |  |  |  |  |  |  |  |  |  |  |  |  |  |
| 12 | MALPY02956 | 2439 | + | 2367712 | 2370125 |  |  |  |  |  | 1280 | 459 | 900 |  |  |  |  |  |  |
| 12 | Pb.c000701550.Contig1 | 6769 | - | 2367769 | 2374997 |  |  |  |  |  |  |  |  |  |  |  |  |  |  |
| 12 | PcContig781 | 3613 | + | 2370585 | 2374186 |  |  |  |  |  |  |  |  |  |  |  |  |  |  |
| 12 | MALPY01743 | 7621 | - | 2370614 | 2378332 |  |  |  |  |  | 1103 | 459 |  |  |  |  |  |  |  |
| 12 | Pb.c001904375.Contig1 | 3093 | - | 2375695 | 2378710 |  |  |  |  |  |  |  |  |  |  |  |  |  |  |
| 12 | Pc.c000602923.Contig1 | 2635 | - | 2376941 | 2380672 |  |  |  |  |  |  |  |  |  |  |  |  |  |  |
| 12 | MALPY00615 | 9887 | + | 2379285 | 2390177 |  |  |  |  |  | 1103 |  |  |  |  |  |  |  |  |
| 12 | Pb.c000301180.Contig1 | 1908 | - | 2379912 | 2381812 | cRMPc14105 | 37676 | 606 | 2352501 | 2390177 |  |  |  | cRMPs1426 | 156242 | 606 | 2233935 | 2390177 |  |
| 12 | Pc.c000903278.Contig1 | 2937 | - | 2390783 | 2394197 |  |  |  |  |  |  |  |  |  |  |  |  |  |  |
| man12 | MALPY00115 - Ins | 17776 | - | 2390824 | 2407849 |  |  |  |  |  |  | 369 | 4300 |  |  |  |  |  |  |
| 12 | Pb.c000301394.Contig1 | 3607 | + | 2390986 | 2395110 |  |  |  |  |  |  |  |  |  |  |  |  |  |  |
| 12 | PcContig587 | 2537 | - | 2394369 | 2396996 |  |  |  |  |  |  |  |  |  |  |  |  |  |  |
| 12 | Pb.c001101896.Contig1 | 6421 | + | 2394701 | 2401182 |  |  |  |  |  |  |  |  |  |  |  |  |  |  |
| 12 | PcContig713 | 3324 | - | 2396678 | 2400113 |  |  |  |  |  |  |  |  |  |  |  |  |  |  |
| 12 | Pc.c001203831.Contig1 | 5083 | - | 2400626 | 2405589 |  |  |  |  |  |  |  |  |  |  |  |  |  |  |
| 12 | Pb.c001101970.Contig1 | 3941 | + | 2401469 | 2405284 | cRMPc14106 | 17066 | 253 | 2390783 | 2407849 |  |  |  |  |  |  |  |  |  |
| 12 | PcContig163 | 4592 | - | 2408102 | 2413014 |  |  |  |  |  |  |  |  |  |  |  |  |  |  |
| 12 | MALPY00646 | 4756 | + | 2412205 | 2416964 |  |  |  |  |  |  | 369 | 1100 |  |  |  |  |  |  |
| 12 | Pc.c001505546.Contig1 | 907 | - | 2413023 | 2413933 | cRMPc14107 | 8862 | 481 | 2408102 | 2416964 |  |  |  |  |  |  |  |  |  |
| 12 | MALPY03156 | 611 | - | 2417445 | 2418056 |  |  |  |  |  |  | 369 | 700 |  |  |  |  |  |  |
| 12 | Pb.c001101594.Contig1 | 8063 | - | 2417698 | 2429528 |  |  |  |  |  |  |  |  |  |  |  |  |  |  |
| 12 | Pc.c001606657.Contig1 | 1260 | - | 2417898 | 2419062 |  |  |  |  |  |  |  |  |  |  |  |  |  |  |
| JC12 | MALPY02395 | 3724 | - | 2418653 | 2422377 |  |  |  |  |  |  | 369 | 3000 |  |  |  |  |  |  |
| 12 | PcContig469 | 2061 | - | 2426705 | 2428820 |  |  |  |  |  |  |  |  |  |  |  |  |  |  |
| 12 | MALPY00419 | 7267 | + | 2427796 | 2436021 |  |  |  |  |  | 2 | 369 |  |  |  |  |  |  |  |
| 12 | Pc.c000403070.Contig1 | 573 | - | 2428394 | 2429018 |  |  |  |  |  |  |  |  |  |  |  |  |  |  |
| 12 | Pc.c000002617.Contig1 | 2257 | - | 2428490 | 2431340 |  |  |  |  |  |  |  |  |  |  |  |  |  |  |
| 12 | Pb.c000901813.Contig1 | 3150 | - | 2431054 | 2434333 |  |  |  |  |  |  |  |  |  |  |  |  |  |  |
| 12 | Pc.c001404703.Contig1 | 2618 | - | 2432034 | 2434868 |  |  |  |  |  |  |  |  |  |  |  |  |  |  |
| 12 | Pb.c000901396.Contig1 | 2800 | - | 2434158 | 2437283 |  |  |  |  |  |  |  |  |  |  |  |  |  |  |
| 12 | MALPY01035 | 3848 | + | 2435091 | 2438815 |  |  |  |  |  | 2 | 145 | 1200 |  |  |  |  |  |  |
| 12 | Pc.c001606247.Contig1 | 695 | - | 2435128 | 2435823 |  |  |  |  |  |  |  |  |  |  |  |  |  |  |
| 12 | PcContig420 | 2501 | - | 2436044 | 2438539 | cRMPc14108 | 21370 | 161 | 2417445 | 2438815 |  |  |  |  |  |  |  |  |  |
| 12 | MALPY00106 | 4014 | - | 2438976 | 2443005 |  |  |  |  |  |  | 145 | 7000 |  |  |  |  |  |  |
| 12 | Pb.c001602072.Contig2 | 3687 | + | 2439389 | 2443091 |  |  |  |  |  |  |  |  |  |  |  |  |  |  |
| 12 | Pc.c000402790.Contig1 | 1791 | - | 2441137 | 2442958 | cRMPc14109 | 4115 | 8935 | 2438976 | 2443091 |  |  |  |  |  |  |  |  |  |
| 12 | Pc.c000002090.Contig2 | 6323 | + | 2452026 | 2458487 |  |  |  |  |  |  |  |  |  |  |  |  |  |  |
| 12 | MALPY00815 | 23913 | + | 2452913 | 2475968 |  |  |  |  |  |  | 145 | 2000 |  |  |  |  |  |  |
| 12 | Pb.c001001682.Contig1 | 3859 | + | 2453453 | 2457537 |  |  |  |  |  |  |  |  |  |  |  |  |  |  |
| 12 | Pc.c000002657.Contig1 | 1986 | + | 2459178 | 2461164 |  |  |  |  |  |  |  |  |  |  |  |  |  |  |
| 12 | Pb.c001301807.Contig1 | 3491 | - | 2459630 | 2463121 |  |  |  |  |  |  |  |  |  |  |  |  |  |  |
| 12 | Pc.c001304654.Contig1 | 1207 | + | 2461530 | 2462737 |  |  |  |  |  |  |  |  |  |  |  |  |  |  |
| 12 | PcContig651 | 2248 | - | 2465703 | 2467972 |  |  |  |  |  |  |  |  |  |  |  |  |  |  |
| 12 | Pb.c001904393.Contig1 | 1368 | - | 2471596 | 2472973 |  |  |  |  |  |  |  |  |  |  |  |  |  |  |
| 12 | Pc.c000302939.Contig1 | 4548 | + | 2471699 | 2475710 |  |  |  |  |  |  |  |  |  |  |  |  |  |  |
| 12 | PbContig344 | 4851 | - | 2473116 | 2477393 | cRMPc14110 | 25367 | 861 | 2452026 | 2477393 |  |  |  |  |  |  |  |  |  |
| 12 | MALPY00778 | 7157 | + | 2478254 | 2485408 |  |  |  |  |  |  | 145 | 2400 |  |  |  |  |  |  |
| 12 | Pb.c001301878.Contig1 | 3145 | - | 2481832 | 2484973 |  |  |  |  |  |  |  |  |  |  |  |  |  |  |
| 12 | Pc.c001304502.Contig1 | 2538 | - | 2482816 | 2485350 | cRMPc14111 | 7154 | 553 | 2478254 | 2485408 |  |  |  |  |  |  |  |  |  |
| 12 | Pb.c001101850.Contig1 | 5153 | - | 2485961 | 2491468 |  |  |  |  |  |  |  |  |  |  |  |  |  |  |
| 12 | MALPY00490 | 8371 | - | 2487860 | 2497668 |  |  |  |  |  | 381 | 145 |  |  |  |  |  |  |  |
| 12 | Pc.c000803898.Contig1 | 1325 | + | 2489938 | 2491620 |  |  |  |  |  |  |  |  |  |  |  |  |  |  |
| 12 | Pb.c000902079.Contig1 | 3099 | + | 2494127 | 2497220 |  |  |  |  |  |  |  |  |  |  |  |  |  |  |
| 12 | Pc.c000602995.Contig1 | 4192 | + | 2494733 | 2498919 |  |  |  |  |  |  |  |  |  |  |  |  |  |  |
| 12 | Pb.c002005906.Contig1 | 3023 | - | 2497707 | 2500751 |  |  |  |  |  |  |  |  |  |  |  |  |  |  |
| 12 | MALPY02110 | 7645 | + | 2498312 | 2506058 |  |  |  |  |  | 381 | 181 | 8000 |  |  |  |  |  |  |
| 12 | Pc.c001003354.Contig1 | 2692 | + | 2499539 | 2502771 |  |  |  |  |  |  |  |  |  |  |  |  |  |  |
| 12 | Pb.c002113570.Contig1 | 1197 | - | 2501438 | 2502638 | cRMPc14112 | 20097 | 9449 | 2485961 | 2506058 |  |  |  |  |  |  |  |  |  |
| 12 | PcContig659 | 5719 | - | 2515507 | 2521963 |  |  |  |  |  |  |  |  |  |  |  |  |  |  |
| 12 | MALPY02331 | 4237 | - | 2516384 | 2521126 |  |  |  |  |  |  | 181 | 1800 |  |  |  |  |  |  |
| 12 | Pb.c001101987.Contig1 | 3082 | - | 2518323 | 2521894 |  |  |  |  |  |  |  |  |  |  |  |  |  |  |
| 12 | MALPY01701 | 3018 | - | 2521896 | 2524911 |  |  |  |  |  |  | 181 | 3500 |  |  |  |  |  |  |
| 12 | Pc.c001708062.Contig1 | 1809 | - | 2522087 | 2523888 | cRMPc14113 | 9404 | 1506 | 2515507 | 2524911 |  |  |  |  |  |  |  |  |  |
| 12 | Pc.c000102698.Contig1 | 3245 | + | 2526417 | 2529609 |  |  |  |  |  |  |  |  |  |  |  |  |  |  |
| 12 | Pb.c001702741.Contig1 | 2151 | + | 2528105 | 2530198 |  |  |  |  |  |  |  |  |  |  |  |  |  |  |
| 12 | MALPY01052 | 2634 | + | 2528496 | 2531274 |  |  |  |  |  |  | 181 | 1300 |  |  |  |  |  |  |
| 12 | Pc.c000103137.Contig1 | 626 | + | 2529592 | 2530206 | cRMPc14114 | 4857 | 1445 | 2526417 | 2531274 |  |  |  |  |  |  |  |  |  |
| 12 | MALPY02265 | 3264 | - | 2532719 | 2535983 |  |  |  |  |  | 83 | 181 |  |  |  |  |  |  |  |
| 12 | Pb.c000701570.Contig1 | 7572 | + | 2535038 | 2542887 |  |  |  |  |  |  |  |  |  |  |  |  |  |  |
| 12 | MALPY00322 | 2985 | - | 2535876 | 2538987 |  |  |  |  |  | 83 | 438A | 2800 |  |  |  |  |  |  |
| 12 | Pc.c000203307.Contig1 | 1253 | - | 2537190 | 2538533 |  |  |  |  |  |  |  |  |  |  |  |  |  |  |
| 12 | MALPY00178 | 4839 | - | 2538155 | 2543045 |  |  |  |  |  | 83 | 480 - NS | 7000 |  |  |  |  |  |  |
| 12 | Pc.c000202858.Contig1 | 1384 | - | 2538611 | 2540067 |  |  |  |  |  |  |  |  |  |  |  |  |  |  |
| 12 | Pc.c001304169.Contig1 | 2392 | - | 2540222 | 2542614 | cRMPc14115 | 10326 | 5723 | 2532719 | 2543045 |  |  |  |  |  |  |  |  |  |
| 12 | PcContig406 | 5455 | - | 2548768 | 2554420 |  |  |  |  |  |  |  |  |  |  |  |  |  |  |
| 12 | Pb.c000901453.Contig1 | 3079 | + | 2549070 | 2552143 |  |  |  |  |  |  |  |  |  |  |  |  |  |  |
| 12 | MALPY00353 | 7426 | - | 2550045 | 2557504 | cRMPc14116 | 8736 | 238 | 2548768 | 2557504 | 1192 | 480 - NS |  |  |  |  |  |  |  |
| 12 | MALPY02828 | 2261 | - | 2557742 | 2560090 |  |  |  |  |  | 1192 | 35 | 2000 |  |  |  |  |  |  |
| 12 | Pc.c001708789.Contig1 | 1468 | - | 2558417 | 2559993 | cRMPc14117 | 2348 | 2162 | 2557742 | 2560090 |  |  |  |  |  |  |  |  |  |
| 12 | PcContig762 | 4834 | + | 2562252 | 2567110 |  |  |  |  |  |  |  |  |  |  |  |  |  |  |
| 12 | Pb.c001602440.Contig1 | 1749 | + | 2565866 | 2567603 |  |  |  |  |  |  |  |  |  |  |  |  |  |  |
| man12 | MALPY00460 | 3565 | - | 2566210 | 2570063 |  |  |  |  |  | 729 |  |  |  |  |  |  |  |  |
| 12 | Pc.c000202727.Contig1 | 4197 | - | 2569454 | 2573490 |  |  |  |  |  |  |  |  |  |  |  |  |  |  |
| 12 | Pb.c000401534.Contig1 | 6332 | - | 2570043 | 2576225 |  |  |  |  |  |  |  |  |  |  |  |  |  |  |
| 12 | MALPY00489 | 11680 | + | 2570092 | 2581715 |  |  |  |  |  | 729 | 35 |  |  |  |  |  |  |  |
| 12 | Pc.c001204031.Contig1 | 1488 | - | 2573705 | 2575193 |  |  |  |  |  |  |  |  |  |  |  |  |  |  |
| 12 | Pb.c000401533.Contig1 | 4375 | + | 2576320 | 2580671 |  |  |  |  |  |  |  |  |  |  |  |  |  |  |
| man12 | PcContig6848 | 4622 | - | 2580089 | 2584711 |  |  |  |  |  |  |  |  |  |  |  |  |  |  |
| man12 | PbContig4101 | 3143 | + | 2582281 | 2585424 | cRMPc14118 | 23172 |  | 2562252 | 2585424 |  |  |  | cRMPs1427 | 194641 |  | 2390783 | 2585424 |  |
| Telomere | |  |  |  |  |  |  |  |  |  |  |  |  |  |  |  |  |  |  |
|  |  | Pb Pc Py contig |  |  |  |  | cRMP contig | Gap to next cRMP contig |  |  |  |  |  |  | cRMP scaffold | Gap to next cRMP scaffold |  |  |  |
|  | Number | 1047 |  |  |  | Number | 118 | 117 |  |  |  |  |  | Number | 27 | 26 |  |  |  |
|  | Average size (bp) | 4575 |  |  |  | Average size (bp) | 20263 | 1661 |  |  |  |  |  | Average size (bp) | 93468 | 2376 |  |  |  |
|  | Minimum size (bp) | 109 |  |  |  | Minimum size (bp) | 816 | 23 |  |  |  |  |  | Minimum size (bp) | 4482 | 31 |  |  |  |
|  | Maximum size (bp) | 31838 |  |  |  | Maximum size (bp) | 121043 | 9449 |  |  |  |  |  | Maximum size (bp) | 248566 | 8939 |  |  |  |
|  | Total size (bp) | 4789518 |  |  |  | Total size (bp) | 2391063 | 194361 |  |  |  |  |  | Total size (bp) | 2523644 | 61780 |  |  |  |
|  |  |  |  |  |  |  |  |  |  |  |  |  |  |  |  |  |  |  |  |
