# Supplementary material for: A Plasmodium Whole-Genome Synteny Map: Indels and Synteny Breakpoints as Foci for Species-Specific Genes
Source: PLoS Pathog. 2005 Dec 23;1(4):e44. doi: 10.1371/journal.ppat.0010044 (PMC1317653; doi:10.1371/journal.ppat.0010044)
Supplement: Supplementary file 15 [file ppat.0010044.st031.doc]

Table S31 - Centromere predictions


|  |  |  |  |  |  |  |  |  |  |  |  |  |
| --- | --- | --- | --- | --- | --- | --- | --- | --- | --- | --- | --- | --- |
| **Table S31:** Centromere predictions^a^. | | | | | | | | | | | | |
| **Pf**  **chr** | Pf gene downstream | **RMP gene downstream** | **RMP contig downstream** | **End of synteny** | **Pf gene upstream** | **RMP gene upstream** | **RMP contig upstream** | **Start of synteny** | **Distance between flanking syntenic genes** | **Method of linkage** | **Size of PCR fragment (bp)** | **RMP**  **chr** |
| 1 | PFA0585w | PY01445 | MALPY00384 | 457,239 | PFA0590w | NI | not syntenic | - | - | SLE | - | 2 |
| 2 | PFB0490c | PB000558.01.0 | Pb.c001904474. Contig1 | 443,244 | PFB0495w | PY05091 | MALPY01600 | 449,666 | 6,422 | PCR | 4,000 | 3 |
| 3 | PFC0610c | PY02287 | MALPY00628 | 587,740 | PFC0615w | PY07280 | MALPY02636 | 601,130 | 13,390 | None | - | 4 |
| 4 | PFD0690c | PY00550 | MALPY00150 | 641,902 | PFD0695w | PY02205 | MALPY00605 | 659,376 | 17,474 | None | - | 7 |
| 5 | chr5-tRNA-Leu-1 | tRNA-Leu-1 | MALPY02997 | 452,699 | PFE0530w | PY04900 | MALPY01520 | 458,119 | 5,420 | PCR | 4,000 | 11 |
| 6 | MAL6P1.152 | PY04637 | MALPY01429 | 1,151,208 | MAL6P1.151 | PY02706 | MALPY00748 | 1,152,410 | 1,202 | None | - | 11 |
| **6^b^** | **MAL6P1.116** | **PY03119** | **MALPY00884** | **526,726** | **MAL6P1.309** | **PY02914** | **MALPY00821** | **528,552** | **1,826** | None | **-** | **1** |
| 7 | PF07\_0050 | NI | not syntenic | 411,168 | MAL7P1.52 | NI | not syntenic | 517,160 | 105,992 | None | - | SBP |
| **7^b^** | **MAL7P1.91** | **PY02993** | **MALPY00846** | **708,966** | **MAL7P1.92** | **PY05845** | **MALPY01919** | **714,099** | **5,133** | None | **-** | **6** |
| 8 | MAL8P1.136 | PY05152 | MALPY01625 | 1,020,763 | PF08\_0118 | PY05558 | MALPY01784 | 1,023,858 | 3,095 | None | - | 12 |
| 9 | PFI1500w | PY06205 | MALPY02081 | 1,240,525 | PFI1505c | PY07561 | MALPY02813 | 1,242,181 | 1,656 | PCR | 2,200 | 8 |
| 10 | PF10\_0216 | PY07622 | MALPY02840 | 934,786 | PF10\_0217 | PY01659 | MALPY00449 | 939,652 | 4,866 | PCR | 3,500 | 5 |
| 11 | PF11\_0226 | PY06052 | MALPY02015 | 826,272 | PF11\_0227 | PY06967 | MALPY02459 | 831,942 | 5,670 | PCR | 2,000 | 9 |
| 12 | PFL1505c | PY05255 | MALPY01663 | 1,282,467 | PFL1510c | PY05931 | MALPY01956 | 1,285,021 | 2,554 | PCR | 3,500 | 14 |
| 13^b^ | **MAL13P1.151** | **PY04447** | **MALPY01358** | **1,200,716** | **PF13\_0157** | **PY01467** | **MALPY00394** | **1,205,374** | **4,658** | **PCR** | **4,000** | **13** |
| 14 | PF14\_0252 | PY05795 | MALPY01896 | 1,068,157 | PF14\_0253 | PY02120 | MALPY00583 | 1,075,409 | 7,252 | PCR | 9,000 | 10 |
| ^a^ Predictions of the location of centromeres in the genome of *P. falciparum* and RMP based on comparison of the synteny blocks. For all previous centromere predictions (Hall *et al.* 2002), the flanking *P. falciparum* genes and their positions on the chromosome as well as the RMP genes and contigs both up- and downstream of the centromere are given.  ^b^ Newly predicted centromeres are shown in bold.   Pf = *P. falciparum*; chr = chromosome; RMP = rodent malaria parasites; NI = not identified; SLE = subtelomere linked end; SBP =� synteny breakpoint | | | | | | | | | | | | |
