# Supplementary material for: A Plasmodium Whole-Genome Synteny Map: Indels and Synteny Breakpoints as Foci for Species-Specific Genes
Source: PLoS Pathog. 2005 Dec 23;1(4):e44. doi: 10.1371/journal.ppat.0010044 (PMC1317653; doi:10.1371/journal.ppat.0010044)
Supplement: Supplementary file 16 [file ppat.0010044.st032.doc]

Table S32 - RMP intersyntenic genes


|  |  |  |  |  |  |  |  |  |  |  |  |  |
| --- | --- | --- | --- | --- | --- | --- | --- | --- | --- | --- | --- | --- |
| **Table S32:** Characteristics of the 5 RMP-specific intersyntenic genes.   A list of 5 RMP-specific genes located in indels between SBs (intersyntenic genes). Proteins with a TM domain in the first 100 amino acids (TM-N) are considered proteins potentially targeted to the surface membrane of the parasite or erythrocyte (1 of 5 genes marked with an asterisk). | | | | | | | | | | | | |
| **RMP chr** | **Pb gene** | **Pb contig** | **Pc gene** | **Pc contig** | **Py gene** | **Py contig** | **Product** | **Size (aa)** | **SP** | **AP** | **TM-N** | **TM** |
| 5 | *c-rrna* gene unit | Contig5161 | *c-rrna* gene unit | Contig4882 | *c-rrna* gene unit | MALPY00527 | 28S, 5.8S, 18S� rRNA | - | **-** | **-** | **-** | **-** |
| 6 | PB101816.00.0 | Pb.Contig31 | PC404234.00.0 | Pch1282h06.q1k UNCHAB.0.29007 | not annotated | MALPY02445 | hypothetical protein | 203 | **-** | **X** | **-** | **-** |
| 10 | not annotated | Pb.Contig126 | not annotated | Pch1539b06.q1k UNCHAB.0.23105 | PY02305 | MALPY00633 | hypothetical protein | 46 | **-** | **-** | **-** | **-** |
| 12 | NI | Pb.c001702793.  Contig1 | NI | no contig | PY01538 | MALPY00409 | hypothetical protein | 73 | **-** | **-** | **-** | **-** |
| 12 \* | PB108348.00.0 - PB000847.03.0 | Pb.c000301471.  Contig1.0/1 | not annotated | Pc.Contig140 | PY01786 | MALPY00483 | dentin phosphoryn, putative | 965 | **-** | **-** | **X** | **X** |
| Abbreviations: RMP, rodent malaria parasites; SB, synteny block; chr, chromosome; Pb, *P. berghei*; Pc, *P. chabaudi*; Py, *P. yoelii*; aa, amino acids; SP, predicted signal peptide; AP, predicted apicoplast transit peptide; TM, transmembrane domain; TM-N, N-terminal TM-domain; NI, not identified. | | | | | | | | | | | | |
