# Supplementary material for: A Plasmodium Whole-Genome Synteny Map: Indels and Synteny Breakpoints as Foci for Species-Specific Genes
Source: PLoS Pathog. 2005 Dec 23;1(4):e44. doi: 10.1371/journal.ppat.0010044 (PMC1317653; doi:10.1371/journal.ppat.0010044)
Supplement: Supplementary file 17 [file ppat.0010044.st033.doc]

Table S33 - Pf intersyntenic genes


|  |  |  |  |  |  |  |  |  |  |
| --- | --- | --- | --- | --- | --- | --- | --- | --- | --- |
| **Table S33:** Characteristics of the 53 *P. falciparum*-specific intersyntenic genes.   A list of 53 *P. falciparum*-specific genes located in indels between SBs (intersyntenic genes) including all intersyntenic pseudogenes. Groups of genes forming a cluster (8 indels) are separated by a black line Proteins with a TM domain in the first 100 amino acids (TM-N) as well as *var* and *rif* genes are considered proteins potentially targeted to the surface membrane of the parasite or erythrocyte (37 of 42 genes and 11 of 11 pseudogenes marked with an asterisk, genes with a PEXEL/VTS (Marti *et al.* 2004; Hiller *et al.* 2004) are marked with two asterisks). | | | | | | | | | |
| **Pf gene** | **Product** | ***pf-fam*****^a^** | **Size indel (bp)** | **trans.** | **exp.** | **SP** | **AP** | **TM-N** | **TM** |
| PFD0615c \*\* | erythrocyte membrane protein 1 (PfEMP1) | *var* | 70,195 |  |  |  |  |  |  |
| chr4.phat\_143/  chr4.glm\_148 \* | var internal cluster associated repeat gene 4a | *vicar* | 70,195 | - | - | **X** | **-** | **X** | **X** |
| PFD0620c \*\* | rifin--related protein | *rif* | 70,195 |  |  |  |  |  |  |
| PFD0625c \*\* | erythrocyte membrane protein 1 (PfEMP1) | *var* | 70,195 |  |  |  |  |  |  |
| - \* | vicar pseudogene | *vicar* | 70,195 |  |  |  |  |  |  |
| PFD0630c \*\* | erythrocyte membrane protein 1 (PfEMP1) | *var* | 70,195 |  |  |  |  |  |  |
| - \* | vicar pseudogene | *vicar* | 70,195 |  |  |  |  |  |  |
| PFD0635c \*\* | erythrocyte membrane protein 1 (PfEMP1) | *var* | 70,195 |  |  |  |  |  |  |
| PFD0640c \*\* | rifin | *rif* | 70,195 |  |  |  |  |  |  |
| PFD0645w \*\* | rifin | *rif* | 70,195 |  |  |  |  |  |  |
| PFD0650w | hypothetical protein | - | 70,195 | - | - | **-** | **-** | **-** | **-** |
| PFD0655w \*\* | var-related pseudogene | *var* | 70,195 |  |  |  |  |  |  |
| MAL7P1.39 \* | var internal cluster associated repeat gene 7a | *vicar* | 105,155 | S,M | - | **X** | **-** | **X** | **X** |
| PF07\_0048 \*\* | erythrocyte membrane protein 1 (PfEMP1) | *var* | 105,155 |  |  |  |  |  |  |
| chr7.phat\_103/  chr7.glm\_101 \* | var internal cluster associated repeat gene 7b | *vicar* | 105,155 | - | - | **X** | **-** | **X** | **X** |
| MAL7P1.43 \*\* | rif pseudogene | *rif* | 105,155 |  |  |  |  |  |  |
| PF07\_0049 \*\* | erythrocyte membrane protein 1 (PfEMP1) | *var* | 105,155 |  |  |  |  |  |  |
| chr7.phat\_108/  chr7.glm\_105 \* | var internal cluster associated repeat gene 7c | *vicar* | 105,155 | - | - | **X** | **-** | **X** | **X** |
| MAL7P1.47 \*\* | rif pseudogene | *rif* | 105,155 |  |  |  |  |  |  |
| MAL7P1.50 \*\* | erythrocyte membrane protein 1 (PfEMP1) | *var* | 105,155 |  |  |  |  |  |  |
| PF07\_0050 \*\* | erythrocyte membrane protein 1 (PfEMP1) | *var* | 105,155 |  |  |  |  |  |  |
| MAL7P1.52 \*\* | rif pseudogene | *rif* | 105,155 |  |  |  |  |  |  |
| PF07\_0051 \*\* | erythrocyte membrane protein 1 (PfEMP1) | *var* | 105,155 |  |  |  |  |  |  |
| chr7.phat\_114 \* | vicar pseudogene | *vicar* | 105,155 |  |  |  |  |  |  |
| MAL7P1.55 \*\* | erythrocyte membrane protein 1 (PfEMP1) | *var* | 105,155 |  |  |  |  |  |  |
| MAL7P1.56 \*\* | erythrocyte membrane protein 1 (PfEMP1) | *var* | 105,155 |  |  |  |  |  |  |
| MAL7P1.57 \*\* | rifin | *rif* | 105,155 |  |  |  |  |  |  |
| MAL7P1.58 \* | hypothetical protein, conserved in Pf | *pfmc-2tm* | 105,155 | ET | - | **-** | **-** | **X** | **X** |
| MAL7P1.59 | hypothetical protein, conserved in Pf | - | 105,155 | - | - | **-** | **-** | **-** | **-** |
| MAL7P1.60 \*\* | var fragment, pseudogene | *var* | 105,155 |  |  |  |  |  |  |
| MAL7P1.62 \*\* | var fragment, pseudogene | *var* | 105,155 |  |  |  |  |  |  |
| MAL7P1.63 \*\* | var fragment, pseudogene | *var* | 105,155 |  |  |  |  |  |  |
| PF07\_0107 \* | hypothetical protein | - | 13,799 | M-LR | - | **-** | **-** | **X** | **X** |
| MAL7P1.144 \*\* | pftstk7a | *tstk* | 13,799 | ER-LT,G | S | **-** | **-** | **-** | **X** |
| PF08\_0103 \*\* | erythrocyte membrane protein 1 (PfEMP1) | *var* | 43,407 |  |  |  |  |  |  |
| - \* | vicar pseudogene | *vicar* | 43,407 |  |  |  |  |  |  |
| PF08\_0104 \*\* | rifin | *rif* | 43,407 |  |  |  |  |  |  |
| PF08\_0105 \*\* | rifin | *rif* | 43,407 |  |  |  |  |  |  |
| PF08\_0106 \*\* | erythrocyte membrane protein 1 (PfEMP1) | *var* | 43,407 |  |  |  |  |  |  |
| PF08\_0107 \*\* | erythrocyte membrane protein 1 (PfEMP1) | *var* | 43,407 |  |  |  |  |  |  |
| chr8.phat\_232/  chr8.glm\_253 \* | var internal cluster associated repeat gene 8 | *vicar* | 43,407 | - | - | **X** | **-** | **X** | **X** |
| PFI0330c | hypothetical protein | - | 11,729 | LR-LT | - | **-** | **-** | **-** | **X** |
| PF10\_0159 \*\* | glycophorin-binding protein 130 precursor | *gbp* | 37,843 | ER-LS | T | **-** | **-** | **X** | **X** |
| PF10\_0160 \*\* | 3.8 protein - pftstk10a | *tstk* | 37,843 | ER-ES | T | **-** | **-** | **X** | **X** |
| PF10\_0161 | hypothetical protein | - | 37,843 | ER-LR | S | **-** | **-** | **-** | **-** |
| PF10\_0162 | hypothetical protein | - | 37,843 | ET,M | - | **-** | **-** | **-** | **-** |
| PF10\_0163 \*\* | hypothetical protein | - | 37,843 | LS-ER | - | **X** | **X** | **X** | **X** |
| PF10\_0164 \* | hypothetical protein | *etramp* | 37,843 | S,G | G | **X** | **-** | **X** | **X** |
| MAL13P1.268 \* | hypothetical protein | - | 18,712 | M-ER | - | **X** | **-** | **X** | **X** |
| MAL13P1.269 \* | hypothetical protein | - | 18,712 | B,G | T | **-** | **-** | **X** | **X** |
| PF13\_0275 \*\* | hypothetical protein | - | 18,712 | M-ET | T,G,M | **-** | **-** | **X** | **X** |
| PF13\_0276 \* | hypothetical protein | - | 18,712 | M-LR | - | **-** | **-** | **X** | **X** |
| PF14\_0708 \* | hypothetical protein | - | 13,194 | G | - | **-** | **-** | **X** | **X** |
| ^a^ References to the papers describing the gene families are as follows: *var* (Baruch *et al.* 1995; Smith *et al.* 1995; Su *et al.* 1995); *vicar*, *var* internal cluster associated repeat gene (this paper); *rif* (Kyes *et al.* 1999); *pfmc-2tm*, *P. falciparum* Maurer�s cleft localized 2 transmembrane domain proteins (Sam-Yellowe *et al.* 2004); *tstk*, transforming growth factor β (TGF-β) receptor-like serine/threonine protein kinases (this paper); *gbp*, glycophorin-binding protein (Nolte *et al.* 1991; Perkins *et al.* 1984; Rudolph *et al.* 1994); *etramp*, early transcribed membrane proteins (Spielmann *et al.* 2003).   Abbreviations:SB, synteny block; PEXEL, *Plasmodium* export element; VTS, vacuolar transport signal; Pf, *P. falciparum*; *pf-fam*, described *P. falciparum* gene family; SP, predicted signal peptide; AP, predicted apicoplast transit peptide; TM, transmembrane domain; TM-N, N-terminal TM-domain; trans., approximate stage of (highest) transcription for all genes except the *var*, *rif* and *vicar* genes based on transcriptome data (Le Roch *et al.* 2003; Bozdech *�et al.* 2003); M, merozoite; ER, early ring; LR, late ring; ET, early trophozoite; LT, late trophozoite; ES, early schizont; LS, late schizont; G, gametocyte; S, sporozoite; B, asexual blood stages; exp., expression of those genes based on proteome data (Florens *et al.* 2002; Lasonder *et al.* 2002); M, merozoite; T, trophozoite; G, gametocyte; S, sporozoite. | | | | | | | | | |
