# Supplementary material for: A Plasmodium Whole-Genome Synteny Map: Indels and Synteny Breakpoints as Foci for Species-Specific Genes
Source: PLoS Pathog. 2005 Dec 23;1(4):e44. doi: 10.1371/journal.ppat.0010044 (PMC1317653; doi:10.1371/journal.ppat.0010044)
Supplement: Supplementary file 18 [file ppat.0010044.st034.doc]

Table S34 - Pf intrasyntenic genes


|  |  |  |  |  |  |  |  |  |  |
| --- | --- | --- | --- | --- | --- | --- | --- | --- | --- |
| **Table S34:** Characteristics of the 138 *P. falciparum*-specific intrasyntenic genes.   A list of 138 *P. falciparum*-specific genes located in indels within SBs (intrasyntenic genes) including all intrasyntenic pseudogenes. Groups of genes forming a cluster (82 indels) are separated by a black line. Proteins with a TM domain in the first 100 amino acids (TM-N) as well as *var* and *rif* genes are considered proteins potentially targeted to the surface membrane of the parasite or erythrocyte (78 of 126 genes and 11 of 12 pseudogenes marked with an asterisk, genes with a PEXEL/VTS (Marti *et al.* 2004; Hiller *et al.* 2004) are marked with two asterisks). | | | | | | | | | |
| **Pf gene** | **Product** | ***pf-fam*****^a^** **or homologue** | **Size indel (bp)** | **trans.** | **exp.** | **SP** | **AP** | **TM-N** | **TM** |
| PFA0360c \*\* | hypothetical protein | PFA0365c | 2,246 | S,G | - | **X** | **X** | **X** | **X** |
| PFA0365c \* | hypothetical protein | PFA0360c | 2,246 | - | - | **X** | **X** | **X** | **X** |
| PFB0140w \* | hypothetical protein | - | 1,237 | LT-LS | - | **-** | **-** | **X** | **X** |
| PFB0225c | hypothetical protein | - | 1,526 | G | S | **-** | **-** | **-** | **-** |
| PFB0300c \* | merozoite surface protein 2 precursor | *msp* | 2,805 | ES-M | M | **X** | **X** | **X** | **X** |
| PFB0305c \* | merozoite surface protein 5 | *msp* | 2,805 | LS-M | - | **X** | **-** | **X** | **X** |
| PFB0340c \* | cysteine protease, putative | *proteases* | 22,130 | ES-LS | M | **X** | **-** | **X** | **X** |
| PFB0345c \* | cysteine protease, putative | *proteases* | 22,130 | LT-LS | S | **X** | **-** | **X** | **X** |
| PFB0350c \* | cysteine protease, putative | *proteases* | 22,130 | LT-LS | G,T | **X** | **-** | **X** | **X** |
| PFB0355c | cysteine protease, putative | *proteases* | 22,130 | ES-LS,S | - | **-** | **-** | **-** | **X** |
| PFB0360c \* | cysteine protease, putative | *proteases* | 22,130 | ES-LS | - | **X** | **-** | **X** | **X** |
| PFB0540w | hypothetical protein | - | 5,534 | LT-ES | S | **-** | **-** | **-** | **-** |
| PFB0650w | hypothetical protein | - | 7,502 | LT-M | - | **-** | **-** | **-** | **-** |
| PFB0695c \* | acyl-CoA synthetase | *acyl-CoA* | 2,666 | M-LR | - | **X** | **X** | **X** | **X** |
| PFC0110w \* | cytoadherence linked asexual protein | *clag* | 21,201 | ES-M | - | **X** | **-** | **X** | **X** |
| PFC0115w \*\* | pseudo var gene | *var* | 21,201 |  |  |  |  |  |  |
| PFC0120w \* | cytoadherence linked asexual protein | *clag* | 21,201 | ES-M | M | **X** | **-** | **X** | **X** |
| PFC0215c \* | hypothetical protein | - | 1,856 | - | - | **X** | **X** | **X** | **X** |
| PFC0440c | helicase, putative | - | 6,809 | LR-ES | S,T | **-** | **-** | **-** | **X** |
| PFD0765w | RING finger protein, putative | - | 2,627 | M | M,S | **-** | **-** | **-** | **X** |
| PFD0995c \*\* | erythrocyte membrane protein 1 (PfEMP1) | *var* | 52,417 |  |  |  |  |  |  |
| \* | vicar pseudogene | *vicar* | 52,417 |  |  |  |  |  |  |
| PFD1000c \*\* | erythrocyte membrane protein 1 (PfEMP1) | *var* | 52,417 |  |  |  |  |  |  |
| \* | vicar pseudogene | *vicar* | 52,417 |  |  |  |  |  |  |
| PFD1005c \*\* | erythrocyte membrane protein 1 (PfEMP1) | *var* | 52,417 |  |  |  |  |  |  |
| PFD1010w \*\* | rifin | *rif* | 52,417 |  |  |  |  |  |  |
| PFD1015c \*\* | erythrocyte membrane protein 1 (PfEMP1) | *var* | 52,417 |  |  |  |  |  |  |
| chr4.phat\_229 \* | var internal cluster associated repeat gene 4b | *vicar* | 52,417 | - | - | **X** | **-** | **X** | **X** |
| PFD1020c \*\* | rifin | *rif* | 52,417 |  |  |  |  |  |  |
| PFD1025c \*\* | var pseudogene | *var* | 52,417 |  |  |  |  |  |  |
| PFE0080c \* | rhoptry-associated protein 2 | PFE0075c | 1,196 | ES-M | M | **X** | **-** | **X** | **X** |
| PFE0125w \* | hypothetical protein | - | 1,784 | M,G | - | **X** | **-** | **X** | **X** |
| PFE0805w \* | cation-transporting ATPase 1 | - | 7,375 | LR-ES,G | S | **-** | **-** | **X** | **X** |
| PFE1055c | hypothetical protein | - | 1,690 | ER-ES,G | - | **-** | **-** | **-** | **-** |
| PFE1325w | hypothetical protein | - | 13,574 | - | - | **-** | **-** | **-** | **X** |
| PFE1455w \* | sugar transporter, putative | - | 2,359 | S | - | **-** | **-** | **X** | **X** |
| MAL6P1.49 | DNA helicase, putative | - | 5,493 | ET | M,T | **-** | **-** | **-** | **X** |
| MAL6P1.71 \* | hypothetical protein | - | 899 | - | - | **X** | **-** | **X** | **X** |
| MAL6P1.99 | hypothetical protein | - | 3,961 | - | M | **-** | **-** | **-** | **X** |
| MAL6P1.108 | calcium-dependent protein kinase | - | 1,907 | ET-LS,G | - | **-** | **-** | **-** | **X** |
| MAL6P1.252 \*\* | erythrocyte membrane protein 1 (PfEMP1) | *var* | 19,289 |  |  |  |  |  |  |
| MAL6P1.251 \*\* | rifin | *rif* | 19,289 |  |  |  |  |  |  |
| MAL6P1.250 \*\* | rifin | *rif* | 19,289 |  |  |  |  |  |  |
| MAL6P1.170 | hypothetical protein | - | 1,928 | S | - | **-** | **-** | **-** | **X** |
| MAL6P1.156 \* | troponin c-like protein, putative | - | 1,248 | - | - | **-** | **-** | **X** | **X** |
| MAL7P1.97 \* | hypothetical protein | - | 904 | - | - | **-** | **-** | **X** | **X** |
| MAL7P1.167 | hypothetical protein | - | 8,321 | LS | S | **-** | **-** | **-** | **-** |
| MAL8P1.97 | hypothetical protein | - | 4,830 | S,G,M | - | **-** | **-** | **-** | **X** |
| MAL8P1.111 | hypothetical protein | - | 4,204 | B,G | G | **-** | **-** | **-** | **X** |
| MAL8P1.126 \* | serine protease, putative | - | 3,139 | S | - | **X** | **-** | **X** | **X** |
| MAL8P1.155 \* | hypothetical protein | - | 1,292 | S | - | **-** | **-** | **X** | **X** |
| PFI0405w \* | hypothetical protein | - | 10,089 | LT-LS | - | **X** | **X** | **X** | **X** |
| PFI0410c | hypothetical protein | - | 10,089 | ES-LS | T | **-** | **-** | **-** | **X** |
| PFI0510c \* | hypothetical protein | - | 5,093 | LS | S | **-** | **-** | **X** | **X** |
| PF10\_0309 | hypothetical protein (helicase, putative) | - | 3,616 | S,B,G | - | **-** | **-** | **-** | **X** |
| PF10\_0342 \* | hypothetical protein | - | 19,110 | ES-M,G | - | **X** | **-** | **X** | **X** |
| PF10\_0343 \* | S-antigen | - | 19,110 | ES-M | - | **X** | **-** | **X** | **X** |
| PF10\_0344 \* | glutamate-rich protein | - | 19,110 | ES-M | G,S | **X** | **X** | **X** | **X** |
| PF10\_0345 \* | merozoite surface protein 3 | *msp* | 19,110 | ES-M | M,T | **X** | **-** | **X** | **X** |
| PF10\_0346 \* | merozoite surface protein 6 | *msp* | 19,110 | LS-M | M | **-** | **-** | **X** | **X** |
| PF10\_0347 \* | hypothetical protein | *msp* | 19,110 | ES-M | - | **-** | **-** | **X** | **X** |
| PF10\_0362 \* | DNA polymerase zeta catalytic subunit, putative | *msp* | 7,204 | LT-LS | - | **-** | **-** | **X** | **X** |
| PF11\_0161 \* | falcipain-2 precursor, putative | *proteases* | 1,448 | ER-LT | T | **X** | **X** | **X** | **X** |
| PF11\_0165 \* | hypothetical protein | *proteases* | 5,073 | LR-LT | T | **X** | **X** | **X** | **X** |
| PF11\_0166 | hypothetical protein | - | 5,073 | LS-LR | M,T | **-** | **-** | **-** | **-** |
| PF11\_0186 \* | hypothetical protein | - | 756 | B,G | S | **-** | **-** | **X** | **X** |
| PF11\_0211 \* | hypothetical protein, conserved | - | 1,348 | M-LR | - | **-** | **-** | **X** | **X** |
| PF11\_0326 | hypothetical protein | - | 8,291 | - | S | **-** | **-** | **-** | **X** |
| PF11\_0357 | hypothetical protein | - | 5,168 | G | M | **-** | **-** | **-** | **-** |
| PFL0105w | hypothetical protein | - | 2,136 | G | M,G,T | **-** | **-** | **-** | **-** |
| PFL0305c | hypothetical protein | - | 2,422 | G,S | G | **-** | **-** | **-** | **-** |
| PFL0360c | hypothetical protein | - | 8,171 | LT | - | **-** | **-** | **-** | **X** |
| PFL0935c \*\* | erythrocyte membrane protein 1 (PfEMP1) | *var* | 16,848 |  |  |  |  |  |  |
| PFL0940c \*\* | var fragment, pseudogene | *var* | 16,848 |  |  |  |  |  |  |
| PFL0945w \*\* | var fragment, pseudogene | *var* | 16,848 |  |  |  |  |  |  |
| PFL1060c \*\* | hypothetical protein, conserved | - | 1,709 | - | - | **X** | **X** | **X** | **X** |
| PFL1075w | Apicomplexan AP2-integrase DNA binding domain containing protein | *apiap2* | 7,676 | ET-LS,G | G,S | **-** | **-** | **-** | **X** |
| PFL1265c | hypothetical protein | - | 1,751 | G | - | **-** | **-** | **-** | **-** |
| PFL1945c \* | hypothetical protein | *etramp* | 58,313 | M | - | **X** | **X** | **X** | **X** |
| PFL1950w \*\* | erythrocyte membrane protein 1 (PfEMP1) | *var* | 58,313 |  |  |  |  |  |  |
| PFL1955w \*\* | erythrocyte membrane protein 1 (PfEMP1) | *var* | 58,313 |  |  |  |  |  |  |
| chr12.phat\_410/  chr12.glm\_457 \* | var internal cluster associated repeat gene 12 | *vicar* | 58,313 | - | - | **X** | **-** | **X** | **X** |
| PFL1960w \*\* | erythrocyte membrane protein 1 (PfEMP1) | *var* | 58,313 |  |  |  |  |  |  |
| PFL1965w \*\* | rif pseudogene | *rif* | 58,313 |  |  |  |  |  |  |
| \* | vicar pseudogene | *vicar* | 58,313 |  |  |  |  |  |  |
| PFL1970w \*\* | var pseudogene | *var* | 58,313 |  |  |  |  |  |  |
| \* | vicar pseudogene | *vicar* | 58,313 |  |  |  |  |  |  |
| PFL2085w | hypothetical protein | - | 1,331 | LT-M | S | **-** | **-** | **-** | **-** |
| PFL2255w | hypothetical protein | - | 1,819 | G,S | - | **-** | **-** | **-** | **-** |
| PF13\_0071 | hypothetical protein | - | 1,688 | ET-LT,G | S | **-** | **-** | **-** | **X** |
| MAL13P1.58 | hypothetical protein | - | 30,826 | ER-LT | - | **-** | **-** | **-** | **-** |
| PF13\_0073 \*\* | hypothetical protein | - | 30,826 | ER-ET | T | **X** | **-** | **X** | **X** |
| MAL13P1.59 \* | hypothetical protein | *pf-fam-f* | 30,826 | - | - | **-** | **-** | **X** | **X** |
| MAL13P1.60 \* | erythrocyte binding antigen 140 | *dbl-ebp* | 30,826 | LS-M | M,T | **X** | **-** | **X** | **X** |
| MAL13P1.61 \*\* | hypothetical protein | - | 30,826 | M-LR | - | **X** | **X** | **X** | **X** |
| PF13\_0074 | hypothetical protein | *pf-fam-b* | 30,826 | M | - | **-** | **-** | **-** | **X** |
| PF13\_0075 | hypothetical protein, conserved in Pf | *pf-fam-b* | 30,826 | M-ER | - | **-** | **-** | **-** | **-** |
| MAL13P1.62 \* | hypothetical protein | - | 30,826 | - | - | **X** | **X** | **X** | **X** |
| PF13\_0076 \*\* | hypothetical protein | - | 30,826 | M-ET | - | **X** | **-** | **X** | **X** |
| MAL13P1.106 \* | hypothetical protein | - | 21,907 | G | G | **X** | **-** | **X** | **X** |
| MAL13P1.107 \* | hypothetical protein | - | 21,907 | ET-LT | G,S | **X** | **-** | **X** | **X** |
| PF13\_0115 \* | frameshifted ebl1, pseudogene | *dbl-ebp* | 21,907 | LS-M | - | **X** | **X** | **X** | **X** |
| MAL13P1.109 \*\* | pftstk13 | *tstk* | 21,907 | - | - | **X** | **-** | **X** | **X** |
| MAL13P1.110 \* | hypothetical protein | - | 21,907 | LR-LT | - | **X** | **-** | **X** | **X** |
| MAL13P1.122 | hypothetical protein | - | 8,478 | S,B,G | T | **-** | **-** | **-** | **-** |
| PF13\_0127 | hypothetical protein | - | 1,538 | LT-LS | - | **-** | **-** | **-** | **X** |
| PF13\_0153 | hypothetical protein | - | 3,014 | S,B,G | M | **-** | **-** | **-** | **X** |
| PF13\_0191 \* | hypothetical protein | *msp* | 10,811 | ET-LT | S | **X** | **X** | **X** | **X** |
| PF13\_0192 \* | hypothetical protein | *msp* | 10,811 | ET-ES | - | **X** | **-** | **X** | **X** |
| PF13\_0193 \* | MSP7-like protein | *msp* | 10,811 | ES-LS | - | **X** | **-** | **X** | **X** |
| PF13\_0194 \* | hypothetical protein | - | 10,811 | M-LR | T | **X** | **-** | **X** | **X** |
| PF13\_0195 | MSP7 fragment, pseudogene | *msp* | 10,811 | S,B,G | - | **-** | **-** | **-** | **-** |
| MAL13P1.176 | reticulocyte binding protein 2 homologue b | PF13\_0198 | 14,894 | LS-M | - | **-** | **-** | **-** | **X** |
| PF13\_0198 \* | reticulocyte binding protein 2 homologue a | MAL13P1.176 | 14,894 | LS-M | S,T | **-** | **-** | **X** | **X** |
| MAL13P1.197 | hypothetical protein | - | 1,092 | - | - | **-** | **-** | **-** | **-** |
| MAL13P1.214 | phosphoethanolamine N-methyltransferase, putative | - | 1,219 | S,B,G | M | **-** | **-** | **-** | **-** |
| MAL13P1.235 \* | hypothetical protein | - | 1,227 | - | - | **-** | **-** | **X** | **X** |
| PF13\_0295 | hypothetical protein | - | 1,870 | G,S | - | **-** | **-** | **-** | **-** |
| MAL13P1.295 | hypothetical protein | - | 6,149 | ET-ES,G | S | **-** | **-** | **-** | **X** |
| MAL13P1.303 \* | polyadenylate binding protein, putative | - | 2,259 | ET-LS,G | G,T,M | **-** | **-** | **X** | **X** |
| MAL13P1.306 | hypothetical protein | - | 2,491 | ES-M | - | **-** | **-** | **-** | **-** |
| PF13\_0338 \* | hypothetical protein | - | 2,390 | LT-LS,G | M | **X** | **-** | **X** | **X** |
| MAL13P1.325 | hypothetical protein | - | 1,094 | ET-LT | - | **-** | **-** | **-** | **-** |
| PF14\_0036 \* | acid phosphatase, putative | - | 1,728 | S,B,G | G,T,M | **-** | **-** | **X** | **X** |
| PF14\_0076 \* | plasmepsin 1 precursor | *Proteases* | 10,526 | M-LR | T,G,M | **X** | **X** | **X** | **X** |
| PF14\_0077 \* | plasmepsin 2 precursor | *Proteases* | 10,526 | LR-LT | T,M,G | **-** | **-** | **X** | **X** |
| PF14\_0078 \* | HAP protein | *Proteases* | 10,526 | ER-ES | T,G,M | **X** | **X** | **X** | **X** |
| PF14\_0119 \* | hypothetical protein, conserved | PF14\_0117 | 962 | ES-M | G,S | **X** | **-** | **X** | **X** |
| PF14\_0206 | hypothetical protein | - | 2,861 | G | - | **-** | **-** | **-** | **X** |
| PF14\_0236 | hypothetical protein | - | 5,345 | G | S,M | **-** | **-** | **-** | **-** |
| PF14\_0262 \* | hypothetical protein | - | 6,925 | S | - | **-** | **-** | **X** | **X** |
| PF14\_0263 | hypothetical protein | - | 6,925 | - | G | **-** | **-** | **-** | **X** |
| PF14\_0291 | hypothetical protein | - | 3,677 | M-LR | - | **-** | **-** | **-** | **X** |
| PF14\_0297 \* | ecto-nucleoside triphosphate diphosphohydrolase 1, putative | - | 2,846 | ET-ES | - | **X** | **X** | **X** | **X** |
| PF14\_0463 | chloroquine resistance marker protein | - | 11,385 | LT,LS | S,M | **-** | **-** | **-** | **X** |
| PF14\_0565 | hypothetical protein | - | 2,867 | B,G | M | **-** | **-** | **-** | **-** |
| PF14\_0594 | hypothetical protein | - | 9,968 | M | S | **-** | **-** | **-** | **-** |
| PF14\_0638 \* | hypothetical protein | - | 2,582 | - | G | **X** | **-** | **X** | **X** |
| ^a^ In the column *pf-fam* or homologue, various gene families are listed as well as the gene names of single homologous genes. References to the papers describing the gene families are as follows: *msp*, merozoite surface proteins (Cowman *et al.* 2000); *proteases* (Bourgon *et al.* 2004); *acyl-CoA*, acyl-CoA synthetases (Hall *et al.* 2005); *clag*, cytoadherence-linked asexual gene (Holt *et al.* 1999); *var* (Baruch *et al.* 1995; Smith *et al.* 1995; Su *et al.* 1995); *vicar*, *var* internal cluster associated repeat gene (this paper); *rif* (Kyes *et al.* 1999); *apiap2* (Balaji *et al.* 2005); *etramp* (Spielmann *et al.* 2003); *pf-fam-f*, *P. falciparum* gene family f (Hall *et al.* 2005); *dbl-ebp*, Duffy-binding-like erythrocyte-binding proteins (Adams *et al.* 1992); *pf-fam-b*, *P. falciparum* gene family b (Hall *et al.* 2005); *tstk*, transforming growth factor β (TGF-β) receptor-like serine/threonine protein kinases (this paper).   Abbreviations: SB, synteny block; PEXEL, *Plasmodium* export element; VTS, vacuolar transport signal; Pf, *P. falciparum*; *pf-fam*, described *P. falciparum* gene family; SP, predicted signal peptide; AP, predicted apicoplast transit peptide; TM, transmembrane domain; TM-N, N-terminal TM-domain; trans., approximate stage of (highest) transcription for all genes except the *var*, *rif* and *vicar* genes based on transcriptome data (Le Roch *et al.* 2003; Bozdech *et al.* 2003; S, sporozoite; M, merozoite; ER, early ring; LR, late ring; ET, early trophozoite; LT, late trophozoite; ES, early schizont; LS, late schizont; B, asexual blood stages; G, gametocyte; exp., expression of those genes based on proteome data (Florens *�et al.* 2002; Lasonder *et al.* 2002); M, merozoite; T, trophozoite; G, gametocyte; S, sporozoite. | | | | | | | | | |
