# Supplementary material for: A Plasmodium Whole-Genome Synteny Map: Indels and Synteny Breakpoints as Foci for Species-Specific Genes
Source: PLoS Pathog. 2005 Dec 23;1(4):e44. doi: 10.1371/journal.ppat.0010044 (PMC1317653; doi:10.1371/journal.ppat.0010044)
Supplement: Supplementary file 19 [file ppat.0010044.st036.doc]

Table S36 - GRIMM analysis


## Input file GRIMM analysis of *Plasmodium* synteny map

\*\*\*\*\*\*\*\*\*\*\*\*\*\*\*\*\*\*\*\*\*\*\*\*\*\*\*\*\*\*\*\*\*\*\*\*\*\*\*\*\*\*\*\*\*\*\*\*\*\*\*\*\*\*\*\*\*\*\*\*\*\*\*\*\*\*\*\*\*\*\*\*\*\*\*\*\*\*\*\*\*\*\*\*\*\*\*\*\*\*\*\*\*\*\*\*\*\*\*\*\*\*\*\*\*\*\*\*\*\*\*\*\*\*\*

#
Chromosomes are in order 1 -> 14

#
Numbers refer to the extremes of synteny blocks, uninterupted by species
specific genes.

#
Numbers 1-14 represent the CAT regions or putative centromeres

>PbGenome

15 1
16 $

2 17
18   19 20 $

21 3
22 $

23 4
24   25 26 $

27 5
28   29 30 $

31
32   33 34   35 36   37 6 38 $

39
40   41 7 42 $

43
44   45 46   47 8 48 $

49 9
50 $

51
52   53 10 54 $

55
11 56   57 58   59 60 $

61
62   63 64   65 66   67 12 68   69 70  
71 72 $

73
74   75 76   77 13 78 $

79
80   81 82   83 84   85 14 86 $

>PfGenome

-18
-17 -2 $

21 3
22 $

23 4
24   -46 -45   -64 -63 $

51
52   35 36   41 7 42   29 30 $

55
11 56   71 72 $

15 1
16   69 70   57 58 $

43
44   65 66   81 82   37 6 38   19 20 $

39
40   83 84   67 12 68 $

-26
-25   47 8 48 $

61
62   27 5 28 $

49 9
50 $

31
32   85 14 86 $

79
80   77 13 78   59 60 $

-54
-10 -53   -34 -33   75 76  
-74 -73 $

\*\*\*\*\*\*\*\*\*\*\*\*\*\*\*\*\*\*\*\*\*\*\*\*\*\*\*\*\*\*\*\*\*\*\*\*\*\*\*\*\*\*\*\*\*\*\*\*\*\*\*\*\*\*\*\*\*\*\*\*\*\*\*\*\*\*\*\*\*\*\*\*\*\*\*\*\*\*\*\*\*\*\*\*\*\*\*\*\*\*\*\*\*\*\*\*\*\*\*\*\*\*\*\*\*\*\*\*\*\*\*\*\*\*\*

 

## One optimal rearrangement scenario

Pb
to Pf

14
chromosomes, 86 genes, 28
caps      Multichromosomal Distance: 16

---

| **St** | **Desc** | **Before** | **After** |
| --- | --- | --- | --- |
| **0** | **Pb**  **Gen** | C_87_ -38 -6 -37 -36 -35 -34 -33 -32 -31 -C_109_; C_111_ 79 80 81 82 83 84 85 14 86 C_110_; -C_108_ -50 -9 -49 -C_107_; -C_106_ -42 -7 -41 -40 -39 -C_101_; -C_100_ -20 -19 -18 -17 -2 C_88_; C_89_ 21 3 22 C_90_; -C_92_ 27 5 28 29 30 C_94_; C_95_ 55 11 56 57 58 59 60 C_112_; C_113_ -54 -10 -53 -52 -51 -C_93_; C_91_ 23 4 24 25 26 -C_103_; -C_102_ -16 -1 -15 -C_97_; -C_96_ -72 -71 -70 -69 -68 -12 -67 -66 -65 -64 -63 -62 -61 -C_105_; -C_104_ -48 -8 -47 -46 -45 -44 -43 -C_99_; -C_98_ -78 -13 -77 -76 -75 -74 -73 C_114_ |  |
| **1** | **Rev** | -C_98_ -78 -13 -77 -76 -75 -74 -73 C_114_ | -C_98_ -78 -13 -77 75 76 -74 -73 C_114_ |
| **2** | **Tra** | C_87_ -38 -6 -37 -36 -35 -34 -33 -32 -31 -C_109_; C_111_ 79 80 81 82 83 84 85 14 86 C_110_ | C_87_ -38 -6 -37 -36 -35 -34 -33 -80 -79 -C_111_; C_109_ 31 32 81 82 83 84 85 14 86 C_110_ |
| **3** | **Tra** | C_87_ -38 -6 -37 -36 -35 -34 -33 -80 -79 -C_111_; C_109_ 31 32 81 82 83 84 85 14 86 C_110_ | C_87_ -38 -6 -37 -82 -81 -32 -31 -C_109_; C_111_ 79 80 33 34 35 36 83 84 85 14 86 C_110_ |
| **4** | **Tra** | C_87_ -38 -6 -37 -82 -81 -32 -31 -C_109_; C_111_ 79 80 33 34 35 36 83 84 85 14 86 C_110_ | C_87_ -38 -6 -37 -82 -81 -84 -83 -36 -35 -34 -33 -80 -79 -C_111_; C_109_ 31 32 85 14 86 C_110_ |
| **5** | **Tra** | -C_106_ -42 -7 -41 -40 -39 -C_101_; -C_92_ 27 5 28 29 30 C_94_ | -C_106_ -28 -5 -27 C_92_; C_101_ 39 40 41 7 42 29 30 C_94_ |
| **6** | **Tra** | C_87_ -38 -6 -37 -82 -81 -84 -83 -36 -35 -34 -33 -80 -79 -C_111_; -C_88_ 2 17 18 19 20 C_100_ | C_87_ -18 -17 -2 C_88_; C_111_ 79 80 33 34 35 36 83 84 81 82 37 6 38 19 20 C_100_ |
| **7** | **Tra** | -C_92_ 27 5 28 C_106_; -C_96_ -72 -71 -70 -69 -68 -12 -67 -66 -65 -64 -63 -62 -61 -C_105_ | -C_92_ 63 64 65 66 67 12 68 69 70 71 72 C_96_; -C_106_ -28 -5 -27 -62 -61 -C_105_ |
| **8** | **Tra** | -C_92_ 63 64 65 66 67 12 68 69 70 71 72 C_96_; -C_112_ -60 -59 -58 -57 -56 -11 -55 -C_95_ | -C_92_ 63 64 65 66 67 12 68 69 70 57 58 59 60 C_112_; -C_96_ -72 -71 -56 -11 -55 -C_95_ |
| **9** | **Tra** | -C_92_ 63 64 65 66 67 12 68 69 70 57 58 59 60 C_112_; -C_102_ -16 -1 -15 -C_97_ | -C_92_ 63 64 65 66 67 12 68 C_102_; -C_112_ -60 -59 -58 -57 -70 -69 -16 -1 -15 -C_97_ |
| **10** | **Tra** | -C_92_ 63 64 65 66 67 12 68 C_102_; -C_100_ -20 -19 -38 -6 -37 -82 -81 -84 -83 -36 -35 -34 -33 -80 -79 -C_111_ | -C_92_ 63 64 65 66 81 82 37 6 38 19 20 C_100_; -C_102_ -68 -12 -67 -84 -83 -36 -35 -34 -33 -80 -79 -C_111_ |
| **11** | **Tra** | -C_92_ 63 64 65 66 81 82 37 6 38 19 20 C_100_; -C_104_ -48 -8 -47 -46 -45 -44 -43 -C_99_ | -C_92_ 63 64 45 46 47 8 48 C_104_; -C_100_ -20 -19 -38 -6 -37 -82 -81 -66 -65 -44 -43 -C_99_ |
| **12** | **Tra** | C_111_ 79 80 33 34 35 36 83 84 67 12 68 C_102_; -C_94_ -30 -29 -42 -7 -41 -40 -39 -C_101_ | C_111_ 79 80 33 34 35 36 41 7 42 29 30 C_94_; -C_102_ -68 -12 -67 -84 -83 -40 -39 -C_101_ |
| **13** | **Tra** | -C_92_ 63 64 45 46 47 8 48 C_104_; C_91_ 23 4 24 25 26 -C_103_ | -C_92_ 63 64 45 46 -24 -4 -23 -C_91_; -C_104_ -48 -8 -47 25 26 -C_103_ |
| **14** | **Tra** | C_93_ 51 52 53 10 54 -C_113_; -C_94_ -30 -29 -42 -7 -41 -36 -35 -34 -33 -80 -79 -C_111_ | C_93_ 51 52 35 36 41 7 42 29 30 C_94_; C_113_ -54 -10 -53 -34 -33 -80 -79 -C_111_ |
| **15** | **Tra** | C_97_ 15 1 16 69 70 57 58 59 60 C_112_; -C_98_ -78 -13 -77 75 76 -74 -73 C_114_ | C_97_ 15 1 16 69 70 57 58 C_98_; -C_112_ -60 -59 -78 -13 -77 75 76 -74 -73 C_114_ |
| **16** | **Tra** | C_111_ 79 80 33 34 53 10 54 -C_113_; -C_112_ -60 -59 -78 -13 -77 75 76 -74 -73 C_114_ | C_111_ 79 80 77 13 78 59 60 C_112_; C_113_ -54 -10 -53 -34 -33 75 76 -74 -73 C_114_ |
| **-** | **Pf**  **Gen** |  | C_87_ -18 -17 -2 C_88_; C_89_ 21 3 22 C_90_; -C_92_ 63 64 45 46 -24 -4 -23 -C_91_; C_93_ 51 52 35 36 41 7 42 29 30 C_94_; C_95_ 55 11 56 71 72 C_96_; C_97_ 15 1 16 69 70 57 58 C_98_; C_99_ 43 44 65 66 81 82 37 6 38 19 20 C_100_; C_101_ 39 40 83 84 67 12 68 C_102_; C_103_ -26 -25 47 8 48 C_104_; C_105_ 61 62 27 5 28 C_106_; C_107_ 49 9 50 C_108_; -C_110_ -86 -14 -85 -32 -31 -C_109_; C_111_ 79 80 77 13 78 59 60 C_112_; C_113_ -54 -10 -53 -34 -33 75 76 -74 -73 C_114_ |

 

## One optimal rearrangement scenario

Pf
to Pb

14
chromosomes, 86 genes, 28
caps      Multichromosomal Distance: 16

---

|  |  |  |  |  |
| --- | --- | --- | --- | --- |
|  |  |  | **Affected chromosomes** | |
| **Step** | **Description** |  | **Before** | **After** |
| **0** | **(PfGenome)** |  | -C_88_ -68 -12 -67 -84 -83 -40 -39 -C_99_; -C_98_ -18 -17 -2 -C_89_; C_87_ 15 1 16 69 70 57 58 C_112_; C_113_ 79 80 77 13 78 59 60 C_108_; C_109_ 61 62 27 5 28 C_100_; C_101_ 43 44 65 66 81 82 37 6 38 19 20 C_90_; C_91_ 21 3 22 C_92_; C_93_ 23 4 24 -46 -45 -64 -63 -C_95_; -C_94_ -26 -25 47 8 48 C_102_; C_103_ 49 9 50 C_104_; -C_106_ -54 -10 -53 -34 -33 75 76 -74 -73 -C_111_; -C_110_ -72 -71 -56 -11 -55 -C_107_; C_105_ 51 52 35 36 41 7 42 29 30 C_96_; C_97_ 31 32 85 14 86 C_114_ |  |
| **1** | **Reversal** |  | -C_106_ -54 -10 -53 -34 -33 75 76 -74 -73 -C_111_ | -C_106_ -54 -10 -53 -34 -33 -76 -75 -74 -73 -C_111_ |
| **2** | **Translocation** |  | -C_88_ -68 -12 -67 -84 -83 -40 -39 -C_99_; C_87_ 15 1 16 69 70 57 58 C_112_ | -C_88_ -16 -1 -15 -C_87_; C_99_ 39 40 83 84 67 12 68 69 70 57 58 C_112_ |
| **3** | **Translocation** |  | C_99_ 39 40 83 84 67 12 68 69 70 57 58 C_112_; -C_106_ -54 -10 -53 -34 -33 -76 -75 -74 -73 -C_111_ | C_99_ 39 40 83 84 33 34 53 10 54 C_106_; -C_112_ -58 -57 -70 -69 -68 -12 -67 -76 -75 -74 -73 -C_111_ |
| **4** | **Translocation** |  | C_89_ 2 17 18 C_98_; -C_90_ -20 -19 -38 -6 -37 -82 -81 -66 -65 -44 -43 -C_101_ | C_89_ 2 17 18 19 20 C_90_; -C_98_ -38 -6 -37 -82 -81 -66 -65 -44 -43 -C_101_ |
| **5** | **Translocation** |  | C_93_ 23 4 24 -46 -45 -64 -63 -C_95_; -C_94_ -26 -25 47 8 48 C_102_ | C_93_ 23 4 24 25 26 C_94_; C_95_ 63 64 45 46 47 8 48 C_102_ |
| **6** | **Translocation** |  | C_95_ 63 64 45 46 47 8 48 C_102_; -C_98_ -38 -6 -37 -82 -81 -66 -65 -44 -43 -C_101_ | C_95_ 63 64 65 66 81 82 37 6 38 C_98_; -C_102_ -48 -8 -47 -46 -45 -44 -43 -C_101_ |
| **7** | **Translocation** |  | C_95_ 63 64 65 66 81 82 37 6 38 C_98_; -C_108_ -60 -59 -78 -13 -77 -80 -79 -C_113_ | C_95_ 63 64 65 66 77 13 78 59 60 C_108_; -C_98_ -38 -6 -37 -82 -81 -80 -79 -C_113_ |
| **8** | **Translocation** |  | C_95_ 63 64 65 66 77 13 78 59 60 C_108_; -C_112_ -58 -57 -70 -69 -68 -12 -67 -76 -75 -74 -73 -C_111_ | C_95_ 63 64 65 66 77 13 78 C_112_; -C_108_ -60 -59 -58 -57 -70 -69 -68 -12 -67 -76 -75 -74 -73 -C_111_ |
| **9** | **Translocation** |  | C_95_ 63 64 65 66 77 13 78 C_112_; -C_108_ -60 -59 -58 -57 -70 -69 -68 -12 -67 -76 -75 -74 -73 -C_111_ | C_95_ 63 64 65 66 67 12 68 69 70 57 58 59 60 C_108_; -C_112_ -78 -13 -77 -76 -75 -74 -73 -C_111_ |
| **10** | **Translocation** |  | C_95_ 63 64 65 66 67 12 68 69 70 57 58 59 60 C_108_; -C_110_ -72 -71 -56 -11 -55 -C_107_ | C_95_ 63 64 65 66 67 12 68 69 70 71 72 C_110_; -C_108_ -60 -59 -58 -57 -56 -11 -55 -C_107_ |
| **11** | **Translocation** |  | C_95_ 63 64 65 66 67 12 68 69 70 71 72 C_110_; -C_100_ -28 -5 -27 -62 -61 -C_109_ | C_95_ 27 5 28 C_100_; -C_110_ -72 -71 -70 -69 -68 -12 -67 -66 -65 -64 -63 -62 -61 -C_109_ |
| **12** | **Translocation** |  | -C_106_ -54 -10 -53 -34 -33 -84 -83 -40 -39 -C_99_; C_105_ 51 52 35 36 41 7 42 29 30 C_96_ | -C_106_ -54 -10 -53 -34 -33 -84 -83 -36 -35 -52 -51 -C_105_; C_99_ 39 40 41 7 42 29 30 C_96_ |
| **13** | **Translocation** |  | -C_106_ -54 -10 -53 -34 -33 -84 -83 -36 -35 -52 -51 -C_105_; C_113_ 79 80 81 82 37 6 38 C_98_ | -C_106_ -54 -10 -53 -34 -33 -84 -83 -82 -81 -80 -79 -C_113_; C_105_ 51 52 35 36 37 6 38 C_98_ |
| **14** | **Translocation** |  | -C_106_ -54 -10 -53 -34 -33 -84 -83 -82 -81 -80 -79 -C_113_; C_97_ 31 32 85 14 86 C_114_ | -C_106_ -54 -10 -53 -34 -33 -32 -31 -C_97_; C_113_ 79 80 81 82 83 84 85 14 86 C_114_ |
| **15** | **Translocation** |  | C_95_ 27 5 28 C_100_; -C_96_ -30 -29 -42 -7 -41 -40 -39 -C_99_ | C_95_ 27 5 28 29 30 C_96_; -C_100_ -42 -7 -41 -40 -39 -C_99_ |
| **16** | **Translocation** |  | C_97_ 31 32 33 34 53 10 54 C_106_; -C_98_ -38 -6 -37 -36 -35 -52 -51 -C_105_ | C_97_ 31 32 33 34 35 36 37 6 38 C_98_; -C_106_ -54 -10 -53 -52 -51 -C_105_ |
| **-** | **(PbGenome)** |  |  | -C_88_ -16 -1 -15 -C_87_; C_89_ 2 17 18 19 20 C_90_; C_91_ 21 3 22 C_92_; C_93_ 23 4 24 25 26 C_94_; C_95_ 27 5 28 29 30 C_96_; C_97_ 31 32 33 34 35 36 37 6 38 C_98_; C_99_ 39 40 41 7 42 C_100_; C_101_ 43 44 45 46 47 8 48 C_102_; C_103_ 49 9 50 C_104_; -C_106_ -54 -10 -53 -52 -51 -C_105_; C_107_ 55 11 56 57 58 59 60 C_108_; C_109_ 61 62 63 64 65 66 67 12 68 69 70 71 72 C_110_; C_111_ 73 74 75 76 77 13 78 C_112_; C_113_ 79 80 81 82 83 84 85 14 86 C_114_ |

---
